# *In silico* design of a multiepitope subunit vaccine targeting *Salmonella enterica* serovar Infantis: an immunoinformatics and reverse vaccinology approach

**DOI:** 10.3389/fimmu.2026.1717278

**Published:** 2026-02-06

**Authors:** Dhiraj Chundru, Mostafa Ghanem

**Affiliations:** Department of Veterinary Medicine, Virginia-Maryland College of Veterinary Medicine, College Park, MD, United States

**Keywords:** foodborne pathogens, immunoinformatics, multiepitope vaccine, reverse vaccinology (RV), *Salmonella* Infantis

## Abstract

*Salmonella enterica* serovar Infantis is an emerging zoonotic pathogen increasingly linked to poultry and multidrug-resistant human infections. Existing vaccines lack serovar-specific efficacy, underscoring the need for targeted immunization strategies. In this study, we employed an immunoinformatics and reverse vaccinology pipeline to design a multiepitope subunit vaccine (MEV) against *S.* Infantis. A subtractive proteomics analysis of 692 poultry-derived genomes identified CsgA as a highly conserved, immunogenic, and non-host homologous antigen. Selected cytotoxic T lymphocyte (CTL), helper T lymphocyte (HTL), and B-cell epitopes were filtered for antigenicity, immunogenicity, allergenicity, and sequence conservation. The epitopes were assembled with appropriate linkers and a cholera toxin B (CTB) adjuvant. Structural modeling and normal mode analysis confirmed the construct’s stability, while molecular docking predicted high-affinity interactions with chicken TLR2, TLR5, MHC-I, and MHC-II. Immune simulations indicated robust humoral and cellular responses. Codon optimization yielded a codon adaptation index (CAI) of 1.0 and GC content of 54.76%, supporting efficient expression in *E. coli.* The optimized construct was successfully cloned in silico into the pET30a(+) vector. These findings present a rationally designed, computationally validated vaccine candidate with the potential to induce protective immunity against *S.* Infantis, warranting further experimental validation.

## 1 Introduction

*Salmonella enterica* remains a prominent etiological agent of foodborne diseases globally (1). It causes an estimated 93.8 million cases and 155,000 deaths worldwide annually, with contaminated food sources being responsible for over 80 million infections (2). In the United States, *Salmonella* causes 1.35 million infections, 26,500 hospitalizations, and 420 fatalities annually (3). Most salmonellosis cases, particularly gastroenteritis, are caused by non-typhoidal *Salmonella* (NTS) serovars, which dominate global foodborne *Salmonella* outbreaks. Symptoms include diarrhea, fever, and abdominal discomfort (4). Although they are generally self-limiting, individuals with compromised immunity are more susceptible to severe outcomes and may even experience fatal complications (5). Beyond the immediate public health implications, *Salmonella* imposes significant economic burdens, encompassing direct medical expenses, productivity losses, and societal impacts from premature mortality. In 2014, the U.S. Department of Agriculture’s Economic Research Service (ERS) estimated the financial impact of *Salmonella*-related illnesses to be approximately $3.7 billion (6). Similarly, the European Food Safety Authority (EFSA) estimated in 2023 that the annual economic toll of human salmonellosis could reach €3 billion (7).

Animals are primary reservoirs of *Salmonella*, which spreads through food, water, and direct animal contact (8). Poultry products, especially eggs and chicken meat, are common sources of infection (9). Recently, *Salmonella enterica* subsp*. Enterica* serovar Infantis (*S.* Infantis) has gained prominence due to its rising prevalence (10). In the U.S., infections caused by *S.* Infantis surged by 167% between 2011 and 2016, with a significant outbreak in 2018 traced back to contaminated poultry products (10). In Europe, *S.* Infantis was the most commonly detected serovar in broiler chickens and poultry meat in 2018 (10), with similar trends reported in South America and Asia (10). Notably, a substantial proportion of these isolates exhibit multidrug resistance (MDR); nearly 70% of *S.* Infantis strains in the European Union carried pESI-like megaplasmid–associated resistance genes in 2016 (10–13). Collectively, these developments pose a significant threat to both human and animal health and highlight urgent gaps in current surveillance systems and control strategies.

Vaccination is a central component of *Salmonella* control strategies in poultry (14). However, commercial poultry vaccines primarily target significant *Salmonella* serovars like *S.* Typhimurium and *S.* Enteritidis, offering limited or no cross-protection against other serovars such as *S.* Infantis (15, 16). Additionally, live attenuated vaccines carry potential safety concerns, including environmental persistence and risk of reversion to virulence (17). Subunit vaccines offer a safer and more targeted alternative, especially when designed using immunoinformatics to select specific antigenic epitopes (18–20).

Immunoinformatics has become instrumental in modern vaccinology, allowing researchers to simulate immune responses and optimize vaccine candidates before laboratory testing, streamlining early-stage development. Subtractive proteomics is a biologically driven reverse vaccinology strategy that systematically narrows the pathogen proteins to select vaccine-relevant targets by integrating conservation, host non-homology, surface accessibility, and functional relevance (21–23). Within this framework, optimal vaccine candidates are those that are highly conserved across circulating isolates to reduce strain restriction and immune escape, surface-exposed or secreted to ensure accessibility to antibodies and mucosal immune mechanisms (24) and functionally associated with core aspects of host–pathogen interaction such as adhesion, invasion, colonization, or other aspects of virulence (21, 25). Subtractive proteomics further prioritizes proteins with evidence of *in vivo* expression, immune recognition, and links to essential or fitness-associated processes that impose evolutionary constraints on antigen loss or variation (essentiality). By embedding these principles directly into the filtering pipeline, subtractive proteomics enables identification of conserved, functionally relevant targets. Following the selection of antigen or vaccine target, immunoinformatics further facilitates high-throughput screening of candidate epitopes for inclusion in multiepitope vaccines (MEV). These vaccines include Cytotoxic T Lymphocyte (CTL), Helper T Lymphocyte (HTL), and B-cell epitopes that stimulate both cellular and humoral immunity, enhancing immune response specificity and eliminating live vaccines safety concerns. Moreover, reverse vaccinology integrates genomic, structural, and immunological data to predict potent immunogens and construct vaccine candidates in silico (26–28). These computational methods have been successfully employed in the rational design of vaccines against a variety of human and animal pathogens (22, 26, 28–33).

**Figure 1:**
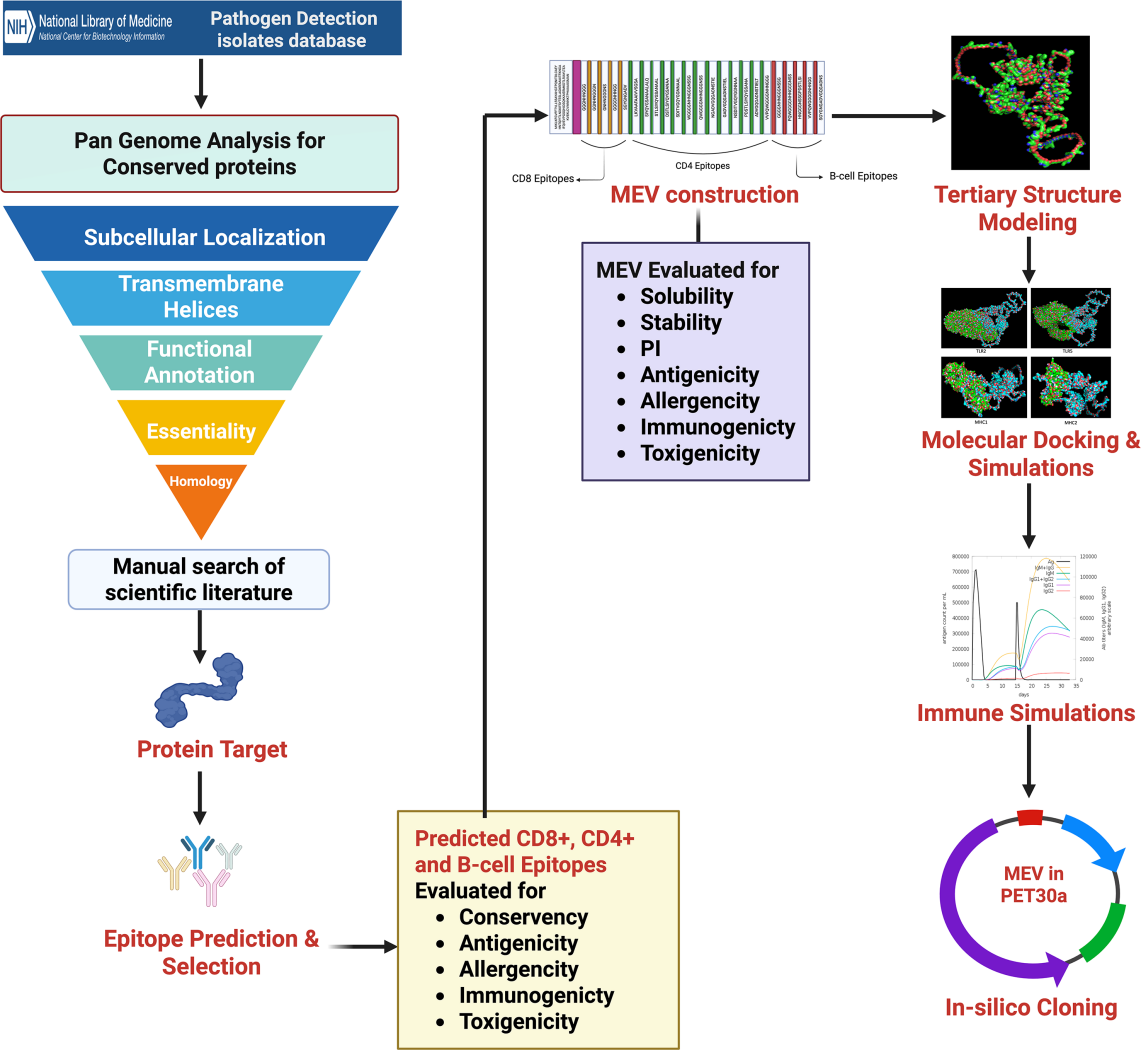
Schematic overview of the pipeline used for the development of a multi-epitope vaccine and validation against *S*. Infantis in poultry.

In this study, we employed an integrated immunoinformatics and reverse vaccinology approach to design a novel multiepitope vaccine targeting *Salmonella enterica* serovar Infantis, a significant and emerging threat to poultry health and food safety. This work addresses the critical gap in serovar-specific poultry vaccines by leveraging conserved antigenic targets identified from genomic data and computationally constructing a rational vaccine candidate with broad immune-stimulatory potential. The vaccine construct was assessed in silico for immunological, structural, and expression parameters, offering a framework for accelerating next-generation poultry vaccine development. An overview of the integrated immunoinformatics and reverse vaccinology workflow employed in this study is illustrated in **Figure 1**.

## 2 Materials and methods

### 2.1 Genome selection

Annotated proteomes of 692 *Salmonella* Infantis genomes were downloaded from the NCBI Isolate Browser on 17 August 2022 (**Supplementary File 1**). Only isolates from the United States, Canada, or Mexico, originating from poultry-related sources labelled as chicken young carcasses, chicken, chicken carcasses, avian caeca, and chicken litter, were included in the analysis.

### 2.2 Protein target identification and filtering

A subtractive proteomics-based in silico screening pipeline was employed to identify conserved, surface-exposed proteins as potential vaccine targets. Proteins present in ≥ 95% of the genomes were considered conserved, as identified using Roary version 3.13.0 (34). The most conserved proteins were chosen, and a consensus sequence was generated for downstream analysis. Subcellular localization was predicted using PSORTb v3.0 (35) (accessed on 17 August 2022), while transmembrane domains and signal peptides were predicted using DeepTMHMM v1.0 (36, 37) and SignalP v4.1 (38) (accessed on 16 August 2022). HMMERSCAN (v2.41.2) was used to annotate conserved domains via PFAM and TIGRFAM databases. Antigenicity was evaluated using VaxiJen v2.0 (accessed on 17 August 2022; threshold = 0.5) (39), and essential genes were prioritized using Geptop 2.0 (accessed on 23 August 2022) (40). Final candidates identified through the subtractive proteomics pipeline were manually reviewed in scientific literature to assess their reported biological functions, involvement in host–pathogen interactions, degree of conservation, and prior evaluation or use as vaccine antigens or immunogenic targets in related pathogens, before selecting the final vaccine candidate.

### 2.3 Epitope prediction and screening

Epitope prediction was conducted for cytotoxic T lymphocytes (CTLs), helper T lymphocytes (HTLs), and B cells. CTL epitopes (9-mers) were predicted using NetCTL v1.2 (41) (accessed on 23 August 2022), filtered for a half-maximal inhibitory concentration (IC_50_) value **< 250** nM and validated via the IEDB MHC-I Binding tool (accessed on 23 August 2022). Immunogenic CTL epitopes were then screened using IEDB Immunogenicity prediction tool.

HTL epitopes (15-mers) were predicted using the IEDB MHC-II binding tool (accessed on 23 August 2022), with the SMM-align method and filtered for their ability to induce both IFN-γ and IL-4 responses using IFNepitope and IL4pred (accessed on 25 August 2022), respectively.

CTL and HTL epitopes were initially predicted using all available HLA alleles to avoid population bias. Following epitope selection, MHC allele associations for each epitope were retrieved from the prediction outputs. In addition, CTL epitopes were evaluated using NetMHCpan v4.2 against all publicly available chicken MHC class I (BF1 and BF2) sequences from the IPD-MHC database (release 3.15.0.0). Given the limited experimental validation of avian MHC binding models and the relatively conserved nature of the chicken MHC complex, these predictions were used to qualitatively assess the breadth of epitope coverage across chicken MHC class I haplotypes rather than to derive quantitative binding conclusions.

B-cell epitopes were predicted using the ABCpred server (accessed on 26 August 2022), with a window size of 12–16 amino acids and a default threshold of 0.51, from amino acids restricted to extracellular regions defined by DeepTMHMM. Chickens produce immunoglobulin Y (IgY), functionally analogous to mammalian IgG. However, as the B-cell epitope prediction tools are based on mammalian immunoglobulin models, the outputs are expressed in terms of IgG. Therefore, throughout this study, references to “IgG” should be interpreted as representing avian IgY in the context of chicken immunology.

### 2.4 Epitope validation and vaccine construction

Shortlisted epitopes were evaluated for antigenicity using VaxiJen v2.0 (39), conservation via the IEDB Epitope Conservancy tool, allergenicity using AllerTOP v2.0 (42), and toxicity using ToxinPred 3.0 (accessed on 09 December 2025) (43). Epitopes that were antigenic, conserved, and non-allergenic were selected for inclusion in the final vaccine construct. CTL, HTL, and B-cell epitopes were joined using AAY, GPGPG, and KK linkers, respectively, to avoid the formation of junctional epitopes (44). A cholera toxin B subunit (CTB, accession no. ABV74245.1) was conjugated to the N-terminal using an EAAK linker to enhance immunogenicity (45).

### 2.5 Antigenicity, and physicochemical characterization of vaccine construct

The vaccine construct was assessed for antigenicity using VaxiJen v2.0 (39) and AntigenPro (46), allergenicity using both AllerTOP v2.0 (42) and AlgPred 2.0 (47), and toxicity ToxinPred 3.0 (43). BLASTp (accessed on 28 August 2022) analysis of homology for vaccine construct was performed against host (*Gallus gallus*) proteome to ensure safety and to mitigate the risk of eliciting an autoimmune response. Physicochemical properties were analyzed using the ProtParam tool (48). Metrics assessed included amino acid composition, molecular weight, theoretical isoelectric point (pI), estimated half-life, instability index, aliphatic index, and GRAVY. Protein solubility was predicted using Protein-Sol (49) (accessed on 20 September 2022).

### 2.6 Structural modeling, refinement and validation

Secondary structure prediction was performed using PSIPRED workbench (accessed on 31 August 2022). Tertiary models were generated using AlphaFold3 (50) (accessed on 9 December 2025), I-TASSER (51) (accessed on 6 September 2022), TrRosetta (52) (accessed on 31 August 2022), and Robetta (53) (accessed on 12 September 2022). Model refinement was conducted using GalaxyRefine server (54), (accessed on 8 September 2022), which was used to improve local geometry and side-chain conformations. Overall model quality and energy profiles were assessed using ProSA-web (55), which estimates the deviation of the total model energy relative to experimentally determined protein structures through Z-score analysis and residue-wise energy plots. Stereochemical quality was further evaluated using the MolProbity server, which generated Ramachandran plots and Rama-Z scores to assess backbone conformational normality. Models with Rama-Z values within the acceptable range (−3 to +3) were considered structurally reliable (56).

### 2.7 Molecular docking and molecular dynamic simulation

Protein-protein docking was conducted using ClusPro 2.0 (57), a widely used rigid-body docking platform that employs fast Fourier transform (FFT)-based sampling to generate and cluster low-energy protein–protein interaction conformations. ClusPro ranks docking poses based on cluster size and weighted interaction energy terms, enabling identification of the most probable binding modes. The vaccine construct was docked against immune receptors relevant to antigen recognition and presentation, including Toll-like receptor 2 (TLR2; PDB ID: 6NIG), Toll-like receptor 5 (TLR5; PDB ID: 3J0A), major histocompatibility complex class I (MHC-I; PDB ID: 4E0R), and major histocompatibility complex class II (MHC-II; PDB ID: 6T3Y). Receptor structures were obtained from the Protein Data Bank (PDB). In cases where experimentally resolved chicken receptor structures were unavailable, homologous receptor structures with high sequence similarity to the corresponding chicken proteins were selected and used as surrogates. The stability of selected docked complexes was further evaluated using PRODIGY (58), which estimates binding free energy and dissociation constants from interfacial contacts. Docking simulations with multiple Toll-like receptors (TLRs) were intended to explore the potential of the structurally engineered vaccine construct to engage innate immune receptors beyond any single ligand–receptor paradigm, recognizing that epitope concatenation and linker incorporation may alter receptor interaction profiles relative to native antigens. Docking with MHC I and II receptors was conducted to evaluate the structural compatibility of the construct with antigen-presentation pathways responsible for CD8^+^ and CD4^+^ T-cell activation, respectively, thereby supporting assessment of the vaccine’s capacity to elicit both cytotoxic and helper T-cell–mediated immune responses. To further assess the stability and collective motions of the docked complexes, molecular dynamics simulation (MDS) and normal mode analysis (NMA) of the docked complexes were performed using the iMODS server (59) (accessed on 16 September 2022 and 09 December 2025). iMODS applies an elastic network model to assess intrinsic flexibility, deformability, and low-frequency collective motions of protein complexes without explicit time-dependent molecular dynamics. In addition, structural deviations associated with dominant normal modes were quantified by calculating root-mean-square deviation (RMSD) values following structural superposition of the initial docked complexes and their corresponding NMA-derived conformers using the Matchmaker tool in UCSF ChimeraX. RMSD analysis was used to assess the extent of conformational displacement induced by collective motions while preserving the overall binding geometry of the complexes. Following this, intermolecular interaction analysis of the top docked complexes was carried out using LigPlot+ 2.3.1 software, enabling visualization of hydrogen bonds and hydrophobic contacts between the vaccine construct and immune receptor residues to characterize binding interfaces at the molecular level.

### 2.8 Immune simulation

The immune response to the final vaccine construct was simulated using the C-ImmSim server (60) (accessed on 15 September 2022). Two parenteral doses were administered on days 1 and 14. The model predicted humoral and cell-mediated immune responses, including antibody titers, B- and T-memory cell formation, and cytokine profiles.

### 2.9 Codon optimization and in silico cloning

Codon optimization for expression in *E. coli* K12 was performed using the JCat server (61) (accessed on 15 September 2022). The gene was in silico cloned into the pET30a(+) expression vector using SnapGene v5.3 by incorporating XhoI and NdeI restriction sites at C-terminus and N-terminus, respectively.

## 3 Results

### 3.1 Identification of vaccine candidate antigen

To identify conserved and immunogenic antigens, 4,583 protein-coding genes from poultry-derived *Salmonella* Infantis genomes were subjected to a subtractive proteomics workflow. Surface-localized and secreted proteins were prioritized using PSORTb 3.0, DeepTMHMM, and SignalP 4.1, resulting in 1,071 candidate proteins. Antigenicity screening using VaxiJen (threshold = 0.89) narrowed the list to 33 highly antigenic proteins (**Supplementary File 2**). Functional annotation with HMMERSCAN against PFAM and TIGRFAM databases identified 888 proteins as potential adhesins, bacteriocins, or signal peptides.

Cross-analysis of antigenicity, localization, and functional domains yielded seven top candidates (**Supplementary File 3**). Geptop 2.0 identified four of these as essential proteins (**Table 1**), with curlin major subunit A (CsgA) receiving the highest essentiality score (1.0). CsgA also ranked as the second most antigenic protein across the core proteome. Global strain coverage of the vaccine target was inferred from sequence conservation analysis. Sequence analysis confirmed that CsgA exhibited complete sequence conservation across all *S.* Infantis isolates included in this study, with no detectable allelic variation (**Supplementary File 4**), supporting broad strain-level coverage of the selected antigen.

**Table 1:** Summary of surface-localized, highly antigenic, and essential *S.* Infantis proteins identified for vaccine development .

No.	Protein name	Vaxijen score	Subcellular location	Essentiality score
1	Curlin major subunit (CsgA)	1.0694	Extracellular	1
2	Flagellar protein (FlhE)	0.9361	Extracellular	0.3
3	Porin (OmpS1)	0.9057	Outer membrane	0.6998
4	Peptidoglycan-associated lipoprotein (Pal)	0.8968	Outer membrane	0.5999

Among the top four candidate proteins identified, CsgA was prioritized based on its well-established functional role in host–pathogen interactions, conservation, and documented innate immunostimulatory properties. As the major structural subunit of curli fimbriae, CsgA is indispensable for *Salmonella* adhesion to the intestinal epithelium and biofilm formation, thereby facilitating persistence and colonization (62). Importantly, CsgA is expressed *in vivo* during infection and biofilm-associated persistence, underscoring its biological relevance during natural host exposure (63). Immunologically, CsgA acts as a pathogen-associated molecular pattern (PAMP) (64) and is recognized through the TLR2/TLR1 axis, with CD14 contributing to innate sensing of bacterial amyloid structures and downstream immune activation (65). Furthermore, genetic deletion of csgA reduced adhesion and invasion and attenuated virulence in oral infection models in chickens, directly linking this antigen to pathogenic fitness in the poultry host (66).

### 3.2 Epitope mapping, validation, and vaccine assembly

#### 3.2.1 Predicted cytotoxic and helper T-cell epitopes and selection

Effective induction of a protective immune response requires the presentation of antigenic peptides to cytotoxic T lymphocytes (CTLs) via MHC class I molecules, enabling targeted elimination of infected cells (67). Therefore, the accurate prediction and selection of CTL epitopes is critical for the immunogenic efficacy of a multi-epitope vaccine construct. Using a combination of NetCTL v1.2 and the IEDB MHC-I tool, 149 candidate CTL 9-mer epitopes were predicted from the CsgA protein. Among these, 79 were identified as immunogenic, and 65 of those also demonstrated antigenicity (**Supplementary Files 5**, **6**). The top five epitopes based on combined antigenicity and immunogenicity scores were selected for inclusion in the final construct.

Helper T lymphocytes (HTLs) play a central role in orchestrating adaptive immunity by promoting B-cell maturation, immunoglobulin class switching, and CTL activation. This coordination is initiated upon recognition of peptides presented via MHC class II molecules (68). Accordingly, robust multi-epitope vaccine design must include HTL epitopes capable of eliciting cytokine-mediated responses. Prediction using the IEDB MHC-II tool yielded 222 HTL epitope candidates. Among these, IL4pred and IFNepitope tools’ analyses identified 22 and 34 cytokine-inducing epitopes, respectively. Thirteen epitopes were dual-positive for IL-4 and IFN-γ induction and met antigenicity criteria; these were selected for incorporation into the vaccine construct (**Supplementary File 7**). Since all the CTL and HTL epitopes were derived from the same highly conserved CsgA protein, antigenic coverage is expected to be uniform across circulating *S.* Infantis strains. Coverage of selected CTL and HTL epitopes across HLA alleles is presented in **Supplementary File 8**. In addition, selected CTL epitopes were also found among the predicted CTL epitopes from all publicly available chicken MHC class I BF1 and BF2 sequences using NetMHCpan v4.1. (**Supplementary File 9**). Although precise affinity estimates remain constrained for avian MHC molecules, the recurrent identification of these epitopes across multiple BF variants indicates a high likelihood of effective presentation in chickens. This, coupled with the relatively limited polymorphism of the chicken MHC’s especially within commercial poultry populations. These results collectively support broad host coverage of the selected epitopes and the overall vaccine construct.

#### 3.2.2 Predicted b-cell epitopes and selection

B-cell epitopes are responsible for initiating the humoral immune response by stimulating B lymphocytes to produce antigen-specific antibodies, an essential mechanism for neutralizing extracellular pathogens. Using the ABCpred server, 41 linear B-cell epitopes were initially predicted, of which 20 were identified as antigenic (**Supplementary Files 6**, **10**). The five most antigenic epitopes were selected for incorporation into the final multi-epitope vaccine construct.

#### 3.2.3 Epitope validation and vaccine assembly

**Figure 2:**
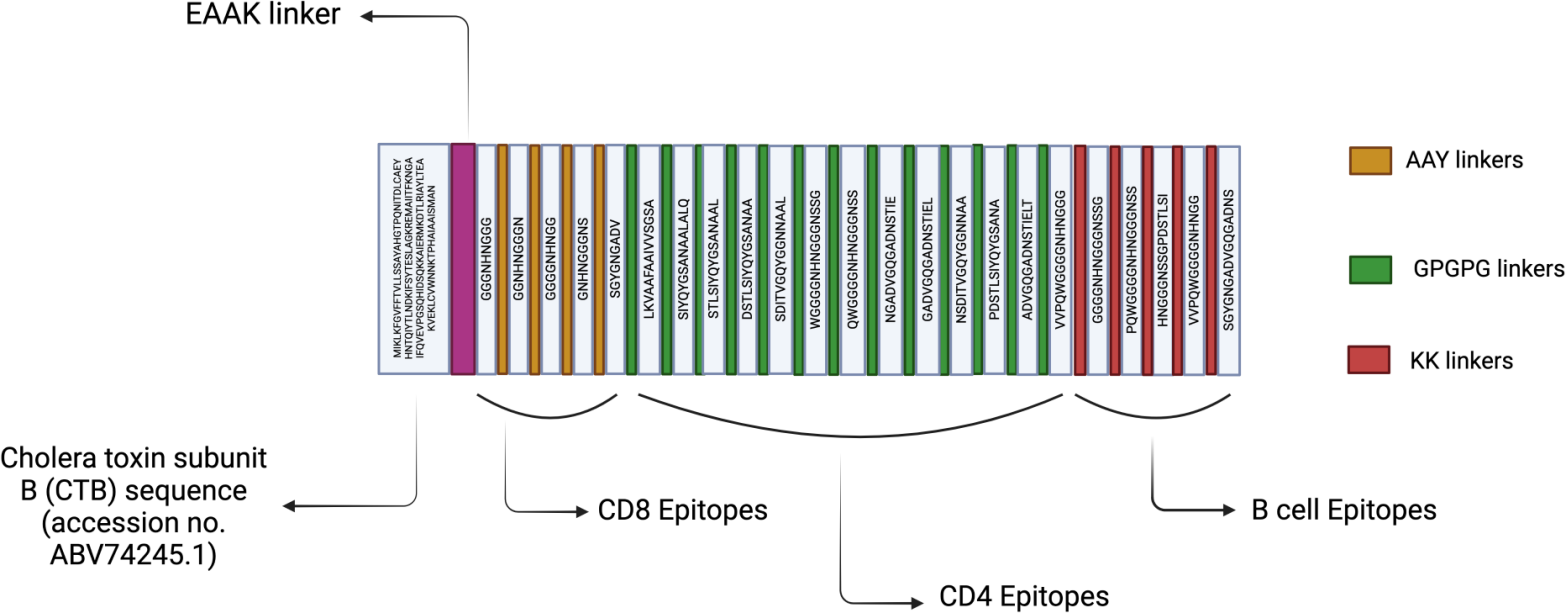
A 532-amino acid (AA) vaccine construct comprising 5 cytotoxic T lymphocyte (CTL), 13 helper T lymphocyte (HTL), and 5 B-cell epitopes connected using AAY, GPGPG, and KK linkers, respectively. Additionally, the construct includes an N-terminal cholera toxin B (CTB) adjuvant linked via an EAAK linker.

Final epitope sets were validated for conservancy, allergenicity, and toxicity. The selected 5 CTL, 13 HTL, and 5 B-cell epitopes (**Table 2**) were joined using AAY, GPGPG, and KK linkers, respectively. A cholera toxin B subunit (CTB) was fused to the N-terminus using an EAAK linker, resulting in a 532-amino acid multi-epitope vaccine (MEV) construct (**Figure 2**).

**Table 2:** Selected top-ranked CTL, HTL, and B-cell epitopes for a multi-epitope vaccine construct (MEV).

NO	CD8^+^ CTL epitopes	CD4^+^ HTL epitopes	B-cell epitopes
1	GGGNHNGGG	LKVAAFAAIVVSGSA	GGGGNHNGGGNSSG
2	GGNHNGGGN	SIYQYGSANAALALQ	PQWGGGGNHNGGGNSS
3	GGGGNHNGG	STLSIYQYGSANAAL	HNGGGNSSGPDSTLSI
4	GNHNGGGNS	DSTLSIYQYGSANAA	VVPQWGGGGNHNGG
5	SGYGNGADV	SDITVGQYGGNNAAL	SGYGNGADVGQGADNS
6		WGGGGNHNGGGNSSG	
7		QWGGGGNHNGGGNSS	
8		NGADVGQGADNSTIE	
9		GADVGQGADNSTIEL	
10		NSDITVGQYGGNNAA	
11		PDSTLSIYQYGSANA	
12		ADVGQGADNSTIELT	
13		VVPQWGGGGNHNGGG	

### 3.3 Antigenicity, stability, solubility, and safety of the MEV

VaxiJen and AntigenPro predicted the MEV construct to have strong antigenicity (1.5127 and 0.7999, respectively). BLASTp confirmed no homology between the MEV and *Gallus gallus* (domestic chicken) proteins (**Supplementary File 11**). Both AllerTOP and AlgPred classified the construct as non-allergenic. ToxinPred 3.0 predicted the MEV construct to be a non-toxin. The construct had favorable physicochemical properties, including a molecular weight of 51.18 kDa, a theoretical isoelectric point (pI) of 6.56, and a half-life of 30 h in mammalian systems, >20 h in yeast, and ~10 h in *E. coli*. Its instability index (II) was 21.53, indicating stability. The aliphatic index (52.58) and the grand average of hydropathicity (GRAVY) (-0.560) suggested thermal stability and hydrophilicity. A solubility score of 0.517 predicted high solubility upon expression.

### 3.4 Secondary and tertiary structural analysis and refinement

**Figure 3:**
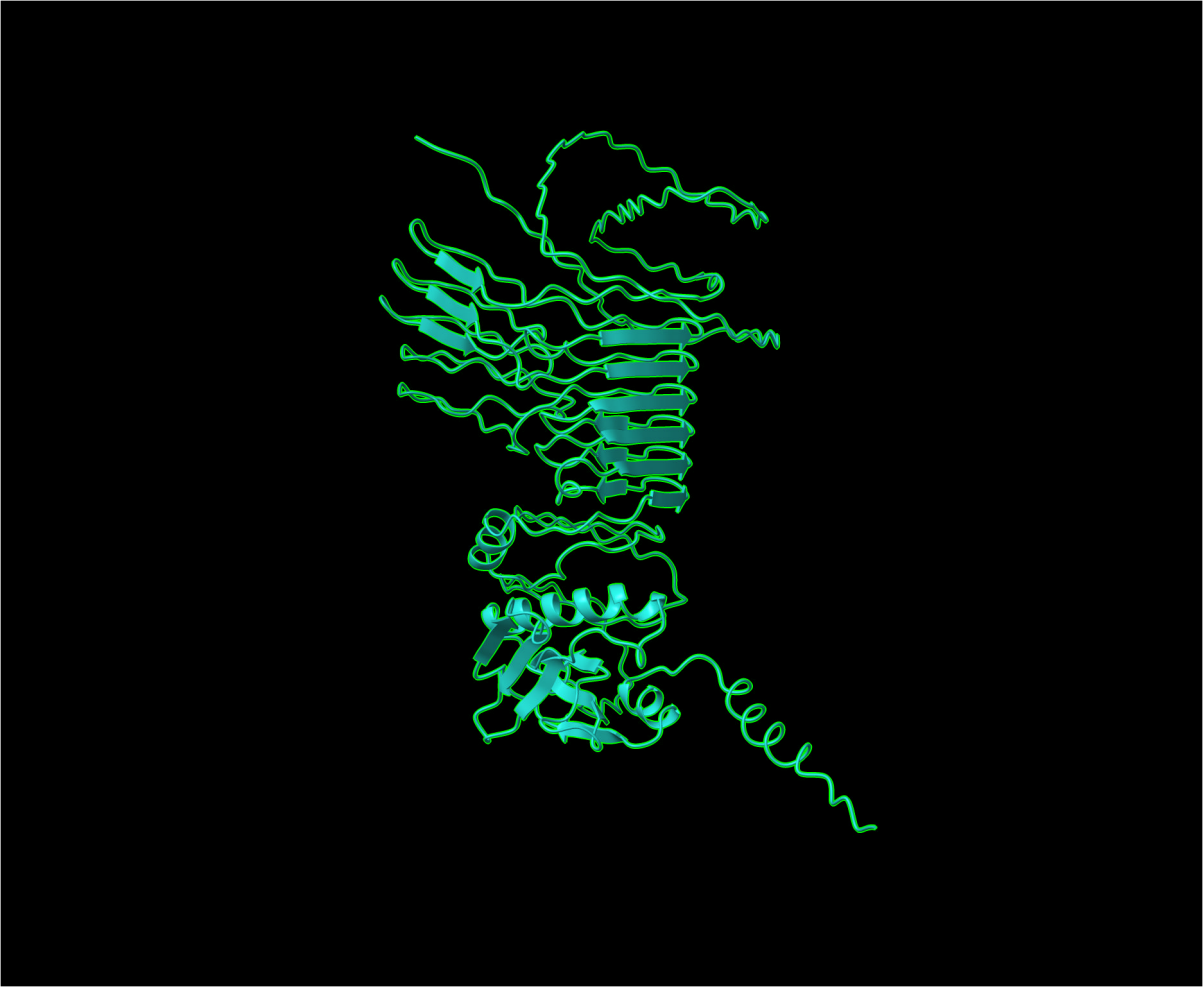
Three-dimensional tertiary structure of the MEV construct predicted by Alphafold 3, visualized by Chimera X.

**Figure 4:**
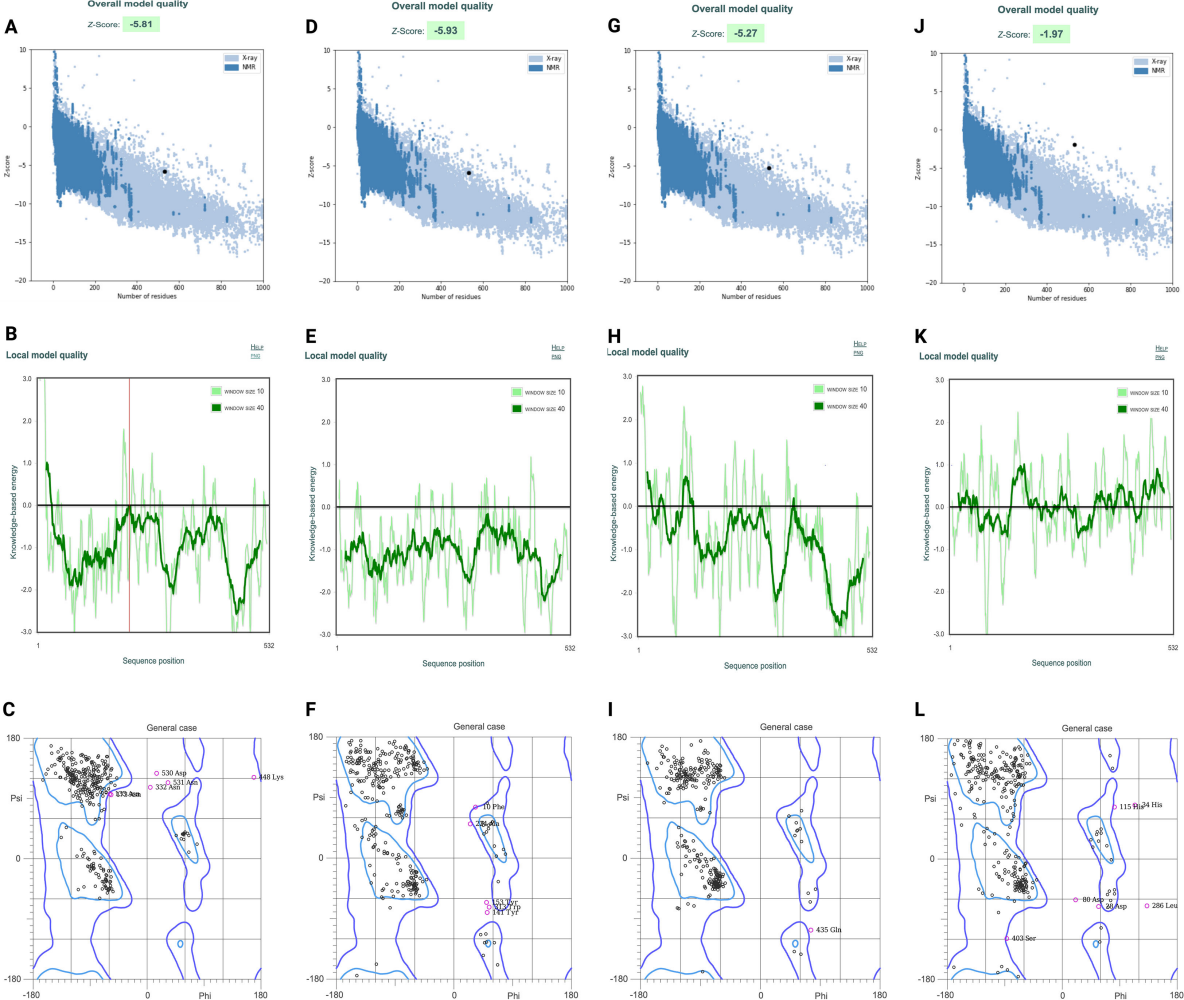
Structural quality assessments of models generated by four independent structure prediction servers. Panels are organized by prediction method from left to right: AlphaFold3 **(A–C)**, Robetta **(D–F**), TrRosetta **(G–I)**, and I-TASSER **(J–L)**. Figures **(A, D, G, J)** represent overall model quality plots showing Z-scores (highlighted) relative to experimentally determined protein structures of comparable size derived from X-ray crystallography and NMR datasets. Figures **(B, E, H, K)** represent local model quality plots depicting residue-wise knowledge-based energy profiles using sliding window sizes of 10 and 40 residues. Regions with negative energy values indicate favorable local structural environments, whereas positive deviations highlight potential local instabilities. Representative model from I-TASSER shows much higher positive values compared to the other three models. Figures **(C, F, I, L)** Ramachandran plots (general case) from MolProbity server representing backbone dihedral angle (φ/ψ) distributions. The majority of residues for AlphaFold3, Robetta, and TrRosetta models fall within favored and allowed regions, yielding acceptable Rama-Z scores, while I-TASSER models show increased outliers consistent with reduced stereochemical quality.

To evaluate the construct’s stability and functionality, a secondary structure analysis was conducted. PSIPRED predicted that the secondary structure comprises 13.34% α-helix, 38.15% β-strand, and 48.51% coil. Tertiary structure models were generated using AlphaFold3, Robetta, TrRosetta, and I-TASSER. Models from I-TASSER and TrRosetta were subjected to refinement using the GalaxyRefine server to improve local geometry and side-chain conformations. Model quality was subsequently evaluated using ProSA-web and MolProbity, servers focusing on global energy profiles (Z-scores) and stereochemical quality (Rama-Z scores), respectively. ProSA-web analysis demonstrated that refined models generated by AlphaFold3 (**Figure 3**), Robetta, and TrRosetta exhibited Z-scores ranging from -5.93 to -5.26, which fall within the range typically observed for experimentally determined protein structures of comparable size, indicating favorable global structural quality (**Table 3**; **Figure 4**). In contrast, refined I-TASSER models showed substantially less favorable Z-scores (approximately -2.18 to −1.91), suggesting lower overall model reliability. Stereochemical validation from MolProbity server using Ramachandran analysis further supported these findings. AlphaFold3, Robetta, and TrRosetta models displayed Rama-Z scores between -1.6 and -2.95, well within the acceptable range (-3 to +3), indicating normal backbone conformational distributions. Conversely, I-TASSER models consistently yielded Rama-Z scores below -3.5, reflecting a higher proportion of unfavorable backbone conformations and reduced stereochemical quality. Based on the combined assessment of global energy profiles (ProSA Z-scores) and backbone geometry (MolProbity Rama-Z scores), refined models from AlphaFold3, Robetta, and TrRosetta were deemed structurally reliable and selected for subsequent protein–protein docking analyses, while I-TASSER models were excluded due to inferior structural quality despite refinement.

**Table 3:** Evaluation metrics of models from TrRosetta, I-TASSER, Alphafold 3, and Robetta, based on tertiary structure refinement and evaluation, for subsequent docking. .

Prediction method	Model	Z-scores	Rama distribution Z-score
TrRosetta (Galaxy Refined)	1	-5.27	-1.81 ± 0.31
	2	-5.34	-1.76 ± 0.32
	3	-5.26	-1.7 ± 0.32
	4	-5.34	-1.73 ± 0.32
	5	-5.33	-1.6 ± 0.32
I-TASSER (Galaxy Refined)	1	-1.97	-3.50 ± 0.31
	2	-1.94	-3.58 ± 0.31
	3	-1.98	-3.63 ± 0.31
	4	-2.18	-3.75 ± 0.30
	5	-1.91	-3.67 ± 0.31
Alphafold 3	1	-5.81	-2.91 ± 0.28
	2	-5.59	-3 ± 0.29
	3	-5.54	-2.87 ± 0.3
	4	-5.48	-2.85 ± 0.29
	5	-5.55	-2.95 ± 0.28
Robetta	1	-5.93	-2.21 ± 0.31
	2	-4.87	-2.56 ± 0.31
	3	-5.29	-2.16 ± 0.33
	4	-4.7	-1.66 ± 0.34
	5	-5.76	-2.05 ± 0.31

### 3.5 Molecular docking of MEV with host receptors

**Figure 5:**
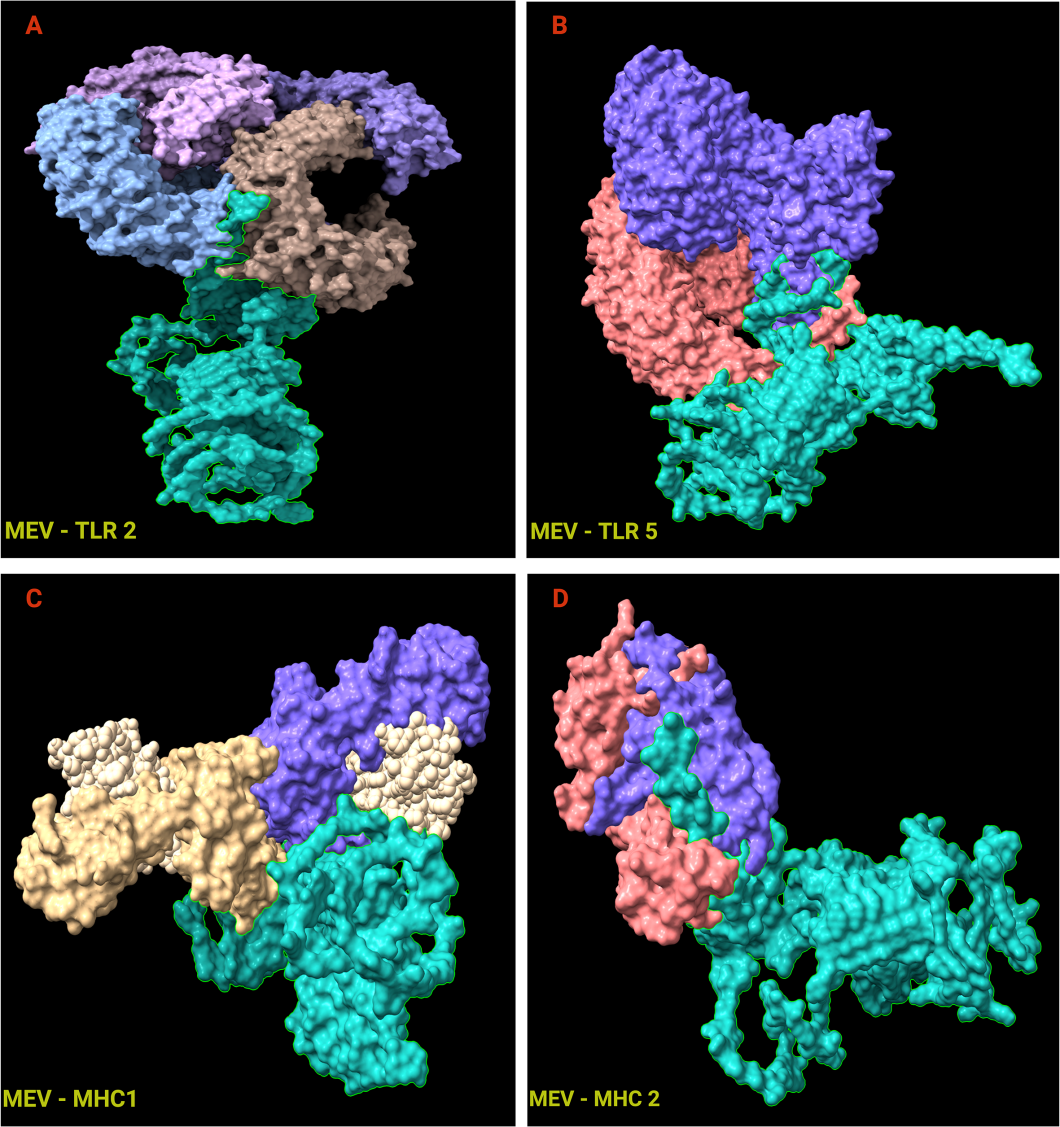
Three-dimensional docked complexes of predicted vaccine construct by Alphafold 3 with ligands TLR2 **(A)**, TLR5 **(B)**, MHC1 **(C)**, MHC2 **(D)**, generated by ClusPro. Structure in green represents the vaccine construct, and other colors represent chains of the receptors. These docked complexes were visualized by Chimera X.

Docking simulations were performed between vaccine constructs (MEV) from AlphaFold3, Robetta, and TrRosetta and TLR2, TLR5, MHC-I, and MHC-II receptors using ClusPro server. TrRosetta derived models consistently failed to dock with TLR2, MHC-I, and MHC-II and were therefore excluded from evaluation. Docked complexes derived from Robetta and Alphafold 3 models, binding affinity estimation using PRODIGY revealed favorable binding free energies and low dissociation constants for MEV interactions with all four immune receptors (**Table 4**) indicating stable complex formation under avian physiological temperature conditions (41 °C). Among these, docked complexes derived from Alphafold 3 structures consistently exhibited more favorable binding free energies and low dissociation constants compared to Robetta derived models. Consequently, AlphaFold3-based docked complexes (**Figure 5**) were selected for subsequent molecular dynamic simulations and normal mode analysis (NMA) using iMODS.

**Table 4:** Binding affinity (ΔG) and dissociation constant (Kd) estimates for MEV–immune receptor complexes generated from AlphaFold3 and Robetta models at 41°C by Prodigy Server.

Prodigy server results			
Tool for tertiary structure prediction	Immune receptor	Binding energy	Kd (M) at 41°C
		ΔG (kcal mol-1)	
AlphaFold 3	TLR2	-23.4	4.80E-17
	TLR5	-26.5	3.60E-19
	MHC1	-15.1	3.00E-11
	MHC2	-16.3	4.50E-12
Robetta	TLR2	-18.9	7.50E-14
	TLR5	-17.2	1.00E-12
	MHC1	-14.2	1.20E-10
	MHC2	-12.8	1.20E-09

### 3.6 Molecular dynamic simulation and molecular interaction analysis of vaccine-receptor complexes

**Figure 6:**
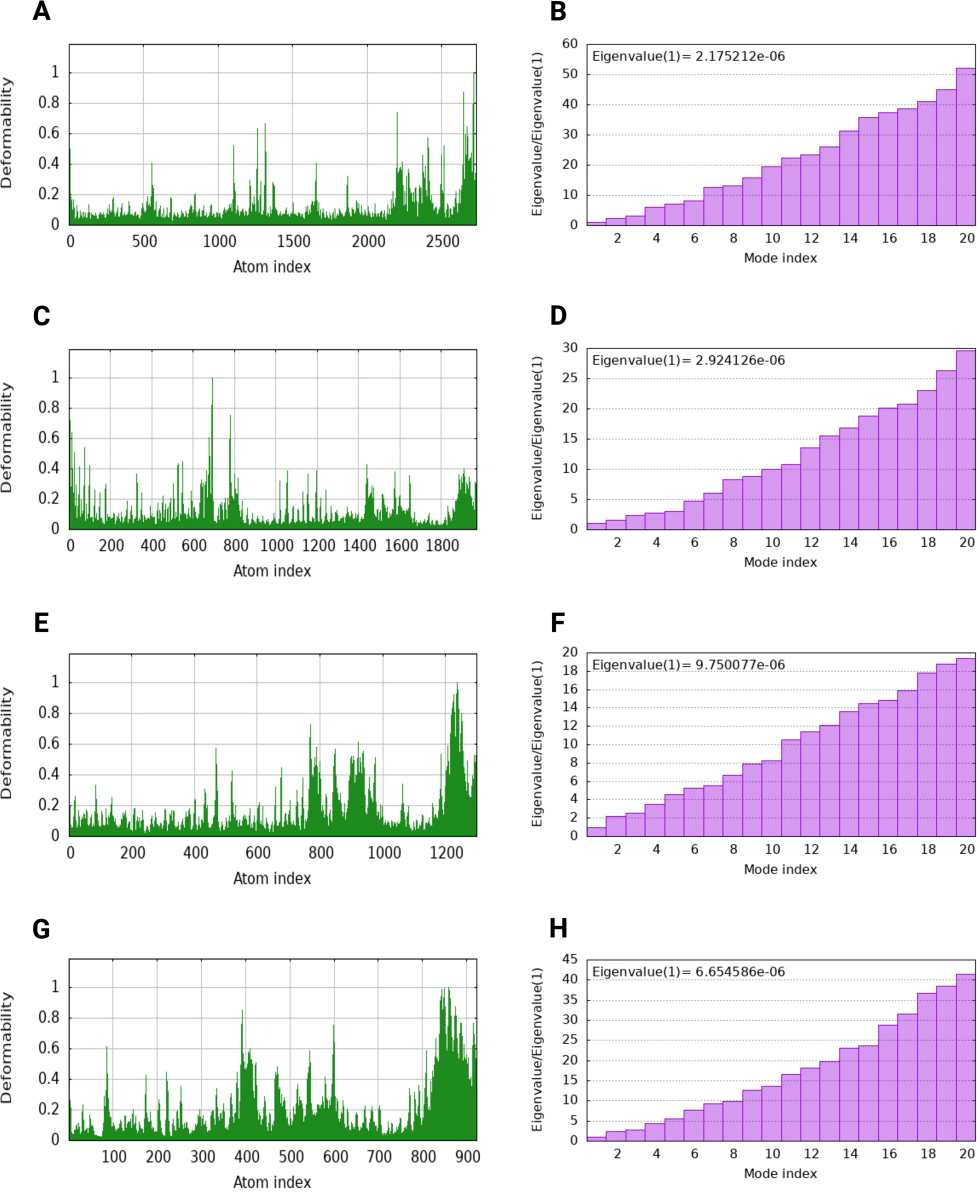
Normal mode analysis (NMA) of docked MEV–receptor complexes. Deformability profiles **(A, C, E, G)** illustrate residue-wise flexibility of the MEV complex with TLR2, TLR5, MHC-1, and MHC-2 complexes, respectively, highlighting localized hinge regions while maintaining overall structural rigidity. Corresponding eigenvalue distributions **(B, D, F, H)** reflect the energetic cost of collective motions. The low eigenvalues observed for all four complexes indicate favorable intrinsic stability and resistance to large-scale conformational deformation, supporting the structural robustness of MEV interactions with both innate (TLRs) and adaptive (MHCs) immune receptors.

Normal mode analysis (NMA) conducted through iMODS evaluated deformability, flexibility, and atomic interactions of docked complexes. Deformability profiles (**Figure 6**) demonstrated localized flexibility primarily at surface-exposed loop regions and hinge points, while the majority of the complexes exhibited limited deformability, indicating overall structural rigidity. The calculated eigenvalues (**Figure 6**) for the lowest-frequency modes of docked complexes ranged from 2.92 × 10^-6^ to 9.75 × 10^-6^, suggesting that only minimal energy is required for collective conformational motions. Consistent with these findings, structural superposition of the docked complexes using ChimeraX Matchmaker tool showed low RMSD values (< 2 Å), indicating minimal structural deviation and preservation of binding geometry during simulated motions (**Supplementary File 12**). Together, the low eigenvalues and RMSD values support the conformational stability and mechanical favorability of MEV complexes with TLR2, TLR5, MHC-I, and MHC-II receptors, consistent with NMA-based stability assessments reported for multi-epitope vaccine–receptor systems (33).

**Figure 7:**
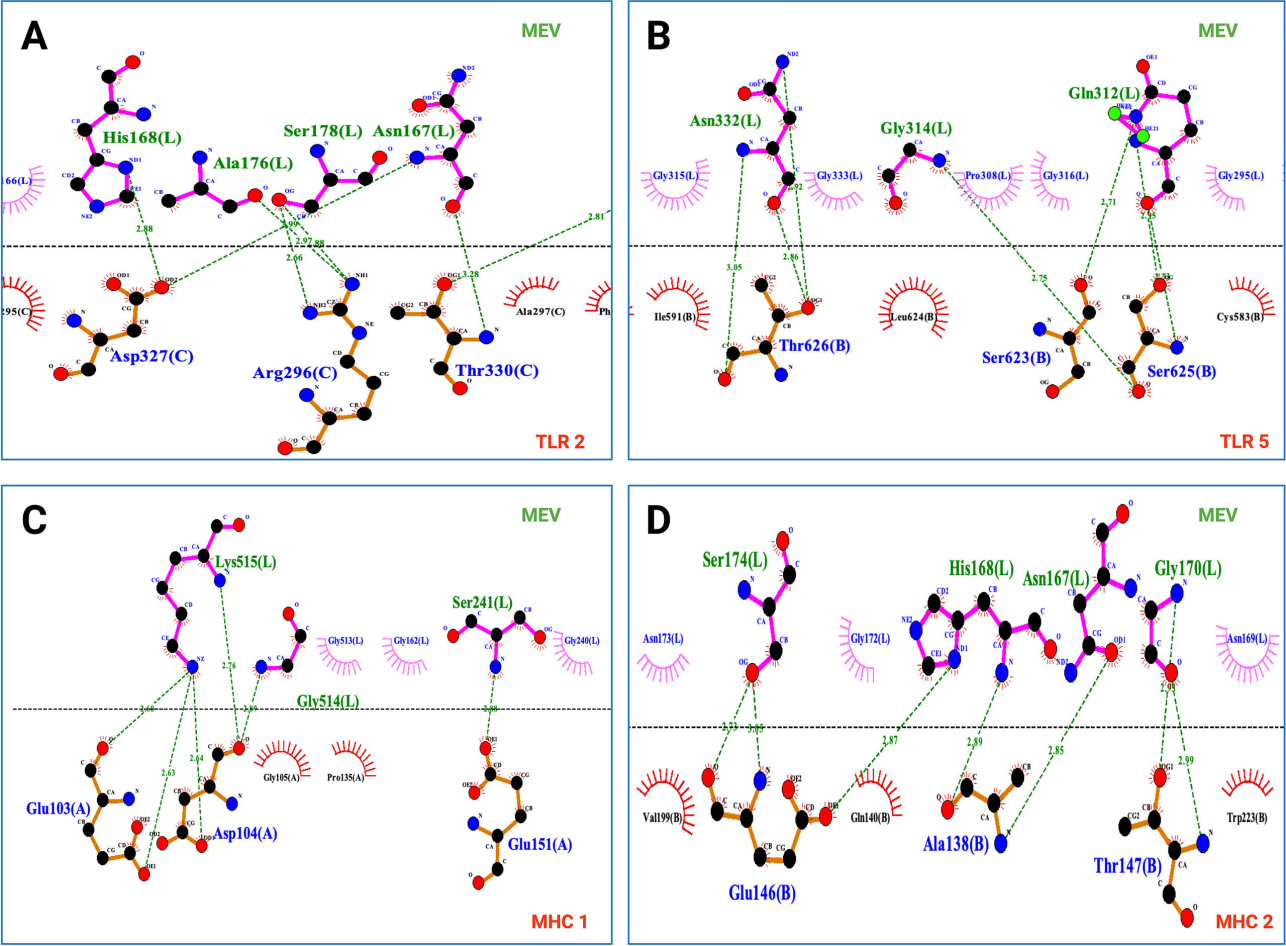
Representative LigPlot+ interaction maps of MEV binding to immune receptors. LigPlot+ diagrams show intermolecular interactions between the multiepitope vaccine (MEV; chain L, green) and immune receptors (blue), including **(A)** TLR2, **(B)** TLR5, **(C)** MHC 1, and **(D)** MHC 2. Hydrogen bonds are indicated by green dashed lines with bond lengths **(Å)**, while hydrophobic contacts are shown as red spoked semicircles. The dashed horizontal line separates ligand (MEV) and receptor chains for clarity. Interactions shown are representative and do not capture the full interface. Across all complexes, MEV engages receptors through multiple distributed hydrogen bonds and extensive hydrophobic packing, supporting stable innate and adaptive immune receptor recognition.

Molecular interaction analysis using LigPlot+ demonstrated that the multi-epitope vaccine (MEV) construct establishes extensive and well-distributed intermolecular contacts with innate immune receptors TLR2 and TLR5, as well as adaptive immune receptors MHC-I and MHC-II, through a combination of directional hydrogen bonding and complementary hydrophobic packing (**Figure 7**; **Supplementary File 13**). In the MEV–TLR2 complex, the interface is stabilized by multiple hydrogen bonds involving MEV residues spanning Ala140–Ala176–Ser178–His168–Asn167–Gly170–Asn169, engaging complementary polar and charged residues on TLR2 (e.g., Asp263, Asn294, Arg296, Ser298, Ala297, Tyr332, and Asp305), together with dense hydrophobic contacts contributed by surrounding nonpolar residues. Notably, engagement of the Ala176–Ser178 segment, with Ser178 forming multiple hydrogen bonds and Ala176 contributing hydrophobic interactions, supports an extended binding surface rather than a single interaction hotspot. Similarly, MEV–TLR5 binding is characterized by a broad interaction network in which MEV residues such as His134, His145, His168, Asn144, Asn146, Asn158, Tyr141, Tyr153, Gly149, Gly157, Ala151, Gln312, Trp313, Asn332, Asp335, and Lys516 form recurrent hydrogen bonds (predominantly ~2.6–3.2 Å) with complementary TLR5 residues, accompanied by continuous hydrophobic packing involving Leu-, Val-, Ile-, Phe-, and Met-rich receptor patches. For adaptive immune receptors, MEV–MHC-I interaction analysis revealed extensive hydrogen bonding between diverse MEV residues (including Tyr258, Tyr260, Tyr239, Tyr216, Ser212, Lys515, Gly514, Ser241, Asn510/512, Asn182, and Gly157) and key MHC-I residues (e.g., Gly18, Gln19, Arg42, Arg74, Arg82, Asp14, Glu103, Asp104, Glu151, and Arg166), together with substantial hydrophobic contacts involving Pro-, Val-, Gly-, and aromatic-rich regions of the MHC-1 binding surface. This distributed interaction network supports stable anchoring of the MEV across multiple interface regions, consistent with efficient MHC-I engagement and peptide presentation. Likewise, MEV–MHC-2 complexes exhibited recurrent hydrogen bonding mediated by MEV residues Gly142–Gly143–Ala140–Ala164–Gly162–Asn158–His159–His168–Asn167–Gly170–Ser174–Ser178–Tyr177–Lys516, interacting with complementary MHC-II residues such as Arg128, Arg149, Arg162, Arg181, Glu146, Glu175, Glu186, Ala138, Thr147, Thr174, Trp183, and Met182, alongside extensive hydrophobic packing within the peptide-binding groove. Importantly, interactions with MHC-2 are distributed along multiple MEV segments rather than localized to a single contact site, indicating broad surface complementarity and stable accommodation within the MHC-2 cleft. Collectively, across all docked complexes examined, the coexistence of multiple directional hydrogen bonds with extensive hydrophobic stabilization supports structurally stable and biologically plausible MEV engagement of both innate and adaptive immune receptors.

### 3.7 Immune simulation predicts robust vaccine-induced immune response

**Figure 8:**
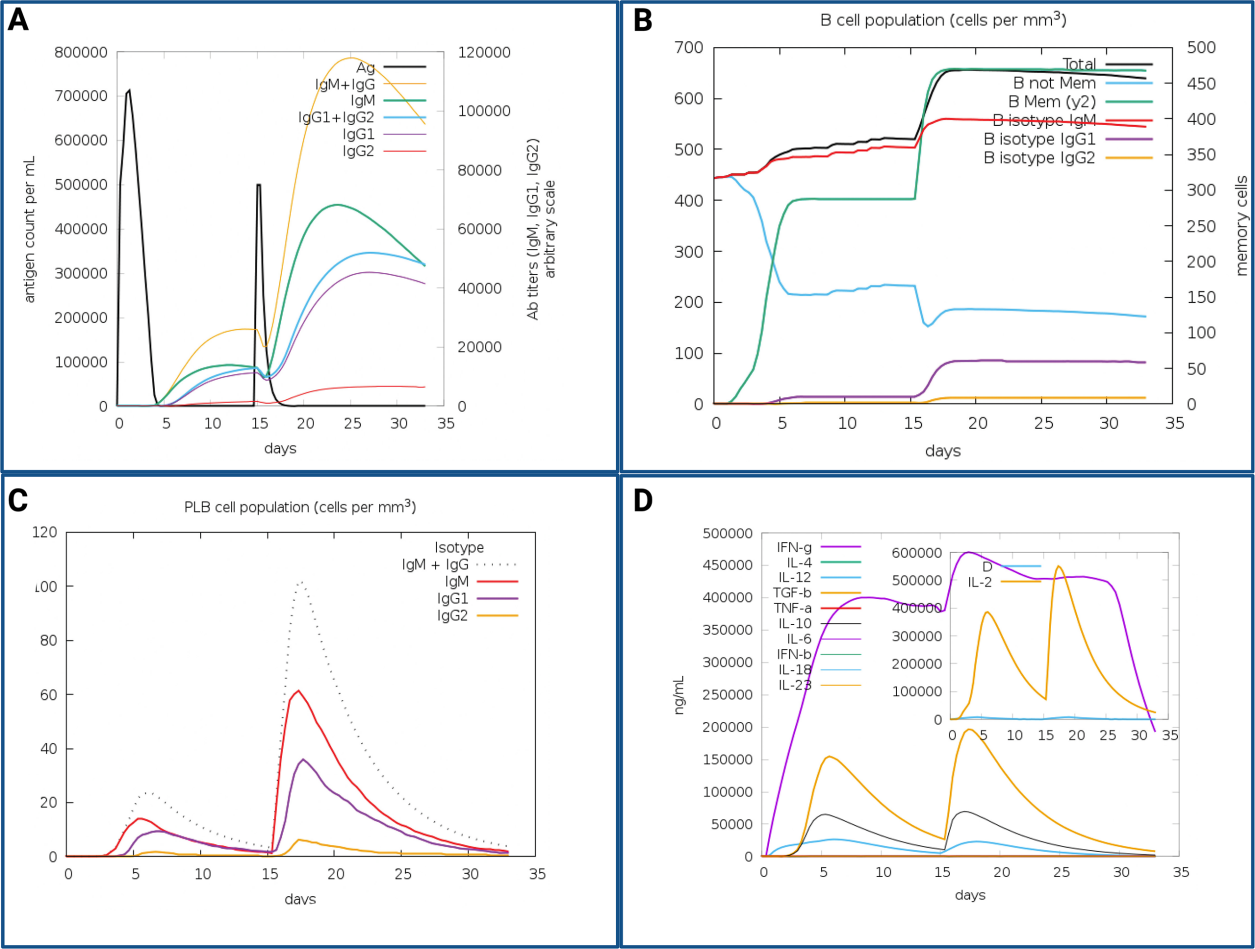
Immune simulation results of the multiepitope vaccine (MEV) using the C-ImmSim server. **(A)** Increased levels of IgM+IgG and IgG1+IgG2, accompanied by a reduction in antigen levels following vaccine administration. **(B)** Progressive increase in total B-cell and memory B-cell populations after both immunizations. **(C)** Elevated levels of plasma B-cell isotypes indicate enhanced humoral response. **(D)** Increased concentrations of cytokines and interleukins, reflecting robust cell-mediated immunity post-vaccination. The insert graph shows a negligible danger signal [D] along with a strong IL-2 response, indicating effective T cell activation without systemic stress. Simulations were conducted with two doses administered on days 1 and 14.

Simulations via C-ImmSim predicted robust humoral and cellular immune responses. Antibody titers (IgM, IgG1, IgG2) increased post-vaccination, coupled with a decrease in antigen titers. Plasma and memory B-cell populations increased over time along with elevated cytokine and interleukin levels, indicating a robust and long-term immunological memory (**Figures 8A–D**).

### 3.8 Codon optimization and *in silico* cloning

**Figure 9:**
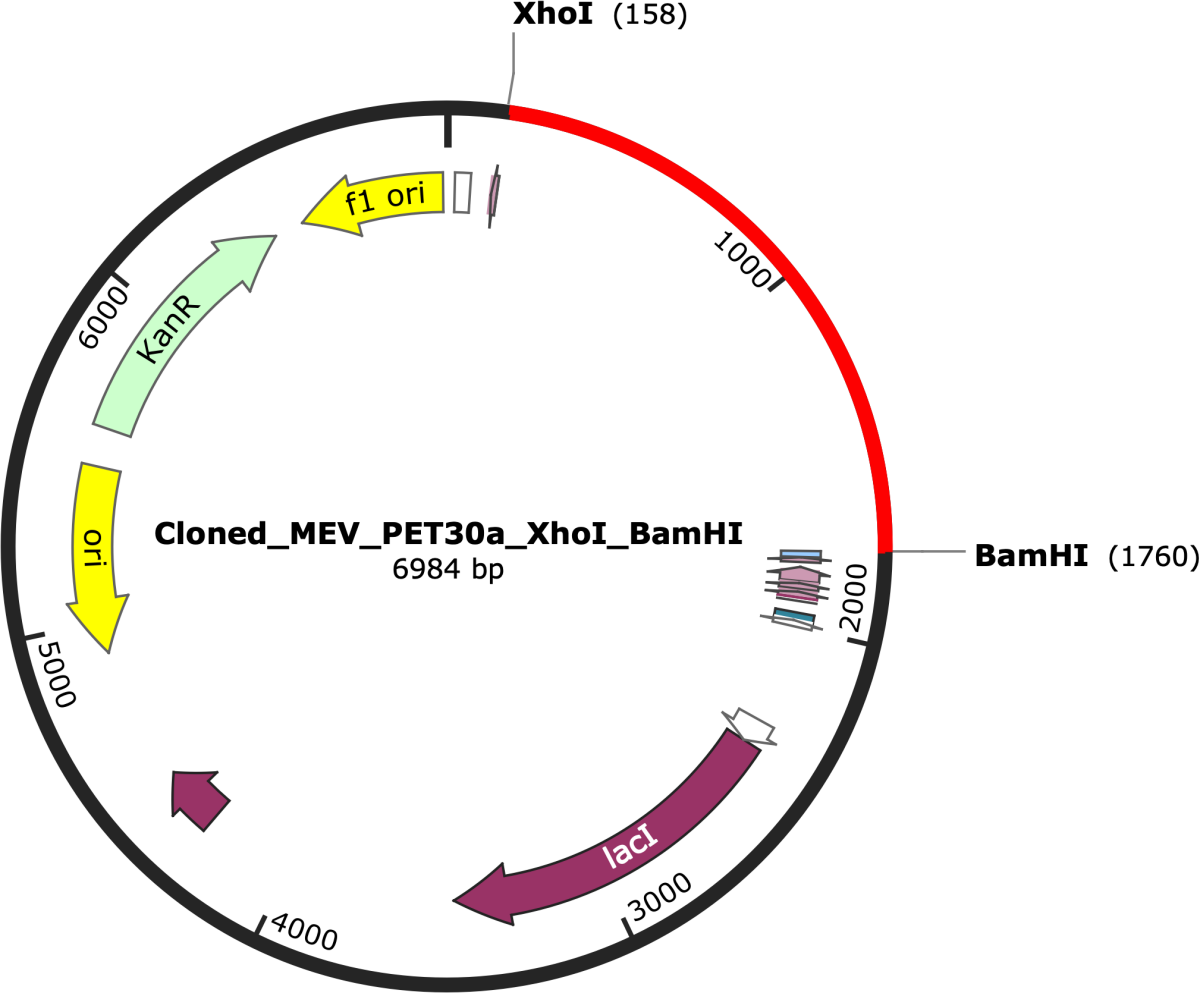
In silico cloning of the codon-optimized MEV sequence into the pET30a(+) plasmid vector using SnapGene Viewer v5.3. The MEV cloned sequence is represented in red in the vector backbone. The construct was inserted between the XhoI (position 158) and BamHI (position 1760) restriction sites for potential expression in E. coli. .

To facilitate experimental expression in *E. coli*, the MEV was codon-optimized using JCat, achieving a CAI of 1.0 and GC content of 54.76%. These values indicate the suitability of the MEV for *E. coli* BL21 expression. The optimized sequence was cloned into the pET30a(+) vector between XhoI and BamHI sites using SnapGene v5.3 (**Figure 9**), enabling future wet-lab validation.

## 4 Discussion

Non-typhoidal *Salmonella* (NTS), particularly serovar Infantis, has emerged as a significant public and animal health threat due to its global dissemination and increasing multidrug resistance (MDR) rates. Between 2011 and 2016, *S*. Infantis infections surged by 167% in the United States, with a notable outbreak in 2018 linked to contaminated poultry products (10). This pattern is mirrored globally, including in Europe, where *S.* Infantis ranks among the most frequently isolated serovars in poultry and poultry meat (10–12). The emergence of clonal MDR strains harboring pESI-like megaplasmids poses a challenge for control and treatment, emphasizing the need for targeted prevention strategies (13). Despite vaccination being central to *Salmonella* control in poultry (15, 16), commercial vaccines predominantly focus on *S.* Typhimurium and *S.* Enteritidis, leaving a critical gap in protection against *S.* Infantis. Our findings address this gap by applying immunoinformatics-driven vaccine design against a high-priority, poultry-associated MDR serovar.

CsgA, a major subunit of curli fibers involved in adhesion and biofilm formation, emerged from a subtractive proteomics pipeline as the most conserved and surface-associated protein across 692 poultry-derived *S.* Infantis genomes (**Supplementary File 1**). Its essentiality, surface exposure, antigenicity, lack of host homology, and its functional significance in biofilm formation qualified it as a strong vaccine target over other targets. While numerous other *Salmonella* vaccine studies have targeted surface-associated structures such as flagellar proteins, outer membrane porins, secretion system components, and polysaccharides, these antigens exhibit inherent biological limitations that may restrict the breadth and durability of protection, particularly in poultry. Flagellar antigens (e.g., FliC/FljB), though capable of eliciting systemic and mucosal antibody responses under experimental conditions, showed poor reduction in shedding, systemic, and cecal *Salmonella* loads post challenge when used as a vaccine candidate (69). In addition, flagellar antigens FliC/FljB undergo phase variation and are dynamically regulated *in vivo* to evade inflammasome-mediated immune detection, reducing their reliability as stable vaccine targets (70, 71). Outer membrane porins, including OmpA, OmpC, OmpD, and OmpF, have also been evaluated either as individual antigens or as components of outer membrane vesicle-based vaccines and have shown varying degrees of protection against homologous *Salmonella Enteritidis* challenge (69, 72, 73). However, porin sequences are not uniformly conserved; even highly conserved proteins such as OmpC display sufficient sequence variability to segregate into distinct clades within the same serovar (74). Moreover, the expression of porins *in vivo* is strongly influenced by environmental conditions (75, 76), which may contribute to variability in vaccine efficacy. Type III secretion system (T3SS) effectors and structural proteins, which constitute major *Salmonella* virulence factors, have also been studied as potential vaccine targets. SseB (a T3SS secretory protein) has been evaluated as a subunit vaccine target in chickens against *Salmonella* Enteritidis, but conferred only limited cross-serovar protection (69), even though SseB is considered highly conserved across serovars. T3SS proteins are expressed transiently either during epithelial invasion or after bacterial internalization, thereby limiting their accessibility to circulating antibodies once bacteria reside intracellularly (77). Furthermore, the relevance of T3SS-based targets may be serovar dependent, as *S. Enteritidis* exhibits greater invasiveness and T3SS utilization than *S. Infantis*, which is largely restricted to the intestine. Peptidoglycan-associated lipoproteins (Pal), a component of the Tol–Pal envelope maintenance system (78), have also been explored as a vaccine target. A ΔPal mutant of *S. Enteritidis* demonstrated reduced adhesion, invasion, and intracellular survival in murine models (79). However, Pal is primarily periplasmic and poorly accessible to antibodies in intact bacteria, limiting its effectiveness as a standalone subunit antigen. In contrast, CsgA is a stably expressed extracellular matrix protein during the sessile and biofilm-associated persistence phase and does not undergo phase variation, rendering it a predictable and accessible immune target at the mucosal surface. Curli fibers extend beyond the lipopolysaccharide layer, facilitating antibody access during early stages of colonization. Furthermore, CsgA is known to engage host pattern recognition receptors like CD14 and TLR2, modulating immune responses (65). Previous studies have identified CsgA as a viable antigen in vaccine designs for *S.* Typhimurium, *S.* Pullorum, and *Vibrio parahaemolyticus*, supporting its broad immunogenic potential (66, 80, 81). The decision to prioritize CsgA in this study aligns with pan-*Salmonella* vaccine studies, where it has been recognized as a top-ranking immunogen (82). These data underscore its suitability as a universal antigen and its potential for cross-serovar protection.

The multi-epitope vaccine (MEV) presented in this study was rationally assembled using a curated set of CTL, HTL, and B-cell epitopes derived from the highly conserved curli subunit CsgA, an antigen functionally linked to *Salmonella* adhesion, biofilm formation, and innate immune activation. In total, 5 CTL, 13 HTL, and 5 B-cell epitopes satisfied stringent criteria for antigenicity, immunogenicity, conservancy, and non-allergenicity, supporting balanced activation of cellular and humoral immune pathways (**Table 2**; **Supplementary Files 5**, **7**, **10**). The construct features an N-terminal Cholera Toxin Subunit B (CTB) adjuvant to enhance mucosal immunogenicity and promote antigen uptake and presentation. CTB is well-documented for augmenting both humoral and mucosal immune responses, making it an effective adjuvant against enteric pathogens (45, 83). Additionally, linkers were incorporated to maintain optimal epitope exposure. This design aligns with established in silico vaccinology methodologies (26, 28, 29, 84, 85). The resulting 532-amino acid construct (**Figure 2**) is predicted to be highly antigenic (VaxiJen score: 1.5127), stable (instability index: 21.53), non-toxic, and soluble (**Supplementary File 11**).

Structural modeling using multiple independent platforms (AlphaFold3, Robetta, TrRosetta, and I-TASSER) enabled comparative evaluation of model quality and minimized bias associated with any single prediction algorithm. Consistent with recent observations for synthetic and chimeric vaccine constructs, models generated by AlphaFold3 and Robetta exhibited superior global energy profiles and stereochemical properties compared to template-dependent approaches, like I-TASSER upon evaluation with tools such as ProSA and MolProbity. Tools such as ProSA and MolProbity are primarily benchmarked against experimentally resolved proteins and may underestimate the quality of *de novo* or multi-domain constructs lacking close structural homologs. Therefore, these validation metrics should be interpreted as relative indicators rather than definitive measures of structural correctness, a limitation increasingly acknowledged while using various validation tools (86).

Cluspro Docking and normal mode analyses from iMODS further suggested that the MEV construct can engage both innate and adaptive immune receptors through structurally stable interfaces. Predicted interactions with TLR2 and TLR5 are particularly relevant given the established role of CsgA in TLR2-mediated immune sensing, as well as the contribution of TLR5 signaling to mucosal immune activation in the avian gut. Likewise, docking with MHC 1 and MHC 2 molecules supports the potential of the construct to enter antigen-processing pathways required for CD8^+^ and CD4^+^ T-cell activation. Consistent with these docking and NMA outputs, LigPlot+ analysis of interaction MEV–receptor docked complexes indicated a dense network of hydrogen bonds and hydrophobic contacts across the MEV–receptor interfaces, supporting broad surface complementarity and plausible stabilization rather than a single localized binding hotspot. However, it is important to emphasize that protein–protein docking and binding energy estimates remain approximations that do not fully capture receptor dynamics, chicken immune receptor structures, membrane context, co-receptor involvement, or intracellular antigen processing. Accordingly, these analyses are viewed as evidence of structural compatibility rather than definitive proof of receptor engagement *in vivo*.

In silico simulated immune responses using C-IMMSIM highlighted strong IgM and IgG titers, robust memory B- and T-cell responses, and high levels of IFN-γ and IL-2 following booster doses (**Figure 5**). These results suggest that the MEV candidate can induce durable and protective immune memory, consistent with immunogenicity benchmarks in poultry vaccine studies, and underscore the value of computational immunology in early-stage vaccine development (22, 28–30, 84, 85, 87–91).

Codon optimization for E. coli K12 (CAI: 1, GC content: 54.76%) and in silico cloning into the pET30a(+) vector (**Figure 6**) confirmed high expression potential, bridging the computational pipeline to experimental feasibility, and supporting cost-effective, large-scale production.

This study builds upon and expands prior MEV designs against other *Salmonella* serovars (32, 82) by offering a serovar-specific, epitope-level vaccine candidate against *S*. Infantis. It is distinct from live attenuated vaccines in terms of safety profile and from whole-protein subunit vaccines in terms of precision immunogenicity. Compared to nanoparticle or polymeric carrier platforms (16), our MEV approach is modular, readily updatable, and compatible with a range of delivery systems.

While these results are based on computational models, numerous experimental studies have shown that recombinant and subunit vaccines delivered via subcutaneous (88), intramuscular (89–92), or oral routes either directly (93) or via bacterial vectors (94, 95) can elicit strong immune responses. These approaches have demonstrated efficacy against colibacillosis (88), necrotic enteritis (89–91), *Salmonella enterica* infections (93–95), *Mycoplasma Synoviae* (92), and H9N2 avian influenza in mice (87), demonstrating enhanced antigen-specific humoral and cellular responses, reduced pathogen burden, and improved disease outcomes.

Despite these promising in silico results, several inherent limitations should be explicitly recognized. Epitope prediction algorithms for CTL, HTL, and B-cell responses are trained predominantly on mammalian datasets and experimentally characterized human or murine MHC alleles; extrapolation to avian MHC (BF/BL) and IgY responses is therefore approximate and may not fully capture chicken-specific peptide binding or B-cell recognition patterns. Similarly, the additional NetMHCpan analyses using chicken MHC sequences provide qualitative support for broad presentation but cannot substitute for empirical peptide–MHC binding or tetramer studies due to the limited structural and affinity data available for chicken MHC molecules. Docking and normal mode simulations also simplify key aspects of biophysics, including solvent effects, glycosylation, membrane context for TLRs, and large-scale receptor dynamics, meaning that predicted affinities and interaction interfaces represent hypotheses rather than definitive interaction maps. Finally, immune simulations such as C-IMMSIM are based on human immunological frameworks adapted for non-human species and do not yet fully capture avian-specific immunodynamics, like bursa-dependent B-cell development and species-specific cytokine profiles. However, future development of chicken-specific MHC-binding prediction tools will certainly enhance precision, improve rational vaccine design for avian pathogens, and is urgently needed.

Nevertheless, computational pipelines like the one used here markedly accelerate antigen discovery and hypothesis generation while reducing early-phase costs and biosafety requirements. Building on the strong in silico immunogenicity and expression profiles of the MEV candidate, this study provides a compelling rationale for experimental validation through a stepwise translational pipeline. Initial efforts will focus on recombinant expression and purification of the vaccine construct in *Escherichia coli*, followed by *in vitro* antigen presentation and cytokine stimulation assays using chicken-derived immune cells. Subsequent *in vivo* trials will involve immunization of broiler chickens using adjuvanted formulations, followed by challenge studies with virulent *S.* Infantis field isolates to evaluate protective efficacy, intestinal colonization, and fecal shedding.

In current poultry practice, the most common vaccine administration routes include drinking water, spray, injection, oculo-nasal drops, and *in ovo* injection, with drinking water and spray being the most widely adopted due to their practicality and ease of mass application. Injectable vaccines, while effective, remain labor-intensive and logistically challenging on commercial farms. Therefore, there is considerable motivation to develop vaccine formulations compatible with oral or spray delivery to align with existing immunization schedules and to stimulate mucosal immune responses critical for reducing intestinal colonization by *Salmonella* spp.

Future *in vivo* studies will assess oral delivery of the vaccine construct, including formulation with chitosan-based nanoparticles, which have been shown to effectively deliver *Salmonella* antigens in broilers and enhance both systemic IgY and mucosal IgA responses (93). Additionally, live bacterial delivery platforms may be explored, such as the USDA-licensed *Salmonella* Typhimurium χ8914 strain, previously employed to deliver *Campylobacter* antigens (96), or *Lactobacillus lactis* (95) and *Bacillus subtilis (*94) used to deliver a multi-epitope vaccine against *Salmonella* in poultry.

Building on these practical and translational considerations, this study leveraged a suite of advanced computational and structural biology tools to design a modular, immunologically optimized MEV candidate rationally. Its modularity facilitates adaptation to evolving antigenic profiles, while its predicted expression characteristics support scalable production and formulation for poultry use. Additionally, the potential cross-protective efficacy against MDR *Salmonella* Typhimurium and *Salmonella* Enteritidis strains sharing conserved CsgA epitopes warrants further investigation via comparative epitope mapping.

## 5 Conclusions

This study presents a rationally designed multi-epitope subunit vaccine targeting *Salmonella enterica* serovar Infantis, an increasingly prevalent and multidrug-resistant foodborne pathogen. By integrating subtractive proteomics, immunoinformatics, and reverse vaccinology, we identified the highly conserved and immunogenic surface-associated protein CsgA as a promising vaccine target across 692 poultry-derived genomes. The final MEV construct demonstrated strong predicted antigenicity, non-allergenicity, structural stability, and high-affinity binding to MHC-1 and MHC-2. In silico immune simulations further supported the vaccine’s ability to induce robust, durable humoral and cellular immune responses, including elevated IgG and memory B and T cell levels. These findings contribute to the urgent need for serovar-specific vaccine strategies against emerging non-typhoidal *Salmonella* in poultry. Although experimental validation is required, our computational approach provides a rapid, cost-effective pipeline for early-stage vaccine discovery and supports the translational potential of MEV platforms in food safety and zoonotic disease control.

## Data Availability

The datasets presented in this study can be found in online repositories. The names of the repository/repositories and accession number(s) can be found in the article/**Supplementary Material**.

## Supplementary material

Supplementary File 1 A comprehensive list of all selected Salmonella Infantis genomes, including accession numbers, source information, and geographic metadata obtained for this study.

Supplementary File 2 Antigenicity Scores of all the core proteins as predicted by the Vaxijen 2.0 tool.

Supplementary File 3 shows Vaxijen antigenic Scores, Subcellular localization by TMHMM & psortb 3.0, Functional annotation by SignalP, and essentially scores by Geptop 2.0 of all seven initial candidate protein coding genes selected.

Supplementary File 4 Multi-fasta file of the CsgA protein sequences across all genomes analyzed in this project.

Supplementary File 5 Results from IEDB MHC-I binding tool and IEDB immunogenicity prediction tool used in the selection of final CTL epitopes.

Supplementary File 6 Antigenicity prediction results by Vaxijen 2.0 Tool, for selected CTL, HTL, and B-cell epitopes.

Supplementary File 7 Predicted helper T-cell (HTL) epitope results, including those capable of inducing IL-4 and IFN-γ responses.

Supplementary File 8 File representing global coverage of selected CTL and HTL epitopes across MHC1 and MHC2 alleles, respectively.

Supplementary File 9 Results of predicted CTL epitopes from all publicly available chicken MHC class I BF1 and BF2 sequences using NetMHCpan v4.1.

Supplementary File 10 Results summarizing all predicted B-cell epitopes as identified by the ABCpred server.

Supplementary File 11 Results of Antigenicity, allergenicity, solubility along with Physico-chemical properties and Blastp analysis of MEV (Multi-epitope Vaccine).

Supplementary File 12 Results of RMSD values from chimeraX, between all the simulated motions given by iMODS and cluspro predicted MEV - immune receptor structures.

Supplementary File 13 *Results* from ligplot+ representing complete analysis of MEV-immune receptor interaction of each of 4 immune receptors used in the manuscript.
