# Supplementary material for: In silico design of a multiepitope subunit vaccine targeting Salmonella enterica serovar Infantis: an immunoinformatics and reverse vaccinology approach
Source: Front Immunol. 2026 Feb 6;17:1717278. doi: 10.3389/fimmu.2026.1717278 (PMC12920593; doi:10.3389/fimmu.2026.1717278)
Supplement: Supplementary File 2 — Antigenicity Scores of all the core proteins as predicted by the Vaxijen 2.0 tool. [file DataSheet2.pdf]

The file contains the results from vaxijen tool, which was used for prediction of antigenic nature of all the core proteins selected. A stringent threshold of 0.89 was used.

VaxiJen RESULTS

Model selected: bacteria

Threshold for this model: 0.4

>EHO9884726.1 aspartate transaminase [Salmonella enterica subsp. enterica serovar Infantis] Overall Protective Antigen Prediction = **0.3657** (Probable **NON-ANTIGEN**).

>EHO9884727.1 porin OmpF [Salmonella enterica subsp. enterica serovar Infantis] Overall Protective Antigen Prediction = **0.8294** (Probable **ANTIGEN**).

>EHO9884728.1 asparagine--tRNA ligase [Salmonella enterica subsp. enterica serovar Infantis] Overall Protective Antigen Prediction = **0.4048** (Probable **ANTIGEN**).

>EHO9884729.1 Lrp/AsnC family transcriptional regulator [Salmonella enterica subsp. enterica serovar Infantis] Overall Protective Antigen Prediction = **0.2006** (Probable **NON-ANTIGEN**).

>EHO9884730.1 diaminopropionate ammonia-lyase [Salmonella enterica subsp. enterica serovar Infantis] Overall Protective Antigen Prediction = **0.4754** (Probable **ANTIGEN**).

>EHO9884731.1 sodium:alanine symporter family protein [Salmonella enterica subsp. enterica serovar Infantis] Overall Protective Antigen Prediction = **0.6068** (Probable **ANTIGEN**).

>EHO9884732.1 nicotinate phosphoribosyltransferase [Salmonella enterica subsp. enterica serovar Infantis] Overall Protective Antigen Prediction = **0.3782** (Probable **NON-ANTIGEN**).

>EHO9884733.1 aminopeptidase N [Salmonella enterica subsp. enterica serovar Infantis] Overall Protective Antigen Prediction = **0.4257** (Probable **ANTIGEN**).

>EHO9884734.1 quinone-dependent dihydroorotate dehydrogenase [Salmonella enterica subsp. enterica serovar Infantis] Overall Protective Antigen Prediction = **0.4029** (Probable **ANTIGEN**).

>EHO9884735.1 cell division protein ZapC [Salmonella enterica subsp. enterica serovar Infantis] Overall Protective Antigen Prediction = **0.4872** (Probable **ANTIGEN**).

>EHO9884736.1 YcbX family protein [Salmonella enterica subsp. enterica serovar Infantis] Overall Protective Antigen Prediction = **0.3866** (Probable **NON-ANTIGEN**).

>EHO9884737.1 bifunctional 23S rRNA (guanine(2069)-N(7))-methyltransferase RlmK/23S rRNA (guanine(2445)-N(2))-methyltransferase RlmL [Salmonella enterica subsp. enterica serovar Infantis] Overall Protective Antigen Prediction = **0.4616** (Probable **ANTIGEN**).

>EHO9884738.1 ABC transporter ATP-binding protein [Salmonella enterica subsp. enterica serovar Infantis] Overall Protective Antigen Prediction = **0.3686** (Probable **NON-ANTIGEN**).

>EHO9884739.1 membrane integrity-associated transporter subunit PqiA [Salmonella enterica subsp. enterica serovar Infantis] Overall Protective Antigen Prediction = **0.5143** (Probable **ANTIGEN**).

>EHO9884740.1 intermembrane transport protein PqiB [Salmonella enterica subsp. enterica serovar Infantis] Overall Protective Antigen Prediction = **0.5197** (Probable **ANTIGEN**).

>EHO9884741.1 membrane integrity-associated transporter subunit PqiC [Salmonella enterica subsp. enterica serovar Infantis] Overall Protective Antigen Prediction = **0.5925** (Probable **ANTIGEN**).

>EHO9884742.1 ribosome modulation factor [Salmonella enterica subsp. enterica serovar Infantis] Overall Protective Antigen Prediction = **0.4675** (Probable **ANTIGEN**).

>EHO9884743.1 bifunctional 3-hydroxydecanoyl-ACP dehydratase/trans-2-decenoyl-ACP isomerase [Salmonella enterica subsp. enterica serovar Infantis] Overall Protective Antigen Prediction = **0.4214** (Probable **ANTIGEN**).

>EHO9884744.1 Lon protease family protein [Salmonella enterica subsp. enterica serovar Infantis] Overall Protective Antigen Prediction = **0.4419** (Probable **ANTIGEN**).

>EHO9884745.1 macrodomain Ter protein MatP [Salmonella enterica subsp. enterica serovar Infantis] Overall Protective Antigen Prediction = **0.5407** (Probable **ANTIGEN**).

>EHO9884746.1 porin OmpA [Salmonella enterica subsp. enterica serovar Infantis] Overall Protective Antigen Prediction = **0.6686** (Probable **ANTIGEN**).

>EHO9884747.1 cell division inhibitor SulA [Salmonella enterica subsp. enterica serovar Infantis] Overall Protective Antigen Prediction = **0.3215** (Probable **NON-ANTIGEN**).

>EHO9884748.1 TfoX/Sxy family DNA transformation protein [Salmonella enterica subsp. enterica serovar Infantis] Overall Protective Antigen Prediction = **0.3161** (Probable **NON-ANTIGEN**).

>EHO9884749.1 TIGR01666 family membrane protein [Salmonella enterica subsp. enterica serovar Infantis] Overall Protective Antigen Prediction = **0.5875** (Probable **ANTIGEN**).

>EHO9884750.1 YccF domain-containing protein [Salmonella enterica subsp. enterica serovar Infantis] Overall Protective Antigen Prediction = **0.6634** (Probable **ANTIGEN**).

>EHO9884751.1 DNA helicase IV [Salmonella enterica subsp. enterica serovar Infantis] Overall Protective Antigen Prediction = **0.3026** (Probable **NON-ANTIGEN**).

>EHO9884752.1 methylglyoxal synthase [Salmonella enterica subsp. enterica serovar Infantis] Overall Protective Antigen Prediction = **0.3841** (Probable **NON-ANTIGEN**).

>EHO9884753.1 DUF2057 family protein [Salmonella enterica subsp. enterica serovar Infantis]

Overall Protective Antigen Prediction = **0.5841** (Probable **ANTIGEN**).

>EHO9884754.1 CoA-binding protein [Salmonella enterica subsp. enterica serovar Infantis] Overall Protective Antigen Prediction = **0.1995** (Probable **NON-ANTIGEN**).

>EHO9884755.1 heat shock protein HspQ [Salmonella enterica subsp. enterica serovar Infantis] Overall Protective Antigen Prediction = **0.5969** (Probable **ANTIGEN**).

>EHO9884756.1 23S rRNA (cytosine(1962)-C(5))-methyltransferase RlmI [Salmonella enterica subsp. enterica serovar Infantis] Overall Protective Antigen Prediction = **0.3222** (Probable **NON-ANTIGEN**).

>EHO9884757.1 YbhB/YbcL family Raf kinase inhibitor-like protein [Salmonella enterica subsp. enterica serovar Infantis] Overall Protective Antigen Prediction = **0.6130** (Probable **ANTIGEN**).

>EHO9884758.1 helix-turn-helix transcriptional regulator [Salmonella enterica subsp. enterica serovar Infantis] Overall Protective Antigen Prediction = **0.3116** (Probable **NON-ANTIGEN**).

>EHO9884759.1 acylphosphatase [Salmonella enterica subsp. enterica serovar Infantis] Overall Protective Antigen Prediction = **0.5829** (Probable **ANTIGEN**).

>EHO9884760.1 sulfurtransferase TusE [Salmonella enterica subsp. enterica serovar Infantis] Overall Protective Antigen Prediction = **0.4303** (Probable **ANTIGEN**).

>EHO9884761.1 FtsH protease modulator YccA [Salmonella enterica subsp. enterica serovar Infantis] Overall Protective Antigen Prediction = **0.2606** (Probable **NON-ANTIGEN**).

>EHO9884762.1 type III secretion system effector protease PipA [Salmonella enterica subsp. enterica serovar Infantis] Overall Protective Antigen Prediction = **0.4366** (Probable **ANTIGEN**).

>EHO9884763.1 SPI-2 type III secretion system effector PipB [Salmonella enterica subsp. enterica serovar Infantis] Overall Protective Antigen Prediction = **0.5510** (Probable **ANTIGEN**).

>EHO9884764.1 type III secretion system chaperone SigE [Salmonella enterica subsp. enterica serovar Infantis] Overall Protective Antigen Prediction = **0.4659** (Probable **ANTIGEN**).

>EHO9884765.1 SPI-1 type III secretion system effector inositol phosphate phosphatase SopB [Salmonella enterica subsp. enterica serovar Infantis] Overall Protective Antigen Prediction = **0.5071** (Probable **ANTIGEN**).

>EHO9884766.1 YlcI/YnfO family protein [Salmonella enterica subsp. enterica serovar Infantis] Overall Protective Antigen Prediction = **0.6230** (Probable **ANTIGEN**).

>EHO9884767.1 hypothetical protein KND05_000043 [Salmonella enterica subsp. enterica serovar Infantis] Overall Protective Antigen Prediction = **0.7180** (Probable **ANTIGEN**).

>EHO9884768.1 C69 family dipeptidase [Salmonella enterica subsp. enterica serovar Infantis] Overall Protective Antigen Prediction = **0.4402** (Probable **ANTIGEN**).

>EHO9884769.1 heavy metal sensor histidine kinase [Salmonella enterica subsp. enterica serovar Infantis] Overall Protective Antigen Prediction = **0.3417** (Probable **NON-ANTIGEN**).

>EHO9884770.1 response regulator transcription factor HprR [Salmonella enterica subsp. enterica serovar Infantis] Overall Protective Antigen Prediction = **0.5937** (Probable **ANTIGEN**).

>EHO9884771.1 hydroxyisourate hydrolase [Salmonella enterica subsp. enterica serovar Infantis] Overall Protective Antigen Prediction = **0.4996** (Probable **ANTIGEN**).

>EHO9884772.1 4-hydroxyphenylacetate 3-monooxygenase reductase subunit [Salmonella enterica subsp. enterica serovar Infantis] Overall Protective Antigen Prediction = **0.5273** (Probable **ANTIGEN**).

>EHO9884773.1 4-hydroxyphenylacetate 3-monooxygenase, oxygenase component [Salmonella enterica subsp. enterica serovar Infantis] Overall Protective Antigen Prediction = **0.4371** (Probable **ANTIGEN**).

>EHO9884774.1 homoprotocatechuate degradation operon regulator HpaR [Salmonella enterica subsp. enterica serovar Infantis] Overall Protective Antigen Prediction = **0.4241** (Probable **ANTIGEN**).

>EHO9884775.1 4-hydroxyphenylacetate degradation bifunctional isomerase/decarboxylase [Salmonella enterica subsp. enterica serovar Infantis] Overall Protective Antigen Prediction = **0.3649** (Probable **NON-ANTIGEN**).

>EHO9884776.1 5-carboxymethyl-2-hydroxymuconate semialdehyde dehydrogenase [Salmonella enterica subsp. enterica serovar Infantis] Overall Protective Antigen Prediction = **0.5337** (Probable **ANTIGEN**).

>EHO9884777.1 3,4-dihydroxyphenylacetate 2,3-dioxygenase [Salmonella enterica subsp. enterica serovar Infantis] Overall Protective Antigen Prediction = **0.3586** (Probable **NON-ANTIGEN**).

>EHO9884778.1 5-carboxymethyl-2-hydroxymuconate Delta-isomerase [Salmonella enterica subsp. enterica serovar Infantis] Overall Protective Antigen Prediction = **0.4491** (Probable **ANTIGEN**).

>EHO9884779.1 2-oxo-hepta-3-ene-1,7-dioic acid hydratase [Salmonella enterica subsp. enterica serovar Infantis] Overall Protective Antigen Prediction = **0.5853** (Probable **ANTIGEN**).

>EHO9884780.1 4-hydroxy-2-oxoheptanedioate aldolase [Salmonella enterica subsp. enterica serovar Infantis] Overall Protective Antigen Prediction = **0.3634** (Probable **NON-ANTIGEN**).

>EHO9884781.1 4-hydroxyphenylacetate permease [Salmonella enterica subsp. enterica serovar Infantis] Overall Protective Antigen Prediction = **0.4982** (Probable **ANTIGEN**).

>EHO9884782.1 4-hydroxyphenylacetate catabolism regulatory protein HpaA [Salmonella enterica subsp. enterica serovar Infantis] Overall Protective Antigen Prediction = **0.4554** (Probable **ANTIGEN**).

>EHO9884783.1 MBL fold metallo-hydrolase [Salmonella enterica subsp. enterica serovar Infantis] Overall Protective Antigen Prediction = **0.3482** (Probable **NON-ANTIGEN**).

>EHO9884784.1 anti-adapter protein IraM [Salmonella enterica subsp. enterica serovar Infantis] Overall Protective Antigen Prediction = **0.3881** (Probable **NON-ANTIGEN**).

>EHO9884785.1 chaperone modulator CbpM [Salmonella enterica subsp. enterica serovar Infantis] Overall Protective Antigen Prediction = **0.3486** (Probable **NON-ANTIGEN**).

>EHO9884786.1 curved DNA-binding protein [Salmonella enterica subsp. enterica serovar Infantis] Overall Protective Antigen Prediction = **0.6896** (Probable **ANTIGEN**).

>EHO9884787.1 copper resistance protein [Salmonella enterica subsp. enterica serovar Infantis] Overall Protective Antigen Prediction = **0.6530** (Probable **ANTIGEN**).

>EHO9884788.1 thioredoxin family protein [Salmonella enterica subsp. enterica serovar Infantis] Overall Protective Antigen Prediction = **0.5108** (Probable **ANTIGEN**).

>EHO9884789.1 DsbA family protein [Salmonella enterica subsp. enterica serovar Infantis] Overall Protective Antigen Prediction = **0.4722** (Probable **ANTIGEN**).

>EHO9884790.1 protein disulfide oxidoreductase [Salmonella enterica subsp. enterica serovar Infantis] Overall Protective Antigen Prediction = **0.5052** (Probable **ANTIGEN**).

>EHO9884791.1 bifunctional glucose-1-phosphatase/inositol phosphatase [Salmonella enterica subsp. enterica serovar Infantis] Overall Protective Antigen Prediction = **0.3078** (Probable **NON-ANTIGEN**).

>EHO9884792.1 YccJ family protein [Salmonella enterica subsp. enterica serovar Infantis] Overall Protective Antigen Prediction = **0.2495** (Probable **NON-ANTIGEN**).

>EHO9884793.1 NAD(P)H:quinone oxidoreductase [Salmonella enterica subsp. enterica serovar Infantis] Overall Protective Antigen Prediction = **0.5356** (Probable **ANTIGEN**).

>EHO9884794.1 general stress protein [Salmonella enterica subsp. enterica serovar Infantis] Overall Protective Antigen Prediction = **1.6197** (Probable **ANTIGEN**).

>EHO9884795.1 HTH-type transcriptional regulator RutR [Salmonella enterica subsp. enterica serovar Infantis] Overall Protective Antigen Prediction = **0.4395** (Probable **ANTIGEN**).

>EHO9884796.1 lysozyme inhibitor LprI family protein [Salmonella enterica subsp. enterica serovar Infantis] Overall Protective Antigen Prediction = **0.4770** (Probable **ANTIGEN**).

>EHO9884797.1 trifunctional transcriptional regulator/proline dehydrogenase/L-glutamate gamma-semialdehyde dehydrogenase [Salmonella enterica subsp. enterica serovar Infantis] Overall Protective Antigen Prediction = **0.4128** (Probable **ANTIGEN**).

>EHO9884798.1 sodium/proline symporter PutP [Salmonella enterica subsp. enterica serovar Infantis] Overall Protective Antigen Prediction = **0.3699** (Probable **NON-ANTIGEN**).

>EHO9884799.1 phosphate starvation-inducible protein PhoH [Salmonella enterica subsp. enterica serovar Infantis] Overall Protective Antigen Prediction = **0.3468** (Probable **NON-ANTIGEN**).

>EHO9884800.1 MurR/RpiR family transcriptional regulator [Salmonella enterica subsp. enterica serovar Infantis] Overall Protective Antigen Prediction = **0.5818** (Probable **ANTIGEN**).

>EHO9884801.1 sodium:solute symporter [Salmonella enterica subsp. enterica serovar Infantis] Overall Protective Antigen Prediction = **0.5689** (Probable **ANTIGEN**).

>EHO9884802.1 N-acetylmannosamine-6-phosphate 2-epimerase [Salmonella enterica subsp. enterica serovar Infantis] Overall Protective Antigen Prediction = **0.3953** (Probable **NON-ANTIGEN**).

>EHO9884803.1 N-acetylneuraminate epimerase [Salmonella enterica subsp. enterica serovar Infantis] Overall Protective Antigen Prediction = **0.3500** (Probable **NON-ANTIGEN**).

>EHO9884804.1 outer membrane protein [Salmonella enterica subsp. enterica serovar Infantis] Overall Protective Antigen Prediction = **0.7383** (Probable **ANTIGEN**).

>EHO9884805.1 MFS transporter [Salmonella enterica subsp. enterica serovar Infantis] Overall Protective Antigen Prediction = **0.6417** (Probable **ANTIGEN**).

>EHO9884806.1 Gfo/Idh/MocA family oxidoreductase [Salmonella enterica subsp. enterica serovar Infantis] Overall Protective Antigen Prediction = **0.3929** (Probable **NON-ANTIGEN**).

>EHO9884807.1 glyoxylate/hydroxypyruvate reductase GhrA [Salmonella enterica subsp. enterica serovar Infantis] Overall Protective Antigen Prediction = **0.3595** (Probable **NON-ANTIGEN**).

>EHO9884808.1 phosphatase [Salmonella enterica subsp. enterica serovar Infantis] Overall Protective Antigen Prediction = **0.3954** (Probable **NON-ANTIGEN**).

>EHO9884809.1 molecular chaperone [Salmonella enterica subsp. enterica serovar Infantis] Overall Protective Antigen Prediction = **0.2674** (Probable **NON-ANTIGEN**).

>EHO9884810.1 DUF1097 domain-containing protein [Salmonella enterica subsp. enterica serovar Infantis] Overall Protective Antigen Prediction = **0.3084** (Probable **NON-ANTIGEN**).

>EHO9884811.1 curli production assembly/transport protein CsgG [Salmonella enterica subsp. enterica serovar Infantis] Overall Protective Antigen Prediction = **0.3397** (Probable **NON-ANTIGEN**).

>EHO9884812.1 curli production assembly/transport protein CsgF [Salmonella enterica subsp. enterica serovar Infantis] Overall Protective Antigen Prediction = **0.6214** (Probable **ANTIGEN**).

>EHO9884813.1 curli production assembly/transport protein CsgE [Salmonella enterica subsp. enterica serovar Infantis] Overall Protective Antigen Prediction = **0.3203** (Probable **NON-ANTIGEN**).

>EHO9884814.1 transcriptional regulator CsgD [Salmonella enterica subsp. enterica serovar Infantis] Overall Protective Antigen Prediction = **0.1737** (Probable **NON-ANTIGEN**).

>EHO9884815.1 curlin minor subunit CsgB [Salmonella enterica subsp. enterica serovar Infantis] Overall Protective Antigen Prediction = **0.6452** (Probable **ANTIGEN**).

>EHO9884816.1 curlin major subunit CsgA [Salmonella enterica subsp. enterica serovar Infantis] Overall Protective Antigen Prediction = **1.0694** (Probable **ANTIGEN**).

>EHO9884817.1 curli assembly protein CsgC [Salmonella enterica subsp. enterica serovar Infantis] Overall Protective Antigen Prediction = **0.7002** (Probable **ANTIGEN**).

>EHO9884818.1 type 1 fimbrial protein [Salmonella enterica subsp. enterica serovar Infantis] Overall Protective Antigen Prediction = **0.6914** (Probable **ANTIGEN**).

>EHO9884819.1 O-acetyl-ADP-ribose deacetylase [Salmonella enterica subsp. enterica serovar Infantis] Overall Protective Antigen Prediction = **0.4205** (Probable **ANTIGEN**).

>EHO9884820.1 phospholipase D family protein [Salmonella enterica subsp. enterica serovar Infantis] Overall Protective Antigen Prediction = **0.4121** (Probable **ANTIGEN**).

>EHO9884821.1 glucans biosynthesis protein MdoC [Salmonella enterica subsp. enterica serovar

Infantis] Overall Protective Antigen Prediction = **0.6595** (Probable **ANTIGEN**).

>EHO9884822.1 glucans biosynthesis protein MdoG [Salmonella enterica subsp. enterica serovar Infantis] Overall Protective Antigen Prediction = **0.4812** (Probable **ANTIGEN**).

>EHO9884823.1 glucans biosynthesis glucosyltransferase MdoH [Salmonella enterica subsp. enterica serovar Infantis] Overall Protective Antigen Prediction = **0.4061** (Probable **ANTIGEN**).

>EHO9884824.1 YceK/YidQ family lipoprotein [Salmonella enterica subsp. enterica serovar Infantis] Overall Protective Antigen Prediction = **0.9588** (Probable **ANTIGEN**).

>EHO9884825.1 secY/secA suppressor protein [Salmonella enterica subsp. enterica serovar Infantis] Overall Protective Antigen Prediction = **0.6174** (Probable **ANTIGEN**).

>EHO9884826.1 multidrug efflux MFS transporter MdtG [Salmonella enterica subsp. enterica serovar Infantis] Overall Protective Antigen Prediction = **0.4576** (Probable **ANTIGEN**).

>EHO9884827.1 LpxL/LpxP family Kdo(2)-lipid IV(A) lauroyl/palmitoleoyl acyltransferasee [Salmonella enterica subsp. enterica serovar Infantis] Overall Protective Antigen Prediction = **0.4347** (Probable **ANTIGEN**).

>EHO9884828.1 rhodanese-related sulfurtransferase [Salmonella enterica subsp. enterica serovar Infantis] Overall Protective Antigen Prediction = **0.4390** (Probable **ANTIGEN**).

>EHO9884829.1 YceI family protein [Salmonella enterica subsp. enterica serovar Infantis] Overall Protective Antigen Prediction = **0.7140** (Probable **ANTIGEN**).

>EHO9884830.1 cytochrome b [Salmonella enterica subsp. enterica serovar Infantis] Overall Protective Antigen Prediction = **0.6074** (Probable **ANTIGEN**).

>EHO9884831.1 YceO family protein [Salmonella enterica subsp. enterica serovar Infantis] Overall Protective Antigen Prediction = **1.0258** (Probable **ANTIGEN**).

>EHO9884832.1 N-methyl-L-tryptophan oxidase [Salmonella enterica subsp. enterica serovar Infantis] Overall Protective Antigen Prediction = **0.4007** (Probable **ANTIGEN**).

>EHO9884833.1 biofilm formation regulator BssS [Salmonella enterica subsp. enterica serovar Infantis] Overall Protective Antigen Prediction = **0.5963** (Probable **ANTIGEN**).

>EHO9884834.1 DNA damage-inducible protein I [Salmonella enterica subsp. enterica serovar Infantis] Overall Protective Antigen Prediction = **0.4561** (Probable **ANTIGEN**).

>EHO9884835.1 dihydroorotase [Salmonella enterica subsp. enterica serovar Infantis] Overall Protective Antigen Prediction = **0.3119** (Probable **NON-ANTIGEN**).

>EHO9884836.1 lipoprotein [Salmonella enterica subsp. enterica serovar Infantis] Overall Protective Antigen Prediction = **0.4802** (Probable **ANTIGEN**).

>EHO9884837.1 glutaredoxin 2 [Salmonella enterica subsp. enterica serovar Infantis] Overall Protective Antigen Prediction = **0.3814** (Probable **NON-ANTIGEN**).

>EHO9884838.1 multidrug efflux MFS transporter MdtH [Salmonella enterica subsp. enterica serovar Infantis] Overall Protective Antigen Prediction = **0.4652** (Probable **ANTIGEN**).

>EHO9884839.1 ribosomal protein S5-alanine N-acetyltransferase [Salmonella enterica subsp. enterica serovar Infantis] Overall Protective Antigen Prediction = **0.2137** (Probable **NON-ANTIGEN**).

>EHO9884840.1 YceH family protein [Salmonella enterica subsp. enterica serovar Infantis] Overall Protective Antigen Prediction = **0.5711** (Probable **ANTIGEN**).

>EHO9884841.1 Gfo/Idh/MocA family oxidoreductase [Salmonella enterica subsp. enterica serovar Infantis] Overall Protective Antigen Prediction = **0.3920** (Probable **NON-ANTIGEN**).

>EHO9884842.1 murein biosynthesis integral membrane protein MurJ [Salmonella enterica subsp. enterica serovar Infantis] Overall Protective Antigen Prediction = **0.3998** (Probable **NON-ANTIGEN**).

>EHO9884843.1 flagella biosynthesis chaperone FlgN [Salmonella enterica subsp. enterica serovar Infantis] Overall Protective Antigen Prediction = **0.4412** (Probable **ANTIGEN**).

>EHO9884844.1 anti-sigma-28 factor FlgM [Salmonella enterica subsp. enterica serovar Infantis] Overall Protective Antigen Prediction = **0.6252** (Probable **ANTIGEN**).

>EHO9884845.1 flagellar basal body P-ring formation protein FlgA [Salmonella enterica subsp. enterica serovar Infantis] Overall Protective Antigen Prediction = **0.6017** (Probable **ANTIGEN**).

>EHO9884846.1 flagellar basal body rod protein FlgB [Salmonella enterica subsp. enterica serovar Infantis] Overall Protective Antigen Prediction = **0.6665** (Probable **ANTIGEN**).

>EHO9884847.1 flagellar basal body rod protein FlgC [Salmonella enterica subsp. enterica serovar Infantis] Overall Protective Antigen Prediction = **0.6184** (Probable **ANTIGEN**).

>EHO9884848.1 flagellar hook assembly protein FlgD [Salmonella enterica subsp. enterica serovar Infantis] Overall Protective Antigen Prediction = **0.6794** (Probable **ANTIGEN**).

>EHO9884849.1 flagellar hook protein FlgE [Salmonella enterica subsp. enterica serovar Infantis] Overall Protective Antigen Prediction = **0.6687** (Probable **ANTIGEN**).

>EHO9884850.1 flagellar basal body rod protein FlgF [Salmonella enterica subsp. enterica serovar Infantis] Overall Protective Antigen Prediction = **0.6603** (Probable **ANTIGEN**).

>EHO9884851.1 flagellar basal-body rod protein FlgG [Salmonella enterica subsp. enterica serovar Infantis] Overall Protective Antigen Prediction = **0.5955** (Probable **ANTIGEN**).

>EHO9884852.1 flagellar basal body L-ring protein FlgH [Salmonella enterica subsp. enterica serovar Infantis] Overall Protective Antigen Prediction = **0.5735** (Probable **ANTIGEN**).

>EHO9884853.1 flagellar basal body P-ring protein FlgI [Salmonella enterica subsp. enterica serovar Infantis] Overall Protective Antigen Prediction = **0.7507** (Probable **ANTIGEN**).

>EHO9884854.1 flagellar assembly peptidoglycan hydrolase FlgJ [Salmonella enterica subsp. enterica serovar Infantis] Overall Protective Antigen Prediction = **0.4341** (Probable **ANTIGEN**).

>EHO9884855.1 flagellar hook-associated protein FlgK [Salmonella enterica subsp. enterica serovar Infantis] Overall Protective Antigen Prediction = **0.6029** (Probable **ANTIGEN**).

>EHO9884856.1 flagellar hook-associated protein FlgL [Salmonella enterica subsp. enterica serovar

Infantis] Overall Protective Antigen Prediction = **0.5057** (Probable **ANTIGEN**).

>EHO9884857.1 ribonuclease E [Salmonella enterica subsp. enterica serovar Infantis] Overall Protective Antigen Prediction = **0.6846** (Probable **ANTIGEN**).

>EHO9884858.1 23S rRNA pseudouridine(955/2504/2580) synthase RluC [Salmonella enterica subsp. enterica serovar Infantis] Overall Protective Antigen Prediction = **0.5395** (Probable **ANTIGEN**).

>EHO9884859.1 septum formation inhibitor Maf [Salmonella enterica subsp. enterica serovar Infantis] Overall Protective Antigen Prediction = **0.4341** (Probable **ANTIGEN**).

>EHO9884860.1 23S rRNA accumulation protein YceD [Salmonella enterica subsp. enterica serovar Infantis] Overall Protective Antigen Prediction = **0.4543** (Probable **ANTIGEN**).

>EHO9884861.1 50S ribosomal protein L32 [Salmonella enterica subsp. enterica serovar Infantis] Overall Protective Antigen Prediction = **0.7637** (Probable **ANTIGEN**).

>EHO9884862.1 phosphate acyltransferase PlsX [Salmonella enterica subsp. enterica serovar Infantis] Overall Protective Antigen Prediction = **0.5287** (Probable **ANTIGEN**).

>EHO9884863.1 beta-ketoacyl-ACP synthase III [Salmonella enterica subsp. enterica serovar Infantis] Overall Protective Antigen Prediction = **0.4630** (Probable **ANTIGEN**).

>EHO9884864.1 ACP S-malonyltransferase [Salmonella enterica subsp. enterica serovar Infantis] Overall Protective Antigen Prediction = **0.4498** (Probable **ANTIGEN**).

>EHO9884865.1 3-oxoacyl-ACP reductase FabG [Salmonella enterica subsp. enterica serovar Infantis] Overall Protective Antigen Prediction = **0.4154** (Probable **ANTIGEN**).

>EHO9884866.1 acyl carrier protein [Salmonella enterica subsp. enterica serovar Infantis] Overall Protective Antigen Prediction = **0.5103** (Probable **ANTIGEN**).

>EHO9884867.1 beta-ketoacyl-ACP synthase II [Salmonella enterica subsp. enterica serovar Infantis] Overall Protective Antigen Prediction = **0.4853** (Probable **ANTIGEN**).

>EHO9884868.1 aminodeoxychorismate lyase [Salmonella enterica subsp. enterica serovar Infantis] Overall Protective Antigen Prediction = **0.5646** (Probable **ANTIGEN**).

>EHO9884869.1 cell division protein YceG [Salmonella enterica subsp. enterica serovar Infantis] Overall Protective Antigen Prediction = **0.4664** (Probable **ANTIGEN**).

>EHO9884870.1 dTMP kinase [Salmonella enterica subsp. enterica serovar Infantis] Overall Protective Antigen Prediction = **0.5556** (Probable **ANTIGEN**).

>EHO9884871.1 DNA polymerase III subunit delta' [Salmonella enterica subsp. enterica serovar Infantis] Overall Protective Antigen Prediction = **0.3695** (Probable **NON-ANTIGEN**).

>EHO9884872.1 metal-dependent hydrolase [Salmonella enterica subsp. enterica serovar Infantis] Overall Protective Antigen Prediction = **0.3659** (Probable **NON-ANTIGEN**).

>EHO9884873.1 PTS glucose transporter subunit IIBC [Salmonella enterica subsp. enterica serovar Infantis] Overall Protective Antigen Prediction = **0.4787** (Probable **ANTIGEN**).

>EHO9884874.1 ferric-rhodotorulic acid/ferric-coprogen receptor FhuE [Salmonella enterica subsp. enterica serovar Infantis] Overall Protective Antigen Prediction = **0.5054** (Probable **ANTIGEN**).

>EHO9884875.1 purine nucleoside phosphoramidase [Salmonella enterica subsp. enterica serovar Infantis] Overall Protective Antigen Prediction = **0.3983** (Probable **NON-ANTIGEN**).

>EHO9884876.1 YcfL family protein [Salmonella enterica subsp. enterica serovar Infantis] Overall Protective Antigen Prediction = **0.5407** (Probable **ANTIGEN**).

>EHO9884877.1 penicillin-binding protein activator LpoB [Salmonella enterica subsp. enterica serovar Infantis] Overall Protective Antigen Prediction = **0.4658** (Probable **ANTIGEN**).

>EHO9884878.1 thiamine kinase [Salmonella enterica subsp. enterica serovar Infantis] Overall Protective Antigen Prediction = **0.6255** (Probable **ANTIGEN**).

>EHO9884879.1 beta-N-acetylhexosaminidase [Salmonella enterica subsp. enterica serovar Infantis] Overall Protective Antigen Prediction = **0.4768** (Probable **ANTIGEN**).

>EHO9884880.1 alpha/beta hydrolase [Salmonella enterica subsp. enterica serovar Infantis] Overall Protective Antigen Prediction = **0.3493** (Probable **NON-ANTIGEN**).

>EHO9884881.1 NADH-quinone dehydrogenase [Salmonella enterica subsp. enterica serovar Infantis] Overall Protective Antigen Prediction = **0.4876** (Probable **ANTIGEN**).

>EHO9884882.1 glycine zipper 2TM domain-containing protein [Salmonella enterica subsp. enterica serovar Infantis] Overall Protective Antigen Prediction = **0.6686** (Probable **ANTIGEN**).

>EHO9884883.1 TetR family copper-responsive transcriptional repressor ComR [Salmonella enterica subsp. enterica serovar Infantis] Overall Protective Antigen Prediction = **0.2567** (Probable **NON-ANTIGEN**).

>EHO9884884.1 DUF1471 domain-containing protein [Salmonella enterica subsp. enterica serovar Infantis] Overall Protective Antigen Prediction = **0.5632** (Probable **ANTIGEN**).

>EHO9884885.1 L,D-transpeptidase family protein [Salmonella enterica subsp. enterica serovar Infantis] Overall Protective Antigen Prediction = **0.6443** (Probable **ANTIGEN**).

>EHO9884886.1 transcription-repair coupling factor [Salmonella enterica subsp. enterica serovar Infantis] Overall Protective Antigen Prediction = **0.4447** (Probable **ANTIGEN**).

>EHO9884887.1 lipoprotein-releasing ABC transporter permease subunit LolC [Salmonella enterica subsp. enterica serovar Infantis] Overall Protective Antigen Prediction = **0.3302** (Probable **NON-ANTIGEN**).

>EHO9884888.1 lipoprotein-releasing ABC transporter ATP-binding protein LolD [Salmonella enterica subsp. enterica serovar Infantis] Overall Protective Antigen Prediction = **0.6138** (Probable **ANTIGEN**).

>EHO9884889.1 lipoprotein-releasing ABC transporter permease subunit LolE [Salmonella enterica subsp. enterica serovar Infantis] Overall Protective Antigen Prediction = **0.4078** (Probable **ANTIGEN**).

>EHO9884890.1 N-acetylglucosamine kinase [Salmonella enterica subsp. enterica serovar Infantis] Overall Protective Antigen Prediction = **0.5766** (Probable **ANTIGEN**).

>EHO9884891.1 NAD-dependent protein deacylase [Salmonella enterica subsp. enterica serovar Infantis] Overall Protective Antigen Prediction = **0.3314** (Probable **NON-ANTIGEN**).

>EHO9884892.1 spermidine/putrescine ABC transporter substrate-binding protein PotD [Salmonella enterica subsp. enterica serovar Infantis] Overall Protective Antigen Prediction = **0.2745** (Probable **NON-ANTIGEN**).

>EHO9884893.1 spermidine/putrescine ABC transporter permease PotC [Salmonella enterica subsp. enterica serovar Infantis] Overall Protective Antigen Prediction = **0.6211** (Probable **ANTIGEN**).

>EHO9884894.1 SPI-2 type III secretion system effector SifA [Salmonella enterica subsp. enterica serovar Infantis] Overall Protective Antigen Prediction = **0.3781** (Probable **NON-ANTIGEN**).

>EHO9884895.1 spermidine/putrescine ABC transporter permease PotB [Salmonella enterica subsp. enterica serovar Infantis] Overall Protective Antigen Prediction = **0.6116** (Probable **ANTIGEN**).

>EHO9884896.1 spermidine/putrescine ABC transporter ATP-binding protein PotA [Salmonella enterica subsp. enterica serovar Infantis] Overall Protective Antigen Prediction = **0.4689** (Probable **ANTIGEN**).

>EHO9884897.1 peptidase T [Salmonella enterica subsp. enterica serovar Infantis] Overall Protective Antigen Prediction = **0.6373** (Probable **ANTIGEN**).

>EHO9884898.1 hypothetical protein KND05_000176 [Salmonella enterica subsp. enterica serovar Infantis] Overall Protective Antigen Prediction = **0.4112** (Probable **ANTIGEN**).

>EHO9884899.1 cupin domain-containing protein [Salmonella enterica subsp. enterica serovar Infantis] Overall Protective Antigen Prediction = **0.3448** (Probable **NON-ANTIGEN**).

>EHO9884900.1 two-component system sensor histidine kinase PhoQ [Salmonella enterica subsp. enterica serovar Infantis] Overall Protective Antigen Prediction = **0.4361** (Probable **ANTIGEN**).

>EHO9884901.1 two-component system response regulator PhoP [Salmonella enterica subsp. enterica serovar Infantis] Overall Protective Antigen Prediction = **0.5751** (Probable **ANTIGEN**).

>EHO9884902.1 adenylosuccinate lyase [Salmonella enterica subsp. enterica serovar Infantis] Overall Protective Antigen Prediction = **0.2953** (Probable **NON-ANTIGEN**).

>EHO9884903.1 high frequency lysogenization protein HflD [Salmonella enterica subsp. enterica serovar Infantis] Overall Protective Antigen Prediction = **0.5881** (Probable **ANTIGEN**).

>EHO9884904.1 tRNA 2-thiouridine(34) synthase MnmA [Salmonella enterica subsp. enterica serovar Infantis] Overall Protective Antigen Prediction = **0.5082** (Probable **ANTIGEN**).

>EHO9884905.1 NUDIX hydrolase [Salmonella enterica subsp. enterica serovar Infantis] Overall Protective Antigen Prediction = **0.4589** (Probable **ANTIGEN**).

>EHO9884906.1 DUF1311 domain-containing protein [Salmonella enterica subsp. enterica serovar Infantis] Overall Protective Antigen Prediction = **0.4479** (Probable **ANTIGEN**).

>EHO9884907.1 23S rRNA pseudouridine(2457) synthase RluE [Salmonella enterica subsp. enterica serovar Infantis] Overall Protective Antigen Prediction = **0.4219** (Probable **ANTIGEN**).

>EHO9884908.1 NADP-dependent isocitrate dehydrogenase [Salmonella enterica subsp. enterica serovar Infantis] Overall Protective Antigen Prediction = **0.4788** (Probable **ANTIGEN**).

>EHO9884909.1 type III secretion system effector SopF [Salmonella enterica subsp. enterica serovar Infantis] Overall Protective Antigen Prediction = **0.4302** (Probable **ANTIGEN**).

>EHO9884910.1 lipoprotein EnvF [Salmonella enterica subsp. enterica serovar Infantis] Overall Protective Antigen Prediction = **0.4758** (Probable **ANTIGEN**).

>EHO9884911.1 virulence protein MsgA [Salmonella enterica subsp. enterica serovar Infantis] Overall Protective Antigen Prediction = **0.5134** (Probable **ANTIGEN**).

>EHO9884912.1 ricin-type beta-trefoil lectin domain protein [Salmonella enterica subsp. enterica serovar Infantis] Overall Protective Antigen Prediction = **0.6210** (Probable **ANTIGEN**).

>EHO9884913.1 cold shock-like protein CspF [Salmonella enterica subsp. enterica serovar Infantis] Overall Protective Antigen Prediction = **0.6221** (Probable **ANTIGEN**).

>EHO9884914.1 virulence protein PagD [Salmonella enterica subsp. enterica serovar Infantis] Overall Protective Antigen Prediction = **0.9662** (Probable **ANTIGEN**).

>EHO9884915.1 virulence membrane protein PagC [Salmonella enterica subsp. enterica serovar Infantis] Overall Protective Antigen Prediction = **0.7070** (Probable **ANTIGEN**).

>EHO9884916.1 c-type lysozyme inhibitor [Salmonella enterica subsp. enterica serovar Infantis] Overall Protective Antigen Prediction = **0.5184** (Probable **ANTIGEN**).

>EHO9884917.1 hypothetical protein KND05_000196 [Salmonella enterica subsp. enterica serovar Infantis] Overall Protective Antigen Prediction = **0.5491** (Probable **ANTIGEN**).

>EHO9884918.1 Hsp20 family protein [Salmonella enterica subsp. enterica serovar Infantis] Overall Protective Antigen Prediction = **0.6190** (Probable **ANTIGEN**).

>EHO9884919.1 exo-alpha-sialidase [Salmonella enterica subsp. enterica serovar Infantis] Overall Protective Antigen Prediction = **0.2489** (Probable **NON-ANTIGEN**).

>EHO9884920.1 cytochrome b [Salmonella enterica subsp. enterica serovar Infantis] Overall Protective Antigen Prediction = **0.6650** (Probable **ANTIGEN**).

>EHO9884921.1 TRL-like family protein [Salmonella enterica subsp. enterica serovar Infantis] Overall Protective Antigen Prediction = **0.5133** (Probable **ANTIGEN**).

>EHO9884922.1 ABC transporter substrate-binding protein [Salmonella enterica subsp. enterica serovar Infantis] Overall Protective Antigen Prediction = **0.5355** (Probable **ANTIGEN**).

>EHO9884923.1 ABC transporter permease [Salmonella enterica subsp. enterica serovar Infantis] Overall Protective Antigen Prediction = **0.3744** (Probable **NON-ANTIGEN**).

>EHO9884924.1 ABC transporter permease [Salmonella enterica subsp. enterica serovar Infantis] Overall Protective Antigen Prediction = **0.5918** (Probable **ANTIGEN**).

>EHO9884925.1 ATP-binding cassette domain-containing protein [Salmonella enterica subsp. enterica serovar Infantis] Overall Protective Antigen Prediction = **0.4125** (Probable **ANTIGEN**).

>EHO9884926.1 ATP-binding cassette domain-containing protein [Salmonella enterica subsp. enterica serovar Infantis] Overall Protective Antigen Prediction = **0.5747** (Probable **ANTIGEN**).

>EHO9884927.1 mechanosensitive ion channel family protein [Salmonella enterica subsp. enterica serovar Infantis] Overall Protective Antigen Prediction = **0.4838** (Probable **ANTIGEN**).

>EHO9884928.1 four-helix bundle copper-binding protein [Salmonella enterica subsp. enterica serovar Infantis] Overall Protective Antigen Prediction = **0.6535** (Probable **ANTIGEN**).

>EHO9884929.1 metal-binding protein ZinT [Salmonella enterica subsp. enterica serovar Infantis] Overall Protective Antigen Prediction = **0.5051** (Probable **ANTIGEN**).

>EHO9884930.1 cryptic aminoglycoside nucleotidyltransferase ANT(3")/ANT(9) [Salmonella enterica subsp. enterica serovar Infantis] Overall Protective Antigen Prediction = **0.4341** (Probable **ANTIGEN**).

>EHO9884931.1 helix-turn-helix transcriptional regulator [Salmonella enterica subsp. enterica serovar Infantis] Overall Protective Antigen Prediction = **0.3125** (Probable **NON-ANTIGEN**).

>EHO9884932.1 MerR family transcriptional regulator [Salmonella enterica subsp. enterica serovar Infantis] Overall Protective Antigen Prediction = **0.3948** (Probable **NON-ANTIGEN**).

>EHO9884933.1 histidine kinase [Salmonella enterica subsp. enterica serovar Infantis] Overall Protective Antigen Prediction = **0.2722** (Probable **NON-ANTIGEN**).

>EHO9884934.1 chorismate mutase [Salmonella enterica subsp. enterica serovar Infantis] Overall Protective Antigen Prediction = **0.2585** (Probable **NON-ANTIGEN**).

>EHO9884935.1 leucine efflux protein LeuE [Salmonella enterica subsp. enterica serovar Infantis] Overall Protective Antigen Prediction = **0.2595** (Probable **NON-ANTIGEN**).

>EHO9884936.1 DUF1971 domain-containing protein [Salmonella enterica subsp. enterica serovar Infantis] Overall Protective Antigen Prediction = **0.2622** (Probable **NON-ANTIGEN**).

>EHO9884937.1 DUF1869 domain-containing protein [Salmonella enterica subsp. enterica serovar Infantis] Overall Protective Antigen Prediction = **0.6014** (Probable **ANTIGEN**).

>EHO9884938.1 hypothetical protein KND05_000218 [Salmonella enterica subsp. enterica serovar Infantis] Overall Protective Antigen Prediction = **0.4876** (Probable **ANTIGEN**).

>EHO9884939.1 GlsB/YeaQ/YmgE family stress response membrane protein [Salmonella enterica subsp. enterica serovar Infantis] Overall Protective Antigen Prediction = **0.1017** (Probable **NON-ANTIGEN**).

>EHO9884940.1 YoaK family small membrane protein [Salmonella enterica subsp. enterica serovar Infantis] Overall Protective Antigen Prediction = **1.1851** (Probable **ANTIGEN**).

>EHO9884941.1 DUF333 domain-containing protein [Salmonella enterica subsp. enterica serovar Infantis] Overall Protective Antigen Prediction = **0.5634** (Probable **ANTIGEN**).

>EHO9884942.1 hypothetical protein KND05_000222 [Salmonella enterica subsp. enterica serovar Infantis] Overall Protective Antigen Prediction = **0.5611** (Probable **ANTIGEN**).

>EHO9884943.1 DUF488 domain-containing protein [Salmonella enterica subsp. enterica serovar

Infantis] Overall Protective Antigen Prediction = **0.3343** (Probable **NON-ANTIGEN**).

>EHO9884944.1 CynX/NimT family MFS transporter [Salmonella enterica subsp. enterica serovar Infantis] Overall Protective Antigen Prediction = **0.5413** (Probable **ANTIGEN**).

>EHO9884945.1 helix-turn-helix transcriptional regulator [Salmonella enterica subsp. enterica serovar Infantis] Overall Protective Antigen Prediction = **0.3846** (Probable **NON-ANTIGEN**).

>EHO9884946.1 DUF441 domain-containing protein [Salmonella enterica subsp. enterica serovar Infantis] Overall Protective Antigen Prediction = **0.1834** (Probable **NON-ANTIGEN**).

>EHO9884947.1 hypothetical protein KND05_000227 [Salmonella enterica subsp. enterica serovar Infantis] Overall Protective Antigen Prediction = **0.9794** (Probable **ANTIGEN**).

>EHO9884948.1 YbaK/prolyl-tRNA synthetase associated domain-containing protein [Salmonella enterica subsp. enterica serovar Infantis] Overall Protective Antigen Prediction = **0.5102** (Probable **ANTIGEN**).

>EHO9884949.1 GGDEF domain-containing protein [Salmonella enterica subsp. enterica serovar Infantis] Overall Protective Antigen Prediction = **0.3983** (Probable **NON-ANTIGEN**).

>EHO9884950.1 hypothetical protein KND05_000230 [Salmonella enterica subsp. enterica serovar Infantis] Overall Protective Antigen Prediction = **0.3604** (Probable **NON-ANTIGEN**).

>EHO9884951.1 YeaH/YhbH family protein [Salmonella enterica subsp. enterica serovar Infantis] Overall Protective Antigen Prediction = **0.6762** (Probable **ANTIGEN**).

>EHO9884952.1 protein kinase YeaG [Salmonella enterica subsp. enterica serovar Infantis] Overall Protective Antigen Prediction = **0.3620** (Probable **NON-ANTIGEN**).

>EHO9884953.1 MipA/OmpV family protein [Salmonella enterica subsp. enterica serovar Infantis] Overall Protective Antigen Prediction = **0.6008** (Probable **ANTIGEN**).

>EHO9884954.1 anaerobic sulfatase maturase [Salmonella enterica subsp. enterica serovar Infantis] Overall Protective Antigen Prediction = **0.3978** (Probable **NON-ANTIGEN**).

>EHO9884955.1 aldo/keto reductase [Salmonella enterica subsp. enterica serovar Infantis] Overall Protective Antigen Prediction = **0.4212** (Probable **ANTIGEN**).

>EHO9884956.1 D-hexose-6-phosphate mutarotase [Salmonella enterica subsp. enterica serovar Infantis] Overall Protective Antigen Prediction = **0.5445** (Probable **ANTIGEN**).

>EHO9884957.1 glyceraldehyde-3-phosphate dehydrogenase [Salmonella enterica subsp. enterica serovar Infantis] Overall Protective Antigen Prediction = **0.5535** (Probable **ANTIGEN**).

>EHO9884958.1 peptide-methionine (R)-S-oxide reductase MsrB [Salmonella enterica subsp. enterica serovar Infantis] Overall Protective Antigen Prediction = **0.4934** (Probable **ANTIGEN**).

>EHO9884959.1 YeaC family protein [Salmonella enterica subsp. enterica serovar Infantis] Overall Protective Antigen Prediction = **0.5900** (Probable **ANTIGEN**).

>EHO9884960.1 bifunctional nicotinamidase/pyrazinamidase [Salmonella enterica subsp. enterica serovar Infantis] Overall Protective Antigen Prediction = **0.3264** (Probable **NON-ANTIGEN**).

>EHO9884961.1 asparaginase [Salmonella enterica subsp. enterica serovar Infantis] Overall Protective Antigen Prediction = **0.3292** (Probable **NON-ANTIGEN**).

>EHO9884962.1 signal peptide peptidase SppA [Salmonella enterica subsp. enterica serovar Infantis] Overall Protective Antigen Prediction = **0.5029** (Probable **ANTIGEN**).

>EHO9884963.1 NAD(P)H nitroreductase [Salmonella enterica subsp. enterica serovar Infantis] Overall Protective Antigen Prediction = **0.3541** (Probable **NON-ANTIGEN**).

>EHO9884964.1 selenide, water dikinase SelD [Salmonella enterica subsp. enterica serovar Infantis] Overall Protective Antigen Prediction = **0.4587** (Probable **ANTIGEN**).

>EHO9884965.1 DNA topoisomerase III [Salmonella enterica subsp. enterica serovar Infantis] Overall Protective Antigen Prediction = **0.3407** (Probable **NON-ANTIGEN**).

>EHO9884966.1 NADP-specific glutamate dehydrogenase [Salmonella enterica subsp. enterica serovar Infantis] Overall Protective Antigen Prediction = **0.5664** (Probable **ANTIGEN**).

>EHO9884967.1 YnjH family protein [Salmonella enterica subsp. enterica serovar Infantis] Overall Protective Antigen Prediction = **0.6679** (Probable **ANTIGEN**).

>EHO9884968.1 pyrimidine (deoxy)nucleoside triphosphate diphosphatase [Salmonella enterica subsp. enterica serovar Infantis] Overall Protective Antigen Prediction = **0.5036** (Probable **ANTIGEN**).

>EHO9884969.1 exodeoxyribonuclease III [Salmonella enterica subsp. enterica serovar Infantis] Overall Protective Antigen Prediction = **0.3977** (Probable **NON-ANTIGEN**).

>EHO9884970.1 aspartate aminotransferase family protein [Salmonella enterica subsp. enterica serovar Infantis] Overall Protective Antigen Prediction = **0.5027** (Probable **ANTIGEN**).

>EHO9884971.1 arginine N-succinyltransferase [Salmonella enterica subsp. enterica serovar Infantis] Overall Protective Antigen Prediction = **0.4906** (Probable **ANTIGEN**).

>EHO9884972.1 succinylglutamate-semialdehyde dehydrogenase [Salmonella enterica subsp. enterica serovar Infantis] Overall Protective Antigen Prediction = **0.3787** (Probable **NON-ANTIGEN**).

>EHO9884973.1 N-succinylarginine dihydrolase [Salmonella enterica subsp. enterica serovar Infantis] Overall Protective Antigen Prediction = **0.3664** (Probable **NON-ANTIGEN**).

>EHO9884974.1 succinylglutamate desuccinylase [Salmonella enterica subsp. enterica serovar Infantis] Overall Protective Antigen Prediction = **0.4941** (Probable **ANTIGEN**).

>EHO9884975.1 ATP-independent periplasmic protein-refolding chaperone [Salmonella enterica subsp. enterica serovar Infantis] Overall Protective Antigen Prediction = **0.3973** (Probable **NON-ANTIGEN**).

>EHO9884976.1 excinuclease Cho [Salmonella enterica subsp. enterica serovar Infantis] Overall Protective Antigen Prediction = **0.3911** (Probable **NON-ANTIGEN**).

>EHO9884977.1 ammonia-dependent NAD(+) synthetase [Salmonella enterica subsp. enterica serovar Infantis] Overall Protective Antigen Prediction = **0.5574** (Probable **ANTIGEN**).

>EHO9884978.1 osmotically-inducible lipoprotein OsmE [Salmonella enterica subsp. enterica serovar

Infantis] Overall Protective Antigen Prediction = **0.3890** (Probable **NON-ANTIGEN**).

>EHO9884979.1 PTS N,N'-diacetylchitobiose transporter subunit IIB [Salmonella enterica subsp. enterica serovar Infantis] Overall Protective Antigen Prediction = **0.4343** (Probable **ANTIGEN**).

>EHO9884980.1 PTS N,N'-diacetylchitobiose transporter subunit IIC [Salmonella enterica subsp. enterica serovar Infantis] Overall Protective Antigen Prediction = **0.5640** (Probable **ANTIGEN**).

>EHO9884981.1 PTS N,N'-diacetylchitobiose transporter subunit IIA [Salmonella enterica subsp. enterica serovar Infantis] Overall Protective Antigen Prediction = **0.4003** (Probable **ANTIGEN**).

>EHO9884982.1 transcriptional regulator ChbR [Salmonella enterica subsp. enterica serovar Infantis] Overall Protective Antigen Prediction = **0.2960** (Probable **NON-ANTIGEN**).

>EHO9884983.1 6-phospho-beta-glucosidase [Salmonella enterica subsp. enterica serovar Infantis] Overall Protective Antigen Prediction = **0.4458** (Probable **ANTIGEN**).

>EHO9884984.1 chitin disaccharide deacetylase [Salmonella enterica subsp. enterica serovar Infantis] Overall Protective Antigen Prediction = **0.4219** (Probable **ANTIGEN**).

>EHO9884985.1 catalase HPII [Salmonella enterica subsp. enterica serovar Infantis] Overall Protective Antigen Prediction = **0.4543** (Probable **ANTIGEN**).

>EHO9884986.1 cell division activator CedA [Salmonella enterica subsp. enterica serovar Infantis] Overall Protective Antigen Prediction = **0.2755** (Probable **NON-ANTIGEN**).

>EHO9884987.1 cystine/sulfocysteine:cation symporter [Salmonella enterica subsp. enterica serovar Infantis] Overall Protective Antigen Prediction = **0.4695** (Probable **ANTIGEN**).

>EHO9884988.1 metal-dependent hydrolase [Salmonella enterica subsp. enterica serovar Infantis] Overall Protective Antigen Prediction = **0.4071** (Probable **ANTIGEN**).

>EHO9884989.1 hexitol phosphatase HxpB [Salmonella enterica subsp. enterica serovar Infantis] Overall Protective Antigen Prediction = **0.2845** (Probable **NON-ANTIGEN**).

>EHO9884990.1 YniB family protein [Salmonella enterica subsp. enterica serovar Infantis] Overall Protective Antigen Prediction = **0.8182** (Probable **ANTIGEN**).

>EHO9884991.1 fructosamine kinase family protein [Salmonella enterica subsp. enterica serovar Infantis] Overall Protective Antigen Prediction = **0.3727** (Probable **NON-ANTIGEN**).

>EHO9884992.1 type V toxin-antitoxin system endoribonuclease antitoxin GhoS [Salmonella enterica subsp. enterica serovar Infantis] Overall Protective Antigen Prediction = **0.3936** (Probable **NON-ANTIGEN**).

>EHO9884993.1 6-phosphofructokinase II [Salmonella enterica subsp. enterica serovar Infantis] Overall Protective Antigen Prediction = **0.5805** (Probable **ANTIGEN**).

>EHO9884994.1 YdiY family protein [Salmonella enterica subsp. enterica serovar Infantis] Overall Protective Antigen Prediction = **0.7168** (Probable **ANTIGEN**).

>EHO9884995.1 lipid A deacylase LpxR family protein [Salmonella enterica subsp. enterica serovar Infantis] Overall Protective Antigen Prediction = **0.5417** (Probable **ANTIGEN**).

>EHO9884996.1 hypothetical protein KND05_000276 [Salmonella enterica subsp. enterica serovar Infantis] Overall Protective Antigen Prediction = **0.6889** (Probable **ANTIGEN**).

>EHO9884997.1 DNA/RNA non-specific endonuclease [Salmonella enterica subsp. enterica serovar Infantis] Overall Protective Antigen Prediction = **0.3998** (Probable **NON-ANTIGEN**).

>EHO9884998.1 threonine--tRNA ligase [Salmonella enterica subsp. enterica serovar Infantis] Overall Protective Antigen Prediction = **0.4081** (Probable **ANTIGEN**).

>EHO9884999.1 translation initiation factor IF-3 [Salmonella enterica subsp. enterica serovar Infantis] Overall Protective Antigen Prediction = **0.6580** (Probable **ANTIGEN**).

>EHO9885000.1 50S ribosomal protein L35 [Salmonella enterica subsp. enterica serovar Infantis] Overall Protective Antigen Prediction = **0.7191** (Probable **ANTIGEN**).

>EHO9885001.1 50S ribosomal protein L20 [Salmonella enterica subsp. enterica serovar Infantis] Overall Protective Antigen Prediction = **0.3013** (Probable **NON-ANTIGEN**).

>EHO9885002.1 pheST operon leader peptide PheM [Salmonella enterica subsp. enterica serovar Infantis] Overall Protective Antigen Prediction = **1.8597** (Probable **ANTIGEN**).

>EHO9885003.1 phenylalanine--tRNA ligase subunit alpha [Salmonella enterica subsp. enterica serovar Infantis] Overall Protective Antigen Prediction = **0.4764** (Probable **ANTIGEN**).

>EHO9885004.1 phenylalanine--tRNA ligase subunit beta [Salmonella enterica subsp. enterica serovar Infantis] Overall Protective Antigen Prediction = **0.4732** (Probable **ANTIGEN**).

>EHO9885005.1 integration host factor subunit alpha [Salmonella enterica subsp. enterica serovar Infantis] Overall Protective Antigen Prediction = **0.6065** (Probable **ANTIGEN**).

>EHO9885006.1 vitamin B12 ABC transporter permease BtuC [Salmonella enterica subsp. enterica serovar Infantis] Overall Protective Antigen Prediction = **0.5072** (Probable **ANTIGEN**).

>EHO9885007.1 glutathione peroxidase [Salmonella enterica subsp. enterica serovar Infantis] Overall Protective Antigen Prediction = **0.4757** (Probable **ANTIGEN**).

>EHO9885008.1 vitamin B12 ABC transporter ATP-binding protein BtuD [Salmonella enterica subsp. enterica serovar Infantis] Overall Protective Antigen Prediction = **0.4437** (Probable **ANTIGEN**).

>EHO9885009.1 lipoprotein [Salmonella enterica subsp. enterica serovar Infantis] Overall Protective Antigen Prediction = **0.5818** (Probable **ANTIGEN**).

>EHO9885010.1 anti-FlhDC factor YdiV [Salmonella enterica subsp. enterica serovar Infantis] Overall Protective Antigen Prediction = **0.3928** (Probable **NON-ANTIGEN**).

>EHO9885011.1 YdiU family protein [Salmonella enterica subsp. enterica serovar Infantis] Overall Protective Antigen Prediction = **0.3846** (Probable **NON-ANTIGEN**).

>EHO9885012.1 hemin uptake protein HemP [Salmonella enterica subsp. enterica serovar Infantis] Overall Protective Antigen Prediction = **0.1832** (Probable **NON-ANTIGEN**).

>EHO9885013.1 3-deoxy-7-phosphoheptulonate synthase AroH [Salmonella enterica subsp. enterica serovar Infantis] Overall Protective Antigen Prediction = **0.4240** (Probable **ANTIGEN**).

>EHO9885014.1 posphoenolpyruvate synthetase regulatory kinase/phosphorylase PpsR [Salmonella enterica subsp. enterica serovar Infantis] Overall Protective Antigen Prediction = **0.4764** (Probable **ANTIGEN**).

>EHO9885015.1 phosphoenolpyruvate synthase [Salmonella enterica subsp. enterica serovar Infantis] Overall Protective Antigen Prediction = **0.4877** (Probable **ANTIGEN**).

>EHO9885016.1 medium-chain fatty-acid--CoA ligase [Salmonella enterica subsp. enterica serovar Infantis] Overall Protective Antigen Prediction = **0.4067** (Probable **ANTIGEN**).

>EHO9885017.1 ferredoxin family protein [Salmonella enterica subsp. enterica serovar Infantis] Overall Protective Antigen Prediction = **0.5454** (Probable **ANTIGEN**).

>EHO9885018.1 FAD-dependent oxidoreductase [Salmonella enterica subsp. enterica serovar Infantis] Overall Protective Antigen Prediction = **0.3970** (Probable **NON-ANTIGEN**).

>EHO9885019.1 electron transfer flavoprotein subunit alpha [Salmonella enterica subsp. enterica serovar Infantis] Overall Protective Antigen Prediction = **0.2758** (Probable **NON-ANTIGEN**).

>EHO9885020.1 electron transfer flavoprotein [Salmonella enterica subsp. enterica serovar Infantis] Overall Protective Antigen Prediction = **0.4338** (Probable **ANTIGEN**).

>EHO9885021.1 AraC family transcriptional regulator [Salmonella enterica subsp. enterica serovar Infantis] Overall Protective Antigen Prediction = **0.1939** (Probable **NON-ANTIGEN**).

>EHO9885022.1 acyl-CoA dehydrogenase [Salmonella enterica subsp. enterica serovar Infantis] Overall Protective Antigen Prediction = **0.3979** (Probable **NON-ANTIGEN**).

>EHO9885023.1 acyl CoA:acetate/3-ketoacid CoA transferase [Salmonella enterica subsp. enterica serovar Infantis] Overall Protective Antigen Prediction = **0.4097** (Probable **ANTIGEN**).

>EHO9885024.1 type I 3-dehydroquinate dehydratase [Salmonella enterica subsp. enterica serovar Infantis] Overall Protective Antigen Prediction = **0.4208** (Probable **ANTIGEN**).

>EHO9885025.1 quinate/shikimate dehydrogenase [Salmonella enterica subsp. enterica serovar Infantis] Overall Protective Antigen Prediction = **0.3989** (Probable **NON-ANTIGEN**).

>EHO9885026.1 MFS transporter [Salmonella enterica subsp. enterica serovar Infantis] Overall Protective Antigen Prediction = **0.5227** (Probable **ANTIGEN**).

>EHO9885027.1 MFS transporter [Salmonella enterica subsp. enterica serovar Infantis] Overall Protective Antigen Prediction = **0.5371** (Probable **ANTIGEN**).

>EHO9885028.1 YdiL family protein [Salmonella enterica subsp. enterica serovar Infantis] Overall Protective Antigen Prediction = **0.4593** (Probable **ANTIGEN**).

>EHO9885029.1 AI-2E family transporter YdiK [Salmonella enterica subsp. enterica serovar Infantis] Overall Protective Antigen Prediction = **0.4664** (Probable **ANTIGEN**).

>EHO9885030.1 FAD-binding oxidoreductase [Salmonella enterica subsp. enterica serovar Infantis] Overall Protective Antigen Prediction = **0.4437** (Probable **ANTIGEN**).

>EHO9885031.1 1,4-dihydroxy-2-naphthoyl-CoA hydrolase [Salmonella enterica subsp. enterica serovar Infantis] Overall Protective Antigen Prediction = **0.6196** (Probable **ANTIGEN**).

>EHO9885032.1 YdiH family protein [Salmonella enterica subsp. enterica serovar Infantis] Overall Protective Antigen Prediction = **0.5935** (Probable **ANTIGEN**).

>EHO9885033.1 L-cystine transporter [Salmonella enterica subsp. enterica serovar Infantis] Overall Protective Antigen Prediction = **0.6017** (Probable **ANTIGEN**).

>EHO9885034.1 Fe-S cluster assembly scaffold SufA [Salmonella enterica subsp. enterica serovar Infantis] Overall Protective Antigen Prediction = **0.6103** (Probable **ANTIGEN**).

>EHO9885035.1 Fe-S cluster assembly protein SufB [Salmonella enterica subsp. enterica serovar Infantis] Overall Protective Antigen Prediction = **0.4043** (Probable **ANTIGEN**).

>EHO9885036.1 Fe-S cluster assembly ATPase SufC [Salmonella enterica subsp. enterica serovar Infantis] Overall Protective Antigen Prediction = **0.6042** (Probable **ANTIGEN**).

>EHO9885037.1 Fe-S cluster assembly protein SufD [Salmonella enterica subsp. enterica serovar Infantis] Overall Protective Antigen Prediction = **0.5096** (Probable **ANTIGEN**).

>EHO9885038.1 cysteine desulfurase SufS [Salmonella enterica subsp. enterica serovar Infantis] Overall Protective Antigen Prediction = **0.3584** (Probable **NON-ANTIGEN**).

>EHO9885039.1 cysteine desulfuration protein SufE [Salmonella enterica subsp. enterica serovar Infantis] Overall Protective Antigen Prediction = **0.3054** (Probable **NON-ANTIGEN**).

>EHO9885040.1 L,D-transpeptidase family protein [Salmonella enterica subsp. enterica serovar Infantis] Overall Protective Antigen Prediction = **0.6057** (Probable **ANTIGEN**).

>EHO9885041.1 major outer membrane lipoprotein [Salmonella enterica subsp. enterica serovar Infantis] Overall Protective Antigen Prediction = **0.7340** (Probable **ANTIGEN**).

>EHO9885042.1 murein lipoprotein Lpp [Salmonella enterica subsp. enterica serovar Infantis] Overall Protective Antigen Prediction = **0.7819** (Probable **ANTIGEN**).

>EHO9885043.1 pyruvate kinase PykF [Salmonella enterica subsp. enterica serovar Infantis] Overall Protective Antigen Prediction = **0.5723** (Probable **ANTIGEN**).

>EHO9885044.1 APC family permease [Salmonella enterica subsp. enterica serovar Infantis] Overall Protective Antigen Prediction = **0.6314** (Probable **ANTIGEN**).

>EHO9885045.1 proline iminopeptidase-family hydrolase [Salmonella enterica subsp. enterica serovar Infantis] Overall Protective Antigen Prediction = **0.2818** (Probable **NON-ANTIGEN**).

>EHO9885046.1 cytoplasmic protein [Salmonella enterica subsp. enterica serovar Infantis] Overall Protective Antigen Prediction = **0.3398** (Probable **NON-ANTIGEN**).

>EHO9885047.1 DeoR family transcriptional regulator [Salmonella enterica subsp. enterica serovar Infantis] Overall Protective Antigen Prediction = **0.3589** (Probable **NON-ANTIGEN**).

>EHO9885048.1 tetrathionate reductase subunit TtrA [Salmonella enterica subsp. enterica serovar Infantis] Overall Protective Antigen Prediction = **0.4657** (Probable **ANTIGEN**).

>EHO9885049.1 tetrathionate reductase subunit TtrC [Salmonella enterica subsp. enterica serovar Infantis] Overall Protective Antigen Prediction = **0.5245** (Probable **ANTIGEN**).

>EHO9885050.1 tetrathionate reductase subunit TtrB [Salmonella enterica subsp. enterica serovar Infantis] Overall Protective Antigen Prediction = **0.4041** (Probable **ANTIGEN**).

>EHO9885051.1 two-component system sensor histidine kinase TtrS [Salmonella enterica subsp. enterica serovar Infantis] Overall Protective Antigen Prediction = **0.4973** (Probable **ANTIGEN**).

>EHO9885052.1 two-component system response regulator TtrR [Salmonella enterica subsp. enterica serovar Infantis] Overall Protective Antigen Prediction = **0.3090** (Probable **NON-ANTIGEN**).

>EHO9885053.1 fumarate hydratase FumD [Salmonella enterica subsp. enterica serovar Infantis] Overall Protective Antigen Prediction = **0.1413** (Probable **NON-ANTIGEN**).

>EHO9885054.1 DUF523 and DUF1722 domain-containing protein [Salmonella enterica subsp. enterica serovar Infantis] Overall Protective Antigen Prediction = **0.5116** (Probable **ANTIGEN**).

>EHO9885055.1 MerR family transcriptional regulator [Salmonella enterica subsp. enterica serovar Infantis] Overall Protective Antigen Prediction = **0.2094** (Probable **NON-ANTIGEN**).

>EHO9885056.1 two component system response regulator [Salmonella enterica subsp. enterica serovar Infantis] Overall Protective Antigen Prediction = **0.4053** (Probable **ANTIGEN**).

>EHO9885057.1 two component system sensor kinase [Salmonella enterica subsp. enterica serovar Infantis] Overall Protective Antigen Prediction = **0.4442** (Probable **ANTIGEN**).

>EHO9885058.1 SPI-2 type III secretion system protein SpiC [Salmonella enterica subsp. enterica serovar Infantis] Overall Protective Antigen Prediction = **0.3317** (Probable **NON-ANTIGEN**).

>EHO9885059.1 SPI-2 type III secretion system protein SpiA [Salmonella enterica subsp. enterica serovar Infantis] Overall Protective Antigen Prediction = **0.4868** (Probable **ANTIGEN**).

>EHO9885060.1 SctD family type III secretion system inner membrane ring subunit SsaD [Salmonella enterica subsp. enterica serovar Infantis] Overall Protective Antigen Prediction = **0.4334** (Probable **ANTIGEN**).

>EHO9885061.1 YscE family type III secretion system co-chaperone SsaE [Salmonella enterica subsp. enterica serovar Infantis] Overall Protective Antigen Prediction = **0.2636** (Probable **NON-ANTIGEN**).

>EHO9885062.1 SPI-2 type III secretion system chaperone SseA [Salmonella enterica subsp. enterica serovar Infantis] Overall Protective Antigen Prediction = **0.5432** (Probable **ANTIGEN**).

>EHO9885063.1 SPI-2 type III secretion system translocon protein SseB [Salmonella enterica subsp. enterica serovar Infantis] Overall Protective Antigen Prediction = **0.5151** (Probable **ANTIGEN**).

>EHO9885064.1 SycD/LcrH family type III secretion system chaperone SscA [Salmonella enterica subsp. enterica serovar Infantis] Overall Protective Antigen Prediction = **0.3940** (Probable **NON-ANTIGEN**).

>EHO9885065.1 SPI-2 type III secretion system translocon protein SseC [Salmonella enterica subsp. enterica serovar Infantis] Overall Protective Antigen Prediction = **0.4557** (Probable **ANTIGEN**).

>EHO9885066.1 SPI-2 type III secretion system translocon protein SseD [Salmonella enterica subsp. enterica serovar Infantis] Overall Protective Antigen Prediction = **0.5413** (Probable **ANTIGEN**).

>EHO9885067.1 LcrR family type III secretion system chaperone SseE [Salmonella enterica subsp. enterica serovar Infantis] Overall Protective Antigen Prediction = **0.1399** (Probable **NON-ANTIGEN**).

>EHO9885068.1 SycD/LcrH family type III secretion system chaperone SscB [Salmonella enterica subsp. enterica serovar Infantis] Overall Protective Antigen Prediction = **0.4174** (Probable **ANTIGEN**).

>EHO9885069.1 type III secretion systems effector SseF [Salmonella enterica subsp. enterica serovar Infantis] Overall Protective Antigen Prediction = **0.5212** (Probable **ANTIGEN**).

>EHO9885070.1 pathogenicity island 2 effector protein SseG [Salmonella enterica subsp. enterica serovar Infantis] Overall Protective Antigen Prediction = **0.3572** (Probable **NON-ANTIGEN**).

>EHO9885071.1 type III secretion system needle filament protein SsaG [Salmonella enterica subsp. enterica serovar Infantis] Overall Protective Antigen Prediction = **0.0434** (Probable **NON-ANTIGEN**).

>EHO9885072.1 EscG/YscG/SsaH family type III secretion system needle protein co-chaperone [Salmonella enterica subsp. enterica serovar Infantis] Overall Protective Antigen Prediction = **0.3371** (Probable **NON-ANTIGEN**).

>EHO9885073.1 SctI family type III secretion system inner rod subunit SsaI [Salmonella enterica subsp. enterica serovar Infantis] Overall Protective Antigen Prediction = **0.3728** (Probable **NON-ANTIGEN**).

>EHO9885074.1 SPI-2 type III secretion system apparatus lipoprotein SsaJ [Salmonella enterica subsp. enterica serovar Infantis] Overall Protective Antigen Prediction = **0.5434** (Probable **ANTIGEN**).

>EHO9885075.1 cytoplasmic protein [Salmonella enterica subsp. enterica serovar Infantis] Overall Protective Antigen Prediction = **0.3809** (Probable **NON-ANTIGEN**).

>EHO9885076.1 SPI-2 type III secretion system apparatus protein SsaK [Salmonella enterica subsp. enterica serovar Infantis] Overall Protective Antigen Prediction = **0.5162** (Probable **ANTIGEN**).

>EHO9885077.1 SPI-2 type III secretion system apparatus protein SsaL [Salmonella enterica subsp. enterica serovar Infantis] Overall Protective Antigen Prediction = **0.4322** (Probable **ANTIGEN**).

>EHO9885078.1 SPI-2 type III secretion system apparatus protein SsaM [Salmonella enterica subsp. enterica serovar Infantis] Overall Protective Antigen Prediction = **0.4379** (Probable **ANTIGEN**).

>EHO9885079.1 SPI-2 type III secretion system apparatus protein SsaV [Salmonella enterica subsp. enterica serovar Infantis] Overall Protective Antigen Prediction = **0.4048** (Probable **ANTIGEN**).

>EHO9885080.1 SctN family type III secretion system ATPase SsaN [Salmonella enterica subsp. enterica serovar Infantis] Overall Protective Antigen Prediction = **0.2693** (Probable **NON-ANTIGEN**).

>EHO9885081.1 SPI-2 type III secretion system apparatus protein SsaO [Salmonella enterica subsp. enterica serovar Infantis] Overall Protective Antigen Prediction = **0.1267** (Probable **NON-ANTIGEN**).

>EHO9885082.1 SPI-2 type III secretion system apparatus protein SsaP [Salmonella enterica subsp. enterica serovar Infantis] Overall Protective Antigen Prediction = **0.5027** (Probable **ANTIGEN**).

>EHO9885083.1 SPI-2 type III secretion system cytoplasmic ring protein SsaQ [Salmonella enterica subsp. enterica serovar Infantis] Overall Protective Antigen Prediction = **0.3850** (Probable **NON-ANTIGEN**).

>EHO9885084.1 SPI-2 type III secretion system export apparatus protein SsaR [Salmonella enterica subsp. enterica serovar Infantis] Overall Protective Antigen Prediction = **0.7004** (Probable **ANTIGEN**).

>EHO9885085.1 SPI-2 type III secretion system apparatus protein SsaS [Salmonella enterica subsp. enterica serovar Infantis] Overall Protective Antigen Prediction = **0.3580** (Probable **NON-ANTIGEN**).

>EHO9885086.1 SPI-2 type III secretion system apparatus protein SsaT [Salmonella enterica subsp. enterica serovar Infantis] Overall Protective Antigen Prediction = **0.5041** (Probable **ANTIGEN**).

>EHO9885087.1 SPI-2 type III secretion system apparatus protein SsaU [Salmonella enterica subsp. enterica serovar Infantis] Overall Protective Antigen Prediction = **0.5213** (Probable **ANTIGEN**).

>EHO9885088.1 multidrug efflux MATE transporter MdtK [Salmonella enterica subsp. enterica serovar Infantis] Overall Protective Antigen Prediction = **0.5074** (Probable **ANTIGEN**).

>EHO9885089.1 riboflavin synthase [Salmonella enterica subsp. enterica serovar Infantis] Overall Protective Antigen Prediction = **0.5504** (Probable **ANTIGEN**).

>EHO9885090.1 cyclopropane fatty acyl phospholipid synthase [Salmonella enterica subsp. enterica serovar Infantis] Overall Protective Antigen Prediction = **0.4192** (Probable **ANTIGEN**).

>EHO9885091.1 Bcr/CflA family multidrug efflux MFS transporter [Salmonella enterica subsp. enterica serovar Infantis] Overall Protective Antigen Prediction = **0.5298** (Probable **ANTIGEN**).

>EHO9885092.1 LysR family transcriptional regulator [Salmonella enterica subsp. enterica serovar Infantis] Overall Protective Antigen Prediction = **0.1790** (Probable **NON-ANTIGEN**).

>EHO9885093.1 HTH-type transcriptional repressor PurR [Salmonella enterica subsp. enterica serovar Infantis] Overall Protective Antigen Prediction = **0.1984** (Probable **NON-ANTIGEN**).

>EHO9885094.1 YnhF family membrane protein [Salmonella enterica subsp. enterica serovar Infantis] Overall Protective Antigen Prediction = **0.4275** (Probable **ANTIGEN**).

>EHO9885095.1 superoxide dismutase [Fe] [Salmonella enterica subsp. enterica serovar Infantis] Overall Protective Antigen Prediction = **0.5059** (Probable **ANTIGEN**).

>EHO9885096.1 C40 family peptidase [Salmonella enterica subsp. enterica serovar Infantis] Overall Protective Antigen Prediction = **0.6969** (Probable **ANTIGEN**).

>EHO9885097.1 monothiol glutaredoxin 4 [Salmonella enterica subsp. enterica serovar Infantis] Overall Protective Antigen Prediction = **0.5514** (Probable **ANTIGEN**).

>EHO9885098.1 ribonuclease T [Salmonella enterica subsp. enterica serovar Infantis] Overall Protective Antigen Prediction = **0.4154** (Probable **ANTIGEN**).

>EHO9885099.1 lactoylglutathione lyase [Salmonella enterica subsp. enterica serovar Infantis] Overall Protective Antigen Prediction = **0.6293** (Probable **ANTIGEN**).

>EHO9885100.1 alkene reductase [Salmonella enterica subsp. enterica serovar Infantis] Overall Protective Antigen Prediction = **0.4154** (Probable **ANTIGEN**).

>EHO9885101.1 TetR/AcrR family transcriptional regulator [Salmonella enterica subsp. enterica serovar Infantis] Overall Protective Antigen Prediction = **0.2542** (Probable **NON-ANTIGEN**).

>EHO9885102.1 DUF1289 domain-containing protein [Salmonella enterica subsp. enterica serovar Infantis] Overall Protective Antigen Prediction = **0.3690** (Probable **NON-ANTIGEN**).

>EHO9885103.1 aldo/keto reductase family oxidoreductase [Salmonella enterica subsp. enterica serovar Infantis] Overall Protective Antigen Prediction = **0.4089** (Probable **ANTIGEN**).

>EHO9885104.1 superoxide dismutase [Cu-Zn] SodC2 [Salmonella enterica subsp. enterica serovar Infantis] Overall Protective Antigen Prediction = **0.8314** (Probable **ANTIGEN**).

>EHO9885105.1 FUSC family protein [Salmonella enterica subsp. enterica serovar Infantis] Overall Protective Antigen Prediction = **0.3983** (Probable **NON-ANTIGEN**).

>EHO9885106.1 HlyD family secretion protein [Salmonella enterica subsp. enterica serovar Infantis] Overall Protective Antigen Prediction = **0.7025** (Probable **ANTIGEN**).

>EHO9885107.1 DUF1656 domain-containing protein [Salmonella enterica subsp. enterica serovar Infantis] Overall Protective Antigen Prediction = **0.6743** (Probable **ANTIGEN**).

>EHO9885108.1 transcriptional regulator SlyA [Salmonella enterica subsp. enterica serovar Infantis] Overall Protective Antigen Prediction = **0.3232** (Probable **NON-ANTIGEN**).

>EHO9885109.1 outer membrane lipoprotein SlyB [Salmonella enterica subsp. enterica serovar Infantis] Overall Protective Antigen Prediction = **0.9263** (Probable **ANTIGEN**).

>EHO9885110.1 anhydro-N-acetylmuramic acid kinase [Salmonella enterica subsp. enterica serovar Infantis] Overall Protective Antigen Prediction = **0.4048** (Probable **ANTIGEN**).

>EHO9885111.1 C-type lysozyme inhibitor [Salmonella enterica subsp. enterica serovar Infantis] Overall Protective Antigen Prediction = **0.4233** (Probable **ANTIGEN**).

>EHO9885112.1 pyridoxamine 5'-phosphate oxidase [Salmonella enterica subsp. enterica serovar Infantis] Overall Protective Antigen Prediction = **0.4084** (Probable **ANTIGEN**).

>EHO9885113.1 tyrosine--tRNA ligase [Salmonella enterica subsp. enterica serovar Infantis] Overall Protective Antigen Prediction = **0.4093** (Probable **ANTIGEN**).

>EHO9885114.1 pyridoxal kinase PdxY [Salmonella enterica subsp. enterica serovar Infantis] Overall Protective Antigen Prediction = **0.1993** (Probable **NON-ANTIGEN**).

>EHO9885115.1 glutathione transferase GstA [Salmonella enterica subsp. enterica serovar Infantis] Overall Protective Antigen Prediction = **0.3707** (Probable **NON-ANTIGEN**).

>EHO9885116.1 dipeptide/tripeptide permease DtpA [Salmonella enterica subsp. enterica serovar Infantis] Overall Protective Antigen Prediction = **0.3843** (Probable **NON-ANTIGEN**).

>EHO9885117.1 endonuclease III [Salmonella enterica subsp. enterica serovar Infantis] Overall Protective Antigen Prediction = **0.4560** (Probable **ANTIGEN**).

>EHO9885118.1 electron transport complex subunit E [Salmonella enterica subsp. enterica serovar Infantis] Overall Protective Antigen Prediction = **0.3907** (Probable **NON-ANTIGEN**).

>EHO9885119.1 electron transport complex subunit RsxG [Salmonella enterica subsp. enterica serovar Infantis] Overall Protective Antigen Prediction = **0.4948** (Probable **ANTIGEN**).

>EHO9885120.1 electron transport complex subunit RsxD [Salmonella enterica subsp. enterica serovar Infantis] Overall Protective Antigen Prediction = **0.5042** (Probable **ANTIGEN**).

>EHO9885121.1 electron transport complex subunit RsxC [Salmonella enterica subsp. enterica serovar Infantis] Overall Protective Antigen Prediction = **0.5569** (Probable **ANTIGEN**).

>EHO9885122.1 electron transport complex subunit RsxB [Salmonella enterica subsp. enterica serovar Infantis] Overall Protective Antigen Prediction = **0.6523** (Probable **ANTIGEN**).

>EHO9885123.1 electron transport complex subunit RsxA [Salmonella enterica subsp. enterica serovar Infantis] Overall Protective Antigen Prediction = **0.4722** (Probable **ANTIGEN**).

>EHO9885124.1 DUF2569 domain-containing protein [Salmonella enterica subsp. enterica serovar Infantis] Overall Protective Antigen Prediction = **0.5060** (Probable **ANTIGEN**).

>EHO9885125.1 transcription modulator YdgT [Salmonella enterica subsp. enterica serovar Infantis] Overall Protective Antigen Prediction = **0.2008** (Probable **NON-ANTIGEN**).

>EHO9885126.1 division septum protein Blr [Salmonella enterica subsp. enterica serovar Infantis] Overall Protective Antigen Prediction = **0.2703** (Probable **NON-ANTIGEN**).

>EHO9885127.1 oxidoreductase [Salmonella enterica subsp. enterica serovar Infantis] Overall Protective Antigen Prediction = **0.3911** (Probable **NON-ANTIGEN**).

>EHO9885128.1 adenosine deaminase [Salmonella enterica subsp. enterica serovar Infantis] Overall Protective Antigen Prediction = **0.4487** (Probable **ANTIGEN**).

>EHO9885129.1 pyridoxal phosphate-dependent aminotransferase [Salmonella enterica subsp. enterica serovar Infantis] Overall Protective Antigen Prediction = **0.4404** (Probable **ANTIGEN**).

>EHO9885130.1 PTS maltose transporter subunit IICB [Salmonella enterica subsp. enterica serovar Infantis] Overall Protective Antigen Prediction = **0.5098** (Probable **ANTIGEN**).

>EHO9885131.1 Mal regulon transcriptional regulator MalI [Salmonella enterica subsp. enterica serovar Infantis] Overall Protective Antigen Prediction = **0.5288** (Probable **ANTIGEN**).

>EHO9885132.1 YdgA family protein [Salmonella enterica subsp. enterica serovar Infantis] Overall Protective Antigen Prediction = **0.5523** (Probable **ANTIGEN**).

>EHO9885133.1 mannose-6-phosphate isomerase [Salmonella enterica subsp. enterica serovar Infantis] Overall Protective Antigen Prediction = **0.5023** (Probable **ANTIGEN**).

>EHO9885134.1 class I fumarate hydratase [Salmonella enterica subsp. enterica serovar Infantis] Overall Protective Antigen Prediction = **0.4850** (Probable **ANTIGEN**).

>EHO9885135.1 class II fumarate hydratase [Salmonella enterica subsp. enterica serovar Infantis] Overall Protective Antigen Prediction = **0.4125** (Probable **ANTIGEN**).

>EHO9885136.1 DNA replication terminus site-binding protein [Salmonella enterica subsp. enterica serovar Infantis] Overall Protective Antigen Prediction = **0.3798** (Probable **NON-ANTIGEN**).

>EHO9885137.1 two-component system sensor histidine kinase RstB [Salmonella enterica subsp. enterica serovar Infantis] Overall Protective Antigen Prediction = **0.6688** (Probable **ANTIGEN**).

>EHO9885138.1 amidohydrolase [Salmonella enterica subsp. enterica serovar Infantis] Overall Protective Antigen Prediction = **0.5582** (Probable **ANTIGEN**).

>EHO9885139.1 porin OmpC [Salmonella enterica subsp. enterica serovar Infantis] Overall Protective Antigen Prediction = **0.7601** (Probable **ANTIGEN**).

>EHO9885140.1 two-component system response regulator RstA [Salmonella enterica subsp. enterica serovar Infantis] Overall Protective Antigen Prediction = **0.4494** (Probable **ANTIGEN**).

>EHO9885141.1 GlpM family protein [Salmonella enterica subsp. enterica serovar Infantis] Overall Protective Antigen Prediction = **0.6945** (Probable **ANTIGEN**).

>EHO9885142.1 amino acid permease [Salmonella enterica subsp. enterica serovar Infantis] Overall Protective Antigen Prediction = **0.4506** (Probable **ANTIGEN**).

>EHO9885143.1 DUF1471 domain-containing protein [Salmonella enterica subsp. enterica serovar Infantis] Overall Protective Antigen Prediction = **0.5868** (Probable **ANTIGEN**).

>EHO9885144.1 Re/Si-specific NAD(P)(+) transhydrogenase subunit alpha [Salmonella enterica subsp. enterica serovar Infantis] Overall Protective Antigen Prediction = **0.3508** (Probable **NON-ANTIGEN**).

>EHO9885145.1 Re/Si-specific NAD(P)(+) transhydrogenase subunit beta [Salmonella enterica subsp. enterica serovar Infantis] Overall Protective Antigen Prediction = **0.4848** (Probable **ANTIGEN**).

>EHO9885146.1 AI-2E family transporter [Salmonella enterica subsp. enterica serovar Infantis] Overall Protective Antigen Prediction = **0.5044** (Probable **ANTIGEN**).

>EHO9885147.1 multidrug/spermidine efflux SMR transporter subunit MdtJ [Salmonella enterica subsp. enterica serovar Infantis] Overall Protective Antigen Prediction = **0.6271** (Probable **ANTIGEN**).

>EHO9885148.1 multidrug/spermidine efflux SMR transporter subunit MdtI [Salmonella enterica subsp. enterica serovar Infantis] Overall Protective Antigen Prediction = **0.3518** (Probable **NON-ANTIGEN**).

>EHO9885149.1 serine protease [Salmonella enterica subsp. enterica serovar Infantis] Overall Protective Antigen Prediction = **0.5138** (Probable **ANTIGEN**).

>EHO9885150.1 acid shock protein [Salmonella enterica subsp. enterica serovar Infantis] Overall Protective Antigen Prediction = **1.0564** (Probable **ANTIGEN**).

>EHO9885151.1 MFS transporter [Salmonella enterica subsp. enterica serovar Infantis] Overall Protective Antigen Prediction = **0.4728** (Probable **ANTIGEN**).

>EHO9885152.1 LysR family transcriptional regulator [Salmonella enterica subsp. enterica serovar Infantis] Overall Protective Antigen Prediction = **0.3750** (Probable **NON-ANTIGEN**).

>EHO9885153.1 ROK family transcriptional regulator [Salmonella enterica subsp. enterica serovar Infantis] Overall Protective Antigen Prediction = **0.3167** (Probable **NON-ANTIGEN**).

>EHO9885154.1 ATP-dependent dethiobiotin synthetase BioD [Salmonella enterica subsp. enterica serovar Infantis] Overall Protective Antigen Prediction = **0.3725** (Probable **NON-ANTIGEN**).

>EHO9885155.1 voltage-gated ClC-type chloride channel ClcB [Salmonella enterica subsp. enterica serovar Infantis] Overall Protective Antigen Prediction = **0.3526** (Probable **NON-ANTIGEN**).

>EHO9885156.1 osmoprotectant ABC transporter ATP-binding protein OsmV [Salmonella enterica subsp. enterica serovar Infantis] Overall Protective Antigen Prediction = **0.3270** (Probable **NON-ANTIGEN**).

>EHO9885157.1 osmoprotectant ABC transporter permease OsmW [Salmonella enterica subsp. enterica serovar Infantis] Overall Protective Antigen Prediction = **0.5161** (Probable **ANTIGEN**).

>EHO9885158.1 osmoprotectant ABC transporter substrate-binding protein OsmX [Salmonella enterica subsp. enterica serovar Infantis] Overall Protective Antigen Prediction = **0.4755** (Probable **ANTIGEN**).

>EHO9885159.1 osmoprotectant ABC transporter permease OsmY [Salmonella enterica subsp. enterica serovar Infantis] Overall Protective Antigen Prediction = **0.4468** (Probable **ANTIGEN**).

>EHO9885160.1 Tat proofreading chaperone DmsD [Salmonella enterica subsp. enterica serovar Infantis] Overall Protective Antigen Prediction = **0.2290** (Probable **NON-ANTIGEN**).

>EHO9885161.1 dimethyl sulfoxide reductase anchor subunit family protein, partial [Salmonella enterica subsp. enterica serovar Infantis] Overall Protective Antigen Prediction = **0.3817** (Probable **NON-ANTIGEN**).

>EHO9885162.1 carbon storage regulator CsrA [Salmonella enterica subsp. enterica serovar Infantis] Overall Protective Antigen Prediction = **0.6601** (Probable **ANTIGEN**).

>EHO9885163.1 alanine--tRNA ligase [Salmonella enterica subsp. enterica serovar Infantis] Overall Protective Antigen Prediction = **0.4971** (Probable **ANTIGEN**).

>EHO9885164.1 recombination regulator RecX [Salmonella enterica subsp. enterica serovar Infantis] Overall Protective Antigen Prediction = **0.3129** (Probable **NON-ANTIGEN**).

>EHO9885165.1 recombinase RecA [Salmonella enterica subsp. enterica serovar Infantis] Overall Protective Antigen Prediction = **0.6150** (Probable **ANTIGEN**).

>EHO9885166.1 nicotinamide-nucleotide amidase [Salmonella enterica subsp. enterica serovar Infantis] Overall Protective Antigen Prediction = **0.5734** (Probable **ANTIGEN**).

>EHO9885167.1 lytic murein transglycosylase B [Salmonella enterica subsp. enterica serovar Infantis] Overall Protective Antigen Prediction = **0.3020** (Probable **NON-ANTIGEN**).

>EHO9885168.1 PTS glucitol/sorbitol transporter subunit IIC [Salmonella enterica subsp. enterica serovar Infantis] Overall Protective Antigen Prediction = **0.1348** (Probable **NON-ANTIGEN**).

>EHO9885169.1 PTS glucitol/sorbitol transporter subunit IIB [Salmonella enterica subsp. enterica serovar Infantis] Overall Protective Antigen Prediction = **0.4359** (Probable **ANTIGEN**).

>EHO9885170.1 PTS glucitol/sorbitol transporter subunit IIA [Salmonella enterica subsp. enterica serovar Infantis] Overall Protective Antigen Prediction = **0.4012** (Probable **ANTIGEN**).

>EHO9885171.1 sorbitol-6-phosphate dehydrogenase [Salmonella enterica subsp. enterica serovar Infantis] Overall Protective Antigen Prediction = **0.6057** (Probable **ANTIGEN**).

>EHO9885172.1 transcriptional regulator GutM [Salmonella enterica subsp. enterica serovar Infantis] Overall Protective Antigen Prediction = **0.5107** (Probable **ANTIGEN**).

>EHO9885173.1 glucitol operon DNA-binding transcriptional repressor SrlR [Salmonella enterica subsp. enterica serovar Infantis] Overall Protective Antigen Prediction = **0.2084** (Probable **NON-ANTIGEN**).

>EHO9885174.1 arabinose-5-phosphate isomerase GutQ [Salmonella enterica subsp. enterica serovar Infantis] Overall Protective Antigen Prediction = **0.3053** (Probable **NON-ANTIGEN**).

>EHO9885175.1 nitric oxide reductase transcriptional regulator NorR [Salmonella enterica subsp. enterica serovar Infantis] Overall Protective Antigen Prediction = **0.3584** (Probable **NON-ANTIGEN**).

>EHO9885176.1 anaerobic nitric oxide reductase flavorubredoxin [Salmonella enterica subsp. enterica serovar Infantis] Overall Protective Antigen Prediction = **0.4195** (Probable **ANTIGEN**).

>EHO9885177.1 NADH:flavorubredoxin reductase NorW [Salmonella enterica subsp. enterica serovar Infantis] Overall Protective Antigen Prediction = **0.5609** (Probable **ANTIGEN**).

>EHO9885178.1 carbamoyltransferase HypF [Salmonella enterica subsp. enterica serovar Infantis] Overall Protective Antigen Prediction = **0.4712** (Probable **ANTIGEN**).

>EHO9885179.1 electron transport protein HydN [Salmonella enterica subsp. enterica serovar Infantis] Overall Protective Antigen Prediction = **0.4055** (Probable **ANTIGEN**).

>EHO9885180.1 hypothetical protein KND05_000465 [Salmonella enterica subsp. enterica serovar Infantis] Overall Protective Antigen Prediction = **0.4444** (Probable **ANTIGEN**).

>EHO9885181.1 hydrogenase maturation peptidase HycI [Salmonella enterica subsp. enterica serovar Infantis] Overall Protective Antigen Prediction = **0.2429** (Probable **NON-ANTIGEN**).

>EHO9885182.1 formate hydrogenlyase maturation HycH family protein [Salmonella enterica subsp. enterica serovar Infantis] Overall Protective Antigen Prediction = **0.2420** (Probable **NON-ANTIGEN**).

>EHO9885183.1 formate hydrogenlyase subunit HycG [Salmonella enterica subsp. enterica serovar Infantis] Overall Protective Antigen Prediction = **0.5560** (Probable **ANTIGEN**).

>EHO9885184.1 formate hydrogenlyase subunit HycF [Salmonella enterica subsp. enterica serovar Infantis] Overall Protective Antigen Prediction = **0.4427** (Probable **ANTIGEN**).

>EHO9885185.1 formate hydrogenlyase subunit HycE [Salmonella enterica subsp. enterica serovar Infantis] Overall Protective Antigen Prediction = **0.3926** (Probable **NON-ANTIGEN**).

>EHO9885186.1 respiratory chain complex I subunit 1 family protein [Salmonella enterica subsp. enterica serovar Infantis] Overall Protective Antigen Prediction = **0.3173** (Probable **NON-ANTIGEN**).

>EHO9885187.1 formate hydrogenlyase subunit 3 [Salmonella enterica subsp. enterica serovar Infantis] Overall Protective Antigen Prediction = **0.3770** (Probable **NON-ANTIGEN**).

>EHO9885188.1 formate hydrogenlyase subunit HycB [Salmonella enterica subsp. enterica serovar Infantis] Overall Protective Antigen Prediction = **0.4346** (Probable **ANTIGEN**).

>EHO9885189.1 formate hydrogenlyase regulator HycA [Salmonella enterica subsp. enterica serovar Infantis] Overall Protective Antigen Prediction = **0.6854** (Probable **ANTIGEN**).

>EHO9885190.1 hydrogenase maturation nickel metallochaperone HypA [Salmonella enterica subsp. enterica serovar Infantis] Overall Protective Antigen Prediction = **0.4472** (Probable **ANTIGEN**).

>EHO9885191.1 hydrogenase nickel incorporation protein HypB [Salmonella enterica subsp. enterica serovar Infantis] Overall Protective Antigen Prediction = **0.6327** (Probable **ANTIGEN**).

>EHO9885192.1 hydrogenase 3 maturation protein HypC [Salmonella enterica subsp. enterica serovar Infantis] Overall Protective Antigen Prediction = **0.6356** (Probable **ANTIGEN**).

>EHO9885193.1 hydrogenase formation protein HypD [Salmonella enterica subsp. enterica serovar Infantis] Overall Protective Antigen Prediction = **0.4335** (Probable **ANTIGEN**).

>EHO9885194.1 hydrogenase expression/formation protein HypE [Salmonella enterica subsp. enterica serovar Infantis] Overall Protective Antigen Prediction = **0.4968** (Probable **ANTIGEN**).

>EHO9885195.1 formate hydrogenlyase transcriptional activator FlhA [Salmonella enterica subsp. enterica serovar Infantis] Overall Protective Antigen Prediction = **0.3115** (Probable **NON-ANTIGEN**).

>EHO9885196.1 nitrous oxide-stimulated promoter family protein [Salmonella enterica subsp. enterica serovar Infantis] Overall Protective Antigen Prediction = **0.2704** (Probable **NON-ANTIGEN**).

>EHO9885197.1 iron/manganese ABC transporter substrate-binding protein SitA [Salmonella enterica subsp. enterica serovar Infantis] Overall Protective Antigen Prediction = **0.3858** (Probable **NON-ANTIGEN**).

>EHO9885198.1 iron/manganese ABC transporter ATP-binding protein SitB [Salmonella enterica subsp. enterica serovar Infantis] Overall Protective Antigen Prediction = **0.3438** (Probable **NON-ANTIGEN**).

>EHO9885199.1 iron/manganese ABC transporter permease subunit SitC [Salmonella enterica subsp. enterica serovar Infantis] Overall Protective Antigen Prediction = **0.4278** (Probable **ANTIGEN**).

>EHO9885200.1 iron/manganese ABC transporter permease subunit SitD [Salmonella enterica subsp. enterica serovar Infantis] Overall Protective Antigen Prediction = **0.5045** (Probable **ANTIGEN**).

>EHO9885201.1 type III secretion system YopJ family effector AvrA [Salmonella enterica subsp. enterica serovar Infantis] Overall Protective Antigen Prediction = **0.5772** (Probable **ANTIGEN**).

>EHO9885202.1 transcriptional regulator [Salmonella enterica subsp. enterica serovar Infantis] Overall Protective Antigen Prediction = **0.3483** (Probable **NON-ANTIGEN**).

>EHO9885203.1 transcriptional regulator SirC [Salmonella enterica subsp. enterica serovar Infantis] Overall Protective Antigen Prediction = **0.3468** (Probable **NON-ANTIGEN**).

>EHO9885204.1 type III secretion system effector protein OrgC [Salmonella enterica subsp. enterica serovar Infantis] Overall Protective Antigen Prediction = **0.3794** (Probable **NON-ANTIGEN**).

>EHO9885205.1 type III secretion system linker protein OrgB [Salmonella enterica subsp. enterica serovar Infantis] Overall Protective Antigen Prediction = **0.2583** (Probable **NON-ANTIGEN**).

>EHO9885206.1 oxygen-regulated invasion protein OrgA [Salmonella enterica subsp. enterica serovar Infantis] Overall Protective Antigen Prediction = **0.4963** (Probable **ANTIGEN**).

>EHO9885207.1 type III secretion system inner membrane ring lipoprotein PrgK [Salmonella enterica subsp. enterica serovar Infantis] Overall Protective Antigen Prediction = **0.5132** (Probable **ANTIGEN**).

>EHO9885208.1 type III secretion system inner rod protein PrgJ [Salmonella enterica subsp. enterica serovar Infantis] Overall Protective Antigen Prediction = **0.4951** (Probable **ANTIGEN**).

>EHO9885209.1 type III secretion system needle filament protein PrgI [Salmonella enterica subsp. enterica serovar Infantis] Overall Protective Antigen Prediction = **0.4637** (Probable **ANTIGEN**).

>EHO9885210.1 type III secretion system inner membrane ring protein PrgH [Salmonella enterica subsp. enterica serovar Infantis] Overall Protective Antigen Prediction = **0.5066** (Probable **ANTIGEN**).

>EHO9885211.1 transcriptional regulator HilD [Salmonella enterica subsp. enterica serovar Infantis] Overall Protective Antigen Prediction = **0.4007** (Probable **ANTIGEN**).

>EHO9885212.1 transcriptional regulator HilA [Salmonella enterica subsp. enterica serovar Infantis] Overall Protective Antigen Prediction = **0.3985** (Probable **NON-ANTIGEN**).

>EHO9885213.1 SPI-1 type III secretion system invasion protein IagB [Salmonella enterica subsp. enterica serovar Infantis] Overall Protective Antigen Prediction = **0.6265** (Probable **ANTIGEN**).

>EHO9885214.1 SPI-1 type III secretion system effector GTPase-activating protein SptP [Salmonella enterica subsp. enterica serovar Infantis] Overall Protective Antigen Prediction = **0.5160** (Probable **ANTIGEN**).

>EHO9885215.1 chaperone SicP [Salmonella enterica subsp. enterica serovar Infantis] Overall Protective Antigen Prediction = **0.0597** (Probable **NON-ANTIGEN**).

>EHO9885216.1 hypothetical protein KND05_000501 [Salmonella enterica subsp. enterica serovar Infantis] Overall Protective Antigen Prediction = **0.3499** (Probable **NON-ANTIGEN**).

>EHO9885217.1 acyl carrier protein [Salmonella enterica subsp. enterica serovar Infantis] Overall Protective Antigen Prediction = **0.5537** (Probable **ANTIGEN**).

>EHO9885218.1 SPI-1 type III secretion system effector SipA [Salmonella enterica subsp. enterica serovar Infantis] Overall Protective Antigen Prediction = **0.6524** (Probable **ANTIGEN**).

>EHO9885219.1 SPI-1 type III secretion system needle tip complex protein SipD [Salmonella enterica subsp. enterica serovar Infantis] Overall Protective Antigen Prediction = **0.4931** (Probable **ANTIGEN**).

>EHO9885220.1 SPI-1 type III secretion system needle tip complex protein SipC [Salmonella enterica subsp. enterica serovar Infantis] Overall Protective Antigen Prediction = **0.7799** (Probable **ANTIGEN**).

>EHO9885221.1 SPI-1 type III secretion system needle tip complex protein SipB [Salmonella enterica subsp. enterica serovar Infantis] Overall Protective Antigen Prediction = **0.4855** (Probable **ANTIGEN**).

>EHO9885222.1 SycD/LcrH family type III secretion system chaperone SicA [Salmonella enterica subsp. enterica serovar Infantis] Overall Protective Antigen Prediction = **0.5954** (Probable **ANTIGEN**).

>EHO9885223.1 SPI-1 type III secretion system export apparatus protein SpaS [Salmonella enterica subsp. enterica serovar Infantis] Overall Protective Antigen Prediction = **0.5188** (Probable **ANTIGEN**).

>EHO9885224.1 SPI-1 type III secretion system export apparatus protein SpaR [Salmonella enterica subsp. enterica serovar Infantis] Overall Protective Antigen Prediction = **0.5568** (Probable **ANTIGEN**).

>EHO9885225.1 SPI-1 type III secretion system export apparatus protein SpaQ [Salmonella enterica subsp. enterica serovar Infantis] Overall Protective Antigen Prediction = **-0.0033** (Probable **NON-ANTIGEN**).

>EHO9885226.1 SPI-1 type III secretion system export apparatus protein SpaP [Salmonella enterica subsp. enterica serovar Infantis] Overall Protective Antigen Prediction = **0.5750** (Probable **ANTIGEN**).

>EHO9885227.1 SPI-1 type III secretion system protein SpaO [Salmonella enterica subsp. enterica serovar Infantis] Overall Protective Antigen Prediction = **0.5313** (Probable **ANTIGEN**).

>EHO9885228.1 SPI-1 type III secretion system protein SpaN [Salmonella enterica subsp. enterica serovar Infantis] Overall Protective Antigen Prediction = **0.7580** (Probable **ANTIGEN**).

>EHO9885229.1 SPI-1 type III secretion system protein SpaM [Salmonella enterica subsp. enterica serovar Infantis] Overall Protective Antigen Prediction = **0.2945** (Probable **NON-ANTIGEN**).

>EHO9885230.1 SctN family type III secretion system ATPase InvC [Salmonella enterica subsp. enterica serovar Infantis] Overall Protective Antigen Prediction = **0.1736** (Probable **NON-ANTIGEN**).

>EHO9885231.1 SPI-1 type III secretion system chaperone SpaK [Salmonella enterica subsp. enterica serovar Infantis] Overall Protective Antigen Prediction = **0.4395** (Probable **ANTIGEN**).

>EHO9885232.1 type III secretion system export apparatus protein InvA [Salmonella enterica subsp. enterica serovar Infantis] Overall Protective Antigen Prediction = **0.5061** (Probable **ANTIGEN**).

>EHO9885233.1 type III secretion system gatekeeper InvE [Salmonella enterica subsp. enterica serovar Infantis] Overall Protective Antigen Prediction = **0.3335** (Probable **NON-ANTIGEN**).

>EHO9885234.1 type III secretion system outer membrane ring protein InvG [Salmonella enterica subsp. enterica serovar Infantis] Overall Protective Antigen Prediction = **0.3710** (Probable **NON-ANTIGEN**).

>EHO9885235.1 type III secretion system transcriptional activator InvF [Salmonella enterica subsp. enterica serovar Infantis] Overall Protective Antigen Prediction = **0.3090** (Probable **NON-ANTIGEN**).

>EHO9885236.1 SPI-1 type III secretion system invasion lipoprotein InvH [Salmonella enterica subsp. enterica serovar Infantis] Overall Protective Antigen Prediction = **0.3427** (Probable **NON-ANTIGEN**).

>EHO9885237.1 hypothetical protein KND05_000522 [Salmonella enterica subsp. enterica serovar Infantis] Overall Protective Antigen Prediction = **0.3635** (Probable **NON-ANTIGEN**).

>EHO9885238.1 DUF1493 family protein [Salmonella enterica subsp. enterica serovar Infantis] Overall Protective Antigen Prediction = **0.4065** (Probable **ANTIGEN**).

>EHO9885239.1 hypothetical protein KND05_000524 [Salmonella enterica subsp. enterica serovar Infantis] Overall Protective Antigen Prediction = **0.3523** (Probable **NON-ANTIGEN**).

>EHO9885240.1 DUF1778 domain-containing protein [Salmonella enterica subsp. enterica serovar Infantis] Overall Protective Antigen Prediction = **0.2569** (Probable **NON-ANTIGEN**).

>EHO9885241.1 GNAT family N-acetyltransferase [Salmonella enterica subsp. enterica serovar Infantis] Overall Protective Antigen Prediction = **0.3710** (Probable **NON-ANTIGEN**).

>EHO9885242.1 protein-serine/threonine phosphatase [Salmonella enterica subsp. enterica serovar Infantis] Overall Protective Antigen Prediction = **0.2805** (Probable **NON-ANTIGEN**).

>EHO9885243.1 cytoplasmic protein [Salmonella enterica subsp. enterica serovar Infantis] Overall Protective Antigen Prediction = **0.4456** (Probable **ANTIGEN**).

>EHO9885244.1 DNA mismatch repair protein MutS [Salmonella enterica subsp. enterica serovar Infantis] Overall Protective Antigen Prediction = **0.3864** (Probable **NON-ANTIGEN**).

>EHO9885245.1 DUF4440 domain-containing protein [Salmonella enterica subsp. enterica serovar Infantis] Overall Protective Antigen Prediction = **0.2649** (Probable **NON-ANTIGEN**).

>EHO9885246.1 MFS transporter [Salmonella enterica subsp. enterica serovar Infantis] Overall Protective Antigen Prediction = **0.5228** (Probable **ANTIGEN**).

>EHO9885247.1 LysR family transcriptional regulator [Salmonella enterica subsp. enterica serovar Infantis] Overall Protective Antigen Prediction = **0.5216** (Probable **ANTIGEN**).

>EHO9885248.1 permease [Salmonella enterica subsp. enterica serovar Infantis] Overall Protective Antigen Prediction = **0.5072** (Probable **ANTIGEN**).

>EHO9885249.1 SDR family oxidoreductase [Salmonella enterica subsp. enterica serovar Infantis] Overall Protective Antigen Prediction = **0.6115** (Probable **ANTIGEN**).

>EHO9885250.1 HPr family phosphocarrier protein [Salmonella enterica subsp. enterica serovar Infantis] Overall Protective Antigen Prediction = **0.5441** (Probable **ANTIGEN**).

>EHO9885251.1 aldolase [Salmonella enterica subsp. enterica serovar Infantis] Overall Protective Antigen Prediction = **0.3794** (Probable **NON-ANTIGEN**).

>EHO9885252.1 3-oxo-tetronate kinase [Salmonella enterica subsp. enterica serovar Infantis] Overall Protective Antigen Prediction = **0.5441** (Probable **ANTIGEN**).

>EHO9885253.1 NAD(P)-dependent oxidoreductase [Salmonella enterica subsp. enterica serovar Infantis] Overall Protective Antigen Prediction = **0.6506** (Probable **ANTIGEN**).

>EHO9885254.1 DeoR/GlpR transcriptional regulator [Salmonella enterica subsp. enterica serovar Infantis] Overall Protective Antigen Prediction = **0.2897** (Probable **NON-ANTIGEN**).

>EHO9885255.1 winged helix-turn-helix transcriptional regulator [Salmonella enterica subsp. enterica serovar Infantis] Overall Protective Antigen Prediction = **0.1705** (Probable **NON-ANTIGEN**).

>EHO9885256.1 UbiX family flavin prenyltransferase [Salmonella enterica subsp. enterica serovar Infantis] Overall Protective Antigen Prediction = **0.3004** (Probable **NON-ANTIGEN**).

>EHO9885257.1 UbiD family decarboxylase [Salmonella enterica subsp. enterica serovar Infantis] Overall Protective Antigen Prediction = **0.3737** (Probable **NON-ANTIGEN**).

>EHO9885258.1 hypothetical protein KND05_000545 [Salmonella enterica subsp. enterica serovar Infantis] Overall Protective Antigen Prediction = **0.4840** (Probable **ANTIGEN**).

>EHO9885259.1 RNA polymerase sigma factor RpoS [Salmonella enterica subsp. enterica serovar Infantis] Overall Protective Antigen Prediction = **0.3835** (Probable **NON-ANTIGEN**).

>EHO9885260.1 murein hydrolase activator NlpD [Salmonella enterica subsp. enterica serovar Infantis] Overall Protective Antigen Prediction = **0.7397** (Probable **ANTIGEN**).

>EHO9885261.1 protein-L-isoaspartate(D-aspartate) O-methyltransferase [Salmonella enterica subsp. enterica serovar Infantis] Overall Protective Antigen Prediction = **0.2467** (Probable **NON-ANTIGEN**).

>EHO9885262.1 5'/3'-nucleotidase SurE [Salmonella enterica subsp. enterica serovar Infantis] Overall Protective Antigen Prediction = **0.4577** (Probable **ANTIGEN**).

>EHO9885263.1 tRNA pseudouridine(13) synthase TruD [Salmonella enterica subsp. enterica serovar Infantis] Overall Protective Antigen Prediction = **0.4666** (Probable **ANTIGEN**).

>EHO9885264.1 2-C-methyl-D-erythritol 2,4-cyclodiphosphate synthase [Salmonella enterica subsp. enterica serovar Infantis] Overall Protective Antigen Prediction = **0.4384** (Probable **ANTIGEN**).

>EHO9885265.1 2-C-methyl-D-erythritol 4-phosphate cytidylyltransferase [Salmonella enterica subsp. enterica serovar Infantis] Overall Protective Antigen Prediction = **0.4028** (Probable **ANTIGEN**).

>EHO9885266.1 cell division protein FtsB [Salmonella enterica subsp. enterica serovar Infantis] Overall Protective Antigen Prediction = **0.7268** (Probable **ANTIGEN**).

>EHO9885267.1 DUF3561 family protein [Salmonella enterica subsp. enterica serovar Infantis] Overall Protective Antigen Prediction = **0.9121** (Probable **ANTIGEN**).

>EHO9885268.1 adenylyl-sulfate kinase [Salmonella enterica subsp. enterica serovar Infantis] Overall

Protective Antigen Prediction = **0.2880** (Probable **NON-ANTIGEN**).

>EHO9885269.1 sulfate adenylyltransferase subunit CysN [Salmonella enterica subsp. enterica serovar Infantis] Overall Protective Antigen Prediction = **0.5664** (Probable **ANTIGEN**).

>EHO9885270.1 sulfate adenylyltransferase subunit CysD [Salmonella enterica subsp. enterica serovar Infantis] Overall Protective Antigen Prediction = **0.5928** (Probable **ANTIGEN**).

>EHO9885271.1 aminopeptidase [Salmonella enterica subsp. enterica serovar Infantis] Overall Protective Antigen Prediction = **0.5354** (Probable **ANTIGEN**).

>EHO9885272.1 type I-E CRISPR-associated endoribonuclease Cas2 [Salmonella enterica subsp. enterica serovar Infantis] Overall Protective Antigen Prediction = **0.3985** (Probable **NON-ANTIGEN**).

>EHO9885273.1 type I-E CRISPR-associated endonuclease Cas1 [Salmonella enterica subsp. enterica serovar Infantis] Overall Protective Antigen Prediction = **0.4654** (Probable **ANTIGEN**).

>EHO9885274.1 type I-E CRISPR-associated protein Cas6/Cse3/CasE [Salmonella enterica subsp. enterica serovar Infantis] Overall Protective Antigen Prediction = **0.5536** (Probable **ANTIGEN**).

>EHO9885275.1 type I-E CRISPR-associated protein Cas5/CasD [Salmonella enterica subsp. enterica serovar Infantis] Overall Protective Antigen Prediction = **0.4241** (Probable **ANTIGEN**).

>EHO9885276.1 type I-E CRISPR-associated protein Cas7/Cse4/CasC [Salmonella enterica subsp. enterica serovar Infantis] Overall Protective Antigen Prediction = **0.4837** (Probable **ANTIGEN**).

>EHO9885277.1 type I-E CRISPR-associated protein Cse2/CasB [Salmonella enterica subsp. enterica serovar Infantis] Overall Protective Antigen Prediction = **0.5562** (Probable **ANTIGEN**).

>EHO9885278.1 type I-E CRISPR-associated protein Cse1/CasA [Salmonella enterica subsp. enterica serovar Infantis] Overall Protective Antigen Prediction = **0.5515** (Probable **ANTIGEN**).

>EHO9885279.1 CRISPR-associated helicase/endonuclease Cas3 [Salmonella enterica subsp. enterica serovar Infantis] Overall Protective Antigen Prediction = **0.3455** (Probable **NON-ANTIGEN**).

>EHO9885280.1 SPI-1 type III secretion system effector SopD [Salmonella enterica subsp. enterica serovar Infantis] Overall Protective Antigen Prediction = **0.4218** (Probable **ANTIGEN**).

>EHO9885281.1 phosphoadenosine phosphosulfate reductase [Salmonella enterica subsp. enterica serovar Infantis] Overall Protective Antigen Prediction = **0.3226** (Probable **NON-ANTIGEN**).

>EHO9885282.1 assimilatory sulfite reductase (NADPH) hemoprotein subunit [Salmonella enterica subsp. enterica serovar Infantis] Overall Protective Antigen Prediction = **0.3903** (Probable **NON-ANTIGEN**).

>EHO9885283.1 NADPH-dependent assimilatory sulfite reductase flavoprotein subunit [Salmonella enterica subsp. enterica serovar Infantis] Overall Protective Antigen Prediction = **0.5356** (Probable **ANTIGEN**).

>EHO9885284.1 6-carboxytetrahydropterin synthase QueD [Salmonella enterica subsp. enterica serovar Infantis] Overall Protective Antigen Prediction = **0.4796** (Probable **ANTIGEN**).

>EHO9885285.1 MBL fold metallo-hydrolase [Salmonella enterica subsp. enterica serovar Infantis]

Overall Protective Antigen Prediction = **0.1793** (Probable **NON-ANTIGEN**).

>EHO9885286.1 7-carboxy-7-deazaguanine synthase QueE [Salmonella enterica subsp. enterica serovar Infantis] Overall Protective Antigen Prediction = **0.4364** (Probable **ANTIGEN**).

>EHO9885287.1 phosphopyruvate hydratase [Salmonella enterica subsp. enterica serovar Infantis] Overall Protective Antigen Prediction = **0.5040** (Probable **ANTIGEN**).

>EHO9885288.1 CTP synthase (glutamine hydrolyzing) [Salmonella enterica subsp. enterica serovar Infantis] Overall Protective Antigen Prediction = **0.5431** (Probable **ANTIGEN**).

>EHO9885289.1 nucleoside triphosphate pyrophosphohydrolase [Salmonella enterica subsp. enterica serovar Infantis] Overall Protective Antigen Prediction = **0.3693** (Probable **NON-ANTIGEN**).

>EHO9885290.1 type II toxin-antitoxin system RelE/ParE family toxin [Salmonella enterica subsp. enterica serovar Infantis] Overall Protective Antigen Prediction = **0.2044** (Probable **NON-ANTIGEN**).

>EHO9885291.1 type II toxin-antitoxin system ParD family antitoxin [Salmonella enterica subsp. enterica serovar Infantis] Overall Protective Antigen Prediction = **0.3077** (Probable **NON-ANTIGEN**).

>EHO9885292.1 GTP diphosphokinase [Salmonella enterica subsp. enterica serovar Infantis] Overall Protective Antigen Prediction = **0.4351** (Probable **ANTIGEN**).

>EHO9885293.1 23S rRNA (uracil(1939)-C(5))-methyltransferase RlmD [Salmonella enterica subsp. enterica serovar Infantis] Overall Protective Antigen Prediction = **0.3656** (Probable **NON-ANTIGEN**).

>EHO9885294.1 two-component sensor histidine kinase BarA [Salmonella enterica subsp. enterica serovar Infantis] Overall Protective Antigen Prediction = **0.4769** (Probable **ANTIGEN**).

>EHO9885295.1 glycerate kinase [Salmonella enterica subsp. enterica serovar Infantis] Overall Protective Antigen Prediction = **0.6673** (Probable **ANTIGEN**).

>EHO9885296.1 glucarate dehydratase [Salmonella enterica subsp. enterica serovar Infantis] Overall Protective Antigen Prediction = **0.5372** (Probable **ANTIGEN**).

>EHO9885297.1 glucarate dehydratase [Salmonella enterica subsp. enterica serovar Infantis] Overall Protective Antigen Prediction = **0.5271** (Probable **ANTIGEN**).

>EHO9885298.1 galactarate/glucarate/glycerate transporter GudP [Salmonella enterica subsp. enterica serovar Infantis] Overall Protective Antigen Prediction = **0.4586** (Probable **ANTIGEN**).

>EHO9885299.1 hypothetical protein KND05_000586 [Salmonella enterica subsp. enterica serovar Infantis] Overall Protective Antigen Prediction = **0.6114** (Probable **ANTIGEN**).

>EHO9885300.1 flavodoxin [Salmonella enterica subsp. enterica serovar Infantis] Overall Protective Antigen Prediction = **0.1966** (Probable **NON-ANTIGEN**).

>EHO9885301.1 tRNA pseudouridine(65) synthase TruC [Salmonella enterica subsp. enterica serovar Infantis] Overall Protective Antigen Prediction = **0.3741** (Probable **NON-ANTIGEN**).

>EHO9885302.1 YqcC family protein [Salmonella enterica subsp. enterica serovar Infantis] Overall

Protective Antigen Prediction = **0.1892** (Probable **NON-ANTIGEN**).

>EHO9885303.1 SecY-interacting protein [Salmonella enterica subsp. enterica serovar Infantis] Overall Protective Antigen Prediction = **0.3434** (Probable **NON-ANTIGEN**).

>EHO9885304.1 NADPH-dependent 7-cyano-7-deazaguanine reductase QueF [Salmonella enterica subsp. enterica serovar Infantis] Overall Protective Antigen Prediction = **0.4177** (Probable **ANTIGEN**).

>EHO9885305.1 nucleotide 5'-monophosphate nucleosidase [Salmonella enterica subsp. enterica serovar Infantis] Overall Protective Antigen Prediction = **0.3856** (Probable **NON-ANTIGEN**).

>EHO9885306.1 HAAAP family serine/threonine permease SdaC [Salmonella enterica subsp. enterica serovar Infantis] Overall Protective Antigen Prediction = **0.6582** (Probable **ANTIGEN**).

>EHO9885307.1 L-serine ammonia-lyase II [Salmonella enterica subsp. enterica serovar Infantis] Overall Protective Antigen Prediction = **0.5488** (Probable **ANTIGEN**).

>EHO9885308.1 flap endonuclease Xni [Salmonella enterica subsp. enterica serovar Infantis] Overall Protective Antigen Prediction = **0.4716** (Probable **ANTIGEN**).

>EHO9885309.1 lactaldehyde reductase [Salmonella enterica subsp. enterica serovar Infantis] Overall Protective Antigen Prediction = **0.4724** (Probable **ANTIGEN**).

>EHO9885310.1 L-fuculose-phosphate aldolase [Salmonella enterica subsp. enterica serovar Infantis] Overall Protective Antigen Prediction = **0.4241** (Probable **ANTIGEN**).

>EHO9885311.1 L-fucose:H+ symporter permease [Salmonella enterica subsp. enterica serovar Infantis] Overall Protective Antigen Prediction = **0.5093** (Probable **ANTIGEN**).

>EHO9885312.1 L-fucose isomerase [Salmonella enterica subsp. enterica serovar Infantis] Overall Protective Antigen Prediction = **0.5511** (Probable **ANTIGEN**).

>EHO9885313.1 L-fuculokinase [Salmonella enterica subsp. enterica serovar Infantis] Overall Protective Antigen Prediction = **0.3665** (Probable **NON-ANTIGEN**).

>EHO9885314.1 L-fucose mutarotase [Salmonella enterica subsp. enterica serovar Infantis] Overall Protective Antigen Prediction = **0.5134** (Probable **ANTIGEN**).

>EHO9885315.1 L-fucose operon activator [Salmonella enterica subsp. enterica serovar Infantis] Overall Protective Antigen Prediction = **0.4836** (Probable **ANTIGEN**).

>EHO9885316.1 23S rRNA (cytidine(2498)-2'-O)-methyltransferase RlmM [Salmonella enterica subsp. enterica serovar Infantis] Overall Protective Antigen Prediction = **0.3968** (Probable **NON-ANTIGEN**).

>EHO9885317.1 DUF423 domain-containing protein [Salmonella enterica subsp. enterica serovar Infantis] Overall Protective Antigen Prediction = **0.3630** (Probable **NON-ANTIGEN**).

>EHO9885318.1 glycine cleavage system transcriptional regulator GcvA [Salmonella enterica subsp. enterica serovar Infantis] Overall Protective Antigen Prediction = **0.4081** (Probable **ANTIGEN**).

>EHO9885319.1 YgdI/YgdR family lipoprotein [Salmonella enterica subsp. enterica serovar Infantis] Overall Protective Antigen Prediction = **0.6850** (Probable **ANTIGEN**).

>EHO9885320.1 cysteine desulfurase CsdA [Salmonella enterica subsp. enterica serovar Infantis] Overall Protective Antigen Prediction = **0.3894** (Probable **NON-ANTIGEN**).

>EHO9885321.1 cysteine desulfurase sulfur acceptor subunit CsdE [Salmonella enterica subsp. enterica serovar Infantis] Overall Protective Antigen Prediction = **0.3381** (Probable **NON-ANTIGEN**).

>EHO9885322.1 EamA family transporter RarD [Salmonella enterica subsp. enterica serovar Infantis] Overall Protective Antigen Prediction = **0.5799** (Probable **ANTIGEN**).

>EHO9885323.1 tRNA cyclic N6-threonylcarbamoyladenosine(37) synthase TcdA [Salmonella enterica subsp. enterica serovar Infantis] Overall Protective Antigen Prediction = **0.4836** (Probable **ANTIGEN**).

>EHO9885324.1 murein transglycosylase A [Salmonella enterica subsp. enterica serovar Infantis] Overall Protective Antigen Prediction = **0.3918** (Probable **NON-ANTIGEN**).

>EHO9885325.1 N-acetylmuramoyl-L-alanine amidase AmiC [Salmonella enterica subsp. enterica serovar Infantis] Overall Protective Antigen Prediction = **0.4868** (Probable **ANTIGEN**).

>EHO9885326.1 amino-acid N-acetyltransferase [Salmonella enterica subsp. enterica serovar Infantis] Overall Protective Antigen Prediction = **0.4268** (Probable **ANTIGEN**).

>EHO9885327.1 exodeoxyribonuclease V subunit alpha [Salmonella enterica subsp. enterica serovar Infantis] Overall Protective Antigen Prediction = **0.3514** (Probable **NON-ANTIGEN**).

>EHO9885328.1 exodeoxyribonuclease V subunit beta [Salmonella enterica subsp. enterica serovar Infantis] Overall Protective Antigen Prediction = **0.4605** (Probable **ANTIGEN**).

>EHO9885329.1 pitrilysin [Salmonella enterica subsp. enterica serovar Infantis] Overall Protective Antigen Prediction = **0.4054** (Probable **ANTIGEN**).

>EHO9885330.1 exodeoxyribonuclease V subunit gamma [Salmonella enterica subsp. enterica serovar Infantis] Overall Protective Antigen Prediction = **0.4258** (Probable **ANTIGEN**).

>EHO9885331.1 prepilin-type N-terminal cleavage/methylation domain-containing protein [Salmonella enterica subsp. enterica serovar Infantis] Overall Protective Antigen Prediction = **0.4889** (Probable **ANTIGEN**).

>EHO9885332.1 DUF2509 family protein [Salmonella enterica subsp. enterica serovar Infantis] Overall Protective Antigen Prediction = **0.2621** (Probable **NON-ANTIGEN**).

>EHO9885333.1 prepilin peptidase-dependent protein [Salmonella enterica subsp. enterica serovar Infantis] Overall Protective Antigen Prediction = **0.4276** (Probable **ANTIGEN**).

>EHO9885334.1 prepilin peptidase-dependent protein [Salmonella enterica subsp. enterica serovar Infantis] Overall Protective Antigen Prediction = **0.2285** (Probable **NON-ANTIGEN**).

>EHO9885335.1 thymidylate synthase [Salmonella enterica subsp. enterica serovar Infantis] Overall Protective Antigen Prediction = **0.6198** (Probable **ANTIGEN**).

>EHO9885336.1 prolipoprotein diacylglyceryl transferase [Salmonella enterica subsp. enterica serovar Infantis] Overall Protective Antigen Prediction = **0.4561** (Probable **ANTIGEN**).

>EHO9885337.1 phosphoenolpyruvate--protein phosphotransferase [Salmonella enterica subsp. enterica serovar Infantis] Overall Protective Antigen Prediction = **0.3650** (Probable **NON-ANTIGEN**).

>EHO9885338.1 RNA pyrophosphohydrolase [Salmonella enterica subsp. enterica serovar Infantis] Overall Protective Antigen Prediction = **0.3216** (Probable **NON-ANTIGEN**).

>EHO9885339.1 DNA mismatch repair endonuclease MutH [Salmonella enterica subsp. enterica serovar Infantis] Overall Protective Antigen Prediction = **0.4077** (Probable **ANTIGEN**).

>EHO9885340.1 TerC family protein [Salmonella enterica subsp. enterica serovar Infantis] Overall Protective Antigen Prediction = **0.4394** (Probable **ANTIGEN**).

>EHO9885341.1 YgdI/YgdR family lipoprotein [Salmonella enterica subsp. enterica serovar Infantis] Overall Protective Antigen Prediction = **0.5087** (Probable **ANTIGEN**).

>EHO9885342.1 NADP(H)-dependent aldo-keto reductase [Salmonella enterica subsp. enterica serovar Infantis] Overall Protective Antigen Prediction = **0.3188** (Probable **NON-ANTIGEN**).

>EHO9885343.1 lysophospholipid transporter LplT [Salmonella enterica subsp. enterica serovar Infantis] Overall Protective Antigen Prediction = **0.4132** (Probable **ANTIGEN**).

>EHO9885344.1 bifunctional acyl-ACP--phospholipid O-acyltransferase/long-chain-fatty-acid--ACP ligase [Salmonella enterica subsp. enterica serovar Infantis] Overall Protective Antigen Prediction = **0.3812** (Probable **NON-ANTIGEN**).

>EHO9885345.1 HTH-type transcriptional regulator GalR [Salmonella enterica subsp. enterica serovar Infantis] Overall Protective Antigen Prediction = **0.3470** (Probable **NON-ANTIGEN**).

>EHO9885346.1 LacI family DNA-binding transcriptional regulator [Salmonella enterica subsp. enterica serovar Infantis] Overall Protective Antigen Prediction = **0.2933** (Probable **NON-ANTIGEN**).

>EHO9885347.1 diaminopimelate decarboxylase [Salmonella enterica subsp. enterica serovar Infantis] Overall Protective Antigen Prediction = **0.4720** (Probable **ANTIGEN**).

>EHO9885348.1 LysR family transcriptional regulator [Salmonella enterica subsp. enterica serovar Infantis] Overall Protective Antigen Prediction = **0.3095** (Probable **NON-ANTIGEN**).

>EHO9885349.1 aspartate/glutamate racemase [Salmonella enterica subsp. enterica serovar Infantis] Overall Protective Antigen Prediction = **0.3116** (Probable **NON-ANTIGEN**).

>EHO9885350.1 arabinose-proton symporter AraE [Salmonella enterica subsp. enterica serovar Infantis] Overall Protective Antigen Prediction = **0.4293** (Probable **ANTIGEN**).

>EHO9885351.1 2-dehydro-3-deoxy-D-gluconate 5-dehydrogenase KduD [Salmonella enterica subsp. enterica serovar Infantis] Overall Protective Antigen Prediction = **0.4197** (Probable **ANTIGEN**).

>EHO9885352.1 5-dehydro-4-deoxy-D-glucuronate isomerase [Salmonella enterica subsp. enterica serovar Infantis] Overall Protective Antigen Prediction = **0.5269** (Probable **ANTIGEN**).

>EHO9885353.1 acetyl-CoA C-acetyltransferase [Salmonella enterica subsp. enterica serovar Infantis] Overall Protective Antigen Prediction = **0.4352** (Probable **ANTIGEN**).

>EHO9885354.1 LysR family transcriptional regulator [Salmonella enterica subsp. enterica serovar Infantis] Overall Protective Antigen Prediction = **0.4004** (Probable **ANTIGEN**).

>EHO9885355.1 multidrug/biocide efflux PACE transporter [Salmonella enterica subsp. enterica serovar Infantis] Overall Protective Antigen Prediction = **0.5741** (Probable **ANTIGEN**).

>EHO9885356.1 transporter [Salmonella enterica subsp. enterica serovar Infantis] Overall Protective Antigen Prediction = **0.5586** (Probable **ANTIGEN**).

>EHO9885357.1 Ni(II)/Co(II)-binding transcriptional repressor RcnR [Salmonella enterica subsp. enterica serovar Infantis] Overall Protective Antigen Prediction = **0.3492** (Probable **NON-ANTIGEN**).

>EHO9885358.1 nickel/cobalt efflux protein RcnA [Salmonella enterica subsp. enterica serovar Infantis] Overall Protective Antigen Prediction = **0.4247** (Probable **ANTIGEN**).

>EHO9885359.1 transcriptional regulator [Salmonella enterica subsp. enterica serovar Infantis] Overall Protective Antigen Prediction = **0.2736** (Probable **NON-ANTIGEN**).

>EHO9885360.1 CaiF/GrlA family transcriptional regulator [Salmonella enterica subsp. enterica serovar Infantis] Overall Protective Antigen Prediction = **0.4616** (Probable **ANTIGEN**).

>EHO9885361.1 fimbrial protein [Salmonella enterica subsp. enterica serovar Infantis] Overall Protective Antigen Prediction = **0.6187** (Probable **ANTIGEN**).

>EHO9885362.1 fimbria/pilus periplasmic chaperone [Salmonella enterica subsp. enterica serovar Infantis] Overall Protective Antigen Prediction = **0.3748** (Probable **NON-ANTIGEN**).

>EHO9885363.1 fimbrial outer membrane usher protein StdB [Salmonella enterica subsp. enterica serovar Infantis] Overall Protective Antigen Prediction = **0.6723** (Probable **ANTIGEN**).

>EHO9885364.1 fimbrial protein [Salmonella enterica subsp. enterica serovar Infantis] Overall Protective Antigen Prediction = **0.5092** (Probable **ANTIGEN**).

>EHO9885365.1 YfdX family protein [Salmonella enterica subsp. enterica serovar Infantis] Overall Protective Antigen Prediction = **0.4781** (Probable **ANTIGEN**).

>EHO9885366.1 porin family protein [Salmonella enterica subsp. enterica serovar Infantis] Overall Protective Antigen Prediction = **0.7960** (Probable **ANTIGEN**).

>EHO9885367.1 SIS domain-containing protein [Salmonella enterica subsp. enterica serovar Infantis] Overall Protective Antigen Prediction = **0.5176** (Probable **ANTIGEN**).

>EHO9885368.1 SDR family oxidoreductase [Salmonella enterica subsp. enterica serovar Infantis] Overall Protective Antigen Prediction = **0.3999** (Probable **NON-ANTIGEN**).

>EHO9885369.1 ribulose-phosphate 3-epimerase [Salmonella enterica subsp. enterica serovar Infantis] Overall Protective Antigen Prediction = **0.3931** (Probable **NON-ANTIGEN**).

>EHO9885370.1 PTS galactitol transporter subunit IIC [Salmonella enterica subsp. enterica serovar Infantis] Overall Protective Antigen Prediction = **0.5138** (Probable **ANTIGEN**).

>EHO9885371.1 PTS sugar transporter subunit IIB [Salmonella enterica subsp. enterica serovar

Infantis] Overall Protective Antigen Prediction = **0.5065** (Probable **ANTIGEN**).

>EHO9885372.1 PTS galactitol transporter subunit IIA [Salmonella enterica subsp. enterica serovar Infantis] Overall Protective Antigen Prediction = **0.3251** (Probable **NON-ANTIGEN**).

>EHO9885373.1 DeoR/GlpR transcriptional regulator [Salmonella enterica subsp. enterica serovar Infantis] Overall Protective Antigen Prediction = **0.1469** (Probable **NON-ANTIGEN**).

>EHO9885374.1 RpiB/LacA/LacB family sugar-phosphate isomerase [Salmonella enterica subsp. enterica serovar Infantis] Overall Protective Antigen Prediction = **0.5376** (Probable **ANTIGEN**).

>EHO9885375.1 PTS galactitol transporter subunit IIC [Salmonella enterica subsp. enterica serovar Infantis] Overall Protective Antigen Prediction = **0.3807** (Probable **NON-ANTIGEN**).

>EHO9885376.1 PTS sugar transporter subunit IIB [Salmonella enterica subsp. enterica serovar Infantis] Overall Protective Antigen Prediction = **0.2847** (Probable **NON-ANTIGEN**).

>EHO9885377.1 PTS sugar transporter subunit IIA [Salmonella enterica subsp. enterica serovar Infantis] Overall Protective Antigen Prediction = **0.2460** (Probable **NON-ANTIGEN**).

>EHO9885378.1 hypothetical protein KND05_000667 [Salmonella enterica subsp. enterica serovar Infantis] Overall Protective Antigen Prediction = **0.4136** (Probable **ANTIGEN**).

>EHO9885379.1 As(III)-sensing metalloregulatory transcriptional repressor ArsR [Salmonella enterica subsp. enterica serovar Infantis] Overall Protective Antigen Prediction = **0.2950** (Probable **NON-ANTIGEN**).

>EHO9885380.1 tyrosine-type recombinase/integrase [Salmonella enterica subsp. enterica serovar Infantis] Overall Protective Antigen Prediction = **0.4666** (Probable **ANTIGEN**).

>EHO9885381.1 peptidoglycan DD-metalloendopeptidase family protein [Salmonella enterica subsp. enterica serovar Infantis] Overall Protective Antigen Prediction = **0.7027** (Probable **ANTIGEN**).

>EHO9885382.1 isopentenyl-diphosphate Delta-isomerase [Salmonella enterica subsp. enterica serovar Infantis] Overall Protective Antigen Prediction = **0.5824** (Probable **ANTIGEN**).

>EHO9885383.1 lysine--tRNA ligase [Salmonella enterica subsp. enterica serovar Infantis] Overall Protective Antigen Prediction = **0.3862** (Probable **NON-ANTIGEN**).

>EHO9885384.1 peptide chain release factor 2 [Salmonella enterica subsp. enterica serovar Infantis] Overall Protective Antigen Prediction = **0.6991** (Probable **ANTIGEN**).

>EHO9885385.1 single-stranded-DNA-specific exonuclease RecJ [Salmonella enterica subsp. enterica serovar Infantis] Overall Protective Antigen Prediction = **0.4689** (Probable **ANTIGEN**).

>EHO9885386.1 bifunctional protein-disulfide isomerase/oxidoreductase DsbC [Salmonella enterica subsp. enterica serovar Infantis] Overall Protective Antigen Prediction = **0.5284** (Probable **ANTIGEN**).

>EHO9885387.1 site-specific tyrosine recombinase XerD [Salmonella enterica subsp. enterica serovar Infantis] Overall Protective Antigen Prediction = **0.2943** (Probable **NON-ANTIGEN**).

>EHO9885388.1 flavodoxin FldB [Salmonella enterica subsp. enterica serovar Infantis] Overall Protective Antigen Prediction = **0.2709** (Probable **NON-ANTIGEN**).

>EHO9885389.1 protein YgfX [Salmonella enterica subsp. enterica serovar Infantis] Overall Protective Antigen Prediction = **0.8430** (Probable **ANTIGEN**).

>EHO9885390.1 FAD assembly factor SdhE [Salmonella enterica subsp. enterica serovar Infantis] Overall Protective Antigen Prediction = **0.4617** (Probable **ANTIGEN**).

>EHO9885391.1 tRNA-modifying protein YgfZ [Salmonella enterica subsp. enterica serovar Infantis] Overall Protective Antigen Prediction = **0.3992** (Probable **NON-ANTIGEN**).

>EHO9885392.1 hemolysin III family protein [Salmonella enterica subsp. enterica serovar Infantis] Overall Protective Antigen Prediction = **0.4220** (Probable **ANTIGEN**).

>EHO9885393.1 ASCH domain-containing protein [Salmonella enterica subsp. enterica serovar Infantis] Overall Protective Antigen Prediction = **0.4379** (Probable **ANTIGEN**).

>EHO9885394.1 6-phospho-beta-glucosidase BglA [Salmonella enterica subsp. enterica serovar Infantis] Overall Protective Antigen Prediction = **0.5556** (Probable **ANTIGEN**).

>EHO9885395.1 transporter [Salmonella enterica subsp. enterica serovar Infantis] Overall Protective Antigen Prediction = **0.7486** (Probable **ANTIGEN**).

>EHO9885396.1 aminomethyl-transferring glycine dehydrogenase [Salmonella enterica subsp. enterica serovar Infantis] Overall Protective Antigen Prediction = **0.5033** (Probable **ANTIGEN**).

>EHO9885397.1 glycine cleavage system protein GcvH [Salmonella enterica subsp. enterica serovar Infantis] Overall Protective Antigen Prediction = **0.5820** (Probable **ANTIGEN**).

>EHO9885398.1 glycine cleavage system aminomethyltransferase GcvT [Salmonella enterica subsp. enterica serovar Infantis] Overall Protective Antigen Prediction = **0.4893** (Probable **ANTIGEN**).

>EHO9885399.1 FAD-dependent 2-octaprenylphenol hydroxylase [Salmonella enterica subsp. enterica serovar Infantis] Overall Protective Antigen Prediction = **0.3556** (Probable **NON-ANTIGEN**).

>EHO9885400.1 2-octaprenyl-6-methoxyphenyl hydroxylase [Salmonella enterica subsp. enterica serovar Infantis] Overall Protective Antigen Prediction = **0.3805** (Probable **NON-ANTIGEN**).

>EHO9885401.1 Xaa-Pro aminopeptidase [Salmonella enterica subsp. enterica serovar Infantis] Overall Protective Antigen Prediction = **0.3607** (Probable **NON-ANTIGEN**).

>EHO9885402.1 YecA family protein [Salmonella enterica subsp. enterica serovar Infantis] Overall Protective Antigen Prediction = **0.3446** (Probable **NON-ANTIGEN**).

>EHO9885403.1 cell division protein ZapA [Salmonella enterica subsp. enterica serovar Infantis] Overall Protective Antigen Prediction = **0.7266** (Probable **ANTIGEN**).

>EHO9885404.1 5-formyltetrahydrofolate cyclo-ligase [Salmonella enterica subsp. enterica serovar Infantis] Overall Protective Antigen Prediction = **0.4626** (Probable **ANTIGEN**).

>EHO9885405.1 phosphoglycerate dehydrogenase [Salmonella enterica subsp. enterica serovar Infantis] Overall Protective Antigen Prediction = **0.4464** (Probable **ANTIGEN**).

>EHO9885406.1 ribose-5-phosphate isomerase RpiA [Salmonella enterica subsp. enterica serovar

Infantis] Overall Protective Antigen Prediction = **0.3807** (Probable **NON-ANTIGEN**).

>EHO9885407.1 DNA-binding transcriptional regulator ArgP [Salmonella enterica subsp. enterica serovar Infantis] Overall Protective Antigen Prediction = **0.4518** (Probable **ANTIGEN**).

>EHO9885408.1 oxidative stress defense protein [Salmonella enterica subsp. enterica serovar Infantis] Overall Protective Antigen Prediction = **0.6665** (Probable **ANTIGEN**).

>EHO9885409.1 arginine exporter ArgO [Salmonella enterica subsp. enterica serovar Infantis] Overall Protective Antigen Prediction = **0.4690** (Probable **ANTIGEN**).

>EHO9885410.1 small-conductance mechanosensitive channel MscS [Salmonella enterica subsp. enterica serovar Infantis] Overall Protective Antigen Prediction = **0.3867** (Probable **NON-ANTIGEN**).

>EHO9885411.1 class II fructose-bisphosphate aldolase [Salmonella enterica subsp. enterica serovar Infantis] Overall Protective Antigen Prediction = **0.4253** (Probable **ANTIGEN**).

>EHO9885412.1 phosphoglycerate kinase [Salmonella enterica subsp. enterica serovar Infantis] Overall Protective Antigen Prediction = **0.4042** (Probable **ANTIGEN**).

>EHO9885413.1 erythrose-4-phosphate dehydrogenase [Salmonella enterica subsp. enterica serovar Infantis] Overall Protective Antigen Prediction = **0.5023** (Probable **ANTIGEN**).

>EHO9885414.1 DNA-binding protein [Salmonella enterica subsp. enterica serovar Infantis] Overall Protective Antigen Prediction = **0.6003** (Probable **ANTIGEN**).

>EHO9885415.1 ECF transporter S component [Salmonella enterica subsp. enterica serovar Infantis] Overall Protective Antigen Prediction = **0.2934** (Probable **NON-ANTIGEN**).

>EHO9885416.1 energy-coupling factor transporter transmembrane protein EcfT [Salmonella enterica subsp. enterica serovar Infantis] Overall Protective Antigen Prediction = **0.2670** (Probable **NON-ANTIGEN**).

>EHO9885417.1 energy-coupling factor ABC transporter ATP-binding protein [Salmonella enterica subsp. enterica serovar Infantis] Overall Protective Antigen Prediction = **0.4636** (Probable **ANTIGEN**).

>EHO9885418.1 ATP-binding cassette domain-containing protein [Salmonella enterica subsp. enterica serovar Infantis] Overall Protective Antigen Prediction = **0.5420** (Probable **ANTIGEN**).

>EHO9885419.1 transketolase [Salmonella enterica subsp. enterica serovar Infantis] Overall Protective Antigen Prediction = **0.4069** (Probable **ANTIGEN**).

>EHO9885420.1 M48 family metallopeptidase [Salmonella enterica subsp. enterica serovar Infantis] Overall Protective Antigen Prediction = **0.5816** (Probable **ANTIGEN**).

>EHO9885421.1 agmatinase [Salmonella enterica subsp. enterica serovar Infantis] Overall Protective Antigen Prediction = **0.4670** (Probable **ANTIGEN**).

>EHO9885422.1 YhcH/YjgK/YiaL family protein [Salmonella enterica subsp. enterica serovar Infantis] Overall Protective Antigen Prediction = **0.3692** (Probable **NON-ANTIGEN**).

>EHO9885423.1 Ldh family oxidoreductase [Salmonella enterica subsp. enterica serovar Infantis]

Overall Protective Antigen Prediction = **0.3840** (Probable **NON-ANTIGEN**).

>EHO9885424.1 zinc-binding alcohol dehydrogenase family protein [Salmonella enterica subsp. enterica serovar Infantis] Overall Protective Antigen Prediction = **0.4538** (Probable **ANTIGEN**).

>EHO9885425.1 mannitol dehydrogenase family protein [Salmonella enterica subsp. enterica serovar Infantis] Overall Protective Antigen Prediction = **0.3847** (Probable **NON-ANTIGEN**).

>EHO9885426.1 FadR family transcriptional regulator [Salmonella enterica subsp. enterica serovar Infantis] Overall Protective Antigen Prediction = **0.2568** (Probable **NON-ANTIGEN**).

>EHO9885427.1 hypothetical protein KND05_000721 [Salmonella enterica subsp. enterica serovar Infantis] Overall Protective Antigen Prediction = **0.6577** (Probable **ANTIGEN**).

>EHO9885428.1 biosynthetic arginine decarboxylase [Salmonella enterica subsp. enterica serovar Infantis] Overall Protective Antigen Prediction = **0.4721** (Probable **ANTIGEN**).

>EHO9885429.1 acid stress response protein YqgB [Salmonella enterica subsp. enterica serovar Infantis] Overall Protective Antigen Prediction = **0.3017** (Probable **NON-ANTIGEN**).

>EHO9885430.1 hypothetical protein KND05_000724 [Salmonella enterica subsp. enterica serovar Infantis] Overall Protective Antigen Prediction = **0.8900** (Probable **ANTIGEN**).

>EHO9885431.1 hypothetical protein KND05_000725 [Salmonella enterica subsp. enterica serovar Infantis] Overall Protective Antigen Prediction = **0.5136** (Probable **ANTIGEN**).

>EHO9885432.1 methionine adenosyltransferase [Salmonella enterica subsp. enterica serovar Infantis] Overall Protective Antigen Prediction = **0.3494** (Probable **NON-ANTIGEN**).

>EHO9885433.1 galactose/proton symporter [Salmonella enterica subsp. enterica serovar Infantis] Overall Protective Antigen Prediction = **0.4234** (Probable **ANTIGEN**).

>EHO9885434.1 SprT family zinc-dependent metalloprotease [Salmonella enterica subsp. enterica serovar Infantis] Overall Protective Antigen Prediction = **0.5428** (Probable **ANTIGEN**).

>EHO9885435.1 deoxyribonuclease I [Salmonella enterica subsp. enterica serovar Infantis] Overall Protective Antigen Prediction = **0.6767** (Probable **ANTIGEN**).

>EHO9885436.1 16S rRNA (uracil(1498)-N(3))-methyltransferase [Salmonella enterica subsp. enterica serovar Infantis] Overall Protective Antigen Prediction = **0.6442** (Probable **ANTIGEN**).

>EHO9885437.1 glutathione synthase [Salmonella enterica subsp. enterica serovar Infantis] Overall Protective Antigen Prediction = **0.5170** (Probable **ANTIGEN**).

>EHO9885438.1 YqgE/AlgH family protein [Salmonella enterica subsp. enterica serovar Infantis] Overall Protective Antigen Prediction = **0.2702** (Probable **NON-ANTIGEN**).

>EHO9885439.1 Holliday junction resolvase RuvX [Salmonella enterica subsp. enterica serovar Infantis] Overall Protective Antigen Prediction = **0.5498** (Probable **ANTIGEN**).

>EHO9885440.1 IclR family transcriptional regulator [Salmonella enterica subsp. enterica serovar Infantis] Overall Protective Antigen Prediction = **0.3729** (Probable **NON-ANTIGEN**).

>EHO9885441.1 type IV pilus twitching motility protein PilT [Salmonella enterica subsp. enterica

serovar Infantis] Overall Protective Antigen Prediction = **0.4796** (Probable **ANTIGEN**).

>EHO9885442.1 YggS family pyridoxal phosphate-dependent enzyme [Salmonella enterica subsp. enterica serovar Infantis] Overall Protective Antigen Prediction = **0.4117** (Probable **ANTIGEN**).

>EHO9885443.1 YggT family protein [Salmonella enterica subsp. enterica serovar Infantis] Overall Protective Antigen Prediction = **0.2335** (Probable **NON-ANTIGEN**).

>EHO9885444.1 YggU family protein [Salmonella enterica subsp. enterica serovar Infantis] Overall Protective Antigen Prediction = **0.6105** (Probable **ANTIGEN**).

>EHO9885445.1 XTP/dITP diphosphatase [Salmonella enterica subsp. enterica serovar Infantis] Overall Protective Antigen Prediction = **0.4108** (Probable **ANTIGEN**).

>EHO9885446.1 radical SAM family heme chaperone HemW [Salmonella enterica subsp. enterica serovar Infantis] Overall Protective Antigen Prediction = **0.3891** (Probable **NON-ANTIGEN**).

>EHO9885447.1 DUF1202 domain-containing protein [Salmonella enterica subsp. enterica serovar Infantis] Overall Protective Antigen Prediction = **0.5880** (Probable **ANTIGEN**).

>EHO9885448.1 L-asparaginase 2 [Salmonella enterica subsp. enterica serovar Infantis] Overall Protective Antigen Prediction = **0.4987** (Probable **ANTIGEN**).

>EHO9885449.1 DUF2884 domain-containing protein [Salmonella enterica subsp. enterica serovar Infantis] Overall Protective Antigen Prediction = **0.4754** (Probable **ANTIGEN**).

>EHO9885450.1 YggL family protein [Salmonella enterica subsp. enterica serovar Infantis] Overall Protective Antigen Prediction = **0.4332** (Probable **ANTIGEN**).

>EHO9885451.1 tRNA (guanosine(46)-N7)-methyltransferase TrmB [Salmonella enterica subsp. enterica serovar Infantis] Overall Protective Antigen Prediction = **0.4721** (Probable **ANTIGEN**).

>EHO9885452.1 A/G-specific adenine glycosylase [Salmonella enterica subsp. enterica serovar Infantis] Overall Protective Antigen Prediction = **0.2985** (Probable **NON-ANTIGEN**).

>EHO9885453.1 oxidative damage protection protein [Salmonella enterica subsp. enterica serovar Infantis] Overall Protective Antigen Prediction = **0.4917** (Probable **ANTIGEN**).

>EHO9885454.1 membrane-bound lytic murein transglycosylase MltC [Salmonella enterica subsp. enterica serovar Infantis] Overall Protective Antigen Prediction = **0.4592** (Probable **ANTIGEN**).

>EHO9885455.1 thymidine kinase [Salmonella enterica subsp. enterica serovar Infantis] Overall Protective Antigen Prediction = **0.4489** (Probable **ANTIGEN**).

>EHO9885456.1 bifunctional acetaldehyde-CoA/alcohol dehydrogenase [Salmonella enterica subsp. enterica serovar Infantis] Overall Protective Antigen Prediction = **0.4045** (Probable **ANTIGEN**).

>EHO9885457.1 YchE family NAAT transporter [Salmonella enterica subsp. enterica serovar Infantis] Overall Protective Antigen Prediction = **0.6277** (Probable **ANTIGEN**).

>EHO9885458.1 oligopeptide ABC transporter substrate-binding protein OppA [Salmonella enterica subsp. enterica serovar Infantis] Overall Protective Antigen Prediction = **0.4084** (Probable **ANTIGEN**).

>EHO9885459.1 oligopeptide ABC transporter permease OppB [Salmonella enterica subsp. enterica serovar Infantis] Overall Protective Antigen Prediction = **0.5221** (Probable **ANTIGEN**).

>EHO9885460.1 oligopeptide ABC transporter permease OppC [Salmonella enterica subsp. enterica serovar Infantis] Overall Protective Antigen Prediction = **0.4936** (Probable **ANTIGEN**).

>EHO9885461.1 ABC transporter ATP-binding protein [Salmonella enterica subsp. enterica serovar Infantis] Overall Protective Antigen Prediction = **0.4503** (Probable **ANTIGEN**).

>EHO9885462.1 murein tripeptide/oligopeptide ABC transporter ATP binding protein OppF [Salmonella enterica subsp. enterica serovar Infantis] Overall Protective Antigen Prediction = **0.5559** (Probable **ANTIGEN**).

>EHO9885463.1 ion transporter [Salmonella enterica subsp. enterica serovar Infantis] Overall Protective Antigen Prediction = **0.6090** (Probable **ANTIGEN**).

>EHO9885464.1 YciU family protein [Salmonella enterica subsp. enterica serovar Infantis] Overall Protective Antigen Prediction = **0.6929** (Probable **ANTIGEN**).

>EHO9885465.1 cardiolipin synthase [Salmonella enterica subsp. enterica serovar Infantis] Overall Protective Antigen Prediction = **0.4833** (Probable **ANTIGEN**).

>EHO9885466.1 YciY family protein [Salmonella enterica subsp. enterica serovar Infantis] Overall Protective Antigen Prediction = **0.2889** (Probable **NON-ANTIGEN**).

>EHO9885467.1 YciI family protein [Salmonella enterica subsp. enterica serovar Infantis] Overall Protective Antigen Prediction = **0.4357** (Probable **ANTIGEN**).

>EHO9885468.1 TonB system transport protein TonB [Salmonella enterica subsp. enterica serovar Infantis] Overall Protective Antigen Prediction = **0.6331** (Probable **ANTIGEN**).

>EHO9885469.1 acyl-CoA thioester hydrolase YciA [Salmonella enterica subsp. enterica serovar Infantis] Overall Protective Antigen Prediction = **0.5611** (Probable **ANTIGEN**).

>EHO9885470.1 septation protein A [Salmonella enterica subsp. enterica serovar Infantis] Overall Protective Antigen Prediction = **0.4271** (Probable **ANTIGEN**).

>EHO9885471.1 UPF0259 family protein [Salmonella enterica subsp. enterica serovar Infantis] Overall Protective Antigen Prediction = **0.4280** (Probable **ANTIGEN**).

>EHO9885472.1 YkgJ family cysteine cluster protein [Salmonella enterica subsp. enterica serovar Infantis] Overall Protective Antigen Prediction = **0.7223** (Probable **ANTIGEN**).

>EHO9885473.1 outer membrane protein OmpW [Salmonella enterica subsp. enterica serovar Infantis] Overall Protective Antigen Prediction = **0.8478** (Probable **ANTIGEN**).

>EHO9885474.1 ferritin-like domain-containing protein [Salmonella enterica subsp. enterica serovar Infantis] Overall Protective Antigen Prediction = **0.4152** (Probable **ANTIGEN**).

>EHO9885475.1 ferritin-like domain-containing protein [Salmonella enterica subsp. enterica serovar Infantis] Overall Protective Antigen Prediction = **0.5874** (Probable **ANTIGEN**).

>EHO9885476.1 general stress protein [Salmonella enterica subsp. enterica serovar Infantis] Overall Protective Antigen Prediction = **1.7335** (Probable **ANTIGEN**).

>EHO9885477.1 tryptophan synthase subunit alpha [Salmonella enterica subsp. enterica serovar Infantis] Overall Protective Antigen Prediction = **0.3623** (Probable **NON-ANTIGEN**).

>EHO9885478.1 tryptophan synthase subunit beta [Salmonella enterica subsp. enterica serovar Infantis] Overall Protective Antigen Prediction = **0.4218** (Probable **ANTIGEN**).

>EHO9885479.1 bifunctional indole-3-glycerol-phosphate synthase TrpC/phosphoribosylanthranilate isomerase TrpF [Salmonella enterica subsp. enterica serovar Infantis] Overall Protective Antigen Prediction = **0.3836** (Probable **NON-ANTIGEN**).

>EHO9885480.1 bifunctional anthranilate synthase glutamate amidotransferase component TrpG/anthranilate phosphoribosyltransferase TrpD [Salmonella enterica subsp. enterica serovar Infantis] Overall Protective Antigen Prediction = **0.5522** (Probable **ANTIGEN**).

>EHO9885481.1 anthranilate synthase component I [Salmonella enterica subsp. enterica serovar Infantis] Overall Protective Antigen Prediction = **0.4571** (Probable **ANTIGEN**).

>EHO9885482.1 trp operon leader peptide [Salmonella enterica subsp. enterica serovar Infantis] Overall Protective Antigen Prediction = **0.1211** (Probable **NON-ANTIGEN**).

>EHO9885483.1 5'-3' exoribonuclease [Salmonella enterica subsp. enterica serovar Infantis] Overall Protective Antigen Prediction = **0.3340** (Probable **NON-ANTIGEN**).

>EHO9885484.1 threonylcarbamoyl-AMP synthase [Salmonella enterica subsp. enterica serovar Infantis] Overall Protective Antigen Prediction = **0.2819** (Probable **NON-ANTIGEN**).

>EHO9885485.1 23S rRNA pseudouridine(2605) synthase RluB [Salmonella enterica subsp. enterica serovar Infantis] Overall Protective Antigen Prediction = **0.5685** (Probable **ANTIGEN**).

>EHO9885486.1 cob(I)yrinic acid a,c-diamide adenosyltransferase [Salmonella enterica subsp. enterica serovar Infantis] Overall Protective Antigen Prediction = **0.5151** (Probable **ANTIGEN**).

>EHO9885487.1 YciK family oxidoreductase [Salmonella enterica subsp. enterica serovar Infantis] Overall Protective Antigen Prediction = **0.6270** (Probable **ANTIGEN**).

>EHO9885488.1 protease SohB [Salmonella enterica subsp. enterica serovar Infantis] Overall Protective Antigen Prediction = **0.4413** (Probable **ANTIGEN**).

>EHO9885489.1 YciN family protein [Salmonella enterica subsp. enterica serovar Infantis] Overall Protective Antigen Prediction = **-0.0991** (Probable **NON-ANTIGEN**).

>EHO9885490.1 type I DNA topoisomerase [Salmonella enterica subsp. enterica serovar Infantis] Overall Protective Antigen Prediction = **0.4469** (Probable **ANTIGEN**).

>EHO9885491.1 HTH-type transcriptional regulator CysB [Salmonella enterica subsp. enterica serovar Infantis] Overall Protective Antigen Prediction = **0.3939** (Probable **NON-ANTIGEN**).

>EHO9885492.1 YmiA family putative membrane protein [Salmonella enterica subsp. enterica serovar Infantis] Overall Protective Antigen Prediction = **0.6882** (Probable **ANTIGEN**).

>EHO9885493.1 aconitate hydratase AcnA [Salmonella enterica subsp. enterica serovar Infantis] Overall Protective Antigen Prediction = **0.4461** (Probable **ANTIGEN**).

>EHO9885494.1 GTP cyclohydrolase II [Salmonella enterica subsp. enterica serovar Infantis] Overall Protective Antigen Prediction = **0.3571** (Probable **NON-ANTIGEN**).

>EHO9885495.1 phosphatidylglycerophosphatase B [Salmonella enterica subsp. enterica serovar Infantis] Overall Protective Antigen Prediction = **0.3622** (Probable **NON-ANTIGEN**).

>EHO9885496.1 LapA family protein [Salmonella enterica subsp. enterica serovar Infantis] Overall Protective Antigen Prediction = **0.8144** (Probable **ANTIGEN**).

>EHO9885497.1 lipopolysaccharide assembly protein LapB [Salmonella enterica subsp. enterica serovar Infantis] Overall Protective Antigen Prediction = **0.4730** (Probable **ANTIGEN**).

>EHO9885498.1 orotidine-5'-phosphate decarboxylase [Salmonella enterica subsp. enterica serovar Infantis] Overall Protective Antigen Prediction = **0.5496** (Probable **ANTIGEN**).

>EHO9885499.1 stress response translation initiation inhibitor YciH [Salmonella enterica subsp. enterica serovar Infantis] Overall Protective Antigen Prediction = **0.7227** (Probable **ANTIGEN**).

>EHO9885500.1 osmotically-inducible lipoprotein OsmB [Salmonella enterica subsp. enterica serovar Infantis] Overall Protective Antigen Prediction = **0.5976** (Probable **ANTIGEN**).

>EHO9885501.1 DeoR/GlpR transcriptional regulator [Salmonella enterica subsp. enterica serovar Infantis] Overall Protective Antigen Prediction = **0.2903** (Probable **NON-ANTIGEN**).

>EHO9885502.1 hypothetical protein KND05_000797 [Salmonella enterica subsp. enterica serovar Infantis] Overall Protective Antigen Prediction = **0.5010** (Probable **ANTIGEN**).

>EHO9885503.1 cyclic di-GMP phosphodiesterase [Salmonella enterica subsp. enterica serovar Infantis] Overall Protective Antigen Prediction = **0.3592** (Probable **NON-ANTIGEN**).

>EHO9885504.1 exoribonuclease II [Salmonella enterica subsp. enterica serovar Infantis] Overall Protective Antigen Prediction = **0.4040** (Probable **ANTIGEN**).

>EHO9885505.1 CMD domain-containing protein [Salmonella enterica subsp. enterica serovar Infantis] Overall Protective Antigen Prediction = **0.2982** (Probable **NON-ANTIGEN**).

>EHO9885506.1 enoyl-ACP reductase FabI [Salmonella enterica subsp. enterica serovar Infantis] Overall Protective Antigen Prediction = **0.4363** (Probable **ANTIGEN**).

>EHO9885507.1 hypothetical protein KND05_000802 [Salmonella enterica subsp. enterica serovar Infantis] Overall Protective Antigen Prediction = **0.5169** (Probable **ANTIGEN**).

>EHO9885508.1 hypothetical protein KND05_000803 [Salmonella enterica subsp. enterica serovar Infantis] Overall Protective Antigen Prediction = **0.4959** (Probable **ANTIGEN**).

>EHO9885509.1 SPI-2 type III secretion system effector kinase SteC [Salmonella enterica subsp. enterica serovar Infantis] Overall Protective Antigen Prediction = **0.4629** (Probable **ANTIGEN**).

>EHO9885510.1 EAL domain-containing protein [Salmonella enterica subsp. enterica serovar Infantis] Overall Protective Antigen Prediction = **0.2806** (Probable **NON-ANTIGEN**).

>EHO9885511.1 peptide ABC transporter ATP-binding protein SapF [Salmonella enterica subsp. enterica serovar Infantis] Overall Protective Antigen Prediction = **0.2833** (Probable **NON-ANTIGEN**).

>EHO9885512.1 peptide ABC transporter ATP-binding protein SapD [Salmonella enterica subsp. enterica serovar Infantis] Overall Protective Antigen Prediction = **0.4827** (Probable **ANTIGEN**).

>EHO9885513.1 peptide ABC transporter permease SapC [Salmonella enterica subsp. enterica serovar Infantis] Overall Protective Antigen Prediction = **0.4265** (Probable **ANTIGEN**).

>EHO9885514.1 peptide ABC transporter permease SapB [Salmonella enterica subsp. enterica serovar Infantis] Overall Protective Antigen Prediction = **0.4402** (Probable **ANTIGEN**).

>EHO9885515.1 peptide ABC transporter substrate-binding protein SapA [Salmonella enterica subsp. enterica serovar Infantis] Overall Protective Antigen Prediction = **0.5139** (Probable **ANTIGEN**).

>EHO9885516.1 phage shock protein operon transcriptional activator [Salmonella enterica subsp. enterica serovar Infantis] Overall Protective Antigen Prediction = **0.3592** (Probable **NON-ANTIGEN**).

>EHO9885517.1 phage shock protein PspA [Salmonella enterica subsp. enterica serovar Infantis] Overall Protective Antigen Prediction = **0.5420** (Probable **ANTIGEN**).

>EHO9885518.1 envelope stress response membrane protein PspB [Salmonella enterica subsp. enterica serovar Infantis] Overall Protective Antigen Prediction = **1.0551** (Probable **ANTIGEN**).

>EHO9885519.1 envelope stress response membrane protein PspC [Salmonella enterica subsp. enterica serovar Infantis] Overall Protective Antigen Prediction = **0.6099** (Probable **ANTIGEN**).

>EHO9885520.1 phage shock protein PspD [Salmonella enterica subsp. enterica serovar Infantis] Overall Protective Antigen Prediction = **0.6828** (Probable **ANTIGEN**).

>EHO9885521.1 thiosulfate sulfurtransferase PspE [Salmonella enterica subsp. enterica serovar Infantis] Overall Protective Antigen Prediction = **0.4792** (Probable **ANTIGEN**).

>EHO9885522.1 YcjX family protein [Salmonella enterica subsp. enterica serovar Infantis] Overall Protective Antigen Prediction = **0.3229** (Probable **NON-ANTIGEN**).

>EHO9885523.1 TIGR01620 family protein [Salmonella enterica subsp. enterica serovar Infantis] Overall Protective Antigen Prediction = **0.4032** (Probable **ANTIGEN**).

>EHO9885524.1 transcriptional regulator TyrR [Salmonella enterica subsp. enterica serovar Infantis] Overall Protective Antigen Prediction = **0.4841** (Probable **ANTIGEN**).

>EHO9885525.1 thiol peroxidase [Salmonella enterica subsp. enterica serovar Infantis] Overall Protective Antigen Prediction = **0.3041** (Probable **NON-ANTIGEN**).

>EHO9885526.1 L-Ala-D/L-Glu epimerase [Salmonella enterica subsp. enterica serovar Infantis] Overall Protective Antigen Prediction = **0.5204** (Probable **ANTIGEN**).

>EHO9885527.1 murein tripeptide amidase MpaA [Salmonella enterica subsp. enterica serovar Infantis] Overall Protective Antigen Prediction = **0.6112** (Probable **ANTIGEN**).

>EHO9885528.1 peptide ABC transporter substrate-binding protein [Salmonella enterica subsp. enterica serovar Infantis] Overall Protective Antigen Prediction = **0.4122** (Probable **ANTIGEN**).

>EHO9885529.1 aromatic alcohol reductase [Salmonella enterica subsp. enterica serovar Infantis]

Overall Protective Antigen Prediction = **0.5141** (Probable **ANTIGEN**).

>EHO9885530.1 LysR family transcriptional regulator [Salmonella enterica subsp. enterica serovar Infantis] Overall Protective Antigen Prediction = **0.2754** (Probable **NON-ANTIGEN**).

>EHO9885531.1 aldo/keto reductase [Salmonella enterica subsp. enterica serovar Infantis] Overall Protective Antigen Prediction = **0.4218** (Probable **ANTIGEN**).

>EHO9885532.1 SDR family oxidoreductase [Salmonella enterica subsp. enterica serovar Infantis] Overall Protective Antigen Prediction = **0.3406** (Probable **NON-ANTIGEN**).

>EHO9885533.1 TetR/AcrR family transcriptional regulator [Salmonella enterica subsp. enterica serovar Infantis] Overall Protective Antigen Prediction = **0.3944** (Probable **NON-ANTIGEN**).

>EHO9885534.1 YgdI/YgdR family lipoprotein [Salmonella enterica subsp. enterica serovar Infantis] Overall Protective Antigen Prediction = **0.5483** (Probable **ANTIGEN**).

>EHO9885535.1 hypothetical protein KND05_000830 [Salmonella enterica subsp. enterica serovar Infantis] Overall Protective Antigen Prediction = **0.3864** (Probable **NON-ANTIGEN**).

>EHO9885536.1 helix-turn-helix domain-containing protein [Salmonella enterica subsp. enterica serovar Infantis] Overall Protective Antigen Prediction = **0.4331** (Probable **ANTIGEN**).

>EHO9885537.1 hypothetical protein KND05_000832 [Salmonella enterica subsp. enterica serovar Infantis] Overall Protective Antigen Prediction = **0.8341** (Probable **ANTIGEN**).

>EHO9885538.1 two-partner secretion translocator ZirT [Salmonella enterica subsp. enterica serovar Infantis] Overall Protective Antigen Prediction = **0.7290** (Probable **ANTIGEN**).

>EHO9885539.1 hypothetical protein KND05_000834 [Salmonella enterica subsp. enterica serovar Infantis] Overall Protective Antigen Prediction = **0.6280** (Probable **ANTIGEN**).

>EHO9885540.1 peroxiredoxin [Salmonella enterica subsp. enterica serovar Infantis] Overall Protective Antigen Prediction = **0.3500** (Probable **NON-ANTIGEN**).

>EHO9885541.1 hypothetical protein KND05_000836 [Salmonella enterica subsp. enterica serovar Infantis] Overall Protective Antigen Prediction = **0.4363** (Probable **ANTIGEN**).

>EHO9885542.1 B3/4 domain-containing protein [Salmonella enterica subsp. enterica serovar Infantis] Overall Protective Antigen Prediction = **0.5511** (Probable **ANTIGEN**).

>EHO9885543.1 helix-turn-helix domain-containing protein [Salmonella enterica subsp. enterica serovar Infantis] Overall Protective Antigen Prediction = **0.5147** (Probable **ANTIGEN**).

>EHO9885544.1 mechanosensitive ion channel family protein [Salmonella enterica subsp. enterica serovar Infantis] Overall Protective Antigen Prediction = **0.3768** (Probable **NON-ANTIGEN**).

>EHO9885545.1 DUF2534 family protein [Salmonella enterica subsp. enterica serovar Infantis] Overall Protective Antigen Prediction = **0.7380** (Probable **ANTIGEN**).

>EHO9885546.1 universal stress protein UspE [Salmonella enterica subsp. enterica serovar Infantis] Overall Protective Antigen Prediction = **0.4257** (Probable **ANTIGEN**).

>EHO9885547.1 fumarate/nitrate reduction transcriptional regulator Fnr [Salmonella enterica subsp.

enterica serovar Infantis] Overall Protective Antigen Prediction = **0.4142** (Probable **ANTIGEN**).

>EHO9885548.1 methylated-DNA--[protein]-cysteine S-methyltransferase [Salmonella enterica subsp. enterica serovar Infantis] Overall Protective Antigen Prediction = **0.4239** (Probable **ANTIGEN**).

>EHO9885549.1 DNA endonuclease SmrA [Salmonella enterica subsp. enterica serovar Infantis] Overall Protective Antigen Prediction = **0.4638** (Probable **ANTIGEN**).

>EHO9885550.1 chemoreceptor protein [Salmonella enterica subsp. enterica serovar Infantis] Overall Protective Antigen Prediction = **0.6838** (Probable **ANTIGEN**).

>EHO9885551.1 zinc transporter ZntB [Salmonella enterica subsp. enterica serovar Infantis] Overall Protective Antigen Prediction = **0.4082** (Probable **ANTIGEN**).

>EHO9885552.1 ATP-dependent RNA helicase DbpA [Salmonella enterica subsp. enterica serovar Infantis] Overall Protective Antigen Prediction = **0.5374** (Probable **ANTIGEN**).

>EHO9885553.1 tRNA 2-thiocytidine(32) synthetase TtcA [Salmonella enterica subsp. enterica serovar Infantis] Overall Protective Antigen Prediction = **0.4128** (Probable **ANTIGEN**).

>EHO9885554.1 EamA family transporter [Salmonella enterica subsp. enterica serovar Infantis] Overall Protective Antigen Prediction = **0.5515** (Probable **ANTIGEN**).

>EHO9885555.1 universal stress protein UspF [Salmonella enterica subsp. enterica serovar Infantis] Overall Protective Antigen Prediction = **0.5109** (Probable **ANTIGEN**).

>EHO9885556.1 pyruvate:ferredoxin (flavodoxin) oxidoreductase [Salmonella enterica subsp. enterica serovar Infantis] Overall Protective Antigen Prediction = **0.5044** (Probable **ANTIGEN**).

>EHO9885557.1 reverse transcriptase [Salmonella enterica subsp. enterica serovar Infantis] Overall Protective Antigen Prediction = **0.5268** (Probable **ANTIGEN**).

>EHO9885558.1 DUF333 domain-containing protein [Salmonella enterica subsp. enterica serovar Infantis] Overall Protective Antigen Prediction = **0.6263** (Probable **ANTIGEN**).

>EHO9885559.1 heat shock protein HslJ [Salmonella enterica subsp. enterica serovar Infantis] Overall Protective Antigen Prediction = **0.7977** (Probable **ANTIGEN**).

>EHO9885560.1 D-lactate dehydrogenase [Salmonella enterica subsp. enterica serovar Infantis] Overall Protective Antigen Prediction = **0.4179** (Probable **ANTIGEN**).

>EHO9885561.1 YdbH family protein [Salmonella enterica subsp. enterica serovar Infantis] Overall Protective Antigen Prediction = **0.5207** (Probable **ANTIGEN**).

>EHO9885562.1 YnbE family lipoprotein [Salmonella enterica subsp. enterica serovar Infantis] Overall Protective Antigen Prediction = **0.8173** (Probable **ANTIGEN**).

>EHO9885563.1 YdbL family protein [Salmonella enterica subsp. enterica serovar Infantis] Overall Protective Antigen Prediction = **0.7045** (Probable **ANTIGEN**).

>EHO9885564.1 GFA family protein [Salmonella enterica subsp. enterica serovar Infantis] Overall Protective Antigen Prediction = **0.6542** (Probable **ANTIGEN**).

>EHO9885565.1 FMN-dependent NADH-azoreductase [Salmonella enterica subsp. enterica serovar Infantis] Overall Protective Antigen Prediction = **0.4956** (Probable **ANTIGEN**).

>EHO9885566.1 ATP-dependent RNA helicase HrpA [Salmonella enterica subsp. enterica serovar Infantis] Overall Protective Antigen Prediction = **0.4455** (Probable **ANTIGEN**).

>EHO9885567.1 YdcF family protein [Salmonella enterica subsp. enterica serovar Infantis] Overall Protective Antigen Prediction = **0.3564** (Probable **NON-ANTIGEN**).

>EHO9885568.1 cytochrome b561 [Salmonella enterica subsp. enterica serovar Infantis] Overall Protective Antigen Prediction = **0.6100** (Probable **ANTIGEN**).

>EHO9885569.1 class I SAM-dependent methyltransferase [Salmonella enterica subsp. enterica serovar Infantis] Overall Protective Antigen Prediction = **0.6425** (Probable **ANTIGEN**).

>EHO9885570.1 glycosyltransferase family 39 protein [Salmonella enterica subsp. enterica serovar Infantis] Overall Protective Antigen Prediction = **0.5505** (Probable **ANTIGEN**).

>EHO9885571.1 amino acid ABC transporter permease [Salmonella enterica subsp. enterica serovar Infantis] Overall Protective Antigen Prediction = **0.4940** (Probable **ANTIGEN**).

>EHO9885572.1 amino acid ABC transporter ATP-binding protein [Salmonella enterica subsp. enterica serovar Infantis] Overall Protective Antigen Prediction = **0.3344** (Probable **NON-ANTIGEN**).

>EHO9885573.1 amino acid ABC transporter permease [Salmonella enterica subsp. enterica serovar Infantis] Overall Protective Antigen Prediction = **0.4520** (Probable **ANTIGEN**).

>EHO9885574.1 transporter substrate-binding domain-containing protein [Salmonella enterica subsp. enterica serovar Infantis] Overall Protective Antigen Prediction = **0.4034** (Probable **ANTIGEN**).

>EHO9885575.1 DUF1294 domain-containing protein [Salmonella enterica subsp. enterica serovar Infantis] Overall Protective Antigen Prediction = **0.4637** (Probable **ANTIGEN**).

>EHO9885576.1 SPI-2 type III secretion system effector SseJ [Salmonella enterica subsp. enterica serovar Infantis] Overall Protective Antigen Prediction = **0.3480** (Probable **NON-ANTIGEN**).

>EHO9885577.1 hypothetical protein KND05_000873 [Salmonella enterica subsp. enterica serovar Infantis] Overall Protective Antigen Prediction = **0.7999** (Probable **ANTIGEN**).

>EHO9885578.1 type III secretion system effector SteB [Salmonella enterica subsp. enterica serovar Infantis] Overall Protective Antigen Prediction = **0.4323** (Probable **ANTIGEN**).

>EHO9885579.1 metal/formaldehyde-sensitive transcriptional repressor [Salmonella enterica subsp. enterica serovar Infantis] Overall Protective Antigen Prediction = **0.3602** (Probable **NON-ANTIGEN**).

>EHO9885580.1 S-(hydroxymethyl)glutathione dehydrogenase/class III alcohol dehydrogenase [Salmonella enterica subsp. enterica serovar Infantis] Overall Protective Antigen Prediction = **0.5300** (Probable **ANTIGEN**).

>EHO9885581.1 Tar ligand binding domain-containing protein [Salmonella enterica subsp. enterica serovar Infantis] Overall Protective Antigen Prediction = **0.5874** (Probable **ANTIGEN**).

>EHO9885582.1 LysR family transcriptional regulator [Salmonella enterica subsp. enterica serovar Infantis] Overall Protective Antigen Prediction = **0.3606** (Probable **NON-ANTIGEN**).

>EHO9885583.1 VOC family protein [Salmonella enterica subsp. enterica serovar Infantis] Overall Protective Antigen Prediction = **0.3680** (Probable **NON-ANTIGEN**).

>EHO9885584.1 carboxylesterase/lipase family protein [Salmonella enterica subsp. enterica serovar Infantis] Overall Protective Antigen Prediction = **0.3725** (Probable **NON-ANTIGEN**).

>EHO9885585.1 glucan biosynthesis protein [Salmonella enterica subsp. enterica serovar Infantis] Overall Protective Antigen Prediction = **0.4723** (Probable **ANTIGEN**).

>EHO9885586.1 polyisoprenoid-binding protein [Salmonella enterica subsp. enterica serovar Infantis] Overall Protective Antigen Prediction = **0.4712** (Probable **ANTIGEN**).

>EHO9885587.1 alpha-hydroxy-acid oxidizing protein [Salmonella enterica subsp. enterica serovar Infantis] Overall Protective Antigen Prediction = **0.5037** (Probable **ANTIGEN**).

>EHO9885588.1 cryptic aminoglycoside N-acetyltransferase AAC(6')-Iy/Iaa [Salmonella enterica subsp. enterica serovar Infantis] Overall Protective Antigen Prediction = **0.3014** (Probable **NON-ANTIGEN**).

>EHO9885589.1 DeoR/GlpR transcriptional regulator [Salmonella enterica subsp. enterica serovar Infantis] Overall Protective Antigen Prediction = **0.2799** (Probable **NON-ANTIGEN**).

>EHO9885590.1 ribulose-phosphate 3 epimerase family protein [Salmonella enterica subsp. enterica serovar Infantis] Overall Protective Antigen Prediction = **0.5272** (Probable **ANTIGEN**).

>EHO9885591.1 PTS sugar transporter subunit IIA [Salmonella enterica subsp. enterica serovar Infantis] Overall Protective Antigen Prediction = **0.3992** (Probable **NON-ANTIGEN**).

>EHO9885592.1 BtpA family protein SgcQ [Salmonella enterica subsp. enterica serovar Infantis] Overall Protective Antigen Prediction = **0.2536** (Probable **NON-ANTIGEN**).

>EHO9885593.1 permease [Salmonella enterica subsp. enterica serovar Infantis] Overall Protective Antigen Prediction = **0.4618** (Probable **ANTIGEN**).

>EHO9885594.1 PTS sugar transporter subunit IIB [Salmonella enterica subsp. enterica serovar Infantis] Overall Protective Antigen Prediction = **0.3369** (Probable **NON-ANTIGEN**).

>EHO9885595.1 M42 family metallopeptidase [Salmonella enterica subsp. enterica serovar Infantis] Overall Protective Antigen Prediction = **0.5021** (Probable **ANTIGEN**).

>EHO9885596.1 50S ribosomal protein L7/L12-serine acetyltransferase [Salmonella enterica subsp. enterica serovar Infantis] Overall Protective Antigen Prediction = **0.4261** (Probable **ANTIGEN**).

>EHO9885597.1 hypothetical protein KND05_000893 [Salmonella enterica subsp. enterica serovar Infantis] Overall Protective Antigen Prediction = **0.5395** (Probable **ANTIGEN**).

>EHO9885598.1 dicarboxylate transporter/tellurite-resistance protein TehA [Salmonella enterica subsp. enterica serovar Infantis] Overall Protective Antigen Prediction = **0.5461** (Probable **ANTIGEN**).

>EHO9885599.1 tellurite resistance methyltransferase TehB [Salmonella enterica subsp. enterica

serovar Infantis] Overall Protective Antigen Prediction = **0.6065** (Probable **ANTIGEN**).

>EHO9885600.1 DUF3313 domain-containing protein [Salmonella enterica subsp. enterica serovar Infantis] Overall Protective Antigen Prediction = **0.3481** (Probable **NON-ANTIGEN**).

>EHO9885601.1 benzoate/H(+) symporter BenE family transporter [Salmonella enterica subsp. enterica serovar Infantis] Overall Protective Antigen Prediction = **0.4166** (Probable **ANTIGEN**).

>EHO9885602.1 helix-turn-helix transcriptional regulator [Salmonella enterica subsp. enterica serovar Infantis] Overall Protective Antigen Prediction = **0.4304** (Probable **ANTIGEN**).

>EHO9885603.1 U32 family peptidase [Salmonella enterica subsp. enterica serovar Infantis] Overall Protective Antigen Prediction = **0.4029** (Probable **ANTIGEN**).

>EHO9885604.1 DUF2554 family protein [Salmonella enterica subsp. enterica serovar Infantis] Overall Protective Antigen Prediction = **0.5338** (Probable **ANTIGEN**).

>EHO9885605.1 SPI-2 type III secretion system effector SifB [Salmonella enterica subsp. enterica serovar Infantis] Overall Protective Antigen Prediction = **0.4234** (Probable **ANTIGEN**).

>EHO9885606.1 D-alanyl-D-alanine dipeptidase [Salmonella enterica subsp. enterica serovar Infantis] Overall Protective Antigen Prediction = **0.6642** (Probable **ANTIGEN**).

>EHO9885607.1 hypothetical protein KND05_000903 [Salmonella enterica subsp. enterica serovar Infantis] Overall Protective Antigen Prediction = **0.4019** (Probable **ANTIGEN**).

>EHO9885608.1 M15 family metallopeptidase [Salmonella enterica subsp. enterica serovar Infantis] Overall Protective Antigen Prediction = **0.3401** (Probable **NON-ANTIGEN**).

>EHO9885609.1 PLP-dependent aminotransferase family protein [Salmonella enterica subsp. enterica serovar Infantis] Overall Protective Antigen Prediction = **0.3330** (Probable **NON-ANTIGEN**).

>EHO9885610.1 aminobutyraldehyde dehydrogenase [Salmonella enterica subsp. enterica serovar Infantis] Overall Protective Antigen Prediction = **0.4950** (Probable **ANTIGEN**).

>EHO9885611.1 stress response membrane protein YncL [Salmonella enterica subsp. enterica serovar Infantis] Overall Protective Antigen Prediction = **0.6043** (Probable **ANTIGEN**).

>EHO9885612.1 GhoT/OrtT family toxin [Salmonella enterica subsp. enterica serovar Infantis] Overall Protective Antigen Prediction = **0.5232** (Probable **ANTIGEN**).

>EHO9885613.1 virulence effector SrfC [Salmonella enterica subsp. enterica serovar Infantis] Overall Protective Antigen Prediction = **0.3543** (Probable **NON-ANTIGEN**).

>EHO9885614.1 virulence factor SrfB [Salmonella enterica subsp. enterica serovar Infantis] Overall Protective Antigen Prediction = **0.5083** (Probable **ANTIGEN**).

>EHO9885615.1 ssrAB-activated protein [Salmonella enterica subsp. enterica serovar Infantis] Overall Protective Antigen Prediction = **0.5097** (Probable **ANTIGEN**).

>EHO9885616.1 DUF2526 family protein [Salmonella enterica subsp. enterica serovar Infantis] Overall Protective Antigen Prediction = **0.6270** (Probable **ANTIGEN**).

>EHO9885617.1 DMT family transporter [Salmonella enterica subsp. enterica serovar Infantis]

Overall Protective Antigen Prediction = **0.4316** (Probable **ANTIGEN**).

>EHO9885618.1 L-methionine sulfoximine/L-methionine sulfone acetyltransferase [Salmonella enterica subsp. enterica serovar Infantis] Overall Protective Antigen Prediction = **0.4828** (Probable **ANTIGEN**).

>EHO9885619.1 NADP-dependent oxidoreductase [Salmonella enterica subsp. enterica serovar Infantis] Overall Protective Antigen Prediction = **0.4081** (Probable **ANTIGEN**).

>EHO9885620.1 colanic acid/biofilm transcriptional regulator McbR [Salmonella enterica subsp. enterica serovar Infantis] Overall Protective Antigen Prediction = **0.4310** (Probable **ANTIGEN**).

>EHO9885621.1 TonB-dependent receptor [Salmonella enterica subsp. enterica serovar Infantis] Overall Protective Antigen Prediction = **0.7394** (Probable **ANTIGEN**).

>EHO9885622.1 YncE family protein [Salmonella enterica subsp. enterica serovar Infantis] Overall Protective Antigen Prediction = **0.5374** (Probable **ANTIGEN**).

>EHO9885623.1 YgdI/YgdR family lipoprotein [Salmonella enterica subsp. enterica serovar Infantis] Overall Protective Antigen Prediction = **0.6092** (Probable **ANTIGEN**).

>EHO9885624.1 L-asparagine permease [Salmonella enterica subsp. enterica serovar Infantis] Overall Protective Antigen Prediction = **0.4140** (Probable **ANTIGEN**).

>EHO9885625.1 effector protein steA [Salmonella enterica subsp. enterica serovar Infantis] Overall Protective Antigen Prediction = **0.4543** (Probable **ANTIGEN**).

>EHO9885626.1 N-hydroxyarylamine O-acetyltransferase [Salmonella enterica subsp. enterica serovar Infantis] Overall Protective Antigen Prediction = **0.4352** (Probable **ANTIGEN**).

>EHO9885627.1 PhzF family isomerase [Salmonella enterica subsp. enterica serovar Infantis] Overall Protective Antigen Prediction = **0.7065** (Probable **ANTIGEN**).

>EHO9885628.1 respiratory nitrate reductase subunit gamma [Salmonella enterica subsp. enterica serovar Infantis] Overall Protective Antigen Prediction = **0.5946** (Probable **ANTIGEN**).

>EHO9885629.1 nitrate reductase molybdenum cofactor assembly chaperone [Salmonella enterica subsp. enterica serovar Infantis] Overall Protective Antigen Prediction = **0.2568** (Probable **NON-ANTIGEN**).

>EHO9885630.1 nitrate reductase subunit beta [Salmonella enterica subsp. enterica serovar Infantis] Overall Protective Antigen Prediction = **0.5023** (Probable **ANTIGEN**).

>EHO9885631.1 nitrate reductase subunit alpha [Salmonella enterica subsp. enterica serovar Infantis] Overall Protective Antigen Prediction = **0.4840** (Probable **ANTIGEN**).

>EHO9885632.1 NarK family nitrate/nitrite MFS transporter [Salmonella enterica subsp. enterica serovar Infantis] Overall Protective Antigen Prediction = **0.6072** (Probable **ANTIGEN**).

>EHO9885633.1 TetR family transcriptional regulator [Salmonella enterica subsp. enterica serovar Infantis] Overall Protective Antigen Prediction = **0.3717** (Probable **NON-ANTIGEN**).

>EHO9885634.1 methyl viologen efflux MFS transporter SmvA [Salmonella enterica subsp. enterica serovar Infantis] Overall Protective Antigen Prediction = **0.4632** (Probable **ANTIGEN**).

>EHO9885635.1 GFA family protein [Salmonella enterica subsp. enterica serovar Infantis] Overall Protective Antigen Prediction = **0.5833** (Probable **ANTIGEN**).

>EHO9885636.1 porin OmpD [Salmonella enterica subsp. enterica serovar Infantis] Overall Protective Antigen Prediction = **0.6635** (Probable **ANTIGEN**).

>EHO9885637.1 aromatic amino acid efflux DMT transporter YddG [Salmonella enterica subsp. enterica serovar Infantis] Overall Protective Antigen Prediction = **0.3599** (Probable **NON-ANTIGEN**).

>EHO9885638.1 formate dehydrogenase-N subunit alpha [Salmonella enterica subsp. enterica serovar Infantis] Overall Protective Antigen Prediction = **0.4388** (Probable **ANTIGEN**).

>EHO9885639.1 formate dehydrogenase subunit beta [Salmonella enterica subsp. enterica serovar Infantis] Overall Protective Antigen Prediction = **0.4829** (Probable **ANTIGEN**).

>EHO9885640.1 formate dehydrogenase-N subunit gamma [Salmonella enterica subsp. enterica serovar Infantis] Overall Protective Antigen Prediction = **0.6714** (Probable **ANTIGEN**).

>EHO9885641.1 alcohol dehydrogenase AdhP [Salmonella enterica subsp. enterica serovar Infantis] Overall Protective Antigen Prediction = **0.6564** (Probable **ANTIGEN**).

>EHO9885642.1 NAD-dependent malic enzyme [Salmonella enterica subsp. enterica serovar Infantis] Overall Protective Antigen Prediction = **0.3350** (Probable **NON-ANTIGEN**).

>EHO9885643.1 stationary-phase-induced ribosome-associated protein [Salmonella enterica subsp. enterica serovar Infantis] Overall Protective Antigen Prediction = **1.1722** (Probable **ANTIGEN**).

>EHO9885644.1 biofilm-dependent modulation protein [Salmonella enterica subsp. enterica serovar Infantis] Overall Protective Antigen Prediction = **0.4096** (Probable **ANTIGEN**).

>EHO9885645.1 OsmC family protein [Salmonella enterica subsp. enterica serovar Infantis] Overall Protective Antigen Prediction = **0.7034** (Probable **ANTIGEN**).

>EHO9885646.1 acid-activated periplasmic chaperone HdeB [Salmonella enterica subsp. enterica serovar Infantis] Overall Protective Antigen Prediction = **0.6636** (Probable **ANTIGEN**).

>EHO9885647.1 OBAP family protein [Salmonella enterica subsp. enterica serovar Infantis] Overall Protective Antigen Prediction = **0.6301** (Probable **ANTIGEN**).

>EHO9885648.1 malto-oligosyltrehalose trehalohydrolase [Salmonella enterica subsp. enterica serovar Infantis] Overall Protective Antigen Prediction = **0.4566** (Probable **ANTIGEN**).

>EHO9885649.1 malto-oligosyltrehalose synthase [Salmonella enterica subsp. enterica serovar Infantis] Overall Protective Antigen Prediction = **0.3829** (Probable **NON-ANTIGEN**).

>EHO9885650.1 glycogen debranching protein GlgX [Salmonella enterica subsp. enterica serovar Infantis] Overall Protective Antigen Prediction = **0.4903** (Probable **ANTIGEN**).

>EHO9885651.1 pyridoxal phosphate-dependent aminotransferase [Salmonella enterica subsp. enterica serovar Infantis] Overall Protective Antigen Prediction = **0.3492** (Probable **NON-ANTIGEN**).

>EHO9885652.1 Na+/H+ antiporter NhaC [Salmonella enterica subsp. enterica serovar Infantis] Overall Protective Antigen Prediction = **0.4159** (Probable **ANTIGEN**).

>EHO9885653.1 LacI family DNA-binding transcriptional regulator [Salmonella enterica subsp. enterica serovar Infantis] Overall Protective Antigen Prediction = **0.3553** (Probable **NON-ANTIGEN**).

>EHO9885654.1 hypothetical protein KND05_000950 [Salmonella enterica subsp. enterica serovar Infantis] Overall Protective Antigen Prediction = **0.2861** (Probable **NON-ANTIGEN**).

>EHO9885655.1 nitronate monooxygenase [Salmonella enterica subsp. enterica serovar Infantis] Overall Protective Antigen Prediction = **0.4421** (Probable **ANTIGEN**).

>EHO9885656.1 LysR family transcriptional regulator [Salmonella enterica subsp. enterica serovar Infantis] Overall Protective Antigen Prediction = **0.2674** (Probable **NON-ANTIGEN**).

>EHO9885657.1 DUF1493 family protein [Salmonella enterica subsp. enterica serovar Infantis] Overall Protective Antigen Prediction = **0.2086** (Probable **NON-ANTIGEN**).

>EHO9885658.1 hypothetical protein KND05_000956 [Salmonella enterica subsp. enterica serovar Infantis] Overall Protective Antigen Prediction = **0.2514** (Probable **NON-ANTIGEN**).

>EHO9885659.1 type II toxin-antitoxin system Phd/YefM family antitoxin [Salmonella enterica subsp. enterica serovar Infantis] Overall Protective Antigen Prediction = **0.5264** (Probable **ANTIGEN**).

>EHO9885660.1 type II toxin-antitoxin system RelE/ParE family toxin [Salmonella enterica subsp. enterica serovar Infantis] Overall Protective Antigen Prediction = **0.6539** (Probable **ANTIGEN**).

>EHO9885661.1 RidA family protein [Salmonella enterica subsp. enterica serovar Infantis] Overall Protective Antigen Prediction = **0.5505** (Probable **ANTIGEN**).

>EHO9885662.1 S-adenosylmethionine:tRNA ribosyltransferase-isomerase [Salmonella enterica subsp. enterica serovar Infantis] Overall Protective Antigen Prediction = **0.4387** (Probable **ANTIGEN**).

>EHO9885663.1 winged helix-turn-helix transcriptional regulator [Salmonella enterica subsp. enterica serovar Infantis] Overall Protective Antigen Prediction = **0.3229** (Probable **NON-ANTIGEN**).

>EHO9885664.1 FAD-dependent oxidoreductase [Salmonella enterica subsp. enterica serovar Infantis] Overall Protective Antigen Prediction = **0.5038** (Probable **ANTIGEN**).

>EHO9885665.1 MFS transporter [Salmonella enterica subsp. enterica serovar Infantis] Overall Protective Antigen Prediction = **0.4304** (Probable **ANTIGEN**).

>EHO9885666.1 PhoPQ-activated pathogenicity-related family protein [Salmonella enterica subsp. enterica serovar Infantis] Overall Protective Antigen Prediction = **0.4543** (Probable **ANTIGEN**).

>EHO9885667.1 MFS transporter [Salmonella enterica subsp. enterica serovar Infantis] Overall Protective Antigen Prediction = **0.6340** (Probable **ANTIGEN**).

>EHO9885668.1 zinc-binding alcohol dehydrogenase family protein [Salmonella enterica subsp. enterica serovar Infantis] Overall Protective Antigen Prediction = **0.4391** (Probable **ANTIGEN**).

>EHO9885669.1 FadR family transcriptional regulator [Salmonella enterica subsp. enterica serovar Infantis] Overall Protective Antigen Prediction = **0.3407** (Probable **NON-ANTIGEN**).

>EHO9885670.1 linear amide C-N hydrolase [Salmonella enterica subsp. enterica serovar Infantis] Overall Protective Antigen Prediction = **0.4205** (Probable **ANTIGEN**).

>EHO9885671.1 hydrogenase small subunit [Salmonella enterica subsp. enterica serovar Infantis] Overall Protective Antigen Prediction = **0.3522** (Probable **NON-ANTIGEN**).

>EHO9885672.1 nickel-dependent hydrogenase large subunit [Salmonella enterica subsp. enterica serovar Infantis] Overall Protective Antigen Prediction = **0.2585** (Probable **NON-ANTIGEN**).

>EHO9885673.1 Ni/Fe-hydrogenase, b-type cytochrome subunit [Salmonella enterica subsp. enterica serovar Infantis] Overall Protective Antigen Prediction = **0.6917** (Probable **ANTIGEN**).

>EHO9885674.1 HyaD/HybD family hydrogenase maturation endopeptidase [Salmonella enterica subsp. enterica serovar Infantis] Overall Protective Antigen Prediction = **0.4118** (Probable **ANTIGEN**).

>EHO9885675.1 HypC/HybG/HupF family hydrogenase formation chaperone [Salmonella enterica subsp. enterica serovar Infantis] Overall Protective Antigen Prediction = **0.4628** (Probable **ANTIGEN**).

>EHO9885676.1 hydrogenase [Salmonella enterica subsp. enterica serovar Infantis] Overall Protective Antigen Prediction = **0.2276** (Probable **NON-ANTIGEN**).

>EHO9885677.1 rubredoxin [Salmonella enterica subsp. enterica serovar Infantis] Overall Protective Antigen Prediction = **0.4379** (Probable **ANTIGEN**).

>EHO9885678.1 ATP/GTP-binding protein [Salmonella enterica subsp. enterica serovar Infantis] Overall Protective Antigen Prediction = **0.3500** (Probable **NON-ANTIGEN**).

>EHO9885679.1 hydrogenase maturation nickel metallochaperone HypA [Salmonella enterica subsp. enterica serovar Infantis] Overall Protective Antigen Prediction = **0.5810** (Probable **ANTIGEN**).

>EHO9885680.1 porin OmpC [Salmonella enterica subsp. enterica serovar Infantis] Overall Protective Antigen Prediction = **0.5190** (Probable **ANTIGEN**).

>EHO9885681.1 nuclear transport factor 2 family protein [Salmonella enterica subsp. enterica serovar Infantis] Overall Protective Antigen Prediction = **0.7162** (Probable **ANTIGEN**).

>EHO9885682.1 bestrophin family protein [Salmonella enterica subsp. enterica serovar Infantis] Overall Protective Antigen Prediction = **0.5496** (Probable **ANTIGEN**).

>EHO9885683.1 DUF4186 domain-containing protein [Salmonella enterica subsp. enterica serovar Infantis] Overall Protective Antigen Prediction = **0.3778** (Probable **NON-ANTIGEN**).

>EHO9885684.1 glutaminase B [Salmonella enterica subsp. enterica serovar Infantis] Overall Protective Antigen Prediction = **0.3514** (Probable **NON-ANTIGEN**).

>EHO9885685.1 succinate-semialdehyde dehydrogenase [Salmonella enterica subsp. enterica serovar Infantis] Overall Protective Antigen Prediction = **0.4466** (Probable **ANTIGEN**).

>EHO9885686.1 LysR family transcriptional regulator [Salmonella enterica subsp. enterica serovar

Infantis] Overall Protective Antigen Prediction = **0.4669** (Probable **ANTIGEN**).

>EHO9885687.1 sugar transporter [Salmonella enterica subsp. enterica serovar Infantis] Overall Protective Antigen Prediction = **0.5501** (Probable **ANTIGEN**).

>EHO9885688.1 MarC family NAAT transporter [Salmonella enterica subsp. enterica serovar Infantis] Overall Protective Antigen Prediction = **0.4759** (Probable **ANTIGEN**).

>EHO9885689.1 multiple antibiotic resistance transcriptional regulator MarR [Salmonella enterica subsp. enterica serovar Infantis] Overall Protective Antigen Prediction = **0.2228** (Probable **NON-ANTIGEN**).

>EHO9885690.1 MDR efflux pump AcrAB transcriptional activator MarA [Salmonella enterica subsp. enterica serovar Infantis] Overall Protective Antigen Prediction = **0.3140** (Probable **NON-ANTIGEN**).

>EHO9885691.1 multiple antibiotic resistance protein MarB [Salmonella enterica subsp. enterica serovar Infantis] Overall Protective Antigen Prediction = **0.3088** (Probable **NON-ANTIGEN**).

>EHO9885692.1 O-acetylserine/cysteine exporter [Salmonella enterica subsp. enterica serovar Infantis] Overall Protective Antigen Prediction = **0.5850** (Probable **ANTIGEN**).

>EHO9885693.1 efflux MFS transporter YdeE [Salmonella enterica subsp. enterica serovar Infantis] Overall Protective Antigen Prediction = **0.4492** (Probable **ANTIGEN**).

>EHO9885694.1 protein MgtS [Salmonella enterica subsp. enterica serovar Infantis] Overall Protective Antigen Prediction = **0.3736** (Probable **NON-ANTIGEN**).

>EHO9885695.1 YdeI family stress tolerance OB fold protein [Salmonella enterica subsp. enterica serovar Infantis] Overall Protective Antigen Prediction = **0.7796** (Probable **ANTIGEN**).

>EHO9885696.1 2-oxo-tetronate isomerase [Salmonella enterica subsp. enterica serovar Infantis] Overall Protective Antigen Prediction = **0.6418** (Probable **ANTIGEN**).

>EHO9885697.1 general stress protein [Salmonella enterica subsp. enterica serovar Infantis] Overall Protective Antigen Prediction = **1.7377** (Probable **ANTIGEN**).

>EHO9885698.1 peptidyl-dipeptidase Dcp [Salmonella enterica subsp. enterica serovar Infantis] Overall Protective Antigen Prediction = **0.3864** (Probable **NON-ANTIGEN**).

>EHO9885699.1 bifunctional NADP-dependent 3-hydroxy acid dehydrogenase/3-hydroxypropionate dehydrogenase YdfG [Salmonella enterica subsp. enterica serovar Infantis] Overall Protective Antigen Prediction = **0.5248** (Probable **ANTIGEN**).

>EHO9885700.1 GntR family transcriptional regulator [Salmonella enterica subsp. enterica serovar Infantis] Overall Protective Antigen Prediction = **0.3181** (Probable **NON-ANTIGEN**).

>EHO9885701.1 putative selenium delivery protein YdfZ [Salmonella enterica subsp. enterica serovar Infantis] Overall Protective Antigen Prediction = **0.5218** (Probable **ANTIGEN**).

>EHO9885702.1 mannitol dehydrogenase family protein [Salmonella enterica subsp. enterica serovar Infantis] Overall Protective Antigen Prediction = **0.3362** (Probable **NON-ANTIGEN**).

>EHO9885703.1 MHS family MFS transporter [Salmonella enterica subsp. enterica serovar Infantis]

Overall Protective Antigen Prediction = **0.4992** (Probable **ANTIGEN**).

>EHO9885704.1 Zn-dependent oxidoreductase [Salmonella enterica subsp. enterica serovar Infantis] Overall Protective Antigen Prediction = **0.3754** (Probable **NON-ANTIGEN**).

>EHO9885705.1 starvation-sensing protein RspA [Salmonella enterica subsp. enterica serovar Infantis] Overall Protective Antigen Prediction = **0.4384** (Probable **ANTIGEN**).

>EHO9885706.1 YnfA family protein [Salmonella enterica subsp. enterica serovar Infantis] Overall Protective Antigen Prediction = **0.6678** (Probable **ANTIGEN**).

>EHO9885707.1 DUF1283 family protein [Salmonella enterica subsp. enterica serovar Infantis] Overall Protective Antigen Prediction = **0.5776** (Probable **ANTIGEN**).

>EHO9885708.1 spermidine N1-acetyltransferase [Salmonella enterica subsp. enterica serovar Infantis] Overall Protective Antigen Prediction = **0.6276** (Probable **ANTIGEN**).

>EHO9885709.1 YnfC family lipoprotein [Salmonella enterica subsp. enterica serovar Infantis] Overall Protective Antigen Prediction = **0.6409** (Probable **ANTIGEN**).

>EHO9885710.1 DUF1161 domain-containing protein [Salmonella enterica subsp. enterica serovar Infantis] Overall Protective Antigen Prediction = **0.6680** (Probable **ANTIGEN**).

>EHO9885711.1 dimethyl sulfoxide reductase subunit A [Salmonella enterica subsp. enterica serovar Infantis] Overall Protective Antigen Prediction = **0.5432** (Probable **ANTIGEN**).

>EHO9885712.1 dimethyl sulfoxide reductase subunit A [Salmonella enterica subsp. enterica serovar Infantis] Overall Protective Antigen Prediction = **0.5583** (Probable **ANTIGEN**).

>EHO9885713.1 dimethylsulfoxide reductase subunit B, partial [Salmonella enterica subsp. enterica serovar Infantis] Overall Protective Antigen Prediction = **0.4931** (Probable **ANTIGEN**).

>EHO9885714.1 Fe(3+)-hydroxamate ABC transporter permease FhuB [Salmonella enterica subsp. enterica serovar Infantis] Overall Protective Antigen Prediction = **0.4405** (Probable **ANTIGEN**).

>EHO9885715.1 Fe(3+)-hydroxamate ABC transporter substrate-binding protein FhuD [Salmonella enterica subsp. enterica serovar Infantis] Overall Protective Antigen Prediction = **0.3582** (Probable **NON-ANTIGEN**).

>EHO9885716.1 Fe3+-hydroxamate ABC transporter ATP-binding protein FhuC [Salmonella enterica subsp. enterica serovar Infantis] Overall Protective Antigen Prediction = **0.2600** (Probable **NON-ANTIGEN**).

>EHO9885717.1 ferrichrome porin FhuA [Salmonella enterica subsp. enterica serovar Infantis] Overall Protective Antigen Prediction = **0.5818** (Probable **ANTIGEN**).

>EHO9885718.1 bifunctional glycosyl transferase/transpeptidase [Salmonella enterica subsp. enterica serovar Infantis] Overall Protective Antigen Prediction = **0.4395** (Probable **ANTIGEN**).

>EHO9885719.1 ATP-dependent helicase HrpB [Salmonella enterica subsp. enterica serovar Infantis] Overall Protective Antigen Prediction = **0.5419** (Probable **ANTIGEN**).

>EHO9885720.1 RNA 2',3'-cyclic phosphodiesterase [Salmonella enterica subsp. enterica serovar Infantis] Overall Protective Antigen Prediction = **0.3648** (Probable **NON-ANTIGEN**).

>EHO9885721.1 DNA/RNA nuclease SfsA [Salmonella enterica subsp. enterica serovar Infantis] Overall Protective Antigen Prediction = **0.5563** (Probable **ANTIGEN**).

>EHO9885722.1 RNA polymerase-binding protein DksA [Salmonella enterica subsp. enterica serovar Infantis] Overall Protective Antigen Prediction = **0.4897** (Probable **ANTIGEN**).

>EHO9885723.1 tRNA glutamyl-Q(34) synthetase GluQRS [Salmonella enterica subsp. enterica serovar Infantis] Overall Protective Antigen Prediction = **0.3354** (Probable **NON-ANTIGEN**).

>EHO9885724.1 polynucleotide adenylyltransferase PcnB [Salmonella enterica subsp. enterica serovar Infantis] Overall Protective Antigen Prediction = **0.5463** (Probable **ANTIGEN**).

>EHO9885725.1 2-amino-4-hydroxy-6-hydroxymethyldihydropteridine diphosphokinase [Salmonella enterica subsp. enterica serovar Infantis] Overall Protective Antigen Prediction = **0.4533** (Probable **ANTIGEN**).

>EHO9885726.1 3-methyl-2-oxobutanoate hydroxymethyltransferase [Salmonella enterica subsp. enterica serovar Infantis] Overall Protective Antigen Prediction = **0.4358** (Probable **ANTIGEN**).

>EHO9885727.1 pantoate--beta-alanine ligase [Salmonella enterica subsp. enterica serovar Infantis] Overall Protective Antigen Prediction = **0.4029** (Probable **ANTIGEN**).

>EHO9885728.1 aspartate 1-decarboxylase [Salmonella enterica subsp. enterica serovar Infantis] Overall Protective Antigen Prediction = **0.5462** (Probable **ANTIGEN**).

>EHO9885729.1 polysaccharide deacetylase family protein [Salmonella enterica subsp. enterica serovar Infantis] Overall Protective Antigen Prediction = **0.4172** (Probable **ANTIGEN**).

>EHO9885730.1 PTS sugar transporter subunit IIA [Salmonella enterica subsp. enterica serovar Infantis] Overall Protective Antigen Prediction = **0.2971** (Probable **NON-ANTIGEN**).

>EHO9885731.1 fimbrial protein [Salmonella enterica subsp. enterica serovar Infantis] Overall Protective Antigen Prediction = **0.6596** (Probable **ANTIGEN**).

>EHO9885732.1 fimbria/pilus periplasmic chaperone [Salmonella enterica subsp. enterica serovar Infantis] Overall Protective Antigen Prediction = **0.5176** (Probable **ANTIGEN**).

>EHO9885733.1 fimbrial outer membrane usher protein [Salmonella enterica subsp. enterica serovar Infantis] Overall Protective Antigen Prediction = **0.6030** (Probable **ANTIGEN**).

>EHO9885734.1 fimbrial protein [Salmonella enterica subsp. enterica serovar Infantis] Overall Protective Antigen Prediction = **0.8662** (Probable **ANTIGEN**).

>EHO9885735.1 ABC transporter permease [Salmonella enterica subsp. enterica serovar Infantis] Overall Protective Antigen Prediction = **0.5099** (Probable **ANTIGEN**).

>EHO9885736.1 ABC transporter ATP-binding protein [Salmonella enterica subsp. enterica serovar Infantis] Overall Protective Antigen Prediction = **0.5059** (Probable **ANTIGEN**).

>EHO9885737.1 carbonate dehydratase [Salmonella enterica subsp. enterica serovar Infantis] Overall Protective Antigen Prediction = **0.5087** (Probable **ANTIGEN**).

>EHO9885738.1 hypoxanthine phosphoribosyltransferase [Salmonella enterica subsp. enterica

serovar Infantis] Overall Protective Antigen Prediction = **0.2950** (Probable **NON-ANTIGEN**).

>EHO9885739.1 pyrroloquinoline quinone-dependent dehydrogenase [Salmonella enterica subsp. enterica serovar Infantis] Overall Protective Antigen Prediction = **0.5009** (Probable **ANTIGEN**).

>EHO9885740.1 multicopper oxidase CueO [Salmonella enterica subsp. enterica serovar Infantis] Overall Protective Antigen Prediction = **0.5108** (Probable **ANTIGEN**).

>EHO9885741.1 YacC family pilotin-like protein [Salmonella enterica subsp. enterica serovar Infantis] Overall Protective Antigen Prediction = **0.2540** (Probable **NON-ANTIGEN**).

>EHO9885742.1 polyamine aminopropyltransferase [Salmonella enterica subsp. enterica serovar Infantis] Overall Protective Antigen Prediction = **0.2615** (Probable **NON-ANTIGEN**).

>EHO9885743.1 adenosylmethionine decarboxylase [Salmonella enterica subsp. enterica serovar Infantis] Overall Protective Antigen Prediction = **0.3810** (Probable **NON-ANTIGEN**).

>EHO9885744.1 DeoR/GlpR transcriptional regulator [Salmonella enterica subsp. enterica serovar Infantis] Overall Protective Antigen Prediction = **0.4445** (Probable **ANTIGEN**).

>EHO9885745.1 D-threonate 4-phosphate dehydrogenase [Salmonella enterica subsp. enterica serovar Infantis] Overall Protective Antigen Prediction = **0.5195** (Probable **ANTIGEN**).

>EHO9885746.1 D-threonate kinase [Salmonella enterica subsp. enterica serovar Infantis] Overall Protective Antigen Prediction = **0.4138** (Probable **ANTIGEN**).

>EHO9885747.1 2-keto-3-deoxygluconate permease 1 [Salmonella enterica subsp. enterica serovar Infantis] Overall Protective Antigen Prediction = **0.4635** (Probable **ANTIGEN**).

>EHO9885748.1 YacL family protein [Salmonella enterica subsp. enterica serovar Infantis] Overall Protective Antigen Prediction = **0.4173** (Probable **ANTIGEN**).

>EHO9885749.1 phosphotyrosine protein phosphatase [Salmonella enterica subsp. enterica serovar Infantis] Overall Protective Antigen Prediction = **0.4079** (Probable **ANTIGEN**).

>EHO9885750.1 hypothetical protein KND05_001048 [Salmonella enterica subsp. enterica serovar Infantis] Overall Protective Antigen Prediction = **0.4821** (Probable **ANTIGEN**).

>EHO9885751.1 bifunctional aconitate hydratase 2/2-methylisocitrate dehydratase [Salmonella enterica subsp. enterica serovar Infantis] Overall Protective Antigen Prediction = **0.4765** (Probable **ANTIGEN**).

>EHO9885752.1 DUF3300 domain-containing protein [Salmonella enterica subsp. enterica serovar Infantis] Overall Protective Antigen Prediction = **0.6389** (Probable **ANTIGEN**).

>EHO9885753.1 DUF2950 family protein [Salmonella enterica subsp. enterica serovar Infantis] Overall Protective Antigen Prediction = **0.5836** (Probable **ANTIGEN**).

>EHO9885754.1 outer membrane protein [Salmonella enterica subsp. enterica serovar Infantis] Overall Protective Antigen Prediction = **0.6207** (Probable **ANTIGEN**).

>EHO9885755.1 dihydrolipoyl dehydrogenase [Salmonella enterica subsp. enterica serovar Infantis] Overall Protective Antigen Prediction = **0.4220** (Probable **ANTIGEN**).

>EHO9885756.1 pyruvate dehydrogenase complex dihydrolipoyllysine-residue acetyltransferase [Salmonella enterica subsp. enterica serovar Infantis] Overall Protective Antigen Prediction = **0.6932** (Probable **ANTIGEN**).

>EHO9885757.1 pyruvate dehydrogenase (acetyl-transferring), homodimeric type [Salmonella enterica subsp. enterica serovar Infantis] Overall Protective Antigen Prediction = **0.4544** (Probable **ANTIGEN**).

>EHO9885758.1 pyruvate dehydrogenase complex transcriptional repressor PdhR [Salmonella enterica subsp. enterica serovar Infantis] Overall Protective Antigen Prediction = **0.3537** (Probable **NON-ANTIGEN**).

>EHO9885759.1 aromatic amino acid transporter AroP [Salmonella enterica subsp. enterica serovar Infantis] Overall Protective Antigen Prediction = **0.4995** (Probable **ANTIGEN**).

>EHO9885760.1 MFS transporter [Salmonella enterica subsp. enterica serovar Infantis] Overall Protective Antigen Prediction = **0.5329** (Probable **ANTIGEN**).

>EHO9885761.1 family 43 glycosylhydrolase [Salmonella enterica subsp. enterica serovar Infantis] Overall Protective Antigen Prediction = **0.3694** (Probable **NON-ANTIGEN**).

>EHO9885762.1 beta-lactamase regulator AmpE [Salmonella enterica subsp. enterica serovar Infantis] Overall Protective Antigen Prediction = **0.3857** (Probable **NON-ANTIGEN**).

>EHO9885763.1 1,6-anhydro-N-acetylmuramyl-L-alanine amidase AmpD [Salmonella enterica subsp. enterica serovar Infantis] Overall Protective Antigen Prediction = **0.4327** (Probable **ANTIGEN**).

>EHO9885764.1 carboxylating nicotinate-nucleotide diphosphorylase [Salmonella enterica subsp. enterica serovar Infantis] Overall Protective Antigen Prediction = **0.5413** (Probable **ANTIGEN**).

>EHO9885765.1 prepilin peptidase-dependent pilin [Salmonella enterica subsp. enterica serovar Infantis] Overall Protective Antigen Prediction = **0.4119** (Probable **ANTIGEN**).

>EHO9885766.1 type II secretion system protein GspE [Salmonella enterica subsp. enterica serovar Infantis] Overall Protective Antigen Prediction = **0.5237** (Probable **ANTIGEN**).

>EHO9885767.1 protein transport protein HofC [Salmonella enterica subsp. enterica serovar Infantis] Overall Protective Antigen Prediction = **0.3589** (Probable **NON-ANTIGEN**).

>EHO9885768.1 GMP reductase [Salmonella enterica subsp. enterica serovar Infantis] Overall Protective Antigen Prediction = **0.4860** (Probable **ANTIGEN**).

>EHO9885769.1 dephospho-CoA kinase [Salmonella enterica subsp. enterica serovar Infantis] Overall Protective Antigen Prediction = **0.3761** (Probable **NON-ANTIGEN**).

>EHO9885770.1 cell division protein ZapD [Salmonella enterica subsp. enterica serovar Infantis] Overall Protective Antigen Prediction = **0.4203** (Probable **ANTIGEN**).

>EHO9885771.1 DNA gyrase inhibitor YacG [Salmonella enterica subsp. enterica serovar Infantis] Overall Protective Antigen Prediction = **0.2908** (Probable **NON-ANTIGEN**).

>EHO9885772.1 LysR family transcriptional regulator [Salmonella enterica subsp. enterica serovar Infantis] Overall Protective Antigen Prediction = **0.5219** (Probable **ANTIGEN**).

>EHO9885773.1 aldo/keto reductase [Salmonella enterica subsp. enterica serovar Infantis] Overall Protective Antigen Prediction = **0.4097** (Probable **ANTIGEN**).

>EHO9885774.1 8-oxo-dGTP diphosphatase MutT [Salmonella enterica subsp. enterica serovar Infantis] Overall Protective Antigen Prediction = **0.2585** (Probable **NON-ANTIGEN**).

>EHO9885775.1 preprotein translocase subunit SecA [Salmonella enterica subsp. enterica serovar Infantis] Overall Protective Antigen Prediction = **0.5471** (Probable **ANTIGEN**).

>EHO9885776.1 secA regulator SecM [Salmonella enterica subsp. enterica serovar Infantis] Overall Protective Antigen Prediction = **0.4175** (Probable **ANTIGEN**).

>EHO9885777.1 UDP-3-O-acyl-N-acetylglucosamine deacetylase [Salmonella enterica subsp. enterica serovar Infantis] Overall Protective Antigen Prediction = **0.4369** (Probable **ANTIGEN**).

>EHO9885778.1 cell division protein FtsZ [Salmonella enterica subsp. enterica serovar Infantis] Overall Protective Antigen Prediction = **0.5595** (Probable **ANTIGEN**).

>EHO9885779.1 cell division protein FtsA [Salmonella enterica subsp. enterica serovar Infantis] Overall Protective Antigen Prediction = **0.4260** (Probable **ANTIGEN**).

>EHO9885780.1 cell division protein FtsQ [Salmonella enterica subsp. enterica serovar Infantis] Overall Protective Antigen Prediction = **0.6343** (Probable **ANTIGEN**).

>EHO9885781.1 D-alanine--D-alanine ligase [Salmonella enterica subsp. enterica serovar Infantis] Overall Protective Antigen Prediction = **0.4830** (Probable **ANTIGEN**).

>EHO9885782.1 UDP-N-acetylmuramate--L-alanine ligase [Salmonella enterica subsp. enterica serovar Infantis] Overall Protective Antigen Prediction = **0.5151** (Probable **ANTIGEN**).

>EHO9885783.1 undecaprenyldiphospho-muramoylpentapeptide beta-N-acetylglucosaminyltransferase [Salmonella enterica subsp. enterica serovar Infantis] Overall Protective Antigen Prediction = **0.4946** (Probable **ANTIGEN**).

>EHO9885784.1 cell division protein FtsW [Salmonella enterica subsp. enterica serovar Infantis] Overall Protective Antigen Prediction = **0.3711** (Probable **NON-ANTIGEN**).

>EHO9885785.1 UDP-N-acetylmuramoyl-L-alanine--D-glutamate ligase [Salmonella enterica subsp. enterica serovar Infantis] Overall Protective Antigen Prediction = **0.5380** (Probable **ANTIGEN**).

>EHO9885786.1 phospho-N-acetylmuramoyl-pentapeptide-transferase [Salmonella enterica subsp. enterica serovar Infantis] Overall Protective Antigen Prediction = **0.3968** (Probable **NON-ANTIGEN**).

>EHO9885787.1 UDP-N-acetylmuramoyl-tripeptide--D-alanyl-D-alanine ligase [Salmonella enterica subsp. enterica serovar Infantis] Overall Protective Antigen Prediction = **0.4635** (Probable **ANTIGEN**).

>EHO9885788.1 UDP-N-acetylmuramoyl-L-alanyl-D-glutamate--2,6-diaminopimelate ligase [Salmonella enterica subsp. enterica serovar Infantis] Overall Protective Antigen Prediction = **0.4480** (Probable **ANTIGEN**).

>EHO9885789.1 peptidoglycan glycosyltransferase FtsI [Salmonella enterica subsp. enterica serovar

Infantis] Overall Protective Antigen Prediction = **0.5698** (Probable **ANTIGEN**).

>EHO9885790.1 cell division protein FtsL [Salmonella enterica subsp. enterica serovar Infantis] Overall Protective Antigen Prediction = **0.3731** (Probable **NON-ANTIGEN**).

>EHO9885791.1 16S rRNA (cytosine(1402)-N(4))-methyltransferase RsmH [Salmonella enterica subsp. enterica serovar Infantis] Overall Protective Antigen Prediction = **0.3879** (Probable **NON-ANTIGEN**).

>EHO9885792.1 division/cell wall cluster transcriptional repressor MraZ [Salmonella enterica subsp. enterica serovar Infantis] Overall Protective Antigen Prediction = **0.3762** (Probable **NON-ANTIGEN**).

>EHO9885793.1 catabolite repressor/activator [Salmonella enterica subsp. enterica serovar Infantis] Overall Protective Antigen Prediction = **0.2745** (Probable **NON-ANTIGEN**).

>EHO9885794.1 acetolactate synthase small subunit [Salmonella enterica subsp. enterica serovar Infantis] Overall Protective Antigen Prediction = **0.2660** (Probable **NON-ANTIGEN**).

>EHO9885795.1 acetolactate synthase 3 large subunit [Salmonella enterica subsp. enterica serovar Infantis] Overall Protective Antigen Prediction = **0.3957** (Probable **NON-ANTIGEN**).

>EHO9885796.1 transcriptional regulator LeuO [Salmonella enterica subsp. enterica serovar Infantis] Overall Protective Antigen Prediction = **0.4259** (Probable **ANTIGEN**).

>EHO9885797.1 leu operon leader peptide [Salmonella enterica subsp. enterica serovar Infantis] Overall Protective Antigen Prediction = **-0.0884** (Probable **NON-ANTIGEN**).

>EHO9885798.1 2-isopropylmalate synthase [Salmonella enterica subsp. enterica serovar Infantis] Overall Protective Antigen Prediction = **0.5496** (Probable **ANTIGEN**).

>EHO9885799.1 3-isopropylmalate dehydrogenase [Salmonella enterica subsp. enterica serovar Infantis] Overall Protective Antigen Prediction = **0.4899** (Probable **ANTIGEN**).

>EHO9885800.1 3-isopropylmalate dehydratase large subunit [Salmonella enterica subsp. enterica serovar Infantis] Overall Protective Antigen Prediction = **0.6218** (Probable **ANTIGEN**).

>EHO9885801.1 3-isopropylmalate dehydratase small subunit [Salmonella enterica subsp. enterica serovar Infantis] Overall Protective Antigen Prediction = **0.5859** (Probable **ANTIGEN**).

>EHO9885802.1 DUF5339 domain-containing protein [Salmonella enterica subsp. enterica serovar Infantis] Overall Protective Antigen Prediction = **0.5493** (Probable **ANTIGEN**).

>EHO9885803.1 glucose uptake inhibitor SgrT [Salmonella enterica subsp. enterica serovar Infantis] Overall Protective Antigen Prediction = **0.1907** (Probable **NON-ANTIGEN**).

>EHO9885804.1 HTH-type transcriptional regulator SgrR [Salmonella enterica subsp. enterica serovar Infantis] Overall Protective Antigen Prediction = **0.4537** (Probable **ANTIGEN**).

>EHO9885805.1 thiamine ABC transporter substrate binding subunit [Salmonella enterica subsp. enterica serovar Infantis] Overall Protective Antigen Prediction = **0.3235** (Probable **NON-ANTIGEN**).

>EHO9885806.1 thiamine/thiamine pyrophosphate ABC transporter permease ThiP [Salmonella

enterica subsp. enterica serovar Infantis] Overall Protective Antigen Prediction = **0.5023** (Probable **ANTIGEN**).

>EHO9885807.1 thiamine ABC transporter ATP-binding protein ThiQ [Salmonella enterica subsp. enterica serovar Infantis] Overall Protective Antigen Prediction = **0.4645** (Probable **ANTIGEN**).

>EHO9885808.1 DedA family protein [Salmonella enterica subsp. enterica serovar Infantis] Overall Protective Antigen Prediction = **0.2223** (Probable **NON-ANTIGEN**).

>EHO9885809.1 arabinose operon transcriptional regulator AraC [Salmonella enterica subsp. enterica serovar Infantis] Overall Protective Antigen Prediction = **0.3155** (Probable **NON-ANTIGEN**).

>EHO9885810.1 ribulokinase [Salmonella enterica subsp. enterica serovar Infantis] Overall Protective Antigen Prediction = **0.5172** (Probable **ANTIGEN**).

>EHO9885811.1 L-arabinose isomerase [Salmonella enterica subsp. enterica serovar Infantis] Overall Protective Antigen Prediction = **0.4251** (Probable **ANTIGEN**).

>EHO9885812.1 L-ribulose-5-phosphate 4-epimerase [Salmonella enterica subsp. enterica serovar Infantis] Overall Protective Antigen Prediction = **0.2776** (Probable **NON-ANTIGEN**).

>EHO9885813.1 DUF4751 family protein [Salmonella enterica subsp. enterica serovar Infantis] Overall Protective Antigen Prediction = **0.3273** (Probable **NON-ANTIGEN**).

>EHO9885814.1 YdcF family protein [Salmonella enterica subsp. enterica serovar Infantis] Overall Protective Antigen Prediction = **0.4754** (Probable **ANTIGEN**).

>EHO9885815.1 DNA polymerase II [Salmonella enterica subsp. enterica serovar Infantis] Overall Protective Antigen Prediction = **0.4009** (Probable **ANTIGEN**).

>EHO9885816.1 RNA polymerase-associated protein RapA [Salmonella enterica subsp. enterica serovar Infantis] Overall Protective Antigen Prediction = **0.4397** (Probable **ANTIGEN**).

>EHO9885817.1 bifunctional tRNA pseudouridine(32) synthase/23S rRNA pseudouridine(746) synthase RluA [Salmonella enterica subsp. enterica serovar Infantis] Overall Protective Antigen Prediction = **0.4911** (Probable **ANTIGEN**).

>EHO9885818.1 co-chaperone DjlA [Salmonella enterica subsp. enterica serovar Infantis] Overall Protective Antigen Prediction = **0.3522** (Probable **NON-ANTIGEN**).

>EHO9885819.1 LPS assembly protein LptD [Salmonella enterica subsp. enterica serovar Infantis] Overall Protective Antigen Prediction = **0.6301** (Probable **ANTIGEN**).

>EHO9885820.1 peptidylprolyl isomerase SurA [Salmonella enterica subsp. enterica serovar Infantis] Overall Protective Antigen Prediction = **0.4823** (Probable **ANTIGEN**).

>EHO9885821.1 4-hydroxythreonine-4-phosphate dehydrogenase PdxA [Salmonella enterica subsp. enterica serovar Infantis] Overall Protective Antigen Prediction = **0.4601** (Probable **ANTIGEN**).

>EHO9885822.1 16S rRNA (adenine(1518)-N(6)/adenine(1519)-N(6))-dimethyltransferase RsmA [Salmonella enterica subsp. enterica serovar Infantis] Overall Protective Antigen Prediction = **0.4056** (Probable **ANTIGEN**).

>EHO9885823.1 Co2+/Mg2+ efflux protein ApaG [Salmonella enterica subsp. enterica serovar

Infantis] Overall Protective Antigen Prediction = **0.8836** (Probable **ANTIGEN**).

>EHO9885824.1 bis(5'-nucleosyl)-tetraphosphatase (symmetrical) ApaH [Salmonella enterica subsp. enterica serovar Infantis] Overall Protective Antigen Prediction = **0.3020** (Probable **NON-ANTIGEN**).

>EHO9885825.1 type 3 dihydrofolate reductase [Salmonella enterica subsp. enterica serovar Infantis] Overall Protective Antigen Prediction = **0.3415** (Probable **NON-ANTIGEN**).

>EHO9885826.1 glutathione-regulated potassium-efflux system protein KefC [Salmonella enterica subsp. enterica serovar Infantis] Overall Protective Antigen Prediction = **0.4285** (Probable **ANTIGEN**).

>EHO9885827.1 glutathione-regulated potassium-efflux system oxidoreductase KefF [Salmonella enterica subsp. enterica serovar Infantis] Overall Protective Antigen Prediction = **0.6857** (Probable **ANTIGEN**).

>EHO9885828.1 sulfatase-like hydrolase/transferase [Salmonella enterica subsp. enterica serovar Infantis] Overall Protective Antigen Prediction = **0.3427** (Probable **NON-ANTIGEN**).

>EHO9885829.1 DUF1471 domain-containing protein [Salmonella enterica subsp. enterica serovar Infantis] Overall Protective Antigen Prediction = **0.4905** (Probable **ANTIGEN**).

>EHO9885830.1 hypothetical protein KND05_001129 [Salmonella enterica subsp. enterica serovar Infantis] Overall Protective Antigen Prediction = **0.7512** (Probable **ANTIGEN**).

>EHO9885831.1 YgdI/YgdR family lipoprotein [Salmonella enterica subsp. enterica serovar Infantis] Overall Protective Antigen Prediction = **0.5641** (Probable **ANTIGEN**).

>EHO9885832.1 MFS transporter [Salmonella enterica subsp. enterica serovar Infantis] Overall Protective Antigen Prediction = **0.3826** (Probable **NON-ANTIGEN**).

>EHO9885833.1 ferredoxin-like protein FixX [Salmonella enterica subsp. enterica serovar Infantis] Overall Protective Antigen Prediction = **0.7714** (Probable **ANTIGEN**).

>EHO9885834.1 FAD-dependent oxidoreductase [Salmonella enterica subsp. enterica serovar Infantis] Overall Protective Antigen Prediction = **0.3899** (Probable **NON-ANTIGEN**).

>EHO9885835.1 electron transfer flavoprotein subunit alpha/FixB family protein [Salmonella enterica subsp. enterica serovar Infantis] Overall Protective Antigen Prediction = **0.5433** (Probable **ANTIGEN**).

>EHO9885836.1 electron transfer flavoprotein FixA [Salmonella enterica subsp. enterica serovar Infantis] Overall Protective Antigen Prediction = **0.4816** (Probable **ANTIGEN**).

>EHO9885837.1 L-carnitine/gamma-butyrobetaine antiport BCCT transporter [Salmonella enterica subsp. enterica serovar Infantis] Overall Protective Antigen Prediction = **0.6939** (Probable **ANTIGEN**).

>EHO9885838.1 crotonobetainyl-CoA dehydrogenase [Salmonella enterica subsp. enterica serovar Infantis] Overall Protective Antigen Prediction = **0.3706** (Probable **NON-ANTIGEN**).

>EHO9885839.1 L-carnitine CoA-transferase [Salmonella enterica subsp. enterica serovar Infantis] Overall Protective Antigen Prediction = **0.3198** (Probable **NON-ANTIGEN**).

>EHO9885840.1 crotonobetaine/carnitine-CoA ligase [Salmonella enterica subsp. enterica serovar Infantis] Overall Protective Antigen Prediction = **0.4082** (Probable **ANTIGEN**).

>EHO9885841.1 crotonobetainyl-CoA hydratase [Salmonella enterica subsp. enterica serovar Infantis] Overall Protective Antigen Prediction = **0.3886** (Probable **NON-ANTIGEN**).

>EHO9885842.1 carnitine operon protein CaiE [Salmonella enterica subsp. enterica serovar Infantis] Overall Protective Antigen Prediction = **0.4695** (Probable **ANTIGEN**).

>EHO9885843.1 carnitine metabolism transcriptional regulator CaiF [Salmonella enterica subsp. enterica serovar Infantis] Overall Protective Antigen Prediction = **0.5400** (Probable **ANTIGEN**).

>EHO9885844.1 carbamoyl-phosphate synthase large subunit [Salmonella enterica subsp. enterica serovar Infantis] Overall Protective Antigen Prediction = **0.5185** (Probable **ANTIGEN**).

>EHO9885845.1 glutamine-hydrolyzing carbamoyl-phosphate synthase small subunit [Salmonella enterica subsp. enterica serovar Infantis] Overall Protective Antigen Prediction = **0.4129** (Probable **ANTIGEN**).

>EHO9885846.1 4-hydroxy-tetrahydrodipicolinate reductase [Salmonella enterica subsp. enterica serovar Infantis] Overall Protective Antigen Prediction = **0.4800** (Probable **ANTIGEN**).

>EHO9885847.1 triphosphoribosyl-dephospho-CoA synthase CitG [Salmonella enterica subsp. enterica serovar Infantis] Overall Protective Antigen Prediction = **0.3960** (Probable **NON-ANTIGEN**).

>EHO9885848.1 citrate lyase holo-[acyl-carrier protein] synthase [Salmonella enterica subsp. enterica serovar Infantis] Overall Protective Antigen Prediction = **0.5847** (Probable **ANTIGEN**).

>EHO9885849.1 citrate lyase subunit alpha [Salmonella enterica subsp. enterica serovar Infantis] Overall Protective Antigen Prediction = **0.5300** (Probable **ANTIGEN**).

>EHO9885850.1 citrate (pro-3S)-lyase subunit beta [Salmonella enterica subsp. enterica serovar Infantis] Overall Protective Antigen Prediction = **0.3512** (Probable **NON-ANTIGEN**).

>EHO9885851.1 citrate lyase acyl carrier protein [Salmonella enterica subsp. enterica serovar Infantis] Overall Protective Antigen Prediction = **0.5729** (Probable **ANTIGEN**).

>EHO9885852.1 [citrate (pro-3S)-lyase] ligase [Salmonella enterica subsp. enterica serovar Infantis] Overall Protective Antigen Prediction = **0.2023** (Probable **NON-ANTIGEN**).

>EHO9885853.1 citrate/sodium symporter CitS [Salmonella enterica subsp. enterica serovar Infantis] Overall Protective Antigen Prediction = **0.5841** (Probable **ANTIGEN**).

>EHO9885854.1 oxaloacetate decarboxylase subunit gamma [Salmonella enterica subsp. enterica serovar Infantis] Overall Protective Antigen Prediction = **0.6376** (Probable **ANTIGEN**).

>EHO9885855.1 sodium-extruding oxaloacetate decarboxylase subunit alpha [Salmonella enterica subsp. enterica serovar Infantis] Overall Protective Antigen Prediction = **0.4932** (Probable **ANTIGEN**).

>EHO9885856.1 oxalacetate decarboxylase subunit beta [Salmonella enterica subsp. enterica serovar Infantis] Overall Protective Antigen Prediction = **0.4069** (Probable **ANTIGEN**).

>EHO9885857.1 sensor histidine kinase [Salmonella enterica subsp. enterica serovar Infantis] Overall Protective Antigen Prediction = **0.3153** (Probable **NON-ANTIGEN**).

>EHO9885858.1 response regulator [Salmonella enterica subsp. enterica serovar Infantis] Overall Protective Antigen Prediction = **0.3714** (Probable **NON-ANTIGEN**).

>EHO9885859.1 ribonucleoside hydrolase RihC [Salmonella enterica subsp. enterica serovar Infantis] Overall Protective Antigen Prediction = **0.5258** (Probable **ANTIGEN**).

>EHO9885860.1 nitrite reductase [Salmonella enterica subsp. enterica serovar Infantis] Overall Protective Antigen Prediction = **0.5229** (Probable **ANTIGEN**).

>EHO9885861.1 4-hydroxy-3-methylbut-2-enyl diphosphate reductase [Salmonella enterica subsp. enterica serovar Infantis] Overall Protective Antigen Prediction = **0.4010** (Probable **ANTIGEN**).

>EHO9885862.1 FKBP-type peptidyl-prolyl cis-trans isomerase [Salmonella enterica subsp. enterica serovar Infantis] Overall Protective Antigen Prediction = **0.8058** (Probable **ANTIGEN**).

>EHO9885863.1 signal peptidase II [Salmonella enterica subsp. enterica serovar Infantis] Overall Protective Antigen Prediction = **0.5063** (Probable **ANTIGEN**).

>EHO9885864.1 isoleucine--tRNA ligase [Salmonella enterica subsp. enterica serovar Infantis] Overall Protective Antigen Prediction = **0.4147** (Probable **ANTIGEN**).

>EHO9885865.1 bifunctional riboflavin kinase/FAD synthetase [Salmonella enterica subsp. enterica serovar Infantis] Overall Protective Antigen Prediction = **0.4608** (Probable **ANTIGEN**).

>EHO9885866.1 DUF2575 family protein [Salmonella enterica subsp. enterica serovar Infantis] Overall Protective Antigen Prediction = **0.6294** (Probable **ANTIGEN**).

>EHO9885867.1 30S ribosomal protein S20 [Salmonella enterica subsp. enterica serovar Infantis] Overall Protective Antigen Prediction = **0.4206** (Probable **ANTIGEN**).

>EHO9885868.1 MFS transporter [Salmonella enterica subsp. enterica serovar Infantis] Overall Protective Antigen Prediction = **0.6117** (Probable **ANTIGEN**).

>EHO9885869.1 glycoside hydrolase family 31 protein [Salmonella enterica subsp. enterica serovar Infantis] Overall Protective Antigen Prediction = **0.4530** (Probable **ANTIGEN**).

>EHO9885870.1 transcriptional activator NhaR [Salmonella enterica subsp. enterica serovar Infantis] Overall Protective Antigen Prediction = **0.3094** (Probable **NON-ANTIGEN**).

>EHO9885871.1 Na+/H+ antiporter NhaA [Salmonella enterica subsp. enterica serovar Infantis] Overall Protective Antigen Prediction = **0.5126** (Probable **ANTIGEN**).

>EHO9885872.1 aryl sulfotransferase [Salmonella enterica subsp. enterica serovar Infantis] Overall Protective Antigen Prediction = **0.3297** (Probable **NON-ANTIGEN**).

>EHO9885873.1 IS1 family transposase [Salmonella enterica subsp. enterica serovar Infantis] Overall Protective Antigen Prediction = **0.4079** (Probable **ANTIGEN**).

>EHO9885874.1 anaerobic sulfatase maturase [Salmonella enterica subsp. enterica serovar Infantis] Overall Protective Antigen Prediction = **0.4173** (Probable **ANTIGEN**).

>EHO9885875.1 sulfatase-like hydrolase/transferase [Salmonella enterica subsp. enterica serovar Infantis] Overall Protective Antigen Prediction = **0.3685** (Probable **NON-ANTIGEN**).

>EHO9885876.1 hypothetical protein KND05_001177 [Salmonella enterica subsp. enterica serovar Infantis] Overall Protective Antigen Prediction = **0.4275** (Probable **ANTIGEN**).

>EHO9885877.1 5'-nucleotidase C-terminal domain-containing protein [Salmonella enterica subsp. enterica serovar Infantis] Overall Protective Antigen Prediction = **0.6628** (Probable **ANTIGEN**).

>EHO9885878.1 arylsulfatase [Salmonella enterica subsp. enterica serovar Infantis] Overall Protective Antigen Prediction = **0.5971** (Probable **ANTIGEN**).

>EHO9885879.1 transcriptional regulator [Salmonella enterica subsp. enterica serovar Infantis] Overall Protective Antigen Prediction = **0.4718** (Probable **ANTIGEN**).

>EHO9885880.1 hypothetical protein KND05_001181 [Salmonella enterica subsp. enterica serovar Infantis] Overall Protective Antigen Prediction = **0.4090** (Probable **ANTIGEN**).

>EHO9885881.1 DsbA family protein [Salmonella enterica subsp. enterica serovar Infantis] Overall Protective Antigen Prediction = **0.5413** (Probable **ANTIGEN**).

>EHO9885882.1 fimbrial chaperone BcfG [Salmonella enterica subsp. enterica serovar Infantis] Overall Protective Antigen Prediction = **0.4324** (Probable **ANTIGEN**).

>EHO9885883.1 fimbrial protein BcfF [Salmonella enterica subsp. enterica serovar Infantis] Overall Protective Antigen Prediction = **0.6975** (Probable **ANTIGEN**).

>EHO9885884.1 fimbrial protein BcfE [Salmonella enterica subsp. enterica serovar Infantis] Overall Protective Antigen Prediction = **0.6961** (Probable **ANTIGEN**).

>EHO9885885.1 fimbrial protein BcfD [Salmonella enterica subsp. enterica serovar Infantis] Overall Protective Antigen Prediction = **0.6728** (Probable **ANTIGEN**).

>EHO9885886.1 fimbrial usher BcfC [Salmonella enterica subsp. enterica serovar Infantis] Overall Protective Antigen Prediction = **0.5614** (Probable **ANTIGEN**).

>EHO9885887.1 fimbrial biogenesis chaperone BcfB [Salmonella enterica subsp. enterica serovar Infantis] Overall Protective Antigen Prediction = **0.5164** (Probable **ANTIGEN**).

>EHO9885888.1 fimbrial protein BcfA [Salmonella enterica subsp. enterica serovar Infantis] Overall Protective Antigen Prediction = **0.7624** (Probable **ANTIGEN**).

>EHO9885889.1 helix-turn-helix domain-containing protein [Salmonella enterica subsp. enterica serovar Infantis] Overall Protective Antigen Prediction = **0.1950** (Probable **NON-ANTIGEN**).

>EHO9885890.1 chitinase [Salmonella enterica subsp. enterica serovar Infantis] Overall Protective Antigen Prediction = **0.4694** (Probable **ANTIGEN**).

>EHO9885891.1 chitinase [Salmonella enterica subsp. enterica serovar Infantis] Overall Protective Antigen Prediction = **0.6045** (Probable **ANTIGEN**).

>EHO9885892.1 transcriptional regulator [Salmonella enterica subsp. enterica serovar Infantis] Overall Protective Antigen Prediction = **0.4726** (Probable **ANTIGEN**).

>EHO9885893.1 glycoside hydrolase family 108 protein [Salmonella enterica subsp. enterica serovar Infantis] Overall Protective Antigen Prediction = **0.4702** (Probable **ANTIGEN**).

>EHO9885894.1 phage holin family protein [Salmonella enterica subsp. enterica serovar Infantis] Overall Protective Antigen Prediction = **0.2420** (Probable **NON-ANTIGEN**).

>EHO9885895.1 LysR family transcriptional regulator [Salmonella enterica subsp. enterica serovar Infantis] Overall Protective Antigen Prediction = **0.2295** (Probable **NON-ANTIGEN**).

>EHO9885896.1 molecular chaperone DnaJ [Salmonella enterica subsp. enterica serovar Infantis] Overall Protective Antigen Prediction = **0.6880** (Probable **ANTIGEN**).

>EHO9885897.1 molecular chaperone DnaK [Salmonella enterica subsp. enterica serovar Infantis] Overall Protective Antigen Prediction = **0.6280** (Probable **ANTIGEN**).

>EHO9885898.1 DUF2541 family protein [Salmonella enterica subsp. enterica serovar Infantis] Overall Protective Antigen Prediction = **0.4904** (Probable **ANTIGEN**).

>EHO9885899.1 acidic protein MsyB [Salmonella enterica subsp. enterica serovar Infantis] Overall Protective Antigen Prediction = **0.3101** (Probable **NON-ANTIGEN**).

>EHO9885900.1 acetate uptake transporter [Salmonella enterica subsp. enterica serovar Infantis] Overall Protective Antigen Prediction = **0.2352** (Probable **NON-ANTIGEN**).

>EHO9885901.1 molybdopterin adenylyltransferase [Salmonella enterica subsp. enterica serovar Infantis] Overall Protective Antigen Prediction = **0.4484** (Probable **ANTIGEN**).

>EHO9885902.1 transaldolase [Salmonella enterica subsp. enterica serovar Infantis] Overall Protective Antigen Prediction = **0.3677** (Probable **NON-ANTIGEN**).

>EHO9885903.1 sodium:alanine symporter family protein [Salmonella enterica subsp. enterica serovar Infantis] Overall Protective Antigen Prediction = **0.5372** (Probable **ANTIGEN**).

>EHO9885904.1 peroxide stress protein YaaA [Salmonella enterica subsp. enterica serovar Infantis] Overall Protective Antigen Prediction = **0.3685** (Probable **NON-ANTIGEN**).

>EHO9885905.1 threonine synthase [Salmonella enterica subsp. enterica serovar Infantis] Overall Protective Antigen Prediction = **0.2857** (Probable **NON-ANTIGEN**).

>EHO9885906.1 homoserine kinase [Salmonella enterica subsp. enterica serovar Infantis] Overall Protective Antigen Prediction = **0.3689** (Probable **NON-ANTIGEN**).

>EHO9885907.1 bifunctional aspartate kinase/homoserine dehydrogenase I [Salmonella enterica subsp. enterica serovar Infantis] Overall Protective Antigen Prediction = **0.3773** (Probable **NON-ANTIGEN**).

>EHO9885908.1 thr operon leader peptide [Salmonella enterica subsp. enterica serovar Infantis] Overall Protective Antigen Prediction = **0.9106** (Probable **ANTIGEN**).

>EHO9885909.1 tRNA/rRNA methyltransferase [Salmonella enterica subsp. enterica serovar Infantis] Overall Protective Antigen Prediction = **0.2930** (Probable **NON-ANTIGEN**).

>EHO9885910.1 hypothetical protein KND05_001211 [Salmonella enterica subsp. enterica serovar

Infantis] Overall Protective Antigen Prediction = **0.2549** (Probable **NON-ANTIGEN**).

>EHO9885911.1 two-component system response regulator ArcA [Salmonella enterica subsp. enterica serovar Infantis] Overall Protective Antigen Prediction = **0.6206** (Probable **ANTIGEN**).

>EHO9885912.1 hypothetical protein KND05_001213 [Salmonella enterica subsp. enterica serovar Infantis] Overall Protective Antigen Prediction = **0.4076** (Probable **ANTIGEN**).

>EHO9885913.1 helix-turn-helix domain-containing protein [Salmonella enterica subsp. enterica serovar Infantis] Overall Protective Antigen Prediction = **0.3478** (Probable **NON-ANTIGEN**).

>EHO9885914.1 fimbrial protein SthA [Salmonella enterica subsp. enterica serovar Infantis] Overall Protective Antigen Prediction = **0.8225** (Probable **ANTIGEN**).

>EHO9885915.1 fimbrial assembly chaperone [Salmonella enterica subsp. enterica serovar Infantis] Overall Protective Antigen Prediction = **0.6729** (Probable **ANTIGEN**).

>EHO9885916.1 fimbrial outer membrane usher protein [Salmonella enterica subsp. enterica serovar Infantis] Overall Protective Antigen Prediction = **0.6693** (Probable **ANTIGEN**).

>EHO9885917.1 fimbrial protein SthD [Salmonella enterica subsp. enterica serovar Infantis] Overall Protective Antigen Prediction = **0.5035** (Probable **ANTIGEN**).

>EHO9885918.1 type 1 fimbrial protein [Salmonella enterica subsp. enterica serovar Infantis] Overall Protective Antigen Prediction = **0.8049** (Probable **ANTIGEN**).

>EHO9885919.1 cell envelope integrity protein CreD [Salmonella enterica subsp. enterica serovar Infantis] Overall Protective Antigen Prediction = **0.5414** (Probable **ANTIGEN**).

>EHO9885920.1 two-component system sensor histidine kinase CreC [Salmonella enterica subsp. enterica serovar Infantis] Overall Protective Antigen Prediction = **0.3623** (Probable **NON-ANTIGEN**).

>EHO9885921.1 two-component system response regulator CreB [Salmonella enterica subsp. enterica serovar Infantis] Overall Protective Antigen Prediction = **0.4265** (Probable **ANTIGEN**).

>EHO9885922.1 protein CreA [Salmonella enterica subsp. enterica serovar Infantis] Overall Protective Antigen Prediction = **0.4602** (Probable **ANTIGEN**).

>EHO9885923.1 MDR efflux pump AcrAB transcriptional activator RobA [Salmonella enterica subsp. enterica serovar Infantis] Overall Protective Antigen Prediction = **0.4945** (Probable **ANTIGEN**).

>EHO9885924.1 2,3-diphosphoglycerate-dependent phosphoglycerate mutase GpmB [Salmonella enterica subsp. enterica serovar Infantis] Overall Protective Antigen Prediction = **0.5199** (Probable **ANTIGEN**).

>EHO9885925.1 inosine/xanthosine triphosphatase [Salmonella enterica subsp. enterica serovar Infantis] Overall Protective Antigen Prediction = **0.4469** (Probable **ANTIGEN**).

>EHO9885926.1 colanic acid biosynthesis phosphomannomutase CpsG, partial [Salmonella enterica subsp. enterica serovar Infantis] Overall Protective Antigen Prediction = **0.5220** (Probable **ANTIGEN**).

>EHO9885927.1 mannose-1-phosphate guanyltransferase [Salmonella enterica subsp. enterica serovar Infantis] Overall Protective Antigen Prediction = **0.3503** (Probable **NON-ANTIGEN**).

>EHO9885928.1 colanic acid biosynthesis fucosyltransferase WcaI [Salmonella enterica subsp. enterica serovar Infantis] Overall Protective Antigen Prediction = **0.4747** (Probable **ANTIGEN**).

>EHO9885929.1 GDP-mannose mannosyl hydrolase [Salmonella enterica subsp. enterica serovar Infantis] Overall Protective Antigen Prediction = **0.5501** (Probable **ANTIGEN**).

>EHO9885930.1 GDP-L-fucose synthase [Salmonella enterica subsp. enterica serovar Infantis] Overall Protective Antigen Prediction = **0.2460** (Probable **NON-ANTIGEN**).

>EHO9885931.1 GDP-mannose 4,6-dehydratase [Salmonella enterica subsp. enterica serovar Infantis] Overall Protective Antigen Prediction = **0.4197** (Probable **ANTIGEN**).

>EHO9885932.1 colanic acid biosynthesis acetyltransferase WcaF [Salmonella enterica subsp. enterica serovar Infantis] Overall Protective Antigen Prediction = **0.4949** (Probable **ANTIGEN**).

>EHO9885933.1 colanic acid biosynthesis glycosyltransferase WcaE [Salmonella enterica subsp. enterica serovar Infantis] Overall Protective Antigen Prediction = **0.3879** (Probable **NON-ANTIGEN**).

>EHO9885934.1 putative colanic acid polymerase WcaD [Salmonella enterica subsp. enterica serovar Infantis] Overall Protective Antigen Prediction = **0.6579** (Probable **ANTIGEN**).

>EHO9885935.1 colanic acid biosynthesis glycosyltransferase WcaC [Salmonella enterica subsp. enterica serovar Infantis] Overall Protective Antigen Prediction = **0.5711** (Probable **ANTIGEN**).

>EHO9885936.1 colanic acid biosynthesis acetyltransferase WcaB [Salmonella enterica subsp. enterica serovar Infantis] Overall Protective Antigen Prediction = **0.6950** (Probable **ANTIGEN**).

>EHO9885937.1 colanic acid biosynthesis glycosyltransferase WcaA [Salmonella enterica subsp. enterica serovar Infantis] Overall Protective Antigen Prediction = **0.4156** (Probable **ANTIGEN**).

>EHO9885938.1 tyrosine-protein kinase Wzc [Salmonella enterica subsp. enterica serovar Infantis] Overall Protective Antigen Prediction = **0.3802** (Probable **NON-ANTIGEN**).

>EHO9885939.1 low molecular weight protein-tyrosine-phosphatase Wzb [Salmonella enterica subsp. enterica serovar Infantis] Overall Protective Antigen Prediction = **0.3106** (Probable **NON-ANTIGEN**).

>EHO9885940.1 polysaccharide export protein [Salmonella enterica subsp. enterica serovar Infantis] Overall Protective Antigen Prediction = **0.5616** (Probable **ANTIGEN**).

>EHO9885941.1 TerC family protein [Salmonella enterica subsp. enterica serovar Infantis] Overall Protective Antigen Prediction = **0.3922** (Probable **NON-ANTIGEN**).

>EHO9885942.1 outer membrane assembly protein AsmA [Salmonella enterica subsp. enterica serovar Infantis] Overall Protective Antigen Prediction = **0.7356** (Probable **ANTIGEN**).

>EHO9885943.1 dCTP deaminase [Salmonella enterica subsp. enterica serovar Infantis] Overall Protective Antigen Prediction = **0.5339** (Probable **ANTIGEN**).

>EHO9885944.1 uridine kinase [Salmonella enterica subsp. enterica serovar Infantis] Overall

Protective Antigen Prediction = **0.4357** (Probable **ANTIGEN**).

>EHO9885945.1 MASE1 domain-containing protein [Salmonella enterica subsp. enterica serovar Infantis] Overall Protective Antigen Prediction = **0.4727** (Probable **ANTIGEN**).

>EHO9885946.1 DNA-3-methyladenine glycosylase 2 [Salmonella enterica subsp. enterica serovar Infantis] Overall Protective Antigen Prediction = **0.4222** (Probable **ANTIGEN**).

>EHO9885947.1 molecular chaperone [Salmonella enterica subsp. enterica serovar Infantis] Overall Protective Antigen Prediction = **0.5586** (Probable **ANTIGEN**).

>EHO9885948.1 type I toxin-antitoxin system toxin IbsA [Salmonella enterica subsp. enterica serovar Infantis] Overall Protective Antigen Prediction = **1.0964** (Probable **ANTIGEN**).

>EHO9885949.1 multidrug efflux RND transporter subunit MdtA [Salmonella enterica subsp. enterica serovar Infantis] Overall Protective Antigen Prediction = **0.6599** (Probable **ANTIGEN**).

>EHO9885950.1 multidrug efflux RND transporter permease subunit MdtB [Salmonella enterica subsp. enterica serovar Infantis] Overall Protective Antigen Prediction = **0.4996** (Probable **ANTIGEN**).

>EHO9885951.1 multidrug efflux RND transporter permease subunit MdtC [Salmonella enterica subsp. enterica serovar Infantis] Overall Protective Antigen Prediction = **0.5684** (Probable **ANTIGEN**).

>EHO9885952.1 MFS transporter [Salmonella enterica subsp. enterica serovar Infantis] Overall Protective Antigen Prediction = **0.5349** (Probable **ANTIGEN**).

>EHO9885953.1 two-component system sensor histidine kinase BaeS [Salmonella enterica subsp. enterica serovar Infantis] Overall Protective Antigen Prediction = **0.4279** (Probable **ANTIGEN**).

>EHO9885954.1 two-component system response regulator BaeR [Salmonella enterica subsp. enterica serovar Infantis] Overall Protective Antigen Prediction = **0.3718** (Probable **NON-ANTIGEN**).

>EHO9885955.1 cytoplasmic protein [Salmonella enterica subsp. enterica serovar Infantis] Overall Protective Antigen Prediction = **0.5611** (Probable **ANTIGEN**).

>EHO9885956.1 DUF1266 domain-containing protein [Salmonella enterica subsp. enterica serovar Infantis] Overall Protective Antigen Prediction = **0.3783** (Probable **NON-ANTIGEN**).

>EHO9885957.1 DUF4034 domain-containing protein [Salmonella enterica subsp. enterica serovar Infantis] Overall Protective Antigen Prediction = **0.3930** (Probable **NON-ANTIGEN**).

>EHO9885958.1 tRNA 5-hydroxyuridine modification protein YegQ [Salmonella enterica subsp. enterica serovar Infantis] Overall Protective Antigen Prediction = **0.5043** (Probable **ANTIGEN**).

>EHO9885959.1 type III secretion system effector arginine glycosyltransferase SseK2 [Salmonella enterica subsp. enterica serovar Infantis] Overall Protective Antigen Prediction = **0.1887** (Probable **NON-ANTIGEN**).

>EHO9885960.1 CesT family type III secretion system chaperone [Salmonella enterica subsp. enterica serovar Infantis] Overall Protective Antigen Prediction = **0.4360** (Probable **ANTIGEN**).

>EHO9885961.1 hypothetical protein KND05_001262 [Salmonella enterica subsp. enterica serovar Infantis] Overall Protective Antigen Prediction = **0.5277** (Probable **ANTIGEN**).

>EHO9885962.1 lipid kinase YegS [Salmonella enterica subsp. enterica serovar Infantis] Overall Protective Antigen Prediction = **0.6047** (Probable **ANTIGEN**).

>EHO9885963.1 class I fructose-bisphosphate aldolase [Salmonella enterica subsp. enterica serovar Infantis] Overall Protective Antigen Prediction = **0.4287** (Probable **ANTIGEN**).

>EHO9885964.1 MFS transporter [Salmonella enterica subsp. enterica serovar Infantis] Overall Protective Antigen Prediction = **0.5845** (Probable **ANTIGEN**).

>EHO9885965.1 ADP-ribosylglycohydrolase family protein [Salmonella enterica subsp. enterica serovar Infantis] Overall Protective Antigen Prediction = **0.4023** (Probable **ANTIGEN**).

>EHO9885966.1 sugar kinase [Salmonella enterica subsp. enterica serovar Infantis] Overall Protective Antigen Prediction = **0.3465** (Probable **NON-ANTIGEN**).

>EHO9885967.1 GntR family transcriptional regulator [Salmonella enterica subsp. enterica serovar Infantis] Overall Protective Antigen Prediction = **0.3143** (Probable **NON-ANTIGEN**).

>EHO9885968.1 bifunctional hydroxymethylpyrimidine kinase/phosphomethylpyrimidine kinase [Salmonella enterica subsp. enterica serovar Infantis] Overall Protective Antigen Prediction = **0.5710** (Probable **ANTIGEN**).

>EHO9885969.1 hydroxyethylthiazole kinase [Salmonella enterica subsp. enterica serovar Infantis] Overall Protective Antigen Prediction = **0.5917** (Probable **ANTIGEN**).

>EHO9885970.1 Ni(II)/Co(II) efflux transporter accessory subunit RcnB [Salmonella enterica subsp. enterica serovar Infantis] Overall Protective Antigen Prediction = **0.5173** (Probable **ANTIGEN**).

>EHO9885971.1 type 1 fimbrial protein [Salmonella enterica subsp. enterica serovar Infantis] Overall Protective Antigen Prediction = **0.8530** (Probable **ANTIGEN**).

>EHO9885972.1 fimbrial biogenesis outer membrane usher protein [Salmonella enterica subsp. enterica serovar Infantis] Overall Protective Antigen Prediction = **0.6144** (Probable **ANTIGEN**).

>EHO9885973.1 fimbria/pilus periplasmic chaperone [Salmonella enterica subsp. enterica serovar Infantis] Overall Protective Antigen Prediction = **0.4729** (Probable **ANTIGEN**).

>EHO9885974.1 fimbrial protein [Salmonella enterica subsp. enterica serovar Infantis] Overall Protective Antigen Prediction = **0.6424** (Probable **ANTIGEN**).

>EHO9885975.1 DUF2574 family protein [Salmonella enterica subsp. enterica serovar Infantis] Overall Protective Antigen Prediction = **0.5350** (Probable **ANTIGEN**).

>EHO9885976.1 iron-sulfur cluster carrier protein ApbC [Salmonella enterica subsp. enterica serovar Infantis] Overall Protective Antigen Prediction = **0.5280** (Probable **ANTIGEN**).

>EHO9885977.1 methionine--tRNA ligase [Salmonella enterica subsp. enterica serovar Infantis] Overall Protective Antigen Prediction = **0.4130** (Probable **ANTIGEN**).

>EHO9885978.1 YehR family lipoprotein [Salmonella enterica subsp. enterica serovar Infantis] Overall Protective Antigen Prediction = **0.8987** (Probable **ANTIGEN**).

>EHO9885979.1 YehR family lipoprotein [Salmonella enterica subsp. enterica serovar Infantis] Overall Protective Antigen Prediction = **0.6224** (Probable **ANTIGEN**).

>EHO9885980.1 DUF1456 family protein [Salmonella enterica subsp. enterica serovar Infantis] Overall Protective Antigen Prediction = **0.4058** (Probable **ANTIGEN**).

>EHO9885981.1 two-component system response regulator BtsR [Salmonella enterica subsp. enterica serovar Infantis] Overall Protective Antigen Prediction = **0.3973** (Probable **NON-ANTIGEN**).

>EHO9885982.1 sensor histidine kinase [Salmonella enterica subsp. enterica serovar Infantis] Overall Protective Antigen Prediction = **0.3918** (Probable **NON-ANTIGEN**).

>EHO9885983.1 HTH-type transcriptional regulator MlrA [Salmonella enterica subsp. enterica serovar Infantis] Overall Protective Antigen Prediction = **0.2487** (Probable **NON-ANTIGEN**).

>EHO9885984.1 protein YohO [Salmonella enterica subsp. enterica serovar Infantis] Overall Protective Antigen Prediction = **0.4807** (Probable **ANTIGEN**).

>EHO9885985.1 ABC transporter permease [Salmonella enterica subsp. enterica serovar Infantis] Overall Protective Antigen Prediction = **0.5167** (Probable **ANTIGEN**).

>EHO9885986.1 ABC transporter ATP-binding protein [Salmonella enterica subsp. enterica serovar Infantis] Overall Protective Antigen Prediction = **0.4157** (Probable **ANTIGEN**).

>EHO9885987.1 ABC transporter permease [Salmonella enterica subsp. enterica serovar Infantis] Overall Protective Antigen Prediction = **0.5124** (Probable **ANTIGEN**).

>EHO9885988.1 ABC transporter substrate-binding protein [Salmonella enterica subsp. enterica serovar Infantis] Overall Protective Antigen Prediction = **0.4698** (Probable **ANTIGEN**).

>EHO9885989.1 beta-glucosidase BglX [Salmonella enterica subsp. enterica serovar Infantis] Overall Protective Antigen Prediction = **0.4524** (Probable **ANTIGEN**).

>EHO9885990.1 D-lactate dehydrogenase [Salmonella enterica subsp. enterica serovar Infantis] Overall Protective Antigen Prediction = **0.3670** (Probable **NON-ANTIGEN**).

>EHO9885991.1 D-alanyl-D-alanine endopeptidase [Salmonella enterica subsp. enterica serovar Infantis] Overall Protective Antigen Prediction = **0.4153** (Probable **ANTIGEN**).

>EHO9885992.1 YIP1 family protein [Salmonella enterica subsp. enterica serovar Infantis] Overall Protective Antigen Prediction = **0.3820** (Probable **NON-ANTIGEN**).

>EHO9885993.1 DedA family protein [Salmonella enterica subsp. enterica serovar Infantis] Overall Protective Antigen Prediction = **0.3709** (Probable **NON-ANTIGEN**).

>EHO9885994.1 SDR family oxidoreductase [Salmonella enterica subsp. enterica serovar Infantis] Overall Protective Antigen Prediction = **0.5437** (Probable **ANTIGEN**).

>EHO9885995.1 multidrug resistance outer membrane protein MdtQ [Salmonella enterica subsp. enterica serovar Infantis] Overall Protective Antigen Prediction = **0.6108** (Probable **ANTIGEN**).

>EHO9885996.1 protein YohP [Salmonella enterica subsp. enterica serovar Infantis] Overall Protective Antigen Prediction = **1.1448** (Probable **ANTIGEN**).

>EHO9885997.1 tRNA dihydrouridine(16) synthase DusC [Salmonella enterica subsp. enterica serovar Infantis] Overall Protective Antigen Prediction = **0.3569** (Probable **NON-ANTIGEN**).

>EHO9885998.1 3-hydroxybenzoate 6-monooxygenase [Salmonella enterica subsp. enterica serovar Infantis] Overall Protective Antigen Prediction = **0.3864** (Probable **NON-ANTIGEN**).

>EHO9885999.1 maleylacetoacetate isomerase [Salmonella enterica subsp. enterica serovar Infantis] Overall Protective Antigen Prediction = **0.5219** (Probable **ANTIGEN**).

>EHO9886000.1 fumarylacetoacetate hydrolase family protein [Salmonella enterica subsp. enterica serovar Infantis] Overall Protective Antigen Prediction = **0.4991** (Probable **ANTIGEN**).

>EHO9886001.1 gentisate 1,2-dioxygenase [Salmonella enterica subsp. enterica serovar Infantis] Overall Protective Antigen Prediction = **0.4670** (Probable **ANTIGEN**).

>EHO9886002.1 aromatic acid/H+ symport family MFS transporter [Salmonella enterica subsp. enterica serovar Infantis] Overall Protective Antigen Prediction = **0.5595** (Probable **ANTIGEN**).

>EHO9886003.1 LysR family transcriptional regulator [Salmonella enterica subsp. enterica serovar Infantis] Overall Protective Antigen Prediction = **0.4038** (Probable **ANTIGEN**).

>EHO9886004.1 CidA/LrgA family protein [Salmonella enterica subsp. enterica serovar Infantis] Overall Protective Antigen Prediction = **0.6102** (Probable **ANTIGEN**).

>EHO9886005.1 CidB/LrgB family autolysis modulator [Salmonella enterica subsp. enterica serovar Infantis] Overall Protective Antigen Prediction = **0.5649** (Probable **ANTIGEN**).

>EHO9886006.1 cytidine deaminase [Salmonella enterica subsp. enterica serovar Infantis] Overall Protective Antigen Prediction = **0.5521** (Probable **ANTIGEN**).

>EHO9886007.1 outer membrane permeability protein SanA [Salmonella enterica subsp. enterica serovar Infantis] Overall Protective Antigen Prediction = **0.2416** (Probable **NON-ANTIGEN**).

>EHO9886008.1 DUF2542 family protein [Salmonella enterica subsp. enterica serovar Infantis] Overall Protective Antigen Prediction = **0.6333** (Probable **ANTIGEN**).

>EHO9886009.1 NAD(P)-dependent oxidoreductase [Salmonella enterica subsp. enterica serovar Infantis] Overall Protective Antigen Prediction = **0.4469** (Probable **ANTIGEN**).

>EHO9886010.1 NAD-dependent dihydropyrimidine dehydrogenase subunit PreA [Salmonella enterica subsp. enterica serovar Infantis] Overall Protective Antigen Prediction = **0.3585** (Probable **NON-ANTIGEN**).

>EHO9886011.1 galactose/methyl galactoside ABC transporter permease MglC [Salmonella enterica subsp. enterica serovar Infantis] Overall Protective Antigen Prediction = **0.3704** (Probable **NON-ANTIGEN**).

>EHO9886012.1 galactose/methyl galactoside ABC transporter ATP-binding protein MglA [Salmonella enterica subsp. enterica serovar Infantis] Overall Protective Antigen Prediction = **0.3487** (Probable **NON-ANTIGEN**).

>EHO9886013.1 galactose/glucose ABC transporter substrate-binding protein MglB [Salmonella enterica subsp. enterica serovar Infantis] Overall Protective Antigen Prediction = **0.5606** (Probable

ANTIGEN).

>EHO9886014.1 glutamine--tRNA ligase [Salmonella enterica subsp. enterica serovar Infantis] Overall Protective Antigen Prediction = **0.4864** (Probable **ANTIGEN**).

>EHO9886015.1 PTS N-acetyl glucosamine transporter subunit IIABC [Salmonella enterica subsp. enterica serovar Infantis] Overall Protective Antigen Prediction = **0.5235** (Probable **ANTIGEN**).

>EHO9886016.1 glucosamine-6-phosphate deaminase [Salmonella enterica subsp. enterica serovar Infantis] Overall Protective Antigen Prediction = **0.4546** (Probable **ANTIGEN**).

>EHO9886017.1 N-acetylglucosamine-6-phosphate deacetylase [Salmonella enterica subsp. enterica serovar Infantis] Overall Protective Antigen Prediction = **0.4304** (Probable **ANTIGEN**).

>EHO9886018.1 DNA-binding transcriptional regulator NagC [Salmonella enterica subsp. enterica serovar Infantis] Overall Protective Antigen Prediction = **0.3361** (Probable **NON-ANTIGEN**).

>EHO9886019.1 ribonucleotide monophosphatase NagD [Salmonella enterica subsp. enterica serovar Infantis] Overall Protective Antigen Prediction = **0.3554** (Probable **NON-ANTIGEN**).

>EHO9886020.1 asparagine synthase B [Salmonella enterica subsp. enterica serovar Infantis] Overall Protective Antigen Prediction = **0.4886** (Probable **ANTIGEN**).

>EHO9886021.1 hypothetical protein KND05_001329 [Salmonella enterica subsp. enterica serovar Infantis] Overall Protective Antigen Prediction = **0.7439** (Probable **ANTIGEN**).

>EHO9886022.1 2-octaprenyl-3-methyl-6-methoxy-1,4-benzoquinol hydroxylase [Salmonella enterica subsp. enterica serovar Infantis] Overall Protective Antigen Prediction = **0.4295** (Probable **ANTIGEN**).

>EHO9886023.1 tRNA (N6-isopentenyl adenosine(37)-C2)-methylthiotransferase MiaB [Salmonella enterica subsp. enterica serovar Infantis] Overall Protective Antigen Prediction = **0.4641** (Probable **ANTIGEN**).

>EHO9886024.1 PhoH family protein [Salmonella enterica subsp. enterica serovar Infantis] Overall Protective Antigen Prediction = **0.4416** (Probable **ANTIGEN**).

>EHO9886025.1 rRNA maturation RNase YbeY [Salmonella enterica subsp. enterica serovar Infantis] Overall Protective Antigen Prediction = **0.5618** (Probable **ANTIGEN**).

>EHO9886026.1 CNNM family magnesium/cobalt transport protein CorC [Salmonella enterica subsp. enterica serovar Infantis] Overall Protective Antigen Prediction = **0.3504** (Probable **NON-ANTIGEN**).

>EHO9886027.1 apolipoprotein N-acyltransferase [Salmonella enterica subsp. enterica serovar Infantis] Overall Protective Antigen Prediction = **0.3348** (Probable **NON-ANTIGEN**).

>EHO9886028.1 amino acid ABC transporter substrate-binding protein [Salmonella enterica subsp. enterica serovar Infantis] Overall Protective Antigen Prediction = **0.4386** (Probable **ANTIGEN**).

>EHO9886029.1 glutamate/aspartate ABC transporter permease GltJ [Salmonella enterica subsp. enterica serovar Infantis] Overall Protective Antigen Prediction = **0.3443** (Probable **NON-ANTIGEN**).

>EHO9886030.1 glutamate/aspartate ABC transporter permease GltK [Salmonella enterica subsp. enterica serovar Infantis] Overall Protective Antigen Prediction = **0.4532** (Probable **ANTIGEN**).

>EHO9886031.1 glutamate/aspartate ABC transporter ATP binding protein GltL [Salmonella enterica subsp. enterica serovar Infantis] Overall Protective Antigen Prediction = **0.3245** (Probable **NON-ANTIGEN**).

>EHO9886032.1 pyrimidine-specific ribonucleoside hydrolase RihA [Salmonella enterica subsp. enterica serovar Infantis] Overall Protective Antigen Prediction = **0.4783** (Probable **ANTIGEN**).

>EHO9886033.1 hypothetical protein KND05_001341 [Salmonella enterica subsp. enterica serovar Infantis] Overall Protective Antigen Prediction = **0.4973** (Probable **ANTIGEN**).

>EHO9886034.1 molecular chaperone HscC [Salmonella enterica subsp. enterica serovar Infantis] Overall Protective Antigen Prediction = **0.4885** (Probable **ANTIGEN**).

>EHO9886035.1 J domain-containing protein [Salmonella enterica subsp. enterica serovar Infantis] Overall Protective Antigen Prediction = **0.4520** (Probable **ANTIGEN**).

>EHO9886036.1 DUF1266 domain-containing protein [Salmonella enterica subsp. enterica serovar Infantis] Overall Protective Antigen Prediction = **0.4047** (Probable **ANTIGEN**).

>EHO9886037.1 J domain-containing protein [Salmonella enterica subsp. enterica serovar Infantis] Overall Protective Antigen Prediction = **0.4461** (Probable **ANTIGEN**).

>EHO9886038.1 DUF1266 domain-containing protein [Salmonella enterica subsp. enterica serovar Infantis] Overall Protective Antigen Prediction = **0.3819** (Probable **NON-ANTIGEN**).

>EHO9886039.1 sel1 repeat family protein [Salmonella enterica subsp. enterica serovar Infantis] Overall Protective Antigen Prediction = **0.5623** (Probable **ANTIGEN**).

>EHO9886040.1 zinc ribbon-containing protein [Salmonella enterica subsp. enterica serovar Infantis] Overall Protective Antigen Prediction = **0.3101** (Probable **NON-ANTIGEN**).

>EHO9886041.1 sigma-54-dependent transcriptional regulator [Salmonella enterica subsp. enterica serovar Infantis] Overall Protective Antigen Prediction = **0.3630** (Probable **NON-ANTIGEN**).

>EHO9886042.1 2-keto-3-deoxygluconate permease 2 [Salmonella enterica subsp. enterica serovar Infantis] Overall Protective Antigen Prediction = **0.5080** (Probable **ANTIGEN**).

>EHO9886043.1 UxaA family hydrolase [Salmonella enterica subsp. enterica serovar Infantis] Overall Protective Antigen Prediction = **0.4962** (Probable **ANTIGEN**).

>EHO9886044.1 UxaA family hydrolase [Salmonella enterica subsp. enterica serovar Infantis] Overall Protective Antigen Prediction = **0.4745** (Probable **ANTIGEN**).

>EHO9886045.1 leucine--tRNA ligase [Salmonella enterica subsp. enterica serovar Infantis] Overall Protective Antigen Prediction = **0.4761** (Probable **ANTIGEN**).

>EHO9886046.1 LPS assembly lipoprotein LptE [Salmonella enterica subsp. enterica serovar Infantis] Overall Protective Antigen Prediction = **0.5083** (Probable **ANTIGEN**).

>EHO9886047.1 DNA polymerase III subunit delta [Salmonella enterica subsp. enterica serovar Infantis] Overall Protective Antigen Prediction = **0.2441** (Probable **NON-ANTIGEN**).

>EHO9886048.1 nicotinate-nucleotide adenylyltransferase [Salmonella enterica subsp. enterica serovar Infantis] Overall Protective Antigen Prediction = **0.2500** (Probable **NON-ANTIGEN**).

>EHO9886049.1 threonine-phosphate decarboxylase [Salmonella enterica subsp. enterica serovar Infantis] Overall Protective Antigen Prediction = **0.3616** (Probable **NON-ANTIGEN**).

>EHO9886050.1 adenosylcobalamin/alpha-ribazole phosphatase [Salmonella enterica subsp. enterica serovar Infantis] Overall Protective Antigen Prediction = **0.3329** (Probable **NON-ANTIGEN**).

>EHO9886051.1 ribosome silencing factor [Salmonella enterica subsp. enterica serovar Infantis] Overall Protective Antigen Prediction = **0.3968** (Probable **NON-ANTIGEN**).

>EHO9886052.1 23S rRNA (pseudouridine(1915)-N(3))-methyltransferase RlmH [Salmonella enterica subsp. enterica serovar Infantis] Overall Protective Antigen Prediction = **0.3689** (Probable **NON-ANTIGEN**).

>EHO9886053.1 peptidoglycan DD-transpeptidase MrdA [Salmonella enterica subsp. enterica serovar Infantis] Overall Protective Antigen Prediction = **0.3648** (Probable **NON-ANTIGEN**).

>EHO9886054.1 peptidoglycan glycosyltransferase MrdB [Salmonella enterica subsp. enterica serovar Infantis] Overall Protective Antigen Prediction = **0.6269** (Probable **ANTIGEN**).

>EHO9886055.1 endolytic peptidoglycan transglycosylase RlpA [Salmonella enterica subsp. enterica serovar Infantis] Overall Protective Antigen Prediction = **0.6132** (Probable **ANTIGEN**).

>EHO9886056.1 D-alanyl-D-alanine carboxypeptidase DacA [Salmonella enterica subsp. enterica serovar Infantis] Overall Protective Antigen Prediction = **0.5255** (Probable **ANTIGEN**).

>EHO9886057.1 YbeD family protein [Salmonella enterica subsp. enterica serovar Infantis] Overall Protective Antigen Prediction = **0.3717** (Probable **NON-ANTIGEN**).

>EHO9886058.1 lipoyl(octanoyl) transferase LipB [Salmonella enterica subsp. enterica serovar Infantis] Overall Protective Antigen Prediction = **0.5710** (Probable **ANTIGEN**).

>EHO9886059.1 DNA-binding transcriptional regulator [Salmonella enterica subsp. enterica serovar Infantis] Overall Protective Antigen Prediction = **0.3785** (Probable **NON-ANTIGEN**).

>EHO9886060.1 lipoyl synthase [Salmonella enterica subsp. enterica serovar Infantis] Overall Protective Antigen Prediction = **0.3953** (Probable **NON-ANTIGEN**).

>EHO9886061.1 twin-arginine translocase subunit TatE [Salmonella enterica subsp. enterica serovar Infantis] Overall Protective Antigen Prediction = **0.6249** (Probable **ANTIGEN**).

>EHO9886062.1 deaminated glutathione amidase [Salmonella enterica subsp. enterica serovar Infantis] Overall Protective Antigen Prediction = **0.4257** (Probable **ANTIGEN**).

>EHO9886063.1 fluoride efflux transporter CrcB [Salmonella enterica subsp. enterica serovar Infantis] Overall Protective Antigen Prediction = **0.4603** (Probable **ANTIGEN**).

>EHO9886064.1 transcription antiterminator/RNA stability regulator CspE [Salmonella enterica subsp. enterica serovar Infantis] Overall Protective Antigen Prediction = **0.3698** (Probable **NON-ANTIGEN**).

>EHO9886065.1 lipid IV(A) palmitoyltransferase PagP [Salmonella enterica subsp. enterica serovar Infantis] Overall Protective Antigen Prediction = **0.6211** (Probable **ANTIGEN**).

>EHO9886066.1 anaerobic C4-dicarboxylate transporter DcuC [Salmonella enterica subsp. enterica serovar Infantis] Overall Protective Antigen Prediction = **0.4307** (Probable **ANTIGEN**).

>EHO9886067.1 two-component response regulator DpiA [Salmonella enterica subsp. enterica serovar Infantis] Overall Protective Antigen Prediction = **0.2856** (Probable **NON-ANTIGEN**).

>EHO9886068.1 sensor histidine kinase DpiB [Salmonella enterica subsp. enterica serovar Infantis] Overall Protective Antigen Prediction = **0.3506** (Probable **NON-ANTIGEN**).

>EHO9886069.1 [citrate (pro-3S)-lyase] ligase [Salmonella enterica subsp. enterica serovar Infantis] Overall Protective Antigen Prediction = **0.3626** (Probable **NON-ANTIGEN**).

>EHO9886070.1 citrate lyase acyl carrier protein [Salmonella enterica subsp. enterica serovar Infantis] Overall Protective Antigen Prediction = **0.7041** (Probable **ANTIGEN**).

>EHO9886071.1 citrate (pro-3S)-lyase subunit beta [Salmonella enterica subsp. enterica serovar Infantis] Overall Protective Antigen Prediction = **0.3712** (Probable **NON-ANTIGEN**).

>EHO9886072.1 citrate lyase subunit alpha [Salmonella enterica subsp. enterica serovar Infantis] Overall Protective Antigen Prediction = **0.4916** (Probable **ANTIGEN**).

>EHO9886073.1 citrate lyase holo-[acyl-carrier protein] synthase [Salmonella enterica subsp. enterica serovar Infantis] Overall Protective Antigen Prediction = **0.4662** (Probable **ANTIGEN**).

>EHO9886074.1 triphosphoribosyl-dephospho-CoA synthase CitG [Salmonella enterica subsp. enterica serovar Infantis] Overall Protective Antigen Prediction = **0.5730** (Probable **ANTIGEN**).

>EHO9886075.1 citrate/succinate antiporter CitT [Salmonella enterica subsp. enterica serovar Infantis] Overall Protective Antigen Prediction = **0.5075** (Probable **ANTIGEN**).

>EHO9886076.1 ribonuclease I [Salmonella enterica subsp. enterica serovar Infantis] Overall Protective Antigen Prediction = **0.5462** (Probable **ANTIGEN**).

>EHO9886077.1 nucleoside diphosphate kinase regulator [Salmonella enterica subsp. enterica serovar Infantis] Overall Protective Antigen Prediction = **0.6030** (Probable **ANTIGEN**).

>EHO9886078.1 glutathione-dependent formaldehyde dehydrogenase [Salmonella enterica subsp. enterica serovar Infantis] Overall Protective Antigen Prediction = **0.4986** (Probable **ANTIGEN**).

>EHO9886079.1 universal stress protein UspG [Salmonella enterica subsp. enterica serovar Infantis] Overall Protective Antigen Prediction = **0.5388** (Probable **ANTIGEN**).

>EHO9886080.1 dimethyl sulfoxide reductase anchor subunit family protein [Salmonella enterica subsp. enterica serovar Infantis] Overall Protective Antigen Prediction = **0.4536** (Probable **ANTIGEN**).

>EHO9886081.1 4Fe-4S dicluster domain-containing protein [Salmonella enterica subsp. enterica serovar Infantis] Overall Protective Antigen Prediction = **0.3837** (Probable **NON-ANTIGEN**).

>EHO9886082.1 molybdopterin-dependent oxidoreductase [Salmonella enterica subsp. enterica serovar Infantis] Overall Protective Antigen Prediction = **0.4953** (Probable **ANTIGEN**).

>EHO9886083.1 molecular chaperone [Salmonella enterica subsp. enterica serovar Infantis] Overall Protective Antigen Prediction = **0.3659** (Probable **NON-ANTIGEN**).

>EHO9886084.1 alkyl hydroperoxide reductase subunit F [Salmonella enterica subsp. enterica serovar Infantis] Overall Protective Antigen Prediction = **0.5415** (Probable **ANTIGEN**).

>EHO9886085.1 alkyl hydroperoxide reductase subunit C [Salmonella enterica subsp. enterica serovar Infantis] Overall Protective Antigen Prediction = **0.4580** (Probable **ANTIGEN**).

>EHO9886086.1 thiol:disulfide interchange protein DsbG [Salmonella enterica subsp. enterica serovar Infantis] Overall Protective Antigen Prediction = **0.1863** (Probable **NON-ANTIGEN**).

>EHO9886087.1 LysR family transcriptional regulator [Salmonella enterica subsp. enterica serovar Infantis] Overall Protective Antigen Prediction = **0.4692** (Probable **ANTIGEN**).

>EHO9886088.1 phosphoadenosine phosphosulfate reductase [Salmonella enterica subsp. enterica serovar Infantis] Overall Protective Antigen Prediction = **0.3696** (Probable **NON-ANTIGEN**).

>EHO9886089.1 ParB-like nuclease domain-containing protein [Salmonella enterica subsp. enterica serovar Infantis] Overall Protective Antigen Prediction = **0.2443** (Probable **NON-ANTIGEN**).

>EHO9886090.1 pyridoxal phosphate-dependent aminotransferase [Salmonella enterica subsp. enterica serovar Infantis] Overall Protective Antigen Prediction = **0.3715** (Probable **NON-ANTIGEN**).

>EHO9886091.1 oxidoreductase [Salmonella enterica subsp. enterica serovar Infantis] Overall Protective Antigen Prediction = **0.3024** (Probable **NON-ANTIGEN**).

>EHO9886092.1 YbdD/YjiX family protein [Salmonella enterica subsp. enterica serovar Infantis] Overall Protective Antigen Prediction = **0.3770** (Probable **NON-ANTIGEN**).

>EHO9886093.1 carbon starvation protein CstA [Salmonella enterica subsp. enterica serovar Infantis] Overall Protective Antigen Prediction = **0.5218** (Probable **ANTIGEN**).

>EHO9886094.1 proofreading thioesterase EntH [Salmonella enterica subsp. enterica serovar Infantis] Overall Protective Antigen Prediction = **0.6557** (Probable **ANTIGEN**).

>EHO9886095.1 2,3-dihydro-2,3-dihydroxybenzoate dehydrogenase EntA [Salmonella enterica subsp. enterica serovar Infantis] Overall Protective Antigen Prediction = **0.4562** (Probable **ANTIGEN**).

>EHO9886096.1 isochorismatase [Salmonella enterica subsp. enterica serovar Infantis] Overall Protective Antigen Prediction = **0.3115** (Probable **NON-ANTIGEN**).

>EHO9886097.1 (2,3-dihydroxybenzoyl)adenylate synthase EntE [Salmonella enterica subsp. enterica serovar Infantis] Overall Protective Antigen Prediction = **0.3879** (Probable **NON-ANTIGEN**).

>EHO9886098.1 isochorismate synthase EntC [Salmonella enterica subsp. enterica serovar Infantis] Overall Protective Antigen Prediction = **0.3707** (Probable **NON-ANTIGEN**).

>EHO9886099.1 Fe2+-enterobactin ABC transporter substrate-binding protein [Salmonella enterica subsp. enterica serovar Infantis] Overall Protective Antigen Prediction = **0.5505** (Probable **ANTIGEN**).

>EHO9886100.1 enterobactin transporter EntS [Salmonella enterica subsp. enterica serovar Infantis] Overall Protective Antigen Prediction = **0.3651** (Probable **NON-ANTIGEN**).

>EHO9886101.1 Fe(3+)-siderophore ABC transporter permease [Salmonella enterica subsp. enterica serovar Infantis] Overall Protective Antigen Prediction = **0.4219** (Probable **ANTIGEN**).

>EHO9886102.1 iron-enterobactin ABC transporter permease [Salmonella enterica subsp. enterica serovar Infantis] Overall Protective Antigen Prediction = **0.3402** (Probable **NON-ANTIGEN**).

>EHO9886103.1 iron-enterobactin ABC transporter ATP-binding protein [Salmonella enterica subsp. enterica serovar Infantis] Overall Protective Antigen Prediction = **0.2593** (Probable **NON-ANTIGEN**).

>EHO9886104.1 LPS O-antigen length regulator [Salmonella enterica subsp. enterica serovar Infantis] Overall Protective Antigen Prediction = **0.5304** (Probable **ANTIGEN**).

>EHO9886105.1 enterobactin non-ribosomal peptide synthetase EntF [Salmonella enterica subsp. enterica serovar Infantis] Overall Protective Antigen Prediction = **0.4533** (Probable **ANTIGEN**).

>EHO9886106.1 MbtH family protein [Salmonella enterica subsp. enterica serovar Infantis] Overall Protective Antigen Prediction = **0.2609** (Probable **NON-ANTIGEN**).

>EHO9886107.1 enterochelin esterase [Salmonella enterica subsp. enterica serovar Infantis] Overall Protective Antigen Prediction = **0.4157** (Probable **ANTIGEN**).

>EHO9886108.1 TonB-dependent siderophore receptor [Salmonella enterica subsp. enterica serovar Infantis] Overall Protective Antigen Prediction = **0.7591** (Probable **ANTIGEN**).

>EHO9886109.1 enterobactin synthase subunit EntD [Salmonella enterica subsp. enterica serovar Infantis] Overall Protective Antigen Prediction = **0.3984** (Probable **NON-ANTIGEN**).

>EHO9886110.1 glutamate--cysteine ligase [Salmonella enterica subsp. enterica serovar Infantis] Overall Protective Antigen Prediction = **0.4246** (Probable **ANTIGEN**).

>EHO9886111.1 DUF1158 family protein [Salmonella enterica subsp. enterica serovar Infantis] Overall Protective Antigen Prediction = **0.7209** (Probable **ANTIGEN**).

>EHO9886112.1 RamA family antibiotic efflux transcriptional regulator [Salmonella enterica subsp. enterica serovar Infantis] Overall Protective Antigen Prediction = **0.3674** (Probable **NON-ANTIGEN**).

>EHO9886113.1 TetR/AcrR family transcriptional regulator [Salmonella enterica subsp. enterica serovar Infantis] Overall Protective Antigen Prediction = **0.3113** (Probable **NON-ANTIGEN**).

>EHO9886114.1 MmcQ/YjbR family DNA-binding protein [Salmonella enterica subsp. enterica serovar Infantis] Overall Protective Antigen Prediction = **0.5360** (Probable **ANTIGEN**).

>EHO9886115.1 autotransporter domain-containing esterase [Salmonella enterica subsp. enterica serovar Infantis] Overall Protective Antigen Prediction = **0.6395** (Probable **ANTIGEN**).

>EHO9886116.1 mechanosensitive ion channel family protein [Salmonella enterica subsp. enterica serovar Infantis] Overall Protective Antigen Prediction = **0.4473** (Probable **ANTIGEN**).

>EHO9886117.1 phenylalanine transporter [Salmonella enterica subsp. enterica serovar Infantis] Overall Protective Antigen Prediction = **0.6520** (Probable **ANTIGEN**).

>EHO9886118.1 DNA repair protein [Salmonella enterica subsp. enterica serovar Infantis] Overall Protective Antigen Prediction = **0.3689** (Probable **NON-ANTIGEN**).

>EHO9886119.1 YkgB family protein [Salmonella enterica subsp. enterica serovar Infantis] Overall Protective Antigen Prediction = **0.2667** (Probable **NON-ANTIGEN**).

>EHO9886120.1 DUF1471 domain-containing protein [Salmonella enterica subsp. enterica serovar Infantis] Overall Protective Antigen Prediction = **0.3883** (Probable **NON-ANTIGEN**).

>EHO9886121.1 pyridine nucleotide-disulfide oxidoreductase [Salmonella enterica subsp. enterica serovar Infantis] Overall Protective Antigen Prediction = **0.3174** (Probable **NON-ANTIGEN**).

>EHO9886122.1 cupin domain-containing protein [Salmonella enterica subsp. enterica serovar Infantis] Overall Protective Antigen Prediction = **0.3598** (Probable **NON-ANTIGEN**).

>EHO9886123.1 copper-binding protein [Salmonella enterica subsp. enterica serovar Infantis] Overall Protective Antigen Prediction = **0.3895** (Probable **NON-ANTIGEN**).

>EHO9886124.1 heme lyase CcmF/NrfE family subunit, partial [Salmonella enterica subsp. enterica serovar Infantis] Overall Protective Antigen Prediction = **0.4534** (Probable **ANTIGEN**).

>EHO9886125.1 thiol:disulfide interchange protein DsbE [Salmonella enterica subsp. enterica serovar Infantis] Overall Protective Antigen Prediction = **0.5948** (Probable **ANTIGEN**).

>EHO9886126.1 cytochrome c-type biogenesis protein CcmH [Salmonella enterica subsp. enterica serovar Infantis] Overall Protective Antigen Prediction = **0.5211** (Probable **ANTIGEN**).

>EHO9886127.1 DUF3748 domain-containing protein [Salmonella enterica subsp. enterica serovar Infantis] Overall Protective Antigen Prediction = **0.4408** (Probable **ANTIGEN**).

>EHO9886128.1 YceK/YidQ family lipoprotein [Salmonella enterica subsp. enterica serovar Infantis] Overall Protective Antigen Prediction = **0.7329** (Probable **ANTIGEN**).

>EHO9886129.1 heat shock chaperone IbpA [Salmonella enterica subsp. enterica serovar Infantis] Overall Protective Antigen Prediction = **0.5502** (Probable **ANTIGEN**).

>EHO9886130.1 heat shock chaperone IbpB [Salmonella enterica subsp. enterica serovar Infantis] Overall Protective Antigen Prediction = **0.5773** (Probable **ANTIGEN**).

>EHO9886131.1 putative transporter [Salmonella enterica subsp. enterica serovar Infantis] Overall Protective Antigen Prediction = **0.5752** (Probable **ANTIGEN**).

>EHO9886132.1 YidH family protein [Salmonella enterica subsp. enterica serovar Infantis] Overall Protective Antigen Prediction = **0.3096** (Probable **NON-ANTIGEN**).

>EHO9886133.1 DUF202 domain-containing protein [Salmonella enterica subsp. enterica serovar Infantis] Overall Protective Antigen Prediction = **0.4516** (Probable **ANTIGEN**).

>EHO9886134.1 radical SAM protein [Salmonella enterica subsp. enterica serovar Infantis] Overall Protective Antigen Prediction = **0.3809** (Probable **NON-ANTIGEN**).

>EHO9886135.1 D-serine ammonia-lyase [Salmonella enterica subsp. enterica serovar Infantis] Overall Protective Antigen Prediction = **0.4750** (Probable **ANTIGEN**).

>EHO9886136.1 D-serine transporter DsdX [Salmonella enterica subsp. enterica serovar Infantis] Overall Protective Antigen Prediction = **0.5148** (Probable **ANTIGEN**).

>EHO9886137.1 DNA-binding transcriptional regulator DsdC [Salmonella enterica subsp. enterica serovar Infantis] Overall Protective Antigen Prediction = **0.3859** (Probable **NON-ANTIGEN**).

>EHO9886138.1 cellulase family glycosylhydrolase [Salmonella enterica subsp. enterica serovar Infantis] Overall Protective Antigen Prediction = **0.5629** (Probable **ANTIGEN**).

>EHO9886139.1 multidrug efflux MFS transporter EmrD [Salmonella enterica subsp. enterica serovar Infantis] Overall Protective Antigen Prediction = **0.5057** (Probable **ANTIGEN**).

>EHO9886140.1 EamA family transporter [Salmonella enterica subsp. enterica serovar Infantis] Overall Protective Antigen Prediction = **0.5137** (Probable **ANTIGEN**).

>EHO9886141.1 type I toxin-antitoxin system toxin TisB [Salmonella enterica subsp. enterica serovar Infantis] Overall Protective Antigen Prediction = **0.4519** (Probable **ANTIGEN**).

>EHO9886142.1 ilvB operon leader peptide IvbL [Salmonella enterica subsp. enterica serovar Infantis] Overall Protective Antigen Prediction = **0.2972** (Probable **NON-ANTIGEN**).

>EHO9886143.1 acetolactate synthase large subunit [Salmonella enterica subsp. enterica serovar Infantis] Overall Protective Antigen Prediction = **0.3536** (Probable **NON-ANTIGEN**).

>EHO9886144.1 acetolactate synthase small subunit [Salmonella enterica subsp. enterica serovar Infantis] Overall Protective Antigen Prediction = **0.3303** (Probable **NON-ANTIGEN**).

>EHO9886145.1 DeoR family transcriptional regulator [Salmonella enterica subsp. enterica serovar Infantis] Overall Protective Antigen Prediction = **0.2889** (Probable **NON-ANTIGEN**).

>EHO9886146.1 ribokinase [Salmonella enterica subsp. enterica serovar Infantis] Overall Protective Antigen Prediction = **0.5167** (Probable **ANTIGEN**).

>EHO9886147.1 L-fucose:H+ symporter permease [Salmonella enterica subsp. enterica serovar Infantis] Overall Protective Antigen Prediction = **0.5024** (Probable **ANTIGEN**).

>EHO9886148.1 aldose 1-epimerase family protein [Salmonella enterica subsp. enterica serovar Infantis] Overall Protective Antigen Prediction = **0.4377** (Probable **ANTIGEN**).

>EHO9886149.1 transcriptional regulator UhpA [Salmonella enterica subsp. enterica serovar Infantis] Overall Protective Antigen Prediction = **0.5584** (Probable **ANTIGEN**).

>EHO9886150.1 signal transduction histidine-protein kinase/phosphatase UhpB [Salmonella enterica subsp. enterica serovar Infantis] Overall Protective Antigen Prediction = **0.5438** (Probable **ANTIGEN**).

>EHO9886151.1 MFS transporter [Salmonella enterica subsp. enterica serovar Infantis] Overall Protective Antigen Prediction = **0.4021** (Probable **ANTIGEN**).

>EHO9886152.1 hexose-6-phosphate:phosphate antiporter [Salmonella enterica subsp. enterica serovar Infantis] Overall Protective Antigen Prediction = **0.5070** (Probable **ANTIGEN**).

>EHO9886153.1 DUF1198 domain-containing protein [Salmonella enterica subsp. enterica serovar Infantis] Overall Protective Antigen Prediction = **0.5019** (Probable **ANTIGEN**).

>EHO9886154.1 GntR family transcriptional regulator [Salmonella enterica subsp. enterica serovar Infantis] Overall Protective Antigen Prediction = **0.0963** (Probable **NON-ANTIGEN**).

>EHO9886155.1 PTS sugar transporter subunit IIA [Salmonella enterica subsp. enterica serovar Infantis] Overall Protective Antigen Prediction = **0.3057** (Probable **NON-ANTIGEN**).

>EHO9886156.1 PTS sugar transporter subunit IIB [Salmonella enterica subsp. enterica serovar Infantis] Overall Protective Antigen Prediction = **0.3527** (Probable **NON-ANTIGEN**).

>EHO9886157.1 PTS galactitol transporter subunit IIC [Salmonella enterica subsp. enterica serovar Infantis] Overall Protective Antigen Prediction = **0.4105** (Probable **ANTIGEN**).

>EHO9886158.1 carbohydrate kinase [Salmonella enterica subsp. enterica serovar Infantis] Overall Protective Antigen Prediction = **0.4908** (Probable **ANTIGEN**).

>EHO9886159.1 class II aldolase [Salmonella enterica subsp. enterica serovar Infantis] Overall Protective Antigen Prediction = **0.2685** (Probable **NON-ANTIGEN**).

>EHO9886160.1 HPr family phosphocarrier protein [Salmonella enterica subsp. enterica serovar Infantis] Overall Protective Antigen Prediction = **0.3107** (Probable **NON-ANTIGEN**).

>EHO9886161.1 DNA-binding transcriptional regulator [Salmonella enterica subsp. enterica serovar Infantis] Overall Protective Antigen Prediction = **0.4756** (Probable **ANTIGEN**).

>EHO9886162.1 type II toxin-antitoxin system RelE/ParE family toxin [Salmonella enterica subsp. enterica serovar Infantis] Overall Protective Antigen Prediction = **0.3694** (Probable **NON-ANTIGEN**).

>EHO9886163.1 purine ribonucleoside efflux pump NepI [Salmonella enterica subsp. enterica serovar Infantis] Overall Protective Antigen Prediction = **0.4137** (Probable **ANTIGEN**).

>EHO9886164.1 glycoside hydrolase family 1 protein [Salmonella enterica subsp. enterica serovar Infantis] Overall Protective Antigen Prediction = **0.4482** (Probable **ANTIGEN**).

>EHO9886165.1 hypothetical protein KND05_001477 [Salmonella enterica subsp. enterica serovar Infantis] Overall Protective Antigen Prediction = **0.0880** (Probable **NON-ANTIGEN**).

>EHO9886166.1 transcriptional regulator DagR [Salmonella enterica subsp. enterica serovar Infantis] Overall Protective Antigen Prediction = **0.3313** (Probable **NON-ANTIGEN**).

>EHO9886167.1 PTS sugar transporter subunit IIA [Salmonella enterica subsp. enterica serovar Infantis] Overall Protective Antigen Prediction = **0.2383** (Probable **NON-ANTIGEN**).

>EHO9886168.1 PTS system mannose/fructose/N-acetylgalactosamine-transporter subunit IIB [Salmonella enterica subsp. enterica serovar Infantis] Overall Protective Antigen Prediction = **0.4799** (Probable **ANTIGEN**).

>EHO9886169.1 PTS sugar transporter subunit IIC [Salmonella enterica subsp. enterica serovar Infantis] Overall Protective Antigen Prediction = **0.5315** (Probable **ANTIGEN**).

>EHO9886170.1 PTS system mannose/fructose/sorbose family transporter subunit IID [Salmonella enterica subsp. enterica serovar Infantis] Overall Protective Antigen Prediction = **0.4859** (Probable **ANTIGEN**).

>EHO9886171.1 D-glucosaminate-6-phosphate ammonia lyase [Salmonella enterica subsp. enterica serovar Infantis] Overall Protective Antigen Prediction = **0.4641** (Probable **ANTIGEN**).

>EHO9886172.1 2-dehydro-3-deoxy-phosphogluconate aldolase [Salmonella enterica subsp. enterica serovar Infantis] Overall Protective Antigen Prediction = **0.5058** (Probable **ANTIGEN**).

>EHO9886173.1 Rpn family recombination-promoting nuclease/putative transposase [Salmonella enterica subsp. enterica serovar Infantis] Overall Protective Antigen Prediction = **0.4906** (Probable **ANTIGEN**).

>EHO9886174.1 EamA family transporter [Salmonella enterica subsp. enterica serovar Infantis] Overall Protective Antigen Prediction = **0.3906** (Probable **NON-ANTIGEN**).

>EHO9886175.1 MgtC family protein [Salmonella enterica subsp. enterica serovar Infantis] Overall Protective Antigen Prediction = **0.5117** (Probable **ANTIGEN**).

>EHO9886176.1 magnesium-translocating P-type ATPase [Salmonella enterica subsp. enterica serovar Infantis] Overall Protective Antigen Prediction = **0.3945** (Probable **NON-ANTIGEN**).

>EHO9886177.1 protein MgtR [Salmonella enterica subsp. enterica serovar Infantis] Overall Protective Antigen Prediction = **1.3915** (Probable **ANTIGEN**).

>EHO9886178.1 hypothetical protein KND05_001490 [Salmonella enterica subsp. enterica serovar Infantis] Overall Protective Antigen Prediction = **0.8813** (Probable **ANTIGEN**).

>EHO9886179.1 isochorismatase family protein [Salmonella enterica subsp. enterica serovar Infantis] Overall Protective Antigen Prediction = **0.2283** (Probable **NON-ANTIGEN**).

>EHO9886180.1 hypothetical protein KND05_001492 [Salmonella enterica subsp. enterica serovar Infantis] Overall Protective Antigen Prediction = **0.6846** (Probable **ANTIGEN**).

>EHO9886181.1 transcriptional regulator [Salmonella enterica subsp. enterica serovar Infantis] Overall Protective Antigen Prediction = **0.3817** (Probable **NON-ANTIGEN**).

>EHO9886182.1 hypothetical protein KND05_001494 [Salmonella enterica subsp. enterica serovar Infantis] Overall Protective Antigen Prediction = **0.2905** (Probable **NON-ANTIGEN**).

>EHO9886183.1 intestinal colonization autotransporter adhesin MisL [Salmonella enterica subsp. enterica serovar Infantis] Overall Protective Antigen Prediction = **0.7005** (Probable **ANTIGEN**).

>EHO9886184.1 helix-turn-helix transcriptional regulator [Salmonella enterica subsp. enterica serovar Infantis] Overall Protective Antigen Prediction = **0.2706** (Probable **NON-ANTIGEN**).

>EHO9886185.1 glycoside-pentoside-hexuronide family transporter [Salmonella enterica subsp. enterica serovar Infantis] Overall Protective Antigen Prediction = **0.4640** (Probable **ANTIGEN**).

>EHO9886186.1 alpha-xylosidase [Salmonella enterica subsp. enterica serovar Infantis] Overall Protective Antigen Prediction = **0.5301** (Probable **ANTIGEN**).

>EHO9886187.1 AsmA family protein [Salmonella enterica subsp. enterica serovar Infantis] Overall

Protective Antigen Prediction = **0.6444** (Probable **ANTIGEN**).

>EHO9886188.1 xanthine/proton symporter XanP [Salmonella enterica subsp. enterica serovar Infantis] Overall Protective Antigen Prediction = **0.4059** (Probable **ANTIGEN**).

>EHO9886189.1 sodium/glutamate symporter [Salmonella enterica subsp. enterica serovar Infantis] Overall Protective Antigen Prediction = **0.4409** (Probable **ANTIGEN**).

>EHO9886190.1 ATP-dependent DNA helicase RecG [Salmonella enterica subsp. enterica serovar Infantis] Overall Protective Antigen Prediction = **0.4144** (Probable **ANTIGEN**).

>EHO9886191.1 tRNA (guanosine(18)-2'-O)-methyltransferase TrmH [Salmonella enterica subsp. enterica serovar Infantis] Overall Protective Antigen Prediction = **0.3484** (Probable **NON-ANTIGEN**).

>EHO9886192.1 bifunctional GTP diphosphokinase/guanosine-3',5'-bis pyrophosphate 3'-pyrophosphohydrolase [Salmonella enterica subsp. enterica serovar Infantis] Overall Protective Antigen Prediction = **0.4628** (Probable **ANTIGEN**).

>EHO9886193.1 DNA-directed RNA polymerase subunit omega [Salmonella enterica subsp. enterica serovar Infantis] Overall Protective Antigen Prediction = **0.5834** (Probable **ANTIGEN**).

>EHO9886194.1 guanylate kinase [Salmonella enterica subsp. enterica serovar Infantis] Overall Protective Antigen Prediction = **0.4600** (Probable **ANTIGEN**).

>EHO9886195.1 NAD-dependent DNA ligase LigB [Salmonella enterica subsp. enterica serovar Infantis] Overall Protective Antigen Prediction = **0.3964** (Probable **NON-ANTIGEN**).

>EHO9886196.1 trimeric intracellular cation channel family protein [Salmonella enterica subsp. enterica serovar Infantis] Overall Protective Antigen Prediction = **0.4391** (Probable **ANTIGEN**).

>EHO9886197.1 HARLDQ motif MBL-fold protein [Salmonella enterica subsp. enterica serovar Infantis] Overall Protective Antigen Prediction = **0.3265** (Probable **NON-ANTIGEN**).

>EHO9886198.1 LysR family transcriptional regulator [Salmonella enterica subsp. enterica serovar Infantis] Overall Protective Antigen Prediction = **0.4448** (Probable **ANTIGEN**).

>EHO9886199.1 YicC family protein [Salmonella enterica subsp. enterica serovar Infantis] Overall Protective Antigen Prediction = **0.5609** (Probable **ANTIGEN**).

>EHO9886200.1 ribonuclease PH [Salmonella enterica subsp. enterica serovar Infantis] Overall Protective Antigen Prediction = **0.5559** (Probable **ANTIGEN**).

>EHO9886201.1 orotate phosphoribosyltransferase [Salmonella enterica subsp. enterica serovar Infantis] Overall Protective Antigen Prediction = **0.2727** (Probable **NON-ANTIGEN**).

>EHO9886202.1 nucleoid occlusion factor SlmA [Salmonella enterica subsp. enterica serovar Infantis] Overall Protective Antigen Prediction = **0.5588** (Probable **ANTIGEN**).

>EHO9886203.1 dUTP diphosphatase [Salmonella enterica subsp. enterica serovar Infantis] Overall Protective Antigen Prediction = **0.7433** (Probable **ANTIGEN**).

>EHO9886204.1 bifunctional phosphopantothenoylcysteine decarboxylase/phosphopantothenate--cysteine ligase CoaBC [Salmonella enterica subsp. enterica serovar Infantis] Overall Protective

Antigen Prediction = **0.4680** (Probable **ANTIGEN**).

>EHO9886205.1 DNA repair protein RadC [Salmonella enterica subsp. enterica serovar Infantis] Overall Protective Antigen Prediction = **0.2174** (Probable **NON-ANTIGEN**).

>EHO9886206.1 50S ribosomal protein L28 [Salmonella enterica subsp. enterica serovar Infantis] Overall Protective Antigen Prediction = **0.5297** (Probable **ANTIGEN**).

>EHO9886207.1 50S ribosomal protein L33 [Salmonella enterica subsp. enterica serovar Infantis] Overall Protective Antigen Prediction = **0.7759** (Probable **ANTIGEN**).

>EHO9886208.1 bifunctional DNA-formamidopyrimidine glycosylase/DNA-(apurinic or apyrimidinic site) lyase [Salmonella enterica subsp. enterica serovar Infantis] Overall Protective Antigen Prediction = **0.4874** (Probable **ANTIGEN**).

>EHO9886209.1 pantetheine-phosphate adenylyltransferase [Salmonella enterica subsp. enterica serovar Infantis] Overall Protective Antigen Prediction = **0.1236** (Probable **NON-ANTIGEN**).

>EHO9886210.1 lipid IV(A) 3-deoxy-D-manno-octulosonic acid transferase [Salmonella enterica subsp. enterica serovar Infantis] Overall Protective Antigen Prediction = **0.3948** (Probable **NON-ANTIGEN**).

>EHO9886211.1 lipopolysaccharide core heptosyltransferase RfaQ [Salmonella enterica subsp. enterica serovar Infantis] Overall Protective Antigen Prediction = **0.2383** (Probable **NON-ANTIGEN**).

>EHO9886212.1 glycosyltransferase family 4 protein [Salmonella enterica subsp. enterica serovar Infantis] Overall Protective Antigen Prediction = **0.3204** (Probable **NON-ANTIGEN**).

>EHO9886213.1 lipopolysaccharide core heptose(I) kinase RfaP [Salmonella enterica subsp. enterica serovar Infantis] Overall Protective Antigen Prediction = **0.4641** (Probable **ANTIGEN**).

>EHO9886214.1 lipopolysaccharide 1,6-galactosyltransferase [Salmonella enterica subsp. enterica serovar Infantis] Overall Protective Antigen Prediction = **0.3198** (Probable **NON-ANTIGEN**).

>EHO9886215.1 lipopolysaccharide 3-alpha-galactosyltransferase [Salmonella enterica subsp. enterica serovar Infantis] Overall Protective Antigen Prediction = **0.3043** (Probable **NON-ANTIGEN**).

>EHO9886216.1 lipopolysaccharide 1,2-glucosyltransferase RfaJ [Salmonella enterica subsp. enterica serovar Infantis] Overall Protective Antigen Prediction = **0.3618** (Probable **NON-ANTIGEN**).

>EHO9886217.1 lipopolysaccharide core heptose(II) kinase RfaY [Salmonella enterica subsp. enterica serovar Infantis] Overall Protective Antigen Prediction = **0.2209** (Probable **NON-ANTIGEN**).

>EHO9886218.1 3-deoxy-D-manno-oct-2-ulosonate III transferase WaaZ [Salmonella enterica subsp. enterica serovar Infantis] Overall Protective Antigen Prediction = **0.3077** (Probable **NON-ANTIGEN**).

>EHO9886219.1 lipopolysaccharide N-acetylglucosaminyltransferase [Salmonella enterica subsp. enterica serovar Infantis] Overall Protective Antigen Prediction = **0.2298** (Probable **NON-ANTIGEN**).

>EHO9886220.1 O-antigen ligase RfaL [Salmonella enterica subsp. enterica serovar Infantis] Overall

Protective Antigen Prediction = **0.4159** (Probable **ANTIGEN**).

>EHO9886221.1 lipopolysaccharide heptosyltransferase RfaC [Salmonella enterica subsp. enterica serovar Infantis] Overall Protective Antigen Prediction = **0.3105** (Probable **NON-ANTIGEN**).

>EHO9886222.1 ADP-heptose--LPS heptosyltransferase RfaF [Salmonella enterica subsp. enterica serovar Infantis] Overall Protective Antigen Prediction = **0.3067** (Probable **NON-ANTIGEN**).

>EHO9886223.1 ADP-glyceromanno-heptose 6-epimerase [Salmonella enterica subsp. enterica serovar Infantis] Overall Protective Antigen Prediction = **0.3652** (Probable **NON-ANTIGEN**).

>EHO9886224.1 glycine C-acetyltransferase [Salmonella enterica subsp. enterica serovar Infantis] Overall Protective Antigen Prediction = **0.3469** (Probable **NON-ANTIGEN**).

>EHO9886225.1 L-threonine 3-dehydrogenase [Salmonella enterica subsp. enterica serovar Infantis] Overall Protective Antigen Prediction = **0.5226** (Probable **ANTIGEN**).

>EHO9886226.1 glycosyltransferase [Salmonella enterica subsp. enterica serovar Infantis] Overall Protective Antigen Prediction = **0.3709** (Probable **NON-ANTIGEN**).

>EHO9886227.1 divergent polysaccharide deacetylase family protein [Salmonella enterica subsp. enterica serovar Infantis] Overall Protective Antigen Prediction = **0.3855** (Probable **NON-ANTIGEN**).

>EHO9886228.1 murein hydrolase activator EnvC [Salmonella enterica subsp. enterica serovar Infantis] Overall Protective Antigen Prediction = **0.7337** (Probable **ANTIGEN**).

>EHO9886229.1 2,3-bisphosphoglycerate-independent phosphoglycerate mutase [Salmonella enterica subsp. enterica serovar Infantis] Overall Protective Antigen Prediction = **0.4957** (Probable **ANTIGEN**).

>EHO9886230.1 rhodanese-like domain-containing protein [Salmonella enterica subsp. enterica serovar Infantis] Overall Protective Antigen Prediction = **0.3722** (Probable **NON-ANTIGEN**).

>EHO9886231.1 glutaredoxin 3 [Salmonella enterica subsp. enterica serovar Infantis] Overall Protective Antigen Prediction = **0.6119** (Probable **ANTIGEN**).

>EHO9886232.1 protein-export chaperone SecB [Salmonella enterica subsp. enterica serovar Infantis] Overall Protective Antigen Prediction = **0.5524** (Probable **ANTIGEN**).

>EHO9886233.1 NAD(P)H-dependent glycerol-3-phosphate dehydrogenase [Salmonella enterica subsp. enterica serovar Infantis] Overall Protective Antigen Prediction = **0.4108** (Probable **ANTIGEN**).

>EHO9886234.1 serine O-acetyltransferase [Salmonella enterica subsp. enterica serovar Infantis] Overall Protective Antigen Prediction = **0.5052** (Probable **ANTIGEN**).

>EHO9886235.1 MFS transporter [Salmonella enterica subsp. enterica serovar Infantis] Overall Protective Antigen Prediction = **0.4199** (Probable **ANTIGEN**).

>EHO9886236.1 L-talarate/galactarate dehydratase [Salmonella enterica subsp. enterica serovar Infantis] Overall Protective Antigen Prediction = **0.5463** (Probable **ANTIGEN**).

>EHO9886237.1 LacI family DNA-binding transcriptional regulator [Salmonella enterica subsp.

enterica serovar Infantis] Overall Protective Antigen Prediction = **0.3947** (Probable **NON-ANTIGEN**).

>EHO9886238.1 tRNA (uridine(34)/cytosine(34)/5-carboxymethylaminomethyluridine(34)-2'-O)-methyltransferase TrmL [Salmonella enterica subsp. enterica serovar Infantis] Overall Protective Antigen Prediction = **0.3663** (Probable **NON-ANTIGEN**).

>EHO9886239.1 FMN-dependent L-lactate dehydrogenase LldD [Salmonella enterica subsp. enterica serovar Infantis] Overall Protective Antigen Prediction = **0.4371** (Probable **ANTIGEN**).

>EHO9886240.1 transcriptional regulator LldR [Salmonella enterica subsp. enterica serovar Infantis] Overall Protective Antigen Prediction = **0.5021** (Probable **ANTIGEN**).

>EHO9886241.1 L-lactate permease [Salmonella enterica subsp. enterica serovar Infantis] Overall Protective Antigen Prediction = **0.6244** (Probable **ANTIGEN**).

>EHO9886242.1 trimeric autotransporter adhesin SadA [Salmonella enterica subsp. enterica serovar Infantis] Overall Protective Antigen Prediction = **0.8036** (Probable **ANTIGEN**).

>EHO9886243.1 DUF3251 domain-containing protein [Salmonella enterica subsp. enterica serovar Infantis] Overall Protective Antigen Prediction = **0.4750** (Probable **ANTIGEN**).

>EHO9886244.1 YibL family ribosome-associated protein [Salmonella enterica subsp. enterica serovar Infantis] Overall Protective Antigen Prediction = **0.4130** (Probable **ANTIGEN**).

>EHO9886245.1 hypothetical protein KND05_001560 [Salmonella enterica subsp. enterica serovar Infantis] Overall Protective Antigen Prediction = **0.2543** (Probable **NON-ANTIGEN**).

>EHO9886246.1 mannitol operon repressor MtlR [Salmonella enterica subsp. enterica serovar Infantis] Overall Protective Antigen Prediction = **0.4026** (Probable **ANTIGEN**).

>EHO9886247.1 mannitol-1-phosphate 5-dehydrogenase [Salmonella enterica subsp. enterica serovar Infantis] Overall Protective Antigen Prediction = **0.2509** (Probable **NON-ANTIGEN**).

>EHO9886248.1 PTS mannitol transporter subunit IICBA [Salmonella enterica subsp. enterica serovar Infantis] Overall Protective Antigen Prediction = **0.3998** (Probable **NON-ANTIGEN**).

>EHO9886249.1 DUF3302 domain-containing protein [Salmonella enterica subsp. enterica serovar Infantis] Overall Protective Antigen Prediction = **0.9237** (Probable **ANTIGEN**).

>EHO9886250.1 HlyD family secretion protein [Salmonella enterica subsp. enterica serovar Infantis] Overall Protective Antigen Prediction = **0.4851** (Probable **ANTIGEN**).

>EHO9886251.1 glutathione S-transferase [Salmonella enterica subsp. enterica serovar Infantis] Overall Protective Antigen Prediction = **0.2556** (Probable **NON-ANTIGEN**).

>EHO9886252.1 L-seryl-tRNA(Sec) selenium transferase [Salmonella enterica subsp. enterica serovar Infantis] Overall Protective Antigen Prediction = **0.4477** (Probable **ANTIGEN**).

>EHO9886253.1 selenocysteine-specific translation elongation factor [Salmonella enterica subsp. enterica serovar Infantis] Overall Protective Antigen Prediction = **0.3828** (Probable **NON-ANTIGEN**).

>EHO9886254.1 ROK family protein [Salmonella enterica subsp. enterica serovar Infantis] Overall

Protective Antigen Prediction = **0.3466** (Probable **NON-ANTIGEN**).

>EHO9886255.1 aldehyde dehydrogenase AldB [Salmonella enterica subsp. enterica serovar Infantis] Overall Protective Antigen Prediction = **0.4785** (Probable **ANTIGEN**).

>EHO9886256.1 glycoside hydrolase family 127 protein [Salmonella enterica subsp. enterica serovar Infantis] Overall Protective Antigen Prediction = **0.4653** (Probable **ANTIGEN**).

>EHO9886257.1 helix-turn-helix domain-containing protein [Salmonella enterica subsp. enterica serovar Infantis] Overall Protective Antigen Prediction = **0.4774** (Probable **ANTIGEN**).

>EHO9886258.1 L-ribulose-5-phosphate 4-epimerase [Salmonella enterica subsp. enterica serovar Infantis] Overall Protective Antigen Prediction = **0.4252** (Probable **ANTIGEN**).

>EHO9886259.1 L-ribulose-5-phosphate 3-epimerase [Salmonella enterica subsp. enterica serovar Infantis] Overall Protective Antigen Prediction = **0.3683** (Probable **NON-ANTIGEN**).

>EHO9886260.1 3-keto-L-gulonate-6-phosphate decarboxylase UlaD [Salmonella enterica subsp. enterica serovar Infantis] Overall Protective Antigen Prediction = **0.4649** (Probable **ANTIGEN**).

>EHO9886261.1 carbohydrate kinase [Salmonella enterica subsp. enterica serovar Infantis] Overall Protective Antigen Prediction = **0.3656** (Probable **NON-ANTIGEN**).

>EHO9886262.1 TRAP transporter substrate-binding protein [Salmonella enterica subsp. enterica serovar Infantis] Overall Protective Antigen Prediction = **0.5919** (Probable **ANTIGEN**).

>EHO9886263.1 2,3-diketo-L-gulonate transporter large permease YiaN [Salmonella enterica subsp. enterica serovar Infantis] Overall Protective Antigen Prediction = **0.5758** (Probable **ANTIGEN**).

>EHO9886264.1 2,3-diketo-L-gulonate TRAP transporter small permease YiaM [Salmonella enterica subsp. enterica serovar Infantis] Overall Protective Antigen Prediction = **0.3114** (Probable **NON-ANTIGEN**).

>EHO9886265.1 DUF4862 family protein [Salmonella enterica subsp. enterica serovar Infantis] Overall Protective Antigen Prediction = **0.4967** (Probable **ANTIGEN**).

>EHO9886266.1 YhcH/YjgK/YiaL family protein [Salmonella enterica subsp. enterica serovar Infantis] Overall Protective Antigen Prediction = **0.3868** (Probable **NON-ANTIGEN**).

>EHO9886267.1 3-dehydro-L-gulonate 2-dehydrogenase [Salmonella enterica subsp. enterica serovar Infantis] Overall Protective Antigen Prediction = **0.3432** (Probable **NON-ANTIGEN**).

>EHO9886268.1 IclR family transcriptional regulator YiaJ [Salmonella enterica subsp. enterica serovar Infantis] Overall Protective Antigen Prediction = **0.3745** (Probable **NON-ANTIGEN**).

>EHO9886269.1 4Fe-4S binding protein [Salmonella enterica subsp. enterica serovar Infantis] Overall Protective Antigen Prediction = **0.3866** (Probable **NON-ANTIGEN**).

>EHO9886270.1 valine--pyruvate transaminase [Salmonella enterica subsp. enterica serovar Infantis] Overall Protective Antigen Prediction = **0.3641** (Probable **NON-ANTIGEN**).

>EHO9886271.1 alpha-amylase [Salmonella enterica subsp. enterica serovar Infantis] Overall Protective Antigen Prediction = **0.5028** (Probable **ANTIGEN**).

>EHO9886272.1 protein bax [Salmonella enterica subsp. enterica serovar Infantis] Overall Protective Antigen Prediction = **0.5764** (Probable **ANTIGEN**).

>EHO9886273.1 XylR family transcriptional regulator [Salmonella enterica subsp. enterica serovar Infantis] Overall Protective Antigen Prediction = **0.2367** (Probable **NON-ANTIGEN**).

>EHO9886274.1 xylose isomerase [Salmonella enterica subsp. enterica serovar Infantis] Overall Protective Antigen Prediction = **0.5027** (Probable **ANTIGEN**).

>EHO9886275.1 xylulokinase [Salmonella enterica subsp. enterica serovar Infantis] Overall Protective Antigen Prediction = **0.3534** (Probable **NON-ANTIGEN**).

>EHO9886276.1 YiaA/YiaB family protein [Salmonella enterica subsp. enterica serovar Infantis] Overall Protective Antigen Prediction = **0.2912** (Probable **NON-ANTIGEN**).

>EHO9886277.1 acyltransferase [Salmonella enterica subsp. enterica serovar Infantis] Overall Protective Antigen Prediction = **0.5923** (Probable **ANTIGEN**).

>EHO9886278.1 lipoprotein [Salmonella enterica subsp. enterica serovar Infantis] Overall Protective Antigen Prediction = **0.7476** (Probable **ANTIGEN**).

>EHO9886279.1 glycine--tRNA ligase subunit alpha [Salmonella enterica subsp. enterica serovar Infantis] Overall Protective Antigen Prediction = **0.4740** (Probable **ANTIGEN**).

>EHO9886280.1 glycine--tRNA ligase subunit beta [Salmonella enterica subsp. enterica serovar Infantis] Overall Protective Antigen Prediction = **0.3878** (Probable **NON-ANTIGEN**).

>EHO9886281.1 IS3 family transposase [Salmonella enterica subsp. enterica serovar Infantis] Overall Protective Antigen Prediction = **0.2970** (Probable **NON-ANTIGEN**).

>EHO9886282.1 GntR family transcriptional regulator [Salmonella enterica subsp. enterica serovar Infantis] Overall Protective Antigen Prediction = **0.3979** (Probable **NON-ANTIGEN**).

>EHO9886283.1 MFS transporter [Salmonella enterica subsp. enterica serovar Infantis] Overall Protective Antigen Prediction = **0.3988** (Probable **NON-ANTIGEN**).

>EHO9886284.1 cysteine hydrolase [Salmonella enterica subsp. enterica serovar Infantis] Overall Protective Antigen Prediction = **0.2156** (Probable **NON-ANTIGEN**).

>EHO9886285.1 DUF1778 domain-containing protein [Salmonella enterica subsp. enterica serovar Infantis] Overall Protective Antigen Prediction = **0.3995** (Probable **NON-ANTIGEN**).

>EHO9886286.1 GNAT family N-acetyltransferase [Salmonella enterica subsp. enterica serovar Infantis] Overall Protective Antigen Prediction = **0.4413** (Probable **ANTIGEN**).

>EHO9886287.1 copper-binding periplasmic metallochaperone CueP [Salmonella enterica subsp. enterica serovar Infantis] Overall Protective Antigen Prediction = **0.6356** (Probable **ANTIGEN**).

>EHO9886288.1 RNA chaperone/antiterminator CspA [Salmonella enterica subsp. enterica serovar Infantis] Overall Protective Antigen Prediction = **0.2514** (Probable **NON-ANTIGEN**).

>EHO9886289.1 HTH-type transcriptional regulator [Salmonella enterica subsp. enterica serovar Infantis] Overall Protective Antigen Prediction = **0.3450** (Probable **NON-ANTIGEN**).

>EHO9886290.1 DUF3053 domain-containing protein [Salmonella enterica subsp. enterica serovar Infantis] Overall Protective Antigen Prediction = **0.3423** (Probable **NON-ANTIGEN**).

>EHO9886291.1 glyoxylate/hydroxypyruvate reductase GhrB [Salmonella enterica subsp. enterica serovar Infantis] Overall Protective Antigen Prediction = **0.4055** (Probable **ANTIGEN**).

>EHO9886292.1 OmpA family lipoprotein [Salmonella enterica subsp. enterica serovar Infantis] Overall Protective Antigen Prediction = **0.8816** (Probable **ANTIGEN**).

>EHO9886293.1 molybdopterin guanine dinucleotide-containing S/N-oxide reductase [Salmonella enterica subsp. enterica serovar Infantis] Overall Protective Antigen Prediction = **0.3954** (Probable **NON-ANTIGEN**).

>EHO9886294.1 N-acetyltransferase [Salmonella enterica subsp. enterica serovar Infantis] Overall Protective Antigen Prediction = **0.3393** (Probable **NON-ANTIGEN**).

>EHO9886295.1 DNA-3-methyladenine glycosylase I [Salmonella enterica subsp. enterica serovar Infantis] Overall Protective Antigen Prediction = **0.2471** (Probable **NON-ANTIGEN**).

>EHO9886296.1 autotransporter outer membrane beta-barrel domain-containing protein [Salmonella enterica subsp. enterica serovar Infantis] Overall Protective Antigen Prediction = **0.6288** (Probable **ANTIGEN**).

>EHO9886297.1 long polar fimbria major subunit LpfA [Salmonella enterica subsp. enterica serovar Infantis] Overall Protective Antigen Prediction = **0.7419** (Probable **ANTIGEN**).

>EHO9886298.1 molecular chaperone LpfB [Salmonella enterica subsp. enterica serovar Infantis] Overall Protective Antigen Prediction = **0.5983** (Probable **ANTIGEN**).

>EHO9886299.1 outer membrane usher protein LpfC [Salmonella enterica subsp. enterica serovar Infantis] Overall Protective Antigen Prediction = **0.6073** (Probable **ANTIGEN**).

>EHO9886300.1 long polar fimbrial protein LpfD [Salmonella enterica subsp. enterica serovar Infantis] Overall Protective Antigen Prediction = **0.4801** (Probable **ANTIGEN**).

>EHO9886301.1 long polar fimbrial protein LpfE [Salmonella enterica subsp. enterica serovar Infantis] Overall Protective Antigen Prediction = **0.8330** (Probable **ANTIGEN**).

>EHO9886302.1 kdo(2)-lipid A phosphoethanolamine 7''-transferase [Salmonella enterica subsp. enterica serovar Infantis] Overall Protective Antigen Prediction = **0.4249** (Probable **ANTIGEN**).

>EHO9886303.1 substrate-binding domain-containing protein [Salmonella enterica subsp. enterica serovar Infantis] Overall Protective Antigen Prediction = **0.5031** (Probable **ANTIGEN**).

>EHO9886304.1 aryl-sulfate sulfotransferase [Salmonella enterica subsp. enterica serovar Infantis] Overall Protective Antigen Prediction = **0.3449** (Probable **NON-ANTIGEN**).

>EHO9886305.1 uracil-xanthine permease [Salmonella enterica subsp. enterica serovar Infantis] Overall Protective Antigen Prediction = **0.4860** (Probable **ANTIGEN**).

>EHO9886306.1 ABC transporter substrate-binding protein [Salmonella enterica subsp. enterica serovar Infantis] Overall Protective Antigen Prediction = **0.4518** (Probable **ANTIGEN**).

>EHO9886307.1 dipeptide ABC transporter permease DppB [Salmonella enterica subsp. enterica

serovar Infantis] Overall Protective Antigen Prediction = **0.3542** (Probable **NON-ANTIGEN**).

>EHO9886308.1 dipeptide ABC transporter permease DppC [Salmonella enterica subsp. enterica serovar Infantis] Overall Protective Antigen Prediction = **0.4227** (Probable **ANTIGEN**).

>EHO9886309.1 dipeptide ABC transporter ATP-binding protein [Salmonella enterica subsp. enterica serovar Infantis] Overall Protective Antigen Prediction = **0.4608** (Probable **ANTIGEN**).

>EHO9886310.1 dipeptide ABC transporter ATP binding subunit DppF [Salmonella enterica subsp. enterica serovar Infantis] Overall Protective Antigen Prediction = **0.4629** (Probable **ANTIGEN**).

>EHO9886311.1 transporter [Salmonella enterica subsp. enterica serovar Infantis] Overall Protective Antigen Prediction = **0.5450** (Probable **ANTIGEN**).

>EHO9886312.1 type I toxin-antitoxin system toxin Ldr family protein [Salmonella enterica subsp. enterica serovar Infantis] Overall Protective Antigen Prediction = **-0.1518** (Probable **NON-ANTIGEN**).

>EHO9886313.1 DNA-binding transcriptional regulator H-NS [Salmonella enterica subsp. enterica serovar Infantis] Overall Protective Antigen Prediction = **0.5456** (Probable **ANTIGEN**).

>EHO9886314.1 UTP--glucose-1-phosphate uridylyltransferase GalU [Salmonella enterica subsp. enterica serovar Infantis] Overall Protective Antigen Prediction = **0.4468** (Probable **ANTIGEN**).

>EHO9886315.1 two-component system response regulator RssB [Salmonella enterica subsp. enterica serovar Infantis] Overall Protective Antigen Prediction = **0.3550** (Probable **NON-ANTIGEN**).

>EHO9886316.1 patatin-like phospholipase RssA [Salmonella enterica subsp. enterica serovar Infantis] Overall Protective Antigen Prediction = **0.3488** (Probable **NON-ANTIGEN**).

>EHO9886317.1 YchJ family protein [Salmonella enterica subsp. enterica serovar Infantis] Overall Protective Antigen Prediction = **0.3420** (Probable **NON-ANTIGEN**).

>EHO9886318.1 formyltetrahydrofolate deformylase [Salmonella enterica subsp. enterica serovar Infantis] Overall Protective Antigen Prediction = **0.2926** (Probable **NON-ANTIGEN**).

>EHO9886319.1 sel1 repeat family protein [Salmonella enterica subsp. enterica serovar Infantis] Overall Protective Antigen Prediction = **0.4189** (Probable **ANTIGEN**).

>EHO9886320.1 respiratory nitrate reductase subunit gamma [Salmonella enterica subsp. enterica serovar Infantis] Overall Protective Antigen Prediction = **0.6135** (Probable **ANTIGEN**).

>EHO9886321.1 nitrate reductase molybdenum cofactor assembly chaperone [Salmonella enterica subsp. enterica serovar Infantis] Overall Protective Antigen Prediction = **0.4405** (Probable **ANTIGEN**).

>EHO9886322.1 nitrate reductase subunit beta [Salmonella enterica subsp. enterica serovar Infantis] Overall Protective Antigen Prediction = **0.5324** (Probable **ANTIGEN**).

>EHO9886323.1 nitrate reductase subunit alpha [Salmonella enterica subsp. enterica serovar Infantis] Overall Protective Antigen Prediction = **0.4908** (Probable **ANTIGEN**).

>EHO9886324.1 nitrate transporter NarK [Salmonella enterica subsp. enterica serovar Infantis] Overall Protective Antigen Prediction = **0.5828** (Probable **ANTIGEN**).

>EHO9886325.1 nitrate/nitrite two-component system sensor histidine kinase NarX [Salmonella enterica subsp. enterica serovar Infantis] Overall Protective Antigen Prediction = **0.5877** (Probable **ANTIGEN**).

>EHO9886326.1 two-component system response regulator NarL [Salmonella enterica subsp. enterica serovar Infantis] Overall Protective Antigen Prediction = **0.4123** (Probable **ANTIGEN**).

>EHO9886327.1 YchO/YchP family invasin [Salmonella enterica subsp. enterica serovar Infantis] Overall Protective Antigen Prediction = **0.6583** (Probable **ANTIGEN**).

>EHO9886328.1 DsrE/DsrF/TusD sulfur relay family protein [Salmonella enterica subsp. enterica serovar Infantis] Overall Protective Antigen Prediction = **0.6084** (Probable **ANTIGEN**).

>EHO9886329.1 putative cation transport regulator ChaB [Salmonella enterica subsp. enterica serovar Infantis] Overall Protective Antigen Prediction = **0.6714** (Probable **ANTIGEN**).

>EHO9886330.1 sodium-potassium/proton antiporter ChaA [Salmonella enterica subsp. enterica serovar Infantis] Overall Protective Antigen Prediction = **0.4690** (Probable **ANTIGEN**).

>EHO9886331.1 3-deoxy-8-phosphooctulonate synthase [Salmonella enterica subsp. enterica serovar Infantis] Overall Protective Antigen Prediction = **0.3558** (Probable **NON-ANTIGEN**).

>EHO9886332.1 tetratricopeptide repeat-containing protein [Salmonella enterica subsp. enterica serovar Infantis] Overall Protective Antigen Prediction = **0.2921** (Probable **NON-ANTIGEN**).

>EHO9886333.1 SirB family protein [Salmonella enterica subsp. enterica serovar Infantis] Overall Protective Antigen Prediction = **0.5167** (Probable **ANTIGEN**).

>EHO9886334.1 peptide chain release factor N(5)-glutamine methyltransferase [Salmonella enterica subsp. enterica serovar Infantis] Overall Protective Antigen Prediction = **0.4206** (Probable **ANTIGEN**).

>EHO9886335.1 peptide chain release factor 1 [Salmonella enterica subsp. enterica serovar Infantis] Overall Protective Antigen Prediction = **0.6876** (Probable **ANTIGEN**).

>EHO9886336.1 glutamyl-tRNA reductase [Salmonella enterica subsp. enterica serovar Infantis] Overall Protective Antigen Prediction = **0.4578** (Probable **ANTIGEN**).

>EHO9886337.1 lipoprotein localization protein LolB [Salmonella enterica subsp. enterica serovar Infantis] Overall Protective Antigen Prediction = **0.7997** (Probable **ANTIGEN**).

>EHO9886338.1 4-(cytidine 5'-diphospho)-2-C-methyl-D-erythritol kinase [Salmonella enterica subsp. enterica serovar Infantis] Overall Protective Antigen Prediction = **0.5590** (Probable **ANTIGEN**).

>EHO9886339.1 ribose-phosphate diphosphokinase [Salmonella enterica subsp. enterica serovar Infantis] Overall Protective Antigen Prediction = **0.4305** (Probable **ANTIGEN**).

>EHO9886340.1 C4-dicarboxylic acid transporter DauA [Salmonella enterica subsp. enterica serovar Infantis] Overall Protective Antigen Prediction = **0.5206** (Probable **ANTIGEN**).

>EHO9886341.1 stress-induced protein YchH [Salmonella enterica subsp. enterica serovar Infantis] Overall Protective Antigen Prediction = **0.6325** (Probable **ANTIGEN**).

>EHO9886342.1 aminoacyl-tRNA hydrolase [Salmonella enterica subsp. enterica serovar Infantis] Overall Protective Antigen Prediction = **0.4260** (Probable **ANTIGEN**).

>EHO9886343.1 redox-regulated ATPase YchF [Salmonella enterica subsp. enterica serovar Infantis] Overall Protective Antigen Prediction = **0.3951** (Probable **NON-ANTIGEN**).

>EHO9886344.1 hypothetical protein KND05_001670 [Salmonella enterica subsp. enterica serovar Infantis] Overall Protective Antigen Prediction = **0.3641** (Probable **NON-ANTIGEN**).

>EHO9886345.1 DUF4427 domain-containing protein [Salmonella enterica subsp. enterica serovar Infantis] Overall Protective Antigen Prediction = **0.2933** (Probable **NON-ANTIGEN**).

>EHO9886346.1 hydrogenase 1 small subunit [Salmonella enterica subsp. enterica serovar Infantis] Overall Protective Antigen Prediction = **0.3528** (Probable **NON-ANTIGEN**).

>EHO9886347.1 Ni/Fe-hydrogenase large subunit [Salmonella enterica subsp. enterica serovar Infantis] Overall Protective Antigen Prediction = **0.2554** (Probable **NON-ANTIGEN**).

>EHO9886348.1 Ni/Fe-hydrogenase b-type cytochrome subunit [Salmonella enterica subsp. enterica serovar Infantis] Overall Protective Antigen Prediction = **0.5533** (Probable **ANTIGEN**).

>EHO9886349.1 hydrogenase 1 maturation protease [Salmonella enterica subsp. enterica serovar Infantis] Overall Protective Antigen Prediction = **0.4600** (Probable **ANTIGEN**).

>EHO9886350.1 hydrogenase-1 operon protein HyaE [Salmonella enterica subsp. enterica serovar Infantis] Overall Protective Antigen Prediction = **0.3656** (Probable **NON-ANTIGEN**).

>EHO9886351.1 hydrogenase expression/formation protein [Salmonella enterica subsp. enterica serovar Infantis] Overall Protective Antigen Prediction = **0.5048** (Probable **ANTIGEN**).

>EHO9886352.1 cytochrome bd-II oxidase subunit 1 [Salmonella enterica subsp. enterica serovar Infantis] Overall Protective Antigen Prediction = **0.5153** (Probable **ANTIGEN**).

>EHO9886353.1 cytochrome d ubiquinol oxidase subunit II [Salmonella enterica subsp. enterica serovar Infantis] Overall Protective Antigen Prediction = **0.6178** (Probable **ANTIGEN**).

>EHO9886354.1 cytochrome bd-II oxidase subunit CbdX [Salmonella enterica subsp. enterica serovar Infantis] Overall Protective Antigen Prediction = **0.7808** (Probable **ANTIGEN**).

>EHO9886355.1 Glu/Leu/Phe/Val dehydrogenase [Salmonella enterica subsp. enterica serovar Infantis] Overall Protective Antigen Prediction = **0.5157** (Probable **ANTIGEN**).

>EHO9886356.1 alpha,alpha-trehalase [Salmonella enterica subsp. enterica serovar Infantis] Overall Protective Antigen Prediction = **0.4757** (Probable **ANTIGEN**).

>EHO9886357.1 GlsB/YeaQ/YmgE family stress response membrane protein [Salmonella enterica subsp. enterica serovar Infantis] Overall Protective Antigen Prediction = **0.3087** (Probable **NON-ANTIGEN**).

>EHO9886358.1 flagellar brake protein YcgR [Salmonella enterica subsp. enterica serovar Infantis] Overall Protective Antigen Prediction = **0.4667** (Probable **ANTIGEN**).

>EHO9886359.1 membrane-bound lytic murein transglycosylase EmtA [Salmonella enterica subsp.

enterica serovar Infantis] Overall Protective Antigen Prediction = **0.6014** (Probable **ANTIGEN**).

>EHO9886360.1 muramoyltetrapeptide carboxypeptidase [Salmonella enterica subsp. enterica serovar Infantis] Overall Protective Antigen Prediction = **0.3948** (Probable **NON-ANTIGEN**).

>EHO9886361.1 potassium/proton antiporter [Salmonella enterica subsp. enterica serovar Infantis] Overall Protective Antigen Prediction = **0.4963** (Probable **ANTIGEN**).

>EHO9886362.1 catabolic alanine racemase DadX [Salmonella enterica subsp. enterica serovar Infantis] Overall Protective Antigen Prediction = **0.5311** (Probable **ANTIGEN**).

>EHO9886363.1 D-amino acid dehydrogenase [Salmonella enterica subsp. enterica serovar Infantis] Overall Protective Antigen Prediction = **0.4891** (Probable **ANTIGEN**).

>EHO9886364.1 SpoVR family protein [Salmonella enterica subsp. enterica serovar Infantis] Overall Protective Antigen Prediction = **0.3287** (Probable **NON-ANTIGEN**).

>EHO9886365.1 fatty acid metabolism transcriptional regulator FadR [Salmonella enterica subsp. enterica serovar Infantis] Overall Protective Antigen Prediction = **0.1724** (Probable **NON-ANTIGEN**).

>EHO9886366.1 Na(+)/H(+) antiporter NhaB [Salmonella enterica subsp. enterica serovar Infantis] Overall Protective Antigen Prediction = **0.4781** (Probable **ANTIGEN**).

>EHO9886367.1 disulfide bond formation protein DsbB [Salmonella enterica subsp. enterica serovar Infantis] Overall Protective Antigen Prediction = **0.4857** (Probable **ANTIGEN**).

>EHO9886368.1 DUF1971 domain-containing protein [Salmonella enterica subsp. enterica serovar Infantis] Overall Protective Antigen Prediction = **0.4588** (Probable **ANTIGEN**).

>EHO9886369.1 addiction module toxin, GnsA/GnsB family [Salmonella enterica subsp. enterica serovar Infantis] Overall Protective Antigen Prediction = **0.7766** (Probable **ANTIGEN**).

>EHO9886370.1 hypothetical protein KND05_001696 [Salmonella enterica subsp. enterica serovar Infantis] Overall Protective Antigen Prediction = **0.2008** (Probable **NON-ANTIGEN**).

>EHO9886371.1 YcgN family cysteine cluster protein [Salmonella enterica subsp. enterica serovar Infantis] Overall Protective Antigen Prediction = **0.6433** (Probable **ANTIGEN**).

>EHO9886372.1 fumarylacetoacetate hydrolase family protein [Salmonella enterica subsp. enterica serovar Infantis] Overall Protective Antigen Prediction = **0.4930** (Probable **ANTIGEN**).

>EHO9886373.1 YcgL domain-containing protein [Salmonella enterica subsp. enterica serovar Infantis] Overall Protective Antigen Prediction = **0.2653** (Probable **NON-ANTIGEN**).

>EHO9886374.1 septum site-determining protein MinC [Salmonella enterica subsp. enterica serovar Infantis] Overall Protective Antigen Prediction = **0.5481** (Probable **ANTIGEN**).

>EHO9886375.1 septum site-determining protein MinD [Salmonella enterica subsp. enterica serovar Infantis] Overall Protective Antigen Prediction = **0.4515** (Probable **ANTIGEN**).

>EHO9886376.1 cell division topological specificity factor MinE [Salmonella enterica subsp. enterica serovar Infantis] Overall Protective Antigen Prediction = **0.6239** (Probable **ANTIGEN**).

>EHO9886377.1 ribonuclease D [Salmonella enterica subsp. enterica serovar Infantis] Overall Protective Antigen Prediction = **0.3937** (Probable **NON-ANTIGEN**).

>EHO9886378.1 long-chain-fatty-acid--CoA ligase FadD [Salmonella enterica subsp. enterica serovar Infantis] Overall Protective Antigen Prediction = **0.4263** (Probable **ANTIGEN**).

>EHO9886379.1 Slp family lipoprotein [Salmonella enterica subsp. enterica serovar Infantis] Overall Protective Antigen Prediction = **0.3819** (Probable **NON-ANTIGEN**).

>EHO9886380.1 tRNA (adenosine(37)-N6)-threonylcarbamoyltransferase complex dimerization subunit type 1 TsaB [Salmonella enterica subsp. enterica serovar Infantis] Overall Protective Antigen Prediction = **0.4102** (Probable **ANTIGEN**).

>EHO9886381.1 ATP-dependent DNA helicase [Salmonella enterica subsp. enterica serovar Infantis] Overall Protective Antigen Prediction = **0.3104** (Probable **NON-ANTIGEN**).

>EHO9886382.1 RidA family protein [Salmonella enterica subsp. enterica serovar Infantis] Overall Protective Antigen Prediction = **0.4262** (Probable **ANTIGEN**).

>EHO9886383.1 YoaH family protein [Salmonella enterica subsp. enterica serovar Infantis] Overall Protective Antigen Prediction = **0.5311** (Probable **ANTIGEN**).

>EHO9886384.1 aminodeoxychorismate synthase component 1 [Salmonella enterica subsp. enterica serovar Infantis] Overall Protective Antigen Prediction = **0.4669** (Probable **ANTIGEN**).

>EHO9886385.1 CoA pyrophosphatase [Salmonella enterica subsp. enterica serovar Infantis] Overall Protective Antigen Prediction = **0.4917** (Probable **ANTIGEN**).

>EHO9886386.1 L-serine ammonia-lyase [Salmonella enterica subsp. enterica serovar Infantis] Overall Protective Antigen Prediction = **0.4787** (Probable **ANTIGEN**).

>EHO9886387.1 EAL domain-containing protein [Salmonella enterica subsp. enterica serovar Infantis] Overall Protective Antigen Prediction = **0.4618** (Probable **ANTIGEN**).

>EHO9886388.1 CNNM family cation transport protein YoaE [Salmonella enterica subsp. enterica serovar Infantis] Overall Protective Antigen Prediction = **0.3479** (Probable **NON-ANTIGEN**).

>EHO9886389.1 PTS mannose transporter subunit IIAB [Salmonella enterica subsp. enterica serovar Infantis] Overall Protective Antigen Prediction = **0.4624** (Probable **ANTIGEN**).

>EHO9886390.1 PTS mannose transporter subunit IIC [Salmonella enterica subsp. enterica serovar Infantis] Overall Protective Antigen Prediction = **0.5319** (Probable **ANTIGEN**).

>EHO9886391.1 PTS mannose transporter subunit IID [Salmonella enterica subsp. enterica serovar Infantis] Overall Protective Antigen Prediction = **0.5759** (Probable **ANTIGEN**).

>EHO9886392.1 DUF986 domain-containing protein [Salmonella enterica subsp. enterica serovar Infantis] Overall Protective Antigen Prediction = **0.2899** (Probable **NON-ANTIGEN**).

>EHO9886393.1 manganese efflux pump MntP [Salmonella enterica subsp. enterica serovar Infantis] Overall Protective Antigen Prediction = **0.4319** (Probable **ANTIGEN**).

>EHO9886394.1 23S rRNA (guanine(745)-N(1))-methyltransferase [Salmonella enterica subsp. enterica serovar Infantis] Overall Protective Antigen Prediction = **0.3619** (Probable **NON-ANTIGEN**

).

>EHO9886395.1 peptidoglycan glycosyltransferase FtsI [Salmonella enterica subsp. enterica serovar Infantis] Overall Protective Antigen Prediction = **0.4909** (Probable **ANTIGEN**).

>EHO9886396.1 transcription antiterminator/RNA stability regulator CspE [Salmonella enterica subsp. enterica serovar Infantis] Overall Protective Antigen Prediction = **0.4460** (Probable **ANTIGEN**).

>EHO9886397.1 YobF family protein [Salmonella enterica subsp. enterica serovar Infantis] Overall Protective Antigen Prediction = **0.8146** (Probable **ANTIGEN**).

>EHO9886398.1 YebO family protein [Salmonella enterica subsp. enterica serovar Infantis] Overall Protective Antigen Prediction = **0.4399** (Probable **ANTIGEN**).

>EHO9886399.1 PhoP/PhoQ regulator MgrB [Salmonella enterica subsp. enterica serovar Infantis] Overall Protective Antigen Prediction = **0.5450** (Probable **ANTIGEN**).

>EHO9886400.1 hypothetical protein KND05_001726 [Salmonella enterica subsp. enterica serovar Infantis] Overall Protective Antigen Prediction = **0.4579** (Probable **ANTIGEN**).

>EHO9886401.1 DNA-binding transcriptional regulator KdgR [Salmonella enterica subsp. enterica serovar Infantis] Overall Protective Antigen Prediction = **0.3792** (Probable **NON-ANTIGEN**).

>EHO9886402.1 MFS transporter [Salmonella enterica subsp. enterica serovar Infantis] Overall Protective Antigen Prediction = **0.5634** (Probable **ANTIGEN**).

>EHO9886403.1 protease HtpX [Salmonella enterica subsp. enterica serovar Infantis] Overall Protective Antigen Prediction = **0.4394** (Probable **ANTIGEN**).

>EHO9886404.1 carboxy terminal-processing peptidase [Salmonella enterica subsp. enterica serovar Infantis] Overall Protective Antigen Prediction = **0.4969** (Probable **ANTIGEN**).

>EHO9886405.1 RNA chaperone ProQ [Salmonella enterica subsp. enterica serovar Infantis] Overall Protective Antigen Prediction = **0.8073** (Probable **ANTIGEN**).

>EHO9886406.1 GAF domain-containing protein [Salmonella enterica subsp. enterica serovar Infantis] Overall Protective Antigen Prediction = **0.2955** (Probable **NON-ANTIGEN**).

>EHO9886407.1 membrane integrity lipid transport subunit YebS [Salmonella enterica subsp. enterica serovar Infantis] Overall Protective Antigen Prediction = **0.6052** (Probable **ANTIGEN**).

>EHO9886408.1 MCE family protein [Salmonella enterica subsp. enterica serovar Infantis] Overall Protective Antigen Prediction = **0.5848** (Probable **ANTIGEN**).

>EHO9886409.1 16S rRNA (cytosine(1407)-C(5))-methyltransferase RsmF [Salmonella enterica subsp. enterica serovar Infantis] Overall Protective Antigen Prediction = **0.3166** (Probable **NON-ANTIGEN**).

>EHO9886410.1 DUF1480 family protein [Salmonella enterica subsp. enterica serovar Infantis] Overall Protective Antigen Prediction = **0.7204** (Probable **ANTIGEN**).

>EHO9886411.1 YebW family protein [Salmonella enterica subsp. enterica serovar Infantis] Overall Protective Antigen Prediction = **0.5214** (Probable **ANTIGEN**).

>EHO9886412.1 protein-serine/threonine phosphatase [Salmonella enterica subsp. enterica serovar Infantis] Overall Protective Antigen Prediction = **0.3336** (Probable **NON-ANTIGEN**).

>EHO9886413.1 hypothetical protein KND05_001739 [Salmonella enterica subsp. enterica serovar Infantis] Overall Protective Antigen Prediction = **0.8257** (Probable **ANTIGEN**).

>EHO9886414.1 SPI-1 type III secretion system guanine nucleotide exchange factor SopE2 [Salmonella enterica subsp. enterica serovar Infantis] Overall Protective Antigen Prediction = **0.4834** (Probable **ANTIGEN**).

>EHO9886415.1 DUF1398 domain-containing protein [Salmonella enterica subsp. enterica serovar Infantis] Overall Protective Antigen Prediction = **0.4034** (Probable **ANTIGEN**).

>EHO9886416.1 hypothetical protein KND05_001742 [Salmonella enterica subsp. enterica serovar Infantis] Overall Protective Antigen Prediction = **0.3466** (Probable **NON-ANTIGEN**).

>EHO9886417.1 GNAT family N-acetyltransferase [Salmonella enterica subsp. enterica serovar Infantis] Overall Protective Antigen Prediction = **0.4113** (Probable **ANTIGEN**).

>EHO9886418.1 hypothetical protein KND05_001744 [Salmonella enterica subsp. enterica serovar Infantis] Overall Protective Antigen Prediction = **0.5591** (Probable **ANTIGEN**).

>EHO9886419.1 hypothetical protein KND05_001745 [Salmonella enterica subsp. enterica serovar Infantis] Overall Protective Antigen Prediction = **0.4731** (Probable **ANTIGEN**).

>EHO9886420.1 IS256 family transposase [Salmonella enterica subsp. enterica serovar Infantis] Overall Protective Antigen Prediction = **0.4201** (Probable **ANTIGEN**).

>EHO9886421.1 DMT family transporter [Salmonella enterica subsp. enterica serovar Infantis] Overall Protective Antigen Prediction = **0.5879** (Probable **ANTIGEN**).

>EHO9886422.1 hypothetical protein KND05_001749 [Salmonella enterica subsp. enterica serovar Infantis] Overall Protective Antigen Prediction = **0.8420** (Probable **ANTIGEN**).

>EHO9886423.1 disulfide bond formation protein B [Salmonella enterica subsp. enterica serovar Infantis] Overall Protective Antigen Prediction = **0.5148** (Probable **ANTIGEN**).

>EHO9886424.1 hypothetical protein KND05_001752 [Salmonella enterica subsp. enterica serovar Infantis] Overall Protective Antigen Prediction = **0.2962** (Probable **NON-ANTIGEN**).

>EHO9886425.1 arsenic transporter [Salmonella enterica subsp. enterica serovar Infantis] Overall Protective Antigen Prediction = **0.5318** (Probable **ANTIGEN**).

>EHO9886426.1 lytic enzyme [Salmonella enterica subsp. enterica serovar Infantis] Overall Protective Antigen Prediction = **0.3670** (Probable **NON-ANTIGEN**).

>EHO9886427.1 DUF2514 domain-containing protein [Salmonella enterica subsp. enterica serovar Infantis] Overall Protective Antigen Prediction = **0.6710** (Probable **ANTIGEN**).

>EHO9886428.1 YebY family protein [Salmonella enterica subsp. enterica serovar Infantis] Overall Protective Antigen Prediction = **0.4305** (Probable **ANTIGEN**).

>EHO9886429.1 copper homeostasis membrane protein CopD [Salmonella enterica subsp. enterica

serovar Infantis] Overall Protective Antigen Prediction = **0.3996** (Probable **NON-ANTIGEN**).

>EHO9886430.1 CopC domain-containing protein YobA [Salmonella enterica subsp. enterica serovar Infantis] Overall Protective Antigen Prediction = **0.7268** (Probable **ANTIGEN**).

>EHO9886431.1 DNA polymerase III subunit theta [Salmonella enterica subsp. enterica serovar Infantis] Overall Protective Antigen Prediction = **0.4436** (Probable **ANTIGEN**).

>EHO9886432.1 carbon-nitrogen hydrolase family protein [Salmonella enterica subsp. enterica serovar Infantis] Overall Protective Antigen Prediction = **0.3859** (Probable **NON-ANTIGEN**).

>EHO9886433.1 exodeoxyribonuclease X [Salmonella enterica subsp. enterica serovar Infantis] Overall Protective Antigen Prediction = **0.3693** (Probable **NON-ANTIGEN**).

>EHO9886434.1 oligopeptidase B [Salmonella enterica subsp. enterica serovar Infantis] Overall Protective Antigen Prediction = **0.4599** (Probable **ANTIGEN**).

>EHO9886435.1 tellurite resistance TerB family protein [Salmonella enterica subsp. enterica serovar Infantis] Overall Protective Antigen Prediction = **0.4418** (Probable **ANTIGEN**).

>EHO9886436.1 hypothetical protein KND05_001770 [Salmonella enterica subsp. enterica serovar Infantis] Overall Protective Antigen Prediction = **0.7027** (Probable **ANTIGEN**).

>EHO9886437.1 DNA damage-inducible protein YebG [Salmonella enterica subsp. enterica serovar Infantis] Overall Protective Antigen Prediction = **0.3584** (Probable **NON-ANTIGEN**).

>EHO9886438.1 formate-dependent phosphoribosylglycinamide formyltransferase [Salmonella enterica subsp. enterica serovar Infantis] Overall Protective Antigen Prediction = **0.4731** (Probable **ANTIGEN**).

>EHO9886439.1 bifunctional 4-hydroxy-2-oxoglutarate aldolase/2-dehydro-3-deoxy-phosphogluconate aldolase [Salmonella enterica subsp. enterica serovar Infantis] Overall Protective Antigen Prediction = **0.3714** (Probable **NON-ANTIGEN**).

>EHO9886440.1 phosphogluconate dehydratase [Salmonella enterica subsp. enterica serovar Infantis] Overall Protective Antigen Prediction = **0.3842** (Probable **NON-ANTIGEN**).

>EHO9886441.1 glucose-6-phosphate dehydrogenase [Salmonella enterica subsp. enterica serovar Infantis] Overall Protective Antigen Prediction = **0.4291** (Probable **ANTIGEN**).

>EHO9886442.1 MurR/RpiR family transcriptional regulator [Salmonella enterica subsp. enterica serovar Infantis] Overall Protective Antigen Prediction = **0.3236** (Probable **NON-ANTIGEN**).

>EHO9886443.1 pyruvate kinase [Salmonella enterica subsp. enterica serovar Infantis] Overall Protective Antigen Prediction = **0.4326** (Probable **ANTIGEN**).

>EHO9886444.1 lauroyl-Kdo(2)-lipid IV(A) myristoyltransferase [Salmonella enterica subsp. enterica serovar Infantis] Overall Protective Antigen Prediction = **0.3086** (Probable **NON-ANTIGEN**).

>EHO9886445.1 murein DD-endopeptidase MepM [Salmonella enterica subsp. enterica serovar Infantis] Overall Protective Antigen Prediction = **0.5976** (Probable **ANTIGEN**).

>EHO9886446.1 zinc ABC transporter substrate-binding protein ZnuA [Salmonella enterica subsp. enterica serovar Infantis] Overall Protective Antigen Prediction = **0.5105** (Probable **ANTIGEN**).

>EHO9886447.1 zinc ABC transporter ATP-binding protein ZnuC [Salmonella enterica subsp. enterica serovar Infantis] Overall Protective Antigen Prediction = **0.4619** (Probable **ANTIGEN**).

>EHO9886448.1 zinc ABC transporter permease subunit ZnuB [Salmonella enterica subsp. enterica serovar Infantis] Overall Protective Antigen Prediction = **0.4749** (Probable **ANTIGEN**).

>EHO9886449.1 Holliday junction branch migration DNA helicase RuvB [Salmonella enterica subsp. enterica serovar Infantis] Overall Protective Antigen Prediction = **0.4090** (Probable **ANTIGEN**).

>EHO9886450.1 Holliday junction branch migration protein RuvA [Salmonella enterica subsp. enterica serovar Infantis] Overall Protective Antigen Prediction = **0.3751** (Probable **NON-ANTIGEN**).

>EHO9886451.1 hypothetical protein KND05_001785 [Salmonella enterica subsp. enterica serovar Infantis] Overall Protective Antigen Prediction = **0.4247** (Probable **ANTIGEN**).

>EHO9886452.1 hypothetical protein KND05_001786 [Salmonella enterica subsp. enterica serovar Infantis] Overall Protective Antigen Prediction = **1.1625** (Probable **ANTIGEN**).

>EHO9886453.1 YebB family permuted papain-like enzyme [Salmonella enterica subsp. enterica serovar Infantis] Overall Protective Antigen Prediction = **0.3581** (Probable **NON-ANTIGEN**).

>EHO9886454.1 crossover junction endodeoxyribonuclease RuvC [Salmonella enterica subsp. enterica serovar Infantis] Overall Protective Antigen Prediction = **0.3588** (Probable **NON-ANTIGEN**).

>EHO9886455.1 YebC/PmpR family DNA-binding transcriptional regulator [Salmonella enterica subsp. enterica serovar Infantis] Overall Protective Antigen Prediction = **0.5352** (Probable **ANTIGEN**).

>EHO9886456.1 dihydroneopterin triphosphate diphosphatase [Salmonella enterica subsp. enterica serovar Infantis] Overall Protective Antigen Prediction = **0.2902** (Probable **NON-ANTIGEN**).

>EHO9886457.1 aspartate--tRNA ligase [Salmonella enterica subsp. enterica serovar Infantis] Overall Protective Antigen Prediction = **0.4439** (Probable **ANTIGEN**).

>EHO9886458.1 hydrolase [Salmonella enterica subsp. enterica serovar Infantis] Overall Protective Antigen Prediction = **0.5093** (Probable **ANTIGEN**).

>EHO9886459.1 DUF72 domain-containing protein [Salmonella enterica subsp. enterica serovar Infantis] Overall Protective Antigen Prediction = **0.5229** (Probable **ANTIGEN**).

>EHO9886460.1 MAPEG family protein [Salmonella enterica subsp. enterica serovar Infantis] Overall Protective Antigen Prediction = **0.8677** (Probable **ANTIGEN**).

>EHO9886461.1 carboxy-S-adenosyl-L-methionine synthase CmoA [Salmonella enterica subsp. enterica serovar Infantis] Overall Protective Antigen Prediction = **0.4021** (Probable **ANTIGEN**).

>EHO9886462.1 tRNA 5-methoxyuridine(34)/uridine 5-oxyacetic acid(34) synthase CmoB [Salmonella enterica subsp. enterica serovar Infantis] Overall Protective Antigen Prediction = **0.2913** (Probable **NON-ANTIGEN**).

>EHO9886463.1 copper homeostasis protein CutC [Salmonella enterica subsp. enterica serovar

Infantis] Overall Protective Antigen Prediction = **0.4275** (Probable **ANTIGEN**).

>EHO9886464.1 VOC family protein [Salmonella enterica subsp. enterica serovar Infantis] Overall Protective Antigen Prediction = **0.7058** (Probable **ANTIGEN**).

>EHO9886465.1 arginine--tRNA ligase [Salmonella enterica subsp. enterica serovar Infantis] Overall Protective Antigen Prediction = **0.3642** (Probable **NON-ANTIGEN**).

>EHO9886466.1 penicillin-binding protein 2 [Salmonella enterica subsp. enterica serovar Infantis] Overall Protective Antigen Prediction = **0.4094** (Probable **ANTIGEN**).

>EHO9886467.1 glycoside hydrolase family 105 protein [Salmonella enterica subsp. enterica serovar Infantis] Overall Protective Antigen Prediction = **0.3370** (Probable **NON-ANTIGEN**).

>EHO9886468.1 flagellar protein FlhE [Salmonella enterica subsp. enterica serovar Infantis] Overall Protective Antigen Prediction = **0.9370** (Probable **ANTIGEN**).

>EHO9886469.1 flagellar biosynthesis protein FlhA [Salmonella enterica subsp. enterica serovar Infantis] Overall Protective Antigen Prediction = **0.5365** (Probable **ANTIGEN**).

>EHO9886470.1 flagellar type III secretion system protein FlhB [Salmonella enterica subsp. enterica serovar Infantis] Overall Protective Antigen Prediction = **0.4378** (Probable **ANTIGEN**).

>EHO9886471.1 protein phosphatase CheZ [Salmonella enterica subsp. enterica serovar Infantis] Overall Protective Antigen Prediction = **0.2839** (Probable **NON-ANTIGEN**).

>EHO9886472.1 chemotaxis response regulator CheY [Salmonella enterica subsp. enterica serovar Infantis] Overall Protective Antigen Prediction = **0.2397** (Probable **NON-ANTIGEN**).

>EHO9886473.1 protein-glutamate methylesterase/protein glutamine deamidase [Salmonella enterica subsp. enterica serovar Infantis] Overall Protective Antigen Prediction = **0.4767** (Probable **ANTIGEN**).

>EHO9886474.1 protein-glutamate O-methyltransferase CheR [Salmonella enterica subsp. enterica serovar Infantis] Overall Protective Antigen Prediction = **0.1975** (Probable **NON-ANTIGEN**).

>EHO9886475.1 methyl-accepting chemotaxis protein II [Salmonella enterica subsp. enterica serovar Infantis] Overall Protective Antigen Prediction = **0.4923** (Probable **ANTIGEN**).

>EHO9886476.1 chemotaxis protein CheW [Salmonella enterica subsp. enterica serovar Infantis] Overall Protective Antigen Prediction = **0.3551** (Probable **NON-ANTIGEN**).

>EHO9886477.1 chemotaxis protein CheA [Salmonella enterica subsp. enterica serovar Infantis] Overall Protective Antigen Prediction = **0.5637** (Probable **ANTIGEN**).

>EHO9886478.1 flagellar motor protein MotB [Salmonella enterica subsp. enterica serovar Infantis] Overall Protective Antigen Prediction = **0.5997** (Probable **ANTIGEN**).

>EHO9886479.1 flagellar motor stator protein MotA [Salmonella enterica subsp. enterica serovar Infantis] Overall Protective Antigen Prediction = **0.3311** (Probable **NON-ANTIGEN**).

>EHO9886480.1 flagellar transcriptional regulator FlhC [Salmonella enterica subsp. enterica serovar Infantis] Overall Protective Antigen Prediction = **0.2291** (Probable **NON-ANTIGEN**).

>EHO9886481.1 flagellar transcriptional regulator FlhD [Salmonella enterica subsp. enterica serovar Infantis] Overall Protective Antigen Prediction = **0.4118** (Probable **ANTIGEN**).

>EHO9886482.1 hypothetical protein KND05_001816 [Salmonella enterica subsp. enterica serovar Infantis] Overall Protective Antigen Prediction = **0.4187** (Probable **ANTIGEN**).

>EHO9886483.1 universal stress protein UspC [Salmonella enterica subsp. enterica serovar Infantis] Overall Protective Antigen Prediction = **0.1644** (Probable **NON-ANTIGEN**).

>EHO9886484.1 alpha,alpha-trehalose-phosphate synthase [Salmonella enterica subsp. enterica serovar Infantis] Overall Protective Antigen Prediction = **0.3977** (Probable **NON-ANTIGEN**).

>EHO9886485.1 trehalose-phosphatase [Salmonella enterica subsp. enterica serovar Infantis] Overall Protective Antigen Prediction = **0.5254** (Probable **ANTIGEN**).

>EHO9886486.1 DJ-1/PfpI family protein [Salmonella enterica subsp. enterica serovar Infantis] Overall Protective Antigen Prediction = **0.4300** (Probable **ANTIGEN**).

>EHO9886487.1 non-heme ferritin-like protein [Salmonella enterica subsp. enterica serovar Infantis] Overall Protective Antigen Prediction = **0.2976** (Probable **NON-ANTIGEN**).

>EHO9886488.1 RpiB/LacA/LacB family sugar-phosphate isomerase [Salmonella enterica subsp. enterica serovar Infantis] Overall Protective Antigen Prediction = **0.5971** (Probable **ANTIGEN**).

>EHO9886489.1 YecR-like lipofamily protein [Salmonella enterica subsp. enterica serovar Infantis] Overall Protective Antigen Prediction = **0.5751** (Probable **ANTIGEN**).

>EHO9886490.1 non-heme ferritin [Salmonella enterica subsp. enterica serovar Infantis] Overall Protective Antigen Prediction = **0.2120** (Probable **NON-ANTIGEN**).

>EHO9886491.1 YecH family protein [Salmonella enterica subsp. enterica serovar Infantis] Overall Protective Antigen Prediction = **0.5131** (Probable **ANTIGEN**).

>EHO9886492.1 tyrosine transporter TyrP [Salmonella enterica subsp. enterica serovar Infantis] Overall Protective Antigen Prediction = **0.5035** (Probable **ANTIGEN**).

>EHO9886493.1 YecA family protein [Salmonella enterica subsp. enterica serovar Infantis] Overall Protective Antigen Prediction = **0.4646** (Probable **ANTIGEN**).

>EHO9886494.1 glucose-6-phosphate dehydrogenase [Salmonella enterica subsp. enterica serovar Infantis] Overall Protective Antigen Prediction = **0.4339** (Probable **ANTIGEN**).

>EHO9886495.1 SH3 domain-containing protein [Salmonella enterica subsp. enterica serovar Infantis] Overall Protective Antigen Prediction = **0.4440** (Probable **ANTIGEN**).

>EHO9886496.1 hypothetical protein KND05_001831 [Salmonella enterica subsp. enterica serovar Infantis] Overall Protective Antigen Prediction = **0.4941** (Probable **ANTIGEN**).

>EHO9886497.1 CDP-diacylglycerol--glycerol-3-phosphate 3-phosphatidyltransferase [Salmonella enterica subsp. enterica serovar Infantis] Overall Protective Antigen Prediction = **0.4634** (Probable **ANTIGEN**).

>EHO9886498.1 excinuclease ABC subunit UvrC [Salmonella enterica subsp. enterica serovar Infantis] Overall Protective Antigen Prediction = **0.4074** (Probable **ANTIGEN**).

>EHO9886499.1 UvrY/SirA/GacA family response regulator transcription factor [Salmonella enterica subsp. enterica serovar Infantis] Overall Protective Antigen Prediction = **0.4532** (Probable **ANTIGEN**).

>EHO9886500.1 hypothetical protein KND05_001838 [Salmonella enterica subsp. enterica serovar Infantis] Overall Protective Antigen Prediction = **0.4504** (Probable **ANTIGEN**).

>EHO9886501.1 DUF2594 family protein [Salmonella enterica subsp. enterica serovar Infantis] Overall Protective Antigen Prediction = **0.4135** (Probable **ANTIGEN**).

>EHO9886502.1 transcriptional regulator SdiA [Salmonella enterica subsp. enterica serovar Infantis] Overall Protective Antigen Prediction = **0.3441** (Probable **NON-ANTIGEN**).

>EHO9886503.1 L-cystine ABC transporter ATP-binding protein YecC [Salmonella enterica subsp. enterica serovar Infantis] Overall Protective Antigen Prediction = **0.2374** (Probable **NON-ANTIGEN**).

>EHO9886504.1 cystine ABC transporter permease [Salmonella enterica subsp. enterica serovar Infantis] Overall Protective Antigen Prediction = **0.3961** (Probable **NON-ANTIGEN**).

>EHO9886505.1 D-cysteine desulfhydrase [Salmonella enterica subsp. enterica serovar Infantis] Overall Protective Antigen Prediction = **0.5279** (Probable **ANTIGEN**).

>EHO9886506.1 cystine ABC transporter substrate-binding protein [Salmonella enterica subsp. enterica serovar Infantis] Overall Protective Antigen Prediction = **0.5290** (Probable **ANTIGEN**).

>EHO9886507.1 flagella biosynthesis regulatory protein FliZ [Salmonella enterica subsp. enterica serovar Infantis] Overall Protective Antigen Prediction = **0.2562** (Probable **NON-ANTIGEN**).

>EHO9886508.1 RNA polymerase sigma factor FliA [Salmonella enterica subsp. enterica serovar Infantis] Overall Protective Antigen Prediction = **0.3188** (Probable **NON-ANTIGEN**).

>EHO9886509.1 flagellin lysine-N-methylase [Salmonella enterica subsp. enterica serovar Infantis] Overall Protective Antigen Prediction = **0.3771** (Probable **NON-ANTIGEN**).

>EHO9886510.1 flagellin FliC, partial [Salmonella enterica subsp. enterica serovar Infantis] Overall Protective Antigen Prediction = **0.8398** (Probable **ANTIGEN**).

>EHO9886511.1 myo-inositol import MFS transporter IolT2 [Salmonella enterica subsp. enterica serovar Infantis] Overall Protective Antigen Prediction = **0.5127** (Probable **ANTIGEN**).

>EHO9886512.1 hypothetical protein KND05_001850 [Salmonella enterica subsp. enterica serovar Infantis] Overall Protective Antigen Prediction = **0.9515** (Probable **ANTIGEN**).

>EHO9886513.1 myo-inositol import MFS transporter IolT1 [Salmonella enterica subsp. enterica serovar Infantis] Overall Protective Antigen Prediction = **0.5810** (Probable **ANTIGEN**).

>EHO9886514.1 myo-inositol utilization transcriptional regulator IolR [Salmonella enterica subsp. enterica serovar Infantis] Overall Protective Antigen Prediction = **0.3200** (Probable **NON-ANTIGEN**).

>EHO9886515.1 UDP-N-acetylmuramate:L-alanyl-gamma-D-glutamyl-meso-diaminopimelate ligase [Salmonella enterica subsp. enterica serovar Infantis] Overall Protective Antigen Prediction = **0.4399** (

Probable **ANTIGEN**).

>EHO9886516.1 class 1 fructose-bisphosphatase [Salmonella enterica subsp. enterica serovar Infantis] Overall Protective Antigen Prediction = **0.2700** (Probable **NON-ANTIGEN**).

>EHO9886517.1 inorganic diphosphatase [Salmonella enterica subsp. enterica serovar Infantis] Overall Protective Antigen Prediction = **0.3272** (Probable **NON-ANTIGEN**).

>EHO9886518.1 metallo-dependent hydrolase [Salmonella enterica subsp. enterica serovar Infantis] Overall Protective Antigen Prediction = **0.3748** (Probable **NON-ANTIGEN**).

>EHO9886519.1 MFS transporter [Salmonella enterica subsp. enterica serovar Infantis] Overall Protective Antigen Prediction = **0.4908** (Probable **ANTIGEN**).

>EHO9886520.1 gamma-glutamylcyclotransferase [Salmonella enterica subsp. enterica serovar Infantis] Overall Protective Antigen Prediction = **0.3549** (Probable **NON-ANTIGEN**).

>EHO9886521.1 autotransporter assembly complex protein TamB [Salmonella enterica subsp. enterica serovar Infantis] Overall Protective Antigen Prediction = **0.7566** (Probable **ANTIGEN**).

>EHO9886522.1 autotransporter assembly complex protein TamA [Salmonella enterica subsp. enterica serovar Infantis] Overall Protective Antigen Prediction = **0.5969** (Probable **ANTIGEN**).

>EHO9886523.1 peptide-methionine (S)-S-oxide reductase MsrA [Salmonella enterica subsp. enterica serovar Infantis] Overall Protective Antigen Prediction = **0.5272** (Probable **ANTIGEN**).

>EHO9886524.1 HlyC/CorC family transporter [Salmonella enterica subsp. enterica serovar Infantis] Overall Protective Antigen Prediction = **0.4608** (Probable **ANTIGEN**).

>EHO9886525.1 DUF1107 domain-containing protein [Salmonella enterica subsp. enterica serovar Infantis] Overall Protective Antigen Prediction = **-0.0872** (Probable **NON-ANTIGEN**).

>EHO9886526.1 3'(2'),5'-bisphosphate nucleotidase CysQ [Salmonella enterica subsp. enterica serovar Infantis] Overall Protective Antigen Prediction = **0.4711** (Probable **ANTIGEN**).

>EHO9886527.1 bifunctional 2',3'-cyclic-nucleotide 2'-phosphodiesterase/3'-nucleotidase [Salmonella enterica subsp. enterica serovar Infantis] Overall Protective Antigen Prediction = **0.4445** (Probable **ANTIGEN**).

>EHO9886528.1 winged helix-turn-helix transcriptional regulator [Salmonella enterica subsp. enterica serovar Infantis] Overall Protective Antigen Prediction = **0.3251** (Probable **NON-ANTIGEN**).

>EHO9886529.1 SDR family oxidoreductase [Salmonella enterica subsp. enterica serovar Infantis] Overall Protective Antigen Prediction = **0.2891** (Probable **NON-ANTIGEN**).

>EHO9886530.1 DMT family transporter [Salmonella enterica subsp. enterica serovar Infantis] Overall Protective Antigen Prediction = **0.4183** (Probable **ANTIGEN**).

>EHO9886531.1 iron-sulfur cluster repair protein YtfE [Salmonella enterica subsp. enterica serovar Infantis] Overall Protective Antigen Prediction = **0.2613** (Probable **NON-ANTIGEN**).

>EHO9886532.1 D-serine/D-alanine/glycine transporter [Salmonella enterica subsp. enterica serovar Infantis] Overall Protective Antigen Prediction = **0.4632** (Probable **ANTIGEN**).

>EHO9886533.1 FKBP-type peptidyl-prolyl cis-trans isomerase [Salmonella enterica subsp. enterica serovar Infantis] Overall Protective Antigen Prediction = **0.6564** (Probable **ANTIGEN**).

>EHO9886534.1 OapA family protein [Salmonella enterica subsp. enterica serovar Infantis] Overall Protective Antigen Prediction = **0.4841** (Probable **ANTIGEN**).

>EHO9886535.1 DMT family transporter [Salmonella enterica subsp. enterica serovar Infantis] Overall Protective Antigen Prediction = **0.4013** (Probable **ANTIGEN**).

>EHO9886536.1 50S ribosomal protein L9 [Salmonella enterica subsp. enterica serovar Infantis] Overall Protective Antigen Prediction = **0.4847** (Probable **ANTIGEN**).

>EHO9886537.1 30S ribosomal protein S18 [Salmonella enterica subsp. enterica serovar Infantis] Overall Protective Antigen Prediction = **0.2311** (Probable **NON-ANTIGEN**).

>EHO9886538.1 primosomal replication protein N [Salmonella enterica subsp. enterica serovar Infantis] Overall Protective Antigen Prediction = **0.4481** (Probable **ANTIGEN**).

>EHO9886539.1 30S ribosomal protein S6 [Salmonella enterica subsp. enterica serovar Infantis] Overall Protective Antigen Prediction = **0.5657** (Probable **ANTIGEN**).

>EHO9886540.1 hypothetical protein KND05_001879 [Salmonella enterica subsp. enterica serovar Infantis] Overall Protective Antigen Prediction = **0.5930** (Probable **ANTIGEN**).

>EHO9886541.1 DUF1471 domain-containing protein [Salmonella enterica subsp. enterica serovar Infantis] Overall Protective Antigen Prediction = **0.6925** (Probable **ANTIGEN**).

>EHO9886542.1 L-ribulose-5-phosphate 4-epimerase [Salmonella enterica subsp. enterica serovar Infantis] Overall Protective Antigen Prediction = **0.3948** (Probable **NON-ANTIGEN**).

>EHO9886543.1 L-ribulose-5-phosphate 3-epimerase UlaE [Salmonella enterica subsp. enterica serovar Infantis] Overall Protective Antigen Prediction = **0.3992** (Probable **NON-ANTIGEN**).

>EHO9886544.1 3-keto-L-gulonate-6-phosphate decarboxylase UlaD [Salmonella enterica subsp. enterica serovar Infantis] Overall Protective Antigen Prediction = **0.3505** (Probable **NON-ANTIGEN**).

>EHO9886545.1 PTS ascorbate transporter subunit IIA [Salmonella enterica subsp. enterica serovar Infantis] Overall Protective Antigen Prediction = **0.4810** (Probable **ANTIGEN**).

>EHO9886546.1 PTS ascorbate transporter subunit IIB [Salmonella enterica subsp. enterica serovar Infantis] Overall Protective Antigen Prediction = **0.6679** (Probable **ANTIGEN**).

>EHO9886547.1 PTS ascorbate transporter subunit IIC [Salmonella enterica subsp. enterica serovar Infantis] Overall Protective Antigen Prediction = **0.4571** (Probable **ANTIGEN**).

>EHO9886548.1 L-ascorbate 6-phosphate lactonase [Salmonella enterica subsp. enterica serovar Infantis] Overall Protective Antigen Prediction = **0.5330** (Probable **ANTIGEN**).

>EHO9886549.1 HTH-type transcriptional regulator UlaR [Salmonella enterica subsp. enterica serovar Infantis] Overall Protective Antigen Prediction = **0.3837** (Probable **NON-ANTIGEN**).

>EHO9886550.1 esterase [Salmonella enterica subsp. enterica serovar Infantis] Overall Protective Antigen Prediction = **0.4283** (Probable **ANTIGEN**).

>EHO9886551.1 biofilm peroxide resistance protein BsmA [Salmonella enterica subsp. enterica serovar Infantis] Overall Protective Antigen Prediction = **0.2932** (Probable **NON-ANTIGEN**).

>EHO9886552.1 DUF1471 domain-containing protein [Salmonella enterica subsp. enterica serovar Infantis] Overall Protective Antigen Prediction = **0.4927** (Probable **ANTIGEN**).

>EHO9886553.1 isovaleryl-CoA dehydrogenase [Salmonella enterica subsp. enterica serovar Infantis] Overall Protective Antigen Prediction = **0.4863** (Probable **ANTIGEN**).

>EHO9886554.1 glutathionylspermidine synthase family protein [Salmonella enterica subsp. enterica serovar Infantis] Overall Protective Antigen Prediction = **0.4997** (Probable **ANTIGEN**).

>EHO9886555.1 DUF1190 domain-containing protein [Salmonella enterica subsp. enterica serovar Infantis] Overall Protective Antigen Prediction = **0.7092** (Probable **ANTIGEN**).

>EHO9886556.1 DUF350 domain-containing protein [Salmonella enterica subsp. enterica serovar Infantis] Overall Protective Antigen Prediction = **0.4852** (Probable **ANTIGEN**).

>EHO9886557.1 YjfK family protein [Salmonella enterica subsp. enterica serovar Infantis] Overall Protective Antigen Prediction = **0.5261** (Probable **ANTIGEN**).

>EHO9886558.1 PspA/IM30 family protein [Salmonella enterica subsp. enterica serovar Infantis] Overall Protective Antigen Prediction = **0.5317** (Probable **ANTIGEN**).

>EHO9886559.1 DUF2170 family protein [Salmonella enterica subsp. enterica serovar Infantis] Overall Protective Antigen Prediction = **0.2949** (Probable **NON-ANTIGEN**).

>EHO9886560.1 23S rRNA (guanosine(2251)-2'-O)-methyltransferase RlmB [Salmonella enterica subsp. enterica serovar Infantis] Overall Protective Antigen Prediction = **0.4625** (Probable **ANTIGEN**).

>EHO9886561.1 ribonuclease R [Salmonella enterica subsp. enterica serovar Infantis] Overall Protective Antigen Prediction = **0.5660** (Probable **ANTIGEN**).

>EHO9886562.1 nitric oxide-sensing transcriptional repressor NsrR [Salmonella enterica subsp. enterica serovar Infantis] Overall Protective Antigen Prediction = **0.1892** (Probable **NON-ANTIGEN**).

>EHO9886563.1 adenylosuccinate synthase [Salmonella enterica subsp. enterica serovar Infantis] Overall Protective Antigen Prediction = **0.3843** (Probable **NON-ANTIGEN**).

>EHO9886564.1 DUF2065 family protein [Salmonella enterica subsp. enterica serovar Infantis] Overall Protective Antigen Prediction = **0.0305** (Probable **NON-ANTIGEN**).

>EHO9886565.1 protease modulator HflC [Salmonella enterica subsp. enterica serovar Infantis] Overall Protective Antigen Prediction = **0.6421** (Probable **ANTIGEN**).

>EHO9886566.1 FtsH protease activity modulator HflK [Salmonella enterica subsp. enterica serovar Infantis] Overall Protective Antigen Prediction = **0.7032** (Probable **ANTIGEN**).

>EHO9886567.1 GTPase HflX [Salmonella enterica subsp. enterica serovar Infantis] Overall Protective Antigen Prediction = **0.4692** (Probable **ANTIGEN**).

>EHO9886568.1 RNA chaperone Hfq [Salmonella enterica subsp. enterica serovar Infantis] Overall Protective Antigen Prediction = **0.6656** (Probable **ANTIGEN**).

>EHO9886569.1 tRNA (adenosine(37)-N6)-dimethylallyltransferase MiaA [Salmonella enterica subsp. enterica serovar Infantis] Overall Protective Antigen Prediction = **0.4171** (Probable **ANTIGEN**).

>EHO9886570.1 DNA mismatch repair endonuclease MutL [Salmonella enterica subsp. enterica serovar Infantis] Overall Protective Antigen Prediction = **0.5582** (Probable **ANTIGEN**).

>EHO9886571.1 N-acetylmuramoyl-L-alanine amidase AmiB [Salmonella enterica subsp. enterica serovar Infantis] Overall Protective Antigen Prediction = **0.6146** (Probable **ANTIGEN**).

>EHO9886572.1 tRNA (adenosine(37)-N6)-threonylcarbamoyltransferase complex ATPase subunit type 1 TsaE [Salmonella enterica subsp. enterica serovar Infantis] Overall Protective Antigen Prediction = **0.5138** (Probable **ANTIGEN**).

>EHO9886573.1 bifunctional ADP-dependent NAD(P)H-hydrate dehydratase/NAD(P)H-hydrate epimerase [Salmonella enterica subsp. enterica serovar Infantis] Overall Protective Antigen Prediction = **0.4267** (Probable **ANTIGEN**).

>EHO9886574.1 tRNA epoxyqueuosine(34) reductase QueG [Salmonella enterica subsp. enterica serovar Infantis] Overall Protective Antigen Prediction = **0.3494** (Probable **NON-ANTIGEN**).

>EHO9886575.1 arginine ABC transporter substrate-binding protein [Salmonella enterica subsp. enterica serovar Infantis] Overall Protective Antigen Prediction = **0.5308** (Probable **ANTIGEN**).

>EHO9886576.1 oligoribonuclease [Salmonella enterica subsp. enterica serovar Infantis] Overall Protective Antigen Prediction = **0.5042** (Probable **ANTIGEN**).

>EHO9886577.1 small ribosomal subunit biogenesis GTPase RsgA [Salmonella enterica subsp. enterica serovar Infantis] Overall Protective Antigen Prediction = **0.4558** (Probable **ANTIGEN**).

>EHO9886578.1 phosphatidylserine decarboxylase [Salmonella enterica subsp. enterica serovar Infantis] Overall Protective Antigen Prediction = **0.2700** (Probable **NON-ANTIGEN**).

>EHO9886579.1 miniconductance mechanosensitive channel MscM [Salmonella enterica subsp. enterica serovar Infantis] Overall Protective Antigen Prediction = **0.5018** (Probable **ANTIGEN**).

>EHO9886580.1 YjeO family protein [Salmonella enterica subsp. enterica serovar Infantis] Overall Protective Antigen Prediction = **0.9401** (Probable **ANTIGEN**).

>EHO9886581.1 glutamate/gamma-aminobutyrate family transporter YjeM [Salmonella enterica subsp. enterica serovar Infantis] Overall Protective Antigen Prediction = **0.4764** (Probable **ANTIGEN**).

>EHO9886582.1 elongation factor P--(R)-beta-lysine ligase [Salmonella enterica subsp. enterica serovar Infantis] Overall Protective Antigen Prediction = **0.5218** (Probable **ANTIGEN**).

>EHO9886583.1 fumarate reductase (quinol) flavoprotein subunit [Salmonella enterica subsp. enterica serovar Infantis] Overall Protective Antigen Prediction = **0.5839** (Probable **ANTIGEN**).

>EHO9886584.1 fumarate reductase iron-sulfur protein [Salmonella enterica subsp. enterica serovar Infantis] Overall Protective Antigen Prediction = **0.4824** (Probable **ANTIGEN**).

>EHO9886585.1 fumarate reductase subunit FrdC [Salmonella enterica subsp. enterica serovar Infantis] Overall Protective Antigen Prediction = **0.3963** (Probable **NON-ANTIGEN**).

>EHO9886586.1 fumarate reductase subunit FrdD [Salmonella enterica subsp. enterica serovar Infantis] Overall Protective Antigen Prediction = **0.2714** (Probable **NON-ANTIGEN**).

>EHO9886587.1 lipocalin family protein [Salmonella enterica subsp. enterica serovar Infantis] Overall Protective Antigen Prediction = **0.2824** (Probable **NON-ANTIGEN**).

>EHO9886588.1 quaternary ammonium compound efflux SMR transporter SugE [Salmonella enterica subsp. enterica serovar Infantis] Overall Protective Antigen Prediction = **0.4380** (Probable **ANTIGEN**).

>EHO9886589.1 response regulator transcription factor [Salmonella enterica subsp. enterica serovar Infantis] Overall Protective Antigen Prediction = **0.4070** (Probable **ANTIGEN**).

>EHO9886590.1 lipoprotein toxin entericidin B [Salmonella enterica subsp. enterica serovar Infantis] Overall Protective Antigen Prediction = **0.5975** (Probable **ANTIGEN**).

>EHO9886591.1 entericidin A/B family lipoprotein [Salmonella enterica subsp. enterica serovar Infantis] Overall Protective Antigen Prediction = **-0.0775** (Probable **NON-ANTIGEN**).

>EHO9886592.1 elongation factor P [Salmonella enterica subsp. enterica serovar Infantis] Overall Protective Antigen Prediction = **0.5698** (Probable **ANTIGEN**).

>EHO9886593.1 EF-P beta-lysylation protein EpmB [Salmonella enterica subsp. enterica serovar Infantis] Overall Protective Antigen Prediction = **0.4006** (Probable **ANTIGEN**).

>EHO9886594.1 YjeJ family protein [Salmonella enterica subsp. enterica serovar Infantis] Overall Protective Antigen Prediction = **0.7415** (Probable **ANTIGEN**).

>EHO9886595.1 DUF4156 domain-containing protein [Salmonella enterica subsp. enterica serovar Infantis] Overall Protective Antigen Prediction = **0.7770** (Probable **ANTIGEN**).

>EHO9886596.1 chaperonin GroEL [Salmonella enterica subsp. enterica serovar Infantis] Overall Protective Antigen Prediction = **0.5817** (Probable **ANTIGEN**).

>EHO9886597.1 co-chaperone GroES [Salmonella enterica subsp. enterica serovar Infantis] Overall Protective Antigen Prediction = **0.6701** (Probable **ANTIGEN**).

>EHO9886598.1 L-methionine/branched-chain amino acid transporter [Salmonella enterica subsp. enterica serovar Infantis] Overall Protective Antigen Prediction = **0.5313** (Probable **ANTIGEN**).

>EHO9886599.1 membrane protein FxsA [Salmonella enterica subsp. enterica serovar Infantis] Overall Protective Antigen Prediction = **0.8527** (Probable **ANTIGEN**).

>EHO9886600.1 aspartate ammonia-lyase [Salmonella enterica subsp. enterica serovar Infantis] Overall Protective Antigen Prediction = **0.4070** (Probable **ANTIGEN**).

>EHO9886601.1 anaerobic C4-dicarboxylate transporter DcuA [Salmonella enterica subsp. enterica serovar Infantis] Overall Protective Antigen Prediction = **0.5132** (Probable **ANTIGEN**).

>EHO9886602.1 divalent cation tolerance protein CutA [Salmonella enterica subsp. enterica serovar

Infantis] Overall Protective Antigen Prediction = **0.4606** (Probable **ANTIGEN**).

>EHO9886603.1 protein-disulfide reductase DsbD [Salmonella enterica subsp. enterica serovar Infantis] Overall Protective Antigen Prediction = **0.5276** (Probable **ANTIGEN**).

>EHO9886604.1 transcriptional regulator [Salmonella enterica subsp. enterica serovar Infantis] Overall Protective Antigen Prediction = **0.1119** (Probable **NON-ANTIGEN**).

>EHO9886605.1 non-specific acid phosphatase [Salmonella enterica subsp. enterica serovar Infantis] Overall Protective Antigen Prediction = **0.5079** (Probable **ANTIGEN**).

>EHO9886606.1 DUF1202 family protein [Salmonella enterica subsp. enterica serovar Infantis] Overall Protective Antigen Prediction = **0.5123** (Probable **ANTIGEN**).

>EHO9886607.1 hypothetical protein KND05_001954 [Salmonella enterica subsp. enterica serovar Infantis] Overall Protective Antigen Prediction = **0.2942** (Probable **NON-ANTIGEN**).

>EHO9886608.1 AraC family transcriptional regulator [Salmonella enterica subsp. enterica serovar Infantis] Overall Protective Antigen Prediction = **0.3506** (Probable **NON-ANTIGEN**).

>EHO9886609.1 spore gernimation protein GerE [Salmonella enterica subsp. enterica serovar Infantis] Overall Protective Antigen Prediction = **0.7849** (Probable **ANTIGEN**).

>EHO9886610.1 ATP-binding cassette domain-containing protein [Salmonella enterica subsp. enterica serovar Infantis] Overall Protective Antigen Prediction = **0.4170** (Probable **ANTIGEN**).

>EHO9886611.1 hypothetical protein KND05_001958 [Salmonella enterica subsp. enterica serovar Infantis] Overall Protective Antigen Prediction = **0.4747** (Probable **ANTIGEN**).

>EHO9886612.1 hypothetical protein KND05_001959 [Salmonella enterica subsp. enterica serovar Infantis] Overall Protective Antigen Prediction = **0.6568** (Probable **ANTIGEN**).

>EHO9886613.1 YjiK family protein [Salmonella enterica subsp. enterica serovar Infantis] Overall Protective Antigen Prediction = **0.4090** (Probable **ANTIGEN**).

>EHO9886614.1 DUF1996 domain-containing protein [Salmonella enterica subsp. enterica serovar Infantis] Overall Protective Antigen Prediction = **0.6421** (Probable **ANTIGEN**).

>EHO9886615.1 molecular chaperone [Salmonella enterica subsp. enterica serovar Infantis] Overall Protective Antigen Prediction = **0.2481** (Probable **NON-ANTIGEN**).

>EHO9886616.1 dimethyl sulfoxide reductase anchor subunit [Salmonella enterica subsp. enterica serovar Infantis] Overall Protective Antigen Prediction = **0.3221** (Probable **NON-ANTIGEN**).

>EHO9886617.1 dimethylsulfoxide reductase subunit B [Salmonella enterica subsp. enterica serovar Infantis] Overall Protective Antigen Prediction = **0.6607** (Probable **ANTIGEN**).

>EHO9886618.1 molybdopterin-dependent oxidoreductase [Salmonella enterica subsp. enterica serovar Infantis] Overall Protective Antigen Prediction = **0.4959** (Probable **ANTIGEN**).

>EHO9886619.1 two-component system sensor histidine kinase DcuS [Salmonella enterica subsp. enterica serovar Infantis] Overall Protective Antigen Prediction = **0.2788** (Probable **NON-ANTIGEN**).

>EHO9886620.1 two-component system response regulator DcuR [Salmonella enterica subsp. enterica serovar Infantis] Overall Protective Antigen Prediction = **0.4794** (Probable **ANTIGEN**).

>EHO9886621.1 hypothetical protein KND05_001968 [Salmonella enterica subsp. enterica serovar Infantis] Overall Protective Antigen Prediction = **0.2785** (Probable **NON-ANTIGEN**).

>EHO9886622.1 anaerobic C4-dicarboxylate transporter DcuB [Salmonella enterica subsp. enterica serovar Infantis] Overall Protective Antigen Prediction = **0.4854** (Probable **ANTIGEN**).

>EHO9886623.1 fumarate hydratase [Salmonella enterica subsp. enterica serovar Infantis] Overall Protective Antigen Prediction = **0.4431** (Probable **ANTIGEN**).

>EHO9886624.1 melibiose:sodium transporter MelB [Salmonella enterica subsp. enterica serovar Infantis] Overall Protective Antigen Prediction = **0.5187** (Probable **ANTIGEN**).

>EHO9886625.1 alpha-glucosidase/alpha-galactosidase [Salmonella enterica subsp. enterica serovar Infantis] Overall Protective Antigen Prediction = **0.4696** (Probable **ANTIGEN**).

>EHO9886626.1 transcriptional regulator MelR [Salmonella enterica subsp. enterica serovar Infantis] Overall Protective Antigen Prediction = **0.4200** (Probable **ANTIGEN**).

>EHO9886627.1 arginine decarboxylase [Salmonella enterica subsp. enterica serovar Infantis] Overall Protective Antigen Prediction = **0.3736** (Probable **NON-ANTIGEN**).

>EHO9886628.1 AraC family transcriptional regulator [Salmonella enterica subsp. enterica serovar Infantis] Overall Protective Antigen Prediction = **0.2103** (Probable **NON-ANTIGEN**).

>EHO9886629.1 arginine/agmatine antiporter [Salmonella enterica subsp. enterica serovar Infantis] Overall Protective Antigen Prediction = **0.4922** (Probable **ANTIGEN**).

>EHO9886630.1 phosphoethanolamine transferase EptA [Salmonella enterica subsp. enterica serovar Infantis] Overall Protective Antigen Prediction = **0.4402** (Probable **ANTIGEN**).

>EHO9886631.1 two-component system response regulator PmrA [Salmonella enterica subsp. enterica serovar Infantis] Overall Protective Antigen Prediction = **0.5035** (Probable **ANTIGEN**).

>EHO9886632.1 two-component system sensor histidine kinase PmrB [Salmonella enterica subsp. enterica serovar Infantis] Overall Protective Antigen Prediction = **0.3655** (Probable **NON-ANTIGEN**).

>EHO9886633.1 LpxT activity modulator PmrR [Salmonella enterica subsp. enterica serovar Infantis] Overall Protective Antigen Prediction = **0.2283** (Probable **NON-ANTIGEN**).

>EHO9886634.1 glycine betaine/L-proline transporter ProP [Salmonella enterica subsp. enterica serovar Infantis] Overall Protective Antigen Prediction = **0.4862** (Probable **ANTIGEN**).

>EHO9886635.1 phnA family protein [Salmonella enterica subsp. enterica serovar Infantis] Overall Protective Antigen Prediction = **0.6420** (Probable **ANTIGEN**).

>EHO9886636.1 VOC family metalloprotein YjdN [Salmonella enterica subsp. enterica serovar Infantis] Overall Protective Antigen Prediction = **0.5595** (Probable **ANTIGEN**).

>EHO9886637.1 aminoalkylphosphonate N-acetyltransferase [Salmonella enterica subsp. enterica serovar Infantis] Overall Protective Antigen Prediction = **0.6017** (Probable **ANTIGEN**).

>EHO9886638.1 lipid A hydroxylase LpxO [Salmonella enterica subsp. enterica serovar Infantis] Overall Protective Antigen Prediction = **0.3506** (Probable **NON-ANTIGEN**).

>EHO9886639.1 formate dehydrogenase H subunit alpha, selenocysteine-containing [Salmonella enterica subsp. enterica serovar Infantis] Overall Protective Antigen Prediction = **0.4592** (Probable **ANTIGEN**).

>EHO9886640.1 sel1 repeat family protein [Salmonella enterica subsp. enterica serovar Infantis] Overall Protective Antigen Prediction = **0.5056** (Probable **ANTIGEN**).

>EHO9886641.1 glutamate/aspartate:proton symporter GltP [Salmonella enterica subsp. enterica serovar Infantis] Overall Protective Antigen Prediction = **0.4166** (Probable **ANTIGEN**).

>EHO9886642.1 heme lyase NrfEFG subunit NrfG [Salmonella enterica subsp. enterica serovar Infantis] Overall Protective Antigen Prediction = **0.3363** (Probable **NON-ANTIGEN**).

>EHO9886643.1 heme lyase NrfEFG subunit NrfF [Salmonella enterica subsp. enterica serovar Infantis] Overall Protective Antigen Prediction = **0.5524** (Probable **ANTIGEN**).

>EHO9886644.1 heme lyase CcmF/NrfE family subunit [Salmonella enterica subsp. enterica serovar Infantis] Overall Protective Antigen Prediction = **0.5057** (Probable **ANTIGEN**).

>EHO9886645.1 cytochrome c nitrite reductase subunit NrfD [Salmonella enterica subsp. enterica serovar Infantis] Overall Protective Antigen Prediction = **0.6993** (Probable **ANTIGEN**).

>EHO9886646.1 cytochrome c nitrite reductase Fe-S protein [Salmonella enterica subsp. enterica serovar Infantis] Overall Protective Antigen Prediction = **0.4612** (Probable **ANTIGEN**).

>EHO9886647.1 cytochrome c nitrite reductase pentaheme subunit [Salmonella enterica subsp. enterica serovar Infantis] Overall Protective Antigen Prediction = **0.5158** (Probable **ANTIGEN**).

>EHO9886648.1 ammonia-forming nitrite reductase cytochrome c552 subunit [Salmonella enterica subsp. enterica serovar Infantis] Overall Protective Antigen Prediction = **0.5988** (Probable **ANTIGEN**).

>EHO9886649.1 acetate--CoA ligase [Salmonella enterica subsp. enterica serovar Infantis] Overall Protective Antigen Prediction = **0.4172** (Probable **ANTIGEN**).

>EHO9886650.1 DUF485 domain-containing protein [Salmonella enterica subsp. enterica serovar Infantis] Overall Protective Antigen Prediction = **0.4403** (Probable **ANTIGEN**).

>EHO9886651.1 cation/acetate symporter ActP [Salmonella enterica subsp. enterica serovar Infantis] Overall Protective Antigen Prediction = **0.4947** (Probable **ANTIGEN**).

>EHO9886652.1 LrgB family protein [Salmonella enterica subsp. enterica serovar Infantis] Overall Protective Antigen Prediction = **0.6050** (Probable **ANTIGEN**).

>EHO9886653.1 CidA/LrgA family protein [Salmonella enterica subsp. enterica serovar Infantis] Overall Protective Antigen Prediction = **0.3720** (Probable **NON-ANTIGEN**).

>EHO9886654.1 LysR family transcriptional regulator [Salmonella enterica subsp. enterica serovar Infantis] Overall Protective Antigen Prediction = **0.2937** (Probable **NON-ANTIGEN**).

>EHO9886655.1 Na+/H+ antiporter [Salmonella enterica subsp. enterica serovar Infantis] Overall Protective Antigen Prediction = **0.6259** (Probable **ANTIGEN**).

>EHO9886656.1 guanine/hypoxanthine transporter GhxP [Salmonella enterica subsp. enterica serovar Infantis] Overall Protective Antigen Prediction = **0.4876** (Probable **ANTIGEN**).

>EHO9886657.1 glutathione S-transferase [Salmonella enterica subsp. enterica serovar Infantis] Overall Protective Antigen Prediction = **0.3680** (Probable **NON-ANTIGEN**).

>EHO9886658.1 redox-sensitive transcriptional activator SoxR [Salmonella enterica subsp. enterica serovar Infantis] Overall Protective Antigen Prediction = **0.4788** (Probable **ANTIGEN**).

>EHO9886659.1 superoxide response transcriptional regulator SoxS [Salmonella enterica subsp. enterica serovar Infantis] Overall Protective Antigen Prediction = **0.2779** (Probable **NON-ANTIGEN**).

>EHO9886660.1 EAL domain-containing protein [Salmonella enterica subsp. enterica serovar Infantis] Overall Protective Antigen Prediction = **0.2436** (Probable **NON-ANTIGEN**).

>EHO9886661.1 YjcB family protein [Salmonella enterica subsp. enterica serovar Infantis] Overall Protective Antigen Prediction = **0.5035** (Probable **ANTIGEN**).

>EHO9886662.1 SPI-4 type I secretion system protein SiiF [Salmonella enterica subsp. enterica serovar Infantis] Overall Protective Antigen Prediction = **0.5203** (Probable **ANTIGEN**).

>EHO9886663.1 non-fimbrial adhesin SiiE [Salmonella enterica subsp. enterica serovar Infantis] Overall Protective Antigen Prediction = **0.7610** (Probable **ANTIGEN**).

>EHO9886664.1 SPI-4 type I secretion system protein SiiD [Salmonella enterica subsp. enterica serovar Infantis] Overall Protective Antigen Prediction = **0.5770** (Probable **ANTIGEN**).

>EHO9886665.1 SPI-4 type I secretion system protein SiiC [Salmonella enterica subsp. enterica serovar Infantis] Overall Protective Antigen Prediction = **0.6315** (Probable **ANTIGEN**).

>EHO9886666.1 MotA/TolQ/ExbB proton channel family protein [Salmonella enterica subsp. enterica serovar Infantis] Overall Protective Antigen Prediction = **0.4515** (Probable **ANTIGEN**).

>EHO9886667.1 SPI-4 type I secretion system auxiliary protein SiiA [Salmonella enterica subsp. enterica serovar Infantis] Overall Protective Antigen Prediction = **0.7115** (Probable **ANTIGEN**).

>EHO9886668.1 single-stranded DNA-binding protein SSB1 [Salmonella enterica subsp. enterica serovar Infantis] Overall Protective Antigen Prediction = **0.6468** (Probable **ANTIGEN**).

>EHO9886669.1 excinuclease ABC subunit UvrA [Salmonella enterica subsp. enterica serovar Infantis] Overall Protective Antigen Prediction = **0.5499** (Probable **ANTIGEN**).

>EHO9886670.1 hypothetical protein KND05_002017 [Salmonella enterica subsp. enterica serovar Infantis] Overall Protective Antigen Prediction = **0.4758** (Probable **ANTIGEN**).

>EHO9886671.1 hypothetical protein KND05_002018 [Salmonella enterica subsp. enterica serovar Infantis] Overall Protective Antigen Prediction = **0.1602** (Probable **NON-ANTIGEN**).

>EHO9886672.1 MmcQ/YjbR family DNA-binding protein [Salmonella enterica subsp. enterica serovar Infantis] Overall Protective Antigen Prediction = **0.5335** (Probable **ANTIGEN**).

>EHO9886673.1 YjbQ family protein [Salmonella enterica subsp. enterica serovar Infantis] Overall Protective Antigen Prediction = **0.2529** (Probable **NON-ANTIGEN**).

>EHO9886674.1 acid phosphatase AphA [Salmonella enterica subsp. enterica serovar Infantis] Overall Protective Antigen Prediction = **0.3550** (Probable **NON-ANTIGEN**).

>EHO9886675.1 aromatic amino acid transaminase [Salmonella enterica subsp. enterica serovar Infantis] Overall Protective Antigen Prediction = **0.4062** (Probable **ANTIGEN**).

>EHO9886676.1 KilA-N domain-containing protein [Salmonella enterica subsp. enterica serovar Infantis] Overall Protective Antigen Prediction = **0.5057** (Probable **ANTIGEN**).

>EHO9886677.1 hypothetical protein KND05_002024 [Salmonella enterica subsp. enterica serovar Infantis] Overall Protective Antigen Prediction = **0.4231** (Probable **ANTIGEN**).

>EHO9886678.1 hypothetical protein KND05_002025 [Salmonella enterica subsp. enterica serovar Infantis] Overall Protective Antigen Prediction = **0.3243** (Probable **NON-ANTIGEN**).

>EHO9886679.1 hypothetical protein KND05_002026 [Salmonella enterica subsp. enterica serovar Infantis] Overall Protective Antigen Prediction = **0.4310** (Probable **ANTIGEN**).

>EHO9886680.1 DUF1983 domain-containing protein [Salmonella enterica subsp. enterica serovar Infantis] Overall Protective Antigen Prediction = **0.4810** (Probable **ANTIGEN**).

>EHO9886681.1 glycosyltransferase family 2 protein [Salmonella enterica subsp. enterica serovar Infantis] Overall Protective Antigen Prediction = **0.3917** (Probable **NON-ANTIGEN**).

>EHO9886682.1 HNH endonuclease [Salmonella enterica subsp. enterica serovar Infantis] Overall Protective Antigen Prediction = **0.4916** (Probable **ANTIGEN**).

>EHO9886683.1 hypothetical protein KND05_002030 [Salmonella enterica subsp. enterica serovar Infantis] Overall Protective Antigen Prediction = **0.3188** (Probable **NON-ANTIGEN**).

>EHO9886684.1 HNH endonuclease [Salmonella enterica subsp. enterica serovar Infantis] Overall Protective Antigen Prediction = **0.9444** (Probable **ANTIGEN**).

>EHO9886685.1 terminase small subunit [Salmonella enterica subsp. enterica serovar Infantis] Overall Protective Antigen Prediction = **0.7459** (Probable **ANTIGEN**).

>EHO9886686.1 terminase large subunit [Salmonella enterica subsp. enterica serovar Infantis] Overall Protective Antigen Prediction = **0.4287** (Probable **ANTIGEN**).

>EHO9886687.1 phage major capsid protein [Salmonella enterica subsp. enterica serovar Infantis] Overall Protective Antigen Prediction = **0.4498** (Probable **ANTIGEN**).

>EHO9886688.1 phage gp6-like head-tail connector protein [Salmonella enterica subsp. enterica serovar Infantis] Overall Protective Antigen Prediction = **0.4141** (Probable **ANTIGEN**).

>EHO9886689.1 phage head closure protein [Salmonella enterica subsp. enterica serovar Infantis] Overall Protective Antigen Prediction = **0.8339** (Probable **ANTIGEN**).

>EHO9886690.1 HK97 gp10 family phage protein [Salmonella enterica subsp. enterica serovar Infantis] Overall Protective Antigen Prediction = **0.7160** (Probable **ANTIGEN**).

>EHO9886691.1 DUF3168 domain-containing protein [Salmonella enterica subsp. enterica serovar Infantis] Overall Protective Antigen Prediction = **0.7304** (Probable **ANTIGEN**).

>EHO9886692.1 phage tail protein [Salmonella enterica subsp. enterica serovar Infantis] Overall Protective Antigen Prediction = **0.8449** (Probable **ANTIGEN**).

>EHO9886693.1 phage tail assembly chaperone [Salmonella enterica subsp. enterica serovar Infantis] Overall Protective Antigen Prediction = **0.4173** (Probable **ANTIGEN**).

>EHO9886694.1 DUF4035 domain-containing protein [Salmonella enterica subsp. enterica serovar Infantis] Overall Protective Antigen Prediction = **0.4646** (Probable **ANTIGEN**).

>EHO9886695.1 hypothetical protein KND05_002043 [Salmonella enterica subsp. enterica serovar Infantis] Overall Protective Antigen Prediction = **0.3800** (Probable **NON-ANTIGEN**).

>EHO9886696.1 phage tail tape measure protein [Salmonella enterica subsp. enterica serovar Infantis] Overall Protective Antigen Prediction = **0.5431** (Probable **ANTIGEN**).

>EHO9886697.1 hypothetical protein KND05_002045 [Salmonella enterica subsp. enterica serovar Infantis] Overall Protective Antigen Prediction = **0.2496** (Probable **NON-ANTIGEN**).

>EHO9886698.1 hypothetical protein KND05_002046 [Salmonella enterica subsp. enterica serovar Infantis] Overall Protective Antigen Prediction = **0.6256** (Probable **ANTIGEN**).

>EHO9886699.1 hypothetical protein KND05_002047 [Salmonella enterica subsp. enterica serovar Infantis] Overall Protective Antigen Prediction = **0.4443** (Probable **ANTIGEN**).

>EHO9886700.1 hypothetical protein KND05_002048 [Salmonella enterica subsp. enterica serovar Infantis] Overall Protective Antigen Prediction = **0.4613** (Probable **ANTIGEN**).

>EHO9886701.1 DUF1983 domain-containing protein [Salmonella enterica subsp. enterica serovar Infantis] Overall Protective Antigen Prediction = **0.5247** (Probable **ANTIGEN**).

>EHO9886702.1 hypothetical protein KND05_002050 [Salmonella enterica subsp. enterica serovar Infantis] Overall Protective Antigen Prediction = **0.5158** (Probable **ANTIGEN**).

>EHO9886703.1 phage tail protein [Salmonella enterica subsp. enterica serovar Infantis] Overall Protective Antigen Prediction = **0.5369** (Probable **ANTIGEN**).

>EHO9886704.1 tyrosine-type recombinase/integrase [Salmonella enterica subsp. enterica serovar Infantis] Overall Protective Antigen Prediction = **0.4456** (Probable **ANTIGEN**).

>EHO9886705.1 formate/nitrite transporter family protein [Salmonella enterica subsp. enterica serovar Infantis] Overall Protective Antigen Prediction = **0.7038** (Probable **ANTIGEN**).

>EHO9886706.1 phospholipid-binding lipoprotein MlaA [Salmonella enterica subsp. enterica serovar Infantis] Overall Protective Antigen Prediction = **0.5458** (Probable **ANTIGEN**).

>EHO9886707.1 long-chain fatty acid transporter FadL [Salmonella enterica subsp. enterica serovar Infantis] Overall Protective Antigen Prediction = **0.6017** (Probable **ANTIGEN**).

>EHO9886708.1 YfcZ/YiiS family protein [Salmonella enterica subsp. enterica serovar Infantis] Overall Protective Antigen Prediction = **0.7219** (Probable **ANTIGEN**).

>EHO9886709.1 acetyl-CoA C-acyltransferase FadI [Salmonella enterica subsp. enterica serovar Infantis] Overall Protective Antigen Prediction = **0.3729** (Probable **NON-ANTIGEN**).

>EHO9886710.1 fatty acid oxidation complex subunit alpha FadJ [Salmonella enterica subsp. enterica serovar Infantis] Overall Protective Antigen Prediction = **0.5107** (Probable **ANTIGEN**).

>EHO9886711.1 phosphohistidine phosphatase SixA [Salmonella enterica subsp. enterica serovar Infantis] Overall Protective Antigen Prediction = **0.4782** (Probable **ANTIGEN**).

>EHO9886712.1 endonuclease SmrB [Salmonella enterica subsp. enterica serovar Infantis] Overall Protective Antigen Prediction = **0.2986** (Probable **NON-ANTIGEN**).

>EHO9886713.1 50S ribosomal protein L3 N(5)-glutamine methyltransferase [Salmonella enterica subsp. enterica serovar Infantis] Overall Protective Antigen Prediction = **0.3056** (Probable **NON-ANTIGEN**).

>EHO9886714.1 chorismate synthase [Salmonella enterica subsp. enterica serovar Infantis] Overall Protective Antigen Prediction = **0.6185** (Probable **ANTIGEN**).

>EHO9886715.1 penicillin-insensitive murein endopeptidase [Salmonella enterica subsp. enterica serovar Infantis] Overall Protective Antigen Prediction = **0.3954** (Probable **NON-ANTIGEN**).

>EHO9886716.1 sulfite exporter TauE/SafE family protein [Salmonella enterica subsp. enterica serovar Infantis] Overall Protective Antigen Prediction = **0.4446** (Probable **ANTIGEN**).

>EHO9886717.1 elongation factor P hydroxylase [Salmonella enterica subsp. enterica serovar Infantis] Overall Protective Antigen Prediction = **0.5419** (Probable **ANTIGEN**).

>EHO9886718.1 YfcL family protein [Salmonella enterica subsp. enterica serovar Infantis] Overall Protective Antigen Prediction = **0.3084** (Probable **NON-ANTIGEN**).

>EHO9886719.1 bifunctional tRNA (5-methylaminomethyl-2-thiouridine)(34)-methyltransferase MnmD/FAD-dependent 5-carboxymethylaminomethyl-2-thiouridine(34) oxidoreductase MnmC [Salmonella enterica subsp. enterica serovar Infantis] Overall Protective Antigen Prediction = **0.4677** (Probable **ANTIGEN**).

>EHO9886720.1 beta-ketoacyl-ACP synthase I [Salmonella enterica subsp. enterica serovar Infantis] Overall Protective Antigen Prediction = **0.5539** (Probable **ANTIGEN**).

>EHO9886721.1 CPBP family intramembrane metalloprotease [Salmonella enterica subsp. enterica serovar Infantis] Overall Protective Antigen Prediction = **0.3478** (Probable **NON-ANTIGEN**).

>EHO9886722.1 YbjP/YqhG family protein [Salmonella enterica subsp. enterica serovar Infantis] Overall Protective Antigen Prediction = **0.3380** (Probable **NON-ANTIGEN**).

>EHO9886723.1 C40 family peptidase [Salmonella enterica subsp. enterica serovar Infantis] Overall Protective Antigen Prediction = **0.3998** (Probable **NON-ANTIGEN**).

>EHO9886724.1 transcriptional regulator [Salmonella enterica subsp. enterica serovar Infantis] Overall Protective Antigen Prediction = **0.4984** (Probable **ANTIGEN**).

>EHO9886725.1 hypothetical protein KND05_002074 [Salmonella enterica subsp. enterica serovar Infantis] Overall Protective Antigen Prediction = **0.8165** (Probable **ANTIGEN**).

>EHO9886726.1 MFS transporter [Salmonella enterica subsp. enterica serovar Infantis] Overall Protective Antigen Prediction = **0.5285** (Probable **ANTIGEN**).

>EHO9886727.1 flagella biosynthesis regulator Flk [Salmonella enterica subsp. enterica serovar Infantis] Overall Protective Antigen Prediction = **0.3335** (Probable **NON-ANTIGEN**).

>EHO9886728.1 4-phosphoerythronate dehydrogenase PdxB [Salmonella enterica subsp. enterica serovar Infantis] Overall Protective Antigen Prediction = **0.3885** (Probable **NON-ANTIGEN**).

>EHO9886729.1 aspartate-semialdehyde dehydrogenase [Salmonella enterica subsp. enterica serovar Infantis] Overall Protective Antigen Prediction = **0.4047** (Probable **ANTIGEN**).

>EHO9886730.1 tRNA pseudouridine(38-40) synthase TruA [Salmonella enterica subsp. enterica serovar Infantis] Overall Protective Antigen Prediction = **0.4739** (Probable **ANTIGEN**).

>EHO9886731.1 DedA family protein [Salmonella enterica subsp. enterica serovar Infantis] Overall Protective Antigen Prediction = **0.4509** (Probable **ANTIGEN**).

>EHO9886732.1 acetyl-CoA carboxylase, carboxyltransferase subunit beta [Salmonella enterica subsp. enterica serovar Infantis] Overall Protective Antigen Prediction = **0.4071** (Probable **ANTIGEN**).

>EHO9886733.1 bifunctional tetrahydrofolate synthase/dihydrofolate synthase [Salmonella enterica subsp. enterica serovar Infantis] Overall Protective Antigen Prediction = **0.4130** (Probable **ANTIGEN**).

>EHO9886734.1 cell division protein DedD [Salmonella enterica subsp. enterica serovar Infantis] Overall Protective Antigen Prediction = **0.5314** (Probable **ANTIGEN**).

>EHO9886735.1 colicin V production protein [Salmonella enterica subsp. enterica serovar Infantis] Overall Protective Antigen Prediction = **0.2768** (Probable **NON-ANTIGEN**).

>EHO9886736.1 amidophosphoribosyltransferase [Salmonella enterica subsp. enterica serovar Infantis] Overall Protective Antigen Prediction = **0.4380** (Probable **ANTIGEN**).

>EHO9886737.1 sigma-54-dependent transcriptional regulator [Salmonella enterica subsp. enterica serovar Infantis] Overall Protective Antigen Prediction = **0.3892** (Probable **NON-ANTIGEN**).

>EHO9886738.1 alanine racemase [Salmonella enterica subsp. enterica serovar Infantis] Overall Protective Antigen Prediction = **0.5056** (Probable **ANTIGEN**).

>EHO9886739.1 amino acid permease [Salmonella enterica subsp. enterica serovar Infantis] Overall Protective Antigen Prediction = **0.5267** (Probable **ANTIGEN**).

>EHO9886740.1 alanine/ornithine racemase family PLP-dependent enzyme [Salmonella enterica subsp. enterica serovar Infantis] Overall Protective Antigen Prediction = **0.3379** (Probable **NON-ANTIGEN**).

>EHO9886741.1 amino acid permease [Salmonella enterica subsp. enterica serovar Infantis] Overall Protective Antigen Prediction = **0.5573** (Probable **ANTIGEN**).

>EHO9886742.1 UbiX family flavin prenyltransferase [Salmonella enterica subsp. enterica serovar Infantis] Overall Protective Antigen Prediction = **0.3465** (Probable **NON-ANTIGEN**).

>EHO9886743.1 lysine/arginine/ornithine ABC transporter substrate-binding protein ArgT [Salmonella enterica subsp. enterica serovar Infantis] Overall Protective Antigen Prediction = **0.6271** (Probable **ANTIGEN**).

>EHO9886744.1 histidine ABC transporter substrate-binding protein HisJ [Salmonella enterica subsp. enterica serovar Infantis] Overall Protective Antigen Prediction = **0.6012** (Probable **ANTIGEN**).

>EHO9886745.1 histidine ABC transporter permease HisQ [Salmonella enterica subsp. enterica serovar Infantis] Overall Protective Antigen Prediction = **0.4405** (Probable **ANTIGEN**).

>EHO9886746.1 histidine ABC transporter permease HisM [Salmonella enterica subsp. enterica serovar Infantis] Overall Protective Antigen Prediction = **0.3629** (Probable **NON-ANTIGEN**).

>EHO9886747.1 histidine ABC transporter ATP-binding protein HisP [Salmonella enterica subsp. enterica serovar Infantis] Overall Protective Antigen Prediction = **0.4775** (Probable **ANTIGEN**).

>EHO9886748.1 TIGR01777 family oxidoreductase [Salmonella enterica subsp. enterica serovar Infantis] Overall Protective Antigen Prediction = **0.2977** (Probable **NON-ANTIGEN**).

>EHO9886749.1 GSH-dependent disulfide bond oxidoreductase [Salmonella enterica subsp. enterica serovar Infantis] Overall Protective Antigen Prediction = **0.5411** (Probable **ANTIGEN**).

>EHO9886750.1 glutathione transferase [Salmonella enterica subsp. enterica serovar Infantis] Overall Protective Antigen Prediction = **0.3147** (Probable **NON-ANTIGEN**).

>EHO9886751.1 phosphodiesterase [Salmonella enterica subsp. enterica serovar Infantis] Overall Protective Antigen Prediction = **0.2617** (Probable **NON-ANTIGEN**).

>EHO9886752.1 NUDIX hydrolase YfcD [Salmonella enterica subsp. enterica serovar Infantis] Overall Protective Antigen Prediction = **0.3299** (Probable **NON-ANTIGEN**).

>EHO9886753.1 LacI family DNA-binding transcriptional regulator [Salmonella enterica subsp. enterica serovar Infantis] Overall Protective Antigen Prediction = **0.3685** (Probable **NON-ANTIGEN**).

>EHO9886754.1 PTS sugar transporter subunit IIA [Salmonella enterica subsp. enterica serovar Infantis] Overall Protective Antigen Prediction = **0.2374** (Probable **NON-ANTIGEN**).

>EHO9886755.1 PTS sugar transporter subunit IIB [Salmonella enterica subsp. enterica serovar Infantis] Overall Protective Antigen Prediction = **0.6800** (Probable **ANTIGEN**).

>EHO9886756.1 PTS ascorbate transporter subunit IIC [Salmonella enterica subsp. enterica serovar Infantis] Overall Protective Antigen Prediction = **0.4119** (Probable **ANTIGEN**).

>EHO9886757.1 transketolase [Salmonella enterica subsp. enterica serovar Infantis] Overall Protective Antigen Prediction = **0.4600** (Probable **ANTIGEN**).

>EHO9886758.1 transketolase family protein [Salmonella enterica subsp. enterica serovar Infantis] Overall Protective Antigen Prediction = **0.3744** (Probable **NON-ANTIGEN**).

>EHO9886759.1 putative basic amino acid antiporter YfcC [Salmonella enterica subsp. enterica serovar Infantis] Overall Protective Antigen Prediction = **0.5385** (Probable **ANTIGEN**).

>EHO9886760.1 phosphate acetyltransferase [Salmonella enterica subsp. enterica serovar Infantis] Overall Protective Antigen Prediction = **0.5302** (Probable **ANTIGEN**).

>EHO9886761.1 acetate kinase [Salmonella enterica subsp. enterica serovar Infantis] Overall Protective Antigen Prediction = **0.5473** (Probable **ANTIGEN**).

>EHO9886762.1 DUF412 domain-containing protein [Salmonella enterica subsp. enterica serovar Infantis] Overall Protective Antigen Prediction = **0.3847** (Probable **NON-ANTIGEN**).

>EHO9886763.1 YfbU family protein [Salmonella enterica subsp. enterica serovar Infantis] Overall Protective Antigen Prediction = **0.3400** (Probable **NON-ANTIGEN**).

>EHO9886764.1 sugar phosphatase [Salmonella enterica subsp. enterica serovar Infantis] Overall Protective Antigen Prediction = **0.3353** (Probable **NON-ANTIGEN**).

>EHO9886765.1 SLC13 family permease [Salmonella enterica subsp. enterica serovar Infantis] Overall Protective Antigen Prediction = **0.5062** (Probable **ANTIGEN**).

>EHO9886766.1 5'-deoxynucleotidase [Salmonella enterica subsp. enterica serovar Infantis] Overall Protective Antigen Prediction = **0.4496** (Probable **ANTIGEN**).

>EHO9886767.1 alanine transaminase AlaA [Salmonella enterica subsp. enterica serovar Infantis] Overall Protective Antigen Prediction = **0.2682** (Probable **NON-ANTIGEN**).

>EHO9886768.1 transcriptional regulator LrhA [Salmonella enterica subsp. enterica serovar Infantis] Overall Protective Antigen Prediction = **0.4476** (Probable **ANTIGEN**).

>EHO9886769.1 hypothetical protein KND05_002118 [Salmonella enterica subsp. enterica serovar Infantis] Overall Protective Antigen Prediction = **0.4682** (Probable **ANTIGEN**).

>EHO9886770.1 NADH-quinone oxidoreductase subunit NuoA [Salmonella enterica subsp. enterica serovar Infantis] Overall Protective Antigen Prediction = **0.7710** (Probable **ANTIGEN**).

>EHO9886771.1 NADH-quinone oxidoreductase subunit NuoB [Salmonella enterica subsp. enterica serovar Infantis] Overall Protective Antigen Prediction = **0.4458** (Probable **ANTIGEN**).

>EHO9886772.1 NADH-quinone oxidoreductase subunit C/D [Salmonella enterica subsp. enterica serovar Infantis] Overall Protective Antigen Prediction = **0.3895** (Probable **NON-ANTIGEN**).

>EHO9886773.1 NADH-quinone oxidoreductase subunit NuoE [Salmonella enterica subsp. enterica serovar Infantis] Overall Protective Antigen Prediction = **0.4226** (Probable **ANTIGEN**).

>EHO9886774.1 NADH-quinone oxidoreductase subunit NuoF [Salmonella enterica subsp. enterica serovar Infantis] Overall Protective Antigen Prediction = **0.4535** (Probable **ANTIGEN**).

>EHO9886775.1 NADH-quinone oxidoreductase subunit NuoG [Salmonella enterica subsp. enterica serovar Infantis] Overall Protective Antigen Prediction = **0.6096** (Probable **ANTIGEN**).

>EHO9886776.1 NADH-quinone oxidoreductase subunit NuoH [Salmonella enterica subsp. enterica serovar Infantis] Overall Protective Antigen Prediction = **0.4433** (Probable **ANTIGEN**).

>EHO9886777.1 NADH-quinone oxidoreductase subunit NuoI [Salmonella enterica subsp. enterica serovar Infantis] Overall Protective Antigen Prediction = **0.5807** (Probable **ANTIGEN**).

>EHO9886778.1 NADH-quinone oxidoreductase subunit J [Salmonella enterica subsp. enterica serovar Infantis] Overall Protective Antigen Prediction = **0.6475** (Probable **ANTIGEN**).

>EHO9886779.1 NADH-quinone oxidoreductase subunit NuoK [Salmonella enterica subsp. enterica serovar Infantis] Overall Protective Antigen Prediction = **0.6878** (Probable **ANTIGEN**).

>EHO9886780.1 NADH-quinone oxidoreductase subunit L [Salmonella enterica subsp. enterica serovar Infantis] Overall Protective Antigen Prediction = **0.3792** (Probable **NON-ANTIGEN**).

>EHO9886781.1 NADH-quinone oxidoreductase subunit M [Salmonella enterica subsp. enterica serovar Infantis] Overall Protective Antigen Prediction = **0.5897** (Probable **ANTIGEN**).

>EHO9886782.1 NADH-quinone oxidoreductase subunit NuoN [Salmonella enterica subsp. enterica serovar Infantis] Overall Protective Antigen Prediction = **0.4439** (Probable **ANTIGEN**).

>EHO9886783.1 DUF1877 family protein [Salmonella enterica subsp. enterica serovar Infantis] Overall Protective Antigen Prediction = **0.2244** (Probable **NON-ANTIGEN**).

>EHO9886784.1 VWA domain-containing protein [Salmonella enterica subsp. enterica serovar Infantis] Overall Protective Antigen Prediction = **0.6186** (Probable **ANTIGEN**).

>EHO9886785.1 chemotaxis protein CheV [Salmonella enterica subsp. enterica serovar Infantis] Overall Protective Antigen Prediction = **0.5740** (Probable **ANTIGEN**).

>EHO9886786.1 ribonuclease BN [Salmonella enterica subsp. enterica serovar Infantis] Overall Protective Antigen Prediction = **0.5293** (Probable **ANTIGEN**).

>EHO9886787.1 GNAT family N-acetyltransferase [Salmonella enterica subsp. enterica serovar Infantis] Overall Protective Antigen Prediction = **0.3968** (Probable **NON-ANTIGEN**).

>EHO9886788.1 stress response protein ElaB [Salmonella enterica subsp. enterica serovar Infantis] Overall Protective Antigen Prediction = **0.4350** (Probable **ANTIGEN**).

>EHO9886789.1 isochorismate synthase MenF [Salmonella enterica subsp. enterica serovar Infantis] Overall Protective Antigen Prediction = **0.4426** (Probable **ANTIGEN**).

>EHO9886790.1 2-succinyl-5-enolpyruvyl-6-hydroxy-3-cyclohexene-1-carboxylic-acid synthase [Salmonella enterica subsp. enterica serovar Infantis] Overall Protective Antigen Prediction = **0.2710** (Probable **NON-ANTIGEN**).

>EHO9886791.1 2-succinyl-6-hydroxy-2,4-cyclohexadiene-1-carboxylate synthase [Salmonella enterica subsp. enterica serovar Infantis] Overall Protective Antigen Prediction = **0.5222** (Probable **ANTIGEN**).

>EHO9886792.1 1,4-dihydroxy-2-naphthoyl-CoA synthase [Salmonella enterica subsp. enterica serovar Infantis] Overall Protective Antigen Prediction = **0.5148** (Probable **ANTIGEN**).

>EHO9886793.1 o-succinylbenzoate synthase [Salmonella enterica subsp. enterica serovar Infantis] Overall Protective Antigen Prediction = **0.4367** (Probable **ANTIGEN**).

>EHO9886794.1 o-succinylbenzoate--CoA ligase [Salmonella enterica subsp. enterica serovar Infantis] Overall Protective Antigen Prediction = **0.5444** (Probable **ANTIGEN**).

>EHO9886795.1 signal transduction protein PmrD [Salmonella enterica subsp. enterica serovar

Infantis] Overall Protective Antigen Prediction = **0.2840** (Probable **NON-ANTIGEN**).

>EHO9886796.1 4-amino-4-deoxy-L-arabinose-phosphoundecaprenol flippase subunit ArnF [Salmonella enterica subsp. enterica serovar Infantis] Overall Protective Antigen Prediction = **0.3090** (Probable **NON-ANTIGEN**).

>EHO9886797.1 4-amino-4-deoxy-L-arabinose-phosphoundecaprenol flippase subunit ArnE [Salmonella enterica subsp. enterica serovar Infantis] Overall Protective Antigen Prediction = **0.5336** (Probable **ANTIGEN**).

>EHO9886798.1 lipid IV(A) 4-amino-4-deoxy-L-arabinosyltransferase [Salmonella enterica subsp. enterica serovar Infantis] Overall Protective Antigen Prediction = **0.4382** (Probable **ANTIGEN**).

>EHO9886799.1 4-deoxy-4-formamido-L-arabinose-phosphoundecaprenol deformylase [Salmonella enterica subsp. enterica serovar Infantis] Overall Protective Antigen Prediction = **0.4694** (Probable **ANTIGEN**).

>EHO9886800.1 bifunctional UDP-4-amino-4-deoxy-L-arabinose formyltransferase/UDP-glucuronic acid oxidase ArnA [Salmonella enterica subsp. enterica serovar Infantis] Overall Protective Antigen Prediction = **0.4349** (Probable **ANTIGEN**).

>EHO9886801.1 undecaprenyl-phosphate 4-deoxy-4-formamido-L-arabinose transferase [Salmonella enterica subsp. enterica serovar Infantis] Overall Protective Antigen Prediction = **0.2990** (Probable **NON-ANTIGEN**).

>EHO9886802.1 UDP-4-amino-4-deoxy-L-arabinose aminotransferase [Salmonella enterica subsp. enterica serovar Infantis] Overall Protective Antigen Prediction = **0.5457** (Probable **ANTIGEN**).

>EHO9886803.1 lipopolysaccharide core heptose(II)-phosphate phosphatase [Salmonella enterica subsp. enterica serovar Infantis] Overall Protective Antigen Prediction = **0.3110** (Probable **NON-ANTIGEN**).

>EHO9886804.1 nucleoside triphosphatase NudI [Salmonella enterica subsp. enterica serovar Infantis] Overall Protective Antigen Prediction = **0.5270** (Probable **ANTIGEN**).

>EHO9886805.1 YfaZ family protein [Salmonella enterica subsp. enterica serovar Infantis] Overall Protective Antigen Prediction = **0.8143** (Probable **ANTIGEN**).

>EHO9886806.1 nicotinamide mononucleotide deamidase-related protein YfaY [Salmonella enterica subsp. enterica serovar Infantis] Overall Protective Antigen Prediction = **0.4023** (Probable **ANTIGEN**).

>EHO9886807.1 IclR family transcriptional regulator [Salmonella enterica subsp. enterica serovar Infantis] Overall Protective Antigen Prediction = **0.1756** (Probable **NON-ANTIGEN**).

>EHO9886808.1 L-rhamnonate dehydratase [Salmonella enterica subsp. enterica serovar Infantis] Overall Protective Antigen Prediction = **0.5214** (Probable **ANTIGEN**).

>EHO9886809.1 MFS transporter [Salmonella enterica subsp. enterica serovar Infantis] Overall Protective Antigen Prediction = **0.4192** (Probable **ANTIGEN**).

>EHO9886810.1 2-keto-3-deoxy-L-rhamnonate aldolase [Salmonella enterica subsp. enterica serovar Infantis] Overall Protective Antigen Prediction = **0.3051** (Probable **NON-ANTIGEN**).

>EHO9886811.1 molybdopterin-binding protein [Salmonella enterica subsp. enterica serovar Infantis] Overall Protective Antigen Prediction = **0.5289** (Probable **ANTIGEN**).

>EHO9886812.1 SPI-2 type III secretion system effector deubiquitinase SseL [Salmonella enterica subsp. enterica serovar Infantis] Overall Protective Antigen Prediction = **0.4024** (Probable **ANTIGEN**).

>EHO9886813.1 anaerobic glycerol-3-phosphate dehydrogenase subunit C [Salmonella enterica subsp. enterica serovar Infantis] Overall Protective Antigen Prediction = **0.4998** (Probable **ANTIGEN**).

>EHO9886814.1 glycerol-3-phosphate dehydrogenase subunit GlpB [Salmonella enterica subsp. enterica serovar Infantis] Overall Protective Antigen Prediction = **0.5355** (Probable **ANTIGEN**).

>EHO9886815.1 anaerobic glycerol-3-phosphate dehydrogenase subunit A [Salmonella enterica subsp. enterica serovar Infantis] Overall Protective Antigen Prediction = **0.4218** (Probable **ANTIGEN**).

>EHO9886816.1 glycerol-3-phosphate transporter [Salmonella enterica subsp. enterica serovar Infantis] Overall Protective Antigen Prediction = **0.4465** (Probable **ANTIGEN**).

>EHO9886817.1 glycerophosphodiester phosphodiesterase [Salmonella enterica subsp. enterica serovar Infantis] Overall Protective Antigen Prediction = **0.6344** (Probable **ANTIGEN**).

>EHO9886818.1 LysR family transcriptional regulator [Salmonella enterica subsp. enterica serovar Infantis] Overall Protective Antigen Prediction = **0.3574** (Probable **NON-ANTIGEN**).

>EHO9886819.1 MFS transporter [Salmonella enterica subsp. enterica serovar Infantis] Overall Protective Antigen Prediction = **0.5733** (Probable **ANTIGEN**).

>EHO9886820.1 ferredoxin-like diferric-tyrosyl radical cofactor maintenance protein YfaE [Salmonella enterica subsp. enterica serovar Infantis] Overall Protective Antigen Prediction = **0.4161** (Probable **ANTIGEN**).

>EHO9886821.1 ribonucleotide-diphosphate reductase subunit beta [Salmonella enterica subsp. enterica serovar Infantis] Overall Protective Antigen Prediction = **0.4267** (Probable **ANTIGEN**).

>EHO9886822.1 ribonucleoside-diphosphate reductase subunit alpha [Salmonella enterica subsp. enterica serovar Infantis] Overall Protective Antigen Prediction = **0.4495** (Probable **ANTIGEN**).

>EHO9886823.1 bifunctional 3-demethylubiquinone 3-O-methyltransferase/2-octaprenyl-6-hydroxy phenol methylase [Salmonella enterica subsp. enterica serovar Infantis] Overall Protective Antigen Prediction = **0.4764** (Probable **ANTIGEN**).

>EHO9886824.1 GntR family transcriptional regulator [Salmonella enterica subsp. enterica serovar Infantis] Overall Protective Antigen Prediction = **0.3687** (Probable **NON-ANTIGEN**).

>EHO9886825.1 MFS transporter [Salmonella enterica subsp. enterica serovar Infantis] Overall Protective Antigen Prediction = **0.4764** (Probable **ANTIGEN**).

>EHO9886826.1 mandelate racemase/muconate lactonizing enzyme family protein [Salmonella enterica subsp. enterica serovar Infantis] Overall Protective Antigen Prediction = **0.5384** (Probable **ANTIGEN**).

>EHO9886827.1 DNA topoisomerase (ATP-hydrolyzing) subunit A [Salmonella enterica subsp. enterica serovar Infantis] Overall Protective Antigen Prediction = **0.4414** (Probable **ANTIGEN**).

>EHO9886828.1 two-component system sensor histidine kinase RcsC [Salmonella enterica subsp. enterica serovar Infantis] Overall Protective Antigen Prediction = **0.4814** (Probable **ANTIGEN**).

>EHO9886829.1 transcriptional regulator RcsB [Salmonella enterica subsp. enterica serovar Infantis] Overall Protective Antigen Prediction = **0.2934** (Probable **NON-ANTIGEN**).

>EHO9886830.1 phosphotransferase RcsD [Salmonella enterica subsp. enterica serovar Infantis] Overall Protective Antigen Prediction = **0.5306** (Probable **ANTIGEN**).

>EHO9886831.1 porin OmpC [Salmonella enterica subsp. enterica serovar Infantis] Overall Protective Antigen Prediction = **0.7860** (Probable **ANTIGEN**).

>EHO9886832.1 FAD:protein FMN transferase ApbE [Salmonella enterica subsp. enterica serovar Infantis] Overall Protective Antigen Prediction = **0.5677** (Probable **ANTIGEN**).

>EHO9886833.1 bifunctional DNA-binding transcriptional regulator/O6-methylguanine-DNA methyltransferase Ada [Salmonella enterica subsp. enterica serovar Infantis] Overall Protective Antigen Prediction = **0.4031** (Probable **ANTIGEN**).

>EHO9886834.1 DNA oxidative demethylase AlkB [Salmonella enterica subsp. enterica serovar Infantis] Overall Protective Antigen Prediction = **0.3422** (Probable **NON-ANTIGEN**).

>EHO9886835.1 multidrug ABC transporter permease/ATP-binding protein [Salmonella enterica subsp. enterica serovar Infantis] Overall Protective Antigen Prediction = **0.3543** (Probable **NON-ANTIGEN**).

>EHO9886836.1 serine protease inhibitor ecotin [Salmonella enterica subsp. enterica serovar Infantis] Overall Protective Antigen Prediction = **0.5870** (Probable **ANTIGEN**).

>EHO9886837.1 ferredoxin-type protein NapF [Salmonella enterica subsp. enterica serovar Infantis] Overall Protective Antigen Prediction = **0.5021** (Probable **ANTIGEN**).

>EHO9886838.1 chaperone NapD [Salmonella enterica subsp. enterica serovar Infantis] Overall Protective Antigen Prediction = **0.5903** (Probable **ANTIGEN**).

>EHO9886839.1 nitrate reductase catalytic subunit NapA [Salmonella enterica subsp. enterica serovar Infantis] Overall Protective Antigen Prediction = **0.4137** (Probable **ANTIGEN**).

>EHO9886840.1 ferredoxin-type protein NapG [Salmonella enterica subsp. enterica serovar Infantis] Overall Protective Antigen Prediction = **0.7294** (Probable **ANTIGEN**).

>EHO9886841.1 quinol dehydrogenase ferredoxin subunit NapH [Salmonella enterica subsp. enterica serovar Infantis] Overall Protective Antigen Prediction = **0.3759** (Probable **NON-ANTIGEN**).

>EHO9886842.1 nitrate reductase cytochrome c-type subunit [Salmonella enterica subsp. enterica serovar Infantis] Overall Protective Antigen Prediction = **0.6602** (Probable **ANTIGEN**).

>EHO9886843.1 cytochrome c-type protein NapC [Salmonella enterica subsp. enterica serovar Infantis] Overall Protective Antigen Prediction = **0.4816** (Probable **ANTIGEN**).

>EHO9886844.1 heme ABC exporter ATP-binding protein CcmA, partial [Salmonella enterica subsp.

enterica serovar Infantis] Overall Protective Antigen Prediction = **0.4029** (Probable **ANTIGEN**).

>EHO9886845.1 type I toxin-antitoxin system toxin Ldr family protein [Salmonella enterica subsp. enterica serovar Infantis] Overall Protective Antigen Prediction = **0.0794** (Probable **NON-ANTIGEN**).

>EHO9886846.1 HNH nuclease family protein [Salmonella enterica subsp. enterica serovar Infantis] Overall Protective Antigen Prediction = **0.6270** (Probable **ANTIGEN**).

>EHO9886847.1 YafY family transcriptional regulator [Salmonella enterica subsp. enterica serovar Infantis] Overall Protective Antigen Prediction = **0.5038** (Probable **ANTIGEN**).

>EHO9886848.1 VOC family protein [Salmonella enterica subsp. enterica serovar Infantis] Overall Protective Antigen Prediction = **0.2799** (Probable **NON-ANTIGEN**).

>EHO9886849.1 protein translocase subunit SecF [Salmonella enterica subsp. enterica serovar Infantis] Overall Protective Antigen Prediction = **0.7144** (Probable **ANTIGEN**).

>EHO9886850.1 protein translocase subunit SecD [Salmonella enterica subsp. enterica serovar Infantis] Overall Protective Antigen Prediction = **0.6058** (Probable **ANTIGEN**).

>EHO9886851.1 preprotein translocase subunit YajC [Salmonella enterica subsp. enterica serovar Infantis] Overall Protective Antigen Prediction = **0.5885** (Probable **ANTIGEN**).

>EHO9886852.1 tRNA guanosine(34) transglycosylase Tgt [Salmonella enterica subsp. enterica serovar Infantis] Overall Protective Antigen Prediction = **0.3420** (Probable **NON-ANTIGEN**).

>EHO9886853.1 tRNA preQ1(34) S-adenosylmethionine ribosyltransferase-isomerase QueA [Salmonella enterica subsp. enterica serovar Infantis] Overall Protective Antigen Prediction = **0.4647** (Probable **ANTIGEN**).

>EHO9886854.1 ACP phosphodiesterase [Salmonella enterica subsp. enterica serovar Infantis] Overall Protective Antigen Prediction = **0.3575** (Probable **NON-ANTIGEN**).

>EHO9886855.1 peroxiredoxin [Salmonella enterica subsp. enterica serovar Infantis] Overall Protective Antigen Prediction = **0.5131** (Probable **ANTIGEN**).

>EHO9886856.1 maltodextrin glucosidase [Salmonella enterica subsp. enterica serovar Infantis] Overall Protective Antigen Prediction = **0.5420** (Probable **ANTIGEN**).

>EHO9886857.1 proline-specific permease ProY [Salmonella enterica subsp. enterica serovar Infantis] Overall Protective Antigen Prediction = **0.5614** (Probable **ANTIGEN**).

>EHO9886858.1 branched-chain amino acid transporter carrier protein BrnQ [Salmonella enterica subsp. enterica serovar Infantis] Overall Protective Antigen Prediction = **0.4198** (Probable **ANTIGEN**).

>EHO9886859.1 phosphate regulon sensor histidine kinase PhoR [Salmonella enterica subsp. enterica serovar Infantis] Overall Protective Antigen Prediction = **0.4953** (Probable **ANTIGEN**).

>EHO9886860.1 phosphate response regulator transcription factor PhoB [Salmonella enterica subsp. enterica serovar Infantis] Overall Protective Antigen Prediction = **0.5691** (Probable **ANTIGEN**).

>EHO9886861.1 exonuclease subunit SbcD [Salmonella enterica subsp. enterica serovar Infantis]

Overall Protective Antigen Prediction = **0.4043** (Probable **ANTIGEN**).

>EHO9886862.1 exonuclease subunit SbcC [Salmonella enterica subsp. enterica serovar Infantis] Overall Protective Antigen Prediction = **0.5090** (Probable **ANTIGEN**).

>EHO9886863.1 MFS transporter AraJ [Salmonella enterica subsp. enterica serovar Infantis] Overall Protective Antigen Prediction = **0.4685** (Probable **ANTIGEN**).

>EHO9886864.1 fructokinase [Salmonella enterica subsp. enterica serovar Infantis] Overall Protective Antigen Prediction = **0.7072** (Probable **ANTIGEN**).

>EHO9886865.1 recombination-associated protein RdgC [Salmonella enterica subsp. enterica serovar Infantis] Overall Protective Antigen Prediction = **0.5050** (Probable **ANTIGEN**).

>EHO9886866.1 pyrimidine/purine nucleoside phosphorylase [Salmonella enterica subsp. enterica serovar Infantis] Overall Protective Antigen Prediction = **0.5333** (Probable **ANTIGEN**).

>EHO9886867.1 AroM family protein [Salmonella enterica subsp. enterica serovar Infantis] Overall Protective Antigen Prediction = **0.2833** (Probable **NON-ANTIGEN**).

>EHO9886868.1 protein YaiA [Salmonella enterica subsp. enterica serovar Infantis] Overall Protective Antigen Prediction = **0.6529** (Probable **ANTIGEN**).

>EHO9886869.1 shikimate kinase AroL [Salmonella enterica subsp. enterica serovar Infantis] Overall Protective Antigen Prediction = **0.3873** (Probable **NON-ANTIGEN**).

>EHO9886870.1 YaiI/YqxD family protein [Salmonella enterica subsp. enterica serovar Infantis] Overall Protective Antigen Prediction = **0.5083** (Probable **ANTIGEN**).

>EHO9886871.1 pyrroline-5-carboxylate reductase [Salmonella enterica subsp. enterica serovar Infantis] Overall Protective Antigen Prediction = **0.3116** (Probable **NON-ANTIGEN**).

>EHO9886872.1 diguanylate cyclase AdrA [Salmonella enterica subsp. enterica serovar Infantis] Overall Protective Antigen Prediction = **0.5806** (Probable **ANTIGEN**).

>EHO9886873.1 phosphate starvation-inducible protein PsiF [Salmonella enterica subsp. enterica serovar Infantis] Overall Protective Antigen Prediction = **0.6704** (Probable **ANTIGEN**).

>EHO9886874.1 anti-adapter protein IraP [Salmonella enterica subsp. enterica serovar Infantis] Overall Protective Antigen Prediction = **0.1892** (Probable **NON-ANTIGEN**).

>EHO9886875.1 multidrug efflux MFS transporter [Salmonella enterica subsp. enterica serovar Infantis] Overall Protective Antigen Prediction = **0.4641** (Probable **ANTIGEN**).

>EHO9886876.1 extensin family protein [Salmonella enterica subsp. enterica serovar Infantis] Overall Protective Antigen Prediction = **0.4038** (Probable **ANTIGEN**).

>EHO9886877.1 D-alanine--D-alanine ligase [Salmonella enterica subsp. enterica serovar Infantis] Overall Protective Antigen Prediction = **0.4656** (Probable **ANTIGEN**).

>EHO9886878.1 DUF2754 family protein [Salmonella enterica subsp. enterica serovar Infantis] Overall Protective Antigen Prediction = **0.7672** (Probable **ANTIGEN**).

>EHO9886879.1 YaiY family protein [Salmonella enterica subsp. enterica serovar Infantis] Overall

Protective Antigen Prediction = **0.6284** (Probable **ANTIGEN**).

>EHO9886880.1 surface-exposed outer membrane lipoprotein YaiW [Salmonella enterica subsp. enterica serovar Infantis] Overall Protective Antigen Prediction = **0.4803** (Probable **ANTIGEN**).

>EHO9886881.1 peptide antibiotic transporter SbmA [Salmonella enterica subsp. enterica serovar Infantis] Overall Protective Antigen Prediction = **0.5345** (Probable **ANTIGEN**).

>EHO9886882.1 D-alanyl-D-alanine-carboxypeptidase/endopeptidase AmpH [Salmonella enterica subsp. enterica serovar Infantis] Overall Protective Antigen Prediction = **0.3608** (Probable **NON-ANTIGEN**).

>EHO9886883.1 hydrogen peroxide resistance inhibitor IprA [Salmonella enterica subsp. enterica serovar Infantis] Overall Protective Antigen Prediction = **0.1826** (Probable **NON-ANTIGEN**).

>EHO9886884.1 autotransporter outer membrane beta-barrel domain-containing protein [Salmonella enterica subsp. enterica serovar Infantis] Overall Protective Antigen Prediction = **0.7016** (Probable **ANTIGEN**).

>EHO9886885.1 porphobilinogen synthase [Salmonella enterica subsp. enterica serovar Infantis] Overall Protective Antigen Prediction = **0.3470** (Probable **NON-ANTIGEN**).

>EHO9886886.1 propionate--CoA ligase [Salmonella enterica subsp. enterica serovar Infantis] Overall Protective Antigen Prediction = **0.3353** (Probable **NON-ANTIGEN**).

>EHO9886887.1 bifunctional 2-methylcitrate dehydratase/aconitate hydratase [Salmonella enterica subsp. enterica serovar Infantis] Overall Protective Antigen Prediction = **0.4207** (Probable **ANTIGEN**).

>EHO9886888.1 2-methylcitrate synthase [Salmonella enterica subsp. enterica serovar Infantis] Overall Protective Antigen Prediction = **0.4728** (Probable **ANTIGEN**).

>EHO9886889.1 methylisocitrate lyase [Salmonella enterica subsp. enterica serovar Infantis] Overall Protective Antigen Prediction = **0.3858** (Probable **NON-ANTIGEN**).

>EHO9886890.1 propionate catabolism operon regulatory protein PrpR [Salmonella enterica subsp. enterica serovar Infantis] Overall Protective Antigen Prediction = **0.4621** (Probable **ANTIGEN**).

>EHO9886891.1 DUF1471 domain-containing protein [Salmonella enterica subsp. enterica serovar Infantis] Overall Protective Antigen Prediction = **0.6578** (Probable **ANTIGEN**).

>EHO9886892.1 LysE family transporter [Salmonella enterica subsp. enterica serovar Infantis] Overall Protective Antigen Prediction = **0.3489** (Probable **NON-ANTIGEN**).

>EHO9886893.1 ferrioxamine B receptor FoxA [Salmonella enterica subsp. enterica serovar Infantis] Overall Protective Antigen Prediction = **0.5888** (Probable **ANTIGEN**).

>EHO9886894.1 helix-turn-helix transcriptional regulator [Salmonella enterica subsp. enterica serovar Infantis] Overall Protective Antigen Prediction = **0.4522** (Probable **ANTIGEN**).

>EHO9886895.1 DUF2474 domain-containing protein [Salmonella enterica subsp. enterica serovar Infantis] Overall Protective Antigen Prediction = **0.3125** (Probable **NON-ANTIGEN**).

>EHO9886896.1 cytochrome d ubiquinol oxidase subunit II [Salmonella enterica subsp. enterica

serovar Infantis] Overall Protective Antigen Prediction = **0.6779** (Probable **ANTIGEN**).

>EHO9886897.1 cytochrome ubiquinol oxidase subunit I [Salmonella enterica subsp. enterica serovar Infantis] Overall Protective Antigen Prediction = **0.5139** (Probable **ANTIGEN**).

>EHO9886898.1 hypothetical protein KND05_002247 [Salmonella enterica subsp. enterica serovar Infantis] Overall Protective Antigen Prediction = **0.7790** (Probable **ANTIGEN**).

>EHO9886899.1 type III restriction-modification system endonuclease [Salmonella enterica subsp. enterica serovar Infantis] Overall Protective Antigen Prediction = **0.5020** (Probable **ANTIGEN**).

>EHO9886900.1 site-specific DNA-methyltransferase [Salmonella enterica subsp. enterica serovar Infantis] Overall Protective Antigen Prediction = **0.4701** (Probable **ANTIGEN**).

>EHO9886901.1 MFS transporter [Salmonella enterica subsp. enterica serovar Infantis] Overall Protective Antigen Prediction = **0.5669** (Probable **ANTIGEN**).

>EHO9886902.1 gold resistance metallochaperone GolB [Salmonella enterica subsp. enterica serovar Infantis] Overall Protective Antigen Prediction = **0.7121** (Probable **ANTIGEN**).

>EHO9886903.1 Au(I) sensor transcriptional regulator GolS [Salmonella enterica subsp. enterica serovar Infantis] Overall Protective Antigen Prediction = **0.2785** (Probable **NON-ANTIGEN**).

>EHO9886904.1 gold/copper-translocating P-type ATPase GolT [Salmonella enterica subsp. enterica serovar Infantis] Overall Protective Antigen Prediction = **0.6003** (Probable **ANTIGEN**).

>EHO9886905.1 multidrug efflux RND transporter periplasmic adaptor subunit MdsA [Salmonella enterica subsp. enterica serovar Infantis] Overall Protective Antigen Prediction = **0.3934** (Probable **NON-ANTIGEN**).

>EHO9886906.1 multidrug efflux RND transporter permease subunit MdsB [Salmonella enterica subsp. enterica serovar Infantis] Overall Protective Antigen Prediction = **0.4879** (Probable **ANTIGEN**).

>EHO9886907.1 multidrug efflux transporter outer membrane subunit MdsC [Salmonella enterica subsp. enterica serovar Infantis] Overall Protective Antigen Prediction = **0.5855** (Probable **ANTIGEN**).

>EHO9886908.1 DUF4156 domain-containing protein [Salmonella enterica subsp. enterica serovar Infantis] Overall Protective Antigen Prediction = **0.6996** (Probable **ANTIGEN**).

>EHO9886909.1 hypothetical protein KND05_002258 [Salmonella enterica subsp. enterica serovar Infantis] Overall Protective Antigen Prediction = **0.5989** (Probable **ANTIGEN**).

>EHO9886910.1 helix-turn-helix transcriptional regulator [Salmonella enterica subsp. enterica serovar Infantis] Overall Protective Antigen Prediction = **0.2397** (Probable **NON-ANTIGEN**).

>EHO9886911.1 Ail/Lom family outer membrane beta-barrel protein [Salmonella enterica subsp. enterica serovar Infantis] Overall Protective Antigen Prediction = **0.5781** (Probable **ANTIGEN**).

>EHO9886912.1 hypothetical protein KND05_002261 [Salmonella enterica subsp. enterica serovar Infantis] Overall Protective Antigen Prediction = **0.4948** (Probable **ANTIGEN**).

>EHO9886913.1 response regulator [Salmonella enterica subsp. enterica serovar Infantis] Overall

Protective Antigen Prediction = **0.4510** (Probable **ANTIGEN**).

>EHO9886914.1 EAL domain-containing protein [Salmonella enterica subsp. enterica serovar Infantis] Overall Protective Antigen Prediction = **0.4137** (Probable **ANTIGEN**).

>EHO9886915.1 hypothetical protein KND05_002265 [Salmonella enterica subsp. enterica serovar Infantis] Overall Protective Antigen Prediction = **0.3178** (Probable **NON-ANTIGEN**).

>EHO9886916.1 type 1 fimbrial protein [Salmonella enterica subsp. enterica serovar Infantis] Overall Protective Antigen Prediction = **0.8444** (Probable **ANTIGEN**).

>EHO9886917.1 fimbrial chaperone [Salmonella enterica subsp. enterica serovar Infantis] Overall Protective Antigen Prediction = **0.5652** (Probable **ANTIGEN**).

>EHO9886918.1 fimbrial outer membrane usher protein [Salmonella enterica subsp. enterica serovar Infantis] Overall Protective Antigen Prediction = **0.5567** (Probable **ANTIGEN**).

>EHO9886919.1 fimbrial usher protein StbD [Salmonella enterica subsp. enterica serovar Infantis] Overall Protective Antigen Prediction = **0.4329** (Probable **ANTIGEN**).

>EHO9886920.1 fimbrial assembly chaperone [Salmonella enterica subsp. enterica serovar Infantis] Overall Protective Antigen Prediction = **0.3239** (Probable **NON-ANTIGEN**).

>EHO9886921.1 hypothetical protein KND05_002271 [Salmonella enterica subsp. enterica serovar Infantis] Overall Protective Antigen Prediction = **0.6558** (Probable **ANTIGEN**).

>EHO9886922.1 cytoplasmic protein [Salmonella enterica subsp. enterica serovar Infantis] Overall Protective Antigen Prediction = **0.5160** (Probable **ANTIGEN**).

>EHO9886923.1 LysR family transcriptional regulator [Salmonella enterica subsp. enterica serovar Infantis] Overall Protective Antigen Prediction = **0.4938** (Probable **ANTIGEN**).

>EHO9886924.1 alpha/beta hydrolase [Salmonella enterica subsp. enterica serovar Infantis] Overall Protective Antigen Prediction = **0.6171** (Probable **ANTIGEN**).

>EHO9886925.1 fumarylacetoacetate hydrolase family protein [Salmonella enterica subsp. enterica serovar Infantis] Overall Protective Antigen Prediction = **0.3537** (Probable **NON-ANTIGEN**).

>EHO9886926.1 3-isopropylmalate dehydratase small subunit [Salmonella enterica subsp. enterica serovar Infantis] Overall Protective Antigen Prediction = **0.4056** (Probable **ANTIGEN**).

>EHO9886927.1 3-isopropylmalate dehydratase large subunit [Salmonella enterica subsp. enterica serovar Infantis] Overall Protective Antigen Prediction = **0.4782** (Probable **ANTIGEN**).

>EHO9886928.1 MFS transporter [Salmonella enterica subsp. enterica serovar Infantis] Overall Protective Antigen Prediction = **0.5289** (Probable **ANTIGEN**).

>EHO9886929.1 DUF1889 family protein [Salmonella enterica subsp. enterica serovar Infantis] Overall Protective Antigen Prediction = **0.2276** (Probable **NON-ANTIGEN**).

>EHO9886930.1 DUF4102 domain-containing protein [Salmonella enterica subsp. enterica serovar Infantis] Overall Protective Antigen Prediction = **0.8747** (Probable **ANTIGEN**).

>EHO9886931.1 glutamate-5-semialdehyde dehydrogenase [Salmonella enterica subsp. enterica

serovar Infantis] Overall Protective Antigen Prediction = **0.4568** (Probable **ANTIGEN**).

>EHO9886932.1 glutamate 5-kinase [Salmonella enterica subsp. enterica serovar Infantis] Overall Protective Antigen Prediction = **0.3716** (Probable **NON-ANTIGEN**).

>EHO9886933.1 phosphoporin PhoE [Salmonella enterica subsp. enterica serovar Infantis] Overall Protective Antigen Prediction = **0.7681** (Probable **ANTIGEN**).

>EHO9886934.1 sigma factor-binding protein Crl [Salmonella enterica subsp. enterica serovar Infantis] Overall Protective Antigen Prediction = **0.4670** (Probable **ANTIGEN**).

>EHO9886935.1 esterase FrsA [Salmonella enterica subsp. enterica serovar Infantis] Overall Protective Antigen Prediction = **0.4758** (Probable **ANTIGEN**).

>EHO9886936.1 xanthine phosphoribosyltransferase [Salmonella enterica subsp. enterica serovar Infantis] Overall Protective Antigen Prediction = **0.4003** (Probable **ANTIGEN**).

>EHO9886937.1 cytosol nonspecific dipeptidase [Salmonella enterica subsp. enterica serovar Infantis] Overall Protective Antigen Prediction = **0.4784** (Probable **ANTIGEN**).

>EHO9886938.1 peptide chain release factor H [Salmonella enterica subsp. enterica serovar Infantis] Overall Protective Antigen Prediction = **0.6578** (Probable **ANTIGEN**).

>EHO9886939.1 RNA ligase RtcB family protein [Salmonella enterica subsp. enterica serovar Infantis] Overall Protective Antigen Prediction = **0.2530** (Probable **NON-ANTIGEN**).

>EHO9886940.1 DNA polymerase IV [Salmonella enterica subsp. enterica serovar Infantis] Overall Protective Antigen Prediction = **0.4399** (Probable **ANTIGEN**).

>EHO9886941.1 murein L,D-transpeptidase [Salmonella enterica subsp. enterica serovar Infantis] Overall Protective Antigen Prediction = **0.4856** (Probable **ANTIGEN**).

>EHO9886942.1 class II glutamine amidotransferase [Salmonella enterica subsp. enterica serovar Infantis] Overall Protective Antigen Prediction = **0.4242** (Probable **ANTIGEN**).

>EHO9886943.1 D-sedoheptulose 7-phosphate isomerase [Salmonella enterica subsp. enterica serovar Infantis] Overall Protective Antigen Prediction = **0.5417** (Probable **ANTIGEN**).

>EHO9886944.1 acyl-CoA dehydrogenase FadE [Salmonella enterica subsp. enterica serovar Infantis] Overall Protective Antigen Prediction = **0.4180** (Probable **ANTIGEN**).

>EHO9886945.1 amidohydrolase [Salmonella enterica subsp. enterica serovar Infantis] Overall Protective Antigen Prediction = **0.5346** (Probable **ANTIGEN**).

>EHO9886946.1 Spy/CpxP family protein refolding chaperone [Salmonella enterica subsp. enterica serovar Infantis] Overall Protective Antigen Prediction = **0.2990** (Probable **NON-ANTIGEN**).

>EHO9886947.1 adhesin/invasin protein PagN [Salmonella enterica subsp. enterica serovar Infantis] Overall Protective Antigen Prediction = **0.6630** (Probable **ANTIGEN**).

>EHO9886948.1 hypothetical protein KND05_002301 [Salmonella enterica subsp. enterica serovar Infantis] Overall Protective Antigen Prediction = **0.0780** (Probable **NON-ANTIGEN**).

>EHO9886949.1 TioA protein [Salmonella enterica subsp. enterica serovar Infantis] Overall

Protective Antigen Prediction = **0.3578** (Probable **NON-ANTIGEN**).

>EHO9886950.1 fimbrial protein TcfD [Salmonella enterica subsp. enterica serovar Infantis] Overall Protective Antigen Prediction = **0.6387** (Probable **ANTIGEN**).

>EHO9886951.1 fimbrial outer membrane usher protein TcfC [Salmonella enterica subsp. enterica serovar Infantis] Overall Protective Antigen Prediction = **0.5749** (Probable **ANTIGEN**).

>EHO9886952.1 fimbrial protein TcfB [Salmonella enterica subsp. enterica serovar Infantis] Overall Protective Antigen Prediction = **0.5749** (Probable **ANTIGEN**).

>EHO9886953.1 fimbrial chaperone TcfA [Salmonella enterica subsp. enterica serovar Infantis] Overall Protective Antigen Prediction = **0.6298** (Probable **ANTIGEN**).

>EHO9886954.1 cytoplasmic protein [Salmonella enterica subsp. enterica serovar Infantis] Overall Protective Antigen Prediction = **0.6797** (Probable **ANTIGEN**).

>EHO9886955.1 LysR family transcriptional regulator [Salmonella enterica subsp. enterica serovar Infantis] Overall Protective Antigen Prediction = **0.4201** (Probable **ANTIGEN**).

>EHO9886956.1 polysaccharide deacetylase family protein [Salmonella enterica subsp. enterica serovar Infantis] Overall Protective Antigen Prediction = **0.2784** (Probable **NON-ANTIGEN**).

>EHO9886957.1 pilin structural protein SafD [Salmonella enterica subsp. enterica serovar Infantis] Overall Protective Antigen Prediction = **0.5029** (Probable **ANTIGEN**).

>EHO9886958.1 fimbrial biogenesis outer membrane usher protein [Salmonella enterica subsp. enterica serovar Infantis] Overall Protective Antigen Prediction = **0.6228** (Probable **ANTIGEN**).

>EHO9886959.1 pili assembly chaperone PapD [Salmonella enterica subsp. enterica serovar Infantis] Overall Protective Antigen Prediction = **0.7839** (Probable **ANTIGEN**).

>EHO9886960.1 Saf-pilin pilus formation protein SafA [Salmonella enterica subsp. enterica serovar Infantis] Overall Protective Antigen Prediction = **0.6559** (Probable **ANTIGEN**).

>EHO9886961.1 type I toxin-antitoxin system SymE family toxin [Salmonella enterica subsp. enterica serovar Infantis] Overall Protective Antigen Prediction = **0.3149** (Probable **NON-ANTIGEN**).

>EHO9886962.1 hypothetical protein KND05_002321 [Salmonella enterica subsp. enterica serovar Infantis] Overall Protective Antigen Prediction = **0.4687** (Probable **ANTIGEN**).

>EHO9886963.1 sugar tyrosine-protein kinase [Salmonella enterica subsp. enterica serovar Infantis] Overall Protective Antigen Prediction = **0.6671** (Probable **ANTIGEN**).

>EHO9886964.1 hypothetical protein KND05_002323 [Salmonella enterica subsp. enterica serovar Infantis] Overall Protective Antigen Prediction = **0.4651** (Probable **ANTIGEN**).

>EHO9886965.1 SymE family type I addiction module toxin [Salmonella enterica subsp. enterica serovar Infantis] Overall Protective Antigen Prediction = **0.6998** (Probable **ANTIGEN**).

>EHO9886966.1 hypothetical protein KND05_002325 [Salmonella enterica subsp. enterica serovar Infantis] Overall Protective Antigen Prediction = **0.3876** (Probable **NON-ANTIGEN**).

>EHO9886967.1 RHS domain-containing protein [Salmonella enterica subsp. enterica serovar Infantis] Overall Protective Antigen Prediction = **0.6239** (Probable **ANTIGEN**).

>EHO9886968.1 DcrB-related protein [Salmonella enterica subsp. enterica serovar Infantis] Overall Protective Antigen Prediction = **0.5894** (Probable **ANTIGEN**).

>EHO9886969.1 type VI secretion system tip protein VgrG [Salmonella enterica subsp. enterica serovar Infantis] Overall Protective Antigen Prediction = **0.5675** (Probable **ANTIGEN**).

>EHO9886970.1 DUF2778 domain-containing protein [Salmonella enterica subsp. enterica serovar Infantis] Overall Protective Antigen Prediction = **0.6994** (Probable **ANTIGEN**).

>EHO9886971.1 DUF2195 family protein [Salmonella enterica subsp. enterica serovar Infantis] Overall Protective Antigen Prediction = **0.5988** (Probable **ANTIGEN**).

>EHO9886972.1 hypothetical protein KND05_002331 [Salmonella enterica subsp. enterica serovar Infantis] Overall Protective Antigen Prediction = **0.5626** (Probable **ANTIGEN**).

>EHO9886973.1 glucosaminidase domain-containing protein [Salmonella enterica subsp. enterica serovar Infantis] Overall Protective Antigen Prediction = **0.5096** (Probable **ANTIGEN**).

>EHO9886974.1 DUF2094 domain-containing protein [Salmonella enterica subsp. enterica serovar Infantis] Overall Protective Antigen Prediction = **0.3111** (Probable **NON-ANTIGEN**).

>EHO9886975.1 type VI secretion system membrane subunit TssM [Salmonella enterica subsp. enterica serovar Infantis] Overall Protective Antigen Prediction = **0.5007** (Probable **ANTIGEN**).

>EHO9886976.1 Shiga toxin A subunit [Salmonella enterica subsp. enterica serovar Infantis] Overall Protective Antigen Prediction = **0.2289** (Probable **NON-ANTIGEN**).

>EHO9886977.1 hypothetical protein KND05_002336 [Salmonella enterica subsp. enterica serovar Infantis] Overall Protective Antigen Prediction = **0.5695** (Probable **ANTIGEN**).

>EHO9886978.1 type VI secretion system protein TssL, long form [Salmonella enterica subsp. enterica serovar Infantis] Overall Protective Antigen Prediction = **0.5033** (Probable **ANTIGEN**).

>EHO9886979.1 type VI secretion system baseplate subunit TssK [Salmonella enterica subsp. enterica serovar Infantis] Overall Protective Antigen Prediction = **0.3637** (Probable **NON-ANTIGEN**).

>EHO9886980.1 type VI secretion system lipoprotein TssJ, partial [Salmonella enterica subsp. enterica serovar Infantis] Overall Protective Antigen Prediction = **0.5223** (Probable **ANTIGEN**).

>EHO9886981.1 nickel-responsive transcriptional regulator NikR [Salmonella enterica subsp. enterica serovar Infantis] Overall Protective Antigen Prediction = **0.4565** (Probable **ANTIGEN**).

>EHO9886982.1 4'-phosphopantetheinyl transferase AcpT [Salmonella enterica subsp. enterica serovar Infantis] Overall Protective Antigen Prediction = **0.4229** (Probable **ANTIGEN**).

>EHO9886983.1 AI-2E family transporter [Salmonella enterica subsp. enterica serovar Infantis] Overall Protective Antigen Prediction = **0.4958** (Probable **ANTIGEN**).

>EHO9886984.1 MFS transporter [Salmonella enterica subsp. enterica serovar Infantis] Overall Protective Antigen Prediction = **0.3909** (Probable **NON-ANTIGEN**).

>EHO9886985.1 DcrB family lipoprotein [Salmonella enterica subsp. enterica serovar Infantis] Overall Protective Antigen Prediction = **0.6414** (Probable **ANTIGEN**).

>EHO9886986.1 sulfurtransferase TusA [Salmonella enterica subsp. enterica serovar Infantis] Overall Protective Antigen Prediction = **0.4556** (Probable **ANTIGEN**).

>EHO9886987.1 methyl-accepting chemotaxis citrate transducer [Salmonella enterica subsp. enterica serovar Infantis] Overall Protective Antigen Prediction = **0.5339** (Probable **ANTIGEN**).

>EHO9886988.1 Zn(II)/Cd(II)/Pb(II) translocating P-type ATPase ZntA [Salmonella enterica subsp. enterica serovar Infantis] Overall Protective Antigen Prediction = **0.3950** (Probable **NON-ANTIGEN**).

>EHO9886989.1 lysoplasmalogenase [Salmonella enterica subsp. enterica serovar Infantis] Overall Protective Antigen Prediction = **0.5191** (Probable **ANTIGEN**).

>EHO9886990.1 DUF2500 domain-containing protein [Salmonella enterica subsp. enterica serovar Infantis] Overall Protective Antigen Prediction = **0.6800** (Probable **ANTIGEN**).

>EHO9886991.1 DUF1145 family protein [Salmonella enterica subsp. enterica serovar Infantis] Overall Protective Antigen Prediction = **0.7438** (Probable **ANTIGEN**).

>EHO9886992.1 16S rRNA (guanine(966)-N(2))-methyltransferase [Salmonella enterica subsp. enterica serovar Infantis] Overall Protective Antigen Prediction = **0.4476** (Probable **ANTIGEN**).

>EHO9886993.1 signal recognition particle-docking protein FtsY [Salmonella enterica subsp. enterica serovar Infantis] Overall Protective Antigen Prediction = **0.4065** (Probable **ANTIGEN**).

>EHO9886994.1 cell division ATP-binding protein FtsE [Salmonella enterica subsp. enterica serovar Infantis] Overall Protective Antigen Prediction = **0.3089** (Probable **NON-ANTIGEN**).

>EHO9886995.1 cell division protein FtsX [Salmonella enterica subsp. enterica serovar Infantis] Overall Protective Antigen Prediction = **0.4821** (Probable **ANTIGEN**).

>EHO9886996.1 RNA polymerase sigma factor RpoH [Salmonella enterica subsp. enterica serovar Infantis] Overall Protective Antigen Prediction = **0.4343** (Probable **ANTIGEN**).

>EHO9886997.1 branched chain amino acid ABC transporter substrate-binding protein LivJ [Salmonella enterica subsp. enterica serovar Infantis] Overall Protective Antigen Prediction = **0.4466** (Probable **ANTIGEN**).

>EHO9886998.1 hypothetical protein KND05_002358 [Salmonella enterica subsp. enterica serovar Infantis] Overall Protective Antigen Prediction = **0.8253** (Probable **ANTIGEN**).

>EHO9886999.1 aspartate 1-decarboxylase autocleavage activator PanM [Salmonella enterica subsp. enterica serovar Infantis] Overall Protective Antigen Prediction = **0.4415** (Probable **ANTIGEN**).

>EHO9887000.1 high-affinity branched-chain amino acid ABC transporter substrate-binding protein LivK [Salmonella enterica subsp. enterica serovar Infantis] Overall Protective Antigen Prediction = **0.3978** (Probable **NON-ANTIGEN**).

>EHO9887001.1 high-affinity branched-chain amino acid ABC transporter permease LivH [Salmonella enterica subsp. enterica serovar Infantis] Overall Protective Antigen Prediction = **0.2711** (Probable **NON-ANTIGEN**).

>EHO9887002.1 branched chain amino acid ABC transporter permease LivM [Salmonella enterica subsp. enterica serovar Infantis] Overall Protective Antigen Prediction = **0.5099** (Probable **ANTIGEN**).

>EHO9887003.1 high-affinity branched-chain amino acid ABC transporter ATP-binding protein LivG [Salmonella enterica subsp. enterica serovar Infantis] Overall Protective Antigen Prediction = **0.2584** (Probable **NON-ANTIGEN**).

>EHO9887004.1 high-affinity branched-chain amino acid ABC transporter ATP-binding protein LivF [Salmonella enterica subsp. enterica serovar Infantis] Overall Protective Antigen Prediction = **0.2130** (Probable **NON-ANTIGEN**).

>EHO9887005.1 type II toxin-antitoxin system Phd/YefM family antitoxin [Salmonella enterica subsp. enterica serovar Infantis] Overall Protective Antigen Prediction = **0.3373** (Probable **NON-ANTIGEN**).

>EHO9887006.1 type II toxin-antitoxin system death-on-curing family toxin [Salmonella enterica subsp. enterica serovar Infantis] Overall Protective Antigen Prediction = **0.5225** (Probable **ANTIGEN**).

>EHO9887007.1 sn-glycerol-3-phosphate ABC transporter substrate-binding protein UgpB [Salmonella enterica subsp. enterica serovar Infantis] Overall Protective Antigen Prediction = **0.3780** (Probable **NON-ANTIGEN**).

>EHO9887008.1 sn-glycerol-3-phosphate ABC transporter permease UgpA [Salmonella enterica subsp. enterica serovar Infantis] Overall Protective Antigen Prediction = **0.5822** (Probable **ANTIGEN**).

>EHO9887009.1 sn-glycerol-3-phosphate ABC transporter permease UgpE [Salmonella enterica subsp. enterica serovar Infantis] Overall Protective Antigen Prediction = **0.6726** (Probable **ANTIGEN**).

>EHO9887010.1 sn-glycerol-3-phosphate import ATP-binding protein UgpC [Salmonella enterica subsp. enterica serovar Infantis] Overall Protective Antigen Prediction = **0.5370** (Probable **ANTIGEN**).

>EHO9887011.1 glycerophosphodiester phosphodiesterase [Salmonella enterica subsp. enterica serovar Infantis] Overall Protective Antigen Prediction = **0.5410** (Probable **ANTIGEN**).

>EHO9887012.1 DUF2756 family protein [Salmonella enterica subsp. enterica serovar Infantis] Overall Protective Antigen Prediction = **0.6294** (Probable **ANTIGEN**).

>EHO9887013.1 gamma-glutamyltransferase [Salmonella enterica subsp. enterica serovar Infantis] Overall Protective Antigen Prediction = **0.5048** (Probable **ANTIGEN**).

>EHO9887014.1 phosphotriesterase [Salmonella enterica subsp. enterica serovar Infantis] Overall Protective Antigen Prediction = **0.4691** (Probable **ANTIGEN**).

>EHO9887015.1 DMT family transporter [Salmonella enterica subsp. enterica serovar Infantis] Overall Protective Antigen Prediction = **0.4605** (Probable **ANTIGEN**).

>EHO9887016.1 cytoplasmic protein [Salmonella enterica subsp. enterica serovar Infantis] Overall Protective Antigen Prediction = **0.3484** (Probable **NON-ANTIGEN**).

>EHO9887017.1 DeoR family transcriptional regulator [Salmonella enterica subsp. enterica serovar Infantis] Overall Protective Antigen Prediction = **0.3860** (Probable **NON-ANTIGEN**).

>EHO9887018.1 N-acetyltransferase [Salmonella enterica subsp. enterica serovar Infantis] Overall Protective Antigen Prediction = **0.5464** (Probable **ANTIGEN**).

>EHO9887019.1 oxidoreductase [Salmonella enterica subsp. enterica serovar Infantis] Overall Protective Antigen Prediction = **0.3368** (Probable **NON-ANTIGEN**).

>EHO9887020.1 pirin family protein [Salmonella enterica subsp. enterica serovar Infantis] Overall Protective Antigen Prediction = **0.6713** (Probable **ANTIGEN**).

>EHO9887021.1 gluconate operon transcriptional repressor GntR [Salmonella enterica subsp. enterica serovar Infantis] Overall Protective Antigen Prediction = **0.3323** (Probable **NON-ANTIGEN**).

>EHO9887022.1 gluconokinase [Salmonella enterica subsp. enterica serovar Infantis] Overall Protective Antigen Prediction = **0.3406** (Probable **NON-ANTIGEN**).

>EHO9887023.1 gluconate transporter [Salmonella enterica subsp. enterica serovar Infantis] Overall Protective Antigen Prediction = **0.4550** (Probable **ANTIGEN**).

>EHO9887024.1 aspartate-semialdehyde dehydrogenase [Salmonella enterica subsp. enterica serovar Infantis] Overall Protective Antigen Prediction = **0.3020** (Probable **NON-ANTIGEN**).

>EHO9887025.1 1,4-alpha-glucan branching enzyme [Salmonella enterica subsp. enterica serovar Infantis] Overall Protective Antigen Prediction = **0.4523** (Probable **ANTIGEN**).

>EHO9887026.1 glycogen debranching protein GlgX [Salmonella enterica subsp. enterica serovar Infantis] Overall Protective Antigen Prediction = **0.5641** (Probable **ANTIGEN**).

>EHO9887027.1 glucose-1-phosphate adenylyltransferase [Salmonella enterica subsp. enterica serovar Infantis] Overall Protective Antigen Prediction = **0.3100** (Probable **NON-ANTIGEN**).

>EHO9887028.1 glycogen synthase GlgA [Salmonella enterica subsp. enterica serovar Infantis] Overall Protective Antigen Prediction = **0.3880** (Probable **NON-ANTIGEN**).

>EHO9887029.1 glycogen phosphorylase [Salmonella enterica subsp. enterica serovar Infantis] Overall Protective Antigen Prediction = **0.3985** (Probable **NON-ANTIGEN**).

>EHO9887030.1 IclR family transcriptional regulator [Salmonella enterica subsp. enterica serovar Infantis] Overall Protective Antigen Prediction = **0.4102** (Probable **ANTIGEN**).

>EHO9887031.1 dihydrodipicolinate synthase family protein [Salmonella enterica subsp. enterica serovar Infantis] Overall Protective Antigen Prediction = **0.3318** (Probable **NON-ANTIGEN**).

>EHO9887032.1 dihydroxy-acid dehydratase [Salmonella enterica subsp. enterica serovar Infantis] Overall Protective Antigen Prediction = **0.5050** (Probable **ANTIGEN**).

>EHO9887033.1 MFS transporter [Salmonella enterica subsp. enterica serovar Infantis] Overall Protective Antigen Prediction = **0.4704** (Probable **ANTIGEN**).

>EHO9887034.1 glycerol dehydrogenase [Salmonella enterica subsp. enterica serovar Infantis] Overall Protective Antigen Prediction = **0.4429** (Probable **ANTIGEN**).

>EHO9887035.1 hypothetical protein KND05_002397 [Salmonella enterica subsp. enterica serovar Infantis] Overall Protective Antigen Prediction = **0.5314** (Probable **ANTIGEN**).

>EHO9887036.1 glycerol-3-phosphate dehydrogenase [Salmonella enterica subsp. enterica serovar Infantis] Overall Protective Antigen Prediction = **0.4138** (Probable **ANTIGEN**).

>EHO9887037.1 thiosulfate sulfurtransferase GlpE [Salmonella enterica subsp. enterica serovar Infantis] Overall Protective Antigen Prediction = **0.4217** (Probable **ANTIGEN**).

>EHO9887038.1 rhomboid family intramembrane serine protease GlpG [Salmonella enterica subsp. enterica serovar Infantis] Overall Protective Antigen Prediction = **0.5742** (Probable **ANTIGEN**).

>EHO9887039.1 DeoR/GlpR family transcriptional regulator [Salmonella enterica subsp. enterica serovar Infantis] Overall Protective Antigen Prediction = **0.4168** (Probable **ANTIGEN**).

>EHO9887040.1 HTH-type transcriptional regulator MalT [Salmonella enterica subsp. enterica serovar Infantis] Overall Protective Antigen Prediction = **0.3363** (Probable **NON-ANTIGEN**).

>EHO9887041.1 maltodextrin phosphorylase [Salmonella enterica subsp. enterica serovar Infantis] Overall Protective Antigen Prediction = **0.4106** (Probable **ANTIGEN**).

>EHO9887042.1 4-alpha-glucanotransferase [Salmonella enterica subsp. enterica serovar Infantis] Overall Protective Antigen Prediction = **0.4296** (Probable **ANTIGEN**).

>EHO9887043.1 gluconate transporter [Salmonella enterica subsp. enterica serovar Infantis] Overall Protective Antigen Prediction = **0.4038** (Probable **ANTIGEN**).

>EHO9887044.1 Fe-S biogenesis protein NfuA [Salmonella enterica subsp. enterica serovar Infantis] Overall Protective Antigen Prediction = **0.6726** (Probable **ANTIGEN**).

>EHO9887045.1 DNA utilization protein GntX [Salmonella enterica subsp. enterica serovar Infantis] Overall Protective Antigen Prediction = **0.3856** (Probable **NON-ANTIGEN**).

>EHO9887046.1 pimeloyl-ACP methyl ester esterase BioH [Salmonella enterica subsp. enterica serovar Infantis] Overall Protective Antigen Prediction = **0.4326** (Probable **ANTIGEN**).

>EHO9887047.1 Rpn family recombination-promoting nuclease/putative transposase [Salmonella enterica subsp. enterica serovar Infantis] Overall Protective Antigen Prediction = **0.3732** (Probable **NON-ANTIGEN**).

>EHO9887048.1 [Fe-S]-dependent transcriptional repressor FeoC [Salmonella enterica subsp. enterica serovar Infantis] Overall Protective Antigen Prediction = **0.6216** (Probable **ANTIGEN**).

>EHO9887049.1 Fe(2+) transporter permease subunit FeoB [Salmonella enterica subsp. enterica serovar Infantis] Overall Protective Antigen Prediction = **0.4653** (Probable **ANTIGEN**).

>EHO9887050.1 ferrous iron transporter A [Salmonella enterica subsp. enterica serovar Infantis] Overall Protective Antigen Prediction = **0.2785** (Probable **NON-ANTIGEN**).

>EHO9887051.1 RNA-binding transcriptional accessory protein [Salmonella enterica subsp. enterica serovar Infantis] Overall Protective Antigen Prediction = **0.4761** (Probable **ANTIGEN**).

>EHO9887052.1 transcription elongation factor GreB [Salmonella enterica subsp. enterica serovar

Infantis] Overall Protective Antigen Prediction = **0.3694** (Probable **NON-ANTIGEN**).

>EHO9887053.1 two-component system response regulator OmpR [Salmonella enterica subsp. enterica serovar Infantis] Overall Protective Antigen Prediction = **0.3056** (Probable **NON-ANTIGEN**).

>EHO9887054.1 two-component system sensor histidine kinase EnvZ [Salmonella enterica subsp. enterica serovar Infantis] Overall Protective Antigen Prediction = **0.5392** (Probable **ANTIGEN**).

>EHO9887055.1 phosphoenolpyruvate carboxykinase (ATP) [Salmonella enterica subsp. enterica serovar Infantis] Overall Protective Antigen Prediction = **0.4592** (Probable **ANTIGEN**).

>EHO9887056.1 DUF4153 domain-containing protein [Salmonella enterica subsp. enterica serovar Infantis] Overall Protective Antigen Prediction = **0.4534** (Probable **ANTIGEN**).

>EHO9887057.1 Hsp33 family molecular chaperone HslO [Salmonella enterica subsp. enterica serovar Infantis] Overall Protective Antigen Prediction = **0.4906** (Probable **ANTIGEN**).

>EHO9887058.1 ribosome-associated heat shock protein Hsp15 [Salmonella enterica subsp. enterica serovar Infantis] Overall Protective Antigen Prediction = **0.7437** (Probable **ANTIGEN**).

>EHO9887059.1 GMP/IMP nucleotidase [Salmonella enterica subsp. enterica serovar Infantis] Overall Protective Antigen Prediction = **0.4880** (Probable **ANTIGEN**).

>EHO9887060.1 intracellular growth attenuator protein IgaA [Salmonella enterica subsp. enterica serovar Infantis] Overall Protective Antigen Prediction = **0.4829** (Probable **ANTIGEN**).

>EHO9887061.1 ADP compounds hydrolase NudE [Salmonella enterica subsp. enterica serovar Infantis] Overall Protective Antigen Prediction = **0.3189** (Probable **NON-ANTIGEN**).

>EHO9887062.1 peptidoglycan glycosyltransferase/peptidoglycan DD-transpeptidase MrcA [Salmonella enterica subsp. enterica serovar Infantis] Overall Protective Antigen Prediction = **0.4752** (Probable **ANTIGEN**).

>EHO9887063.1 DNA utilization protein HofM [Salmonella enterica subsp. enterica serovar Infantis] Overall Protective Antigen Prediction = **0.4573** (Probable **ANTIGEN**).

>EHO9887064.1 PilN domain-containing protein [Salmonella enterica subsp. enterica serovar Infantis] Overall Protective Antigen Prediction = **0.4144** (Probable **ANTIGEN**).

>EHO9887065.1 hypothetical protein KND05_002427 [Salmonella enterica subsp. enterica serovar Infantis] Overall Protective Antigen Prediction = **0.6330** (Probable **ANTIGEN**).

>EHO9887066.1 DUF2531 family protein [Salmonella enterica subsp. enterica serovar Infantis] Overall Protective Antigen Prediction = **0.6536** (Probable **ANTIGEN**).

>EHO9887067.1 DNA uptake porin HofQ [Salmonella enterica subsp. enterica serovar Infantis] Overall Protective Antigen Prediction = **0.6295** (Probable **ANTIGEN**).

>EHO9887068.1 shikimate kinase AroK [Salmonella enterica subsp. enterica serovar Infantis] Overall Protective Antigen Prediction = **0.6738** (Probable **ANTIGEN**).

>EHO9887069.1 3-dehydroquinate synthase [Salmonella enterica subsp. enterica serovar Infantis] Overall Protective Antigen Prediction = **0.3417** (Probable **NON-ANTIGEN**).

>EHO9887070.1 cell division protein DamX [Salmonella enterica subsp. enterica serovar Infantis] Overall Protective Antigen Prediction = **0.8826** (Probable **ANTIGEN**).

>EHO9887071.1 adenine-specific DNA-methyltransferase [Salmonella enterica subsp. enterica serovar Infantis] Overall Protective Antigen Prediction = **0.1854** (Probable **NON-ANTIGEN**).

>EHO9887072.1 ribulose-phosphate 3-epimerase [Salmonella enterica subsp. enterica serovar Infantis] Overall Protective Antigen Prediction = **0.5704** (Probable **ANTIGEN**).

>EHO9887073.1 phosphoglycolate phosphatase [Salmonella enterica subsp. enterica serovar Infantis] Overall Protective Antigen Prediction = **0.3235** (Probable **NON-ANTIGEN**).

>EHO9887074.1 tryptophan--tRNA ligase [Salmonella enterica subsp. enterica serovar Infantis] Overall Protective Antigen Prediction = **0.2591** (Probable **NON-ANTIGEN**).

>EHO9887075.1 YhfL family protein [Salmonella enterica subsp. enterica serovar Infantis] Overall Protective Antigen Prediction = **0.4630** (Probable **ANTIGEN**).

>EHO9887076.1 autotransporter adhesin BigA, partial [Salmonella enterica subsp. enterica serovar Infantis] Overall Protective Antigen Prediction = **0.8360** (Probable **ANTIGEN**).

>EHO9887077.1 DUF1471 domain-containing protein [Salmonella enterica subsp. enterica serovar Infantis] Overall Protective Antigen Prediction = **0.6378** (Probable **ANTIGEN**).

>EHO9887078.1 DksA/TraR family C4-type zinc finger protein [Salmonella enterica subsp. enterica serovar Infantis] Overall Protective Antigen Prediction = **0.6622** (Probable **ANTIGEN**).

>EHO9887079.1 23S rRNA (adenine(1618)-N(6))-methyltransferase RlmF [Salmonella enterica subsp. enterica serovar Infantis] Overall Protective Antigen Prediction = **0.3837** (Probable **NON-ANTIGEN**).

>EHO9887080.1 mechanosensitive channel protein [Salmonella enterica subsp. enterica serovar Infantis] Overall Protective Antigen Prediction = **0.5145** (Probable **ANTIGEN**).

>EHO9887081.1 glutamine ABC transporter ATP-binding protein GlnQ [Salmonella enterica subsp. enterica serovar Infantis] Overall Protective Antigen Prediction = **0.4830** (Probable **ANTIGEN**).

>EHO9887082.1 glutamine ABC transporter permease GlnP [Salmonella enterica subsp. enterica serovar Infantis] Overall Protective Antigen Prediction = **0.3434** (Probable **NON-ANTIGEN**).

>EHO9887083.1 glutamine ABC transporter substrate-binding protein GlnH [Salmonella enterica subsp. enterica serovar Infantis] Overall Protective Antigen Prediction = **0.6081** (Probable **ANTIGEN**).

>EHO9887084.1 DNA starvation/stationary phase protection protein Dps [Salmonella enterica subsp. enterica serovar Infantis] Overall Protective Antigen Prediction = **0.4378** (Probable **ANTIGEN**).

>EHO9887085.1 threonine/homoserine exporter RhtA [Salmonella enterica subsp. enterica serovar Infantis] Overall Protective Antigen Prediction = **0.6745** (Probable **ANTIGEN**).

>EHO9887086.1 outer membrane protein OmpX [Salmonella enterica subsp. enterica serovar Infantis] Overall Protective Antigen Prediction = **0.6531** (Probable **ANTIGEN**).

>EHO9887087.1 phosphoethanolamine transferase [Salmonella enterica subsp. enterica serovar Infantis] Overall Protective Antigen Prediction = **0.4269** (Probable **ANTIGEN**).

>EHO9887088.1 manganase accumulation protein MntS [Salmonella enterica subsp. enterica serovar Infantis] Overall Protective Antigen Prediction = **0.7019** (Probable **ANTIGEN**).

>EHO9887089.1 manganese-binding transcriptional regulator MntR [Salmonella enterica subsp. enterica serovar Infantis] Overall Protective Antigen Prediction = **0.4644** (Probable **ANTIGEN**).

>EHO9887090.1 anion transporter [Salmonella enterica subsp. enterica serovar Infantis] Overall Protective Antigen Prediction = **0.4406** (Probable **ANTIGEN**).

>EHO9887091.1 L,D-transpeptidase [Salmonella enterica subsp. enterica serovar Infantis] Overall Protective Antigen Prediction = **0.5273** (Probable **ANTIGEN**).

>EHO9887092.1 ABC-F family ATPase [Salmonella enterica subsp. enterica serovar Infantis] Overall Protective Antigen Prediction = **0.4639** (Probable **ANTIGEN**).

>EHO9887093.1 DUF1479 domain-containing protein [Salmonella enterica subsp. enterica serovar Infantis] Overall Protective Antigen Prediction = **0.3824** (Probable **NON-ANTIGEN**).

>EHO9887094.1 HAD family hydrolase [Salmonella enterica subsp. enterica serovar Infantis] Overall Protective Antigen Prediction = **0.2238** (Probable **NON-ANTIGEN**).

>EHO9887095.1 glycyl radical protein [Salmonella enterica subsp. enterica serovar Infantis] Overall Protective Antigen Prediction = **0.4277** (Probable **ANTIGEN**).

>EHO9887096.1 glycyl-radical enzyme activating protein [Salmonella enterica subsp. enterica serovar Infantis] Overall Protective Antigen Prediction = **0.5472** (Probable **ANTIGEN**).

>EHO9887097.1 molybdopterin-synthase adenylyltransferase MoeB [Salmonella enterica subsp. enterica serovar Infantis] Overall Protective Antigen Prediction = **0.4800** (Probable **ANTIGEN**).

>EHO9887098.1 molybdopterin molybdotransferase MoeA [Salmonella enterica subsp. enterica serovar Infantis] Overall Protective Antigen Prediction = **0.4395** (Probable **ANTIGEN**).

>EHO9887099.1 beta-aspartyl-peptidase [Salmonella enterica subsp. enterica serovar Infantis] Overall Protective Antigen Prediction = **0.4533** (Probable **ANTIGEN**).

>EHO9887100.1 glutathione ABC transporter ATP-binding protein GsiA [Salmonella enterica subsp. enterica serovar Infantis] Overall Protective Antigen Prediction = **0.5061** (Probable **ANTIGEN**).

>EHO9887101.1 glutathione ABC transporter substrate-binding protein GsiB [Salmonella enterica subsp. enterica serovar Infantis] Overall Protective Antigen Prediction = **0.4190** (Probable **ANTIGEN**).

>EHO9887102.1 glutathione ABC transporter permease GsiC [Salmonella enterica subsp. enterica serovar Infantis] Overall Protective Antigen Prediction = **0.5982** (Probable **ANTIGEN**).

>EHO9887103.1 glutathione ABC transporter permease GsiD [Salmonella enterica subsp. enterica serovar Infantis] Overall Protective Antigen Prediction = **0.3478** (Probable **NON-ANTIGEN**).

>EHO9887104.1 30S ribosomal protein S12 methylthiotransferase RimO [Salmonella enterica subsp. enterica serovar Infantis] Overall Protective Antigen Prediction = **0.4169** (Probable **ANTIGEN**).

>EHO9887105.1 biofilm formation regulator BssR [Salmonella enterica subsp. enterica serovar Infantis] Overall Protective Antigen Prediction = **0.4075** (Probable **ANTIGEN**).

>EHO9887106.1 CoA ester lyase [Salmonella enterica subsp. enterica serovar Infantis] Overall Protective Antigen Prediction = **0.2241** (Probable **NON-ANTIGEN**).

>EHO9887107.1 electron transfer flavoprotein subunit beta/FixA family protein [Salmonella enterica subsp. enterica serovar Infantis] Overall Protective Antigen Prediction = **0.3395** (Probable **NON-ANTIGEN**).

>EHO9887108.1 FAD-binding protein [Salmonella enterica subsp. enterica serovar Infantis] Overall Protective Antigen Prediction = **0.3415** (Probable **NON-ANTIGEN**).

>EHO9887109.1 hypothetical protein KND05_002471 [Salmonella enterica subsp. enterica serovar Infantis] Overall Protective Antigen Prediction = **0.3656** (Probable **NON-ANTIGEN**).

>EHO9887110.1 acyl-CoA/acyl-ACP dehydrogenase [Salmonella enterica subsp. enterica serovar Infantis] Overall Protective Antigen Prediction = **0.4229** (Probable **ANTIGEN**).

>EHO9887111.1 electron transfer flavoprotein-ubiquinone oxidoreductase [Salmonella enterica subsp. enterica serovar Infantis] Overall Protective Antigen Prediction = **0.4212** (Probable **ANTIGEN**).

>EHO9887112.1 LysR family transcriptional regulator [Salmonella enterica subsp. enterica serovar Infantis] Overall Protective Antigen Prediction = **0.4296** (Probable **ANTIGEN**).

>EHO9887113.1 GntP family permease [Salmonella enterica subsp. enterica serovar Infantis] Overall Protective Antigen Prediction = **0.5299** (Probable **ANTIGEN**).

>EHO9887114.1 glutathione S-transferase family protein [Salmonella enterica subsp. enterica serovar Infantis] Overall Protective Antigen Prediction = **0.2686** (Probable **NON-ANTIGEN**).

>EHO9887115.1 serine-type D-Ala-D-Ala carboxypeptidase [Salmonella enterica subsp. enterica serovar Infantis] Overall Protective Antigen Prediction = **0.4760** (Probable **ANTIGEN**).

>EHO9887116.1 DNA-binding transcriptional repressor DeoR [Salmonella enterica subsp. enterica serovar Infantis] Overall Protective Antigen Prediction = **0.3565** (Probable **NON-ANTIGEN**).

>EHO9887117.1 undecaprenyl-diphosphate phosphatase [Salmonella enterica subsp. enterica serovar Infantis] Overall Protective Antigen Prediction = **0.3878** (Probable **NON-ANTIGEN**).

>EHO9887118.1 MFS transporter [Salmonella enterica subsp. enterica serovar Infantis] Overall Protective Antigen Prediction = **0.4413** (Probable **ANTIGEN**).

>EHO9887119.1 HAD family hydrolase [Salmonella enterica subsp. enterica serovar Infantis] Overall Protective Antigen Prediction = **0.3474** (Probable **NON-ANTIGEN**).

>EHO9887120.1 MFS transporter [Salmonella enterica subsp. enterica serovar Infantis] Overall Protective Antigen Prediction = **0.5499** (Probable **ANTIGEN**).

>EHO9887121.1 TetR/AcrR family transcriptional regulator [Salmonella enterica subsp. enterica serovar Infantis] Overall Protective Antigen Prediction = **0.4354** (Probable **ANTIGEN**).

>EHO9887122.1 aspartate:alanine antiporter [Salmonella enterica subsp. enterica serovar Infantis]

Overall Protective Antigen Prediction = **0.4932** (Probable **ANTIGEN**).

>EHO9887123.1 inner membrane protein YbjM [Salmonella enterica subsp. enterica serovar Infantis] Overall Protective Antigen Prediction = **0.6275** (Probable **ANTIGEN**).

>EHO9887124.1 GrxA family glutaredoxin [Salmonella enterica subsp. enterica serovar Infantis] Overall Protective Antigen Prediction = **0.5367** (Probable **ANTIGEN**).

>EHO9887125.1 YbjC family protein [Salmonella enterica subsp. enterica serovar Infantis] Overall Protective Antigen Prediction = **0.5345** (Probable **ANTIGEN**).

>EHO9887126.1 30S ribosomal protein S6--L-glutamate ligase [Salmonella enterica subsp. enterica serovar Infantis] Overall Protective Antigen Prediction = **0.4077** (Probable **ANTIGEN**).

>EHO9887127.1 YbjN domain-containing protein [Salmonella enterica subsp. enterica serovar Infantis] Overall Protective Antigen Prediction = **0.3614** (Probable **NON-ANTIGEN**).

>EHO9887128.1 spermidine/putrescine ABC transporter substrate-binding protein PotF [Salmonella enterica subsp. enterica serovar Infantis] Overall Protective Antigen Prediction = **0.3792** (Probable **NON-ANTIGEN**).

>EHO9887129.1 putrescine ABC transporter ATP-binding subunit PotG [Salmonella enterica subsp. enterica serovar Infantis] Overall Protective Antigen Prediction = **0.4108** (Probable **ANTIGEN**).

>EHO9887130.1 putrescine ABC transporter permease PotH [Salmonella enterica subsp. enterica serovar Infantis] Overall Protective Antigen Prediction = **0.5930** (Probable **ANTIGEN**).

>EHO9887131.1 putrescine ABC transporter permease PotI [Salmonella enterica subsp. enterica serovar Infantis] Overall Protective Antigen Prediction = **0.4897** (Probable **ANTIGEN**).

>EHO9887132.1 YbjO family protein [Salmonella enterica subsp. enterica serovar Infantis] Overall Protective Antigen Prediction = **0.5092** (Probable **ANTIGEN**).

>EHO9887133.1 23S rRNA (uracil(747)-C(5))-methyltransferase RlmC [Salmonella enterica subsp. enterica serovar Infantis] Overall Protective Antigen Prediction = **0.4645** (Probable **ANTIGEN**).

>EHO9887134.1 PTS sugar transporter subunit IIC [Salmonella enterica subsp. enterica serovar Infantis] Overall Protective Antigen Prediction = **0.3897** (Probable **NON-ANTIGEN**).

>EHO9887135.1 PTS sugar transporter subunit IIB [Salmonella enterica subsp. enterica serovar Infantis] Overall Protective Antigen Prediction = **0.5006** (Probable **ANTIGEN**).

>EHO9887136.1 sulfatase [Salmonella enterica subsp. enterica serovar Infantis] Overall Protective Antigen Prediction = **0.2835** (Probable **NON-ANTIGEN**).

>EHO9887137.1 ABC transporter substrate-binding protein ArtJ [Salmonella enterica subsp. enterica serovar Infantis] Overall Protective Antigen Prediction = **0.5695** (Probable **ANTIGEN**).

>EHO9887138.1 arginine ABC transporter permease ArtM [Salmonella enterica subsp. enterica serovar Infantis] Overall Protective Antigen Prediction = **0.2927** (Probable **NON-ANTIGEN**).

>EHO9887139.1 arginine ABC transporter permease ArtQ [Salmonella enterica subsp. enterica serovar Infantis] Overall Protective Antigen Prediction = **0.5857** (Probable **ANTIGEN**).

>EHO9887140.1 arginine ABC transporter substrate-binding protein [Salmonella enterica subsp. enterica serovar Infantis] Overall Protective Antigen Prediction = **0.5941** (Probable **ANTIGEN**).

>EHO9887141.1 arginine ABC transporter ATP-binding protein ArtP [Salmonella enterica subsp. enterica serovar Infantis] Overall Protective Antigen Prediction = **0.3813** (Probable **NON-ANTIGEN**).

>EHO9887142.1 lipoprotein [Salmonella enterica subsp. enterica serovar Infantis] Overall Protective Antigen Prediction = **0.4906** (Probable **ANTIGEN**).

>EHO9887143.1 heavy metal-binding domain-containing protein [Salmonella enterica subsp. enterica serovar Infantis] Overall Protective Antigen Prediction = **0.6797** (Probable **ANTIGEN**).

>EHO9887144.1 N-acetylmuramoyl-L-alanine amidase [Salmonella enterica subsp. enterica serovar Infantis] Overall Protective Antigen Prediction = **0.5388** (Probable **ANTIGEN**).

>EHO9887145.1 NAD(P)-dependent oxidoreductase [Salmonella enterica subsp. enterica serovar Infantis] Overall Protective Antigen Prediction = **0.3254** (Probable **NON-ANTIGEN**).

>EHO9887146.1 DUF2867 domain-containing protein [Salmonella enterica subsp. enterica serovar Infantis] Overall Protective Antigen Prediction = **0.3919** (Probable **NON-ANTIGEN**).

>EHO9887147.1 low-specificity L-threonine aldolase [Salmonella enterica subsp. enterica serovar Infantis] Overall Protective Antigen Prediction = **0.3074** (Probable **NON-ANTIGEN**).

>EHO9887148.1 ubiquinone-dependent pyruvate dehydrogenase [Salmonella enterica subsp. enterica serovar Infantis] Overall Protective Antigen Prediction = **0.3994** (Probable **NON-ANTIGEN**).

>EHO9887149.1 NADH oxidoreductase [Salmonella enterica subsp. enterica serovar Infantis] Overall Protective Antigen Prediction = **0.4254** (Probable **ANTIGEN**).

>EHO9887150.1 hydroxylamine reductase [Salmonella enterica subsp. enterica serovar Infantis] Overall Protective Antigen Prediction = **0.4175** (Probable **ANTIGEN**).

>EHO9887151.1 lysine exporter LysO family protein [Salmonella enterica subsp. enterica serovar Infantis] Overall Protective Antigen Prediction = **0.5027** (Probable **ANTIGEN**).

>EHO9887152.1 ATP-dependent endonuclease [Salmonella enterica subsp. enterica serovar Infantis] Overall Protective Antigen Prediction = **0.4765** (Probable **ANTIGEN**).

>EHO9887153.1 VirK/YbjX family protein [Salmonella enterica subsp. enterica serovar Infantis] Overall Protective Antigen Prediction = **0.2779** (Probable **NON-ANTIGEN**).

>EHO9887154.1 macrolide transporter subunit MacA [Salmonella enterica subsp. enterica serovar Infantis] Overall Protective Antigen Prediction = **0.5254** (Probable **ANTIGEN**).

>EHO9887155.1 macrolide ABC transporter ATP-binding protein/permease MacB [Salmonella enterica subsp. enterica serovar Infantis] Overall Protective Antigen Prediction = **0.5271** (Probable **ANTIGEN**).

>EHO9887156.1 cold shock-like protein CspD [Salmonella enterica subsp. enterica serovar Infantis] Overall Protective Antigen Prediction = **0.4925** (Probable **ANTIGEN**).

>EHO9887157.1 ATP-dependent Clp protease adapter ClpS [Salmonella enterica subsp. enterica

serovar Infantis] Overall Protective Antigen Prediction = **0.0860** (Probable **NON-ANTIGEN**).

>EHO9887158.1 ATP-dependent Clp protease ATP-binding subunit ClpA [Salmonella enterica subsp. enterica serovar Infantis] Overall Protective Antigen Prediction = **0.4214** (Probable **ANTIGEN**).

>EHO9887159.1 hypothetical protein KND05_002524 [Salmonella enterica subsp. enterica serovar Infantis] Overall Protective Antigen Prediction = **0.0976** (Probable **NON-ANTIGEN**).

>EHO9887160.1 integrase [Salmonella enterica subsp. enterica serovar Infantis] Overall Protective Antigen Prediction = **-0.0730** (Probable **NON-ANTIGEN**).

>EHO9887161.1 hydrolase [Salmonella enterica subsp. enterica serovar Infantis] Overall Protective Antigen Prediction = **0.1908** (Probable **NON-ANTIGEN**).

>EHO9887162.1 pirin family protein [Salmonella enterica subsp. enterica serovar Infantis] Overall Protective Antigen Prediction = **0.4945** (Probable **ANTIGEN**).

>EHO9887163.1 LysR family transcriptional regulator [Salmonella enterica subsp. enterica serovar Infantis] Overall Protective Antigen Prediction = **0.3929** (Probable **NON-ANTIGEN**).

>EHO9887164.1 translation initiation factor IF-1 [Salmonella enterica subsp. enterica serovar Infantis] Overall Protective Antigen Prediction = **0.6077** (Probable **ANTIGEN**).

>EHO9887165.1 hypothetical protein KND05_002531 [Salmonella enterica subsp. enterica serovar Infantis] Overall Protective Antigen Prediction = **0.3193** (Probable **NON-ANTIGEN**).

>EHO9887166.1 leucyl/phenylalanyl-tRNA--protein transferase [Salmonella enterica subsp. enterica serovar Infantis] Overall Protective Antigen Prediction = **0.1510** (Probable **NON-ANTIGEN**).

>EHO9887167.1 cysteine/glutathione ABC transporter ATP-binding protein/permease CydC [Salmonella enterica subsp. enterica serovar Infantis] Overall Protective Antigen Prediction = **0.4068** (Probable **ANTIGEN**).

>EHO9887168.1 cysteine/glutathione ABC transporter permease/ATP-binding protein CydD [Salmonella enterica subsp. enterica serovar Infantis] Overall Protective Antigen Prediction = **0.3214** (Probable **NON-ANTIGEN**).

>EHO9887169.1 thioredoxin-disulfide reductase [Salmonella enterica subsp. enterica serovar Infantis] Overall Protective Antigen Prediction = **0.4856** (Probable **ANTIGEN**).

>EHO9887170.1 leucine-responsive transcriptional regulator Lrp [Salmonella enterica subsp. enterica serovar Infantis] Overall Protective Antigen Prediction = **0.3012** (Probable **NON-ANTIGEN**).

>EHO9887171.1 DNA translocase FtsK [Salmonella enterica subsp. enterica serovar Infantis] Overall Protective Antigen Prediction = **0.5752** (Probable **ANTIGEN**).

>EHO9887172.1 outer membrane lipoprotein chaperone LolA [Salmonella enterica subsp. enterica serovar Infantis] Overall Protective Antigen Prediction = **0.6546** (Probable **ANTIGEN**).

>EHO9887173.1 replication-associated recombination protein RarA [Salmonella enterica subsp. enterica serovar Infantis] Overall Protective Antigen Prediction = **0.3932** (Probable **NON-ANTIGEN**).

>EHO9887174.1 serine--tRNA ligase [Salmonella enterica subsp. enterica serovar Infantis] Overall

Protective Antigen Prediction = **0.5084** (Probable **ANTIGEN**).

>EHO9887175.1 dimethylsulfoxide reductase subunit A [Salmonella enterica subsp. enterica serovar Infantis] Overall Protective Antigen Prediction = **0.5147** (Probable **ANTIGEN**).

>EHO9887176.1 dimethylsulfoxide reductase subunit B, partial [Salmonella enterica subsp. enterica serovar Infantis] Overall Protective Antigen Prediction = **0.4732** (Probable **ANTIGEN**).

>EHO9887177.1 IS110 family transposase, partial [Salmonella enterica subsp. enterica serovar Infantis] Overall Protective Antigen Prediction = **0.5247** (Probable **ANTIGEN**).

>EHO9887178.1 serine-type D-Ala-D-Ala carboxypeptidase [Salmonella enterica subsp. enterica serovar Infantis] Overall Protective Antigen Prediction = **0.3772** (Probable **NON-ANTIGEN**).

>EHO9887179.1 transcription elongation factor GreA [Salmonella enterica subsp. enterica serovar Infantis] Overall Protective Antigen Prediction = **0.3949** (Probable **NON-ANTIGEN**).

>EHO9887180.1 ribosome assembly RNA-binding protein YhbY [Salmonella enterica subsp. enterica serovar Infantis] Overall Protective Antigen Prediction = **0.2991** (Probable **NON-ANTIGEN**).

>EHO9887181.1 23S rRNA (uridine(2552)-2'-O)-methyltransferase RlmE [Salmonella enterica subsp. enterica serovar Infantis] Overall Protective Antigen Prediction = **0.4268** (Probable **ANTIGEN**).

>EHO9887182.1 ATP-dependent zinc metalloprotease FtsH [Salmonella enterica subsp. enterica serovar Infantis] Overall Protective Antigen Prediction = **0.4629** (Probable **ANTIGEN**).

>EHO9887183.1 dihydropteroate synthase [Salmonella enterica subsp. enterica serovar Infantis] Overall Protective Antigen Prediction = **0.4087** (Probable **ANTIGEN**).

>EHO9887184.1 phosphoglucosamine mutase [Salmonella enterica subsp. enterica serovar Infantis] Overall Protective Antigen Prediction = **0.5699** (Probable **ANTIGEN**).

>EHO9887185.1 preprotein translocase subunit SecG [Salmonella enterica subsp. enterica serovar Infantis] Overall Protective Antigen Prediction = **0.5910** (Probable **ANTIGEN**).

>EHO9887186.1 DUF1963 domain-containing protein [Salmonella enterica subsp. enterica serovar Infantis] Overall Protective Antigen Prediction = **0.4549** (Probable **ANTIGEN**).

>EHO9887187.1 argininosuccinate synthase [Salmonella enterica subsp. enterica serovar Infantis] Overall Protective Antigen Prediction = **0.3579** (Probable **NON-ANTIGEN**).

>EHO9887188.1 ribosome maturation factor RimP [Salmonella enterica subsp. enterica serovar Infantis] Overall Protective Antigen Prediction = **0.4805** (Probable **ANTIGEN**).

>EHO9887189.1 transcription termination/antitermination protein NusA [Salmonella enterica subsp. enterica serovar Infantis] Overall Protective Antigen Prediction = **0.4835** (Probable **ANTIGEN**).

>EHO9887190.1 translation initiation factor IF-2 [Salmonella enterica subsp. enterica serovar Infantis] Overall Protective Antigen Prediction = **0.7163** (Probable **ANTIGEN**).

>EHO9887191.1 30S ribosome-binding factor RbfA [Salmonella enterica subsp. enterica serovar Infantis] Overall Protective Antigen Prediction = **0.3392** (Probable **NON-ANTIGEN**).

>EHO9887192.1 tRNA pseudouridine(55) synthase TruB [Salmonella enterica subsp. enterica serovar

Infantis] Overall Protective Antigen Prediction = **0.3581** (Probable **NON-ANTIGEN**).

>EHO9887193.1 30S ribosomal protein S15 [Salmonella enterica subsp. enterica serovar Infantis] Overall Protective Antigen Prediction = **0.3719** (Probable **NON-ANTIGEN**).

>EHO9887194.1 polyribonucleotide nucleotidyltransferase [Salmonella enterica subsp. enterica serovar Infantis] Overall Protective Antigen Prediction = **0.5330** (Probable **ANTIGEN**).

>EHO9887195.1 lipoprotein NlpI [Salmonella enterica subsp. enterica serovar Infantis] Overall Protective Antigen Prediction = **0.3520** (Probable **NON-ANTIGEN**).

>EHO9887196.1 protein YrbN [Salmonella enterica subsp. enterica serovar Infantis] Overall Protective Antigen Prediction = **0.6127** (Probable **ANTIGEN**).

>EHO9887197.1 ATP-dependent RNA helicase DeaD [Salmonella enterica subsp. enterica serovar Infantis] Overall Protective Antigen Prediction = **0.6246** (Probable **ANTIGEN**).

>EHO9887198.1 tryptophan permease [Salmonella enterica subsp. enterica serovar Infantis] Overall Protective Antigen Prediction = **0.4511** (Probable **ANTIGEN**).

>EHO9887199.1 LLM class flavin-dependent oxidoreductase [Salmonella enterica subsp. enterica serovar Infantis] Overall Protective Antigen Prediction = **0.4405** (Probable **ANTIGEN**).

>EHO9887200.1 U32 family peptidase [Salmonella enterica subsp. enterica serovar Infantis] Overall Protective Antigen Prediction = **0.2668** (Probable **NON-ANTIGEN**).

>EHO9887201.1 U32 family peptidase [Salmonella enterica subsp. enterica serovar Infantis] Overall Protective Antigen Prediction = **0.3901** (Probable **NON-ANTIGEN**).

>EHO9887202.1 SCP2 domain-containing protein [Salmonella enterica subsp. enterica serovar Infantis] Overall Protective Antigen Prediction = **0.4751** (Probable **ANTIGEN**).

>EHO9887203.1 N-acetyltransferase [Salmonella enterica subsp. enterica serovar Infantis] Overall Protective Antigen Prediction = **0.4326** (Probable **ANTIGEN**).

>EHO9887204.1 GIY-YIG nuclease family protein [Salmonella enterica subsp. enterica serovar Infantis] Overall Protective Antigen Prediction = **0.7097** (Probable **ANTIGEN**).

>EHO9887205.1 YhbP family protein [Salmonella enterica subsp. enterica serovar Infantis] Overall Protective Antigen Prediction = **0.6235** (Probable **ANTIGEN**).

>EHO9887206.1 protein/nucleic acid deglycase [Salmonella enterica subsp. enterica serovar Infantis] Overall Protective Antigen Prediction = **0.2788** (Probable **NON-ANTIGEN**).

>EHO9887207.1 NAD(P)H-binding protein [Salmonella enterica subsp. enterica serovar Infantis] Overall Protective Antigen Prediction = **0.3771** (Probable **NON-ANTIGEN**).

>EHO9887208.1 divisome-associated lipoprotein YraP [Salmonella enterica subsp. enterica serovar Infantis] Overall Protective Antigen Prediction = **0.6930** (Probable **ANTIGEN**).

>EHO9887209.1 DnaA initiator-associating protein DiaA [Salmonella enterica subsp. enterica serovar Infantis] Overall Protective Antigen Prediction = **0.3455** (Probable **NON-ANTIGEN**).

>EHO9887210.1 YraN family protein [Salmonella enterica subsp. enterica serovar Infantis] Overall

Protective Antigen Prediction = **0.4410** (Probable **ANTIGEN**).

>EHO9887211.1 penicillin-binding protein activator [Salmonella enterica subsp. enterica serovar Infantis] Overall Protective Antigen Prediction = **0.5125** (Probable **ANTIGEN**).

>EHO9887212.1 16S rRNA (cytidine(1402)-2'-O)-methyltransferase [Salmonella enterica subsp. enterica serovar Infantis] Overall Protective Antigen Prediction = **0.3145** (Probable **NON-ANTIGEN**).

>EHO9887213.1 DeoR/GlpR transcriptional regulator [Salmonella enterica subsp. enterica serovar Infantis] Overall Protective Antigen Prediction = **0.4543** (Probable **ANTIGEN**).

>EHO9887214.1 galactitol-1-phosphate 5-dehydrogenase [Salmonella enterica subsp. enterica serovar Infantis] Overall Protective Antigen Prediction = **0.4832** (Probable **ANTIGEN**).

>EHO9887215.1 PTS galactitol transporter subunit IIC [Salmonella enterica subsp. enterica serovar Infantis] Overall Protective Antigen Prediction = **0.3509** (Probable **NON-ANTIGEN**).

>EHO9887216.1 PTS galactitol transporter subunit IIB [Salmonella enterica subsp. enterica serovar Infantis] Overall Protective Antigen Prediction = **0.3203** (Probable **NON-ANTIGEN**).

>EHO9887217.1 PTS galactitol transporter subunit IIA [Salmonella enterica subsp. enterica serovar Infantis] Overall Protective Antigen Prediction = **0.2386** (Probable **NON-ANTIGEN**).

>EHO9887218.1 tagatose-bisphosphate aldolase subunit GatZ [Salmonella enterica subsp. enterica serovar Infantis] Overall Protective Antigen Prediction = **0.3557** (Probable **NON-ANTIGEN**).

>EHO9887219.1 tagatose bisphosphate family class II aldolase [Salmonella enterica subsp. enterica serovar Infantis] Overall Protective Antigen Prediction = **0.3739** (Probable **NON-ANTIGEN**).

>EHO9887220.1 galactarate dehydratase [Salmonella enterica subsp. enterica serovar Infantis] Overall Protective Antigen Prediction = **0.4764** (Probable **ANTIGEN**).

>EHO9887221.1 2-dehydro-3-deoxyglucarate aldolase [Salmonella enterica subsp. enterica serovar Infantis] Overall Protective Antigen Prediction = **0.3303** (Probable **NON-ANTIGEN**).

>EHO9887222.1 2-hydroxy-3-oxopropionate reductase [Salmonella enterica subsp. enterica serovar Infantis] Overall Protective Antigen Prediction = **0.5686** (Probable **ANTIGEN**).

>EHO9887223.1 glycerate 2-kinase [Salmonella enterica subsp. enterica serovar Infantis] Overall Protective Antigen Prediction = **0.5760** (Probable **ANTIGEN**).

>EHO9887224.1 hypothetical protein KND05_002593 [Salmonella enterica subsp. enterica serovar Infantis] Overall Protective Antigen Prediction = **0.4840** (Probable **ANTIGEN**).

>EHO9887225.1 transcriptional regulator TdcA [Salmonella enterica subsp. enterica serovar Infantis] Overall Protective Antigen Prediction = **0.2371** (Probable **NON-ANTIGEN**).

>EHO9887226.1 bifunctional threonine ammonia-lyase/L-serine ammonia-lyase TdcB [Salmonella enterica subsp. enterica serovar Infantis] Overall Protective Antigen Prediction = **0.3928** (Probable **NON-ANTIGEN**).

>EHO9887227.1 threonine/serine transporter TdcC [Salmonella enterica subsp. enterica serovar Infantis] Overall Protective Antigen Prediction = **0.6410** (Probable **ANTIGEN**).

>EHO9887228.1 propionate kinase [Salmonella enterica subsp. enterica serovar Infantis] Overall Protective Antigen Prediction = **0.3883** (Probable **NON-ANTIGEN**).

>EHO9887229.1 formate C-acetyltransferase [Salmonella enterica subsp. enterica serovar Infantis] Overall Protective Antigen Prediction = **0.4773** (Probable **ANTIGEN**).

>EHO9887230.1 L-serine ammonia-lyase [Salmonella enterica subsp. enterica serovar Infantis] Overall Protective Antigen Prediction = **0.5055** (Probable **ANTIGEN**).

>EHO9887231.1 HAAAP family serine/threonine permease [Salmonella enterica subsp. enterica serovar Infantis] Overall Protective Antigen Prediction = **0.5074** (Probable **ANTIGEN**).

>EHO9887232.1 serine dehydratase subunit alpha family protein [Salmonella enterica subsp. enterica serovar Infantis] Overall Protective Antigen Prediction = **0.5344** (Probable **ANTIGEN**).

>EHO9887233.1 hypothetical protein KND05_002602 [Salmonella enterica subsp. enterica serovar Infantis] Overall Protective Antigen Prediction = **0.5181** (Probable **ANTIGEN**).

>EHO9887234.1 pirin family protein [Salmonella enterica subsp. enterica serovar Infantis] Overall Protective Antigen Prediction = **0.6493** (Probable **ANTIGEN**).

>EHO9887235.1 DNA-binding transcriptional regulator YhaJ [Salmonella enterica subsp. enterica serovar Infantis] Overall Protective Antigen Prediction = **0.3678** (Probable **NON-ANTIGEN**).

>EHO9887236.1 DUF805 domain-containing protein [Salmonella enterica subsp. enterica serovar Infantis] Overall Protective Antigen Prediction = **1.1631** (Probable **ANTIGEN**).

>EHO9887237.1 glutathione S-transferase family protein [Salmonella enterica subsp. enterica serovar Infantis] Overall Protective Antigen Prediction = **0.3980** (Probable **NON-ANTIGEN**).

>EHO9887238.1 DoxX family protein [Salmonella enterica subsp. enterica serovar Infantis] Overall Protective Antigen Prediction = **0.2702** (Probable **NON-ANTIGEN**).

>EHO9887239.1 YqjK-like family protein [Salmonella enterica subsp. enterica serovar Infantis] Overall Protective Antigen Prediction = **0.3648** (Probable **NON-ANTIGEN**).

>EHO9887240.1 phage holin family protein [Salmonella enterica subsp. enterica serovar Infantis] Overall Protective Antigen Prediction = **0.4594** (Probable **ANTIGEN**).

>EHO9887241.1 DUF883 domain-containing protein [Salmonella enterica subsp. enterica serovar Infantis] Overall Protective Antigen Prediction = **0.6555** (Probable **ANTIGEN**).

>EHO9887242.1 DUF1090 domain-containing protein [Salmonella enterica subsp. enterica serovar Infantis] Overall Protective Antigen Prediction = **0.8292** (Probable **ANTIGEN**).

>EHO9887243.1 EnvZ/OmpR regulon moderator MzrA [Salmonella enterica subsp. enterica serovar Infantis] Overall Protective Antigen Prediction = **0.5313** (Probable **ANTIGEN**).

>EHO9887244.1 DedA family protein [Salmonella enterica subsp. enterica serovar Infantis] Overall Protective Antigen Prediction = **0.2634** (Probable **NON-ANTIGEN**).

>EHO9887245.1 serine/threonine transporter SstT [Salmonella enterica subsp. enterica serovar Infantis] Overall Protective Antigen Prediction = **0.5022** (Probable **ANTIGEN**).

>EHO9887246.1 TerC family protein [Salmonella enterica subsp. enterica serovar Infantis] Overall Protective Antigen Prediction = **0.5696** (Probable **ANTIGEN**).

>EHO9887247.1 Gfo/Idh/MocA family oxidoreductase [Salmonella enterica subsp. enterica serovar Infantis] Overall Protective Antigen Prediction = **0.1900** (Probable **NON-ANTIGEN**).

>EHO9887248.1 vancomycin high temperature exclusion protein [Salmonella enterica subsp. enterica serovar Infantis] Overall Protective Antigen Prediction = **0.3533** (Probable **NON-ANTIGEN**).

>EHO9887249.1 M48 family metallopeptidase [Salmonella enterica subsp. enterica serovar Infantis] Overall Protective Antigen Prediction = **0.4263** (Probable **ANTIGEN**).

>EHO9887250.1 23S rRNA (guanine(1835)-N(2))-methyltransferase RlmG [Salmonella enterica subsp. enterica serovar Infantis] Overall Protective Antigen Prediction = **0.3387** (Probable **NON-ANTIGEN**).

>EHO9887251.1 NADPH-dependent 2,4-dienoyl-CoA reductase [Salmonella enterica subsp. enterica serovar Infantis] Overall Protective Antigen Prediction = **0.3672** (Probable **NON-ANTIGEN**).

>EHO9887252.1 putrescine aminotransferase [Salmonella enterica subsp. enterica serovar Infantis] Overall Protective Antigen Prediction = **0.3014** (Probable **NON-ANTIGEN**).

>EHO9887253.1 PAS domain-containing protein [Salmonella enterica subsp. enterica serovar Infantis] Overall Protective Antigen Prediction = **0.4764** (Probable **ANTIGEN**).

>EHO9887254.1 MCP four helix bundle domain-containing protein [Salmonella enterica subsp. enterica serovar Infantis] Overall Protective Antigen Prediction = **0.4089** (Probable **ANTIGEN**).

>EHO9887255.1 PadR family transcriptional regulator [Salmonella enterica subsp. enterica serovar Infantis] Overall Protective Antigen Prediction = **0.8725** (Probable **ANTIGEN**).

>EHO9887256.1 siderophore-interacting protein [Salmonella enterica subsp. enterica serovar Infantis] Overall Protective Antigen Prediction = **0.3571** (Probable **NON-ANTIGEN**).

>EHO9887257.1 G/U mismatch-specific DNA glycosylase [Salmonella enterica subsp. enterica serovar Infantis] Overall Protective Antigen Prediction = **0.2892** (Probable **NON-ANTIGEN**).

>EHO9887258.1 RNA polymerase sigma factor RpoD [Salmonella enterica subsp. enterica serovar Infantis] Overall Protective Antigen Prediction = **0.3431** (Probable **NON-ANTIGEN**).

>EHO9887259.1 DNA primase [Salmonella enterica subsp. enterica serovar Infantis] Overall Protective Antigen Prediction = **0.3772** (Probable **NON-ANTIGEN**).

>EHO9887260.1 30S ribosomal protein S21 [Salmonella enterica subsp. enterica serovar Infantis] Overall Protective Antigen Prediction = **0.6071** (Probable **ANTIGEN**).

>EHO9887261.1 tRNA (adenosine(37)-N6)-threonylcarbamoyltransferase complex transferase subunit TsaD [Salmonella enterica subsp. enterica serovar Infantis] Overall Protective Antigen Prediction = **0.5283** (Probable **ANTIGEN**).

>EHO9887262.1 glycerol-3-phosphate 1-O-acyltransferase PlsY [Salmonella enterica subsp. enterica serovar Infantis] Overall Protective Antigen Prediction = **0.6288** (Probable **ANTIGEN**).

>EHO9887263.1 bifunctional dihydroneopterin aldolase/7,8-dihydroneopterin epimerase [Salmonella enterica subsp. enterica serovar Infantis] Overall Protective Antigen Prediction = **0.4275** (Probable **ANTIGEN**).

>EHO9887264.1 undecaprenyl-diphosphate phosphatase [Salmonella enterica subsp. enterica serovar Infantis] Overall Protective Antigen Prediction = **0.3716** (Probable **NON-ANTIGEN**).

>EHO9887265.1 multifunctional CCA addition/repair protein [Salmonella enterica subsp. enterica serovar Infantis] Overall Protective Antigen Prediction = **0.5167** (Probable **ANTIGEN**).

>EHO9887266.1 SH3 domain-containing protein [Salmonella enterica subsp. enterica serovar Infantis] Overall Protective Antigen Prediction = **0.6017** (Probable **ANTIGEN**).

>EHO9887267.1 inorganic triphosphatase [Salmonella enterica subsp. enterica serovar Infantis] Overall Protective Antigen Prediction = **0.5296** (Probable **ANTIGEN**).

>EHO9887268.1 bifunctional [glutamate--ammonia ligase]-adenylyl-L-tyrosine phosphorylase/[glutamate--ammonia-ligase] adenylyltransferase [Salmonella enterica subsp. enterica serovar Infantis] Overall Protective Antigen Prediction = **0.3754** (Probable **NON-ANTIGEN**).

>EHO9887269.1 bifunctional D-glycero-beta-D-manno-heptose-7-phosphate kinase/D-glycero-beta-D-manno-heptose 1-phosphate adenylyltransferase HldE [Salmonella enterica subsp. enterica serovar Infantis] Overall Protective Antigen Prediction = **0.4862** (Probable **ANTIGEN**).

>EHO9887270.1 type I toxin-antitoxin system Ibs family toxin [Salmonella enterica subsp. enterica serovar Infantis] Overall Protective Antigen Prediction = **0.9231** (Probable **ANTIGEN**).

>EHO9887271.1 flotillin family protein [Salmonella enterica subsp. enterica serovar Infantis] Overall Protective Antigen Prediction = **0.7082** (Probable **ANTIGEN**).

>EHO9887272.1 DUF1449 family protein [Salmonella enterica subsp. enterica serovar Infantis] Overall Protective Antigen Prediction = **0.3956** (Probable **NON-ANTIGEN**).

>EHO9887273.1 cell surface composition regulator GlgS [Salmonella enterica subsp. enterica serovar Infantis] Overall Protective Antigen Prediction = **0.4932** (Probable **ANTIGEN**).

>EHO9887274.1 ubiquinone biosynthesis accessory factor UbiK [Salmonella enterica subsp. enterica serovar Infantis] Overall Protective Antigen Prediction = **0.3942** (Probable **NON-ANTIGEN**).

>EHO9887275.1 3,4-dihydroxy-2-butanone-4-phosphate synthase [Salmonella enterica subsp. enterica serovar Infantis] Overall Protective Antigen Prediction = **0.5762** (Probable **ANTIGEN**).

>EHO9887276.1 DUF4385 domain-containing protein [Salmonella enterica subsp. enterica serovar Infantis] Overall Protective Antigen Prediction = **0.4740** (Probable **ANTIGEN**).

>EHO9887277.1 MFS transporter [Salmonella enterica subsp. enterica serovar Infantis] Overall Protective Antigen Prediction = **0.4707** (Probable **ANTIGEN**).

>EHO9887278.1 YfiM family lipoprotein [Salmonella enterica subsp. enterica serovar Infantis] Overall Protective Antigen Prediction = **0.5910** (Probable **ANTIGEN**).

>EHO9887279.1 CDP-diacylglycerol--serine O-phosphatidyltransferase [Salmonella enterica subsp. enterica serovar Infantis] Overall Protective Antigen Prediction = **0.3991** (Probable **NON-ANTIGEN**).

>EHO9887280.1 protein lysine acetyltransferase [Salmonella enterica subsp. enterica serovar Infantis] Overall Protective Antigen Prediction = **0.3730** (Probable **NON-ANTIGEN**).

>EHO9887281.1 tRNA-uridine aminocarboxypropyltransferase [Salmonella enterica subsp. enterica serovar Infantis] Overall Protective Antigen Prediction = **0.3698** (Probable **NON-ANTIGEN**).

>EHO9887282.1 thioredoxin TrxC [Salmonella enterica subsp. enterica serovar Infantis] Overall Protective Antigen Prediction = **0.5844** (Probable **ANTIGEN**).

>EHO9887283.1 tRNA/rRNA methyltransferase [Salmonella enterica subsp. enterica serovar Infantis] Overall Protective Antigen Prediction = **0.6865** (Probable **ANTIGEN**).

>EHO9887284.1 uracil-DNA glycosylase [Salmonella enterica subsp. enterica serovar Infantis] Overall Protective Antigen Prediction = **0.2568** (Probable **NON-ANTIGEN**).

>EHO9887285.1 autonomous glycyl radical cofactor GrcA [Salmonella enterica subsp. enterica serovar Infantis] Overall Protective Antigen Prediction = **0.6418** (Probable **ANTIGEN**).

>EHO9887286.1 cysteine/O-acetylserine transporter [Salmonella enterica subsp. enterica serovar Infantis] Overall Protective Antigen Prediction = **0.4802** (Probable **ANTIGEN**).

>EHO9887287.1 LysR family transcriptional regulator [Salmonella enterica subsp. enterica serovar Infantis] Overall Protective Antigen Prediction = **0.4515** (Probable **ANTIGEN**).

>EHO9887288.1 ATP-dependent RNA helicase SrmB [Salmonella enterica subsp. enterica serovar Infantis] Overall Protective Antigen Prediction = **0.4497** (Probable **ANTIGEN**).

>EHO9887289.1 tRNA(1)(Val) (adenine(37)-N(6))-methyltransferase TrmN [Salmonella enterica subsp. enterica serovar Infantis] Overall Protective Antigen Prediction = **0.6386** (Probable **ANTIGEN**).

>EHO9887290.1 L-aspartate oxidase [Salmonella enterica subsp. enterica serovar Infantis] Overall Protective Antigen Prediction = **0.4331** (Probable **ANTIGEN**).

>EHO9887291.1 rpoE leader peptide RseD [Salmonella enterica subsp. enterica serovar Infantis] Overall Protective Antigen Prediction = **0.8570** (Probable **ANTIGEN**).

>EHO9887292.1 RNA polymerase sigma factor RpoE [Salmonella enterica subsp. enterica serovar Infantis] Overall Protective Antigen Prediction = **0.3692** (Probable **NON-ANTIGEN**).

>EHO9887293.1 anti-sigma-E factor RseA [Salmonella enterica subsp. enterica serovar Infantis] Overall Protective Antigen Prediction = **0.5390** (Probable **ANTIGEN**).

>EHO9887294.1 sigma-E factor regulatory protein RseB [Salmonella enterica subsp. enterica serovar Infantis] Overall Protective Antigen Prediction = **0.5159** (Probable **ANTIGEN**).

>EHO9887295.1 SoxR-reducing system protein RseC [Salmonella enterica subsp. enterica serovar Infantis] Overall Protective Antigen Prediction = **0.5831** (Probable **ANTIGEN**).

>EHO9887296.1 elongation factor 4 [Salmonella enterica subsp. enterica serovar Infantis] Overall Protective Antigen Prediction = **0.4309** (Probable **ANTIGEN**).

>EHO9887297.1 signal peptidase I [Salmonella enterica subsp. enterica serovar Infantis] Overall

Protective Antigen Prediction = **0.4025** (Probable **ANTIGEN**).

>EHO9887298.1 ribonuclease III [Salmonella enterica subsp. enterica serovar Infantis] Overall Protective Antigen Prediction = **0.5958** (Probable **ANTIGEN**).

>EHO9887299.1 GTPase Era [Salmonella enterica subsp. enterica serovar Infantis] Overall Protective Antigen Prediction = **0.3387** (Probable **NON-ANTIGEN**).

>EHO9887300.1 DNA repair protein RecO [Salmonella enterica subsp. enterica serovar Infantis] Overall Protective Antigen Prediction = **0.5067** (Probable **ANTIGEN**).

>EHO9887301.1 pyridoxine 5'-phosphate synthase [Salmonella enterica subsp. enterica serovar Infantis] Overall Protective Antigen Prediction = **0.6616** (Probable **ANTIGEN**).

>EHO9887302.1 holo-ACP synthase [Salmonella enterica subsp. enterica serovar Infantis] Overall Protective Antigen Prediction = **0.2227** (Probable **NON-ANTIGEN**).

>EHO9887303.1 YfhL family 4Fe-4S dicluster ferredoxin [Salmonella enterica subsp. enterica serovar Infantis] Overall Protective Antigen Prediction = **0.2263** (Probable **NON-ANTIGEN**).

>EHO9887304.1 LysR family transcriptional regulator [Salmonella enterica subsp. enterica serovar Infantis] Overall Protective Antigen Prediction = **0.3973** (Probable **NON-ANTIGEN**).

>EHO9887305.1 OFA family MFS transporter [Salmonella enterica subsp. enterica serovar Infantis] Overall Protective Antigen Prediction = **0.4232** (Probable **ANTIGEN**).

>EHO9887306.1 oxidoreductase [Salmonella enterica subsp. enterica serovar Infantis] Overall Protective Antigen Prediction = **0.4336** (Probable **ANTIGEN**).

>EHO9887307.1 MurR/RpiR family transcriptional regulator [Salmonella enterica subsp. enterica serovar Infantis] Overall Protective Antigen Prediction = **0.5159** (Probable **ANTIGEN**).

>EHO9887308.1 N-acetylmuramic acid 6-phosphate etherase [Salmonella enterica subsp. enterica serovar Infantis] Overall Protective Antigen Prediction = **0.5822** (Probable **ANTIGEN**).

>EHO9887309.1 PTS transporter subunit EIIC [Salmonella enterica subsp. enterica serovar Infantis] Overall Protective Antigen Prediction = **0.3352** (Probable **NON-ANTIGEN**).

>EHO9887310.1 phosphatidylglycerophosphatase C [Salmonella enterica subsp. enterica serovar Infantis] Overall Protective Antigen Prediction = **0.3781** (Probable **NON-ANTIGEN**).

>EHO9887311.1 tRNA adenosine(34) deaminase TadA [Salmonella enterica subsp. enterica serovar Infantis] Overall Protective Antigen Prediction = **0.3799** (Probable **NON-ANTIGEN**).

>EHO9887312.1 membrane-bound lytic murein transglycosylase MltF [Salmonella enterica subsp. enterica serovar Infantis] Overall Protective Antigen Prediction = **0.5015** (Probable **ANTIGEN**).

>EHO9887313.1 hypothetical protein KND05_002685 [Salmonella enterica subsp. enterica serovar Infantis] Overall Protective Antigen Prediction = **0.4288** (Probable **ANTIGEN**).

>EHO9887314.1 phosphoribosylformylglycinamidine synthase [Salmonella enterica subsp. enterica serovar Infantis] Overall Protective Antigen Prediction = **0.4878** (Probable **ANTIGEN**).

>EHO9887315.1 two component system sensor histidine kinase QseE/GlrK [Salmonella enterica

subsp. enterica serovar Infantis] Overall Protective Antigen Prediction = **0.3557** (Probable **NON-ANTIGEN**).

>EHO9887316.1 two-component system QseEF-associated lipoprotein QseG [Salmonella enterica subsp. enterica serovar Infantis] Overall Protective Antigen Prediction = **0.5306** (Probable **ANTIGEN**).

>EHO9887317.1 two-component system response regulator GlrR [Salmonella enterica subsp. enterica serovar Infantis] Overall Protective Antigen Prediction = **0.3292** (Probable **NON-ANTIGEN**).

>EHO9887318.1 nitrogen regulatory protein P-II [Salmonella enterica subsp. enterica serovar Infantis] Overall Protective Antigen Prediction = **0.4624** (Probable **ANTIGEN**).

>EHO9887319.1 dipeptide permease DtpD [Salmonella enterica subsp. enterica serovar Infantis] Overall Protective Antigen Prediction = **0.3766** (Probable **NON-ANTIGEN**).

>EHO9887320.1 lysine decarboxylase CadA [Salmonella enterica subsp. enterica serovar Infantis] Overall Protective Antigen Prediction = **0.4428** (Probable **ANTIGEN**).

>EHO9887321.1 cadaverine/lysine antiporter [Salmonella enterica subsp. enterica serovar Infantis] Overall Protective Antigen Prediction = **0.4580** (Probable **ANTIGEN**).

>EHO9887322.1 lysine decarboxylation/transport transcriptional activator CadC [Salmonella enterica subsp. enterica serovar Infantis] Overall Protective Antigen Prediction = **0.4262** (Probable **ANTIGEN**).

>EHO9887323.1 NO-inducible flavohemoprotein [Salmonella enterica subsp. enterica serovar Infantis] Overall Protective Antigen Prediction = **0.4021** (Probable **ANTIGEN**).

>EHO9887324.1 serine hydroxymethyltransferase [Salmonella enterica subsp. enterica serovar Infantis] Overall Protective Antigen Prediction = **0.4485** (Probable **ANTIGEN**).

>EHO9887325.1 3-phenylpropionate MFS transporter [Salmonella enterica subsp. enterica serovar Infantis] Overall Protective Antigen Prediction = **0.4215** (Probable **ANTIGEN**).

>EHO9887326.1 stationary phase inducible protein CsiE [Salmonella enterica subsp. enterica serovar Infantis] Overall Protective Antigen Prediction = **0.4946** (Probable **ANTIGEN**).

>EHO9887327.1 DUF1007 family protein [Salmonella enterica subsp. enterica serovar Infantis] Overall Protective Antigen Prediction = **0.4894** (Probable **ANTIGEN**).

>EHO9887328.1 nickel/cobalt transporter [Salmonella enterica subsp. enterica serovar Infantis] Overall Protective Antigen Prediction = **0.4909** (Probable **ANTIGEN**).

>EHO9887329.1 sulfite reductase subunit C [Salmonella enterica subsp. enterica serovar Infantis] Overall Protective Antigen Prediction = **0.5701** (Probable **ANTIGEN**).

>EHO9887330.1 anaerobic sulfite reductase subunit B [Salmonella enterica subsp. enterica serovar Infantis] Overall Protective Antigen Prediction = **0.3577** (Probable **NON-ANTIGEN**).

>EHO9887331.1 anaerobic sulfite reductase subunit A [Salmonella enterica subsp. enterica serovar Infantis] Overall Protective Antigen Prediction = **0.4948** (Probable **ANTIGEN**).

>EHO9887332.1 alpha/beta hydrolase [Salmonella enterica subsp. enterica serovar Infantis] Overall

Protective Antigen Prediction = **0.4953** (Probable **ANTIGEN**).

>EHO9887333.1 inositol-1-monophosphatase [Salmonella enterica subsp. enterica serovar Infantis] Overall Protective Antigen Prediction = **0.5601** (Probable **ANTIGEN**).

>EHO9887334.1 tRNA (cytosine(32)/uridine(32)-2'-O)-methyltransferase TrmJ [Salmonella enterica subsp. enterica serovar Infantis] Overall Protective Antigen Prediction = **0.4063** (Probable **ANTIGEN**).

>EHO9887335.1 Fe-S cluster assembly transcriptional regulator IscR [Salmonella enterica subsp. enterica serovar Infantis] Overall Protective Antigen Prediction = **0.5676** (Probable **ANTIGEN**).

>EHO9887336.1 cysteine desulfurase [Salmonella enterica subsp. enterica serovar Infantis] Overall Protective Antigen Prediction = **0.4623** (Probable **ANTIGEN**).

>EHO9887337.1 Fe-S cluster assembly scaffold IscU [Salmonella enterica subsp. enterica serovar Infantis] Overall Protective Antigen Prediction = **0.6848** (Probable **ANTIGEN**).

>EHO9887338.1 iron-sulfur cluster assembly protein IscA [Salmonella enterica subsp. enterica serovar Infantis] Overall Protective Antigen Prediction = **0.8214** (Probable **ANTIGEN**).

>EHO9887339.1 co-chaperone HscB [Salmonella enterica subsp. enterica serovar Infantis] Overall Protective Antigen Prediction = **0.4728** (Probable **ANTIGEN**).

>EHO9887340.1 Fe-S protein assembly chaperone HscA [Salmonella enterica subsp. enterica serovar Infantis] Overall Protective Antigen Prediction = **0.5009** (Probable **ANTIGEN**).

>EHO9887341.1 ISC system 2Fe-2S type ferredoxin [Salmonella enterica subsp. enterica serovar Infantis] Overall Protective Antigen Prediction = **0.6446** (Probable **ANTIGEN**).

>EHO9887342.1 Fe-S cluster assembly protein IscX [Salmonella enterica subsp. enterica serovar Infantis] Overall Protective Antigen Prediction = **0.4973** (Probable **ANTIGEN**).

>EHO9887343.1 aminopeptidase PepB [Salmonella enterica subsp. enterica serovar Infantis] Overall Protective Antigen Prediction = **0.5530** (Probable **ANTIGEN**).

>EHO9887344.1 enhanced serine sensitivity protein SseB [Salmonella enterica subsp. enterica serovar Infantis] Overall Protective Antigen Prediction = **0.1164** (Probable **NON-ANTIGEN**).

>EHO9887345.1 DUF5066 family protein [Salmonella enterica subsp. enterica serovar Infantis] Overall Protective Antigen Prediction = **0.3011** (Probable **NON-ANTIGEN**).

>EHO9887346.1 hypothetical protein KND05_002718 [Salmonella enterica subsp. enterica serovar Infantis] Overall Protective Antigen Prediction = **0.5618** (Probable **ANTIGEN**).

>EHO9887347.1 3-mercaptopyruvate sulfurtransferase [Salmonella enterica subsp. enterica serovar Infantis] Overall Protective Antigen Prediction = **0.4579** (Probable **ANTIGEN**).

>EHO9887348.1 alpha-2-macroglobulin family protein [Salmonella enterica subsp. enterica serovar Infantis] Overall Protective Antigen Prediction = **0.5553** (Probable **ANTIGEN**).

>EHO9887349.1 peptidoglycan glycosyltransferase PbpC [Salmonella enterica subsp. enterica serovar Infantis] Overall Protective Antigen Prediction = **0.4719** (Probable **ANTIGEN**).

>EHO9887350.1 molybdopterin-dependent oxidoreductase [Salmonella enterica subsp. enterica serovar Infantis] Overall Protective Antigen Prediction = **0.4818** (Probable **ANTIGEN**).

>EHO9887351.1 dimethylsulfoxide reductase subunit B [Salmonella enterica subsp. enterica serovar Infantis] Overall Protective Antigen Prediction = **0.6415** (Probable **ANTIGEN**).

>EHO9887352.1 dimethyl sulfoxide reductase anchor subunit family protein [Salmonella enterica subsp. enterica serovar Infantis] Overall Protective Antigen Prediction = **0.4292** (Probable **ANTIGEN**).

>EHO9887353.1 4Fe-4S binding protein [Salmonella enterica subsp. enterica serovar Infantis] Overall Protective Antigen Prediction = **0.5358** (Probable **ANTIGEN**).

>EHO9887354.1 nucleoside-diphosphate kinase [Salmonella enterica subsp. enterica serovar Infantis] Overall Protective Antigen Prediction = **0.2865** (Probable **NON-ANTIGEN**).

>EHO9887355.1 bifunctional tRNA (adenosine(37)-C2)-methyltransferase TrmG/ribosomal RNA large subunit methyltransferase RlmN [Salmonella enterica subsp. enterica serovar Infantis] Overall Protective Antigen Prediction = **0.4348** (Probable **ANTIGEN**).

>EHO9887356.1 cytoskeleton protein RodZ [Salmonella enterica subsp. enterica serovar Infantis] Overall Protective Antigen Prediction = **0.6836** (Probable **ANTIGEN**).

>EHO9887357.1 flavodoxin-dependent (E)-4-hydroxy-3-methylbut-2-enyl-diphosphate synthase [Salmonella enterica subsp. enterica serovar Infantis] Overall Protective Antigen Prediction = **0.6427** (Probable **ANTIGEN**).

>EHO9887358.1 histidine--tRNA ligase [Salmonella enterica subsp. enterica serovar Infantis] Overall Protective Antigen Prediction = **0.5695** (Probable **ANTIGEN**).

>EHO9887359.1 YfgM family protein [Salmonella enterica subsp. enterica serovar Infantis] Overall Protective Antigen Prediction = **0.5948** (Probable **ANTIGEN**).

>EHO9887360.1 outer membrane protein assembly factor BamB [Salmonella enterica subsp. enterica serovar Infantis] Overall Protective Antigen Prediction = **0.4115** (Probable **ANTIGEN**).

>EHO9887361.1 ribosome biogenesis GTPase Der [Salmonella enterica subsp. enterica serovar Infantis] Overall Protective Antigen Prediction = **0.4285** (Probable **ANTIGEN**).

>EHO9887362.1 zinc ribbon domain-containing protein [Salmonella enterica subsp. enterica serovar Infantis] Overall Protective Antigen Prediction = **0.5989** (Probable **ANTIGEN**).

>EHO9887363.1 phage tail protein, partial [Salmonella enterica subsp. enterica serovar Infantis] Overall Protective Antigen Prediction = **0.4976** (Probable **ANTIGEN**).

>EHO9887364.1 phage tail protein I [Salmonella enterica subsp. enterica serovar Infantis] Overall Protective Antigen Prediction = **0.3818** (Probable **NON-ANTIGEN**).

>EHO9887365.1 baseplate assembly protein [Salmonella enterica subsp. enterica serovar Infantis] Overall Protective Antigen Prediction = **0.6072** (Probable **ANTIGEN**).

>EHO9887366.1 GPW/gp25 family protein [Salmonella enterica subsp. enterica serovar Infantis] Overall Protective Antigen Prediction = **0.5308** (Probable **ANTIGEN**).

>EHO9887367.1 phage baseplate assembly protein V [Salmonella enterica subsp. enterica serovar Infantis] Overall Protective Antigen Prediction = **0.6017** (Probable **ANTIGEN**).

>EHO9887368.1 phage virion morphogenesis protein [Salmonella enterica subsp. enterica serovar Infantis] Overall Protective Antigen Prediction = **0.5851** (Probable **ANTIGEN**).

>EHO9887369.1 phage tail protein [Salmonella enterica subsp. enterica serovar Infantis] Overall Protective Antigen Prediction = **0.4270** (Probable **ANTIGEN**).

>EHO9887370.1 phage lysis protein [Salmonella enterica subsp. enterica serovar Infantis] Overall Protective Antigen Prediction = **0.8666** (Probable **ANTIGEN**).

>EHO9887371.1 LysB family phage lysis regulatory protein [Salmonella enterica subsp. enterica serovar Infantis] Overall Protective Antigen Prediction = **0.3018** (Probable **NON-ANTIGEN**).

>EHO9887372.1 protein lysA [Salmonella enterica subsp. enterica serovar Infantis] Overall Protective Antigen Prediction = **0.5974** (Probable **ANTIGEN**).

>EHO9887373.1 glycoside hydrolase family 104 protein [Salmonella enterica subsp. enterica serovar Infantis] Overall Protective Antigen Prediction = **0.4264** (Probable **ANTIGEN**).

>EHO9887374.1 phage holin family protein [Salmonella enterica subsp. enterica serovar Infantis] Overall Protective Antigen Prediction = **0.2765** (Probable **NON-ANTIGEN**).

>EHO9887375.1 tail protein X [Salmonella enterica subsp. enterica serovar Infantis] Overall Protective Antigen Prediction = **0.4155** (Probable **ANTIGEN**).

>EHO9887376.1 head completion/stabilization protein [Salmonella enterica subsp. enterica serovar Infantis] Overall Protective Antigen Prediction = **0.6463** (Probable **ANTIGEN**).

>EHO9887377.1 terminase endonuclease subunit [Salmonella enterica subsp. enterica serovar Infantis] Overall Protective Antigen Prediction = **0.6291** (Probable **ANTIGEN**).

>EHO9887378.1 phage major capsid protein, P2 family [Salmonella enterica subsp. enterica serovar Infantis] Overall Protective Antigen Prediction = **0.5813** (Probable **ANTIGEN**).

>EHO9887379.1 GPO family capsid scaffolding protein [Salmonella enterica subsp. enterica serovar Infantis] Overall Protective Antigen Prediction = **0.5985** (Probable **ANTIGEN**).

>EHO9887380.1 terminase ATPase subunit family protein [Salmonella enterica subsp. enterica serovar Infantis] Overall Protective Antigen Prediction = **0.3947** (Probable **NON-ANTIGEN**).

>EHO9887381.1 phage portal protein [Salmonella enterica subsp. enterica serovar Infantis] Overall Protective Antigen Prediction = **0.2725** (Probable **NON-ANTIGEN**).

>EHO9887382.1 hypothetical protein KND05_002754 [Salmonella enterica subsp. enterica serovar Infantis] Overall Protective Antigen Prediction = **0.4945** (Probable **ANTIGEN**).

>EHO9887383.1 hypothetical protein KND05_002755 [Salmonella enterica subsp. enterica serovar Infantis] Overall Protective Antigen Prediction = **0.5534** (Probable **ANTIGEN**).

>EHO9887384.1 ImmA/IrrE family metallo-endopeptidase [Salmonella enterica subsp. enterica serovar Infantis] Overall Protective Antigen Prediction = **0.5477** (Probable **ANTIGEN**).

>EHO9887385.1 replication endonuclease [Salmonella enterica subsp. enterica serovar Infantis] Overall Protective Antigen Prediction = **0.4782** (Probable **ANTIGEN**).

>EHO9887386.1 DUF5405 family protein [Salmonella enterica subsp. enterica serovar Infantis] Overall Protective Antigen Prediction = **0.5921** (Probable **ANTIGEN**).

>EHO9887387.1 TraR/DksA C4-type zinc finger protein [Salmonella enterica subsp. enterica serovar Infantis] Overall Protective Antigen Prediction = **0.3088** (Probable **NON-ANTIGEN**).

>EHO9887388.1 DUF5405 family protein [Salmonella enterica subsp. enterica serovar Infantis] Overall Protective Antigen Prediction = **0.2122** (Probable **NON-ANTIGEN**).

>EHO9887389.1 DUF2732 family protein [Salmonella enterica subsp. enterica serovar Infantis] Overall Protective Antigen Prediction = **0.3267** (Probable **NON-ANTIGEN**).

>EHO9887390.1 replication protein B [Salmonella enterica subsp. enterica serovar Infantis] Overall Protective Antigen Prediction = **0.3006** (Probable **NON-ANTIGEN**).

>EHO9887391.1 hypothetical protein KND05_002763 [Salmonella enterica subsp. enterica serovar Infantis] Overall Protective Antigen Prediction = **0.4044** (Probable **ANTIGEN**).

>EHO9887392.1 helix-turn-helix domain-containing protein [Salmonella enterica subsp. enterica serovar Infantis] Overall Protective Antigen Prediction = **0.5374** (Probable **ANTIGEN**).

>EHO9887393.1 tyrosine-type recombinase/integrase [Salmonella enterica subsp. enterica serovar Infantis] Overall Protective Antigen Prediction = **0.5110** (Probable **ANTIGEN**).

>EHO9887394.1 cell-envelope stress modulator CpxP [Salmonella enterica subsp. enterica serovar Infantis] Overall Protective Antigen Prediction = **0.4751** (Probable **ANTIGEN**).

>EHO9887395.1 envelope stress response regulator transcription factor CpxR [Salmonella enterica subsp. enterica serovar Infantis] Overall Protective Antigen Prediction = **0.3871** (Probable **NON-ANTIGEN**).

>EHO9887396.1 envelope stress sensor histidine kinase CpxA [Salmonella enterica subsp. enterica serovar Infantis] Overall Protective Antigen Prediction = **0.5243** (Probable **ANTIGEN**).

>EHO9887397.1 nuclear transport factor 2 family protein [Salmonella enterica subsp. enterica serovar Infantis] Overall Protective Antigen Prediction = **0.1725** (Probable **NON-ANTIGEN**).

>EHO9887398.1 6-N-hydroxylaminopurine resistance protein [Salmonella enterica subsp. enterica serovar Infantis] Overall Protective Antigen Prediction = **0.5084** (Probable **ANTIGEN**).

>EHO9887399.1 superoxide dismutase [Mn] [Salmonella enterica subsp. enterica serovar Infantis] Overall Protective Antigen Prediction = **0.4761** (Probable **ANTIGEN**).

>EHO9887400.1 TRAP transporter substrate-binding protein [Salmonella enterica subsp. enterica serovar Infantis] Overall Protective Antigen Prediction = **0.3874** (Probable **NON-ANTIGEN**).

>EHO9887401.1 TRAP transporter small permease [Salmonella enterica subsp. enterica serovar Infantis] Overall Protective Antigen Prediction = **0.6086** (Probable **ANTIGEN**).

>EHO9887402.1 TRAP transporter large permease [Salmonella enterica subsp. enterica serovar Infantis] Overall Protective Antigen Prediction = **0.4519** (Probable **ANTIGEN**).

>EHO9887403.1 oligogalacturonate-specific porin KdgM family protein [Salmonella enterica subsp. enterica serovar Infantis] Overall Protective Antigen Prediction = **0.7905** (Probable **ANTIGEN**).

>EHO9887404.1 L-rhamnose/proton symporter RhaT [Salmonella enterica subsp. enterica serovar Infantis] Overall Protective Antigen Prediction = **0.6126** (Probable **ANTIGEN**).

>EHO9887405.1 HTH-type transcriptional activator RhaR [Salmonella enterica subsp. enterica serovar Infantis] Overall Protective Antigen Prediction = **0.3913** (Probable **NON-ANTIGEN**).

>EHO9887406.1 HTH-type transcriptional activator RhaS [Salmonella enterica subsp. enterica serovar Infantis] Overall Protective Antigen Prediction = **0.3428** (Probable **NON-ANTIGEN**).

>EHO9887407.1 rhamnulokinase [Salmonella enterica subsp. enterica serovar Infantis] Overall Protective Antigen Prediction = **0.4257** (Probable **ANTIGEN**).

>EHO9887408.1 L-rhamnose isomerase [Salmonella enterica subsp. enterica serovar Infantis] Overall Protective Antigen Prediction = **0.3713** (Probable **NON-ANTIGEN**).

>EHO9887409.1 rhamnulose-1-phosphate aldolase [Salmonella enterica subsp. enterica serovar Infantis] Overall Protective Antigen Prediction = **0.4434** (Probable **ANTIGEN**).

>EHO9887410.1 lactaldehyde reductase [Salmonella enterica subsp. enterica serovar Infantis] Overall Protective Antigen Prediction = **0.4225** (Probable **ANTIGEN**).

>EHO9887411.1 L-rhamnose mutarotase [Salmonella enterica subsp. enterica serovar Infantis] Overall Protective Antigen Prediction = **0.3956** (Probable **NON-ANTIGEN**).

>EHO9887412.1 helix-turn-helix transcriptional regulator [Salmonella enterica subsp. enterica serovar Infantis] Overall Protective Antigen Prediction = **0.2952** (Probable **NON-ANTIGEN**).

>EHO9887413.1 AzlC family ABC transporter permease [Salmonella enterica subsp. enterica serovar Infantis] Overall Protective Antigen Prediction = **0.4648** (Probable **ANTIGEN**).

>EHO9887414.1 AzlD domain-containing protein [Salmonella enterica subsp. enterica serovar Infantis] Overall Protective Antigen Prediction = **0.2519** (Probable **NON-ANTIGEN**).

>EHO9887415.1 DUF3829 domain-containing protein [Salmonella enterica subsp. enterica serovar Infantis] Overall Protective Antigen Prediction = **0.4192** (Probable **ANTIGEN**).

>EHO9887416.1 YiiG family protein [Salmonella enterica subsp. enterica serovar Infantis] Overall Protective Antigen Prediction = **0.4804** (Probable **ANTIGEN**).

>EHO9887417.1 formate dehydrogenase accessory sulfurtransferase FdhD [Salmonella enterica subsp. enterica serovar Infantis] Overall Protective Antigen Prediction = **0.2919** (Probable **NON-ANTIGEN**).

>EHO9887418.1 formate dehydrogenase-N subunit alpha [Salmonella enterica subsp. enterica serovar Infantis] Overall Protective Antigen Prediction = **0.3986** (Probable **NON-ANTIGEN**).

>EHO9887419.1 formate dehydrogenase subunit beta [Salmonella enterica subsp. enterica serovar Infantis] Overall Protective Antigen Prediction = **0.5034** (Probable **ANTIGEN**).

>EHO9887420.1 formate dehydrogenase cytochrome b556 subunit [Salmonella enterica subsp.

enterica serovar Infantis] Overall Protective Antigen Prediction = **0.6553** (Probable **ANTIGEN**).

>EHO9887421.1 formate dehydrogenase accessory protein FdhE [Salmonella enterica subsp. enterica serovar Infantis] Overall Protective Antigen Prediction = **0.6040** (Probable **ANTIGEN**).

>EHO9887422.1 DNA-binding transcriptional regulator [Salmonella enterica subsp. enterica serovar Infantis] Overall Protective Antigen Prediction = **0.2334** (Probable **NON-ANTIGEN**).

>EHO9887423.1 type II toxin-antitoxin system RelE/ParE family toxin [Salmonella enterica subsp. enterica serovar Infantis] Overall Protective Antigen Prediction = **0.4140** (Probable **ANTIGEN**).

>EHO9887424.1 alpha/beta hydrolase [Salmonella enterica subsp. enterica serovar Infantis] Overall Protective Antigen Prediction = **0.5286** (Probable **ANTIGEN**).

>EHO9887425.1 type II toxin-antitoxin system HigB family toxin [Salmonella enterica subsp. enterica serovar Infantis] Overall Protective Antigen Prediction = **0.2941** (Probable **NON-ANTIGEN**).

>EHO9887426.1 type II toxin-antitoxin system HigA family antitoxin [Salmonella enterica subsp. enterica serovar Infantis] Overall Protective Antigen Prediction = **0.5038** (Probable **ANTIGEN**).

>EHO9887427.1 fatty acid biosynthesis protein FabY [Salmonella enterica subsp. enterica serovar Infantis] Overall Protective Antigen Prediction = **0.4859** (Probable **ANTIGEN**).

>EHO9887428.1 D-tyrosyl-tRNA(Tyr) deacylase [Salmonella enterica subsp. enterica serovar Infantis] Overall Protective Antigen Prediction = **0.7418** (Probable **ANTIGEN**).

>EHO9887429.1 virulence factor BrkB family protein [Salmonella enterica subsp. enterica serovar Infantis] Overall Protective Antigen Prediction = **0.7373** (Probable **ANTIGEN**).

>EHO9887430.1 glucose-1-phosphatase [Salmonella enterica subsp. enterica serovar Infantis] Overall Protective Antigen Prediction = **0.3294** (Probable **NON-ANTIGEN**).

>EHO9887431.1 DeoR/GlpR transcriptional regulator [Salmonella enterica subsp. enterica serovar Infantis] Overall Protective Antigen Prediction = **0.2794** (Probable **NON-ANTIGEN**).

>EHO9887432.1 sugar kinase [Salmonella enterica subsp. enterica serovar Infantis] Overall Protective Antigen Prediction = **0.5009** (Probable **ANTIGEN**).

>EHO9887433.1 sulfolactaldehyde 3-reductase [Salmonella enterica subsp. enterica serovar Infantis] Overall Protective Antigen Prediction = **0.5294** (Probable **ANTIGEN**).

>EHO9887434.1 sulfofructosephosphate aldolase [Salmonella enterica subsp. enterica serovar Infantis] Overall Protective Antigen Prediction = **0.4243** (Probable **ANTIGEN**).

>EHO9887435.1 sulfoquinovose isomerase [Salmonella enterica subsp. enterica serovar Infantis] Overall Protective Antigen Prediction = **0.3489** (Probable **NON-ANTIGEN**).

>EHO9887436.1 aldose-1-epimerase [Salmonella enterica subsp. enterica serovar Infantis] Overall Protective Antigen Prediction = **0.4642** (Probable **ANTIGEN**).

>EHO9887437.1 alpha-glucosidase [Salmonella enterica subsp. enterica serovar Infantis] Overall Protective Antigen Prediction = **0.4708** (Probable **ANTIGEN**).

>EHO9887438.1 MFS transporter [Salmonella enterica subsp. enterica serovar Infantis] Overall Protective Antigen Prediction = **0.4191** (Probable **ANTIGEN**).

>EHO9887439.1 MFS transporter [Salmonella enterica subsp. enterica serovar Infantis] Overall Protective Antigen Prediction = **0.4105** (Probable **ANTIGEN**).

>EHO9887440.1 porin OmpL [Salmonella enterica subsp. enterica serovar Infantis] Overall Protective Antigen Prediction = **0.7802** (Probable **ANTIGEN**).

>EHO9887441.1 STM4015 family protein [Salmonella enterica subsp. enterica serovar Infantis] Overall Protective Antigen Prediction = **0.3866** (Probable **NON-ANTIGEN**).

>EHO9887442.1 STM4014 family protein [Salmonella enterica subsp. enterica serovar Infantis] Overall Protective Antigen Prediction = **0.4215** (Probable **ANTIGEN**).

>EHO9887443.1 STM4013/SEN3800 family hydrolase [Salmonella enterica subsp. enterica serovar Infantis] Overall Protective Antigen Prediction = **0.4104** (Probable **ANTIGEN**).

>EHO9887444.1 STM4012 family radical SAM protein [Salmonella enterica subsp. enterica serovar Infantis] Overall Protective Antigen Prediction = **0.3342** (Probable **NON-ANTIGEN**).

>EHO9887445.1 STM4011 family radical SAM protein [Salmonella enterica subsp. enterica serovar Infantis] Overall Protective Antigen Prediction = **0.4769** (Probable **ANTIGEN**).

>EHO9887446.1 HAD family phosphatase [Salmonella enterica subsp. enterica serovar Infantis] Overall Protective Antigen Prediction = **0.3802** (Probable **NON-ANTIGEN**).

>EHO9887447.1 ribosome-dependent GTPase TypA [Salmonella enterica subsp. enterica serovar Infantis] Overall Protective Antigen Prediction = **0.5329** (Probable **ANTIGEN**).

>EHO9887448.1 glutamate--ammonia ligase [Salmonella enterica subsp. enterica serovar Infantis] Overall Protective Antigen Prediction = **0.5576** (Probable **ANTIGEN**).

>EHO9887449.1 nitrogen regulation protein NR(II) [Salmonella enterica subsp. enterica serovar Infantis] Overall Protective Antigen Prediction = **0.3152** (Probable **NON-ANTIGEN**).

>EHO9887450.1 nitrogen regulation protein NR(I) [Salmonella enterica subsp. enterica serovar Infantis] Overall Protective Antigen Prediction = **0.3652** (Probable **NON-ANTIGEN**).

>EHO9887451.1 YshB family small membrane protein [Salmonella enterica subsp. enterica serovar Infantis] Overall Protective Antigen Prediction = **0.3399** (Probable **NON-ANTIGEN**).

>EHO9887452.1 oxygen-independent coproporphyrinogen III oxidase [Salmonella enterica subsp. enterica serovar Infantis] Overall Protective Antigen Prediction = **0.3542** (Probable **NON-ANTIGEN**).

>EHO9887453.1 Der GTPase-activating protein YihI [Salmonella enterica subsp. enterica serovar Infantis] Overall Protective Antigen Prediction = **0.8371** (Probable **ANTIGEN**).

>EHO9887454.1 hypothetical protein KND05_002826 [Salmonella enterica subsp. enterica serovar Infantis] Overall Protective Antigen Prediction = **0.9157** (Probable **ANTIGEN**).

>EHO9887455.1 YihA family ribosome biogenesis GTP-binding protein [Salmonella enterica subsp. enterica serovar Infantis] Overall Protective Antigen Prediction = **0.3959** (Probable **NON-ANTIGEN**

).

>EHO9887456.1 DNA polymerase I [Salmonella enterica subsp. enterica serovar Infantis] Overall Protective Antigen Prediction = **0.5151** (Probable **ANTIGEN**).

>EHO9887457.1 acyltransferase [Salmonella enterica subsp. enterica serovar Infantis] Overall Protective Antigen Prediction = **0.5348** (Probable **ANTIGEN**).

>EHO9887458.1 thiol:disulfide interchange protein DsbA [Salmonella enterica subsp. enterica serovar Infantis] Overall Protective Antigen Prediction = **0.4377** (Probable **ANTIGEN**).

>EHO9887459.1 serine/threonine protein kinase [Salmonella enterica subsp. enterica serovar Infantis] Overall Protective Antigen Prediction = **0.3383** (Probable **NON-ANTIGEN**).

>EHO9887460.1 YihD family protein [Salmonella enterica subsp. enterica serovar Infantis] Overall Protective Antigen Prediction = **0.4690** (Probable **ANTIGEN**).

>EHO9887461.1 molybdenum cofactor guanylyltransferase MobA [Salmonella enterica subsp. enterica serovar Infantis] Overall Protective Antigen Prediction = **0.2448** (Probable **NON-ANTIGEN**).

>EHO9887462.1 molybdopterin-guanine dinucleotide biosynthesis protein B [Salmonella enterica subsp. enterica serovar Infantis] Overall Protective Antigen Prediction = **0.5819** (Probable **ANTIGEN**).

>EHO9887463.1 menaquinone-dependent protoporphyrinogen IX dehydrogenase [Salmonella enterica subsp. enterica serovar Infantis] Overall Protective Antigen Prediction = **0.3596** (Probable **NON-ANTIGEN**).

>EHO9887464.1 Trk system potassium transporter TrkH [Salmonella enterica subsp. enterica serovar Infantis] Overall Protective Antigen Prediction = **0.5327** (Probable **ANTIGEN**).

>EHO9887465.1 IMPACT family protein [Salmonella enterica subsp. enterica serovar Infantis] Overall Protective Antigen Prediction = **0.3938** (Probable **NON-ANTIGEN**).

>EHO9887466.1 Xaa-Pro dipeptidase [Salmonella enterica subsp. enterica serovar Infantis] Overall Protective Antigen Prediction = **0.4095** (Probable **ANTIGEN**).

>EHO9887467.1 fatty acid oxidation complex subunit alpha FadB [Salmonella enterica subsp. enterica serovar Infantis] Overall Protective Antigen Prediction = **0.3506** (Probable **NON-ANTIGEN**).

>EHO9887468.1 acetyl-CoA C-acyltransferase FadA [Salmonella enterica subsp. enterica serovar Infantis] Overall Protective Antigen Prediction = **0.4539** (Probable **ANTIGEN**).

>EHO9887469.1 aryl-sulfate sulfotransferase [Salmonella enterica subsp. enterica serovar Infantis] Overall Protective Antigen Prediction = **0.6228** (Probable **ANTIGEN**).

>EHO9887470.1 NAD(P)H-flavin reductase [Salmonella enterica subsp. enterica serovar Infantis] Overall Protective Antigen Prediction = **0.3526** (Probable **NON-ANTIGEN**).

>EHO9887471.1 4-hydroxy-3-polyprenylbenzoate decarboxylase [Salmonella enterica subsp. enterica serovar Infantis] Overall Protective Antigen Prediction = **0.2334** (Probable **NON-ANTIGEN**).

>EHO9887472.1 transcription/translation regulatory transformer protein RfaH [Salmonella enterica subsp. enterica serovar Infantis] Overall Protective Antigen Prediction = **0.4415** (Probable **ANTIGEN**).

>EHO9887473.1 3'-5' ssDNA/RNA exonuclease TatD [Salmonella enterica subsp. enterica serovar Infantis] Overall Protective Antigen Prediction = **0.3752** (Probable **NON-ANTIGEN**).

>EHO9887474.1 Sec-independent protein translocase subunit TatC [Salmonella enterica subsp. enterica serovar Infantis] Overall Protective Antigen Prediction = **0.6392** (Probable **ANTIGEN**).

>EHO9887475.1 Sec-independent protein translocase subunit TatB [Salmonella enterica subsp. enterica serovar Infantis] Overall Protective Antigen Prediction = **0.6397** (Probable **ANTIGEN**).

>EHO9887476.1 Sec-independent protein translocase subunit TatA [Salmonella enterica subsp. enterica serovar Infantis] Overall Protective Antigen Prediction = **0.8828** (Probable **ANTIGEN**).

>EHO9887477.1 ubiquinone biosynthesis regulatory protein kinase UbiB [Salmonella enterica subsp. enterica serovar Infantis] Overall Protective Antigen Prediction = **0.3435** (Probable **NON-ANTIGEN**).

>EHO9887478.1 ubiquinone biosynthesis protein UbiJ [Salmonella enterica subsp. enterica serovar Infantis] Overall Protective Antigen Prediction = **0.4646** (Probable **ANTIGEN**).

>EHO9887479.1 bifunctional demethylmenaquinone methyltransferase/2-methoxy-6-polyprenyl-1,4-benzoquinol methylase UbiE [Salmonella enterica subsp. enterica serovar Infantis] Overall Protective Antigen Prediction = **0.2796** (Probable **NON-ANTIGEN**).

>EHO9887480.1 DNA recombination protein RmuC [Salmonella enterica subsp. enterica serovar Infantis] Overall Protective Antigen Prediction = **0.4951** (Probable **ANTIGEN**).

>EHO9887481.1 uridine phosphorylase [Salmonella enterica subsp. enterica serovar Infantis] Overall Protective Antigen Prediction = **0.4891** (Probable **ANTIGEN**).

>EHO9887482.1 dienelactone hydrolase family protein [Salmonella enterica subsp. enterica serovar Infantis] Overall Protective Antigen Prediction = **0.4781** (Probable **ANTIGEN**).

>EHO9887483.1 anaerobic sulfatase maturase [Salmonella enterica subsp. enterica serovar Infantis] Overall Protective Antigen Prediction = **0.4876** (Probable **ANTIGEN**).

>EHO9887484.1 5-methyltetrahydropteroyltriglutamate--homocysteine S-methyltransferase [Salmonella enterica subsp. enterica serovar Infantis] Overall Protective Antigen Prediction = **0.4351** (Probable **ANTIGEN**).

>EHO9887485.1 HTH-type transcriptional regulator MetR [Salmonella enterica subsp. enterica serovar Infantis] Overall Protective Antigen Prediction = **0.4086** (Probable **ANTIGEN**).

>EHO9887486.1 DMT family transporter [Salmonella enterica subsp. enterica serovar Infantis] Overall Protective Antigen Prediction = **0.5880** (Probable **ANTIGEN**).

>EHO9887487.1 sugar/pyridoxal phosphate phosphatase YigL [Salmonella enterica subsp. enterica serovar Infantis] Overall Protective Antigen Prediction = **0.4962** (Probable **ANTIGEN**).

>EHO9887488.1 lysophospholipase L2 [Salmonella enterica subsp. enterica serovar Infantis] Overall Protective Antigen Prediction = **0.4358** (Probable **ANTIGEN**).

>EHO9887489.1 homoserine/homoserine lactone efflux protein [Salmonella enterica subsp. enterica serovar Infantis] Overall Protective Antigen Prediction = **0.3762** (Probable **NON-ANTIGEN**).

>EHO9887490.1 threonine export protein RhtC [Salmonella enterica subsp. enterica serovar Infantis] Overall Protective Antigen Prediction = **0.2689** (Probable **NON-ANTIGEN**).

>EHO9887491.1 ATP-dependent DNA helicase RecQ [Salmonella enterica subsp. enterica serovar Infantis] Overall Protective Antigen Prediction = **0.4181** (Probable **ANTIGEN**).

>EHO9887492.1 phospholipase A [Salmonella enterica subsp. enterica serovar Infantis] Overall Protective Antigen Prediction = **0.4982** (Probable **ANTIGEN**).

>EHO9887493.1 thioesterase family protein [Salmonella enterica subsp. enterica serovar Infantis] Overall Protective Antigen Prediction = **0.4140** (Probable **ANTIGEN**).

>EHO9887494.1 EamA family transporter RarD [Salmonella enterica subsp. enterica serovar Infantis] Overall Protective Antigen Prediction = **0.6249** (Probable **ANTIGEN**).

>EHO9887495.1 hypothetical protein KND05_002868 [Salmonella enterica subsp. enterica serovar Infantis] Overall Protective Antigen Prediction = **0.2038** (Probable **NON-ANTIGEN**).

>EHO9887496.1 hypothetical protein KND05_002869 [Salmonella enterica subsp. enterica serovar Infantis] Overall Protective Antigen Prediction = **0.6843** (Probable **ANTIGEN**).

>EHO9887497.1 magnesium/cobalt transporter CorA [Salmonella enterica subsp. enterica serovar Infantis] Overall Protective Antigen Prediction = **0.4627** (Probable **ANTIGEN**).

>EHO9887498.1 DNA helicase II [Salmonella enterica subsp. enterica serovar Infantis] Overall Protective Antigen Prediction = **0.3565** (Probable **NON-ANTIGEN**).

>EHO9887499.1 5-amino-6-(5-phospho-D-ribitylamino)uracil phosphatase YigB [Salmonella enterica subsp. enterica serovar Infantis] Overall Protective Antigen Prediction = **0.3178** (Probable **NON-ANTIGEN**).

>EHO9887500.1 tyrosine recombinase XerC [Salmonella enterica subsp. enterica serovar Infantis] Overall Protective Antigen Prediction = **0.4432** (Probable **ANTIGEN**).

>EHO9887501.1 DUF484 domain-containing protein [Salmonella enterica subsp. enterica serovar Infantis] Overall Protective Antigen Prediction = **0.3778** (Probable **NON-ANTIGEN**).

>EHO9887502.1 diaminopimelate epimerase [Salmonella enterica subsp. enterica serovar Infantis] Overall Protective Antigen Prediction = **0.6423** (Probable **ANTIGEN**).

>EHO9887503.1 lipoprotein [Salmonella enterica subsp. enterica serovar Infantis] Overall Protective Antigen Prediction = **0.7177** (Probable **ANTIGEN**).

>EHO9887504.1 hypothetical protein KND05_002877 [Salmonella enterica subsp. enterica serovar Infantis] Overall Protective Antigen Prediction = **0.7215** (Probable **ANTIGEN**).

>EHO9887505.1 DUF3021 family protein [Salmonella enterica subsp. enterica serovar Infantis] Overall Protective Antigen Prediction = **0.5132** (Probable **ANTIGEN**).

>EHO9887506.1 iron donor protein CyaY [Salmonella enterica subsp. enterica serovar Infantis]

Overall Protective Antigen Prediction = **0.5874** (Probable **ANTIGEN**).

>EHO9887507.1 class I adenylate cyclase [Salmonella enterica subsp. enterica serovar Infantis] Overall Protective Antigen Prediction = **0.4248** (Probable **ANTIGEN**).

>EHO9887508.1 hydroxymethylbilane synthase [Salmonella enterica subsp. enterica serovar Infantis] Overall Protective Antigen Prediction = **0.4843** (Probable **ANTIGEN**).

>EHO9887509.1 uroporphyrinogen-III synthase [Salmonella enterica subsp. enterica serovar Infantis] Overall Protective Antigen Prediction = **0.3882** (Probable **NON-ANTIGEN**).

>EHO9887510.1 uroporphyrinogen-III C-methyltransferase [Salmonella enterica subsp. enterica serovar Infantis] Overall Protective Antigen Prediction = **0.4933** (Probable **ANTIGEN**).

>EHO9887511.1 protoheme IX biogenesis protein HemY [Salmonella enterica subsp. enterica serovar Infantis] Overall Protective Antigen Prediction = **0.4506** (Probable **ANTIGEN**).

>EHO9887512.1 amino acid permease [Salmonella enterica subsp. enterica serovar Infantis] Overall Protective Antigen Prediction = **0.4809** (Probable **ANTIGEN**).

>EHO9887513.1 lipopolysaccharide N-acetylmannosaminouronosyltransferase [Salmonella enterica subsp. enterica serovar Infantis] Overall Protective Antigen Prediction = **0.4338** (Probable **ANTIGEN**).

>EHO9887514.1 ECA oligosaccharide polymerase [Salmonella enterica subsp. enterica serovar Infantis] Overall Protective Antigen Prediction = **0.5776** (Probable **ANTIGEN**).

>EHO9887515.1 TDP-N-acetylfucosamine:lipid II N-acetylfucosaminyltransferase [Salmonella enterica subsp. enterica serovar Infantis] Overall Protective Antigen Prediction = **0.4213** (Probable **ANTIGEN**).

>EHO9887516.1 lipid III flippase WzxE [Salmonella enterica subsp. enterica serovar Infantis] Overall Protective Antigen Prediction = **0.2795** (Probable **NON-ANTIGEN**).

>EHO9887517.1 dTDP-4-amino-4,6-dideoxygalactose transaminase [Salmonella enterica subsp. enterica serovar Infantis] Overall Protective Antigen Prediction = **0.3935** (Probable **NON-ANTIGEN**).

>EHO9887518.1 dTDP-4-amino-4,6-dideoxy-D-galactose acyltransferase [Salmonella enterica subsp. enterica serovar Infantis] Overall Protective Antigen Prediction = **0.4894** (Probable **ANTIGEN**).

>EHO9887519.1 glucose-1-phosphate thymidylyltransferase RfbA [Salmonella enterica subsp. enterica serovar Infantis] Overall Protective Antigen Prediction = **0.3411** (Probable **NON-ANTIGEN**).

>EHO9887520.1 dTDP-glucose 4,6-dehydratase [Salmonella enterica subsp. enterica serovar Infantis] Overall Protective Antigen Prediction = **0.3917** (Probable **NON-ANTIGEN**).

>EHO9887521.1 UDP-N-acetyl-D-mannosamine dehydrogenase [Salmonella enterica subsp. enterica serovar Infantis] Overall Protective Antigen Prediction = **0.4708** (Probable **ANTIGEN**).

>EHO9887522.1 UDP-N-acetylglucosamine 2-epimerase (non-hydrolyzing) [Salmonella enterica subsp. enterica serovar Infantis] Overall Protective Antigen Prediction = **0.4204** (Probable **ANTIGEN**).

>EHO9887523.1 ECA polysaccharide chain length modulation protein [Salmonella enterica subsp. enterica serovar Infantis] Overall Protective Antigen Prediction = **0.4113** (Probable **ANTIGEN**).

>EHO9887524.1 UDP-N-acetylglucosamine--undecaprenyl-phosphate N-acetylglucosaminephosphotransferase [Salmonella enterica subsp. enterica serovar Infantis] Overall Protective Antigen Prediction = **0.4678** (Probable **ANTIGEN**).

>EHO9887525.1 transcription termination factor Rho [Salmonella enterica subsp. enterica serovar Infantis] Overall Protective Antigen Prediction = **0.3127** (Probable **NON-ANTIGEN**).

>EHO9887526.1 rho operon leader peptide [Salmonella enterica subsp. enterica serovar Infantis] Overall Protective Antigen Prediction = **0.4245** (Probable **ANTIGEN**).

>EHO9887527.1 thioredoxin TrxA [Salmonella enterica subsp. enterica serovar Infantis] Overall Protective Antigen Prediction = **0.3799** (Probable **NON-ANTIGEN**).

>EHO9887528.1 ATP-dependent RNA helicase RhlB [Salmonella enterica subsp. enterica serovar Infantis] Overall Protective Antigen Prediction = **0.5350** (Probable **ANTIGEN**).

>EHO9887529.1 guanosine-5'-triphosphate,3'-diphosphate diphosphatase [Salmonella enterica subsp. enterica serovar Infantis] Overall Protective Antigen Prediction = **0.4102** (Probable **ANTIGEN**).

>EHO9887530.1 DNA helicase Rep [Salmonella enterica subsp. enterica serovar Infantis] Overall Protective Antigen Prediction = **0.3473** (Probable **NON-ANTIGEN**).

>EHO9887531.1 peptidylprolyl isomerase PpiC [Salmonella enterica subsp. enterica serovar Infantis] Overall Protective Antigen Prediction = **0.2240** (Probable **NON-ANTIGEN**).

>EHO9887532.1 ketol-acid reductoisomerase [Salmonella enterica subsp. enterica serovar Infantis] Overall Protective Antigen Prediction = **0.3436** (Probable **NON-ANTIGEN**).

>EHO9887533.1 HTH-type transcriptional activator IlvY [Salmonella enterica subsp. enterica serovar Infantis] Overall Protective Antigen Prediction = **0.4888** (Probable **ANTIGEN**).

>EHO9887534.1 XRE family transcriptional regulator [Salmonella enterica subsp. enterica serovar Infantis] Overall Protective Antigen Prediction = **0.1619** (Probable **NON-ANTIGEN**).

>EHO9887535.1 type II toxin-antitoxin system RelE/ParE family toxin [Salmonella enterica subsp. enterica serovar Infantis] Overall Protective Antigen Prediction = **0.5629** (Probable **ANTIGEN**).

>EHO9887536.1 threonine ammonia-lyase, biosynthetic [Salmonella enterica subsp. enterica serovar Infantis] Overall Protective Antigen Prediction = **0.4979** (Probable **ANTIGEN**).

>EHO9887537.1 dihydroxy-acid dehydratase [Salmonella enterica subsp. enterica serovar Infantis] Overall Protective Antigen Prediction = **0.5130** (Probable **ANTIGEN**).

>EHO9887538.1 branched-chain-amino-acid transaminase [Salmonella enterica subsp. enterica serovar Infantis] Overall Protective Antigen Prediction = **0.4111** (Probable **ANTIGEN**).

>EHO9887539.1 acetolactate synthase 2 small subunit [Salmonella enterica subsp. enterica serovar Infantis] Overall Protective Antigen Prediction = **0.6148** (Probable **ANTIGEN**).

>EHO9887540.1 acetolactate synthase 2 catalytic subunit [Salmonella enterica subsp. enterica serovar

Infantis] Overall Protective Antigen Prediction = **0.4352** (Probable **ANTIGEN**).

>EHO9887541.1 hypothetical protein KND05_002920 [Salmonella enterica subsp. enterica serovar Infantis] Overall Protective Antigen Prediction = **0.2045** (Probable **NON-ANTIGEN**).

>EHO9887542.1 ilv operon leader peptide [Salmonella enterica subsp. enterica serovar Infantis] Overall Protective Antigen Prediction = **0.4242** (Probable **ANTIGEN**).

>EHO9887543.1 YifB family Mg chelatase-like AAA ATPase [Salmonella enterica subsp. enterica serovar Infantis] Overall Protective Antigen Prediction = **0.4498** (Probable **ANTIGEN**).

>EHO9887544.1 macrodomain Ori organization protein MaoP [Salmonella enterica subsp. enterica serovar Infantis] Overall Protective Antigen Prediction = **0.2617** (Probable **NON-ANTIGEN**).

>EHO9887545.1 HTH-type transcriptional regulator HdfR [Salmonella enterica subsp. enterica serovar Infantis] Overall Protective Antigen Prediction = **0.4700** (Probable **ANTIGEN**).

>EHO9887546.1 Kef family K(+) transporter [Salmonella enterica subsp. enterica serovar Infantis] Overall Protective Antigen Prediction = **0.3855** (Probable **NON-ANTIGEN**).

>EHO9887547.1 inosine/guanosine kinase [Salmonella enterica subsp. enterica serovar Infantis] Overall Protective Antigen Prediction = **0.4444** (Probable **ANTIGEN**).

>EHO9887548.1 acetyl esterase [Salmonella enterica subsp. enterica serovar Infantis] Overall Protective Antigen Prediction = **0.3832** (Probable **NON-ANTIGEN**).

>EHO9887549.1 ferrochelatase [Salmonella enterica subsp. enterica serovar Infantis] Overall Protective Antigen Prediction = **0.2881** (Probable **NON-ANTIGEN**).

>EHO9887550.1 adenylate kinase [Salmonella enterica subsp. enterica serovar Infantis] Overall Protective Antigen Prediction = **0.6723** (Probable **ANTIGEN**).

>EHO9887551.1 molecular chaperone HtpG [Salmonella enterica subsp. enterica serovar Infantis] Overall Protective Antigen Prediction = **0.3782** (Probable **NON-ANTIGEN**).

>EHO9887552.1 recombination protein RecR [Salmonella enterica subsp. enterica serovar Infantis] Overall Protective Antigen Prediction = **0.5313** (Probable **ANTIGEN**).

>EHO9887553.1 YbaB/EbfC family nucleoid-associated protein [Salmonella enterica subsp. enterica serovar Infantis] Overall Protective Antigen Prediction = **0.5934** (Probable **ANTIGEN**).

>EHO9887554.1 DNA polymerase III subunit gamma/tau [Salmonella enterica subsp. enterica serovar Infantis] Overall Protective Antigen Prediction = **0.4590** (Probable **ANTIGEN**).

>EHO9887555.1 adenine phosphoribosyltransferase [Salmonella enterica subsp. enterica serovar Infantis] Overall Protective Antigen Prediction = **0.3843** (Probable **NON-ANTIGEN**).

>EHO9887556.1 DUF454 family protein [Salmonella enterica subsp. enterica serovar Infantis] Overall Protective Antigen Prediction = **0.7024** (Probable **ANTIGEN**).

>EHO9887557.1 primosomal replication protein N'' [Salmonella enterica subsp. enterica serovar Infantis] Overall Protective Antigen Prediction = **0.2855** (Probable **NON-ANTIGEN**).

>EHO9887558.1 pleiotropic regulatory protein RsmS [Salmonella enterica subsp. enterica serovar

Infantis] Overall Protective Antigen Prediction = **0.5279** (Probable **ANTIGEN**).

>EHO9887559.1 mechanosensitive channel MscK [Salmonella enterica subsp. enterica serovar Infantis] Overall Protective Antigen Prediction = **0.4942** (Probable **ANTIGEN**).

>EHO9887560.1 multidrug efflux transporter transcriptional repressor AcrR [Salmonella enterica subsp. enterica serovar Infantis] Overall Protective Antigen Prediction = **0.3972** (Probable **NON-ANTIGEN**).

>EHO9887561.1 multidrug efflux RND transporter periplasmic adaptor subunit AcrA [Salmonella enterica subsp. enterica serovar Infantis] Overall Protective Antigen Prediction = **0.6642** (Probable **ANTIGEN**).

>EHO9887562.1 multidrug efflux RND transporter permease subunit [Salmonella enterica subsp. enterica serovar Infantis] Overall Protective Antigen Prediction = **0.6191** (Probable **ANTIGEN**).

>EHO9887563.1 Hha toxicity modulator TomB [Salmonella enterica subsp. enterica serovar Infantis] Overall Protective Antigen Prediction = **0.3711** (Probable **NON-ANTIGEN**).

>EHO9887564.1 hemolysin expression modulator Hha [Salmonella enterica subsp. enterica serovar Infantis] Overall Protective Antigen Prediction = **0.3358** (Probable **NON-ANTIGEN**).

>EHO9887565.1 maltose O-acetyltransferase [Salmonella enterica subsp. enterica serovar Infantis] Overall Protective Antigen Prediction = **0.4678** (Probable **ANTIGEN**).

>EHO9887566.1 hypothetical protein KND05_002949 [Salmonella enterica subsp. enterica serovar Infantis] Overall Protective Antigen Prediction = **0.6169** (Probable **ANTIGEN**).

>EHO9887567.1 type B 50S ribosomal protein L36 [Salmonella enterica subsp. enterica serovar Infantis] Overall Protective Antigen Prediction = **0.6044** (Probable **ANTIGEN**).

>EHO9887568.1 type B 50S ribosomal protein L31 [Salmonella enterica subsp. enterica serovar Infantis] Overall Protective Antigen Prediction = **0.5297** (Probable **ANTIGEN**).

>EHO9887569.1 EAL domain-containing protein [Salmonella enterica subsp. enterica serovar Infantis] Overall Protective Antigen Prediction = **0.3699** (Probable **NON-ANTIGEN**).

>EHO9887570.1 MGMT family protein [Salmonella enterica subsp. enterica serovar Infantis] Overall Protective Antigen Prediction = **0.3700** (Probable **NON-ANTIGEN**).

>EHO9887571.1 YbaY family lipoprotein [Salmonella enterica subsp. enterica serovar Infantis] Overall Protective Antigen Prediction = **0.6636** (Probable **ANTIGEN**).

>EHO9887572.1 acyl-CoA thioesterase II [Salmonella enterica subsp. enterica serovar Infantis] Overall Protective Antigen Prediction = **0.3838** (Probable **NON-ANTIGEN**).

>EHO9887573.1 ammonium transporter AmtB [Salmonella enterica subsp. enterica serovar Infantis] Overall Protective Antigen Prediction = **0.3928** (Probable **NON-ANTIGEN**).

>EHO9887574.1 P-II family nitrogen regulator [Salmonella enterica subsp. enterica serovar Infantis] Overall Protective Antigen Prediction = **0.4610** (Probable **ANTIGEN**).

>EHO9887575.1 SmdB family multidrug efflux ABC transporter permease/ATP-binding protein [Salmonella enterica subsp. enterica serovar Infantis] Overall Protective Antigen Prediction = **0.3157** (

Probable **NON-ANTIGEN**).

>EHO9887576.1 SmdA family multidrug ABC transporter permease/ATP-binding protein [Salmonella enterica subsp. enterica serovar Infantis] Overall Protective Antigen Prediction = **0.5443** (Probable **ANTIGEN**).

>EHO9887577.1 DNA-binding transcriptional regulator DecR [Salmonella enterica subsp. enterica serovar Infantis] Overall Protective Antigen Prediction = **0.2416** (Probable **NON-ANTIGEN**).

>EHO9887578.1 PLP-dependent cysteine synthase family protein [Salmonella enterica subsp. enterica serovar Infantis] Overall Protective Antigen Prediction = **0.4915** (Probable **ANTIGEN**).

>EHO9887579.1 HMP-PP phosphatase [Salmonella enterica subsp. enterica serovar Infantis] Overall Protective Antigen Prediction = **0.3784** (Probable **NON-ANTIGEN**).

>EHO9887580.1 SgrR family transcriptional regulator [Salmonella enterica subsp. enterica serovar Infantis] Overall Protective Antigen Prediction = **0.4327** (Probable **ANTIGEN**).

>EHO9887581.1 7-cyano-7-deazaguanine synthase QueC [Salmonella enterica subsp. enterica serovar Infantis] Overall Protective Antigen Prediction = **0.4601** (Probable **ANTIGEN**).

>EHO9887582.1 long-chain acyl-CoA thioesterase FadM [Salmonella enterica subsp. enterica serovar Infantis] Overall Protective Antigen Prediction = **0.6032** (Probable **ANTIGEN**).

>EHO9887583.1 helix-hairpin-helix domain-containing protein [Salmonella enterica subsp. enterica serovar Infantis] Overall Protective Antigen Prediction = **0.6822** (Probable **ANTIGEN**).

>EHO9887584.1 peptidylprolyl isomerase [Salmonella enterica subsp. enterica serovar Infantis] Overall Protective Antigen Prediction = **0.5207** (Probable **ANTIGEN**).

>EHO9887585.1 DNA-binding protein HU-beta [Salmonella enterica subsp. enterica serovar Infantis] Overall Protective Antigen Prediction = **0.5598** (Probable **ANTIGEN**).

>EHO9887586.1 endopeptidase La [Salmonella enterica subsp. enterica serovar Infantis] Overall Protective Antigen Prediction = **0.4489** (Probable **ANTIGEN**).

>EHO9887587.1 ATP-dependent protease ATP-binding subunit ClpX [Salmonella enterica subsp. enterica serovar Infantis] Overall Protective Antigen Prediction = **0.4530** (Probable **ANTIGEN**).

>EHO9887588.1 ATP-dependent Clp endopeptidase proteolytic subunit ClpP [Salmonella enterica subsp. enterica serovar Infantis] Overall Protective Antigen Prediction = **0.3896** (Probable **NON-ANTIGEN**).

>EHO9887589.1 trigger factor [Salmonella enterica subsp. enterica serovar Infantis] Overall Protective Antigen Prediction = **0.5991** (Probable **ANTIGEN**).

>EHO9887590.1 transcriptional regulator BolA [Salmonella enterica subsp. enterica serovar Infantis] Overall Protective Antigen Prediction = **0.4999** (Probable **ANTIGEN**).

>EHO9887591.1 lipoprotein [Salmonella enterica subsp. enterica serovar Infantis] Overall Protective Antigen Prediction = **0.5927** (Probable **ANTIGEN**).

>EHO9887592.1 muropeptide MFS transporter AmpG [Salmonella enterica subsp. enterica serovar Infantis] Overall Protective Antigen Prediction = **0.3274** (Probable **NON-ANTIGEN**).

>EHO9887593.1 cytochrome o ubiquinol oxidase subunit II [Salmonella enterica subsp. enterica serovar Infantis] Overall Protective Antigen Prediction = **0.5820** (Probable **ANTIGEN**).

>EHO9887594.1 cytochrome o ubiquinol oxidase subunit I [Salmonella enterica subsp. enterica serovar Infantis] Overall Protective Antigen Prediction = **0.5486** (Probable **ANTIGEN**).

>EHO9887595.1 cytochrome o ubiquinol oxidase subunit III [Salmonella enterica subsp. enterica serovar Infantis] Overall Protective Antigen Prediction = **0.6541** (Probable **ANTIGEN**).

>EHO9887596.1 cytochrome o ubiquinol oxidase subunit IV [Salmonella enterica subsp. enterica serovar Infantis] Overall Protective Antigen Prediction = **0.8845** (Probable **ANTIGEN**).

>EHO9887597.1 protoheme IX farnesyltransferase [Salmonella enterica subsp. enterica serovar Infantis] Overall Protective Antigen Prediction = **0.4341** (Probable **ANTIGEN**).

>EHO9887598.1 sel1 repeat family protein [Salmonella enterica subsp. enterica serovar Infantis] Overall Protective Antigen Prediction = **0.5568** (Probable **ANTIGEN**).

>EHO9887599.1 sel1 repeat family protein [Salmonella enterica subsp. enterica serovar Infantis] Overall Protective Antigen Prediction = **0.5917** (Probable **ANTIGEN**).

>EHO9887600.1 MFS transporter [Salmonella enterica subsp. enterica serovar Infantis] Overall Protective Antigen Prediction = **0.5408** (Probable **ANTIGEN**).

>EHO9887601.1 YajQ family cyclic di-GMP-binding protein [Salmonella enterica subsp. enterica serovar Infantis] Overall Protective Antigen Prediction = **0.5871** (Probable **ANTIGEN**).

>EHO9887602.1 2-dehydropantoate 2-reductase [Salmonella enterica subsp. enterica serovar Infantis] Overall Protective Antigen Prediction = **0.2748** (Probable **NON-ANTIGEN**).

>EHO9887603.1 protein deglycase YajL [Salmonella enterica subsp. enterica serovar Infantis] Overall Protective Antigen Prediction = **0.3849** (Probable **NON-ANTIGEN**).

>EHO9887604.1 phosphonoacetaldehyde hydrolase [Salmonella enterica subsp. enterica serovar Infantis] Overall Protective Antigen Prediction = **0.5172** (Probable **ANTIGEN**).

>EHO9887605.1 2-aminoethylphosphonate--pyruvate transaminase [Salmonella enterica subsp. enterica serovar Infantis] Overall Protective Antigen Prediction = **0.3654** (Probable **NON-ANTIGEN**).

>EHO9887606.1 phosphonate utilization transcriptional regulator PhnR [Salmonella enterica subsp. enterica serovar Infantis] Overall Protective Antigen Prediction = **0.4660** (Probable **ANTIGEN**).

>EHO9887607.1 2-aminoethylphosphonate ABC transporter substrate-binding protein [Salmonella enterica subsp. enterica serovar Infantis] Overall Protective Antigen Prediction = **0.4845** (Probable **ANTIGEN**).

>EHO9887608.1 2-aminoethylphosphonate ABC transport system ATP-binding subunit PhnT [Salmonella enterica subsp. enterica serovar Infantis] Overall Protective Antigen Prediction = **0.4905** (Probable **ANTIGEN**).

>EHO9887609.1 2-aminoethylphosphonate ABC transporter permease subunit [Salmonella enterica subsp. enterica serovar Infantis] Overall Protective Antigen Prediction = **0.6354** (Probable

ANTIGEN).

>EHO9887610.1 2-aminoethylphosphonate ABC transport system, membrane component PhnV [Salmonella enterica subsp. enterica serovar Infantis] Overall Protective Antigen Prediction = **0.6341** (Probable **ANTIGEN**).

>EHO9887611.1 tRNA 4-thiouridine(8) synthase ThiI [Salmonella enterica subsp. enterica serovar Infantis] Overall Protective Antigen Prediction = **0.5853** (Probable **ANTIGEN**).

>EHO9887612.1 exodeoxyribonuclease VII small subunit [Salmonella enterica subsp. enterica serovar Infantis] Overall Protective Antigen Prediction = **0.2073** (Probable **NON-ANTIGEN**).

>EHO9887613.1 (2E,6E)-farnesyl diphosphate synthase [Salmonella enterica subsp. enterica serovar Infantis] Overall Protective Antigen Prediction = **0.3732** (Probable **NON-ANTIGEN**).

>EHO9887614.1 1-deoxy-D-xylulose-5-phosphate synthase [Salmonella enterica subsp. enterica serovar Infantis] Overall Protective Antigen Prediction = **0.4618** (Probable **ANTIGEN**).

>EHO9887615.1 aldo/keto reductase [Salmonella enterica subsp. enterica serovar Infantis] Overall Protective Antigen Prediction = **0.3044** (Probable **NON-ANTIGEN**).

>EHO9887616.1 phosphatidylglycerophosphatase A [Salmonella enterica subsp. enterica serovar Infantis] Overall Protective Antigen Prediction = **0.6925** (Probable **ANTIGEN**).

>EHO9887617.1 thiamine-phosphate kinase [Salmonella enterica subsp. enterica serovar Infantis] Overall Protective Antigen Prediction = **0.4007** (Probable **ANTIGEN**).

>EHO9887618.1 transcription antitermination factor NusB [Salmonella enterica subsp. enterica serovar Infantis] Overall Protective Antigen Prediction = **0.3807** (Probable **NON-ANTIGEN**).

>EHO9887619.1 6,7-dimethyl-8-ribityllumazine synthase [Salmonella enterica subsp. enterica serovar Infantis] Overall Protective Antigen Prediction = **0.4237** (Probable **ANTIGEN**).

>EHO9887620.1 bifunctional diaminohydroxyphosphoribosylaminopyrimidine deaminase/5-amino-6-(5-phosphoribosylamino)uracil reductase RibD [Salmonella enterica subsp. enterica serovar Infantis] Overall Protective Antigen Prediction = **0.4396** (Probable **ANTIGEN**).

>EHO9887621.1 transcriptional regulator NrdR [Salmonella enterica subsp. enterica serovar Infantis] Overall Protective Antigen Prediction = **0.3525** (Probable **NON-ANTIGEN**).

>EHO9887622.1 DUF3251 domain-containing protein [Salmonella enterica subsp. enterica serovar Infantis] Overall Protective Antigen Prediction = **0.5814** (Probable **ANTIGEN**).

>EHO9887623.1 nucleoside-specific channel-forming protein Tsx [Salmonella enterica subsp. enterica serovar Infantis] Overall Protective Antigen Prediction = **0.5408** (Probable **ANTIGEN**).

>EHO9887624.1 heme ABC exporter ATP-binding protein CcmA, partial [Salmonella enterica subsp. enterica serovar Infantis] Overall Protective Antigen Prediction = **0.4250** (Probable **ANTIGEN**).

>EHO9887625.1 cytochrome-c peroxidase [Salmonella enterica subsp. enterica serovar Infantis] Overall Protective Antigen Prediction = **0.4223** (Probable **ANTIGEN**).

>EHO9887626.1 molecular chaperone TorD [Salmonella enterica subsp. enterica serovar Infantis] Overall Protective Antigen Prediction = **0.4208** (Probable **ANTIGEN**).

>EHO9887627.1 trimethylamine-N-oxide reductase TorA [Salmonella enterica subsp. enterica serovar Infantis] Overall Protective Antigen Prediction = **0.4353** (Probable **ANTIGEN**).

>EHO9887628.1 pentaheme c-type cytochrome TorC [Salmonella enterica subsp. enterica serovar Infantis] Overall Protective Antigen Prediction = **0.5277** (Probable **ANTIGEN**).

>EHO9887629.1 two-component system response regulator TorR [Salmonella enterica subsp. enterica serovar Infantis] Overall Protective Antigen Prediction = **0.5489** (Probable **ANTIGEN**).

>EHO9887630.1 TMAO reductase system periplasmic protein TorT [Salmonella enterica subsp. enterica serovar Infantis] Overall Protective Antigen Prediction = **0.4963** (Probable **ANTIGEN**).

>EHO9887631.1 TMAO reductase system sensor histidine kinase/response regulator TorS [Salmonella enterica subsp. enterica serovar Infantis] Overall Protective Antigen Prediction = **0.4420** (Probable **ANTIGEN**).

>EHO9887632.1 DUF4056 domain-containing protein [Salmonella enterica subsp. enterica serovar Infantis] Overall Protective Antigen Prediction = **0.4520** (Probable **ANTIGEN**).

>EHO9887633.1 BamA/TamA family outer membrane protein [Salmonella enterica subsp. enterica serovar Infantis] Overall Protective Antigen Prediction = **0.5823** (Probable **ANTIGEN**).

>EHO9887634.1 hypothetical protein KND05_003018 [Salmonella enterica subsp. enterica serovar Infantis] Overall Protective Antigen Prediction = **0.6178** (Probable **ANTIGEN**).

>EHO9887635.1 sugar-phosphatase [Salmonella enterica subsp. enterica serovar Infantis] Overall Protective Antigen Prediction = **0.4217** (Probable **ANTIGEN**).

>EHO9887636.1 MFS transporter [Salmonella enterica subsp. enterica serovar Infantis] Overall Protective Antigen Prediction = **0.6177** (Probable **ANTIGEN**).

>EHO9887637.1 mandelate racemase/muconate lactonizing enzyme family protein [Salmonella enterica subsp. enterica serovar Infantis] Overall Protective Antigen Prediction = **0.4897** (Probable **ANTIGEN**).

>EHO9887638.1 LysR family transcriptional regulator [Salmonella enterica subsp. enterica serovar Infantis] Overall Protective Antigen Prediction = **0.3905** (Probable **NON-ANTIGEN**).

>EHO9887639.1 DNA topoisomerase (ATP-hydrolyzing) subunit B [Salmonella enterica subsp. enterica serovar Infantis] Overall Protective Antigen Prediction = **0.6235** (Probable **ANTIGEN**).

>EHO9887640.1 DNA replication/repair protein RecF [Salmonella enterica subsp. enterica serovar Infantis] Overall Protective Antigen Prediction = **0.3817** (Probable **NON-ANTIGEN**).

>EHO9887641.1 DNA polymerase III subunit beta [Salmonella enterica subsp. enterica serovar Infantis] Overall Protective Antigen Prediction = **0.4735** (Probable **ANTIGEN**).

>EHO9887642.1 chromosomal replication initiator protein DnaA [Salmonella enterica subsp. enterica serovar Infantis] Overall Protective Antigen Prediction = **0.3785** (Probable **NON-ANTIGEN**).

>EHO9887643.1 50S ribosomal protein L34 [Salmonella enterica subsp. enterica serovar Infantis] Overall Protective Antigen Prediction = **0.6060** (Probable **ANTIGEN**).

>EHO9887644.1 ribonuclease P protein component [Salmonella enterica subsp. enterica serovar Infantis] Overall Protective Antigen Prediction = **0.4446** (Probable **ANTIGEN**).

>EHO9887645.1 membrane protein insertion efficiency factor YidD [Salmonella enterica subsp. enterica serovar Infantis] Overall Protective Antigen Prediction = **0.2095** (Probable **NON-ANTIGEN**).

>EHO9887646.1 membrane protein insertase YidC [Salmonella enterica subsp. enterica serovar Infantis] Overall Protective Antigen Prediction = **0.6579** (Probable **ANTIGEN**).

>EHO9887647.1 tRNA uridine-5-carboxymethylaminomethyl(34) synthesis GTPase MnmE [Salmonella enterica subsp. enterica serovar Infantis] Overall Protective Antigen Prediction = **0.4403** (Probable **ANTIGEN**).

>EHO9887648.1 MFS transporter [Salmonella enterica subsp. enterica serovar Infantis] Overall Protective Antigen Prediction = **0.4891** (Probable **ANTIGEN**).

>EHO9887649.1 HTH-type transcriptional regulator YidZ [Salmonella enterica subsp. enterica serovar Infantis] Overall Protective Antigen Prediction = **0.5072** (Probable **ANTIGEN**).

>EHO9887650.1 4'-phosphopantetheinyl transferase superfamily protein [Salmonella enterica subsp. enterica serovar Infantis] Overall Protective Antigen Prediction = **0.4158** (Probable **ANTIGEN**).

>EHO9887651.1 NAD(P)H-dependent oxidoreductase [Salmonella enterica subsp. enterica serovar Infantis] Overall Protective Antigen Prediction = **0.3402** (Probable **NON-ANTIGEN**).

>EHO9887652.1 adenine permease AdeP [Salmonella enterica subsp. enterica serovar Infantis] Overall Protective Antigen Prediction = **0.5008** (Probable **ANTIGEN**).

>EHO9887653.1 6-phosphogluconate phosphatase [Salmonella enterica subsp. enterica serovar Infantis] Overall Protective Antigen Prediction = **0.3558** (Probable **NON-ANTIGEN**).

>EHO9887654.1 phosphate signaling complex protein PhoU [Salmonella enterica subsp. enterica serovar Infantis] Overall Protective Antigen Prediction = **0.3951** (Probable **NON-ANTIGEN**).

>EHO9887655.1 phosphate ABC transporter ATP-binding protein PstB [Salmonella enterica subsp. enterica serovar Infantis] Overall Protective Antigen Prediction = **0.3390** (Probable **NON-ANTIGEN**).

>EHO9887656.1 phosphate ABC transporter permease PstA [Salmonella enterica subsp. enterica serovar Infantis] Overall Protective Antigen Prediction = **0.4465** (Probable **ANTIGEN**).

>EHO9887657.1 phosphate ABC transporter permease PstC [Salmonella enterica subsp. enterica serovar Infantis] Overall Protective Antigen Prediction = **0.4181** (Probable **ANTIGEN**).

>EHO9887658.1 phosphate ABC transporter substrate-binding protein PstS [Salmonella enterica subsp. enterica serovar Infantis] Overall Protective Antigen Prediction = **0.5889** (Probable **ANTIGEN**).

>EHO9887659.1 PTS transporter subunit EIIC [Salmonella enterica subsp. enterica serovar Infantis] Overall Protective Antigen Prediction = **0.4914** (Probable **ANTIGEN**).

>EHO9887660.1 shikimate 5-dehydrogenase [Salmonella enterica subsp. enterica serovar Infantis] Overall Protective Antigen Prediction = **0.5058** (Probable **ANTIGEN**).

>EHO9887661.1 SgrR family transcriptional regulator [Salmonella enterica subsp. enterica serovar Infantis] Overall Protective Antigen Prediction = **0.3947** (Probable **NON-ANTIGEN**).

>EHO9887662.1 glutamine--fructose-6-phosphate transaminase (isomerizing) [Salmonella enterica subsp. enterica serovar Infantis] Overall Protective Antigen Prediction = **0.3942** (Probable **NON-ANTIGEN**).

>EHO9887663.1 bifunctional UDP-N-acetylglucosamine diphosphorylase/glucosamine-1-phosphate N-acetyltransferase GlmU [Salmonella enterica subsp. enterica serovar Infantis] Overall Protective Antigen Prediction = **0.4758** (Probable **ANTIGEN**).

>EHO9887664.1 Bax inhibitor-1/YccA family protein [Salmonella enterica subsp. enterica serovar Infantis] Overall Protective Antigen Prediction = **0.3718** (Probable **NON-ANTIGEN**).

>EHO9887665.1 F0F1 ATP synthase subunit epsilon [Salmonella enterica subsp. enterica serovar Infantis] Overall Protective Antigen Prediction = **0.5615** (Probable **ANTIGEN**).

>EHO9887666.1 F0F1 ATP synthase subunit beta [Salmonella enterica subsp. enterica serovar Infantis] Overall Protective Antigen Prediction = **0.3203** (Probable **NON-ANTIGEN**).

>EHO9887667.1 F0F1 ATP synthase subunit gamma [Salmonella enterica subsp. enterica serovar Infantis] Overall Protective Antigen Prediction = **0.3827** (Probable **NON-ANTIGEN**).

>EHO9887668.1 F0F1 ATP synthase subunit alpha [Salmonella enterica subsp. enterica serovar Infantis] Overall Protective Antigen Prediction = **0.3394** (Probable **NON-ANTIGEN**).

>EHO9887669.1 F0F1 ATP synthase subunit delta [Salmonella enterica subsp. enterica serovar Infantis] Overall Protective Antigen Prediction = **0.4807** (Probable **ANTIGEN**).

>EHO9887670.1 F0F1 ATP synthase subunit B [Salmonella enterica subsp. enterica serovar Infantis] Overall Protective Antigen Prediction = **0.7096** (Probable **ANTIGEN**).

>EHO9887671.1 F0F1 ATP synthase subunit C [Salmonella enterica subsp. enterica serovar Infantis] Overall Protective Antigen Prediction = **0.4729** (Probable **ANTIGEN**).

>EHO9887672.1 F0F1 ATP synthase subunit A [Salmonella enterica subsp. enterica serovar Infantis] Overall Protective Antigen Prediction = **0.7214** (Probable **ANTIGEN**).

>EHO9887673.1 F0F1 ATP synthase subunit I [Salmonella enterica subsp. enterica serovar Infantis] Overall Protective Antigen Prediction = **0.7155** (Probable **ANTIGEN**).

>EHO9887674.1 16S rRNA (guanine(527)-N(7))-methyltransferase RsmG [Salmonella enterica subsp. enterica serovar Infantis] Overall Protective Antigen Prediction = **0.2119** (Probable **NON-ANTIGEN**).

>EHO9887675.1 tRNA uridine-5-carboxymethylaminomethyl(34) synthesis enzyme MnmG [Salmonella enterica subsp. enterica serovar Infantis] Overall Protective Antigen Prediction = **0.5969** (Probable **ANTIGEN**).

>EHO9887676.1 FMN-binding protein MioC [Salmonella enterica subsp. enterica serovar Infantis] Overall Protective Antigen Prediction = **0.4738** (Probable **ANTIGEN**).

>EHO9887677.1 transcriptional regulator AsnC [Salmonella enterica subsp. enterica serovar Infantis]

Overall Protective Antigen Prediction = **0.2340** (Probable **NON-ANTIGEN**).

>EHO9887678.1 aspartate--ammonia ligase [Salmonella enterica subsp. enterica serovar Infantis] Overall Protective Antigen Prediction = **0.6004** (Probable **ANTIGEN**).

>EHO9887679.1 ATPase RavA stimulator ViaA [Salmonella enterica subsp. enterica serovar Infantis] Overall Protective Antigen Prediction = **0.3971** (Probable **NON-ANTIGEN**).

>EHO9887680.1 ATPase RavA [Salmonella enterica subsp. enterica serovar Infantis] Overall Protective Antigen Prediction = **0.4088** (Probable **ANTIGEN**).

>EHO9887681.1 low affinity potassium transporter Kup [Salmonella enterica subsp. enterica serovar Infantis] Overall Protective Antigen Prediction = **0.5866** (Probable **ANTIGEN**).

>EHO9887682.1 D-ribose pyranase [Salmonella enterica subsp. enterica serovar Infantis] Overall Protective Antigen Prediction = **0.4143** (Probable **ANTIGEN**).

>EHO9887683.1 ribose ABC transporter ATP-binding protein RbsA [Salmonella enterica subsp. enterica serovar Infantis] Overall Protective Antigen Prediction = **0.3116** (Probable **NON-ANTIGEN**).

>EHO9887684.1 ribose ABC transporter permease [Salmonella enterica subsp. enterica serovar Infantis] Overall Protective Antigen Prediction = **0.3984** (Probable **NON-ANTIGEN**).

>EHO9887685.1 ribose ABC transporter substrate-binding protein RbsB [Salmonella enterica subsp. enterica serovar Infantis] Overall Protective Antigen Prediction = **0.6795** (Probable **ANTIGEN**).

>EHO9887686.1 ribokinase [Salmonella enterica subsp. enterica serovar Infantis] Overall Protective Antigen Prediction = **0.5698** (Probable **ANTIGEN**).

>EHO9887687.1 ribose operon transcriptional repressor RbsR [Salmonella enterica subsp. enterica serovar Infantis] Overall Protective Antigen Prediction = **0.3785** (Probable **NON-ANTIGEN**).

>EHO9887688.1 multidrug transporter subunit MdtD [Salmonella enterica subsp. enterica serovar Infantis] Overall Protective Antigen Prediction = **0.5272** (Probable **ANTIGEN**).

>EHO9887689.1 FadR family transcriptional regulator [Salmonella enterica subsp. enterica serovar Infantis] Overall Protective Antigen Prediction = **0.4360** (Probable **ANTIGEN**).

>EHO9887690.1 5-deoxy-glucuronate isomerase [Salmonella enterica subsp. enterica serovar Infantis] Overall Protective Antigen Prediction = **0.5645** (Probable **ANTIGEN**).

>EHO9887691.1 malonate-semialdehyde dehydrogenase IolA [Salmonella enterica subsp. enterica serovar Infantis] Overall Protective Antigen Prediction = **0.4019** (Probable **ANTIGEN**).

>EHO9887692.1 hypothetical protein KND05_003076 [Salmonella enterica subsp. enterica serovar Infantis] Overall Protective Antigen Prediction = **0.6811** (Probable **ANTIGEN**).

>EHO9887693.1 myo-inositol utilization transcriptional regulator ReiD [Salmonella enterica subsp. enterica serovar Infantis] Overall Protective Antigen Prediction = **0.3813** (Probable **NON-ANTIGEN**).

>EHO9887694.1 myo-inosose-2 dehydratase [Salmonella enterica subsp. enterica serovar Infantis] Overall Protective Antigen Prediction = **0.3253** (Probable **NON-ANTIGEN**).

>EHO9887695.1 inositol 2-dehydrogenase IolG1 [Salmonella enterica subsp. enterica serovar Infantis] Overall Protective Antigen Prediction = **0.5317** (Probable **ANTIGEN**).

>EHO9887696.1 lysosomal glucosyl ceramidase-like type III secretion effector SrfJ [Salmonella enterica subsp. enterica serovar Infantis] Overall Protective Antigen Prediction = **0.4109** (Probable **ANTIGEN**).

>EHO9887697.1 2-keto-myo-inositol isomerase IolI1 [Salmonella enterica subsp. enterica serovar Infantis] Overall Protective Antigen Prediction = **0.2373** (Probable **NON-ANTIGEN**).

>EHO9887698.1 MFS transporter [Salmonella enterica subsp. enterica serovar Infantis] Overall Protective Antigen Prediction = **0.5205** (Probable **ANTIGEN**).

>EHO9887699.1 5-dehydro-2-deoxygluconokinase [Salmonella enterica subsp. enterica serovar Infantis] Overall Protective Antigen Prediction = **0.3361** (Probable **NON-ANTIGEN**).

>EHO9887700.1 3D-(3,5/4)-trihydroxycyclohexane-1,2-dione acylhydrolase (decyclizing) [Salmonella enterica subsp. enterica serovar Infantis] Overall Protective Antigen Prediction = **0.4314** (Probable **ANTIGEN**).

>EHO9887701.1 D-chiro-inositol-2-dehydrogenase IolG2 [Salmonella enterica subsp. enterica serovar Infantis] Overall Protective Antigen Prediction = **0.3579** (Probable **NON-ANTIGEN**).

>EHO9887702.1 MFS transporter [Salmonella enterica subsp. enterica serovar Infantis] Overall Protective Antigen Prediction = **0.4068** (Probable **ANTIGEN**).

>EHO9887703.1 2-keto-myo-inositol isomerase IolI2 [Salmonella enterica subsp. enterica serovar Infantis] Overall Protective Antigen Prediction = **0.3393** (Probable **NON-ANTIGEN**).

>EHO9887704.1 sugar phosphate isomerase/epimerase IolH [Salmonella enterica subsp. enterica serovar Infantis] Overall Protective Antigen Prediction = **0.4435** (Probable **ANTIGEN**).

>EHO9887705.1 ribosome-associated protein [Salmonella enterica subsp. enterica serovar Infantis] Overall Protective Antigen Prediction = **0.1864** (Probable **NON-ANTIGEN**).

>EHO9887706.1 metalloprotease PmbA [Salmonella enterica subsp. enterica serovar Infantis] Overall Protective Antigen Prediction = **0.5542** (Probable **ANTIGEN**).

>EHO9887707.1 cytochrome b562 [Salmonella enterica subsp. enterica serovar Infantis] Overall Protective Antigen Prediction = **0.5769** (Probable **ANTIGEN**).

>EHO9887708.1 glycine dehydrogenase [Salmonella enterica subsp. enterica serovar Infantis] Overall Protective Antigen Prediction = **0.4605** (Probable **ANTIGEN**).

>EHO9887709.1 hypothetical protein KND05_003093 [Salmonella enterica subsp. enterica serovar Infantis] Overall Protective Antigen Prediction = **0.8490** (Probable **ANTIGEN**).

>EHO9887710.1 DUF4312 family protein [Salmonella enterica subsp. enterica serovar Infantis] Overall Protective Antigen Prediction = **0.7841** (Probable **ANTIGEN**).

>EHO9887711.1 DUF4311 domain-containing protein [Salmonella enterica subsp. enterica serovar Infantis] Overall Protective Antigen Prediction = **0.4980** (Probable **ANTIGEN**).

>EHO9887712.1 DUF4310 family protein [Salmonella enterica subsp. enterica serovar Infantis] Overall Protective Antigen Prediction = **0.3711** (Probable **NON-ANTIGEN**).

>EHO9887713.1 amidohydrolase/deacetylase family metallohydrolase [Salmonella enterica subsp. enterica serovar Infantis] Overall Protective Antigen Prediction = **0.4862** (Probable **ANTIGEN**).

>EHO9887714.1 DgaE family pyridoxal phosphate-dependent ammonia lyase [Salmonella enterica subsp. enterica serovar Infantis] Overall Protective Antigen Prediction = **0.3430** (Probable **NON-ANTIGEN**).

>EHO9887715.1 KDGP aldolase family protein [Salmonella enterica subsp. enterica serovar Infantis] Overall Protective Antigen Prediction = **0.4417** (Probable **ANTIGEN**).

>EHO9887716.1 BglG family transcription antiterminator [Salmonella enterica subsp. enterica serovar Infantis] Overall Protective Antigen Prediction = **0.3853** (Probable **NON-ANTIGEN**).

>EHO9887717.1 type II toxin-antitoxin system RelB/DinJ family antitoxin [Salmonella enterica subsp. enterica serovar Infantis] Overall Protective Antigen Prediction = **0.6155** (Probable **ANTIGEN**).

>EHO9887718.1 type II toxin-antitoxin system RelE/ParE family toxin [Salmonella enterica subsp. enterica serovar Infantis] Overall Protective Antigen Prediction = **0.7456** (Probable **ANTIGEN**).

>EHO9887719.1 anaerobic ribonucleoside-triphosphate reductase-activating protein [Salmonella enterica subsp. enterica serovar Infantis] Overall Protective Antigen Prediction = **0.3705** (Probable **NON-ANTIGEN**).

>EHO9887720.1 anaerobic ribonucleoside-triphosphate reductase [Salmonella enterica subsp. enterica serovar Infantis] Overall Protective Antigen Prediction = **0.4702** (Probable **ANTIGEN**).

>EHO9887721.1 alpha,alpha-phosphotrehalase [Salmonella enterica subsp. enterica serovar Infantis] Overall Protective Antigen Prediction = **0.4063** (Probable **ANTIGEN**).

>EHO9887722.1 PTS trehalose transporter subunit IIBC [Salmonella enterica subsp. enterica serovar Infantis] Overall Protective Antigen Prediction = **0.4973** (Probable **ANTIGEN**).

>EHO9887723.1 HTH-type transcriptional regulator TreR [Salmonella enterica subsp. enterica serovar Infantis] Overall Protective Antigen Prediction = **0.4149** (Probable **ANTIGEN**).

>EHO9887724.1 mgtA regulatory leader peptide MgtL [Salmonella enterica subsp. enterica serovar Infantis] Overall Protective Antigen Prediction = **0.7493** (Probable **ANTIGEN**).

>EHO9887725.1 magnesium-translocating P-type ATPase [Salmonella enterica subsp. enterica serovar Infantis] Overall Protective Antigen Prediction = **0.4345** (Probable **ANTIGEN**).

>EHO9887726.1 transposase [Salmonella enterica subsp. enterica serovar Infantis] Overall Protective Antigen Prediction = **0.8138** (Probable **ANTIGEN**).

>EHO9887727.1 2-iminobutanoate/2-iminopropanoate deaminase [Salmonella enterica subsp. enterica serovar Infantis] Overall Protective Antigen Prediction = **0.3438** (Probable **NON-ANTIGEN**).

>EHO9887728.1 aspartate carbamoyltransferase regulatory subunit [Salmonella enterica subsp. enterica serovar Infantis] Overall Protective Antigen Prediction = **0.6126** (Probable **ANTIGEN**).

>EHO9887729.1 aspartate carbamoyltransferase [Salmonella enterica subsp. enterica serovar Infantis] Overall Protective Antigen Prediction = **0.5791** (Probable **ANTIGEN**).

>EHO9887730.1 pyr operon leader peptide [Salmonella enterica subsp. enterica serovar Infantis] Overall Protective Antigen Prediction = **0.3752** (Probable **NON-ANTIGEN**).

>EHO9887731.1 hypothetical protein KND05_003115 [Salmonella enterica subsp. enterica serovar Infantis] Overall Protective Antigen Prediction = **0.3585** (Probable **NON-ANTIGEN**).

>EHO9887732.1 arginine repressor [Salmonella enterica subsp. enterica serovar Infantis] Overall Protective Antigen Prediction = **0.3808** (Probable **NON-ANTIGEN**).

>EHO9887733.1 YfcC family protein [Salmonella enterica subsp. enterica serovar Infantis] Overall Protective Antigen Prediction = **0.3698** (Probable **NON-ANTIGEN**).

>EHO9887734.1 ornithine carbamoyltransferase [Salmonella enterica subsp. enterica serovar Infantis] Overall Protective Antigen Prediction = **0.4503** (Probable **ANTIGEN**).

>EHO9887735.1 carbamate kinase [Salmonella enterica subsp. enterica serovar Infantis] Overall Protective Antigen Prediction = **0.3574** (Probable **NON-ANTIGEN**).

>EHO9887736.1 arginine deiminase [Salmonella enterica subsp. enterica serovar Infantis] Overall Protective Antigen Prediction = **0.4621** (Probable **ANTIGEN**).

>EHO9887737.1 YhcH/YjgK/YiaL family protein [Salmonella enterica subsp. enterica serovar Infantis] Overall Protective Antigen Prediction = **0.3795** (Probable **NON-ANTIGEN**).

>EHO9887738.1 ornithine carbamoyltransferase [Salmonella enterica subsp. enterica serovar Infantis] Overall Protective Antigen Prediction = **0.5037** (Probable **ANTIGEN**).

>EHO9887739.1 ribonuclease E inhibitor RraB [Salmonella enterica subsp. enterica serovar Infantis] Overall Protective Antigen Prediction = **0.6597** (Probable **ANTIGEN**).

>EHO9887740.1 tRNA isopentenyl-2-thiomethyl-A-37 hydroxylase MiaE [Salmonella enterica subsp. enterica serovar Infantis] Overall Protective Antigen Prediction = **0.2776** (Probable **NON-ANTIGEN**).

>EHO9887741.1 DUF3617 domain-containing protein [Salmonella enterica subsp. enterica serovar Infantis] Overall Protective Antigen Prediction = **0.8322** (Probable **ANTIGEN**).

>EHO9887742.1 GNAT family N-acetyltransferase [Salmonella enterica subsp. enterica serovar Infantis] Overall Protective Antigen Prediction = **0.3188** (Probable **NON-ANTIGEN**).

>EHO9887743.1 DUF898 domain-containing protein [Salmonella enterica subsp. enterica serovar Infantis] Overall Protective Antigen Prediction = **0.6915** (Probable **ANTIGEN**).

>EHO9887744.1 valine--tRNA ligase [Salmonella enterica subsp. enterica serovar Infantis] Overall Protective Antigen Prediction = **0.4687** (Probable **ANTIGEN**).

>EHO9887745.1 DNA polymerase III subunit chi [Salmonella enterica subsp. enterica serovar Infantis] Overall Protective Antigen Prediction = **0.3947** (Probable **NON-ANTIGEN**).

>EHO9887746.1 leucyl aminopeptidase [Salmonella enterica subsp. enterica serovar Infantis] Overall

Protective Antigen Prediction = **0.4919** (Probable **ANTIGEN**).

>EHO9887747.1 LPS export ABC transporter permease LptF [Salmonella enterica subsp. enterica serovar Infantis] Overall Protective Antigen Prediction = **0.6610** (Probable **ANTIGEN**).

>EHO9887748.1 LPS export ABC transporter permease LptG [Salmonella enterica subsp. enterica serovar Infantis] Overall Protective Antigen Prediction = **0.3473** (Probable **NON-ANTIGEN**).

>EHO9887749.1 LacI family DNA-binding transcriptional regulator [Salmonella enterica subsp. enterica serovar Infantis] Overall Protective Antigen Prediction = **0.3714** (Probable **NON-ANTIGEN**).

>EHO9887750.1 gnt-II system L-idonate transporter [Salmonella enterica subsp. enterica serovar Infantis] Overall Protective Antigen Prediction = **0.4292** (Probable **ANTIGEN**).

>EHO9887751.1 gluconate 5-dehydrogenase [Salmonella enterica subsp. enterica serovar Infantis] Overall Protective Antigen Prediction = **0.2583** (Probable **NON-ANTIGEN**).

>EHO9887752.1 L-idonate 5-dehydrogenase [Salmonella enterica subsp. enterica serovar Infantis] Overall Protective Antigen Prediction = **0.4633** (Probable **ANTIGEN**).

>EHO9887753.1 gluconokinase [Salmonella enterica subsp. enterica serovar Infantis] Overall Protective Antigen Prediction = **0.2851** (Probable **NON-ANTIGEN**).

>EHO9887754.1 NAD(P)-dependent alcohol dehydrogenase [Salmonella enterica subsp. enterica serovar Infantis] Overall Protective Antigen Prediction = **0.6487** (Probable **ANTIGEN**).

>EHO9887755.1 SinI family restriction endonuclease [Salmonella enterica subsp. enterica serovar Infantis] Overall Protective Antigen Prediction = **0.4242** (Probable **ANTIGEN**).

>EHO9887756.1 DNA cytosine methyltransferase [Salmonella enterica subsp. enterica serovar Infantis] Overall Protective Antigen Prediction = **0.3200** (Probable **NON-ANTIGEN**).

>EHO9887757.1 colanic acid biosynthesis phosphomannomutase CpsG, partial [Salmonella enterica subsp. enterica serovar Infantis] Overall Protective Antigen Prediction = **0.5631** (Probable **ANTIGEN**).

>EHO9887758.1 hypothetical protein KND05_003144 [Salmonella enterica subsp. enterica serovar Infantis] Overall Protective Antigen Prediction = **0.4144** (Probable **ANTIGEN**).

>EHO9887759.1 NADP-dependent phosphogluconate dehydrogenase [Salmonella enterica subsp. enterica serovar Infantis] Overall Protective Antigen Prediction = **0.2592** (Probable **NON-ANTIGEN**).

>EHO9887760.1 UDP-glucose 6-dehydrogenase [Salmonella enterica subsp. enterica serovar Infantis] Overall Protective Antigen Prediction = **0.3490** (Probable **NON-ANTIGEN**).

>EHO9887761.1 LPS O-antigen chain length determinant protein WzzB [Salmonella enterica subsp. enterica serovar Infantis] Overall Protective Antigen Prediction = **0.5556** (Probable **ANTIGEN**).

>EHO9887762.1 bifunctional phosphoribosyl-AMP cyclohydrolase/phosphoribosyl-ATP diphosphatase HisIE [Salmonella enterica subsp. enterica serovar Infantis] Overall Protective Antigen Prediction = **0.4491** (Probable **ANTIGEN**).

>EHO9887763.1 imidazole glycerol phosphate synthase subunit HisF [Salmonella enterica subsp. enterica serovar Infantis] Overall Protective Antigen Prediction = **0.5463** (Probable **ANTIGEN**).

>EHO9887764.1 1-(5-phosphoribosyl)-5-[(5-phosphoribosylamino)methylideneamino]imidazole-4-carboxamide isomerase [Salmonella enterica subsp. enterica serovar Infantis] Overall Protective Antigen Prediction = **0.5411** (Probable **ANTIGEN**).

>EHO9887765.1 imidazole glycerol phosphate synthase subunit HisH [Salmonella enterica subsp. enterica serovar Infantis] Overall Protective Antigen Prediction = **0.4630** (Probable **ANTIGEN**).

>EHO9887766.1 bifunctional histidinol-phosphatase/imidazoleglycerol-phosphate dehydratase HisB [Salmonella enterica subsp. enterica serovar Infantis] Overall Protective Antigen Prediction = **0.4930** (Probable **ANTIGEN**).

>EHO9887767.1 histidinol-phosphate transaminase [Salmonella enterica subsp. enterica serovar Infantis] Overall Protective Antigen Prediction = **0.3997** (Probable **NON-ANTIGEN**).

>EHO9887768.1 histidinol dehydrogenase [Salmonella enterica subsp. enterica serovar Infantis] Overall Protective Antigen Prediction = **0.4006** (Probable **ANTIGEN**).

>EHO9887769.1 ATP phosphoribosyltransferase [Salmonella enterica subsp. enterica serovar Infantis] Overall Protective Antigen Prediction = **0.3758** (Probable **NON-ANTIGEN**).

>EHO9887770.1 his operon leader peptide [Salmonella enterica subsp. enterica serovar Infantis] Overall Protective Antigen Prediction = **0.5498** (Probable **ANTIGEN**).

>EHO9887771.1 SDR family oxidoreductase [Salmonella enterica subsp. enterica serovar Infantis] Overall Protective Antigen Prediction = **0.4770** (Probable **ANTIGEN**).

>EHO9887772.1 LysR family transcriptional regulator [Salmonella enterica subsp. enterica serovar Infantis] Overall Protective Antigen Prediction = **0.3033** (Probable **NON-ANTIGEN**).

>EHO9887773.1 membrane protein YoeI [Salmonella enterica subsp. enterica serovar Infantis] Overall Protective Antigen Prediction = **0.1403** (Probable **NON-ANTIGEN**).

>EHO9887774.1 putrescine/proton symporter PlaP [Salmonella enterica subsp. enterica serovar Infantis] Overall Protective Antigen Prediction = **0.4600** (Probable **ANTIGEN**).

>EHO9887775.1 exodeoxyribonuclease I [Salmonella enterica subsp. enterica serovar Infantis] Overall Protective Antigen Prediction = **0.3642** (Probable **NON-ANTIGEN**).

>EHO9887776.1 SPI-1 type III secretion system effector HECT-type E3 ubiquitin transferase SopA [Salmonella enterica subsp. enterica serovar Infantis] Overall Protective Antigen Prediction = **0.4319** (Probable **ANTIGEN**).

>EHO9887777.1 thiosulfate reductase PhsA [Salmonella enterica subsp. enterica serovar Infantis] Overall Protective Antigen Prediction = **0.4742** (Probable **ANTIGEN**).

>EHO9887778.1 thiosulfate reductase electron transport protein PhsB [Salmonella enterica subsp. enterica serovar Infantis] Overall Protective Antigen Prediction = **0.5077** (Probable **ANTIGEN**).

>EHO9887779.1 thiosulfate reductase cytochrome B subunit [Salmonella enterica subsp. enterica serovar Infantis] Overall Protective Antigen Prediction = **0.5891** (Probable **ANTIGEN**).

>EHO9887780.1 serine-type D-Ala-D-Ala carboxypeptidase DacD [Salmonella enterica subsp. enterica serovar Infantis] Overall Protective Antigen Prediction = **0.4257** (Probable **ANTIGEN**).

>EHO9887781.1 DNA gyrase inhibitor SbmC [Salmonella enterica subsp. enterica serovar Infantis] Overall Protective Antigen Prediction = **0.2917** (Probable **NON-ANTIGEN**).

>EHO9887782.1 FUSC family protein [Salmonella enterica subsp. enterica serovar Infantis] Overall Protective Antigen Prediction = **0.4143** (Probable **ANTIGEN**).

>EHO9887783.1 DUF496 family protein [Salmonella enterica subsp. enterica serovar Infantis] Overall Protective Antigen Prediction = **0.2996** (Probable **NON-ANTIGEN**).

>EHO9887784.1 L-threonine kinase PduX [Salmonella enterica subsp. enterica serovar Infantis] Overall Protective Antigen Prediction = **0.3723** (Probable **NON-ANTIGEN**).

>EHO9887785.1 propanediol utilization propionate kinase PduW [Salmonella enterica subsp. enterica serovar Infantis] Overall Protective Antigen Prediction = **0.5025** (Probable **ANTIGEN**).

>EHO9887786.1 propanediol utilization protein PduV [Salmonella enterica subsp. enterica serovar Infantis] Overall Protective Antigen Prediction = **0.3310** (Probable **NON-ANTIGEN**).

>EHO9887787.1 propanediol utilization microcompartment protein PduU [Salmonella enterica subsp. enterica serovar Infantis] Overall Protective Antigen Prediction = **0.2242** (Probable **NON-ANTIGEN**).

>EHO9887788.1 propanediol utilization microcompartment protein PduT [Salmonella enterica subsp. enterica serovar Infantis] Overall Protective Antigen Prediction = **0.4521** (Probable **ANTIGEN**).

>EHO9887789.1 cobalamin reductase PduS [Salmonella enterica subsp. enterica serovar Infantis] Overall Protective Antigen Prediction = **0.4117** (Probable **ANTIGEN**).

>EHO9887790.1 1-propanol dehydrogenase PduQ [Salmonella enterica subsp. enterica serovar Infantis] Overall Protective Antigen Prediction = **0.4735** (Probable **ANTIGEN**).

>EHO9887791.1 CoA-acylating propionaldehyde dehydrogenase PduP [Salmonella enterica subsp. enterica serovar Infantis] Overall Protective Antigen Prediction = **0.4352** (Probable **ANTIGEN**).

>EHO9887792.1 two-domain cob(I)yrinic acid a,c-diamide adenosyltransferase PduO [Salmonella enterica subsp. enterica serovar Infantis] Overall Protective Antigen Prediction = **0.4717** (Probable **ANTIGEN**).

>EHO9887793.1 propanediol utilization microcompartment protein PduN [Salmonella enterica subsp. enterica serovar Infantis] Overall Protective Antigen Prediction = **0.5308** (Probable **ANTIGEN**).

>EHO9887794.1 propanediol utilization microcompartment protein PduM [Salmonella enterica subsp. enterica serovar Infantis] Overall Protective Antigen Prediction = **0.4780** (Probable **ANTIGEN**).

>EHO9887795.1 phosphate propanoyltransferase [Salmonella enterica subsp. enterica serovar Infantis] Overall Protective Antigen Prediction = **0.4946** (Probable **ANTIGEN**).

>EHO9887796.1 propanediol utilization microcompartment protein PduK [Salmonella enterica subsp. enterica serovar Infantis] Overall Protective Antigen Prediction = **0.6490** (Probable **ANTIGEN**).

>EHO9887797.1 propanediol utilization microcompartment protein PduJ [Salmonella enterica subsp.

enterica serovar Infantis] Overall Protective Antigen Prediction = **0.4764** (Probable **ANTIGEN**).

>EHO9887798.1 propanediol dehydratase reactivase beta subunit PduH [Salmonella enterica subsp. enterica serovar Infantis] Overall Protective Antigen Prediction = **0.4045** (Probable **ANTIGEN**).

>EHO9887799.1 propanediol dehydratase reactivase alpha subunit PduG [Salmonella enterica subsp. enterica serovar Infantis] Overall Protective Antigen Prediction = **0.4907** (Probable **ANTIGEN**).

>EHO9887800.1 propanediol dehydratase small subunit PduE [Salmonella enterica subsp. enterica serovar Infantis] Overall Protective Antigen Prediction = **0.6213** (Probable **ANTIGEN**).

>EHO9887801.1 propanediol dehydratase medium subunit PduD [Salmonella enterica subsp. enterica serovar Infantis] Overall Protective Antigen Prediction = **0.5143** (Probable **ANTIGEN**).

>EHO9887802.1 propanediol dehydratase large subunit PduC [Salmonella enterica subsp. enterica serovar Infantis] Overall Protective Antigen Prediction = **0.4599** (Probable **ANTIGEN**).

>EHO9887803.1 propanediol utilization microcompartment protein PduB [Salmonella enterica subsp. enterica serovar Infantis] Overall Protective Antigen Prediction = **0.5039** (Probable **ANTIGEN**).

>EHO9887804.1 propanediol utilization microcompartment protein PduA [Salmonella enterica subsp. enterica serovar Infantis] Overall Protective Antigen Prediction = **0.4617** (Probable **ANTIGEN**).

>EHO9887805.1 propanediol diffusion facilitator PduF [Salmonella enterica subsp. enterica serovar Infantis] Overall Protective Antigen Prediction = **0.4542** (Probable **ANTIGEN**).

>EHO9887806.1 regulatory protein PocR [Salmonella enterica subsp. enterica serovar Infantis] Overall Protective Antigen Prediction = **0.4405** (Probable **ANTIGEN**).

>EHO9887807.1 cobyrinate a,c-diamide synthase [Salmonella enterica subsp. enterica serovar Infantis] Overall Protective Antigen Prediction = **0.4778** (Probable **ANTIGEN**).

>EHO9887808.1 cobalamin biosynthesis protein [Salmonella enterica subsp. enterica serovar Infantis] Overall Protective Antigen Prediction = **0.4485** (Probable **ANTIGEN**).

>EHO9887809.1 cobalt-precorrin-8 methylmutase [Salmonella enterica subsp. enterica serovar Infantis] Overall Protective Antigen Prediction = **0.3437** (Probable **NON-ANTIGEN**).

>EHO9887810.1 cobalt-precorrin-5B (C(1))-methyltransferase [Salmonella enterica subsp. enterica serovar Infantis] Overall Protective Antigen Prediction = **0.4934** (Probable **ANTIGEN**).

>EHO9887811.1 cobalt-precorrin-7 (C(5))-methyltransferase [Salmonella enterica subsp. enterica serovar Infantis] Overall Protective Antigen Prediction = **0.3498** (Probable **NON-ANTIGEN**).

>EHO9887812.1 decarboxylating cobalt-precorrin-6B (C(15))-methyltransferase [Salmonella enterica subsp. enterica serovar Infantis] Overall Protective Antigen Prediction = **0.4836** (Probable **ANTIGEN**).

>EHO9887813.1 cobalt-precorrin-4 methyltransferase [Salmonella enterica subsp. enterica serovar Infantis] Overall Protective Antigen Prediction = **0.3803** (Probable **NON-ANTIGEN**).

>EHO9887814.1 cobalt-precorrin 5A hydrolase [Salmonella enterica subsp. enterica serovar Infantis] Overall Protective Antigen Prediction = **0.4167** (Probable **ANTIGEN**).

>EHO9887815.1 precorrin-3B C(17)-methyltransferase [Salmonella enterica subsp. enterica serovar Infantis] Overall Protective Antigen Prediction = **0.3870** (Probable **NON-ANTIGEN**).

>EHO9887816.1 cobalt-precorrin-6A reductase [Salmonella enterica subsp. enterica serovar Infantis] Overall Protective Antigen Prediction = **0.2897** (Probable **NON-ANTIGEN**).

>EHO9887817.1 sirohydrochlorin cobaltochelatase [Salmonella enterica subsp. enterica serovar Infantis] Overall Protective Antigen Prediction = **0.3849** (Probable **NON-ANTIGEN**).

>EHO9887818.1 cobalt-factor II C(20)-methyltransferase [Salmonella enterica subsp. enterica serovar Infantis] Overall Protective Antigen Prediction = **0.4155** (Probable **ANTIGEN**).

>EHO9887819.1 cobalt ECF transporter S component CbiM [Salmonella enterica subsp. enterica serovar Infantis] Overall Protective Antigen Prediction = **0.5813** (Probable **ANTIGEN**).

>EHO9887820.1 energy-coupling factor ABC transporter substrate-binding protein [Salmonella enterica subsp. enterica serovar Infantis] Overall Protective Antigen Prediction = **0.9212** (Probable **ANTIGEN**).

>EHO9887821.1 energy-coupling factor ABC transporter transmembrane protein [Salmonella enterica subsp. enterica serovar Infantis] Overall Protective Antigen Prediction = **0.4885** (Probable **ANTIGEN**).

>EHO9887822.1 energy-coupling factor ABC transporter ATP-binding protein [Salmonella enterica subsp. enterica serovar Infantis] Overall Protective Antigen Prediction = **0.2170** (Probable **NON-ANTIGEN**).

>EHO9887823.1 cobyric acid synthase [Salmonella enterica subsp. enterica serovar Infantis] Overall Protective Antigen Prediction = **0.3993** (Probable **NON-ANTIGEN**).

>EHO9887824.1 bifunctional adenosylcobinamide kinase/adenosylcobinamide-phosphate guanylyltransferase [Salmonella enterica subsp. enterica serovar Infantis] Overall Protective Antigen Prediction = **0.5188** (Probable **ANTIGEN**).

>EHO9887825.1 adenosylcobinamide-GDP ribazoletransferase [Salmonella enterica subsp. enterica serovar Infantis] Overall Protective Antigen Prediction = **0.5492** (Probable **ANTIGEN**).

>EHO9887826.1 nicotinate-nucleotide--dimethylbenzimidazole phosphoribosyltransferase [Salmonella enterica subsp. enterica serovar Infantis] Overall Protective Antigen Prediction = **0.4947** (Probable **ANTIGEN**).

>EHO9887827.1 L,D-transpeptidase [Salmonella enterica subsp. enterica serovar Infantis] Overall Protective Antigen Prediction = **0.5759** (Probable **ANTIGEN**).

>EHO9887828.1 EmmdR/YeeO family multidrug/toxin efflux MATE transporter [Salmonella enterica subsp. enterica serovar Infantis] Overall Protective Antigen Prediction = **0.4818** (Probable **ANTIGEN**).

>EHO9887829.1 chitoporin [Salmonella enterica subsp. enterica serovar Infantis] Overall Protective Antigen Prediction = **0.5911** (Probable **ANTIGEN**).

>EHO9887830.1 lipoprotein [Salmonella enterica subsp. enterica serovar Infantis] Overall Protective Antigen Prediction = **0.6439** (Probable **ANTIGEN**).

>EHO9887831.1 tricarballylate/proton symporter TcuC [Salmonella enterica subsp. enterica serovar Infantis] Overall Protective Antigen Prediction = **0.4689** (Probable **ANTIGEN**).

>EHO9887832.1 tricarballylate utilization protein TcuB [Salmonella enterica subsp. enterica serovar Infantis] Overall Protective Antigen Prediction = **0.4643** (Probable **ANTIGEN**).

>EHO9887833.1 FAD-dependent tricarballylate dehydrogenase TcuA [Salmonella enterica subsp. enterica serovar Infantis] Overall Protective Antigen Prediction = **0.4853** (Probable **ANTIGEN**).

>EHO9887834.1 tricarballylate utilization LysR family transcriptional regulator TcuR [Salmonella enterica subsp. enterica serovar Infantis] Overall Protective Antigen Prediction = **0.4523** (Probable **ANTIGEN**).

>EHO9887835.1 ferric iron uptake transcriptional regulator [Salmonella enterica subsp. enterica serovar Infantis] Overall Protective Antigen Prediction = **0.5819** (Probable **ANTIGEN**).

>EHO9887836.1 flavodoxin FldA [Salmonella enterica subsp. enterica serovar Infantis] Overall Protective Antigen Prediction = **0.2516** (Probable **NON-ANTIGEN**).

>EHO9887837.1 LexA regulated protein [Salmonella enterica subsp. enterica serovar Infantis] Overall Protective Antigen Prediction = **0.3219** (Probable **NON-ANTIGEN**).

>EHO9887838.1 esterase [Salmonella enterica subsp. enterica serovar Infantis] Overall Protective Antigen Prediction = **0.3772** (Probable **NON-ANTIGEN**).

>EHO9887839.1 replication initiation negative regulator SeqA [Salmonella enterica subsp. enterica serovar Infantis] Overall Protective Antigen Prediction = **0.4065** (Probable **ANTIGEN**).

>EHO9887840.1 alpha-D-glucose phosphate-specific phosphoglucomutase [Salmonella enterica subsp. enterica serovar Infantis] Overall Protective Antigen Prediction = **0.5188** (Probable **ANTIGEN**).

>EHO9887841.1 pyridoxamine 5'-phosphate oxidase family protein [Salmonella enterica subsp. enterica serovar Infantis] Overall Protective Antigen Prediction = **0.4678** (Probable **ANTIGEN**).

>EHO9887842.1 putrescine-ornithine antiporter [Salmonella enterica subsp. enterica serovar Infantis] Overall Protective Antigen Prediction = **0.4867** (Probable **ANTIGEN**).

>EHO9887843.1 ornithine decarboxylase SpeF [Salmonella enterica subsp. enterica serovar Infantis] Overall Protective Antigen Prediction = **0.3574** (Probable **NON-ANTIGEN**).

>EHO9887844.1 DUF2618 domain-containing protein [Salmonella enterica subsp. enterica serovar Infantis] Overall Protective Antigen Prediction = **0.4226** (Probable **ANTIGEN**).

>EHO9887845.1 two-component system response regulator KdpE [Salmonella enterica subsp. enterica serovar Infantis] Overall Protective Antigen Prediction = **0.4044** (Probable **ANTIGEN**).

>EHO9887846.1 two-component system sensor histidine kinase KdpD [Salmonella enterica subsp. enterica serovar Infantis] Overall Protective Antigen Prediction = **0.3873** (Probable **NON-ANTIGEN**).

>EHO9887847.1 potassium-transporting ATPase subunit KdpC [Salmonella enterica subsp. enterica serovar Infantis] Overall Protective Antigen Prediction = **0.5922** (Probable **ANTIGEN**).

>EHO9887848.1 potassium-transporting ATPase subunit KdpB [Salmonella enterica subsp. enterica serovar Infantis] Overall Protective Antigen Prediction = **0.4984** (Probable **ANTIGEN**).

>EHO9887849.1 potassium-transporting ATPase subunit KdpA [Salmonella enterica subsp. enterica serovar Infantis] Overall Protective Antigen Prediction = **0.5176** (Probable **ANTIGEN**).

>EHO9887850.1 K(+)-transporting ATPase subunit F [Salmonella enterica subsp. enterica serovar Infantis] Overall Protective Antigen Prediction = **0.5208** (Probable **ANTIGEN**).

>EHO9887851.1 YbfA family protein [Salmonella enterica subsp. enterica serovar Infantis] Overall Protective Antigen Prediction = **0.2199** (Probable **NON-ANTIGEN**).

>EHO9887852.1 deoxyribodipyrimidine photo-lyase [Salmonella enterica subsp. enterica serovar Infantis] Overall Protective Antigen Prediction = **0.4049** (Probable **ANTIGEN**).

>EHO9887853.1 MFS transporter [Salmonella enterica subsp. enterica serovar Infantis] Overall Protective Antigen Prediction = **0.4706** (Probable **ANTIGEN**).

>EHO9887854.1 radiation resistance protein YbgI [Salmonella enterica subsp. enterica serovar Infantis] Overall Protective Antigen Prediction = **0.4868** (Probable **ANTIGEN**).

>EHO9887855.1 5-oxoprolinase subunit PxpB [Salmonella enterica subsp. enterica serovar Infantis] Overall Protective Antigen Prediction = **0.3265** (Probable **NON-ANTIGEN**).

>EHO9887856.1 biotin-dependent carboxyltransferase family protein [Salmonella enterica subsp. enterica serovar Infantis] Overall Protective Antigen Prediction = **0.5142** (Probable **ANTIGEN**).

>EHO9887857.1 5-oxoprolinase subunit PxpA [Salmonella enterica subsp. enterica serovar Infantis] Overall Protective Antigen Prediction = **0.3759** (Probable **NON-ANTIGEN**).

>EHO9887858.1 endonuclease VIII [Salmonella enterica subsp. enterica serovar Infantis] Overall Protective Antigen Prediction = **0.4651** (Probable **ANTIGEN**).

>EHO9887859.1 AbrB family transcriptional regulator [Salmonella enterica subsp. enterica serovar Infantis] Overall Protective Antigen Prediction = **0.6661** (Probable **ANTIGEN**).

>EHO9887860.1 citrate synthase [Salmonella enterica subsp. enterica serovar Infantis] Overall Protective Antigen Prediction = **0.3892** (Probable **NON-ANTIGEN**).

>EHO9887861.1 hypothetical protein KND05_003250 [Salmonella enterica subsp. enterica serovar Infantis] Overall Protective Antigen Prediction = **0.5224** (Probable **ANTIGEN**).

>EHO9887862.1 succinate dehydrogenase cytochrome b556 subunit [Salmonella enterica subsp. enterica serovar Infantis] Overall Protective Antigen Prediction = **0.4532** (Probable **ANTIGEN**).

>EHO9887863.1 succinate dehydrogenase membrane anchor subunit [Salmonella enterica subsp. enterica serovar Infantis] Overall Protective Antigen Prediction = **0.3635** (Probable **NON-ANTIGEN**).

>EHO9887864.1 succinate dehydrogenase flavoprotein subunit [Salmonella enterica subsp. enterica serovar Infantis] Overall Protective Antigen Prediction = **0.6372** (Probable **ANTIGEN**).

>EHO9887865.1 succinate dehydrogenase iron-sulfur subunit SdhB [Salmonella enterica subsp. enterica serovar Infantis] Overall Protective Antigen Prediction = **0.4967** (Probable **ANTIGEN**).

>EHO9887866.1 2-oxoglutarate dehydrogenase E1 component [Salmonella enterica subsp. enterica serovar Infantis] Overall Protective Antigen Prediction = **0.4240** (Probable **ANTIGEN**).

>EHO9887867.1 2-oxoglutarate dehydrogenase complex dihydrolipoyllysine-residue succinyltransferase [Salmonella enterica subsp. enterica serovar Infantis] Overall Protective Antigen Prediction = **0.6274** (Probable **ANTIGEN**).

>EHO9887868.1 ADP-forming succinate--CoA ligase subunit beta [Salmonella enterica subsp. enterica serovar Infantis] Overall Protective Antigen Prediction = **0.5957** (Probable **ANTIGEN**).

>EHO9887869.1 succinate--CoA ligase subunit alpha [Salmonella enterica subsp. enterica serovar Infantis] Overall Protective Antigen Prediction = **0.3431** (Probable **NON-ANTIGEN**).

>EHO9887870.1 cytochrome ubiquinol oxidase subunit I [Salmonella enterica subsp. enterica serovar Infantis] Overall Protective Antigen Prediction = **0.5373** (Probable **ANTIGEN**).

>EHO9887871.1 cytochrome d ubiquinol oxidase subunit II [Salmonella enterica subsp. enterica serovar Infantis] Overall Protective Antigen Prediction = **0.5057** (Probable **ANTIGEN**).

>EHO9887872.1 cytochrome bd-I oxidase subunit CydX [Salmonella enterica subsp. enterica serovar Infantis] Overall Protective Antigen Prediction = **1.1323** (Probable **ANTIGEN**).

>EHO9887873.1 cyd operon protein YbgE [Salmonella enterica subsp. enterica serovar Infantis] Overall Protective Antigen Prediction = **0.6197** (Probable **ANTIGEN**).

>EHO9887874.1 tol-pal system-associated acyl-CoA thioesterase [Salmonella enterica subsp. enterica serovar Infantis] Overall Protective Antigen Prediction = **0.3840** (Probable **NON-ANTIGEN**).

>EHO9887875.1 Tol-Pal system protein TolQ [Salmonella enterica subsp. enterica serovar Infantis] Overall Protective Antigen Prediction = **0.4034** (Probable **ANTIGEN**).

>EHO9887876.1 colicin uptake protein TolR [Salmonella enterica subsp. enterica serovar Infantis] Overall Protective Antigen Prediction = **0.4838** (Probable **ANTIGEN**).

>EHO9887877.1 cell envelope integrity protein TolA [Salmonella enterica subsp. enterica serovar Infantis] Overall Protective Antigen Prediction = **1.1164** (Probable **ANTIGEN**).

>EHO9887878.1 Tol-Pal system protein TolB [Salmonella enterica subsp. enterica serovar Infantis] Overall Protective Antigen Prediction = **0.5828** (Probable **ANTIGEN**).

>EHO9887879.1 peptidoglycan-associated lipoprotein Pal [Salmonella enterica subsp. enterica serovar Infantis] Overall Protective Antigen Prediction = **0.8968** (Probable **ANTIGEN**).

>EHO9887880.1 cell division protein CpoB [Salmonella enterica subsp. enterica serovar Infantis] Overall Protective Antigen Prediction = **0.5531** (Probable **ANTIGEN**).

>EHO9887881.1 quinolinate synthase NadA [Salmonella enterica subsp. enterica serovar Infantis] Overall Protective Antigen Prediction = **0.2565** (Probable **NON-ANTIGEN**).

>EHO9887882.1 nicotinamide riboside transporter PnuC [Salmonella enterica subsp. enterica serovar Infantis] Overall Protective Antigen Prediction = **0.6192** (Probable **ANTIGEN**).

>EHO9887883.1 CDF family zinc transporter ZitB [Salmonella enterica subsp. enterica serovar

Infantis] Overall Protective Antigen Prediction = **0.4287** (Probable **ANTIGEN**).

>EHO9887884.1 YbgS-like family protein [Salmonella enterica subsp. enterica serovar Infantis] Overall Protective Antigen Prediction = **1.2105** (Probable **ANTIGEN**).

>EHO9887885.1 3-deoxy-7-phosphoheptulonate synthase AroG [Salmonella enterica subsp. enterica serovar Infantis] Overall Protective Antigen Prediction = **0.4653** (Probable **ANTIGEN**).

>EHO9887886.1 fumarate hydratase subunit beta [Salmonella enterica subsp. enterica serovar Infantis] Overall Protective Antigen Prediction = **0.4407** (Probable **ANTIGEN**).

>EHO9887887.1 fumarate hydratase [Salmonella enterica subsp. enterica serovar Infantis] Overall Protective Antigen Prediction = **0.3865** (Probable **NON-ANTIGEN**).

>EHO9887888.1 LysR family transcriptional regulator [Salmonella enterica subsp. enterica serovar Infantis] Overall Protective Antigen Prediction = **0.3311** (Probable **NON-ANTIGEN**).

>EHO9887889.1 LysR family transcriptional regulator [Salmonella enterica subsp. enterica serovar Infantis] Overall Protective Antigen Prediction = **0.4204** (Probable **ANTIGEN**).

>EHO9887890.1 SLC13/DASS family transporter [Salmonella enterica subsp. enterica serovar Infantis] Overall Protective Antigen Prediction = **0.5603** (Probable **ANTIGEN**).

>EHO9887891.1 oxaloacetate decarboxylase subunit gamma [Salmonella enterica subsp. enterica serovar Infantis] Overall Protective Antigen Prediction = **0.7648** (Probable **ANTIGEN**).

>EHO9887892.1 amidohydrolase family protein, partial [Salmonella enterica subsp. enterica serovar Infantis] Overall Protective Antigen Prediction = **0.3832** (Probable **NON-ANTIGEN**).

>EHO9887893.1 class I SAM-dependent methyltransferase [Salmonella enterica subsp. enterica serovar Infantis] Overall Protective Antigen Prediction = **0.2713** (Probable **NON-ANTIGEN**).

>EHO9887894.1 YjhX family toxin [Salmonella enterica subsp. enterica serovar Infantis] Overall Protective Antigen Prediction = **0.5472** (Probable **ANTIGEN**).

>EHO9887895.1 DUF1062 domain-containing protein [Salmonella enterica subsp. enterica serovar Infantis] Overall Protective Antigen Prediction = **0.4961** (Probable **ANTIGEN**).

>EHO9887896.1 hypothetical protein KND05_003290 [Salmonella enterica subsp. enterica serovar Infantis] Overall Protective Antigen Prediction = **0.3529** (Probable **NON-ANTIGEN**).

>EHO9887897.1 DUF302 domain-containing protein [Salmonella enterica subsp. enterica serovar Infantis] Overall Protective Antigen Prediction = **0.5338** (Probable **ANTIGEN**).

>EHO9887898.1 dienelactone hydrolase family protein [Salmonella enterica subsp. enterica serovar Infantis] Overall Protective Antigen Prediction = **0.5840** (Probable **ANTIGEN**).

>EHO9887899.1 Uxu operon transcriptional regulator [Salmonella enterica subsp. enterica serovar Infantis] Overall Protective Antigen Prediction = **0.3968** (Probable **NON-ANTIGEN**).

>EHO9887900.1 tryptophan--tRNA ligase [Salmonella enterica subsp. enterica serovar Infantis] Overall Protective Antigen Prediction = **0.3226** (Probable **NON-ANTIGEN**).

>EHO9887901.1 type VI secretion system tube protein Hcp [Salmonella enterica subsp. enterica

serovar Infantis] Overall Protective Antigen Prediction = **0.7194** (Probable **ANTIGEN**).

>EHO9887902.1 aspartate/glutamate racemase family protein [Salmonella enterica subsp. enterica serovar Infantis] Overall Protective Antigen Prediction = **0.2494** (Probable **NON-ANTIGEN**).

>EHO9887903.1 hypochlorite stress DNA-binding transcriptional regulator HypT [Salmonella enterica subsp. enterica serovar Infantis] Overall Protective Antigen Prediction = **0.4390** (Probable **ANTIGEN**).

>EHO9887904.1 beta-aspartyl-peptidase [Salmonella enterica subsp. enterica serovar Infantis] Overall Protective Antigen Prediction = **0.4483** (Probable **ANTIGEN**).

>EHO9887905.1 YjiG family protein [Salmonella enterica subsp. enterica serovar Infantis] Overall Protective Antigen Prediction = **0.3716** (Probable **NON-ANTIGEN**).

>EHO9887906.1 YjiH family protein [Salmonella enterica subsp. enterica serovar Infantis] Overall Protective Antigen Prediction = **0.6632** (Probable **ANTIGEN**).

>EHO9887907.1 MFS transporter [Salmonella enterica subsp. enterica serovar Infantis] Overall Protective Antigen Prediction = **0.3763** (Probable **NON-ANTIGEN**).

>EHO9887908.1 DUF445 domain-containing protein [Salmonella enterica subsp. enterica serovar Infantis] Overall Protective Antigen Prediction = **0.1907** (Probable **NON-ANTIGEN**).

>EHO9887909.1 multidrug efflux MFS transporter MdtM [Salmonella enterica subsp. enterica serovar Infantis] Overall Protective Antigen Prediction = **0.3677** (Probable **NON-ANTIGEN**).

>EHO9887910.1 Rpn family recombination-promoting nuclease/putative transposase [Salmonella enterica subsp. enterica serovar Infantis] Overall Protective Antigen Prediction = **0.4388** (Probable **ANTIGEN**).

>EHO9887911.1 NAD-dependent succinate-semialdehyde dehydrogenase [Salmonella enterica subsp. enterica serovar Infantis] Overall Protective Antigen Prediction = **0.3413** (Probable **NON-ANTIGEN**).

>EHO9887912.1 hypothetical protein KND05_003306 [Salmonella enterica subsp. enterica serovar Infantis] Overall Protective Antigen Prediction = **0.5368** (Probable **ANTIGEN**).

>EHO9887913.1 DUF1127 domain-containing protein [Salmonella enterica subsp. enterica serovar Infantis] Overall Protective Antigen Prediction = **0.2547** (Probable **NON-ANTIGEN**).

>EHO9887914.1 hypothetical protein KND05_003308 [Salmonella enterica subsp. enterica serovar Infantis] Overall Protective Antigen Prediction = **0.5477** (Probable **ANTIGEN**).

>EHO9887915.1 endoribonuclease SymE [Salmonella enterica subsp. enterica serovar Infantis] Overall Protective Antigen Prediction = **0.4454** (Probable **ANTIGEN**).

>EHO9887916.1 LysE family translocator [Salmonella enterica subsp. enterica serovar Infantis] Overall Protective Antigen Prediction = **0.4492** (Probable **ANTIGEN**).

>EHO9887917.1 AraC family transcriptional regulator [Salmonella enterica subsp. enterica serovar Infantis] Overall Protective Antigen Prediction = **0.3524** (Probable **NON-ANTIGEN**).

>EHO9887918.1 GTPase [Salmonella enterica subsp. enterica serovar Infantis] Overall Protective

Antigen Prediction = **0.2265** (Probable **NON-ANTIGEN**).

>EHO9887919.1 YbdD/YjiX family protein [Salmonella enterica subsp. enterica serovar Infantis] Overall Protective Antigen Prediction = **0.3778** (Probable **NON-ANTIGEN**).

>EHO9887920.1 pyruvate/proton symporter BtsT [Salmonella enterica subsp. enterica serovar Infantis] Overall Protective Antigen Prediction = **0.4589** (Probable **ANTIGEN**).

>EHO9887921.1 methyl-accepting chemotaxis protein [Salmonella enterica subsp. enterica serovar Infantis] Overall Protective Antigen Prediction = **0.4402** (Probable **ANTIGEN**).

>EHO9887922.1 phosphatidylglycerol--membrane-oligosaccharide glycerophosphotransferase [Salmonella enterica subsp. enterica serovar Infantis] Overall Protective Antigen Prediction = **0.4054** (Probable **ANTIGEN**).

>EHO9887923.1 DUF2501 domain-containing protein [Salmonella enterica subsp. enterica serovar Infantis] Overall Protective Antigen Prediction = **0.5636** (Probable **ANTIGEN**).

>EHO9887924.1 DNA replication protein DnaC [Salmonella enterica subsp. enterica serovar Infantis] Overall Protective Antigen Prediction = **0.4319** (Probable **ANTIGEN**).

>EHO9887925.1 primosomal protein DnaT [Salmonella enterica subsp. enterica serovar Infantis] Overall Protective Antigen Prediction = **0.4767** (Probable **ANTIGEN**).

>EHO9887926.1 threonine/serine exporter [Salmonella enterica subsp. enterica serovar Infantis] Overall Protective Antigen Prediction = **0.4057** (Probable **ANTIGEN**).

>EHO9887927.1 threonine/serine exporter ThrE family protein [Salmonella enterica subsp. enterica serovar Infantis] Overall Protective Antigen Prediction = **0.4486** (Probable **ANTIGEN**).

>EHO9887928.1 response regulator transcription factor [Salmonella enterica subsp. enterica serovar Infantis] Overall Protective Antigen Prediction = **0.3438** (Probable **NON-ANTIGEN**).

>EHO9887929.1 DNA-binding transcriptional activator BglJ [Salmonella enterica subsp. enterica serovar Infantis] Overall Protective Antigen Prediction = **0.3576** (Probable **NON-ANTIGEN**).

>EHO9887930.1 YbaK/EbsC family protein [Salmonella enterica subsp. enterica serovar Infantis] Overall Protective Antigen Prediction = **0.7245** (Probable **ANTIGEN**).

>EHO9887931.1 siderophore-iron reductase FhuF [Salmonella enterica subsp. enterica serovar Infantis] Overall Protective Antigen Prediction = **0.3849** (Probable **NON-ANTIGEN**).

>EHO9887932.1 GGDEF domain-containing protein [Salmonella enterica subsp. enterica serovar Infantis] Overall Protective Antigen Prediction = **0.6523** (Probable **ANTIGEN**).

>EHO9887933.1 DUF1435 domain-containing protein [Salmonella enterica subsp. enterica serovar Infantis] Overall Protective Antigen Prediction = **0.3821** (Probable **NON-ANTIGEN**).

>EHO9887934.1 16S rRNA (guanine(1207)-N(2))-methyltransferase RsmC [Salmonella enterica subsp. enterica serovar Infantis] Overall Protective Antigen Prediction = **0.5448** (Probable **ANTIGEN**).

>EHO9887935.1 DNA polymerase III subunit psi [Salmonella enterica subsp. enterica serovar Infantis] Overall Protective Antigen Prediction = **0.4015** (Probable **ANTIGEN**).

>EHO9887936.1 ribosomal protein S18-alanine N-acetyltransferase [Salmonella enterica subsp. enterica serovar Infantis] Overall Protective Antigen Prediction = **0.3644** (Probable **NON-ANTIGEN**).

>EHO9887937.1 pyrimidine 5'-nucleotidase [Salmonella enterica subsp. enterica serovar Infantis] Overall Protective Antigen Prediction = **0.3625** (Probable **NON-ANTIGEN**).

>EHO9887938.1 peptide chain release factor 3 [Salmonella enterica subsp. enterica serovar Infantis] Overall Protective Antigen Prediction = **0.4496** (Probable **ANTIGEN**).

>EHO9887939.1 molecular chaperone OsmY [Salmonella enterica subsp. enterica serovar Infantis] Overall Protective Antigen Prediction = **0.8815** (Probable **ANTIGEN**).

>EHO9887940.1 DUF1328 domain-containing protein [Salmonella enterica subsp. enterica serovar Infantis] Overall Protective Antigen Prediction = **0.6815** (Probable **ANTIGEN**).

>EHO9887941.1 patatin family protein [Salmonella enterica subsp. enterica serovar Infantis] Overall Protective Antigen Prediction = **0.3005** (Probable **NON-ANTIGEN**).

>EHO9887942.1 metal-dependent hydrolase [Salmonella enterica subsp. enterica serovar Infantis] Overall Protective Antigen Prediction = **0.5335** (Probable **ANTIGEN**).

>EHO9887943.1 YjjW family glycine radical enzyme activase [Salmonella enterica subsp. enterica serovar Infantis] Overall Protective Antigen Prediction = **0.5401** (Probable **ANTIGEN**).

>EHO9887944.1 YjjI family glycine radical enzyme [Salmonella enterica subsp. enterica serovar Infantis] Overall Protective Antigen Prediction = **0.4089** (Probable **ANTIGEN**).

>EHO9887945.1 deoxyribose-phosphate aldolase [Salmonella enterica subsp. enterica serovar Infantis] Overall Protective Antigen Prediction = **0.5007** (Probable **ANTIGEN**).

>EHO9887946.1 thymidine phosphorylase [Salmonella enterica subsp. enterica serovar Infantis] Overall Protective Antigen Prediction = **0.4618** (Probable **ANTIGEN**).

>EHO9887947.1 phosphopentomutase [Salmonella enterica subsp. enterica serovar Infantis] Overall Protective Antigen Prediction = **0.4674** (Probable **ANTIGEN**).

>EHO9887948.1 purine-nucleoside phosphorylase [Salmonella enterica subsp. enterica serovar Infantis] Overall Protective Antigen Prediction = **0.5195** (Probable **ANTIGEN**).

>EHO9887949.1 type II toxin-antitoxin system HipA family toxin YjjJ [Salmonella enterica subsp. enterica serovar Infantis] Overall Protective Antigen Prediction = **0.4623** (Probable **ANTIGEN**).

>EHO9887950.1 lipoate--protein ligase LplA [Salmonella enterica subsp. enterica serovar Infantis] Overall Protective Antigen Prediction = **0.4480** (Probable **ANTIGEN**).

>EHO9887951.1 YtjB family periplasmic protein [Salmonella enterica subsp. enterica serovar Infantis] Overall Protective Antigen Prediction = **0.5094** (Probable **ANTIGEN**).

>EHO9887952.1 phosphoserine phosphatase [Salmonella enterica subsp. enterica serovar Infantis] Overall Protective Antigen Prediction = **0.5002** (Probable **ANTIGEN**).

>EHO9887953.1 DNA repair protein RadA [Salmonella enterica subsp. enterica serovar Infantis]

Overall Protective Antigen Prediction = **0.3183** (Probable **NON-ANTIGEN**).

>EHO9887954.1 multifunctional transcriptional regulator/nicotinamide-nucleotide adenylyltransferase/ribosylnicotinamide kinase NadR [Salmonella enterica subsp. enterica serovar Infantis] Overall Protective Antigen Prediction = **0.3588** (Probable **NON-ANTIGEN**).

>EHO9887955.1 energy-dependent translational throttle protein EttA [Salmonella enterica subsp. enterica serovar Infantis] Overall Protective Antigen Prediction = **0.4452** (Probable **ANTIGEN**).

>EHO9887956.1 murein transglycosylase [Salmonella enterica subsp. enterica serovar Infantis] Overall Protective Antigen Prediction = **0.4177** (Probable **ANTIGEN**).

>EHO9887957.1 trp operon repressor [Salmonella enterica subsp. enterica serovar Infantis] Overall Protective Antigen Prediction = **0.4571** (Probable **ANTIGEN**).

>EHO9887958.1 hypothetical protein KND05_003355, partial [Salmonella enterica subsp. enterica serovar Infantis] Overall Protective Antigen Prediction = **1.2705** (Probable **ANTIGEN**).

>EHO9887959.1 hypothetical protein KND05_003356 [Salmonella enterica subsp. enterica serovar Infantis] Overall Protective Antigen Prediction = **0.5782** (Probable **ANTIGEN**).

>EHO9887960.1 hypothetical protein KND05_003357 [Salmonella enterica subsp. enterica serovar Infantis] Overall Protective Antigen Prediction = **0.5640** (Probable **ANTIGEN**).

>EHO9887961.1 CaiF/GrlA family transcriptional regulator [Salmonella enterica subsp. enterica serovar Infantis] Overall Protective Antigen Prediction = **0.7811** (Probable **ANTIGEN**).

>EHO9887962.1 hypothetical protein KND05_003359 [Salmonella enterica subsp. enterica serovar Infantis] Overall Protective Antigen Prediction = **0.3338** (Probable **NON-ANTIGEN**).

>EHO9887963.1 hypothetical protein KND05_003360 [Salmonella enterica subsp. enterica serovar Infantis] Overall Protective Antigen Prediction = **0.5845** (Probable **ANTIGEN**).

>EHO9887964.1 peptidase domain-containing ABC transporter [Salmonella enterica subsp. enterica serovar Infantis] Overall Protective Antigen Prediction = **0.4079** (Probable **ANTIGEN**).

>EHO9887965.1 HlyD family efflux transporter periplasmic adaptor subunit [Salmonella enterica subsp. enterica serovar Infantis] Overall Protective Antigen Prediction = **0.4021** (Probable **ANTIGEN**).

>EHO9887966.1 hypothetical protein KND05_003364 [Salmonella enterica subsp. enterica serovar Infantis] Overall Protective Antigen Prediction = **0.6564** (Probable **ANTIGEN**).

>EHO9887967.1 transcriptional regulator [Salmonella enterica subsp. enterica serovar Infantis] Overall Protective Antigen Prediction = **0.2870** (Probable **NON-ANTIGEN**).

>EHO9887968.1 CaiF/GrlA family transcriptional regulator [Salmonella enterica subsp. enterica serovar Infantis] Overall Protective Antigen Prediction = **0.6898** (Probable **ANTIGEN**).

>EHO9887969.1 helix-turn-helix domain-containing protein [Salmonella enterica subsp. enterica serovar Infantis] Overall Protective Antigen Prediction = **1.1100** (Probable **ANTIGEN**).

>EHO9887970.1 hypothetical protein KND05_003373 [Salmonella enterica subsp. enterica serovar Infantis] Overall Protective Antigen Prediction = **0.8852** (Probable **ANTIGEN**).

>EHO9887971.1 type 1 fimbrial protein [Salmonella enterica subsp. enterica serovar Infantis] Overall Protective Antigen Prediction = **0.8319** (Probable **ANTIGEN**).

>EHO9887972.1 fimbrial biogenesis outer membrane usher protein [Salmonella enterica subsp. enterica serovar Infantis] Overall Protective Antigen Prediction = **0.5914** (Probable **ANTIGEN**).

>EHO9887973.1 fimbria/pilus periplasmic chaperone [Salmonella enterica subsp. enterica serovar Infantis] Overall Protective Antigen Prediction = **0.4992** (Probable **ANTIGEN**).

>EHO9887974.1 fimbrial protein [Salmonella enterica subsp. enterica serovar Infantis] Overall Protective Antigen Prediction = **0.8824** (Probable **ANTIGEN**).

>EHO9887975.1 cytoplasmic protein [Salmonella enterica subsp. enterica serovar Infantis] Overall Protective Antigen Prediction = **0.6117** (Probable **ANTIGEN**).

>EHO9887976.1 type II toxin-antitoxin system toxin CcdB [Salmonella enterica subsp. enterica serovar Infantis] Overall Protective Antigen Prediction = **0.4215** (Probable **ANTIGEN**).

>EHO9887977.1 type II toxin-antitoxin system antitoxin CcdA [Salmonella enterica subsp. enterica serovar Infantis] Overall Protective Antigen Prediction = **0.4426** (Probable **ANTIGEN**).

>EHO9887978.1 AbrB/MazE/SpoVT family DNA-binding domain-containing protein [Salmonella enterica subsp. enterica serovar Infantis] Overall Protective Antigen Prediction = **0.3940** (Probable **NON-ANTIGEN**).

>EHO9887979.1 type II toxin-antitoxin system VapC family toxin [Salmonella enterica subsp. enterica serovar Infantis] Overall Protective Antigen Prediction = **0.4229** (Probable **ANTIGEN**).

>EHO9887980.1 YjiK family protein [Salmonella enterica subsp. enterica serovar Infantis] Overall Protective Antigen Prediction = **0.4033** (Probable **ANTIGEN**).

>EHO9887981.1 hypothetical protein KND05_003387 [Salmonella enterica subsp. enterica serovar Infantis] Overall Protective Antigen Prediction = **0.7585** (Probable **ANTIGEN**).

>EHO9887982.1 faeA-like family protein [Salmonella enterica subsp. enterica serovar Infantis] Overall Protective Antigen Prediction = **0.5688** (Probable **ANTIGEN**).

>EHO9887983.1 hypothetical protein KND05_003389 [Salmonella enterica subsp. enterica serovar Infantis] Overall Protective Antigen Prediction = **0.4692** (Probable **ANTIGEN**).

>EHO9887984.1 fimbrial protein [Salmonella enterica subsp. enterica serovar Infantis] Overall Protective Antigen Prediction = **0.6721** (Probable **ANTIGEN**).

>EHO9887985.1 F4 (K88) fimbria minor subunit FaeH [Salmonella enterica subsp. enterica serovar Infantis] Overall Protective Antigen Prediction = **0.6515** (Probable **ANTIGEN**).

>EHO9887986.1 hypothetical protein KND05_003392 [Salmonella enterica subsp. enterica serovar Infantis] Overall Protective Antigen Prediction = **0.5170** (Probable **ANTIGEN**).

>EHO9887987.1 DUF5462 family protein [Salmonella enterica subsp. enterica serovar Infantis] Overall Protective Antigen Prediction = **0.6059** (Probable **ANTIGEN**).

>EHO9887988.1 fimbria/pilus periplasmic chaperone [Salmonella enterica subsp. enterica serovar

Infantis] Overall Protective Antigen Prediction = **0.5094** (Probable **ANTIGEN**).

>EHO9887989.1 type 1 fimbrial protein [Salmonella enterica subsp. enterica serovar Infantis] Overall Protective Antigen Prediction = **0.8726** (Probable **ANTIGEN**).

>EHO9887990.1 adhesin biosynthesis transcription regulatory family protein [Salmonella enterica subsp. enterica serovar Infantis] Overall Protective Antigen Prediction = **0.3151** (Probable **NON-ANTIGEN**).

>EHO9887991.1 transposase [Salmonella enterica subsp. enterica serovar Infantis] Overall Protective Antigen Prediction = **0.4511** (Probable **ANTIGEN**).

>EHO9887992.1 helix-turn-helix domain-containing protein [Salmonella enterica subsp. enterica serovar Infantis] Overall Protective Antigen Prediction = **0.3790** (Probable **NON-ANTIGEN**).

>EHO9887993.1 Rpn family recombination-promoting nuclease/putative transposase [Salmonella enterica subsp. enterica serovar Infantis] Overall Protective Antigen Prediction = **0.8545** (Probable **ANTIGEN**).

>EHO9887994.1 Rpn family recombination-promoting nuclease/putative transposase [Salmonella enterica subsp. enterica serovar Infantis] Overall Protective Antigen Prediction = **0.5194** (Probable **ANTIGEN**).

>EHO9887995.1 hypothetical protein KND05_003405 [Salmonella enterica subsp. enterica serovar Infantis] Overall Protective Antigen Prediction = **0.5976** (Probable **ANTIGEN**).

>EHO9887996.1 hemolysin expression modulator Hha [Salmonella enterica subsp. enterica serovar Infantis] Overall Protective Antigen Prediction = **0.3860** (Probable **NON-ANTIGEN**).

>EHO9887997.1 GNAT family N-acetyltransferase [Salmonella enterica subsp. enterica serovar Infantis] Overall Protective Antigen Prediction = **0.3012** (Probable **NON-ANTIGEN**).

>EHO9887998.1 DUF1778 domain-containing protein [Salmonella enterica subsp. enterica serovar Infantis] Overall Protective Antigen Prediction = **0.4646** (Probable **ANTIGEN**).

>EHO9887999.1 PIN domain-containing protein [Salmonella enterica subsp. enterica serovar Infantis] Overall Protective Antigen Prediction = **0.6435** (Probable **ANTIGEN**).

>EHO9888000.1 hypothetical protein KND05_003414 [Salmonella enterica subsp. enterica serovar Infantis] Overall Protective Antigen Prediction = **0.1607** (Probable **NON-ANTIGEN**).

>EHO9888001.1 type II toxin-antitoxin system RelE/ParE family toxin [Salmonella enterica subsp. enterica serovar Infantis] Overall Protective Antigen Prediction = **0.5486** (Probable **ANTIGEN**).

>EHO9888002.1 damage-inducible protein J [Salmonella enterica subsp. enterica serovar Infantis] Overall Protective Antigen Prediction = **0.5614** (Probable **ANTIGEN**).

>EHO9888003.1 IS66 family insertion sequence hypothetical protein [Salmonella enterica subsp. enterica serovar Infantis] Overall Protective Antigen Prediction = **0.3409** (Probable **NON-ANTIGEN**).

>EHO9888004.1 IS66 family insertion sequence element accessory protein TnpB [Salmonella enterica subsp. enterica serovar Infantis] Overall Protective Antigen Prediction = **0.4837** (Probable **ANTIGEN**).

>EHO9888005.1 IS110 family transposase [Salmonella enterica subsp. enterica serovar Infantis] Overall Protective Antigen Prediction = **0.3913** (Probable **NON-ANTIGEN**).

>EHO9888006.1 TerB N-terminal domain-containing protein [Salmonella enterica subsp. enterica serovar Infantis] Overall Protective Antigen Prediction = **0.4791** (Probable **ANTIGEN**).

>EHO9888007.1 ATP-binding protein [Salmonella enterica subsp. enterica serovar Infantis] Overall Protective Antigen Prediction = **0.4598** (Probable **ANTIGEN**).

>EHO9888008.1 DEAD/DEAH box helicase [Salmonella enterica subsp. enterica serovar Infantis] Overall Protective Antigen Prediction = **0.4323** (Probable **ANTIGEN**).

>EHO9888009.1 type II toxin-antitoxin system PemK/MazF family toxin [Salmonella enterica subsp. enterica serovar Infantis] Overall Protective Antigen Prediction = **0.6397** (Probable **ANTIGEN**).

>EHO9888010.1 antitoxin [Salmonella enterica subsp. enterica serovar Infantis] Overall Protective Antigen Prediction = **0.3942** (Probable **NON-ANTIGEN**).

>EHO9888011.1 phosphoenolpyruvate carboxylase [Salmonella enterica subsp. enterica serovar Infantis] Overall Protective Antigen Prediction = **0.3442** (Probable **NON-ANTIGEN**).

>EHO9888012.1 phosphoethanolamine transferase CptA [Salmonella enterica subsp. enterica serovar Infantis] Overall Protective Antigen Prediction = **0.4545** (Probable **ANTIGEN**).

>EHO9888013.1 helix-turn-helix transcriptional regulator [Salmonella enterica subsp. enterica serovar Infantis] Overall Protective Antigen Prediction = **0.4162** (Probable **ANTIGEN**).

>EHO9888014.1 fructose-6-phosphate aldolase [Salmonella enterica subsp. enterica serovar Infantis] Overall Protective Antigen Prediction = **0.3474** (Probable **NON-ANTIGEN**).

>EHO9888015.1 glycerol dehydrogenase [Salmonella enterica subsp. enterica serovar Infantis] Overall Protective Antigen Prediction = **0.4343** (Probable **ANTIGEN**).

>EHO9888016.1 DUF1287 domain-containing protein [Salmonella enterica subsp. enterica serovar Infantis] Overall Protective Antigen Prediction = **0.5583** (Probable **ANTIGEN**).

>EHO9888017.1 catalase/peroxidase HPI [Salmonella enterica subsp. enterica serovar Infantis] Overall Protective Antigen Prediction = **0.5548** (Probable **ANTIGEN**).

>EHO9888018.1 methylenetetrahydrofolate reductase [Salmonella enterica subsp. enterica serovar Infantis] Overall Protective Antigen Prediction = **0.5286** (Probable **ANTIGEN**).

>EHO9888019.1 bifunctional metallophosphatase/5'-nucleotidase [Salmonella enterica subsp. enterica serovar Infantis] Overall Protective Antigen Prediction = **0.4207** (Probable **ANTIGEN**).

>EHO9888020.1 hypothetical protein KND05_003440 [Salmonella enterica subsp. enterica serovar Infantis] Overall Protective Antigen Prediction = **0.7126** (Probable **ANTIGEN**).

>EHO9888021.1 small-conductance mechanosensitive channel MscS [Salmonella enterica subsp. enterica serovar Infantis] Overall Protective Antigen Prediction = **0.2842** (Probable **NON-ANTIGEN**).

>EHO9888022.1 bifunctional aspartate kinase/homoserine dehydrogenase II [Salmonella enterica

subsp. enterica serovar Infantis] Overall Protective Antigen Prediction = **0.3929** (Probable **NON-ANTIGEN**).

>EHO9888023.1 cystathionine gamma-synthase [Salmonella enterica subsp. enterica serovar Infantis] Overall Protective Antigen Prediction = **0.4283** (Probable **ANTIGEN**).

>EHO9888024.1 met regulon transcriptional regulator MetJ [Salmonella enterica subsp. enterica serovar Infantis] Overall Protective Antigen Prediction = **0.4321** (Probable **ANTIGEN**).

>EHO9888025.1 aryl-sulfate sulfotransferase [Salmonella enterica subsp. enterica serovar Infantis] Overall Protective Antigen Prediction = **0.3532** (Probable **NON-ANTIGEN**).

>EHO9888026.1 TIGR02117 family protein [Salmonella enterica subsp. enterica serovar Infantis] Overall Protective Antigen Prediction = **0.2571** (Probable **NON-ANTIGEN**).

>EHO9888027.1 50S ribosomal protein L31 [Salmonella enterica subsp. enterica serovar Infantis] Overall Protective Antigen Prediction = **0.8833** (Probable **ANTIGEN**).

>EHO9888028.1 primosomal protein N' [Salmonella enterica subsp. enterica serovar Infantis] Overall Protective Antigen Prediction = **0.4350** (Probable **ANTIGEN**).

>EHO9888029.1 DNA-binding transcriptional regulator CytR [Salmonella enterica subsp. enterica serovar Infantis] Overall Protective Antigen Prediction = **0.4030** (Probable **ANTIGEN**).

>EHO9888030.1 cell division protein FtsN [Salmonella enterica subsp. enterica serovar Infantis] Overall Protective Antigen Prediction = **0.6864** (Probable **ANTIGEN**).

>EHO9888031.1 ATP-dependent protease subunit HslV [Salmonella enterica subsp. enterica serovar Infantis] Overall Protective Antigen Prediction = **0.3687** (Probable **NON-ANTIGEN**).

>EHO9888032.1 HslU--HslV peptidase ATPase subunit [Salmonella enterica subsp. enterica serovar Infantis] Overall Protective Antigen Prediction = **0.5309** (Probable **ANTIGEN**).

>EHO9888033.1 1,4-dihydroxy-2-naphthoate polyprenyltransferase [Salmonella enterica subsp. enterica serovar Infantis] Overall Protective Antigen Prediction = **0.3908** (Probable **NON-ANTIGEN**).

>EHO9888034.1 ribonuclease E activity regulator RraA [Salmonella enterica subsp. enterica serovar Infantis] Overall Protective Antigen Prediction = **0.4293** (Probable **ANTIGEN**).

>EHO9888035.1 septal ring assembly protein ZapB [Salmonella enterica subsp. enterica serovar Infantis] Overall Protective Antigen Prediction = **0.6882** (Probable **ANTIGEN**).

>EHO9888036.1 aquaporin [Salmonella enterica subsp. enterica serovar Infantis] Overall Protective Antigen Prediction = **0.4321** (Probable **ANTIGEN**).

>EHO9888037.1 glycerol kinase GlpK [Salmonella enterica subsp. enterica serovar Infantis] Overall Protective Antigen Prediction = **0.4494** (Probable **ANTIGEN**).

>EHO9888038.1 class II fructose-bisphosphatase [Salmonella enterica subsp. enterica serovar Infantis] Overall Protective Antigen Prediction = **0.4536** (Probable **ANTIGEN**).

>EHO9888039.1 ferredoxin--NADP(+) reductase [Salmonella enterica subsp. enterica serovar Infantis] Overall Protective Antigen Prediction = **0.3579** (Probable **NON-ANTIGEN**).

>EHO9888040.1 DUF805 domain-containing protein [Salmonella enterica subsp. enterica serovar Infantis] Overall Protective Antigen Prediction = **0.7337** (Probable **ANTIGEN**).

>EHO9888041.1 YiiQ family protein [Salmonella enterica subsp. enterica serovar Infantis] Overall Protective Antigen Prediction = **0.7097** (Probable **ANTIGEN**).

>EHO9888042.1 triose-phosphate isomerase [Salmonella enterica subsp. enterica serovar Infantis] Overall Protective Antigen Prediction = **0.6221** (Probable **ANTIGEN**).

>EHO9888043.1 epimerase [Salmonella enterica subsp. enterica serovar Infantis] Overall Protective Antigen Prediction = **0.4470** (Probable **ANTIGEN**).

>EHO9888044.1 (4S)-4-hydroxy-5-phosphonooxypentane-2,3-dione isomerase [Salmonella enterica subsp. enterica serovar Infantis] Overall Protective Antigen Prediction = **0.3988** (Probable **NON-ANTIGEN**).

>EHO9888045.1 3-hydroxy-5-phosphonooxypentane-2,4-dione thiolase [Salmonella enterica subsp. enterica serovar Infantis] Overall Protective Antigen Prediction = **0.3136** (Probable **NON-ANTIGEN**).

>EHO9888046.1 autoinducer 2 ABC transporter substrate-binding protein LsrB [Salmonella enterica subsp. enterica serovar Infantis] Overall Protective Antigen Prediction = **0.3626** (Probable **NON-ANTIGEN**).

>EHO9888047.1 autoinducer 2 ABC transporter permease LsrD [Salmonella enterica subsp. enterica serovar Infantis] Overall Protective Antigen Prediction = **0.5278** (Probable **ANTIGEN**).

>EHO9888048.1 autoinducer 2 ABC transporter permease LsrC [Salmonella enterica subsp. enterica serovar Infantis] Overall Protective Antigen Prediction = **0.4351** (Probable **ANTIGEN**).

>EHO9888049.1 autoinducer 2 ABC transporter ATP-binding protein LsrA [Salmonella enterica subsp. enterica serovar Infantis] Overall Protective Antigen Prediction = **0.4320** (Probable **ANTIGEN**).

>EHO9888050.1 transcriptional regulator LsrR [Salmonella enterica subsp. enterica serovar Infantis] Overall Protective Antigen Prediction = **0.4643** (Probable **ANTIGEN**).

>EHO9888051.1 autoinducer-2 kinase [Salmonella enterica subsp. enterica serovar Infantis] Overall Protective Antigen Prediction = **0.4224** (Probable **ANTIGEN**).

>EHO9888052.1 cupin domain-containing protein [Salmonella enterica subsp. enterica serovar Infantis] Overall Protective Antigen Prediction = **0.4539** (Probable **ANTIGEN**).

>EHO9888053.1 hypothetical protein KND05_003474 [Salmonella enterica subsp. enterica serovar Infantis] Overall Protective Antigen Prediction = **0.7650** (Probable **ANTIGEN**).

>EHO9888054.1 GntR family transcriptional regulator [Salmonella enterica subsp. enterica serovar Infantis] Overall Protective Antigen Prediction = **0.3520** (Probable **NON-ANTIGEN**).

>EHO9888055.1 ADP-ribosylglycohydrolase family protein [Salmonella enterica subsp. enterica serovar Infantis] Overall Protective Antigen Prediction = **0.3426** (Probable **NON-ANTIGEN**).

>EHO9888056.1 aminoimidazole riboside kinase [Salmonella enterica subsp. enterica serovar

Infantis] Overall Protective Antigen Prediction = **0.3451** (Probable **NON-ANTIGEN**).

>EHO9888057.1 MFS transporter [Salmonella enterica subsp. enterica serovar Infantis] Overall Protective Antigen Prediction = **0.5589** (Probable **ANTIGEN**).

>EHO9888058.1 CDP-diacylglycerol diphosphatase [Salmonella enterica subsp. enterica serovar Infantis] Overall Protective Antigen Prediction = **0.4990** (Probable **ANTIGEN**).

>EHO9888059.1 sulfate ABC transporter substrate-binding protein [Salmonella enterica subsp. enterica serovar Infantis] Overall Protective Antigen Prediction = **0.4471** (Probable **ANTIGEN**).

>EHO9888060.1 6-phosphofructokinase [Salmonella enterica subsp. enterica serovar Infantis] Overall Protective Antigen Prediction = **0.2521** (Probable **NON-ANTIGEN**).

>EHO9888061.1 CDF family cation-efflux transporter FieF [Salmonella enterica subsp. enterica serovar Infantis] Overall Protective Antigen Prediction = **0.4306** (Probable **ANTIGEN**).

>EHO9888062.1 prophage transcriptional regulator OgrK [Salmonella enterica subsp. enterica serovar Infantis] Overall Protective Antigen Prediction = **0.4806** (Probable **ANTIGEN**).

>EHO9888063.1 phage late control D family protein [Salmonella enterica subsp. enterica serovar Infantis] Overall Protective Antigen Prediction = **0.6984** (Probable **ANTIGEN**).

>EHO9888064.1 phage tail protein [Salmonella enterica subsp. enterica serovar Infantis] Overall Protective Antigen Prediction = **0.3991** (Probable **NON-ANTIGEN**).

>EHO9888065.1 phage tail tape measure protein [Salmonella enterica subsp. enterica serovar Infantis] Overall Protective Antigen Prediction = **0.5196** (Probable **ANTIGEN**).

>EHO9888066.1 GpE family phage tail protein [Salmonella enterica subsp. enterica serovar Infantis] Overall Protective Antigen Prediction = **0.6319** (Probable **ANTIGEN**).

>EHO9888067.1 phage tail assembly protein [Salmonella enterica subsp. enterica serovar Infantis] Overall Protective Antigen Prediction = **0.2650** (Probable **NON-ANTIGEN**).

>EHO9888068.1 phage major tail tube protein [Salmonella enterica subsp. enterica serovar Infantis] Overall Protective Antigen Prediction = **0.7080** (Probable **ANTIGEN**).

>EHO9888069.1 phage tail sheath protein [Salmonella enterica subsp. enterica serovar Infantis] Overall Protective Antigen Prediction = **0.5039** (Probable **ANTIGEN**).

>EHO9888070.1 recombinase family protein [Salmonella enterica subsp. enterica serovar Infantis] Overall Protective Antigen Prediction = **0.3861** (Probable **NON-ANTIGEN**).

>EHO9888071.1 TIGR00645 family protein [Salmonella enterica subsp. enterica serovar Infantis] Overall Protective Antigen Prediction = **0.4480** (Probable **ANTIGEN**).

>EHO9888072.1 HAMP domain-containing protein [Salmonella enterica subsp. enterica serovar Infantis] Overall Protective Antigen Prediction = **0.4441** (Probable **ANTIGEN**).

>EHO9888073.1 DUF2623 domain-containing protein [Salmonella enterica subsp. enterica serovar Infantis] Overall Protective Antigen Prediction = **0.3565** (Probable **NON-ANTIGEN**).

>EHO9888074.1 hydrogenase 2 small subunit [Salmonella enterica subsp. enterica serovar Infantis]

Overall Protective Antigen Prediction = **0.4547** (Probable **ANTIGEN**).

>EHO9888075.1 hydrogenase 2 operon protein HybA [Salmonella enterica subsp. enterica serovar Infantis] Overall Protective Antigen Prediction = **0.4473** (Probable **ANTIGEN**).

>EHO9888076.1 Ni/Fe-hydrogenase cytochrome b subunit [Salmonella enterica subsp. enterica serovar Infantis] Overall Protective Antigen Prediction = **0.6061** (Probable **ANTIGEN**).

>EHO9888077.1 hydrogenase 2 large subunit [Salmonella enterica subsp. enterica serovar Infantis] Overall Protective Antigen Prediction = **0.2433** (Probable **NON-ANTIGEN**).

>EHO9888078.1 HyaD/HybD family hydrogenase maturation endopeptidase [Salmonella enterica subsp. enterica serovar Infantis] Overall Protective Antigen Prediction = **0.3252** (Probable **NON-ANTIGEN**).

>EHO9888079.1 hydrogenase-2 assembly chaperone [Salmonella enterica subsp. enterica serovar Infantis] Overall Protective Antigen Prediction = **0.2819** (Probable **NON-ANTIGEN**).

>EHO9888080.1 hydrogenase maturation nickel metallochaperone HypA [Salmonella enterica subsp. enterica serovar Infantis] Overall Protective Antigen Prediction = **0.7601** (Probable **ANTIGEN**).

>EHO9888081.1 hydrogenase maturation factor HybG [Salmonella enterica subsp. enterica serovar Infantis] Overall Protective Antigen Prediction = **0.4193** (Probable **ANTIGEN**).

>EHO9888082.1 ABC transporter substrate-binding protein [Salmonella enterica subsp. enterica serovar Infantis] Overall Protective Antigen Prediction = **0.3732** (Probable **NON-ANTIGEN**).

>EHO9888083.1 molybdate ABC transporter substrate-binding protein [Salmonella enterica subsp. enterica serovar Infantis] Overall Protective Antigen Prediction = **0.3814** (Probable **NON-ANTIGEN**).

>EHO9888084.1 glutathione-dependent disulfide-bond oxidoreductase [Salmonella enterica subsp. enterica serovar Infantis] Overall Protective Antigen Prediction = **0.4112** (Probable **ANTIGEN**).

>EHO9888085.1 bifunctional glutathionylspermidine amidase/synthase [Salmonella enterica subsp. enterica serovar Infantis] Overall Protective Antigen Prediction = **0.4609** (Probable **ANTIGEN**).

>EHO9888086.1 CZB domain-containing protein [Salmonella enterica subsp. enterica serovar Infantis] Overall Protective Antigen Prediction = **0.3313** (Probable **NON-ANTIGEN**).

>EHO9888087.1 glucuronate isomerase [Salmonella enterica subsp. enterica serovar Infantis] Overall Protective Antigen Prediction = **0.3082** (Probable **NON-ANTIGEN**).

>EHO9888088.1 fructuronate reductase [Salmonella enterica subsp. enterica serovar Infantis] Overall Protective Antigen Prediction = **0.3758** (Probable **NON-ANTIGEN**).

>EHO9888089.1 mannonate dehydratase [Salmonella enterica subsp. enterica serovar Infantis] Overall Protective Antigen Prediction = **0.3729** (Probable **NON-ANTIGEN**).

>EHO9888090.1 MFS transporter [Salmonella enterica subsp. enterica serovar Infantis] Overall Protective Antigen Prediction = **0.5480** (Probable **ANTIGEN**).

>EHO9888091.1 carbon-nitrogen hydrolase family protein [Salmonella enterica subsp. enterica serovar Infantis] Overall Protective Antigen Prediction = **0.3032** (Probable **NON-ANTIGEN**).

>EHO9888092.1 polysaccharide deacetylase [Salmonella enterica subsp. enterica serovar Infantis] Overall Protective Antigen Prediction = **0.2170** (Probable **NON-ANTIGEN**).

>EHO9888093.1 type VI secretion system tube protein Hcp [Salmonella enterica subsp. enterica serovar Infantis] Overall Protective Antigen Prediction = **0.8309** (Probable **ANTIGEN**).

>EHO9888094.1 anti-adapter protein IraD [Salmonella enterica subsp. enterica serovar Infantis] Overall Protective Antigen Prediction = **0.5343** (Probable **ANTIGEN**).

>EHO9888095.1 NAD-dependent phenylacetaldehyde dehydrogenase [Salmonella enterica subsp. enterica serovar Infantis] Overall Protective Antigen Prediction = **0.3612** (Probable **NON-ANTIGEN**).

>EHO9888096.1 FAD-binding oxidoreductase [Salmonella enterica subsp. enterica serovar Infantis] Overall Protective Antigen Prediction = **0.4191** (Probable **ANTIGEN**).

>EHO9888097.1 cupin domain-containing protein [Salmonella enterica subsp. enterica serovar Infantis] Overall Protective Antigen Prediction = **0.6108** (Probable **ANTIGEN**).

>EHO9888098.1 APC family permease [Salmonella enterica subsp. enterica serovar Infantis] Overall Protective Antigen Prediction = **0.3965** (Probable **NON-ANTIGEN**).

>EHO9888099.1 DUF3156 family protein [Salmonella enterica subsp. enterica serovar Infantis] Overall Protective Antigen Prediction = **0.2548** (Probable **NON-ANTIGEN**).

>EHO9888100.1 LuxR family transcriptional regulator [Salmonella enterica subsp. enterica serovar Infantis] Overall Protective Antigen Prediction = **0.3442** (Probable **NON-ANTIGEN**).

>EHO9888101.1 anaerobic sulfatase maturase [Salmonella enterica subsp. enterica serovar Infantis] Overall Protective Antigen Prediction = **0.4409** (Probable **ANTIGEN**).

>EHO9888102.1 arylsulfatase [Salmonella enterica subsp. enterica serovar Infantis] Overall Protective Antigen Prediction = **0.3854** (Probable **NON-ANTIGEN**).

>EHO9888103.1 itaconate degradation transcriptional regulator RipR [Salmonella enterica subsp. enterica serovar Infantis] Overall Protective Antigen Prediction = **0.4731** (Probable **ANTIGEN**).

>EHO9888104.1 itaconate degradation C-C-lyase RipC [Salmonella enterica subsp. enterica serovar Infantis] Overall Protective Antigen Prediction = **0.4644** (Probable **ANTIGEN**).

>EHO9888105.1 (R)-specific enoyl-CoA hydratase RipB/Ich [Salmonella enterica subsp. enterica serovar Infantis] Overall Protective Antigen Prediction = **0.4879** (Probable **ANTIGEN**).

>EHO9888106.1 itaconate CoA-transferase RipA/Ict [Salmonella enterica subsp. enterica serovar Infantis] Overall Protective Antigen Prediction = **0.4804** (Probable **ANTIGEN**).

>EHO9888107.1 VOC family protein [Salmonella enterica subsp. enterica serovar Infantis] Overall Protective Antigen Prediction = **0.3850** (Probable **NON-ANTIGEN**).

>EHO9888108.1 hypothetical protein KND05_003529 [Salmonella enterica subsp. enterica serovar Infantis] Overall Protective Antigen Prediction = **0.5005** (Probable **ANTIGEN**).

>EHO9888109.1 hypothetical protein KND05_003530 [Salmonella enterica subsp. enterica serovar

Infantis] Overall Protective Antigen Prediction = **0.3948** (Probable **NON-ANTIGEN**).

>EHO9888110.1 DEAD/DEAH box helicase [Salmonella enterica subsp. enterica serovar Infantis] Overall Protective Antigen Prediction = **0.3530** (Probable **NON-ANTIGEN**).

>EHO9888111.1 ATP-binding protein [Salmonella enterica subsp. enterica serovar Infantis] Overall Protective Antigen Prediction = **0.4798** (Probable **ANTIGEN**).

>EHO9888112.1 TerB N-terminal domain-containing protein [Salmonella enterica subsp. enterica serovar Infantis] Overall Protective Antigen Prediction = **0.5025** (Probable **ANTIGEN**).

>EHO9888113.1 nucleotidyl transferase AbiEii/AbiGii toxin family protein [Salmonella enterica subsp. enterica serovar Infantis] Overall Protective Antigen Prediction = **0.3751** (Probable **NON-ANTIGEN**).

>EHO9888114.1 hypothetical protein KND05_003535 [Salmonella enterica subsp. enterica serovar Infantis] Overall Protective Antigen Prediction = **0.3298** (Probable **NON-ANTIGEN**).

>EHO9888115.1 ferric reductase-like transmembrane domain-containing protein [Salmonella enterica subsp. enterica serovar Infantis] Overall Protective Antigen Prediction = **0.3799** (Probable **NON-ANTIGEN**).

>EHO9888116.1 DUF1471 domain-containing protein [Salmonella enterica subsp. enterica serovar Infantis] Overall Protective Antigen Prediction = **0.5175** (Probable **ANTIGEN**).

>EHO9888117.1 NAD(P)H-dependent oxidoreductase [Salmonella enterica subsp. enterica serovar Infantis] Overall Protective Antigen Prediction = **0.5380** (Probable **ANTIGEN**).

>EHO9888118.1 LysR family transcriptional regulator [Salmonella enterica subsp. enterica serovar Infantis] Overall Protective Antigen Prediction = **0.4406** (Probable **ANTIGEN**).

>EHO9888119.1 DUF4102 domain-containing protein [Salmonella enterica subsp. enterica serovar Infantis] Overall Protective Antigen Prediction = **0.7680** (Probable **ANTIGEN**).

>EHO9888120.1 DUF554 domain-containing protein [Salmonella enterica subsp. enterica serovar Infantis] Overall Protective Antigen Prediction = **0.4343** (Probable **ANTIGEN**).

>EHO9888121.1 ornithine decarboxylase [Salmonella enterica subsp. enterica serovar Infantis] Overall Protective Antigen Prediction = **0.3717** (Probable **NON-ANTIGEN**).

>EHO9888122.1 nucleoside permease [Salmonella enterica subsp. enterica serovar Infantis] Overall Protective Antigen Prediction = **0.5280** (Probable **ANTIGEN**).

>EHO9888123.1 intimin-like inverse autotransporter SinH [Salmonella enterica subsp. enterica serovar Infantis] Overall Protective Antigen Prediction = **0.6847** (Probable **ANTIGEN**).

>EHO9888124.1 hypothetical protein KND05_003547 [Salmonella enterica subsp. enterica serovar Infantis] Overall Protective Antigen Prediction = **0.8321** (Probable **ANTIGEN**).

>EHO9888125.1 hypothetical protein KND05_003548 [Salmonella enterica subsp. enterica serovar Infantis] Overall Protective Antigen Prediction = **0.6112** (Probable **ANTIGEN**).

>EHO9888126.1 hypothetical protein KND05_003549 [Salmonella enterica subsp. enterica serovar Infantis] Overall Protective Antigen Prediction = **0.6564** (Probable **ANTIGEN**).

>EHO9888127.1 fibronectin-binding autotransporter adhesin ShdA [Salmonella enterica subsp. enterica serovar Infantis] Overall Protective Antigen Prediction = **0.8319** (Probable **ANTIGEN**).

>EHO9888128.1 exodeoxyribonuclease VII large subunit [Salmonella enterica subsp. enterica serovar Infantis] Overall Protective Antigen Prediction = **0.4830** (Probable **ANTIGEN**).

>EHO9888129.1 IMP dehydrogenase [Salmonella enterica subsp. enterica serovar Infantis] Overall Protective Antigen Prediction = **0.6028** (Probable **ANTIGEN**).

>EHO9888130.1 glutamine-hydrolyzing GMP synthase [Salmonella enterica subsp. enterica serovar Infantis] Overall Protective Antigen Prediction = **0.4359** (Probable **ANTIGEN**).

>EHO9888131.1 DUF1493 family protein [Salmonella enterica subsp. enterica serovar Infantis] Overall Protective Antigen Prediction = **0.1947** (Probable **NON-ANTIGEN**).

>EHO9888132.1 hypothetical protein KND05_003557 [Salmonella enterica subsp. enterica serovar Infantis] Overall Protective Antigen Prediction = **0.0532** (Probable **NON-ANTIGEN**).

>EHO9888133.1 DUF2633 family protein [Salmonella enterica subsp. enterica serovar Infantis] Overall Protective Antigen Prediction = **0.7739** (Probable **ANTIGEN**).

>EHO9888134.1 hypothetical protein KND05_003559 [Salmonella enterica subsp. enterica serovar Infantis] Overall Protective Antigen Prediction = **0.3383** (Probable **NON-ANTIGEN**).

>EHO9888135.1 sensor domain-containing phosphodiesterase [Salmonella enterica subsp. enterica serovar Infantis] Overall Protective Antigen Prediction = **0.5898** (Probable **ANTIGEN**).

>EHO9888136.1 exopolyphosphatase [Salmonella enterica subsp. enterica serovar Infantis] Overall Protective Antigen Prediction = **0.4444** (Probable **ANTIGEN**).

>EHO9888137.1 polyphosphate kinase 1 [Salmonella enterica subsp. enterica serovar Infantis] Overall Protective Antigen Prediction = **0.3177** (Probable **NON-ANTIGEN**).

>EHO9888138.1 phosphoribosylglycinamide formyltransferase [Salmonella enterica subsp. enterica serovar Infantis] Overall Protective Antigen Prediction = **0.5240** (Probable **ANTIGEN**).

>EHO9888139.1 phosphoribosylformylglycinamidine cyclo-ligase [Salmonella enterica subsp. enterica serovar Infantis] Overall Protective Antigen Prediction = **0.4522** (Probable **ANTIGEN**).

>EHO9888140.1 uracil phosphoribosyltransferase [Salmonella enterica subsp. enterica serovar Infantis] Overall Protective Antigen Prediction = **0.4343** (Probable **ANTIGEN**).

>EHO9888141.1 uracil permease [Salmonella enterica subsp. enterica serovar Infantis] Overall Protective Antigen Prediction = **0.4154** (Probable **ANTIGEN**).

>EHO9888142.1 DnaA inactivator Hda [Salmonella enterica subsp. enterica serovar Infantis] Overall Protective Antigen Prediction = **0.2464** (Probable **NON-ANTIGEN**).

>EHO9888143.1 arsenate reductase (glutaredoxin) [Salmonella enterica subsp. enterica serovar Infantis] Overall Protective Antigen Prediction = **0.3307** (Probable **NON-ANTIGEN**).

>EHO9888144.1 beta-barrel assembly-enhancing protease [Salmonella enterica subsp. enterica serovar Infantis] Overall Protective Antigen Prediction = **0.5287** (Probable **ANTIGEN**).

>EHO9888145.1 AI-2E family transporter [Salmonella enterica subsp. enterica serovar Infantis] Overall Protective Antigen Prediction = **0.4238** (Probable **ANTIGEN**).

>EHO9888146.1 helix-turn-helix domain-containing protein [Salmonella enterica subsp. enterica serovar Infantis] Overall Protective Antigen Prediction = **0.3558** (Probable **NON-ANTIGEN**).

>EHO9888147.1 GntP family permease [Salmonella enterica subsp. enterica serovar Infantis] Overall Protective Antigen Prediction = **0.4544** (Probable **ANTIGEN**).

>EHO9888148.1 glycerate kinase [Salmonella enterica subsp. enterica serovar Infantis] Overall Protective Antigen Prediction = **0.4672** (Probable **ANTIGEN**).

>EHO9888149.1 thioredoxin-dependent thiol peroxidase [Salmonella enterica subsp. enterica serovar Infantis] Overall Protective Antigen Prediction = **0.5143** (Probable **ANTIGEN**).

>EHO9888150.1 glycine cleavage system transcriptional repressor [Salmonella enterica subsp. enterica serovar Infantis] Overall Protective Antigen Prediction = **0.4651** (Probable **ANTIGEN**).

>EHO9888151.1 4-hydroxy-tetrahydrodipicolinate synthase [Salmonella enterica subsp. enterica serovar Infantis] Overall Protective Antigen Prediction = **0.3923** (Probable **NON-ANTIGEN**).

>EHO9888152.1 outer membrane protein assembly factor BamC [Salmonella enterica subsp. enterica serovar Infantis] Overall Protective Antigen Prediction = **0.5783** (Probable **ANTIGEN**).

>EHO9888153.1 phosphoribosylaminoimidazolesuccinocarboxamide synthase [Salmonella enterica subsp. enterica serovar Infantis] Overall Protective Antigen Prediction = **0.4150** (Probable **ANTIGEN**).

>EHO9888154.1 neutral zinc metallopeptidase [Salmonella enterica subsp. enterica serovar Infantis] Overall Protective Antigen Prediction = **0.6047** (Probable **ANTIGEN**).

>EHO9888155.1 tRNA cytosine(34) acetyltransferase TmcA [Salmonella enterica subsp. enterica serovar Infantis] Overall Protective Antigen Prediction = **0.3859** (Probable **NON-ANTIGEN**).

>EHO9888156.1 YpfN family protein [Salmonella enterica subsp. enterica serovar Infantis] Overall Protective Antigen Prediction = **0.4885** (Probable **ANTIGEN**).

>EHO9888157.1 succinyl-diaminopimelate desuccinylase [Salmonella enterica subsp. enterica serovar Infantis] Overall Protective Antigen Prediction = **0.4940** (Probable **ANTIGEN**).

>EHO9888158.1 ArsC family reductase [Salmonella enterica subsp. enterica serovar Infantis] Overall Protective Antigen Prediction = **0.5195** (Probable **ANTIGEN**).

>EHO9888159.1 protein YpfM [Salmonella enterica subsp. enterica serovar Infantis] Overall Protective Antigen Prediction = **-0.6793** (Probable **NON-ANTIGEN**).

>EHO9888160.1 multidrug efflux RND transporter permease AcrD [Salmonella enterica subsp. enterica serovar Infantis] Overall Protective Antigen Prediction = **0.6018** (Probable **ANTIGEN**).

>EHO9888161.1 nitrate/nitrite two-component system sensor histidine kinase NarQ [Salmonella enterica subsp. enterica serovar Infantis] Overall Protective Antigen Prediction = **0.4529** (Probable **ANTIGEN**).

>EHO9888162.1 formate-dependent uric acid utilization protein AegA [Salmonella enterica subsp. enterica serovar Infantis] Overall Protective Antigen Prediction = **0.5539** (Probable **ANTIGEN**).

>EHO9888163.1 MFS transporter, partial [Salmonella enterica subsp. enterica serovar Infantis] Overall Protective Antigen Prediction = **0.4213** (Probable **ANTIGEN**).

>EHO9888164.1 bifunctional UDP-sugar hydrolase/5'-nucleotidase [Salmonella enterica subsp. enterica serovar Infantis] Overall Protective Antigen Prediction = **0.4293** (Probable **ANTIGEN**).

>EHO9888165.1 Cys-tRNA(Pro)/Cys-tRNA(Cys) deacylase YbaK [Salmonella enterica subsp. enterica serovar Infantis] Overall Protective Antigen Prediction = **0.3913** (Probable **NON-ANTIGEN**).

>EHO9888166.1 TraB/GumN family protein [Salmonella enterica subsp. enterica serovar Infantis] Overall Protective Antigen Prediction = **0.3177** (Probable **NON-ANTIGEN**).

>EHO9888167.1 DUF1311 domain-containing protein [Salmonella enterica subsp. enterica serovar Infantis] Overall Protective Antigen Prediction = **0.5135** (Probable **ANTIGEN**).

>EHO9888168.1 copper-exporting P-type ATPase CopA [Salmonella enterica subsp. enterica serovar Infantis] Overall Protective Antigen Prediction = **0.5162** (Probable **ANTIGEN**).

>EHO9888169.1 Cu(I)-responsive transcriptional regulator [Salmonella enterica subsp. enterica serovar Infantis] Overall Protective Antigen Prediction = **0.3088** (Probable **NON-ANTIGEN**).

>EHO9888170.1 NfeD family protein [Salmonella enterica subsp. enterica serovar Infantis] Overall Protective Antigen Prediction = **0.8616** (Probable **ANTIGEN**).

>EHO9888171.1 SPFH/Band 7/PHB domain protein [Salmonella enterica subsp. enterica serovar Infantis] Overall Protective Antigen Prediction = **0.6021** (Probable **ANTIGEN**).

>EHO9888172.1 iron ABC transporter ATP-binding protein FetA [Salmonella enterica subsp. enterica serovar Infantis] Overall Protective Antigen Prediction = **0.3853** (Probable **NON-ANTIGEN**).

>EHO9888173.1 iron export ABC transporter permease subunit FetB [Salmonella enterica subsp. enterica serovar Infantis] Overall Protective Antigen Prediction = **0.4168** (Probable **ANTIGEN**).

>EHO9888174.1 chaperedoxin [Salmonella enterica subsp. enterica serovar Infantis] Overall Protective Antigen Prediction = **0.4749** (Probable **ANTIGEN**).

>EHO9888175.1 SDR family oxidoreductase [Salmonella enterica subsp. enterica serovar Infantis] Overall Protective Antigen Prediction = **0.4667** (Probable **ANTIGEN**).

>EHO9888176.1 multifunctional acyl-CoA thioesterase I/protease I/lysophospholipase L1 [Salmonella enterica subsp. enterica serovar Infantis] Overall Protective Antigen Prediction = **0.6979** (Probable **ANTIGEN**).

>EHO9888177.1 ABC transporter ATP-binding protein [Salmonella enterica subsp. enterica serovar Infantis] Overall Protective Antigen Prediction = **0.7059** (Probable **ANTIGEN**).

>EHO9888178.1 ABC transporter permease [Salmonella enterica subsp. enterica serovar Infantis] Overall Protective Antigen Prediction = **0.5329** (Probable **ANTIGEN**).

>EHO9888179.1 porin [Salmonella enterica subsp. enterica serovar Infantis] Overall Protective

Antigen Prediction = **0.7723** (Probable **ANTIGEN**).

>EHO9888180.1 metal ABC transporter substrate-binding protein [Salmonella enterica subsp. enterica serovar Infantis] Overall Protective Antigen Prediction = **0.2789** (Probable **NON-ANTIGEN**).

>EHO9888181.1 virulence-associated ABC transporter ATP-binding protein SfbB [Salmonella enterica subsp. enterica serovar Infantis] Overall Protective Antigen Prediction = **0.5550** (Probable **ANTIGEN**).

>EHO9888182.1 ABC transporter permease [Salmonella enterica subsp. enterica serovar Infantis] Overall Protective Antigen Prediction = **0.3679** (Probable **NON-ANTIGEN**).

>EHO9888183.1 tRNA 2-selenouridine(34) synthase MnmH [Salmonella enterica subsp. enterica serovar Infantis] Overall Protective Antigen Prediction = **0.5163** (Probable **ANTIGEN**).

>EHO9888184.1 HTH-type transcriptional activator AllS [Salmonella enterica subsp. enterica serovar Infantis] Overall Protective Antigen Prediction = **0.3677** (Probable **NON-ANTIGEN**).

>EHO9888185.1 ureidoglycolate lyase [Salmonella enterica subsp. enterica serovar Infantis] Overall Protective Antigen Prediction = **0.2521** (Probable **NON-ANTIGEN**).

>EHO9888186.1 HTH-type transcriptional repressor AllR [Salmonella enterica subsp. enterica serovar Infantis] Overall Protective Antigen Prediction = **0.4349** (Probable **ANTIGEN**).

>EHO9888187.1 glyoxylate carboligase [Salmonella enterica subsp. enterica serovar Infantis] Overall Protective Antigen Prediction = **0.4398** (Probable **ANTIGEN**).

>EHO9888188.1 hydroxypyruvate isomerase [Salmonella enterica subsp. enterica serovar Infantis] Overall Protective Antigen Prediction = **0.4550** (Probable **ANTIGEN**).

>EHO9888189.1 2-hydroxy-3-oxopropionate reductase [Salmonella enterica subsp. enterica serovar Infantis] Overall Protective Antigen Prediction = **0.6231** (Probable **ANTIGEN**).

>EHO9888190.1 MFS transporter [Salmonella enterica subsp. enterica serovar Infantis] Overall Protective Antigen Prediction = **0.4427** (Probable **ANTIGEN**).

>EHO9888191.1 putative allantoin permease [Salmonella enterica subsp. enterica serovar Infantis] Overall Protective Antigen Prediction = **0.4356** (Probable **ANTIGEN**).

>EHO9888192.1 allantoinase AllB [Salmonella enterica subsp. enterica serovar Infantis] Overall Protective Antigen Prediction = **0.3888** (Probable **NON-ANTIGEN**).

>EHO9888193.1 uracil/xanthine transporter [Salmonella enterica subsp. enterica serovar Infantis] Overall Protective Antigen Prediction = **0.4899** (Probable **ANTIGEN**).

>EHO9888194.1 glycerate 3-kinase [Salmonella enterica subsp. enterica serovar Infantis] Overall Protective Antigen Prediction = **0.6721** (Probable **ANTIGEN**).

>EHO9888195.1 (S)-ureidoglycine aminohydrolase [Salmonella enterica subsp. enterica serovar Infantis] Overall Protective Antigen Prediction = **0.5308** (Probable **ANTIGEN**).

>EHO9888196.1 allantoate deiminase [Salmonella enterica subsp. enterica serovar Infantis] Overall Protective Antigen Prediction = **0.5650** (Probable **ANTIGEN**).

>EHO9888197.1 ureidoglycolate dehydrogenase [Salmonella enterica subsp. enterica serovar Infantis] Overall Protective Antigen Prediction = **0.4884** (Probable **ANTIGEN**).

>EHO9888198.1 acyl-CoA synthetase FdrA [Salmonella enterica subsp. enterica serovar Infantis] Overall Protective Antigen Prediction = **0.4013** (Probable **ANTIGEN**).

>EHO9888199.1 DUF1116 domain-containing protein [Salmonella enterica subsp. enterica serovar Infantis] Overall Protective Antigen Prediction = **0.3707** (Probable **NON-ANTIGEN**).

>EHO9888200.1 DUF2877 domain-containing protein [Salmonella enterica subsp. enterica serovar Infantis] Overall Protective Antigen Prediction = **0.3354** (Probable **NON-ANTIGEN**).

>EHO9888201.1 carbamate kinase [Salmonella enterica subsp. enterica serovar Infantis] Overall Protective Antigen Prediction = **0.4226** (Probable **ANTIGEN**).

>EHO9888202.1 5-(carboxyamino)imidazole ribonucleotide synthase [Salmonella enterica subsp. enterica serovar Infantis] Overall Protective Antigen Prediction = **0.4100** (Probable **ANTIGEN**).

>EHO9888203.1 5-(carboxyamino)imidazole ribonucleotide mutase [Salmonella enterica subsp. enterica serovar Infantis] Overall Protective Antigen Prediction = **0.3779** (Probable **NON-ANTIGEN**).

>EHO9888204.1 UDP-2,3-diacylglucosamine diphosphatase [Salmonella enterica subsp. enterica serovar Infantis] Overall Protective Antigen Prediction = **0.4538** (Probable **ANTIGEN**).

>EHO9888205.1 peptidylprolyl isomerase B [Salmonella enterica subsp. enterica serovar Infantis] Overall Protective Antigen Prediction = **0.4078** (Probable **ANTIGEN**).

>EHO9888206.1 cysteine--tRNA ligase [Salmonella enterica subsp. enterica serovar Infantis] Overall Protective Antigen Prediction = **0.3810** (Probable **NON-ANTIGEN**).

>EHO9888207.1 DUF2145 domain-containing protein [Salmonella enterica subsp. enterica serovar Infantis] Overall Protective Antigen Prediction = **0.4700** (Probable **ANTIGEN**).

>EHO9888208.1 STM0539 family protein [Salmonella enterica subsp. enterica serovar Infantis] Overall Protective Antigen Prediction = **0.7260** (Probable **ANTIGEN**).

>EHO9888209.1 metal-dependent hydrolase [Salmonella enterica subsp. enterica serovar Infantis] Overall Protective Antigen Prediction = **0.6654** (Probable **ANTIGEN**).

>EHO9888210.1 ribosome-associated protein YbcJ [Salmonella enterica subsp. enterica serovar Infantis] Overall Protective Antigen Prediction = **0.6996** (Probable **ANTIGEN**).

>EHO9888211.1 bifunctional methylenetetrahydrofolate dehydrogenase/methenyltetrahydrofolate cyclohydrolase FolD [Salmonella enterica subsp. enterica serovar Infantis] Overall Protective Antigen Prediction = **0.4439** (Probable **ANTIGEN**).

>EHO9888212.1 type 1 fimbrial protein subunit FimA [Salmonella enterica subsp. enterica serovar Infantis] Overall Protective Antigen Prediction = **0.7420** (Probable **ANTIGEN**).

>EHO9888213.1 type 1 fimbrial protein subunit FimI [Salmonella enterica subsp. enterica serovar Infantis] Overall Protective Antigen Prediction = **0.4814** (Probable **ANTIGEN**).

>EHO9888214.1 type 1 fimbria chaperone FimC [Salmonella enterica subsp. enterica serovar Infantis] Overall Protective Antigen Prediction = **0.4616** (Probable **ANTIGEN**).

>EHO9888215.1 fimbrial biogenesis usher protein [Salmonella enterica subsp. enterica serovar Infantis] Overall Protective Antigen Prediction = **0.5327** (Probable **ANTIGEN**).

>EHO9888216.1 type 1 fimbrin D-mannose specific adhesin FimH [Salmonella enterica subsp. enterica serovar Infantis] Overall Protective Antigen Prediction = **0.6075** (Probable **ANTIGEN**).

>EHO9888217.1 fimbria assembly protein [Salmonella enterica subsp. enterica serovar Infantis] Overall Protective Antigen Prediction = **0.6827** (Probable **ANTIGEN**).

>EHO9888218.1 fimbria biosynthesis transcriptional regulator FimZ [Salmonella enterica subsp. enterica serovar Infantis] Overall Protective Antigen Prediction = **0.2051** (Probable **NON-ANTIGEN**).

>EHO9888219.1 fimbria biosynthesis regulator FimY [Salmonella enterica subsp. enterica serovar Infantis] Overall Protective Antigen Prediction = **0.1754** (Probable **NON-ANTIGEN**).

>EHO9888220.1 diguanylate cyclase [Salmonella enterica subsp. enterica serovar Infantis] Overall Protective Antigen Prediction = **0.4300** (Probable **ANTIGEN**).

>EHO9888221.1 fimbria biosynthesis transcriptional regulator FimW [Salmonella enterica subsp. enterica serovar Infantis] Overall Protective Antigen Prediction = **0.4499** (Probable **ANTIGEN**).

>EHO9888222.1 hypothetical protein KND05_003650 [Salmonella enterica subsp. enterica serovar Infantis] Overall Protective Antigen Prediction = **0.4444** (Probable **ANTIGEN**).

>EHO9888223.1 hypothetical protein KND05_003652 [Salmonella enterica subsp. enterica serovar Infantis] Overall Protective Antigen Prediction = **0.6472** (Probable **ANTIGEN**).

>EHO9888224.1 oxygen-dependent coproporphyrinogen oxidase [Salmonella enterica subsp. enterica serovar Infantis] Overall Protective Antigen Prediction = **0.4174** (Probable **ANTIGEN**).

>EHO9888225.1 N-acetylmuramoyl-L-alanine amidase AmiA [Salmonella enterica subsp. enterica serovar Infantis] Overall Protective Antigen Prediction = **0.5270** (Probable **ANTIGEN**).

>EHO9888226.1 GNAT family acetyltransferase [Salmonella enterica subsp. enterica serovar Infantis] Overall Protective Antigen Prediction = **0.3243** (Probable **NON-ANTIGEN**).

>EHO9888227.1 DUF2919 domain-containing protein [Salmonella enterica subsp. enterica serovar Infantis] Overall Protective Antigen Prediction = **0.6577** (Probable **ANTIGEN**).

>EHO9888228.1 RpoE-regulated lipoprotein [Salmonella enterica subsp. enterica serovar Infantis] Overall Protective Antigen Prediction = **0.5635** (Probable **ANTIGEN**).

>EHO9888229.1 porphyrinogen peroxidase [Salmonella enterica subsp. enterica serovar Infantis] Overall Protective Antigen Prediction = **0.5391** (Probable **ANTIGEN**).

>EHO9888230.1 SDR family oxidoreductase UcpA [Salmonella enterica subsp. enterica serovar Infantis] Overall Protective Antigen Prediction = **0.3922** (Probable **NON-ANTIGEN**).

>EHO9888231.1 thiosulfate/sulfate ABC transporter substrate-binding protein CysP [Salmonella enterica subsp. enterica serovar Infantis] Overall Protective Antigen Prediction = **0.4929** (Probable

ANTIGEN).

>EHO9888232.1 sulfate/thiosulfate ABC transporter permease CysT [Salmonella enterica subsp. enterica serovar Infantis] Overall Protective Antigen Prediction = **0.4605** (Probable **ANTIGEN**).

>EHO9888233.1 sulfate/thiosulfate ABC transporter permease CysW [Salmonella enterica subsp. enterica serovar Infantis] Overall Protective Antigen Prediction = **0.5227** (Probable **ANTIGEN**).

>EHO9888234.1 sulfate/thiosulfate ABC transporter ATP-binding protein CysA [Salmonella enterica subsp. enterica serovar Infantis] Overall Protective Antigen Prediction = **0.4043** (Probable **ANTIGEN**).

>EHO9888235.1 cysteine synthase CysM [Salmonella enterica subsp. enterica serovar Infantis] Overall Protective Antigen Prediction = **0.4559** (Probable **ANTIGEN**).

>EHO9888236.1 sel1 repeat family protein [Salmonella enterica subsp. enterica serovar Infantis] Overall Protective Antigen Prediction = **0.5990** (Probable **ANTIGEN**).

>EHO9888237.1 YfeK family protein [Salmonella enterica subsp. enterica serovar Infantis] Overall Protective Antigen Prediction = **0.5932** (Probable **ANTIGEN**).

>EHO9888238.1 GMP synthase [Salmonella enterica subsp. enterica serovar Infantis] Overall Protective Antigen Prediction = **0.2934** (Probable **NON-ANTIGEN**).

>EHO9888239.1 transcriptional regulator PtsJ [Salmonella enterica subsp. enterica serovar Infantis] Overall Protective Antigen Prediction = **0.4846** (Probable **ANTIGEN**).

>EHO9888240.1 pyridoxine/pyridoxal/pyridoxamine kinase [Salmonella enterica subsp. enterica serovar Infantis] Overall Protective Antigen Prediction = **0.4323** (Probable **ANTIGEN**).

>EHO9888241.1 cytoplasmic protein [Salmonella enterica subsp. enterica serovar Infantis] Overall Protective Antigen Prediction = **0.2986** (Probable **NON-ANTIGEN**).

>EHO9888242.1 PTS glucose transporter subunit IIA [Salmonella enterica subsp. enterica serovar Infantis] Overall Protective Antigen Prediction = **0.4968** (Probable **ANTIGEN**).

>EHO9888243.1 phosphoenolpyruvate-protein phosphotransferase PtsI [Salmonella enterica subsp. enterica serovar Infantis] Overall Protective Antigen Prediction = **0.3473** (Probable **NON-ANTIGEN**).

>EHO9888244.1 phosphocarrier protein Hpr [Salmonella enterica subsp. enterica serovar Infantis] Overall Protective Antigen Prediction = **0.5761** (Probable **ANTIGEN**).

>EHO9888245.1 cysteine synthase A [Salmonella enterica subsp. enterica serovar Infantis] Overall Protective Antigen Prediction = **0.4818** (Probable **ANTIGEN**).

>EHO9888246.1 sulfate transporter CysZ [Salmonella enterica subsp. enterica serovar Infantis] Overall Protective Antigen Prediction = **0.5714** (Probable **ANTIGEN**).

>EHO9888247.1 cell division protein ZipA [Salmonella enterica subsp. enterica serovar Infantis] Overall Protective Antigen Prediction = **0.4494** (Probable **ANTIGEN**).

>EHO9888248.1 NAD-dependent DNA ligase LigA [Salmonella enterica subsp. enterica serovar Infantis] Overall Protective Antigen Prediction = **0.4713** (Probable **ANTIGEN**).

>EHO9888249.1 DUF3820 family protein [Salmonella enterica subsp. enterica serovar Infantis] Overall Protective Antigen Prediction = **-0.3868** (Probable **NON-ANTIGEN**).

>EHO9888250.1 bile acid:sodium symporter [Salmonella enterica subsp. enterica serovar Infantis] Overall Protective Antigen Prediction = **0.5310** (Probable **ANTIGEN**).

>EHO9888251.1 LysR family transcriptional regulator [Salmonella enterica subsp. enterica serovar Infantis] Overall Protective Antigen Prediction = **0.3932** (Probable **NON-ANTIGEN**).

>EHO9888252.1 outer membrane protein [Salmonella enterica subsp. enterica serovar Infantis] Overall Protective Antigen Prediction = **0.5878** (Probable **ANTIGEN**).

>EHO9888253.1 xanthosine phosphorylase [Salmonella enterica subsp. enterica serovar Infantis] Overall Protective Antigen Prediction = **0.4430** (Probable **ANTIGEN**).

>EHO9888254.1 nucleoside permease [Salmonella enterica subsp. enterica serovar Infantis] Overall Protective Antigen Prediction = **0.5256** (Probable **ANTIGEN**).

>EHO9888255.1 DUF1427 family protein [Salmonella enterica subsp. enterica serovar Infantis] Overall Protective Antigen Prediction = **0.3225** (Probable **NON-ANTIGEN**).

>EHO9888256.1 LysR family transcriptional regulator [Salmonella enterica subsp. enterica serovar Infantis] Overall Protective Antigen Prediction = **0.4021** (Probable **ANTIGEN**).

>EHO9888257.1 glutamate--tRNA ligase [Salmonella enterica subsp. enterica serovar Infantis] Overall Protective Antigen Prediction = **0.4606** (Probable **ANTIGEN**).

>EHO9888258.1 putative DNA-binding transcriptional regulator [Salmonella enterica subsp. enterica serovar Infantis] Overall Protective Antigen Prediction = **0.3348** (Probable **NON-ANTIGEN**).

>EHO9888259.1 putative DNA-binding transcriptional regulator [Salmonella enterica subsp. enterica serovar Infantis] Overall Protective Antigen Prediction = **0.3920** (Probable **NON-ANTIGEN**).

>EHO9888260.1 sensor domain-containing phosphodiesterase [Salmonella enterica subsp. enterica serovar Infantis] Overall Protective Antigen Prediction = **0.4883** (Probable **ANTIGEN**).

>EHO9888261.1 nucleoside permease NupC [Salmonella enterica subsp. enterica serovar Infantis] Overall Protective Antigen Prediction = **0.3716** (Probable **NON-ANTIGEN**).

>EHO9888262.1 Nramp family divalent metal transporter [Salmonella enterica subsp. enterica serovar Infantis] Overall Protective Antigen Prediction = **0.5031** (Probable **ANTIGEN**).

>EHO9888263.1 DUF2502 domain-containing protein [Salmonella enterica subsp. enterica serovar Infantis] Overall Protective Antigen Prediction = **0.5111** (Probable **ANTIGEN**).

>EHO9888264.1 alpha-keto acid decarboxylase family protein [Salmonella enterica subsp. enterica serovar Infantis] Overall Protective Antigen Prediction = **0.3456** (Probable **NON-ANTIGEN**).

>EHO9888265.1 ion channel protein [Salmonella enterica subsp. enterica serovar Infantis] Overall Protective Antigen Prediction = **0.4220** (Probable **ANTIGEN**).

>EHO9888266.1 glucokinase [Salmonella enterica subsp. enterica serovar Infantis] Overall Protective Antigen Prediction = **0.4829** (Probable **ANTIGEN**).

>EHO9888267.1 alanine transaminase [Salmonella enterica subsp. enterica serovar Infantis] Overall Protective Antigen Prediction = **0.3284** (Probable **NON-ANTIGEN**).

>EHO9888268.1 membrane protein YpdK [Salmonella enterica subsp. enterica serovar Infantis] Overall Protective Antigen Prediction = **0.9584** (Probable **ANTIGEN**).

>EHO9888269.1 kdo(2)-lipid IV(A) palmitoleoyltransferase [Salmonella enterica subsp. enterica serovar Infantis] Overall Protective Antigen Prediction = **0.3003** (Probable **NON-ANTIGEN**).

>EHO9888270.1 YfdY family protein [Salmonella enterica subsp. enterica serovar Infantis] Overall Protective Antigen Prediction = **0.6192** (Probable **ANTIGEN**).

>EHO9888271.1 phosphoglycerate transporter PgtP [Salmonella enterica subsp. enterica serovar Infantis] Overall Protective Antigen Prediction = **0.4511** (Probable **ANTIGEN**).

>EHO9888272.1 phosphoglycerate transport regulator PgtC [Salmonella enterica subsp. enterica serovar Infantis] Overall Protective Antigen Prediction = **0.3599** (Probable **NON-ANTIGEN**).

>EHO9888273.1 two-component system sensor histidine kinase PgtB [Salmonella enterica subsp. enterica serovar Infantis] Overall Protective Antigen Prediction = **0.3906** (Probable **NON-ANTIGEN**).

>EHO9888274.1 two-component system response regulator PgtA [Salmonella enterica subsp. enterica serovar Infantis] Overall Protective Antigen Prediction = **0.3038** (Probable **NON-ANTIGEN**).

>EHO9888275.1 omptin family outer membrane protease PgtE [Salmonella enterica subsp. enterica serovar Infantis] Overall Protective Antigen Prediction = **0.6079** (Probable **ANTIGEN**).

>EHO9888276.1 hypothetical protein KND05_003712 [Salmonella enterica subsp. enterica serovar Infantis] Overall Protective Antigen Prediction = **0.2012** (Probable **NON-ANTIGEN**).

>EHO9888277.1 hypothetical protein KND05_003713 [Salmonella enterica subsp. enterica serovar Infantis] Overall Protective Antigen Prediction = **0.3667** (Probable **NON-ANTIGEN**).

>EHO9888278.1 hypothetical protein KND05_003716 [Salmonella enterica subsp. enterica serovar Infantis] Overall Protective Antigen Prediction = **0.3335** (Probable **NON-ANTIGEN**).

>EHO9888279.1 ParB/RepB/Spo0J family partition protein [Salmonella enterica subsp. enterica serovar Infantis] Overall Protective Antigen Prediction = **0.2394** (Probable **NON-ANTIGEN**).

>EHO9888280.1 D-glycero-beta-D-manno-heptose 1,7-bisphosphate 7-phosphatase [Salmonella enterica subsp. enterica serovar Infantis] Overall Protective Antigen Prediction = **0.2290** (Probable **NON-ANTIGEN**).

>EHO9888281.1 methionine ABC transporter ATP-binding protein MetN [Salmonella enterica subsp. enterica serovar Infantis] Overall Protective Antigen Prediction = **0.4145** (Probable **ANTIGEN**).

>EHO9888282.1 methionine ABC transporter permease MetI [Salmonella enterica subsp. enterica serovar Infantis] Overall Protective Antigen Prediction = **0.3596** (Probable **NON-ANTIGEN**).

>EHO9888283.1 methionine ABC transporter substrate-binding lipoprotein MetQ [Salmonella enterica subsp. enterica serovar Infantis] Overall Protective Antigen Prediction = **0.3539** (Probable **NON-ANTIGEN**).

>EHO9888284.1 Rcs stress response system protein RcsF [Salmonella enterica subsp. enterica serovar Infantis] Overall Protective Antigen Prediction = **0.5808** (Probable **ANTIGEN**).

>EHO9888285.1 tRNA (N6-threonylcarbamoyladenosine(37)-N6)-methyltransferase TrmO [Salmonella enterica subsp. enterica serovar Infantis] Overall Protective Antigen Prediction = **0.5831** (Probable **ANTIGEN**).

>EHO9888286.1 proline--tRNA ligase [Salmonella enterica subsp. enterica serovar Infantis] Overall Protective Antigen Prediction = **0.3975** (Probable **NON-ANTIGEN**).

>EHO9888287.1 envelope stress response activation lipoprotein NlpE [Salmonella enterica subsp. enterica serovar Infantis] Overall Protective Antigen Prediction = **0.4832** (Probable **ANTIGEN**).

>EHO9888288.1 aminoacyl-tRNA hydrolase [Salmonella enterica subsp. enterica serovar Infantis] Overall Protective Antigen Prediction = **0.6712** (Probable **ANTIGEN**).

>EHO9888289.1 YaeQ family protein [Salmonella enterica subsp. enterica serovar Infantis] Overall Protective Antigen Prediction = **0.3528** (Probable **NON-ANTIGEN**).

>EHO9888290.1 YaeP family protein [Salmonella enterica subsp. enterica serovar Infantis] Overall Protective Antigen Prediction = **0.4262** (Probable **ANTIGEN**).

>EHO9888291.1 Rho-binding antiterminator [Salmonella enterica subsp. enterica serovar Infantis] Overall Protective Antigen Prediction = **0.5414** (Probable **ANTIGEN**).

>EHO9888292.1 tRNA lysidine(34) synthetase TilS [Salmonella enterica subsp. enterica serovar Infantis] Overall Protective Antigen Prediction = **0.3232** (Probable **NON-ANTIGEN**).

>EHO9888293.1 VOC family protein [Salmonella enterica subsp. enterica serovar Infantis] Overall Protective Antigen Prediction = **0.5036** (Probable **ANTIGEN**).

>EHO9888294.1 lysine decarboxylase LdcC [Salmonella enterica subsp. enterica serovar Infantis] Overall Protective Antigen Prediction = **0.4607** (Probable **ANTIGEN**).

>EHO9888295.1 chitinase [Salmonella enterica subsp. enterica serovar Infantis] Overall Protective Antigen Prediction = **0.5252** (Probable **ANTIGEN**).

>EHO9888296.1 acetyl-CoA carboxylase carboxyl transferase subunit alpha [Salmonella enterica subsp. enterica serovar Infantis] Overall Protective Antigen Prediction = **0.4965** (Probable **ANTIGEN**).

>EHO9888297.1 DNA polymerase III subunit alpha [Salmonella enterica subsp. enterica serovar Infantis] Overall Protective Antigen Prediction = **0.4055** (Probable **ANTIGEN**).

>EHO9888298.1 ribonuclease HII [Salmonella enterica subsp. enterica serovar Infantis] Overall Protective Antigen Prediction = **0.3089** (Probable **NON-ANTIGEN**).

>EHO9888299.1 lipid-A-disaccharide synthase [Salmonella enterica subsp. enterica serovar Infantis] Overall Protective Antigen Prediction = **0.4005** (Probable **ANTIGEN**).

>EHO9888300.1 acyl-ACP--UDP-N-acetylglucosamine O-acyltransferase [Salmonella enterica subsp. enterica serovar Infantis] Overall Protective Antigen Prediction = **0.5355** (Probable **ANTIGEN**).

>EHO9888301.1 3-hydroxyacyl-ACP dehydratase FabZ [Salmonella enterica subsp. enterica serovar Infantis] Overall Protective Antigen Prediction = **0.2511** (Probable **NON-ANTIGEN**).

>EHO9888302.1 UDP-3-O-(3-hydroxymyristoyl)glucosamine N-acyltransferase [Salmonella enterica subsp. enterica serovar Infantis] Overall Protective Antigen Prediction = **0.4691** (Probable **ANTIGEN**).

>EHO9888303.1 molecular chaperone Skp [Salmonella enterica subsp. enterica serovar Infantis] Overall Protective Antigen Prediction = **0.5595** (Probable **ANTIGEN**).

>EHO9888304.1 outer membrane protein assembly factor BamA [Salmonella enterica subsp. enterica serovar Infantis] Overall Protective Antigen Prediction = **0.5842** (Probable **ANTIGEN**).

>EHO9888305.1 sigma E protease regulator RseP [Salmonella enterica subsp. enterica serovar Infantis] Overall Protective Antigen Prediction = **0.5866** (Probable **ANTIGEN**).

>EHO9888306.1 phosphatidate cytidylyltransferase [Salmonella enterica subsp. enterica serovar Infantis] Overall Protective Antigen Prediction = **0.5268** (Probable **ANTIGEN**).

>EHO9888307.1 (2E,6E)-farnesyl-diphosphate-specific ditrans,polycis-undecaprenyl-diphosphate synthase [Salmonella enterica subsp. enterica serovar Infantis] Overall Protective Antigen Prediction = **0.5340** (Probable **ANTIGEN**).

>EHO9888308.1 1-deoxy-D-xylulose-5-phosphate reductoisomerase [Salmonella enterica subsp. enterica serovar Infantis] Overall Protective Antigen Prediction = **0.3799** (Probable **NON-ANTIGEN**).

>EHO9888309.1 ribosome recycling factor [Salmonella enterica subsp. enterica serovar Infantis] Overall Protective Antigen Prediction = **0.6526** (Probable **ANTIGEN**).

>EHO9888310.1 UMP kinase [Salmonella enterica subsp. enterica serovar Infantis] Overall Protective Antigen Prediction = **0.4539** (Probable **ANTIGEN**).

>EHO9888311.1 elongation factor Ts [Salmonella enterica subsp. enterica serovar Infantis] Overall Protective Antigen Prediction = **0.6174** (Probable **ANTIGEN**).

>EHO9888312.1 30S ribosomal protein S2 [Salmonella enterica subsp. enterica serovar Infantis] Overall Protective Antigen Prediction = **0.3101** (Probable **NON-ANTIGEN**).

>EHO9888313.1 type I methionyl aminopeptidase [Salmonella enterica subsp. enterica serovar Infantis] Overall Protective Antigen Prediction = **0.6114** (Probable **ANTIGEN**).

>EHO9888314.1 bifunctional uridylyltransferase/uridylyl-removing protein GlnD [Salmonella enterica subsp. enterica serovar Infantis] Overall Protective Antigen Prediction = **0.3957** (Probable **NON-ANTIGEN**).

>EHO9888315.1 2,3,4,5-tetrahydropyridine-2,6-dicarboxylate N-succinyltransferase [Salmonella enterica subsp. enterica serovar Infantis] Overall Protective Antigen Prediction = **0.4969** (Probable **ANTIGEN**).

>EHO9888316.1 MHS family MFS transporter [Salmonella enterica subsp. enterica serovar Infantis] Overall Protective Antigen Prediction = **0.4398** (Probable **ANTIGEN**).

>EHO9888317.1 DUF3461 family protein [Salmonella enterica subsp. enterica serovar Infantis]

Overall Protective Antigen Prediction = **0.2607** (Probable **NON-ANTIGEN**).

>EHO9888318.1 DNA-binding transcriptional regulator CdaR [Salmonella enterica subsp. enterica serovar Infantis] Overall Protective Antigen Prediction = **0.2833** (Probable **NON-ANTIGEN**).

>EHO9888319.1 serine endoprotease DegP [Salmonella enterica subsp. enterica serovar Infantis] Overall Protective Antigen Prediction = **0.5514** (Probable **ANTIGEN**).

>EHO9888320.1 dGTPase [Salmonella enterica subsp. enterica serovar Infantis] Overall Protective Antigen Prediction = **0.1674** (Probable **NON-ANTIGEN**).

>EHO9888321.1 5'-methylthioadenosine/S-adenosylhomocysteine nucleosidase [Salmonella enterica subsp. enterica serovar Infantis] Overall Protective Antigen Prediction = **0.3801** (Probable **NON-ANTIGEN**).

>EHO9888322.1 vitamin B12 ABC transporter substrate-binding protein BtuF [Salmonella enterica subsp. enterica serovar Infantis] Overall Protective Antigen Prediction = **0.4002** (Probable **ANTIGEN**).

>EHO9888323.1 TRIC cation channel family protein [Salmonella enterica subsp. enterica serovar Infantis] Overall Protective Antigen Prediction = **0.4078** (Probable **ANTIGEN**).

>EHO9888324.1 iron-sulfur cluster insertion protein ErpA [Salmonella enterica subsp. enterica serovar Infantis] Overall Protective Antigen Prediction = **0.9959** (Probable **ANTIGEN**).

>EHO9888325.1 H(+)/Cl(-) exchange transporter ClcA [Salmonella enterica subsp. enterica serovar Infantis] Overall Protective Antigen Prediction = **0.4488** (Probable **ANTIGEN**).

>EHO9888326.1 glutamate-1-semialdehyde 2,1-aminomutase [Salmonella enterica subsp. enterica serovar Infantis] Overall Protective Antigen Prediction = **0.2632** (Probable **NON-ANTIGEN**).

>EHO9888327.1 YfcO family protein [Salmonella enterica subsp. enterica serovar Infantis] Overall Protective Antigen Prediction = **0.6968** (Probable **ANTIGEN**).

>EHO9888328.1 fimbrial protein [Salmonella enterica subsp. enterica serovar Infantis] Overall Protective Antigen Prediction = **0.5050** (Probable **ANTIGEN**).

>EHO9888329.1 fimbrial minor subunit StfF [Salmonella enterica subsp. enterica serovar Infantis] Overall Protective Antigen Prediction = **0.7518** (Probable **ANTIGEN**).

>EHO9888330.1 fimbrial protein [Salmonella enterica subsp. enterica serovar Infantis] Overall Protective Antigen Prediction = **0.9618** (Probable **ANTIGEN**).

>EHO9888331.1 fimbrial chaperone StfD [Salmonella enterica subsp. enterica serovar Infantis] Overall Protective Antigen Prediction = **0.5293** (Probable **ANTIGEN**).

>EHO9888332.1 fimbrial biogenesis outer membrane usher protein [Salmonella enterica subsp. enterica serovar Infantis] Overall Protective Antigen Prediction = **0.6321** (Probable **ANTIGEN**).

>EHO9888333.1 fimbrial major subunit StfA [Salmonella enterica subsp. enterica serovar Infantis] Overall Protective Antigen Prediction = **0.7617** (Probable **ANTIGEN**).

>EHO9888334.1 DNA-binding protein YbiB, partial [Salmonella enterica subsp. enterica serovar Infantis] Overall Protective Antigen Prediction = **0.5488** (Probable **ANTIGEN**).

>EHO9888335.1 ATP-dependent DNA helicase DinG [Salmonella enterica subsp. enterica serovar Infantis] Overall Protective Antigen Prediction = **0.3547** (Probable **NON-ANTIGEN**).

>EHO9888336.1 ATP-dependent RNA helicase RhlE [Salmonella enterica subsp. enterica serovar Infantis] Overall Protective Antigen Prediction = **0.6370** (Probable **ANTIGEN**).

>EHO9888337.1 transcriptional regulator CecR [Salmonella enterica subsp. enterica serovar Infantis] Overall Protective Antigen Prediction = **0.2635** (Probable **NON-ANTIGEN**).

>EHO9888338.1 secretion protein HlyD [Salmonella enterica subsp. enterica serovar Infantis] Overall Protective Antigen Prediction = **0.5139** (Probable **ANTIGEN**).

>EHO9888339.1 ABC transporter ATP-binding protein [Salmonella enterica subsp. enterica serovar Infantis] Overall Protective Antigen Prediction = **0.4484** (Probable **ANTIGEN**).

>EHO9888340.1 ABC transporter permease [Salmonella enterica subsp. enterica serovar Infantis] Overall Protective Antigen Prediction = **0.4493** (Probable **ANTIGEN**).

>EHO9888341.1 ABC transporter permease [Salmonella enterica subsp. enterica serovar Infantis] Overall Protective Antigen Prediction = **0.4032** (Probable **ANTIGEN**).

>EHO9888342.1 YbhQ family protein [Salmonella enterica subsp. enterica serovar Infantis] Overall Protective Antigen Prediction = **0.3343** (Probable **NON-ANTIGEN**).

>EHO9888343.1 endonuclease/exonuclease/phosphatase family protein [Salmonella enterica subsp. enterica serovar Infantis] Overall Protective Antigen Prediction = **0.4549** (Probable **ANTIGEN**).

>EHO9888344.1 cardiolipin synthase ClsB [Salmonella enterica subsp. enterica serovar Infantis] Overall Protective Antigen Prediction = **0.4663** (Probable **ANTIGEN**).

>EHO9888345.1 UPF0104 family protein [Salmonella enterica subsp. enterica serovar Infantis] Overall Protective Antigen Prediction = **0.6016** (Probable **ANTIGEN**).

>EHO9888346.1 hypothetical protein KND05_003785 [Salmonella enterica subsp. enterica serovar Infantis] Overall Protective Antigen Prediction = **0.3559** (Probable **NON-ANTIGEN**).

>EHO9888347.1 hypothetical protein KND05_003786 [Salmonella enterica subsp. enterica serovar Infantis] Overall Protective Antigen Prediction = **0.6604** (Probable **ANTIGEN**).

>EHO9888348.1 Bax inhibitor-1/YccA family protein [Salmonella enterica subsp. enterica serovar Infantis] Overall Protective Antigen Prediction = **0.3925** (Probable **NON-ANTIGEN**).

>EHO9888349.1 Bax inhibitor-1/YccA family protein [Salmonella enterica subsp. enterica serovar Infantis] Overall Protective Antigen Prediction = **0.3700** (Probable **NON-ANTIGEN**).

>EHO9888350.1 molybdopterin synthase catalytic subunit MoaE [Salmonella enterica subsp. enterica serovar Infantis] Overall Protective Antigen Prediction = **0.1627** (Probable **NON-ANTIGEN**).

>EHO9888351.1 molybdopterin synthase sulfur carrier subunit [Salmonella enterica subsp. enterica serovar Infantis] Overall Protective Antigen Prediction = **0.4796** (Probable **ANTIGEN**).

>EHO9888352.1 cyclic pyranopterin monophosphate synthase MoaC [Salmonella enterica subsp. enterica serovar Infantis] Overall Protective Antigen Prediction = **0.6204** (Probable **ANTIGEN**).

>EHO9888353.1 molybdenum cofactor biosynthesis protein B [Salmonella enterica subsp. enterica serovar Infantis] Overall Protective Antigen Prediction = **0.6423** (Probable **ANTIGEN**).

>EHO9888354.1 GTP 3',8-cyclase MoaA [Salmonella enterica subsp. enterica serovar Infantis] Overall Protective Antigen Prediction = **0.4750** (Probable **ANTIGEN**).

>EHO9888355.1 uridine diphosphate-N-acetylglucosamine-binding protein YvcK [Salmonella enterica subsp. enterica serovar Infantis] Overall Protective Antigen Prediction = **0.3607** (Probable **NON-ANTIGEN**).

>EHO9888356.1 SPI-1 type III secretion system effector E3 ubiquitin transferase SlrP [Salmonella enterica subsp. enterica serovar Infantis] Overall Protective Antigen Prediction = **0.4688** (Probable **ANTIGEN**).

>EHO9888357.1 excinuclease ABC subunit B [Salmonella enterica subsp. enterica serovar Infantis] Overall Protective Antigen Prediction = **0.3815** (Probable **NON-ANTIGEN**).

>EHO9888358.1 ATP-dependent dethiobiotin synthetase BioD [Salmonella enterica subsp. enterica serovar Infantis] Overall Protective Antigen Prediction = **0.4236** (Probable **ANTIGEN**).

>EHO9888359.1 malonyl-ACP O-methyltransferase BioC [Salmonella enterica subsp. enterica serovar Infantis] Overall Protective Antigen Prediction = **0.3599** (Probable **NON-ANTIGEN**).

>EHO9888360.1 8-amino-7-oxononanoate synthase [Salmonella enterica subsp. enterica serovar Infantis] Overall Protective Antigen Prediction = **0.3946** (Probable **NON-ANTIGEN**).

>EHO9888361.1 biotin synthase BioB [Salmonella enterica subsp. enterica serovar Infantis] Overall Protective Antigen Prediction = **0.3300** (Probable **NON-ANTIGEN**).

>EHO9888362.1 adenosylmethionine--8-amino-7-oxononanoate transaminase [Salmonella enterica subsp. enterica serovar Infantis] Overall Protective Antigen Prediction = **0.2729** (Probable **NON-ANTIGEN**).

>EHO9888363.1 kinase inhibitor [Salmonella enterica subsp. enterica serovar Infantis] Overall Protective Antigen Prediction = **0.6231** (Probable **ANTIGEN**).

>EHO9888364.1 histidine ammonia-lyase [Salmonella enterica subsp. enterica serovar Infantis] Overall Protective Antigen Prediction = **0.3781** (Probable **NON-ANTIGEN**).

>EHO9888365.1 urocanate hydratase [Salmonella enterica subsp. enterica serovar Infantis] Overall Protective Antigen Prediction = **0.4750** (Probable **ANTIGEN**).

>EHO9888366.1 histidine utilization repressor [Salmonella enterica subsp. enterica serovar Infantis] Overall Protective Antigen Prediction = **0.2809** (Probable **NON-ANTIGEN**).

>EHO9888367.1 formimidoylglutamase [Salmonella enterica subsp. enterica serovar Infantis] Overall Protective Antigen Prediction = **0.5019** (Probable **ANTIGEN**).

>EHO9888368.1 imidazolonepropionase [Salmonella enterica subsp. enterica serovar Infantis] Overall Protective Antigen Prediction = **0.3513** (Probable **NON-ANTIGEN**).

>EHO9888369.1 hypothetical protein KND05_003810 [Salmonella enterica subsp. enterica serovar Infantis] Overall Protective Antigen Prediction = **0.5459** (Probable **ANTIGEN**).

>EHO9888370.1 DNA transfer protein [Salmonella enterica subsp. enterica serovar Infantis] Overall Protective Antigen Prediction = **0.4624** (Probable **ANTIGEN**).

>EHO9888371.1 phage DNA ejection protein [Salmonella enterica subsp. enterica serovar Infantis] Overall Protective Antigen Prediction = **0.6496** (Probable **ANTIGEN**).

>EHO9888372.1 DNA transfer protein [Salmonella enterica subsp. enterica serovar Infantis] Overall Protective Antigen Prediction = **0.7292** (Probable **ANTIGEN**).

>EHO9888373.1 DUF2824 family protein [Salmonella enterica subsp. enterica serovar Infantis] Overall Protective Antigen Prediction = **0.3934** (Probable **NON-ANTIGEN**).

>EHO9888374.1 phage tail protein [Salmonella enterica subsp. enterica serovar Infantis] Overall Protective Antigen Prediction = **0.7305** (Probable **ANTIGEN**).

>EHO9888375.1 packaged DNA stabilization protein gp10 [Salmonella enterica subsp. enterica serovar Infantis] Overall Protective Antigen Prediction = **0.4286** (Probable **ANTIGEN**).

>EHO9888376.1 packaged DNA stabilization gp4 family protein [Salmonella enterica subsp. enterica serovar Infantis] Overall Protective Antigen Prediction = **0.4988** (Probable **ANTIGEN**).

>EHO9888377.1 hypothetical protein KND05_003818 [Salmonella enterica subsp. enterica serovar Infantis] Overall Protective Antigen Prediction = **0.5283** (Probable **ANTIGEN**).

>EHO9888378.1 coat protein [Salmonella enterica subsp. enterica serovar Infantis] Overall Protective Antigen Prediction = **0.4413** (Probable **ANTIGEN**).

>EHO9888379.1 scaffolding protein [Salmonella enterica subsp. enterica serovar Infantis] Overall Protective Antigen Prediction = **0.6432** (Probable **ANTIGEN**).

>EHO9888380.1 portal protein [Salmonella enterica subsp. enterica serovar Infantis] Overall Protective Antigen Prediction = **0.3868** (Probable **NON-ANTIGEN**).

>EHO9888381.1 terminase large subunit [Salmonella enterica subsp. enterica serovar Infantis] Overall Protective Antigen Prediction = **0.3663** (Probable **NON-ANTIGEN**).

>EHO9888382.1 DNA-packaging protein [Salmonella enterica subsp. enterica serovar Infantis] Overall Protective Antigen Prediction = **0.5622** (Probable **ANTIGEN**).

>EHO9888383.1 Decoration protein [Salmonella enterica subsp. enterica serovar Infantis] Overall Protective Antigen Prediction = **0.7769** (Probable **ANTIGEN**).

>EHO9888384.1 DUF2560 family protein [Salmonella enterica subsp. enterica serovar Infantis] Overall Protective Antigen Prediction = **0.7767** (Probable **ANTIGEN**).

>EHO9888385.1 KilA-N domain-containing protein, partial [Salmonella enterica subsp. enterica serovar Infantis] Overall Protective Antigen Prediction = **0.6636** (Probable **ANTIGEN**).

>EHO9888386.1 flagellin FliC, partial [Salmonella enterica subsp. enterica serovar Infantis] Overall Protective Antigen Prediction = **0.6378** (Probable **ANTIGEN**).

>EHO9888387.1 flagellar phase variation DNA invertase Hin [Salmonella enterica subsp. enterica serovar Infantis] Overall Protective Antigen Prediction = **0.3716** (Probable **NON-ANTIGEN**).

>EHO9888388.1 salmochelin biosynthesis C-glycosyltransferase IroB [Salmonella enterica subsp. enterica serovar Infantis] Overall Protective Antigen Prediction = **0.4371** (Probable **ANTIGEN**).

>EHO9888389.1 salmochelin/enterobactin export ABC transporter IroC [Salmonella enterica subsp. enterica serovar Infantis] Overall Protective Antigen Prediction = **0.4852** (Probable **ANTIGEN**).

>EHO9888390.1 esterase family protein [Salmonella enterica subsp. enterica serovar Infantis] Overall Protective Antigen Prediction = **0.4194** (Probable **ANTIGEN**).

>EHO9888391.1 alpha/beta hydrolase [Salmonella enterica subsp. enterica serovar Infantis] Overall Protective Antigen Prediction = **0.5706** (Probable **ANTIGEN**).

>EHO9888392.1 TonB-dependent siderophore receptor [Salmonella enterica subsp. enterica serovar Infantis] Overall Protective Antigen Prediction = **0.8060** (Probable **ANTIGEN**).

>EHO9888393.1 hypothetical protein KND05_003834 [Salmonella enterica subsp. enterica serovar Infantis] Overall Protective Antigen Prediction = **0.5026** (Probable **ANTIGEN**).

>EHO9888394.1 SPI-2 type III secretion system effector PipB2 [Salmonella enterica subsp. enterica serovar Infantis] Overall Protective Antigen Prediction = **0.6645** (Probable **ANTIGEN**).

>EHO9888395.1 VirK family antimicrobial peptide resistance protein [Salmonella enterica subsp. enterica serovar Infantis] Overall Protective Antigen Prediction = **0.2144** (Probable **NON-ANTIGEN**).

>EHO9888396.1 antimicrobial resistance protein Mig-14 [Salmonella enterica subsp. enterica serovar Infantis] Overall Protective Antigen Prediction = **0.3442** (Probable **NON-ANTIGEN**).

>EHO9888397.1 HoxN/HupN/NixA family nickel/cobalt transporter [Salmonella enterica subsp. enterica serovar Infantis] Overall Protective Antigen Prediction = **0.5545** (Probable **ANTIGEN**).

>EHO9888398.1 sensor histidine kinase [Salmonella enterica subsp. enterica serovar Infantis] Overall Protective Antigen Prediction = **0.4247** (Probable **ANTIGEN**).

>EHO9888399.1 transcriptional regulator TctD [Salmonella enterica subsp. enterica serovar Infantis] Overall Protective Antigen Prediction = **0.3096** (Probable **NON-ANTIGEN**).

>EHO9888400.1 tripartite tricarboxylate transporter substrate binding protein [Salmonella enterica subsp. enterica serovar Infantis] Overall Protective Antigen Prediction = **0.3203** (Probable **NON-ANTIGEN**).

>EHO9888401.1 tripartite tricarboxylate transporter TctB family protein [Salmonella enterica subsp. enterica serovar Infantis] Overall Protective Antigen Prediction = **0.5263** (Probable **ANTIGEN**).

>EHO9888402.1 tripartite tricarboxylate transporter permease [Salmonella enterica subsp. enterica serovar Infantis] Overall Protective Antigen Prediction = **0.5390** (Probable **ANTIGEN**).

>EHO9888403.1 carbon starvation induced protein CsiD [Salmonella enterica subsp. enterica serovar Infantis] Overall Protective Antigen Prediction = **0.2720** (Probable **NON-ANTIGEN**).

>EHO9888404.1 L-2-hydroxyglutarate oxidase [Salmonella enterica subsp. enterica serovar Infantis] Overall Protective Antigen Prediction = **0.4546** (Probable **ANTIGEN**).

>EHO9888405.1 NADP-dependent succinate-semialdehyde dehydrogenase [Salmonella enterica subsp. enterica serovar Infantis] Overall Protective Antigen Prediction = **0.4248** (Probable **ANTIGEN**).

>EHO9888406.1 4-aminobutyrate--2-oxoglutarate transaminase [Salmonella enterica subsp. enterica serovar Infantis] Overall Protective Antigen Prediction = **0.3469** (Probable **NON-ANTIGEN**).

>EHO9888407.1 GABA permease [Salmonella enterica subsp. enterica serovar Infantis] Overall Protective Antigen Prediction = **0.5360** (Probable **ANTIGEN**).

>EHO9888408.1 DNA-binding transcriptional regulator CsiR [Salmonella enterica subsp. enterica serovar Infantis] Overall Protective Antigen Prediction = **0.3742** (Probable **NON-ANTIGEN**).

>EHO9888409.1 peptidoglycan-binding protein LysM [Salmonella enterica subsp. enterica serovar Infantis] Overall Protective Antigen Prediction = **0.4344** (Probable **ANTIGEN**).

>EHO9888410.1 YqaE/Pmp3 family membrane protein [Salmonella enterica subsp. enterica serovar Infantis] Overall Protective Antigen Prediction = **0.7969** (Probable **ANTIGEN**).

>EHO9888411.1 helix-turn-helix transcriptional regulator [Salmonella enterica subsp. enterica serovar Infantis] Overall Protective Antigen Prediction = **0.2678** (Probable **NON-ANTIGEN**).

>EHO9888412.1 rhodanese family protein [Salmonella enterica subsp. enterica serovar Infantis] Overall Protective Antigen Prediction = **0.2699** (Probable **NON-ANTIGEN**).

>EHO9888413.1 hypothetical protein KND05_003854 [Salmonella enterica subsp. enterica serovar Infantis] Overall Protective Antigen Prediction = **0.6381** (Probable **ANTIGEN**).

>EHO9888414.1 DNA-binding protein StpA [Salmonella enterica subsp. enterica serovar Infantis] Overall Protective Antigen Prediction = **0.4192** (Probable **ANTIGEN**).

>EHO9888415.1 L-alanine exporter AlaE [Salmonella enterica subsp. enterica serovar Infantis] Overall Protective Antigen Prediction = **0.3958** (Probable **NON-ANTIGEN**).

>EHO9888416.1 YgaC family protein [Salmonella enterica subsp. enterica serovar Infantis] Overall Protective Antigen Prediction = **0.4838** (Probable **ANTIGEN**).

>EHO9888417.1 DUF883 domain-containing protein [Salmonella enterica subsp. enterica serovar Infantis] Overall Protective Antigen Prediction = **0.2286** (Probable **NON-ANTIGEN**).

>EHO9888418.1 PLP-dependent aminotransferase family protein [Salmonella enterica subsp. enterica serovar Infantis] Overall Protective Antigen Prediction = **0.2828** (Probable **NON-ANTIGEN**).

>EHO9888419.1 carboxymuconolactone decarboxylase family protein [Salmonella enterica subsp. enterica serovar Infantis] Overall Protective Antigen Prediction = **0.6168** (Probable **ANTIGEN**).

>EHO9888420.1 glutaredoxin-like protein NrdH [Salmonella enterica subsp. enterica serovar Infantis] Overall Protective Antigen Prediction = **0.6184** (Probable **ANTIGEN**).

>EHO9888421.1 class Ib ribonucleoside-diphosphate reductase assembly flavoprotein NrdI [Salmonella enterica subsp. enterica serovar Infantis] Overall Protective Antigen Prediction = **0.3268** (Probable **NON-ANTIGEN**).

>EHO9888422.1 class 1b ribonucleoside-diphosphate reductase subunit alpha [Salmonella enterica

subsp. enterica serovar Infantis] Overall Protective Antigen Prediction = **0.4546** (Probable **ANTIGEN**).

>EHO9888423.1 class 1b ribonucleoside-diphosphate reductase subunit beta [Salmonella enterica subsp. enterica serovar Infantis] Overall Protective Antigen Prediction = **0.3540** (Probable **NON-ANTIGEN**).

>EHO9888424.1 glycine betaine/L-proline ABC transporter ATP-binding protein ProV [Salmonella enterica subsp. enterica serovar Infantis] Overall Protective Antigen Prediction = **0.3256** (Probable **NON-ANTIGEN**).

>EHO9888425.1 glycine betaine/L-proline ABC transporter permease ProW [Salmonella enterica subsp. enterica serovar Infantis] Overall Protective Antigen Prediction = **0.4099** (Probable **ANTIGEN**).

>EHO9888426.1 glycine betaine/L-proline ABC transporter substrate-binding protein ProX [Salmonella enterica subsp. enterica serovar Infantis] Overall Protective Antigen Prediction = **0.5120** (Probable **ANTIGEN**).

>EHO9888427.1 MFS transporter [Salmonella enterica subsp. enterica serovar Infantis] Overall Protective Antigen Prediction = **0.3136** (Probable **NON-ANTIGEN**).

>EHO9888428.1 transcriptional repressor MprA [Salmonella enterica subsp. enterica serovar Infantis] Overall Protective Antigen Prediction = **0.3470** (Probable **NON-ANTIGEN**).

>EHO9888429.1 multidrug efflux MFS transporter periplasmic adaptor subunit EmrA [Salmonella enterica subsp. enterica serovar Infantis] Overall Protective Antigen Prediction = **0.5859** (Probable **ANTIGEN**).

>EHO9888430.1 multidrug efflux MFS transporter permease subunit EmrB [Salmonella enterica subsp. enterica serovar Infantis] Overall Protective Antigen Prediction = **0.5507** (Probable **ANTIGEN**).

>EHO9888431.1 carbohydrate porin [Salmonella enterica subsp. enterica serovar Infantis] Overall Protective Antigen Prediction = **0.5560** (Probable **ANTIGEN**).

>EHO9888432.1 S-ribosylhomocysteine lyase [Salmonella enterica subsp. enterica serovar Infantis] Overall Protective Antigen Prediction = **0.4653** (Probable **ANTIGEN**).

>EHO9888433.1 glutamate--cysteine ligase [Salmonella enterica subsp. enterica serovar Infantis] Overall Protective Antigen Prediction = **0.5637** (Probable **ANTIGEN**).

>EHO9888434.1 DedA family protein [Salmonella enterica subsp. enterica serovar Infantis] Overall Protective Antigen Prediction = **0.2103** (Probable **NON-ANTIGEN**).

>EHO9888435.1 fructose-1-phosphate/6-phosphogluconate phosphatase [Salmonella enterica subsp. enterica serovar Infantis] Overall Protective Antigen Prediction = **0.4297** (Probable **ANTIGEN**).

>EHO9888436.1 ATP-dependent chaperone ClpB [Salmonella enterica subsp. enterica serovar Infantis] Overall Protective Antigen Prediction = **0.4823** (Probable **ANTIGEN**).

>EHO9888437.1 polyphenol oxidase [Salmonella enterica subsp. enterica serovar Infantis] Overall Protective Antigen Prediction = **0.4208** (Probable **ANTIGEN**).

>EHO9888438.1 23S rRNA pseudouridine(1911/1915/1917) synthase RluD [Salmonella enterica subsp. enterica serovar Infantis] Overall Protective Antigen Prediction = **0.4875** (Probable **ANTIGEN**).

>EHO9888439.1 outer membrane protein assembly factor BamD [Salmonella enterica subsp. enterica serovar Infantis] Overall Protective Antigen Prediction = **0.2740** (Probable **NON-ANTIGEN**).

>EHO9888440.1 ribosome-associated translation inhibitor RaiA [Salmonella enterica subsp. enterica serovar Infantis] Overall Protective Antigen Prediction = **0.3907** (Probable **NON-ANTIGEN**).

>EHO9888441.1 pheA operon leader peptide PheL [Salmonella enterica subsp. enterica serovar Infantis] Overall Protective Antigen Prediction = **2.9824** (Probable **ANTIGEN**).

>EHO9888442.1 bifunctional chorismate mutase/prephenate dehydratase [Salmonella enterica subsp. enterica serovar Infantis] Overall Protective Antigen Prediction = **0.2978** (Probable **NON-ANTIGEN**).

>EHO9888443.1 SMP-30/gluconolactonase/LRE family protein [Salmonella enterica subsp. enterica serovar Infantis] Overall Protective Antigen Prediction = **0.4638** (Probable **ANTIGEN**).

>EHO9888444.1 bifunctional chorismate mutase/prephenate dehydrogenase [Salmonella enterica subsp. enterica serovar Infantis] Overall Protective Antigen Prediction = **0.3132** (Probable **NON-ANTIGEN**).

>EHO9888445.1 3-deoxy-7-phosphoheptulonate synthase AroF [Salmonella enterica subsp. enterica serovar Infantis] Overall Protective Antigen Prediction = **0.5315** (Probable **ANTIGEN**).

>EHO9888446.1 YfiR family protein [Salmonella enterica subsp. enterica serovar Infantis] Overall Protective Antigen Prediction = **0.5408** (Probable **ANTIGEN**).

>EHO9888447.1 diguanylate cyclase DgcN [Salmonella enterica subsp. enterica serovar Infantis] Overall Protective Antigen Prediction = **0.5296** (Probable **ANTIGEN**).

>EHO9888448.1 50S ribosomal protein L19 [Salmonella enterica subsp. enterica serovar Infantis] Overall Protective Antigen Prediction = **0.6067** (Probable **ANTIGEN**).

>EHO9888449.1 tRNA (guanosine(37)-N1)-methyltransferase TrmD [Salmonella enterica subsp. enterica serovar Infantis] Overall Protective Antigen Prediction = **0.4800** (Probable **ANTIGEN**).

>EHO9888450.1 ribosome maturation factor RimM [Salmonella enterica subsp. enterica serovar Infantis] Overall Protective Antigen Prediction = **0.4420** (Probable **ANTIGEN**).

>EHO9888451.1 30S ribosomal protein S16 [Salmonella enterica subsp. enterica serovar Infantis] Overall Protective Antigen Prediction = **0.3631** (Probable **NON-ANTIGEN**).

>EHO9888452.1 signal recognition particle protein [Salmonella enterica subsp. enterica serovar Infantis] Overall Protective Antigen Prediction = **0.3734** (Probable **NON-ANTIGEN**).

>EHO9888453.1 inner membrane protein YpjD [Salmonella enterica subsp. enterica serovar Infantis] Overall Protective Antigen Prediction = **0.3731** (Probable **NON-ANTIGEN**).

>EHO9888454.1 HlyC/CorC family transporter [Salmonella enterica subsp. enterica serovar Infantis] Overall Protective Antigen Prediction = **0.3594** (Probable **NON-ANTIGEN**).

>EHO9888455.1 cytoplasmic protein [Salmonella enterica subsp. enterica serovar Infantis] Overall Protective Antigen Prediction = **0.3066** (Probable **NON-ANTIGEN**).

>EHO9888456.1 nucleotide exchange factor GrpE [Salmonella enterica subsp. enterica serovar Infantis] Overall Protective Antigen Prediction = **0.5366** (Probable **ANTIGEN**).

>EHO9888457.1 NAD(+) kinase [Salmonella enterica subsp. enterica serovar Infantis] Overall Protective Antigen Prediction = **0.5137** (Probable **ANTIGEN**).

>EHO9888458.1 DNA repair protein RecN [Salmonella enterica subsp. enterica serovar Infantis] Overall Protective Antigen Prediction = **0.4367** (Probable **ANTIGEN**).

>EHO9888459.1 outer membrane protein assembly factor BamE [Salmonella enterica subsp. enterica serovar Infantis] Overall Protective Antigen Prediction = **0.5900** (Probable **ANTIGEN**).

>EHO9888460.1 RnfH family protein [Salmonella enterica subsp. enterica serovar Infantis] Overall Protective Antigen Prediction = **0.5192** (Probable **ANTIGEN**).

>EHO9888461.1 type II toxin-antitoxin system RatA family toxin [Salmonella enterica subsp. enterica serovar Infantis] Overall Protective Antigen Prediction = **0.4342** (Probable **ANTIGEN**).

>EHO9888462.1 SsrA-binding protein SmpB [Salmonella enterica subsp. enterica serovar Infantis] Overall Protective Antigen Prediction = **0.6244** (Probable **ANTIGEN**).

>EHO9888463.1 biofilm-associated protein BapA [Salmonella enterica subsp. enterica serovar Infantis] Overall Protective Antigen Prediction = **0.7030** (Probable **ANTIGEN**).

>EHO9888464.1 TolC family outer membrane protein [Salmonella enterica subsp. enterica serovar Infantis] Overall Protective Antigen Prediction = **0.5282** (Probable **ANTIGEN**).

>EHO9888465.1 type I secretion system permease/ATPase [Salmonella enterica subsp. enterica serovar Infantis] Overall Protective Antigen Prediction = **0.4490** (Probable **ANTIGEN**).

>EHO9888466.1 HlyD family type I secretion periplasmic adaptor subunit [Salmonella enterica subsp. enterica serovar Infantis] Overall Protective Antigen Prediction = **0.5834** (Probable **ANTIGEN**).

>EHO9888467.1 tyrosine-type recombinase/integrase [Salmonella enterica subsp. enterica serovar Infantis] Overall Protective Antigen Prediction = **0.3617** (Probable **NON-ANTIGEN**).

>EHO9888468.1 hypothetical protein KND05_003910 [Salmonella enterica subsp. enterica serovar Infantis] Overall Protective Antigen Prediction = **0.4287** (Probable **ANTIGEN**).

>EHO9888469.1 phage polarity suppression protein [Salmonella enterica subsp. enterica serovar Infantis] Overall Protective Antigen Prediction = **0.3282** (Probable **NON-ANTIGEN**).

>EHO9888470.1 ogr/Delta-like zinc finger family protein [Salmonella enterica subsp. enterica serovar Infantis] Overall Protective Antigen Prediction = **0.4174** (Probable **ANTIGEN**).

>EHO9888471.1 glycoprotein 3 [Salmonella enterica subsp. enterica serovar Infantis] Overall Protective Antigen Prediction = **0.5733** (Probable **ANTIGEN**).

>EHO9888472.1 AlpA family transcriptional regulator [Salmonella enterica subsp. enterica serovar Infantis] Overall Protective Antigen Prediction = **0.1501** (Probable **NON-ANTIGEN**).

>EHO9888473.1 host cell division inhibitor Icd-like protein [Salmonella enterica subsp. enterica serovar Infantis] Overall Protective Antigen Prediction = **0.6074** (Probable **ANTIGEN**).

>EHO9888474.1 hypothetical protein KND05_003916 [Salmonella enterica subsp. enterica serovar Infantis] Overall Protective Antigen Prediction = **0.3185** (Probable **NON-ANTIGEN**).

>EHO9888475.1 DUF5375 domain-containing protein [Salmonella enterica subsp. enterica serovar Infantis] Overall Protective Antigen Prediction = **0.4999** (Probable **ANTIGEN**).

>EHO9888476.1 toprim domain-containing protein [Salmonella enterica subsp. enterica serovar Infantis] Overall Protective Antigen Prediction = **0.5940** (Probable **ANTIGEN**).

>EHO9888477.1 phase 1 flagellin gene repressor FljA [Salmonella enterica subsp. enterica serovar Infantis] Overall Protective Antigen Prediction = **0.6668** (Probable **ANTIGEN**).

>EHO9888478.1 flagellin FliC, partial [Salmonella enterica subsp. enterica serovar Infantis] Overall Protective Antigen Prediction = **0.8524** (Probable **ANTIGEN**).

>EHO9888479.1 maltose/maltodextrin ABC transporter substrate-binding protein MalE [Salmonella enterica subsp. enterica serovar Infantis] Overall Protective Antigen Prediction = **0.4373** (Probable **ANTIGEN**).

>EHO9888480.1 maltose ABC transporter permease MalF [Salmonella enterica subsp. enterica serovar Infantis] Overall Protective Antigen Prediction = **0.5387** (Probable **ANTIGEN**).

>EHO9888481.1 maltose ABC transporter permease MalG [Salmonella enterica subsp. enterica serovar Infantis] Overall Protective Antigen Prediction = **0.6369** (Probable **ANTIGEN**).

>EHO9888482.1 phosphate-starvation-inducible protein PsiE [Salmonella enterica subsp. enterica serovar Infantis] Overall Protective Antigen Prediction = **0.3421** (Probable **NON-ANTIGEN**).

>EHO9888483.1 YjbH domain-containing protein [Salmonella enterica subsp. enterica serovar Infantis] Overall Protective Antigen Prediction = **0.5504** (Probable **ANTIGEN**).

>EHO9888484.1 capsule biosynthesis GfcC family protein [Salmonella enterica subsp. enterica serovar Infantis] Overall Protective Antigen Prediction = **0.3324** (Probable **NON-ANTIGEN**).

>EHO9888485.1 YjbF family lipoprotein [Salmonella enterica subsp. enterica serovar Infantis] Overall Protective Antigen Prediction = **0.4709** (Probable **ANTIGEN**).

>EHO9888486.1 hypothetical protein KND05_003929 [Salmonella enterica subsp. enterica serovar Infantis] Overall Protective Antigen Prediction = **1.2746** (Probable **ANTIGEN**).

>EHO9888487.1 glucose-6-phosphate isomerase [Salmonella enterica subsp. enterica serovar Infantis] Overall Protective Antigen Prediction = **0.2939** (Probable **NON-ANTIGEN**).

>EHO9888488.1 lysine-sensitive aspartokinase 3 [Salmonella enterica subsp. enterica serovar Infantis] Overall Protective Antigen Prediction = **0.4128** (Probable **ANTIGEN**).

>EHO9888489.1 DNA-binding protein [Salmonella enterica subsp. enterica serovar Infantis] Overall Protective Antigen Prediction = **0.5243** (Probable **ANTIGEN**).

>EHO9888490.1 putative holin [Salmonella enterica subsp. enterica serovar Infantis] Overall

Protective Antigen Prediction = **0.1294** (Probable **NON-ANTIGEN**).

>EHO9888491.1 lytic transglycosylase domain-containing protein [Salmonella enterica subsp. enterica serovar Infantis] Overall Protective Antigen Prediction = **0.3484** (Probable **NON-ANTIGEN**).

>EHO9888492.1 hypothetical protein KND05_003935 [Salmonella enterica subsp. enterica serovar Infantis] Overall Protective Antigen Prediction = **0.4214** (Probable **ANTIGEN**).

>EHO9888493.1 Gp37 family protein [Salmonella enterica subsp. enterica serovar Infantis] Overall Protective Antigen Prediction = **0.2353** (Probable **NON-ANTIGEN**).

>EHO9888494.1 hypothetical protein KND05_003937 [Salmonella enterica subsp. enterica serovar Infantis] Overall Protective Antigen Prediction = **0.2842** (Probable **NON-ANTIGEN**).

>EHO9888495.1 phage tail sheath family protein [Salmonella enterica subsp. enterica serovar Infantis] Overall Protective Antigen Prediction = **0.4899** (Probable **ANTIGEN**).

>EHO9888496.1 phage major tail tube protein [Salmonella enterica subsp. enterica serovar Infantis] Overall Protective Antigen Prediction = **0.3412** (Probable **NON-ANTIGEN**).

>EHO9888497.1 phage tail assembly protein [Salmonella enterica subsp. enterica serovar Infantis] Overall Protective Antigen Prediction = **0.7400** (Probable **ANTIGEN**).

>EHO9888498.1 GpE family phage tail protein [Salmonella enterica subsp. enterica serovar Infantis] Overall Protective Antigen Prediction = **0.6884** (Probable **ANTIGEN**).

>EHO9888499.1 phage tail tape measure protein [Salmonella enterica subsp. enterica serovar Infantis] Overall Protective Antigen Prediction = **0.4347** (Probable **ANTIGEN**).

>EHO9888500.1 phage tail protein [Salmonella enterica subsp. enterica serovar Infantis] Overall Protective Antigen Prediction = **0.2758** (Probable **NON-ANTIGEN**).

>EHO9888501.1 tail protein X [Salmonella enterica subsp. enterica serovar Infantis] Overall Protective Antigen Prediction = **0.4846** (Probable **ANTIGEN**).

>EHO9888502.1 phage late control D family protein [Salmonella enterica subsp. enterica serovar Infantis] Overall Protective Antigen Prediction = **0.5357** (Probable **ANTIGEN**).

>EHO9888503.1 phage baseplate assembly protein V [Salmonella enterica subsp. enterica serovar Infantis] Overall Protective Antigen Prediction = **0.5907** (Probable **ANTIGEN**).

>EHO9888504.1 GtrA family protein [Salmonella enterica subsp. enterica serovar Infantis] Overall Protective Antigen Prediction = **0.4918** (Probable **ANTIGEN**).

>EHO9888505.1 glycosyltransferase family 2 protein [Salmonella enterica subsp. enterica serovar Infantis] Overall Protective Antigen Prediction = **0.3782** (Probable **NON-ANTIGEN**).

>EHO9888506.1 hypothetical protein KND05_003949 [Salmonella enterica subsp. enterica serovar Infantis] Overall Protective Antigen Prediction = **0.5731** (Probable **ANTIGEN**).

>EHO9888507.1 GPW/gp25 family protein [Salmonella enterica subsp. enterica serovar Infantis] Overall Protective Antigen Prediction = **0.4943** (Probable **ANTIGEN**).

>EHO9888508.1 baseplate J/gp47 family protein [Salmonella enterica subsp. enterica serovar Infantis] Overall Protective Antigen Prediction = **0.4969** (Probable **ANTIGEN**).

>EHO9888509.1 phage tail protein I [Salmonella enterica subsp. enterica serovar Infantis] Overall Protective Antigen Prediction = **0.3663** (Probable **NON-ANTIGEN**).

>EHO9888510.1 tail fiber protein [Salmonella enterica subsp. enterica serovar Infantis] Overall Protective Antigen Prediction = **0.7714** (Probable **ANTIGEN**).

>EHO9888511.1 DUF4376 domain-containing protein [Salmonella enterica subsp. enterica serovar Infantis] Overall Protective Antigen Prediction = **0.3785** (Probable **NON-ANTIGEN**).

>EHO9888512.1 hypothetical protein KND05_003955 [Salmonella enterica subsp. enterica serovar Infantis] Overall Protective Antigen Prediction = **0.2733** (Probable **NON-ANTIGEN**).

>EHO9888513.1 hypothetical protein KND05_003957 [Salmonella enterica subsp. enterica serovar Infantis] Overall Protective Antigen Prediction = **0.4103** (Probable **ANTIGEN**).

>EHO9888514.1 ketopantoate/pantoate/pantothenate transporter PanS [Salmonella enterica subsp. enterica serovar Infantis] Overall Protective Antigen Prediction = **0.5737** (Probable **ANTIGEN**).

>EHO9888515.1 DUF3811 domain-containing protein [Salmonella enterica subsp. enterica serovar Infantis] Overall Protective Antigen Prediction = **0.6790** (Probable **ANTIGEN**).

>EHO9888516.1 23S rRNA pseudouridine(2604) synthase RluF [Salmonella enterica subsp. enterica serovar Infantis] Overall Protective Antigen Prediction = **0.2828** (Probable **NON-ANTIGEN**).

>EHO9888517.1 DUF2058 domain-containing protein [Salmonella enterica subsp. enterica serovar Infantis] Overall Protective Antigen Prediction = **0.5482** (Probable **ANTIGEN**).

>EHO9888518.1 hypothetical protein KND05_003962 [Salmonella enterica subsp. enterica serovar Infantis] Overall Protective Antigen Prediction = **0.5003** (Probable **ANTIGEN**).

>EHO9888519.1 dipeptidase PepE [Salmonella enterica subsp. enterica serovar Infantis] Overall Protective Antigen Prediction = **0.3960** (Probable **NON-ANTIGEN**).

>EHO9888520.1 Na/Pi cotransporter family protein [Salmonella enterica subsp. enterica serovar Infantis] Overall Protective Antigen Prediction = **0.4270** (Probable **ANTIGEN**).

>EHO9888521.1 methionine synthase [Salmonella enterica subsp. enterica serovar Infantis] Overall Protective Antigen Prediction = **0.4341** (Probable **ANTIGEN**).

>EHO9888522.1 glyoxylate bypass operon transcriptional repressor IclR [Salmonella enterica subsp. enterica serovar Infantis] Overall Protective Antigen Prediction = **0.3433** (Probable **NON-ANTIGEN**).

>EHO9888523.1 ASCH domain-containing protein [Salmonella enterica subsp. enterica serovar Infantis] Overall Protective Antigen Prediction = **0.5732** (Probable **ANTIGEN**).

>EHO9888524.1 bifunctional isocitrate dehydrogenase kinase/phosphatase [Salmonella enterica subsp. enterica serovar Infantis] Overall Protective Antigen Prediction = **0.4090** (Probable **ANTIGEN**).

>EHO9888525.1 isocitrate lyase [Salmonella enterica subsp. enterica serovar Infantis] Overall

Protective Antigen Prediction = **0.4089** (Probable **ANTIGEN**).

>EHO9888526.1 malate synthase A [Salmonella enterica subsp. enterica serovar Infantis] Overall Protective Antigen Prediction = **0.3551** (Probable **NON-ANTIGEN**).

>EHO9888527.1 homoserine O-succinyltransferase [Salmonella enterica subsp. enterica serovar Infantis] Overall Protective Antigen Prediction = **0.4284** (Probable **ANTIGEN**).

>EHO9888528.1 acetyltransferase [Salmonella enterica subsp. enterica serovar Infantis] Overall Protective Antigen Prediction = **0.5793** (Probable **ANTIGEN**).

>EHO9888529.1 HTH-type transcriptional regulator GalS [Salmonella enterica subsp. enterica serovar Infantis] Overall Protective Antigen Prediction = **0.3595** (Probable **NON-ANTIGEN**).

>EHO9888530.1 DUF418 family protein [Salmonella enterica subsp. enterica serovar Infantis] Overall Protective Antigen Prediction = **0.4708** (Probable **ANTIGEN**).

>EHO9888531.1 GTP cyclohydrolase I FolE [Salmonella enterica subsp. enterica serovar Infantis] Overall Protective Antigen Prediction = **0.2657** (Probable **NON-ANTIGEN**).

>EHO9888532.1 S-formylglutathione hydrolase [Salmonella enterica subsp. enterica serovar Infantis] Overall Protective Antigen Prediction = **0.3882** (Probable **NON-ANTIGEN**).

>EHO9888533.1 helix-turn-helix transcriptional regulator [Salmonella enterica subsp. enterica serovar Infantis] Overall Protective Antigen Prediction = **0.5866** (Probable **ANTIGEN**).

>EHO9888534.1 L-serine ammonia-lyase [Salmonella enterica subsp. enterica serovar Infantis] Overall Protective Antigen Prediction = **0.4705** (Probable **ANTIGEN**).

>EHO9888535.1 phosphoserine phosphatase SerB [Salmonella enterica subsp. enterica serovar Infantis] Overall Protective Antigen Prediction = **0.5940** (Probable **ANTIGEN**).

>EHO9888536.1 MFS transporter [Salmonella enterica subsp. enterica serovar Infantis] Overall Protective Antigen Prediction = **0.3425** (Probable **NON-ANTIGEN**).

>EHO9888537.1 catecholate siderophore receptor CirA [Salmonella enterica subsp. enterica serovar Infantis] Overall Protective Antigen Prediction = **0.7303** (Probable **ANTIGEN**).

>EHO9888538.1 lysine-specific permease [Salmonella enterica subsp. enterica serovar Infantis] Overall Protective Antigen Prediction = **0.4926** (Probable **ANTIGEN**).

>EHO9888539.1 LysR family transcriptional regulator [Salmonella enterica subsp. enterica serovar Infantis] Overall Protective Antigen Prediction = **0.3601** (Probable **NON-ANTIGEN**).

>EHO9888540.1 YeiH family protein [Salmonella enterica subsp. enterica serovar Infantis] Overall Protective Antigen Prediction = **0.5770** (Probable **ANTIGEN**).

>EHO9888541.1 deoxyribonuclease IV [Salmonella enterica subsp. enterica serovar Infantis] Overall Protective Antigen Prediction = **0.4254** (Probable **ANTIGEN**).

>EHO9888542.1 PTS fructose transporter subunit IIBC [Salmonella enterica subsp. enterica serovar Infantis] Overall Protective Antigen Prediction = **0.4774** (Probable **ANTIGEN**).

>EHO9888543.1 1-phosphofructokinase [Salmonella enterica subsp. enterica serovar Infantis] Overall

Protective Antigen Prediction = **0.4196** (Probable **ANTIGEN**).

>EHO9888544.1 fused PTS fructose transporter subunit IIA/HPr protein [Salmonella enterica subsp. enterica serovar Infantis] Overall Protective Antigen Prediction = **0.5093** (Probable **ANTIGEN**).

>EHO9888545.1 sugar efflux transporter SetB [Salmonella enterica subsp. enterica serovar Infantis] Overall Protective Antigen Prediction = **0.5354** (Probable **ANTIGEN**).

>EHO9888546.1 hypothetical protein KND05_003990 [Salmonella enterica subsp. enterica serovar Infantis] Overall Protective Antigen Prediction = **0.5271** (Probable **ANTIGEN**).

>EHO9888547.1 hypothetical protein KND05_003991 [Salmonella enterica subsp. enterica serovar Infantis] Overall Protective Antigen Prediction = **0.7050** (Probable **ANTIGEN**).

>EHO9888548.1 zinc/iron-chelating domain-containing protein [Salmonella enterica subsp. enterica serovar Infantis] Overall Protective Antigen Prediction = **0.6586** (Probable **ANTIGEN**).

>EHO9888549.1 elongation factor P-like protein YeiP [Salmonella enterica subsp. enterica serovar Infantis] Overall Protective Antigen Prediction = **0.5639** (Probable **ANTIGEN**).

>EHO9888550.1 GTP-binding protein [Salmonella enterica subsp. enterica serovar Infantis] Overall Protective Antigen Prediction = **0.4221** (Probable **ANTIGEN**).

>EHO9888551.1 phosphatase PAP2 family protein [Salmonella enterica subsp. enterica serovar Infantis] Overall Protective Antigen Prediction = **0.4503** (Probable **ANTIGEN**).

>EHO9888552.1 bifunctional murein DD-endopeptidase/murein LD-carboxypeptidase [Salmonella enterica subsp. enterica serovar Infantis] Overall Protective Antigen Prediction = **0.6394** (Probable **ANTIGEN**).

>EHO9888553.1 cyclic di-GMP phosphodiesterase [Salmonella enterica subsp. enterica serovar Infantis] Overall Protective Antigen Prediction = **0.4070** (Probable **ANTIGEN**).

>EHO9888554.1 ABC transporter substrate-binding protein [Salmonella enterica subsp. enterica serovar Infantis] Overall Protective Antigen Prediction = **0.4530** (Probable **ANTIGEN**).

>EHO9888555.1 microcin C ABC transporter permease YejB [Salmonella enterica subsp. enterica serovar Infantis] Overall Protective Antigen Prediction = **0.5589** (Probable **ANTIGEN**).

>EHO9888556.1 ABC transporter permease [Salmonella enterica subsp. enterica serovar Infantis] Overall Protective Antigen Prediction = **0.4534** (Probable **ANTIGEN**).

>EHO9888557.1 microcin C ABC transporter ATP-binding protein YejF [Salmonella enterica subsp. enterica serovar Infantis] Overall Protective Antigen Prediction = **0.4662** (Probable **ANTIGEN**).

>EHO9888558.1 hypothetical protein KND05_004002 [Salmonella enterica subsp. enterica serovar Infantis] Overall Protective Antigen Prediction = **0.3126** (Probable **NON-ANTIGEN**).

>EHO9888559.1 Bcr/CflA family multidrug efflux MFS transporter [Salmonella enterica subsp. enterica serovar Infantis] Overall Protective Antigen Prediction = **0.5742** (Probable **ANTIGEN**).

>EHO9888560.1 16S rRNA pseudouridine(516) synthase RsuA [Salmonella enterica subsp. enterica serovar Infantis] Overall Protective Antigen Prediction = **0.3619** (Probable **NON-ANTIGEN**).

>EHO9888561.1 DEAD/DEAH box helicase [Salmonella enterica subsp. enterica serovar Infantis] Overall Protective Antigen Prediction = **0.5045** (Probable **ANTIGEN**).

>EHO9888562.1 50S ribosomal protein L25 [Salmonella enterica subsp. enterica serovar Infantis] Overall Protective Antigen Prediction = **0.9269** (Probable **ANTIGEN**).

>EHO9888563.1 hypothetical protein KND05_004007 [Salmonella enterica subsp. enterica serovar Infantis] Overall Protective Antigen Prediction = **0.7988** (Probable **ANTIGEN**).

>EHO9888564.1 nucleoid-associated protein YejK [Salmonella enterica subsp. enterica serovar Infantis] Overall Protective Antigen Prediction = **0.5046** (Probable **ANTIGEN**).

>EHO9888565.1 YejL family protein [Salmonella enterica subsp. enterica serovar Infantis] Overall Protective Antigen Prediction = **0.0175** (Probable **NON-ANTIGEN**).

>EHO9888566.1 cardiolipin transport protein PbgA [Salmonella enterica subsp. enterica serovar Infantis] Overall Protective Antigen Prediction = **0.4154** (Probable **ANTIGEN**).

>EHO9888567.1 SPI-2 type III secretion system effector E3 ubiquitin transferase SspH2 [Salmonella enterica subsp. enterica serovar Infantis] Overall Protective Antigen Prediction = **0.3809** (Probable **NON-ANTIGEN**).

>EHO9888568.1 phage tail protein [Salmonella enterica subsp. enterica serovar Infantis] Overall Protective Antigen Prediction = **0.7189** (Probable **ANTIGEN**).

>EHO9888569.1 tail fiber protein [Salmonella enterica subsp. enterica serovar Infantis] Overall Protective Antigen Prediction = **0.3587** (Probable **NON-ANTIGEN**).

>EHO9888570.1 hypothetical protein KND05_004016 [Salmonella enterica subsp. enterica serovar Infantis] Overall Protective Antigen Prediction = **0.8169** (Probable **ANTIGEN**).

>EHO9888571.1 virulence protein MsgA [Salmonella enterica subsp. enterica serovar Infantis] Overall Protective Antigen Prediction = **0.5100** (Probable **ANTIGEN**).

>EHO9888572.1 acyloxyacyl hydrolase [Salmonella enterica subsp. enterica serovar Infantis] Overall Protective Antigen Prediction = **0.5890** (Probable **ANTIGEN**).

>EHO9888573.1 nitrate/nitrite response regulator protein NarP [Salmonella enterica subsp. enterica serovar Infantis] Overall Protective Antigen Prediction = **0.4908** (Probable **ANTIGEN**).

>EHO9888574.1 Obg family GTPase CgtA [Salmonella enterica subsp. enterica serovar Infantis] Overall Protective Antigen Prediction = **0.6027** (Probable **ANTIGEN**).

>EHO9888575.1 DMT family transporter [Salmonella enterica subsp. enterica serovar Infantis] Overall Protective Antigen Prediction = **0.2322** (Probable **NON-ANTIGEN**).

>EHO9888576.1 50S ribosomal protein L27 [Salmonella enterica subsp. enterica serovar Infantis] Overall Protective Antigen Prediction = **0.9417** (Probable **ANTIGEN**).

>EHO9888577.1 50S ribosomal protein L21 [Salmonella enterica subsp. enterica serovar Infantis] Overall Protective Antigen Prediction = **0.7498** (Probable **ANTIGEN**).

>EHO9888578.1 octaprenyl diphosphate synthase [Salmonella enterica subsp. enterica serovar Infantis] Overall Protective Antigen Prediction = **0.3929** (Probable **NON-ANTIGEN**).

>EHO9888579.1 DNA-binding transcriptional regulator SfsB [Salmonella enterica subsp. enterica serovar Infantis] Overall Protective Antigen Prediction = **0.3742** (Probable **NON-ANTIGEN**).

>EHO9888580.1 UDP-N-acetylglucosamine 1-carboxyvinyltransferase [Salmonella enterica subsp. enterica serovar Infantis] Overall Protective Antigen Prediction = **0.5570** (Probable **ANTIGEN**).

>EHO9888581.1 BolA family iron metabolism protein IbaG [Salmonella enterica subsp. enterica serovar Infantis] Overall Protective Antigen Prediction = **0.4324** (Probable **ANTIGEN**).

>EHO9888582.1 lipid asymmetry maintenance protein MlaB [Salmonella enterica subsp. enterica serovar Infantis] Overall Protective Antigen Prediction = **0.5180** (Probable **ANTIGEN**).

>EHO9888583.1 phospholipid-binding protein MlaC [Salmonella enterica subsp. enterica serovar Infantis] Overall Protective Antigen Prediction = **0.4394** (Probable **ANTIGEN**).

>EHO9888584.1 outer membrane lipid asymmetry maintenance protein MlaD [Salmonella enterica subsp. enterica serovar Infantis] Overall Protective Antigen Prediction = **0.7173** (Probable **ANTIGEN**).

>EHO9888585.1 lipid asymmetry maintenance ABC transporter permease subunit MlaE [Salmonella enterica subsp. enterica serovar Infantis] Overall Protective Antigen Prediction = **0.2755** (Probable **NON-ANTIGEN**).

>EHO9888586.1 phospholipid ABC transporter ATP-binding protein MlaF [Salmonella enterica subsp. enterica serovar Infantis] Overall Protective Antigen Prediction = **0.1906** (Probable **NON-ANTIGEN**).

>EHO9888587.1 calcium/sodium antiporter [Salmonella enterica subsp. enterica serovar Infantis] Overall Protective Antigen Prediction = **0.5181** (Probable **ANTIGEN**).

>EHO9888588.1 arabinose-5-phosphate isomerase KdsD [Salmonella enterica subsp. enterica serovar Infantis] Overall Protective Antigen Prediction = **0.4131** (Probable **ANTIGEN**).

>EHO9888589.1 3-deoxy-manno-octulosonate-8-phosphatase KdsC [Salmonella enterica subsp. enterica serovar Infantis] Overall Protective Antigen Prediction = **0.5319** (Probable **ANTIGEN**).

>EHO9888590.1 LPS export ABC transporter periplasmic protein LptC [Salmonella enterica subsp. enterica serovar Infantis] Overall Protective Antigen Prediction = **0.5506** (Probable **ANTIGEN**).

>EHO9888591.1 lipopolysaccharide ABC transporter substrate-binding protein LptA [Salmonella enterica subsp. enterica serovar Infantis] Overall Protective Antigen Prediction = **0.6148** (Probable **ANTIGEN**).

>EHO9888592.1 LPS export ABC transporter ATP-binding protein [Salmonella enterica subsp. enterica serovar Infantis] Overall Protective Antigen Prediction = **0.3342** (Probable **NON-ANTIGEN**).

>EHO9888593.1 RNA polymerase factor sigma-54 [Salmonella enterica subsp. enterica serovar Infantis] Overall Protective Antigen Prediction = **0.3203** (Probable **NON-ANTIGEN**).

>EHO9888594.1 ribosome hibernation promoting factor [Salmonella enterica subsp. enterica serovar Infantis] Overall Protective Antigen Prediction = **0.5029** (Probable **ANTIGEN**).

>EHO9888595.1 PTS IIA-like nitrogen regulatory protein PtsN [Salmonella enterica subsp. enterica serovar Infantis] Overall Protective Antigen Prediction = **0.6011** (Probable **ANTIGEN**).

>EHO9888596.1 RNase adapter RapZ [Salmonella enterica subsp. enterica serovar Infantis] Overall Protective Antigen Prediction = **0.5114** (Probable **ANTIGEN**).

>EHO9888597.1 PTS phosphocarrier protein NPr [Salmonella enterica subsp. enterica serovar Infantis] Overall Protective Antigen Prediction = **0.7806** (Probable **ANTIGEN**).

>EHO9888598.1 PhoP regulatory network protein YrbL [Salmonella enterica subsp. enterica serovar Infantis] Overall Protective Antigen Prediction = **0.3626** (Probable **NON-ANTIGEN**).

>EHO9888599.1 monofunctional biosynthetic peptidoglycan transglycosylase [Salmonella enterica subsp. enterica serovar Infantis] Overall Protective Antigen Prediction = **0.2545** (Probable **NON-ANTIGEN**).

>EHO9888600.1 isoprenoid biosynthesis glyoxalase ElbB [Salmonella enterica subsp. enterica serovar Infantis] Overall Protective Antigen Prediction = **0.3793** (Probable **NON-ANTIGEN**).

>EHO9888601.1 aerobic respiration two-component sensor histidine kinase ArcB [Salmonella enterica subsp. enterica serovar Infantis] Overall Protective Antigen Prediction = **0.5199** (Probable **ANTIGEN**).

>EHO9888602.1 TIGR01212 family radical SAM protein [Salmonella enterica subsp. enterica serovar Infantis] Overall Protective Antigen Prediction = **0.3879** (Probable **NON-ANTIGEN**).

>EHO9888603.1 glutamate synthase large subunit [Salmonella enterica subsp. enterica serovar Infantis] Overall Protective Antigen Prediction = **0.4766** (Probable **ANTIGEN**).

>EHO9888604.1 glutamate synthase subunit GltD [Salmonella enterica subsp. enterica serovar Infantis] Overall Protective Antigen Prediction = **0.5656** (Probable **ANTIGEN**).

>EHO9888605.1 DUF1016 family protein [Salmonella enterica subsp. enterica serovar Infantis] Overall Protective Antigen Prediction = **0.4592** (Probable **ANTIGEN**).

>EHO9888606.1 cytosine permease [Salmonella enterica subsp. enterica serovar Infantis] Overall Protective Antigen Prediction = **0.3426** (Probable **NON-ANTIGEN**).

>EHO9888607.1 cytosine deaminase [Salmonella enterica subsp. enterica serovar Infantis] Overall Protective Antigen Prediction = **0.4260** (Probable **ANTIGEN**).

>EHO9888608.1 YhcH/YjgK/YiaL family protein [Salmonella enterica subsp. enterica serovar Infantis] Overall Protective Antigen Prediction = **0.6285** (Probable **ANTIGEN**).

>EHO9888609.1 N-acetylmannosamine kinase [Salmonella enterica subsp. enterica serovar Infantis] Overall Protective Antigen Prediction = **0.4231** (Probable **ANTIGEN**).

>EHO9888610.1 N-acetylmannosamine-6-phosphate 2-epimerase [Salmonella enterica subsp. enterica serovar Infantis] Overall Protective Antigen Prediction = **0.3935** (Probable **NON-ANTIGEN**).

>EHO9888611.1 sialic acid transporter NanT [Salmonella enterica subsp. enterica serovar Infantis] Overall Protective Antigen Prediction = **0.5013** (Probable **ANTIGEN**).

>EHO9888612.1 N-acetylneuraminate lyase [Salmonella enterica subsp. enterica serovar Infantis]

Overall Protective Antigen Prediction = **0.3811** (Probable **NON-ANTIGEN**).

>EHO9888613.1 transcriptional regulator NanR [Salmonella enterica subsp. enterica serovar Infantis] Overall Protective Antigen Prediction = **0.3474** (Probable **NON-ANTIGEN**).

>EHO9888614.1 ClpXP protease specificity-enhancing factor [Salmonella enterica subsp. enterica serovar Infantis] Overall Protective Antigen Prediction = **0.6713** (Probable **ANTIGEN**).

>EHO9888615.1 stringent starvation protein A [Salmonella enterica subsp. enterica serovar Infantis] Overall Protective Antigen Prediction = **0.5576** (Probable **ANTIGEN**).

>EHO9888616.1 DUF695 domain-containing protein [Salmonella enterica subsp. enterica serovar Infantis] Overall Protective Antigen Prediction = **0.4148** (Probable **ANTIGEN**).

>EHO9888617.1 30S ribosomal protein S9 [Salmonella enterica subsp. enterica serovar Infantis] Overall Protective Antigen Prediction = **0.5754** (Probable **ANTIGEN**).

>EHO9888618.1 50S ribosomal protein L13 [Salmonella enterica subsp. enterica serovar Infantis] Overall Protective Antigen Prediction = **0.4890** (Probable **ANTIGEN**).

>EHO9888619.1 AFG1 family ATPase [Salmonella enterica subsp. enterica serovar Infantis] Overall Protective Antigen Prediction = **0.3793** (Probable **NON-ANTIGEN**).

>EHO9888620.1 DUF1043 family protein [Salmonella enterica subsp. enterica serovar Infantis] Overall Protective Antigen Prediction = **0.4549** (Probable **ANTIGEN**).

>EHO9888621.1 serine endoprotease DegQ [Salmonella enterica subsp. enterica serovar Infantis] Overall Protective Antigen Prediction = **0.5146** (Probable **ANTIGEN**).

>EHO9888622.1 outer membrane-stress sensor serine endopeptidase DegS [Salmonella enterica subsp. enterica serovar Infantis] Overall Protective Antigen Prediction = **0.4335** (Probable **ANTIGEN**).

>EHO9888623.1 triphosphoribosyl-dephospho-CoA synthase [Salmonella enterica subsp. enterica serovar Infantis] Overall Protective Antigen Prediction = **0.2571** (Probable **NON-ANTIGEN**).

>EHO9888624.1 sodium ion-translocating decarboxylase subunit beta, partial [Salmonella enterica subsp. enterica serovar Infantis] Overall Protective Antigen Prediction = **0.4704** (Probable **ANTIGEN**).

>EHO9888625.1 hypothetical protein KND05_004071, partial [Salmonella enterica subsp. enterica serovar Infantis] Overall Protective Antigen Prediction = **0.4978** (Probable **ANTIGEN**).

>EHO9888626.1 hypothetical protein KND05_004072 [Salmonella enterica subsp. enterica serovar Infantis] Overall Protective Antigen Prediction = **0.8685** (Probable **ANTIGEN**).

>EHO9888627.1 integrase [Salmonella enterica subsp. enterica serovar Infantis] Overall Protective Antigen Prediction = **0.6682** (Probable **ANTIGEN**).

>EHO9888628.1 conjugal transfer protein TraE [Salmonella enterica subsp. enterica serovar Infantis] Overall Protective Antigen Prediction = **0.4724** (Probable **ANTIGEN**).

>EHO9888629.1 conjugal transfer protein TraF [Salmonella enterica subsp. enterica serovar Infantis] Overall Protective Antigen Prediction = **0.6868** (Probable **ANTIGEN**).

>EHO9888630.1 histidine phosphatase family protein [Salmonella enterica subsp. enterica serovar Infantis] Overall Protective Antigen Prediction = **0.3888** (Probable **NON-ANTIGEN**).

>EHO9888631.1 IncI1-type conjugal transfer lipoprotein TraH [Salmonella enterica subsp. enterica serovar Infantis] Overall Protective Antigen Prediction = **0.4308** (Probable **ANTIGEN**).

>EHO9888632.1 IncI1-type conjugal transfer lipoprotein TraI [Salmonella enterica subsp. enterica serovar Infantis] Overall Protective Antigen Prediction = **0.5685** (Probable **ANTIGEN**).

>EHO9888633.1 plasmid transfer ATPase TraJ [Salmonella enterica subsp. enterica serovar Infantis] Overall Protective Antigen Prediction = **0.5869** (Probable **ANTIGEN**).

>EHO9888634.1 conjugal transfer protein [Salmonella enterica subsp. enterica serovar Infantis] Overall Protective Antigen Prediction = **0.4222** (Probable **ANTIGEN**).

>EHO9888635.1 phospholipase D family protein [Salmonella enterica subsp. enterica serovar Infantis] Overall Protective Antigen Prediction = **0.6901** (Probable **ANTIGEN**).

>EHO9888636.1 DNA primase [Salmonella enterica subsp. enterica serovar Infantis] Overall Protective Antigen Prediction = **0.5279** (Probable **ANTIGEN**).

>EHO9888637.1 conjugal transfer protein [Salmonella enterica subsp. enterica serovar Infantis] Overall Protective Antigen Prediction = **0.5293** (Probable **ANTIGEN**).

>EHO9888638.1 DotI/IcmL family type IV secretion protein [Salmonella enterica subsp. enterica serovar Infantis] Overall Protective Antigen Prediction = **0.5435** (Probable **ANTIGEN**).

>EHO9888639.1 IncI1-type conjugal transfer protein TraN [Salmonella enterica subsp. enterica serovar Infantis] Overall Protective Antigen Prediction = **0.6290** (Probable **ANTIGEN**).

>EHO9888640.1 conjugal transfer protein TraO [Salmonella enterica subsp. enterica serovar Infantis] Overall Protective Antigen Prediction = **0.6342** (Probable **ANTIGEN**).

>EHO9888641.1 IncI1-type conjugal transfer protein TraP [Salmonella enterica subsp. enterica serovar Infantis] Overall Protective Antigen Prediction = **0.5579** (Probable **ANTIGEN**).

>EHO9888642.1 conjugal transfer protein TraQ [Salmonella enterica subsp. enterica serovar Infantis] Overall Protective Antigen Prediction = **0.2817** (Probable **NON-ANTIGEN**).

>EHO9888643.1 IncI1-type conjugal transfer protein TraR [Salmonella enterica subsp. enterica serovar Infantis] Overall Protective Antigen Prediction = **0.4302** (Probable **ANTIGEN**).

>EHO9888644.1 putative conjugal transfer protein TraS [Salmonella enterica subsp. enterica serovar Infantis] Overall Protective Antigen Prediction = **0.3183** (Probable **NON-ANTIGEN**).

>EHO9888645.1 IncI1-type conjugal transfer protein TraT [Salmonella enterica subsp. enterica serovar Infantis] Overall Protective Antigen Prediction = **0.4657** (Probable **ANTIGEN**).

>EHO9888646.1 IncI1-type conjugal transfer protein TraU [Salmonella enterica subsp. enterica serovar Infantis] Overall Protective Antigen Prediction = **0.3644** (Probable **NON-ANTIGEN**).

>EHO9888647.1 IncI1-type conjugal transfer protein TraV [Salmonella enterica subsp. enterica serovar Infantis] Overall Protective Antigen Prediction = **0.5273** (Probable **ANTIGEN**).

>EHO9888648.1 IncI1-type conjugal transfer protein TraW [Salmonella enterica subsp. enterica serovar Infantis] Overall Protective Antigen Prediction = **0.5637** (Probable **ANTIGEN**).

>EHO9888649.1 IncI1-type conjugal transfer protein TraX [Salmonella enterica subsp. enterica serovar Infantis] Overall Protective Antigen Prediction = **0.3095** (Probable **NON-ANTIGEN**).

>EHO9888650.1 DotA/TraY family protein [Salmonella enterica subsp. enterica serovar Infantis] Overall Protective Antigen Prediction = **0.6079** (Probable **ANTIGEN**).

>EHO9888651.1 plasmid IncI1-type surface exclusion protein ExcA [Salmonella enterica subsp. enterica serovar Infantis] Overall Protective Antigen Prediction = **0.6110** (Probable **ANTIGEN**).

>EHO9888652.1 HEAT repeat domain-containing protein [Salmonella enterica subsp. enterica serovar Infantis] Overall Protective Antigen Prediction = **0.4781** (Probable **ANTIGEN**).

>EHO9888653.1 hypothetical protein KND05_004099 [Salmonella enterica subsp. enterica serovar Infantis] Overall Protective Antigen Prediction = **0.0112** (Probable **NON-ANTIGEN**).

>EHO9888654.1 hypothetical protein KND05_004100 [Salmonella enterica subsp. enterica serovar Infantis] Overall Protective Antigen Prediction = **0.2409** (Probable **NON-ANTIGEN**).

>EHO9888655.1 hypothetical protein KND05_004101 [Salmonella enterica subsp. enterica serovar Infantis] Overall Protective Antigen Prediction = **0.5283** (Probable **ANTIGEN**).

>EHO9888656.1 Hok/Gef family protein [Salmonella enterica subsp. enterica serovar Infantis] Overall Protective Antigen Prediction = **0.7538** (Probable **ANTIGEN**).

>EHO9888657.1 protein finQ [Salmonella enterica subsp. enterica serovar Infantis] Overall Protective Antigen Prediction = **0.5718** (Probable **ANTIGEN**).

>EHO9888658.1 IncI1-type conjugal transfer protein TrbA [Salmonella enterica subsp. enterica serovar Infantis] Overall Protective Antigen Prediction = **0.4676** (Probable **ANTIGEN**).

>EHO9888659.1 IncI1-type conjugal transfer protein TrbB [Salmonella enterica subsp. enterica serovar Infantis] Overall Protective Antigen Prediction = **0.4398** (Probable **ANTIGEN**).

>EHO9888660.1 conjugal transfer protein TrbC [Salmonella enterica subsp. enterica serovar Infantis] Overall Protective Antigen Prediction = **0.5060** (Probable **ANTIGEN**).

>EHO9888661.1 IncI1-type relaxase NikB [Salmonella enterica subsp. enterica serovar Infantis] Overall Protective Antigen Prediction = **0.4790** (Probable **ANTIGEN**).

>EHO9888662.1 IncI1-type relaxosome accessory protein NikA [Salmonella enterica subsp. enterica serovar Infantis] Overall Protective Antigen Prediction = **0.5644** (Probable **ANTIGEN**).

>EHO9888663.1 molybdopterin-guanine dinucleotide biosynthesis protein MobC [Salmonella enterica subsp. enterica serovar Infantis] Overall Protective Antigen Prediction = **0.4391** (Probable **ANTIGEN**).

>EHO9888664.1 DUF4942 domain-containing protein [Salmonella enterica subsp. enterica serovar Infantis] Overall Protective Antigen Prediction = **0.3151** (Probable **NON-ANTIGEN**).

>EHO9888665.1 hypothetical protein KND05_004111 [Salmonella enterica subsp. enterica serovar

Infantis] Overall Protective Antigen Prediction = **0.8599** (Probable **ANTIGEN**).

>EHO9888666.1 helix-turn-helix transcriptional regulator [Salmonella enterica subsp. enterica serovar Infantis] Overall Protective Antigen Prediction = **0.2701** (Probable **NON-ANTIGEN**).

>EHO9888667.1 hypothetical protein KND05_004113 [Salmonella enterica subsp. enterica serovar Infantis] Overall Protective Antigen Prediction = **0.1486** (Probable **NON-ANTIGEN**).

>EHO9888668.1 Rpn family recombination-promoting nuclease/putative transposase [Salmonella enterica subsp. enterica serovar Infantis] Overall Protective Antigen Prediction = **0.4710** (Probable **ANTIGEN**).

>EHO9888669.1 hypothetical protein KND05_004115 [Salmonella enterica subsp. enterica serovar Infantis] Overall Protective Antigen Prediction = **0.4752** (Probable **ANTIGEN**).

>EHO9888670.1 post-segregation killing protein PndC [Salmonella enterica subsp. enterica serovar Infantis] Overall Protective Antigen Prediction = **0.5761** (Probable **ANTIGEN**).

>EHO9888671.1 antirestriction protein ArdA [Salmonella enterica subsp. enterica serovar Infantis] Overall Protective Antigen Prediction = **0.3377** (Probable **NON-ANTIGEN**).

>EHO9888672.1 hypothetical protein KND05_004118 [Salmonella enterica subsp. enterica serovar Infantis] Overall Protective Antigen Prediction = **0.2747** (Probable **NON-ANTIGEN**).

>EHO9888673.1 plasmid SOS inhibition protein A [Salmonella enterica subsp. enterica serovar Infantis] Overall Protective Antigen Prediction = **0.5142** (Probable **ANTIGEN**).

>EHO9888674.1 conjugation system SOS inhibitor PsiB [Salmonella enterica subsp. enterica serovar Infantis] Overall Protective Antigen Prediction = **0.5654** (Probable **ANTIGEN**).

>EHO9888675.1 chromosome partitioning protein ParB, partial [Salmonella enterica subsp. enterica serovar Infantis] Overall Protective Antigen Prediction = **0.7710** (Probable **ANTIGEN**).

>EHO9888676.1 hypothetical protein KND05_004124 [Salmonella enterica subsp. enterica serovar Infantis] Overall Protective Antigen Prediction = **0.4289** (Probable **ANTIGEN**).

>EHO9888677.1 multidrug efflux RND transporter permease subunit [Salmonella enterica subsp. enterica serovar Infantis] Overall Protective Antigen Prediction = **0.5997** (Probable **ANTIGEN**).

>EHO9888678.1 efflux RND transporter periplasmic adaptor subunit [Salmonella enterica subsp. enterica serovar Infantis] Overall Protective Antigen Prediction = **0.6876** (Probable **ANTIGEN**).

>EHO9888679.1 acrEF/envCD operon transcriptional regulator [Salmonella enterica subsp. enterica serovar Infantis] Overall Protective Antigen Prediction = **0.2230** (Probable **NON-ANTIGEN**).

>EHO9888680.1 EAL domain-containing protein [Salmonella enterica subsp. enterica serovar Infantis] Overall Protective Antigen Prediction = **0.4681** (Probable **ANTIGEN**).

>EHO9888681.1 DUF2556 family protein [Salmonella enterica subsp. enterica serovar Infantis] Overall Protective Antigen Prediction = **0.9432** (Probable **ANTIGEN**).

>EHO9888682.1 adenine-specific DNA-methyltransferase [Salmonella enterica subsp. enterica serovar Infantis] Overall Protective Antigen Prediction = **0.3073** (Probable **NON-ANTIGEN**).

>EHO9888683.1 DNA-binding transcriptional regulator Fis [Salmonella enterica subsp. enterica serovar Infantis] Overall Protective Antigen Prediction = **0.4281** (Probable **ANTIGEN**).

>EHO9888684.1 tRNA dihydrouridine synthase DusB [Salmonella enterica subsp. enterica serovar Infantis] Overall Protective Antigen Prediction = **0.4496** (Probable **ANTIGEN**).

>EHO9888685.1 50S ribosomal protein L11 methyltransferase [Salmonella enterica subsp. enterica serovar Infantis] Overall Protective Antigen Prediction = **0.5668** (Probable **ANTIGEN**).

>EHO9888686.1 sodium/pantothenate symporter [Salmonella enterica subsp. enterica serovar Infantis] Overall Protective Antigen Prediction = **0.5128** (Probable **ANTIGEN**).

>EHO9888687.1 YhdT family protein [Salmonella enterica subsp. enterica serovar Infantis] Overall Protective Antigen Prediction = **0.8600** (Probable **ANTIGEN**).

>EHO9888688.1 acetyl-CoA carboxylase biotin carboxylase subunit [Salmonella enterica subsp. enterica serovar Infantis] Overall Protective Antigen Prediction = **0.4861** (Probable **ANTIGEN**).

>EHO9888689.1 acetyl-CoA carboxylase biotin carboxyl carrier protein [Salmonella enterica subsp. enterica serovar Infantis] Overall Protective Antigen Prediction = **0.3343** (Probable **NON-ANTIGEN**).

>EHO9888690.1 protein-methionine-sulfoxide reductase heme-binding subunit MsrQ [Salmonella enterica subsp. enterica serovar Infantis] Overall Protective Antigen Prediction = **0.5456** (Probable **ANTIGEN**).

>EHO9888691.1 protein-methionine-sulfoxide reductase catalytic subunit MsrP [Salmonella enterica subsp. enterica serovar Infantis] Overall Protective Antigen Prediction = **0.3872** (Probable **NON-ANTIGEN**).

>EHO9888692.1 oxidoreductase [Salmonella enterica subsp. enterica serovar Infantis] Overall Protective Antigen Prediction = **0.5632** (Probable **ANTIGEN**).

>EHO9888693.1 RNase E specificity factor CsrD [Salmonella enterica subsp. enterica serovar Infantis] Overall Protective Antigen Prediction = **0.5302** (Probable **ANTIGEN**).

>EHO9888694.1 rod shape-determining protein MreB [Salmonella enterica subsp. enterica serovar Infantis] Overall Protective Antigen Prediction = **0.3889** (Probable **NON-ANTIGEN**).

>EHO9888695.1 rod shape-determining protein MreC [Salmonella enterica subsp. enterica serovar Infantis] Overall Protective Antigen Prediction = **0.5160** (Probable **ANTIGEN**).

>EHO9888696.1 rod shape-determining protein MreD [Salmonella enterica subsp. enterica serovar Infantis] Overall Protective Antigen Prediction = **0.9105** (Probable **ANTIGEN**).

>EHO9888697.1 septum formation inhibitor Maf [Salmonella enterica subsp. enterica serovar Infantis] Overall Protective Antigen Prediction = **0.4878** (Probable **ANTIGEN**).

>EHO9888698.1 ribonuclease G [Salmonella enterica subsp. enterica serovar Infantis] Overall Protective Antigen Prediction = **0.3949** (Probable **NON-ANTIGEN**).

>EHO9888699.1 AsmA2 domain-containing protein YhdP [Salmonella enterica subsp. enterica serovar Infantis] Overall Protective Antigen Prediction = **0.6577** (Probable **ANTIGEN**).

>EHO9888700.1 metalloprotease TldD [Salmonella enterica subsp. enterica serovar Infantis] Overall Protective Antigen Prediction = **0.6601** (Probable **ANTIGEN**).

>EHO9888701.1 HTH-type transcriptional activator AaeR [Salmonella enterica subsp. enterica serovar Infantis] Overall Protective Antigen Prediction = **0.3524** (Probable **NON-ANTIGEN**).

>EHO9888702.1 AaeX family protein [Salmonella enterica subsp. enterica serovar Infantis] Overall Protective Antigen Prediction = **0.8959** (Probable **ANTIGEN**).

>EHO9888703.1 p-hydroxybenzoic acid efflux pump subunit AaeA [Salmonella enterica subsp. enterica serovar Infantis] Overall Protective Antigen Prediction = **0.4719** (Probable **ANTIGEN**).

>EHO9888704.1 p-hydroxybenzoic acid efflux pump subunit AaeB [Salmonella enterica subsp. enterica serovar Infantis] Overall Protective Antigen Prediction = **0.4556** (Probable **ANTIGEN**).

>EHO9888705.1 hypothetical protein KND05_004153 [Salmonella enterica subsp. enterica serovar Infantis] Overall Protective Antigen Prediction = **0.6632** (Probable **ANTIGEN**).

>EHO9888706.1 peroxide/acid stress response protein YhcN [Salmonella enterica subsp. enterica serovar Infantis] Overall Protective Antigen Prediction = **0.5175** (Probable **ANTIGEN**).

>EHO9888707.1 peroxide/acid stress response protein YhcN [Salmonella enterica subsp. enterica serovar Infantis] Overall Protective Antigen Prediction = **0.6785** (Probable **ANTIGEN**).

>EHO9888708.1 transcriptional regulator ArgR [Salmonella enterica subsp. enterica serovar Infantis] Overall Protective Antigen Prediction = **0.2303** (Probable **NON-ANTIGEN**).

>EHO9888709.1 malate dehydrogenase [Salmonella enterica subsp. enterica serovar Infantis] Overall Protective Antigen Prediction = **0.4061** (Probable **ANTIGEN**).

>EHO9888710.1 GntR family transcriptional regulator [Salmonella enterica subsp. enterica serovar Infantis] Overall Protective Antigen Prediction = **0.3867** (Probable **NON-ANTIGEN**).

>EHO9888711.1 GntR family transcriptional regulator [Salmonella enterica subsp. enterica serovar Infantis] Overall Protective Antigen Prediction = **0.3612** (Probable **NON-ANTIGEN**).

>EHO9888712.1 SLC13/DASS family transporter [Salmonella enterica subsp. enterica serovar Infantis] Overall Protective Antigen Prediction = **0.4154** (Probable **ANTIGEN**).

>EHO9888713.1 L(+)-tartrate dehydratase subunit alpha [Salmonella enterica subsp. enterica serovar Infantis] Overall Protective Antigen Prediction = **0.4345** (Probable **ANTIGEN**).

>EHO9888714.1 L(+)-tartrate dehydratase subunit beta [Salmonella enterica subsp. enterica serovar Infantis] Overall Protective Antigen Prediction = **0.3380** (Probable **NON-ANTIGEN**).

>EHO9888715.1 oxaloacetate decarboxylase subunit gamma [Salmonella enterica subsp. enterica serovar Infantis] Overall Protective Antigen Prediction = **0.6031** (Probable **ANTIGEN**).

>EHO9888716.1 amidohydrolase family protein, partial [Salmonella enterica subsp. enterica serovar Infantis] Overall Protective Antigen Prediction = **0.3981** (Probable **NON-ANTIGEN**).

>EHO9888717.1 ABC transporter permease [Salmonella enterica subsp. enterica serovar Infantis] Overall Protective Antigen Prediction = **0.5860** (Probable **ANTIGEN**).

>EHO9888718.1 ribosome-associated ATPase/putative transporter RbbA [Salmonella enterica subsp. enterica serovar Infantis] Overall Protective Antigen Prediction = **0.4769** (Probable **ANTIGEN**).

>EHO9888719.1 HlyD family efflux transporter periplasmic adaptor subunit [Salmonella enterica subsp. enterica serovar Infantis] Overall Protective Antigen Prediction = **0.5923** (Probable **ANTIGEN**).

>EHO9888720.1 NAD(P)/FAD-dependent oxidoreductase [Salmonella enterica subsp. enterica serovar Infantis] Overall Protective Antigen Prediction = **0.4958** (Probable **ANTIGEN**).

>EHO9888721.1 inorganic phosphate transporter PitA [Salmonella enterica subsp. enterica serovar Infantis] Overall Protective Antigen Prediction = **0.5009** (Probable **ANTIGEN**).

>EHO9888722.1 universal stress protein UspB [Salmonella enterica subsp. enterica serovar Infantis] Overall Protective Antigen Prediction = **0.3904** (Probable **NON-ANTIGEN**).

>EHO9888723.1 universal stress protein UspA [Salmonella enterica subsp. enterica serovar Infantis] Overall Protective Antigen Prediction = **0.3416** (Probable **NON-ANTIGEN**).

>EHO9888724.1 dipeptide/tripeptide permease DtpB [Salmonella enterica subsp. enterica serovar Infantis] Overall Protective Antigen Prediction = **0.4548** (Probable **ANTIGEN**).

>EHO9888725.1 16S rRNA (guanine(1516)-N(2))-methyltransferase RsmJ [Salmonella enterica subsp. enterica serovar Infantis] Overall Protective Antigen Prediction = **0.4547** (Probable **ANTIGEN**).

>EHO9888726.1 oligopeptidase A [Salmonella enterica subsp. enterica serovar Infantis] Overall Protective Antigen Prediction = **0.3758** (Probable **NON-ANTIGEN**).

>EHO9888727.1 phosphatase PAP2 family protein [Salmonella enterica subsp. enterica serovar Infantis] Overall Protective Antigen Prediction = **0.5167** (Probable **ANTIGEN**).

>EHO9888728.1 23S rRNA (adenine(2030)-N(6))-methyltransferase RlmJ [Salmonella enterica subsp. enterica serovar Infantis] Overall Protective Antigen Prediction = **0.4816** (Probable **ANTIGEN**).

>EHO9888729.1 glutathione-disulfide reductase [Salmonella enterica subsp. enterica serovar Infantis] Overall Protective Antigen Prediction = **0.5840** (Probable **ANTIGEN**).

>EHO9888730.1 type II asparaginase [Salmonella enterica subsp. enterica serovar Infantis] Overall Protective Antigen Prediction = **0.4752** (Probable **ANTIGEN**).

>EHO9888731.1 anaerobic C4-dicarboxylate transporter [Salmonella enterica subsp. enterica serovar Infantis] Overall Protective Antigen Prediction = **0.4725** (Probable **ANTIGEN**).

>EHO9888732.1 fructoselysine 6-kinase [Salmonella enterica subsp. enterica serovar Infantis] Overall Protective Antigen Prediction = **0.4265** (Probable **ANTIGEN**).

>EHO9888733.1 SIS domain-containing protein [Salmonella enterica subsp. enterica serovar Infantis] Overall Protective Antigen Prediction = **0.4416** (Probable **ANTIGEN**).

>EHO9888734.1 GntR family transcriptional regulator [Salmonella enterica subsp. enterica serovar Infantis] Overall Protective Antigen Prediction = **0.3704** (Probable **NON-ANTIGEN**).

>EHO9888735.1 alpha,alpha-trehalase [Salmonella enterica subsp. enterica serovar Infantis] Overall Protective Antigen Prediction = **0.3247** (Probable **NON-ANTIGEN**).

>EHO9888736.1 hypothetical protein KND05_004184 [Salmonella enterica subsp. enterica serovar Infantis] Overall Protective Antigen Prediction = **0.4310** (Probable **ANTIGEN**).

>EHO9888737.1 lysozyme [Salmonella enterica subsp. enterica serovar Infantis] Overall Protective Antigen Prediction = **0.2819** (Probable **NON-ANTIGEN**).

>EHO9888738.1 response regulator transcription factor [Salmonella enterica subsp. enterica serovar Infantis] Overall Protective Antigen Prediction = **0.4421** (Probable **ANTIGEN**).

>EHO9888739.1 hypothetical protein KND05_004187 [Salmonella enterica subsp. enterica serovar Infantis] Overall Protective Antigen Prediction = **0.6855** (Probable **ANTIGEN**).

>EHO9888740.1 LysR family transcriptional regulator [Salmonella enterica subsp. enterica serovar Infantis] Overall Protective Antigen Prediction = **0.3189** (Probable **NON-ANTIGEN**).

>EHO9888741.1 inner membrane protein YhjD [Salmonella enterica subsp. enterica serovar Infantis] Overall Protective Antigen Prediction = **0.4990** (Probable **ANTIGEN**).

>EHO9888742.1 MHS family MFS transporter [Salmonella enterica subsp. enterica serovar Infantis] Overall Protective Antigen Prediction = **0.4771** (Probable **ANTIGEN**).

>EHO9888743.1 AsmA family protein [Salmonella enterica subsp. enterica serovar Infantis] Overall Protective Antigen Prediction = **0.7220** (Probable **ANTIGEN**).

>EHO9888744.1 cyclic-guanylate-specific phosphodiesterase [Salmonella enterica subsp. enterica serovar Infantis] Overall Protective Antigen Prediction = **0.2587** (Probable **NON-ANTIGEN**).

>EHO9888745.1 sugar kinase [Salmonella enterica subsp. enterica serovar Infantis] Overall Protective Antigen Prediction = **0.4146** (Probable **ANTIGEN**).

>EHO9888746.1 insulinase family protein [Salmonella enterica subsp. enterica serovar Infantis] Overall Protective Antigen Prediction = **0.5081** (Probable **ANTIGEN**).

>EHO9888747.1 C4-dicarboxylate transporter DctC [Salmonella enterica subsp. enterica serovar Infantis] Overall Protective Antigen Prediction = **0.4207** (Probable **ANTIGEN**).

>EHO9888748.1 biofilm formation regulator HmsP [Salmonella enterica subsp. enterica serovar Infantis] Overall Protective Antigen Prediction = **0.3873** (Probable **NON-ANTIGEN**).

>EHO9888749.1 cellulose biosynthesis protein BcsC, partial [Salmonella enterica subsp. enterica serovar Infantis] Overall Protective Antigen Prediction = **0.5739** (Probable **ANTIGEN**).

>EHO9888750.1 DUF1493 family protein [Salmonella enterica subsp. enterica serovar Infantis] Overall Protective Antigen Prediction = **0.5940** (Probable **ANTIGEN**).

>EHO9888751.1 cytoplasmic protein [Salmonella enterica subsp. enterica serovar Infantis] Overall Protective Antigen Prediction = **0.5886** (Probable **ANTIGEN**).

>EHO9888752.1 AMP nucleosidase [Salmonella enterica subsp. enterica serovar Infantis] Overall Protective Antigen Prediction = **0.4601** (Probable **ANTIGEN**).

>EHO9888753.1 SIR2 family protein [Salmonella enterica subsp. enterica serovar Infantis] Overall Protective Antigen Prediction = **0.3353** (Probable **NON-ANTIGEN**).

>EHO9888754.1 ParA family protein [Salmonella enterica subsp. enterica serovar Infantis] Overall Protective Antigen Prediction = **0.3145** (Probable **NON-ANTIGEN**).

>EHO9888755.1 hypothetical protein KND05_004204 [Salmonella enterica subsp. enterica serovar Infantis] Overall Protective Antigen Prediction = **0.3542** (Probable **NON-ANTIGEN**).

>EHO9888756.1 peptidase [Salmonella enterica subsp. enterica serovar Infantis] Overall Protective Antigen Prediction = **0.6447** (Probable **ANTIGEN**).

>EHO9888757.1 DgsA anti-repressor MtfA [Salmonella enterica subsp. enterica serovar Infantis] Overall Protective Antigen Prediction = **0.6097** (Probable **ANTIGEN**).

>EHO9888758.1 hypothetical protein KND05_004212 [Salmonella enterica subsp. enterica serovar Infantis] Overall Protective Antigen Prediction = **0.7128** (Probable **ANTIGEN**).

>EHO9888759.1 translesion error-prone DNA polymerase V autoproteolytic subunit [Salmonella enterica subsp. enterica serovar Infantis] Overall Protective Antigen Prediction = **0.4581** (Probable **ANTIGEN**).

>EHO9888760.1 Y-family DNA polymerase [Salmonella enterica subsp. enterica serovar Infantis] Overall Protective Antigen Prediction = **0.3566** (Probable **NON-ANTIGEN**).

>EHO9888761.1 cold-shock protein [Salmonella enterica subsp. enterica serovar Infantis] Overall Protective Antigen Prediction = **0.2919** (Probable **NON-ANTIGEN**).

>EHO9888762.1 cold-shock protein [Salmonella enterica subsp. enterica serovar Infantis] Overall Protective Antigen Prediction = **0.5280** (Probable **ANTIGEN**).

>EHO9888763.1 porin OmpS1 [Salmonella enterica subsp. enterica serovar Infantis] Overall Protective Antigen Prediction = **0.9057** (Probable **ANTIGEN**).

>EHO9888764.1 hypothetical protein KND05_004218 [Salmonella enterica subsp. enterica serovar Infantis] Overall Protective Antigen Prediction = **0.6867** (Probable **ANTIGEN**).

>EHO9888765.1 phosphohydrolase [Salmonella enterica subsp. enterica serovar Infantis] Overall Protective Antigen Prediction = **0.2629** (Probable **NON-ANTIGEN**).

>EHO9888766.1 DNA cytosine methyltransferase [Salmonella enterica subsp. enterica serovar Infantis] Overall Protective Antigen Prediction = **0.4115** (Probable **ANTIGEN**).

>EHO9888767.1 very short patch repair endonuclease [Salmonella enterica subsp. enterica serovar Infantis] Overall Protective Antigen Prediction = **0.6292** (Probable **ANTIGEN**).

>EHO9888768.1 drug/metabolite exporter YedA [Salmonella enterica subsp. enterica serovar Infantis] Overall Protective Antigen Prediction = **0.3613** (Probable **NON-ANTIGEN**).

>EHO9888769.1 DUF808 domain-containing protein [Salmonella enterica subsp. enterica serovar Infantis] Overall Protective Antigen Prediction = **0.2402** (Probable **NON-ANTIGEN**).

>EHO9888770.1 YodC family protein [Salmonella enterica subsp. enterica serovar Infantis] Overall Protective Antigen Prediction = **0.7444** (Probable **ANTIGEN**).

>EHO9888771.1 cellulose biosynthesis regulator YedQ [Salmonella enterica subsp. enterica serovar Infantis] Overall Protective Antigen Prediction = **0.3876** (Probable **NON-ANTIGEN**).

>EHO9888772.1 mannosyl-3-phosphoglycerate phosphatase-related protein [Salmonella enterica subsp. enterica serovar Infantis] Overall Protective Antigen Prediction = **0.4672** (Probable **ANTIGEN**).

>EHO9888773.1 YodD family peroxide/acid resistance protein [Salmonella enterica subsp. enterica serovar Infantis] Overall Protective Antigen Prediction = **0.7938** (Probable **ANTIGEN**).

>EHO9888774.1 protein DsrB [Salmonella enterica subsp. enterica serovar Infantis] Overall Protective Antigen Prediction = **0.5937** (Probable **ANTIGEN**).

>EHO9888775.1 transcriptional regulator RcsA [Salmonella enterica subsp. enterica serovar Infantis] Overall Protective Antigen Prediction = **0.4231** (Probable **ANTIGEN**).

>EHO9888776.1 flagellar type III secretion system protein FliR [Salmonella enterica subsp. enterica serovar Infantis] Overall Protective Antigen Prediction = **0.6192** (Probable **ANTIGEN**).

>EHO9888777.1 flagellar biosynthesis protein FliQ [Salmonella enterica subsp. enterica serovar Infantis] Overall Protective Antigen Prediction = **0.4222** (Probable **ANTIGEN**).

>EHO9888778.1 flagellar type III secretion system pore protein FliP [Salmonella enterica subsp. enterica serovar Infantis] Overall Protective Antigen Prediction = **0.6145** (Probable **ANTIGEN**).

>EHO9888779.1 flagellar type III secretion system protein FliO [Salmonella enterica subsp. enterica serovar Infantis] Overall Protective Antigen Prediction = **0.3658** (Probable **NON-ANTIGEN**).

>EHO9888780.1 flagellar motor switch protein FliN [Salmonella enterica subsp. enterica serovar Infantis] Overall Protective Antigen Prediction = **0.5635** (Probable **ANTIGEN**).

>EHO9888781.1 flagellar motor switch protein FliM [Salmonella enterica subsp. enterica serovar Infantis] Overall Protective Antigen Prediction = **0.6244** (Probable **ANTIGEN**).

>EHO9888782.1 flagellar basal body-associated protein FliL [Salmonella enterica subsp. enterica serovar Infantis] Overall Protective Antigen Prediction = **0.5821** (Probable **ANTIGEN**).

>EHO9888783.1 flagellar hook length control protein FliK [Salmonella enterica subsp. enterica serovar Infantis] Overall Protective Antigen Prediction = **0.7428** (Probable **ANTIGEN**).

>EHO9888784.1 flagella biosynthesis chaperone FliJ [Salmonella enterica subsp. enterica serovar Infantis] Overall Protective Antigen Prediction = **0.4045** (Probable **ANTIGEN**).

>EHO9888785.1 flagellum-specific ATP synthase FliI [Salmonella enterica subsp. enterica serovar Infantis] Overall Protective Antigen Prediction = **0.2784** (Probable **NON-ANTIGEN**).

>EHO9888786.1 flagellar assembly protein FliH [Salmonella enterica subsp. enterica serovar Infantis] Overall Protective Antigen Prediction = **0.6589** (Probable **ANTIGEN**).

>EHO9888787.1 flagellar motor switch protein FliG [Salmonella enterica subsp. enterica serovar Infantis] Overall Protective Antigen Prediction = **0.3528** (Probable **NON-ANTIGEN**).

>EHO9888788.1 flagellar M-ring protein FliF [Salmonella enterica subsp. enterica serovar Infantis]

Overall Protective Antigen Prediction = **0.6686** (Probable **ANTIGEN**).

>EHO9888789.1 flagellar hook-basal body complex protein FliE [Salmonella enterica subsp. enterica serovar Infantis] Overall Protective Antigen Prediction = **0.7373** (Probable **ANTIGEN**).

>EHO9888790.1 hypothetical protein KND05_004244 [Salmonella enterica subsp. enterica serovar Infantis] Overall Protective Antigen Prediction = **0.3480** (Probable **NON-ANTIGEN**).

>EHO9888791.1 sulfurtransferase-like selenium metabolism protein YedF [Salmonella enterica subsp. enterica serovar Infantis] Overall Protective Antigen Prediction = **0.3762** (Probable **NON-ANTIGEN**).

>EHO9888792.1 selenium metabolism membrane protein YedE/FdhT [Salmonella enterica subsp. enterica serovar Infantis] Overall Protective Antigen Prediction = **0.4201** (Probable **ANTIGEN**).

>EHO9888793.1 lipoprotein [Salmonella enterica subsp. enterica serovar Infantis] Overall Protective Antigen Prediction = **0.3768** (Probable **NON-ANTIGEN**).

>EHO9888794.1 alpha-amylase [Salmonella enterica subsp. enterica serovar Infantis] Overall Protective Antigen Prediction = **0.4671** (Probable **ANTIGEN**).

>EHO9888795.1 flagella biosynthesis regulatory protein FliT [Salmonella enterica subsp. enterica serovar Infantis] Overall Protective Antigen Prediction = **0.0790** (Probable **NON-ANTIGEN**).

>EHO9888796.1 flagellar export chaperone FliS [Salmonella enterica subsp. enterica serovar Infantis] Overall Protective Antigen Prediction = **0.4301** (Probable **ANTIGEN**).

>EHO9888797.1 flagellar filament capping protein FliD [Salmonella enterica subsp. enterica serovar Infantis] Overall Protective Antigen Prediction = **0.6204** (Probable **ANTIGEN**).

>EHO9888798.1 flagellin FliC, partial [Salmonella enterica subsp. enterica serovar Infantis] Overall Protective Antigen Prediction = **0.6378** (Probable **ANTIGEN**).

>EHO9888799.1 yersiniabactin ABC transporter ATP-binding/permease protein YbtQ, partial [Salmonella enterica subsp. enterica serovar Infantis] Overall Protective Antigen Prediction = **0.6117** (Probable **ANTIGEN**).

>EHO9888800.1 yersiniabactin ABC transporter ATP-binding/permease protein YbtP [Salmonella enterica subsp. enterica serovar Infantis] Overall Protective Antigen Prediction = **0.4096** (Probable **ANTIGEN**).

>EHO9888801.1 yersiniabactin transcriptional regulator YbtA [Salmonella enterica subsp. enterica serovar Infantis] Overall Protective Antigen Prediction = **0.3714** (Probable **NON-ANTIGEN**).

>EHO9888802.1 yersiniabactin non-ribosomal peptide synthetase HMWP2 [Salmonella enterica subsp. enterica serovar Infantis] Overall Protective Antigen Prediction = **0.4255** (Probable **ANTIGEN**).

>EHO9888803.1 yersiniabactin polyketide synthase HMWP1 [Salmonella enterica subsp. enterica serovar Infantis] Overall Protective Antigen Prediction = **0.4114** (Probable **ANTIGEN**).

>EHO9888804.1 yersiniabactin biosynthesis oxidoreductase YbtU [Salmonella enterica subsp. enterica serovar Infantis] Overall Protective Antigen Prediction = **0.3351** (Probable **NON-ANTIGEN**).

>EHO9888805.1 yersiniabactin biosynthesis thioesterase YbtT [Salmonella enterica subsp. enterica serovar Infantis] Overall Protective Antigen Prediction = **0.3243** (Probable **NON-ANTIGEN**).

>EHO9888806.1 yersiniabactin biosynthesis salycil-AMP ligase YbtE [Salmonella enterica subsp. enterica serovar Infantis] Overall Protective Antigen Prediction = **0.4506** (Probable **ANTIGEN**).

>EHO9888807.1 siderophore yersiniabactin receptor FyuA [Salmonella enterica subsp. enterica serovar Infantis] Overall Protective Antigen Prediction = **0.6719** (Probable **ANTIGEN**).

>EHO9888808.1 N-acetylmuramoyl-L-alanine amidase [Salmonella enterica subsp. enterica serovar Infantis] Overall Protective Antigen Prediction = **0.4652** (Probable **ANTIGEN**).

>EHO9888809.1 hypothetical protein KND05_004263 [Salmonella enterica subsp. enterica serovar Infantis] Overall Protective Antigen Prediction = **0.6313** (Probable **ANTIGEN**).

>EHO9888810.1 methionine gamma-lyase [Salmonella enterica subsp. enterica serovar Infantis] Overall Protective Antigen Prediction = **0.3256** (Probable **NON-ANTIGEN**).

>EHO9888811.1 transporter [Salmonella enterica subsp. enterica serovar Infantis] Overall Protective Antigen Prediction = **0.5557** (Probable **ANTIGEN**).

>EHO9888812.1 sigma 54-interacting transcriptional regulator [Salmonella enterica subsp. enterica serovar Infantis] Overall Protective Antigen Prediction = **0.3831** (Probable **NON-ANTIGEN**).

>EHO9888813.1 helix-turn-helix domain-containing protein [Salmonella enterica subsp. enterica serovar Infantis] Overall Protective Antigen Prediction = **0.5441** (Probable **ANTIGEN**).

>EHO9888814.1 hypothetical protein KND05_004270 [Salmonella enterica subsp. enterica serovar Infantis] Overall Protective Antigen Prediction = **0.5115** (Probable **ANTIGEN**).

>EHO9888815.1 APH(6) family putative aminoglycoside O-phosphotransferase [Salmonella enterica subsp. enterica serovar Infantis] Overall Protective Antigen Prediction = **0.5451** (Probable **ANTIGEN**).

>EHO9888816.1 DNA-binding protein [Salmonella enterica subsp. enterica serovar Infantis] Overall Protective Antigen Prediction = **0.6440** (Probable **ANTIGEN**).

>EHO9888817.1 reverse transcriptase N-terminal domain-containing protein [Salmonella enterica subsp. enterica serovar Infantis] Overall Protective Antigen Prediction = **0.9468** (Probable **ANTIGEN**).

>EHO9888818.1 transposase, partial [Salmonella enterica subsp. enterica serovar Infantis] Overall Protective Antigen Prediction = **0.5617** (Probable **ANTIGEN**).

>EHO9888819.1 RNA helicase [Salmonella enterica subsp. enterica serovar Infantis] Overall Protective Antigen Prediction = **0.6246** (Probable **ANTIGEN**).

>EHO9888820.1 hypothetical protein KND05_004280 [Salmonella enterica subsp. enterica serovar Infantis] Overall Protective Antigen Prediction = **0.4660** (Probable **ANTIGEN**).

>EHO9888821.1 cytoplasmic protein [Salmonella enterica subsp. enterica serovar Infantis] Overall Protective Antigen Prediction = **0.5609** (Probable **ANTIGEN**).

>EHO9888822.1 SDR family oxidoreductase [Salmonella enterica subsp. enterica serovar Infantis] Overall Protective Antigen Prediction = **0.5245** (Probable **ANTIGEN**).

>EHO9888823.1 TonB system transport protein ExbD [Salmonella enterica subsp. enterica serovar Infantis] Overall Protective Antigen Prediction = **0.6510** (Probable **ANTIGEN**).

>EHO9888824.1 tol-pal system-associated acyl-CoA thioesterase [Salmonella enterica subsp. enterica serovar Infantis] Overall Protective Antigen Prediction = **0.5003** (Probable **ANTIGEN**).

>EHO9888825.1 cystathionine beta-lyase [Salmonella enterica subsp. enterica serovar Infantis] Overall Protective Antigen Prediction = **0.3876** (Probable **NON-ANTIGEN**).

>EHO9888826.1 DedA family protein [Salmonella enterica subsp. enterica serovar Infantis] Overall Protective Antigen Prediction = **0.1446** (Probable **NON-ANTIGEN**).

>EHO9888827.1 AraC family transcriptional regulator [Salmonella enterica subsp. enterica serovar Infantis] Overall Protective Antigen Prediction = **0.4212** (Probable **ANTIGEN**).

>EHO9888828.1 alcohol dehydrogenase [Salmonella enterica subsp. enterica serovar Infantis] Overall Protective Antigen Prediction = **0.4429** (Probable **ANTIGEN**).

>EHO9888829.1 2,5-didehydrogluconate reductase DkgA [Salmonella enterica subsp. enterica serovar Infantis] Overall Protective Antigen Prediction = **0.4650** (Probable **ANTIGEN**).

>EHO9888830.1 DASS family sodium-coupled anion symporter [Salmonella enterica subsp. enterica serovar Infantis] Overall Protective Antigen Prediction = **0.4535** (Probable **ANTIGEN**).

>EHO9888831.1 HIT family protein [Salmonella enterica subsp. enterica serovar Infantis] Overall Protective Antigen Prediction = **0.4526** (Probable **ANTIGEN**).

>EHO9888832.1 hypothetical protein KND05_004292 [Salmonella enterica subsp. enterica serovar Infantis] Overall Protective Antigen Prediction = **0.8788** (Probable **ANTIGEN**).

>EHO9888833.1 YgiQ family radical SAM protein [Salmonella enterica subsp. enterica serovar Infantis] Overall Protective Antigen Prediction = **0.4160** (Probable **ANTIGEN**).

>EHO9888834.1 TRAP transporter substrate-binding protein [Salmonella enterica subsp. enterica serovar Infantis] Overall Protective Antigen Prediction = **0.4366** (Probable **ANTIGEN**).

>EHO9888835.1 TRAP transporter small permease [Salmonella enterica subsp. enterica serovar Infantis] Overall Protective Antigen Prediction = **0.5805** (Probable **ANTIGEN**).

>EHO9888836.1 TRAP transporter large permease subunit [Salmonella enterica subsp. enterica serovar Infantis] Overall Protective Antigen Prediction = **0.5723** (Probable **ANTIGEN**).

>EHO9888837.1 cell division protein FtsP [Salmonella enterica subsp. enterica serovar Infantis] Overall Protective Antigen Prediction = **0.4375** (Probable **ANTIGEN**).

>EHO9888838.1 1-acylglycerol-3-phosphate O-acyltransferase [Salmonella enterica subsp. enterica serovar Infantis] Overall Protective Antigen Prediction = **0.4990** (Probable **ANTIGEN**).

>EHO9888839.1 DNA topoisomerase IV subunit A [Salmonella enterica subsp. enterica serovar Infantis] Overall Protective Antigen Prediction = **0.4501** (Probable **ANTIGEN**).

>EHO9888840.1 AraC family transcriptional regulator [Salmonella enterica subsp. enterica serovar Infantis] Overall Protective Antigen Prediction = **0.4394** (Probable **ANTIGEN**).

>EHO9888841.1 YgiW/YdeI family stress tolerance OB fold protein [Salmonella enterica subsp. enterica serovar Infantis] Overall Protective Antigen Prediction = **0.6168** (Probable **ANTIGEN**).

>EHO9888842.1 two-component system response regulator QseB [Salmonella enterica subsp. enterica serovar Infantis] Overall Protective Antigen Prediction = **0.5145** (Probable **ANTIGEN**).

>EHO9888843.1 two-component system sensor histidine kinase QseC [Salmonella enterica subsp. enterica serovar Infantis] Overall Protective Antigen Prediction = **0.5285** (Probable **ANTIGEN**).

>EHO9888844.1 NAD(P)H-dependent oxidoreductase [Salmonella enterica subsp. enterica serovar Infantis] Overall Protective Antigen Prediction = **0.3302** (Probable **NON-ANTIGEN**).

>EHO9888845.1 antibiotic biosynthesis monooxygenase [Salmonella enterica subsp. enterica serovar Infantis] Overall Protective Antigen Prediction = **0.5378** (Probable **ANTIGEN**).

>EHO9888846.1 DNA topoisomerase IV subunit B [Salmonella enterica subsp. enterica serovar Infantis] Overall Protective Antigen Prediction = **0.5247** (Probable **ANTIGEN**).

>EHO9888847.1 esterase YqiA [Salmonella enterica subsp. enterica serovar Infantis] Overall Protective Antigen Prediction = **0.4166** (Probable **ANTIGEN**).

>EHO9888848.1 3',5'-cyclic-AMP phosphodiesterase [Salmonella enterica subsp. enterica serovar Infantis] Overall Protective Antigen Prediction = **0.4064** (Probable **ANTIGEN**).

>EHO9888849.1 DUF1249 family protein [Salmonella enterica subsp. enterica serovar Infantis] Overall Protective Antigen Prediction = **0.4406** (Probable **ANTIGEN**).

>EHO9888850.1 ADP-ribose diphosphatase [Salmonella enterica subsp. enterica serovar Infantis] Overall Protective Antigen Prediction = **0.4792** (Probable **ANTIGEN**).

>EHO9888851.1 outer membrane channel protein TolC [Salmonella enterica subsp. enterica serovar Infantis] Overall Protective Antigen Prediction = **0.5590** (Probable **ANTIGEN**).

>EHO9888852.1 DUF1190 family protein [Salmonella enterica subsp. enterica serovar Infantis] Overall Protective Antigen Prediction = **0.7201** (Probable **ANTIGEN**).

>EHO9888853.1 glutathionylspermidine synthase family protein [Salmonella enterica subsp. enterica serovar Infantis] Overall Protective Antigen Prediction = **0.5243** (Probable **ANTIGEN**).

>EHO9888854.1 4,5-DOPA dioxygenase extradiol [Salmonella enterica subsp. enterica serovar Infantis] Overall Protective Antigen Prediction = **0.2810** (Probable **NON-ANTIGEN**).

>EHO9888855.1 zinc transporter ZupT [Salmonella enterica subsp. enterica serovar Infantis] Overall Protective Antigen Prediction = **0.3373** (Probable **NON-ANTIGEN**).

>EHO9888856.1 aryl-sulfate sulfotransferase [Salmonella enterica subsp. enterica serovar Infantis] Overall Protective Antigen Prediction = **0.4430** (Probable **ANTIGEN**).

>EHO9888857.1 thiol:disulfide interchange protein DsbA/DsbL [Salmonella enterica subsp. enterica serovar Infantis] Overall Protective Antigen Prediction = **0.3538** (Probable **NON-ANTIGEN**).

>EHO9888858.1 protein-disulfide oxidoreductase DsbI [Salmonella enterica subsp. enterica serovar Infantis] Overall Protective Antigen Prediction = **0.6208** (Probable **ANTIGEN**).

>EHO9888859.1 MBL fold metallo-hydrolase [Salmonella enterica subsp. enterica serovar Infantis] Overall Protective Antigen Prediction = **0.2340** (Probable **NON-ANTIGEN**).

>EHO9888860.1 YcbK family protein [Salmonella enterica subsp. enterica serovar Infantis] Overall Protective Antigen Prediction = **0.4650** (Probable **ANTIGEN**).

>EHO9888861.1 L,D-transpeptidase [Salmonella enterica subsp. enterica serovar Infantis] Overall Protective Antigen Prediction = **0.4668** (Probable **ANTIGEN**).

>EHO9888862.1 chromosome partition protein MukB [Salmonella enterica subsp. enterica serovar Infantis] Overall Protective Antigen Prediction = **0.5449** (Probable **ANTIGEN**).

>EHO9888863.1 chromosome partition protein MukE [Salmonella enterica subsp. enterica serovar Infantis] Overall Protective Antigen Prediction = **0.4484** (Probable **ANTIGEN**).

>EHO9888864.1 chromosome partition protein MukF [Salmonella enterica subsp. enterica serovar Infantis] Overall Protective Antigen Prediction = **0.4236** (Probable **ANTIGEN**).

>EHO9888865.1 tRNA uridine 5-oxyacetic acid(34) methyltransferase CmoM [Salmonella enterica subsp. enterica serovar Infantis] Overall Protective Antigen Prediction = **0.5252** (Probable **ANTIGEN**).

>EHO9888866.1 envelope biogenesis factor ElyC [Salmonella enterica subsp. enterica serovar Infantis] Overall Protective Antigen Prediction = **0.3847** (Probable **NON-ANTIGEN**).

>EHO9888867.1 YcbJ family phosphotransferase [Salmonella enterica subsp. enterica serovar Infantis] Overall Protective Antigen Prediction = **0.2745** (Probable **NON-ANTIGEN**).

>EHO9888868.1 3-deoxy-manno-octulosonate cytidylyltransferase [Salmonella enterica subsp. enterica serovar Infantis] Overall Protective Antigen Prediction = **0.4340** (Probable **ANTIGEN**).

>EHO9888869.1 protein YcaR [Salmonella enterica subsp. enterica serovar Infantis] Overall Protective Antigen Prediction = **0.0560** (Probable **NON-ANTIGEN**).

>EHO9888870.1 YcaQ family DNA glycosylase [Salmonella enterica subsp. enterica serovar Infantis] Overall Protective Antigen Prediction = **0.2859** (Probable **NON-ANTIGEN**).

>EHO9888871.1 tetraacyldisaccharide 4'-kinase [Salmonella enterica subsp. enterica serovar Infantis] Overall Protective Antigen Prediction = **0.5588** (Probable **ANTIGEN**).

>EHO9888872.1 lipid A ABC transporter ATP-binding protein/permease MsbA [Salmonella enterica subsp. enterica serovar Infantis] Overall Protective Antigen Prediction = **0.4006** (Probable **ANTIGEN**).

>EHO9888873.1 ComEC family protein [Salmonella enterica subsp. enterica serovar Infantis] Overall Protective Antigen Prediction = **0.5692** (Probable **ANTIGEN**).

>EHO9888874.1 integration host factor subunit beta [Salmonella enterica subsp. enterica serovar Infantis] Overall Protective Antigen Prediction = **0.6698** (Probable **ANTIGEN**).

>EHO9888875.1 30S ribosomal protein S1 [Salmonella enterica subsp. enterica serovar Infantis]

Overall Protective Antigen Prediction = **0.5018** (Probable **ANTIGEN**).

>EHO9888876.1 (d)CMP kinase [Salmonella enterica subsp. enterica serovar Infantis] Overall Protective Antigen Prediction = **0.5074** (Probable **ANTIGEN**).

>EHO9888877.1 M48 family metallopeptidase [Salmonella enterica subsp. enterica serovar Infantis] Overall Protective Antigen Prediction = **0.5490** (Probable **ANTIGEN**).

>EHO9888878.1 3-phosphoshikimate 1-carboxyvinyltransferase [Salmonella enterica subsp. enterica serovar Infantis] Overall Protective Antigen Prediction = **0.4856** (Probable **ANTIGEN**).

>EHO9888879.1 3-phosphoserine/phosphohydroxythreonine transaminase [Salmonella enterica subsp. enterica serovar Infantis] Overall Protective Antigen Prediction = **0.3697** (Probable **NON-ANTIGEN**).

>EHO9888880.1 DUF421 domain-containing protein [Salmonella enterica subsp. enterica serovar Infantis] Overall Protective Antigen Prediction = **0.5480** (Probable **ANTIGEN**).

>EHO9888881.1 YcaO-like family protein [Salmonella enterica subsp. enterica serovar Infantis] Overall Protective Antigen Prediction = **0.3609** (Probable **NON-ANTIGEN**).

>EHO9888882.1 formate transporter FocA [Salmonella enterica subsp. enterica serovar Infantis] Overall Protective Antigen Prediction = **0.4409** (Probable **ANTIGEN**).

>EHO9888883.1 formate C-acetyltransferase [Salmonella enterica subsp. enterica serovar Infantis] Overall Protective Antigen Prediction = **0.4660** (Probable **ANTIGEN**).

>EHO9888884.1 SPI-2 type III secretion system effector SopD2 [Salmonella enterica subsp. enterica serovar Infantis] Overall Protective Antigen Prediction = **0.3760** (Probable **NON-ANTIGEN**).

>EHO9888885.1 pyruvate formate lyase 1-activating protein [Salmonella enterica subsp. enterica serovar Infantis] Overall Protective Antigen Prediction = **0.3980** (Probable **NON-ANTIGEN**).

>EHO9888886.1 amino acid permease [Salmonella enterica subsp. enterica serovar Infantis] Overall Protective Antigen Prediction = **0.6751** (Probable **ANTIGEN**).

>EHO9888887.1 MFS transporter [Salmonella enterica subsp. enterica serovar Infantis] Overall Protective Antigen Prediction = **0.4056** (Probable **ANTIGEN**).

>EHO9888888.1 dimethyl sulfoxide reductase anchor subunit, partial [Salmonella enterica subsp. enterica serovar Infantis] Overall Protective Antigen Prediction = **0.4467** (Probable **ANTIGEN**).

>EHO9888889.1 class 1 integron integrase IntI1 [Salmonella enterica subsp. enterica serovar Infantis] Overall Protective Antigen Prediction = **0.4675** (Probable **ANTIGEN**).

>EHO9888890.1 ANT(3")-Ia family aminoglycoside nucleotidyltransferase AadA1 [Salmonella enterica subsp. enterica serovar Infantis] Overall Protective Antigen Prediction = **0.2619** (Probable **NON-ANTIGEN**).

>EHO9888891.1 quaternary ammonium compound efflux SMR transporter QacE delta 1 [Salmonella enterica subsp. enterica serovar Infantis] Overall Protective Antigen Prediction = **0.3843** (Probable **NON-ANTIGEN**).

>EHO9888892.1 sulfonamide-resistant dihydropteroate synthase Sul1 [Salmonella enterica subsp.

enterica serovar Infantis] Overall Protective Antigen Prediction = **0.3201** (Probable **NON-ANTIGEN**).

>EHO9888893.1 GNAT family N-acetyltransferase [Salmonella enterica subsp. enterica serovar Infantis] Overall Protective Antigen Prediction = **0.6325** (Probable **ANTIGEN**).

>EHO9888894.1 IS21-like element IS1326 family helper ATPase IstB [Salmonella enterica subsp. enterica serovar Infantis] Overall Protective Antigen Prediction = **0.4151** (Probable **ANTIGEN**).

>EHO9888895.1 IS21-like element IS1326 family transposase [Salmonella enterica subsp. enterica serovar Infantis] Overall Protective Antigen Prediction = **0.4533** (Probable **ANTIGEN**).

>EHO9888896.1 TniB family NTP-binding protein [Salmonella enterica subsp. enterica serovar Infantis] Overall Protective Antigen Prediction = **0.3411** (Probable **NON-ANTIGEN**).

>EHO9888897.1 DDE-type integrase/transposase/recombinase [Salmonella enterica subsp. enterica serovar Infantis] Overall Protective Antigen Prediction = **0.3684** (Probable **NON-ANTIGEN**).

>EHO9888898.1 EAL domain-containing protein [Salmonella enterica subsp. enterica serovar Infantis] Overall Protective Antigen Prediction = **0.4951** (Probable **ANTIGEN**).

>EHO9888899.1 broad-spectrum mercury transporter MerE [Salmonella enterica subsp. enterica serovar Infantis] Overall Protective Antigen Prediction = **0.1650** (Probable **NON-ANTIGEN**).

>EHO9888900.1 mercury resistance co-regulator MerD [Salmonella enterica subsp. enterica serovar Infantis] Overall Protective Antigen Prediction = **0.3857** (Probable **NON-ANTIGEN**).

>EHO9888901.1 mercury(II) reductase [Salmonella enterica subsp. enterica serovar Infantis] Overall Protective Antigen Prediction = **0.5596** (Probable **ANTIGEN**).

>EHO9888902.1 organomercurial transporter MerC [Salmonella enterica subsp. enterica serovar Infantis] Overall Protective Antigen Prediction = **0.2045** (Probable **NON-ANTIGEN**).

>EHO9888903.1 mercury resistance system periplasmic binding protein MerP [Salmonella enterica subsp. enterica serovar Infantis] Overall Protective Antigen Prediction = **0.3522** (Probable **NON-ANTIGEN**).

>EHO9888904.1 mercuric transport protein MerT [Salmonella enterica subsp. enterica serovar Infantis] Overall Protective Antigen Prediction = **0.4464** (Probable **ANTIGEN**).

>EHO9888905.1 Hg(II)-responsive transcriptional regulator [Salmonella enterica subsp. enterica serovar Infantis] Overall Protective Antigen Prediction = **0.3528** (Probable **NON-ANTIGEN**).

>EHO9888906.1 relaxase [Salmonella enterica subsp. enterica serovar Infantis] Overall Protective Antigen Prediction = **0.8136** (Probable **ANTIGEN**).

>EHO9888907.1 tetracycline resistance transcriptional repressor TetR(A) [Salmonella enterica subsp. enterica serovar Infantis] Overall Protective Antigen Prediction = **0.5897** (Probable **ANTIGEN**).

>EHO9888908.1 tetracycline efflux MFS transporter Tet(A) [Salmonella enterica subsp. enterica serovar Infantis] Overall Protective Antigen Prediction = **0.4535** (Probable **ANTIGEN**).

>EHO9888909.1 EamA family transporter [Salmonella enterica subsp. enterica serovar Infantis] Overall Protective Antigen Prediction = **0.5316** (Probable **ANTIGEN**).

>EHO9888910.1 cysteine hydrolase [Salmonella enterica subsp. enterica serovar Infantis] Overall Protective Antigen Prediction = **0.4888** (Probable **ANTIGEN**).

>EHO9888911.1 ash family protein [Salmonella enterica subsp. enterica serovar Infantis] Overall Protective Antigen Prediction = **0.4314** (Probable **ANTIGEN**).

>EHO9888912.1 conjugal transfer protein TraA [Salmonella enterica subsp. enterica serovar Infantis] Overall Protective Antigen Prediction = **0.5407** (Probable **ANTIGEN**).

>EHO9888913.1 transcription termination factor NusG [Salmonella enterica subsp. enterica serovar Infantis] Overall Protective Antigen Prediction = **0.4012** (Probable **ANTIGEN**).

>EHO9888914.1 conjugal transfer protein TraC [Salmonella enterica subsp. enterica serovar Infantis] Overall Protective Antigen Prediction = **0.3275** (Probable **NON-ANTIGEN**).

>EHO9888915.1 PilI type IV pilus biogenesis protein [Salmonella enterica subsp. enterica serovar Infantis] Overall Protective Antigen Prediction = **0.3256** (Probable **NON-ANTIGEN**).

>EHO9888916.1 hypothetical protein KND05_004377 [Salmonella enterica subsp. enterica serovar Infantis] Overall Protective Antigen Prediction = **0.5463** (Probable **ANTIGEN**).

>EHO9888917.1 type IV pilus biogenesis lipoprotein PilL [Salmonella enterica subsp. enterica serovar Infantis] Overall Protective Antigen Prediction = **0.5057** (Probable **ANTIGEN**).

>EHO9888918.1 type IV pilus biogenesis protein PilM [Salmonella enterica subsp. enterica serovar Infantis] Overall Protective Antigen Prediction = **0.3900** (Probable **NON-ANTIGEN**).

>EHO9888919.1 PilN family type IVB pilus formation outer membrane protein [Salmonella enterica subsp. enterica serovar Infantis] Overall Protective Antigen Prediction = **0.7700** (Probable **ANTIGEN**).

>EHO9888920.1 type 4b pilus protein PilO2 [Salmonella enterica subsp. enterica serovar Infantis] Overall Protective Antigen Prediction = **0.4820** (Probable **ANTIGEN**).

>EHO9888921.1 type IV pilus biogenesis protein PilP [Salmonella enterica subsp. enterica serovar Infantis] Overall Protective Antigen Prediction = **0.8111** (Probable **ANTIGEN**).

>EHO9888922.1 Flp pilus assembly complex ATPase component TadA [Salmonella enterica subsp. enterica serovar Infantis] Overall Protective Antigen Prediction = **0.3420** (Probable **NON-ANTIGEN**).

>EHO9888923.1 type II secretion system F family protein [Salmonella enterica subsp. enterica serovar Infantis] Overall Protective Antigen Prediction = **0.2772** (Probable **NON-ANTIGEN**).

>EHO9888924.1 pilus assembly protein PilX [Salmonella enterica subsp. enterica serovar Infantis] Overall Protective Antigen Prediction = **0.6723** (Probable **ANTIGEN**).

>EHO9888925.1 lytic transglycosylase domain-containing protein [Salmonella enterica subsp. enterica serovar Infantis] Overall Protective Antigen Prediction = **0.7100** (Probable **ANTIGEN**).

>EHO9888926.1 prepilin peptidase [Salmonella enterica subsp. enterica serovar Infantis] Overall Protective Antigen Prediction = **0.3950** (Probable **NON-ANTIGEN**).

>EHO9888927.1 shufflon system plasmid conjugative transfer pilus tip adhesin PilV, partial [Salmonella enterica subsp. enterica serovar Infantis] Overall Protective Antigen Prediction = **0.5348** (Probable **ANTIGEN**).

>EHO9888928.1 bifunctional phosphoribosylaminoimidazolecarboxamide formyltransferase/IMP cyclohydrolase [Salmonella enterica subsp. enterica serovar Infantis] Overall Protective Antigen Prediction = **0.5599** (Probable **ANTIGEN**).

>EHO9888929.1 phosphoribosylamine--glycine ligase [Salmonella enterica subsp. enterica serovar Infantis] Overall Protective Antigen Prediction = **0.4931** (Probable **ANTIGEN**).

>EHO9888930.1 sigma-54-dependent response regulator transcription factor ZraR [Salmonella enterica subsp. enterica serovar Infantis] Overall Protective Antigen Prediction = **0.4617** (Probable **ANTIGEN**).

>EHO9888931.1 two-component system sensor histidine kinase ZraS [Salmonella enterica subsp. enterica serovar Infantis] Overall Protective Antigen Prediction = **0.5549** (Probable **ANTIGEN**).

>EHO9888932.1 zinc resistance sensor/chaperone ZraP [Salmonella enterica subsp. enterica serovar Infantis] Overall Protective Antigen Prediction = **0.6306** (Probable **ANTIGEN**).

>EHO9888933.1 DUF1481 domain-containing protein [Salmonella enterica subsp. enterica serovar Infantis] Overall Protective Antigen Prediction = **0.4079** (Probable **ANTIGEN**).

>EHO9888934.1 DNA-binding protein HU-alpha [Salmonella enterica subsp. enterica serovar Infantis] Overall Protective Antigen Prediction = **0.5652** (Probable **ANTIGEN**).

>EHO9888935.1 YjaG family protein [Salmonella enterica subsp. enterica serovar Infantis] Overall Protective Antigen Prediction = **0.6317** (Probable **ANTIGEN**).

>EHO9888936.1 deoxyribonuclease V [Salmonella enterica subsp. enterica serovar Infantis] Overall Protective Antigen Prediction = **0.4717** (Probable **ANTIGEN**).

>EHO9888937.1 uroporphyrinogen decarboxylase [Salmonella enterica subsp. enterica serovar Infantis] Overall Protective Antigen Prediction = **0.4503** (Probable **ANTIGEN**).

>EHO9888938.1 NAD(+) diphosphatase [Salmonella enterica subsp. enterica serovar Infantis] Overall Protective Antigen Prediction = **0.3048** (Probable **NON-ANTIGEN**).

>EHO9888939.1 sigma D regulator [Salmonella enterica subsp. enterica serovar Infantis] Overall Protective Antigen Prediction = **0.4017** (Probable **ANTIGEN**).

>EHO9888940.1 phosphomethylpyrimidine synthase ThiC [Salmonella enterica subsp. enterica serovar Infantis] Overall Protective Antigen Prediction = **0.4410** (Probable **ANTIGEN**).

>EHO9888941.1 thiamine phosphate synthase [Salmonella enterica subsp. enterica serovar Infantis] Overall Protective Antigen Prediction = **0.6260** (Probable **ANTIGEN**).

>EHO9888942.1 HesA/MoeB/ThiF family protein [Salmonella enterica subsp. enterica serovar Infantis] Overall Protective Antigen Prediction = **0.3821** (Probable **NON-ANTIGEN**).

>EHO9888943.1 sulfur carrier protein ThiS [Salmonella enterica subsp. enterica serovar Infantis] Overall Protective Antigen Prediction = **0.5392** (Probable **ANTIGEN**).

>EHO9888944.1 thiazole synthase [Salmonella enterica subsp. enterica serovar Infantis] Overall Protective Antigen Prediction = **0.4663** (Probable **ANTIGEN**).

>EHO9888945.1 2-iminoacetate synthase ThiH [Salmonella enterica subsp. enterica serovar Infantis] Overall Protective Antigen Prediction = **0.5388** (Probable **ANTIGEN**).

>EHO9888946.1 type III secretion system effector arginine glycosyltransferase SseK1 [Salmonella enterica subsp. enterica serovar Infantis] Overall Protective Antigen Prediction = **0.1964** (Probable **NON-ANTIGEN**).

>EHO9888947.1 cytoplasmic protein [Salmonella enterica subsp. enterica serovar Infantis] Overall Protective Antigen Prediction = **0.3179** (Probable **NON-ANTIGEN**).

>EHO9888948.1 hypothetical protein KND05_004409 [Salmonella enterica subsp. enterica serovar Infantis] Overall Protective Antigen Prediction = **0.8568** (Probable **ANTIGEN**).

>EHO9888949.1 DNA-directed RNA polymerase subunit beta' [Salmonella enterica subsp. enterica serovar Infantis] Overall Protective Antigen Prediction = **0.4918** (Probable **ANTIGEN**).

>EHO9888950.1 DNA-directed RNA polymerase subunit beta [Salmonella enterica subsp. enterica serovar Infantis] Overall Protective Antigen Prediction = **0.4360** (Probable **ANTIGEN**).

>EHO9888951.1 50S ribosomal protein L7/L12 [Salmonella enterica subsp. enterica serovar Infantis] Overall Protective Antigen Prediction = **0.5569** (Probable **ANTIGEN**).

>EHO9888952.1 50S ribosomal protein L10 [Salmonella enterica subsp. enterica serovar Infantis] Overall Protective Antigen Prediction = **0.3436** (Probable **NON-ANTIGEN**).

>EHO9888953.1 50S ribosomal protein L1 [Salmonella enterica subsp. enterica serovar Infantis] Overall Protective Antigen Prediction = **0.6674** (Probable **ANTIGEN**).

>EHO9888954.1 50S ribosomal protein L11 [Salmonella enterica subsp. enterica serovar Infantis] Overall Protective Antigen Prediction = **0.5084** (Probable **ANTIGEN**).

>EHO9888955.1 transcription termination/antitermination protein NusG [Salmonella enterica subsp. enterica serovar Infantis] Overall Protective Antigen Prediction = **0.3657** (Probable **NON-ANTIGEN**).

>EHO9888956.1 preprotein translocase subunit SecE [Salmonella enterica subsp. enterica serovar Infantis] Overall Protective Antigen Prediction = **0.4957** (Probable **ANTIGEN**).

>EHO9888957.1 elongation factor Tu, partial [Salmonella enterica subsp. enterica serovar Infantis] Overall Protective Antigen Prediction = **0.4368** (Probable **ANTIGEN**).

>EHO9888958.1 elongation factor Tu, partial [Salmonella enterica subsp. enterica serovar Infantis] Overall Protective Antigen Prediction = **0.4129** (Probable **ANTIGEN**).

>EHO9888959.1 elongation factor G [Salmonella enterica subsp. enterica serovar Infantis] Overall Protective Antigen Prediction = **0.5393** (Probable **ANTIGEN**).

>EHO9888960.1 30S ribosomal protein S7 [Salmonella enterica subsp. enterica serovar Infantis] Overall Protective Antigen Prediction = **0.7520** (Probable **ANTIGEN**).

>EHO9888961.1 30S ribosomal protein S12 [Salmonella enterica subsp. enterica serovar Infantis]

Overall Protective Antigen Prediction = **0.8034** (Probable **ANTIGEN**).

>EHO9888962.1 sulfurtransferase complex subunit TusB [Salmonella enterica subsp. enterica serovar Infantis] Overall Protective Antigen Prediction = **0.4357** (Probable **ANTIGEN**).

>EHO9888963.1 sulfurtransferase complex subunit TusC [Salmonella enterica subsp. enterica serovar Infantis] Overall Protective Antigen Prediction = **0.3854** (Probable **NON-ANTIGEN**).

>EHO9888964.1 sulfurtransferase complex subunit TusD [Salmonella enterica subsp. enterica serovar Infantis] Overall Protective Antigen Prediction = **0.3574** (Probable **NON-ANTIGEN**).

>EHO9888965.1 transcriptional regulator [Salmonella enterica subsp. enterica serovar Infantis] Overall Protective Antigen Prediction = **0.5208** (Probable **ANTIGEN**).

>EHO9888966.1 FKBP-type peptidyl-prolyl cis-trans isomerase [Salmonella enterica subsp. enterica serovar Infantis] Overall Protective Antigen Prediction = **0.7141** (Probable **ANTIGEN**).

>EHO9888967.1 protein SlyX [Salmonella enterica subsp. enterica serovar Infantis] Overall Protective Antigen Prediction = **0.6803** (Probable **ANTIGEN**).

>EHO9888968.1 peptidylprolyl isomerase [Salmonella enterica subsp. enterica serovar Infantis] Overall Protective Antigen Prediction = **1.0154** (Probable **ANTIGEN**).

>EHO9888969.1 YheV family putative metal-binding protein [Salmonella enterica subsp. enterica serovar Infantis] Overall Protective Antigen Prediction = **0.3559** (Probable **NON-ANTIGEN**).

>EHO9888970.1 glutathione-regulated potassium-efflux system protein KefB [Salmonella enterica subsp. enterica serovar Infantis] Overall Protective Antigen Prediction = **0.3163** (Probable **NON-ANTIGEN**).

>EHO9888971.1 glutathione-regulated potassium-efflux system ancillary protein KefG [Salmonella enterica subsp. enterica serovar Infantis] Overall Protective Antigen Prediction = **0.4315** (Probable **ANTIGEN**).

>EHO9888972.1 ABC transporter ATP-binding protein [Salmonella enterica subsp. enterica serovar Infantis] Overall Protective Antigen Prediction = **0.4179** (Probable **ANTIGEN**).

>EHO9888973.1 LysR family transcriptional regulator [Salmonella enterica subsp. enterica serovar Infantis] Overall Protective Antigen Prediction = **0.5745** (Probable **ANTIGEN**).

>EHO9888974.1 monooxygenase [Salmonella enterica subsp. enterica serovar Infantis] Overall Protective Antigen Prediction = **0.4521** (Probable **ANTIGEN**).

>EHO9888975.1 hydrolase [Salmonella enterica subsp. enterica serovar Infantis] Overall Protective Antigen Prediction = **0.2904** (Probable **NON-ANTIGEN**).

>EHO9888976.1 YheU family protein [Salmonella enterica subsp. enterica serovar Infantis] Overall Protective Antigen Prediction = **0.4059** (Probable **ANTIGEN**).

>EHO9888977.1 phosphoribulokinase [Salmonella enterica subsp. enterica serovar Infantis] Overall Protective Antigen Prediction = **0.4934** (Probable **ANTIGEN**).

>EHO9888978.1 OsmC family protein [Salmonella enterica subsp. enterica serovar Infantis] Overall Protective Antigen Prediction = **0.5702** (Probable **ANTIGEN**).

>EHO9888979.1 cAMP-activated global transcriptional regulator CRP [Salmonella enterica subsp. enterica serovar Infantis] Overall Protective Antigen Prediction = **0.2730** (Probable **NON-ANTIGEN**).

>EHO9888980.1 FUSC family protein [Salmonella enterica subsp. enterica serovar Infantis] Overall Protective Antigen Prediction = **0.4081** (Probable **ANTIGEN**).

>EHO9888981.1 aspartate aminotransferase family protein [Salmonella enterica subsp. enterica serovar Infantis] Overall Protective Antigen Prediction = **0.4379** (Probable **ANTIGEN**).

>EHO9888982.1 aminodeoxychorismate synthase component 2 [Salmonella enterica subsp. enterica serovar Infantis] Overall Protective Antigen Prediction = **0.4512** (Probable **ANTIGEN**).

>EHO9888983.1 putative adenosine monophosphate-protein transferase Fic [Salmonella enterica subsp. enterica serovar Infantis] Overall Protective Antigen Prediction = **0.4023** (Probable **ANTIGEN**).

>EHO9888984.1 YhfG family protein [Salmonella enterica subsp. enterica serovar Infantis] Overall Protective Antigen Prediction = **0.3464** (Probable **NON-ANTIGEN**).

>EHO9888985.1 peptidylprolyl isomerase A [Salmonella enterica subsp. enterica serovar Infantis] Overall Protective Antigen Prediction = **0.4155** (Probable **ANTIGEN**).

>EHO9888986.1 MFS transporter TsgA [Salmonella enterica subsp. enterica serovar Infantis] Overall Protective Antigen Prediction = **0.5373** (Probable **ANTIGEN**).

>EHO9888987.1 NADPH-nitrite reductase large subunit [Salmonella enterica subsp. enterica serovar Infantis] Overall Protective Antigen Prediction = **0.4916** (Probable **ANTIGEN**).

>EHO9888988.1 nitrite reductase small subunit NirD [Salmonella enterica subsp. enterica serovar Infantis] Overall Protective Antigen Prediction = **0.3245** (Probable **NON-ANTIGEN**).

>EHO9888989.1 nitrite transporter NirC [Salmonella enterica subsp. enterica serovar Infantis] Overall Protective Antigen Prediction = **0.3879** (Probable **NON-ANTIGEN**).

>EHO9888990.1 uroporphyrinogen-III C-methyltransferase [Salmonella enterica subsp. enterica serovar Infantis] Overall Protective Antigen Prediction = **0.3557** (Probable **NON-ANTIGEN**).

>EHO9888991.1 hypothetical protein KND05_004452, partial [Salmonella enterica subsp. enterica serovar Infantis] Overall Protective Antigen Prediction = **0.7134** (Probable **ANTIGEN**).

>EHO9888992.1 gamma carbonic anhydrase family protein [Salmonella enterica subsp. enterica serovar Infantis] Overall Protective Antigen Prediction = **0.3731** (Probable **NON-ANTIGEN**).

>EHO9888993.1 DUF1488 domain-containing protein [Salmonella enterica subsp. enterica serovar Infantis] Overall Protective Antigen Prediction = **0.3766** (Probable **NON-ANTIGEN**).

>EHO9888994.1 shikimate dehydrogenase [Salmonella enterica subsp. enterica serovar Infantis] Overall Protective Antigen Prediction = **0.4042** (Probable **ANTIGEN**).

>EHO9888995.1 L-threonylcarbamoyladenylate synthase type 1 TsaC [Salmonella enterica subsp. enterica serovar Infantis] Overall Protective Antigen Prediction = **0.4072** (Probable **ANTIGEN**).

>EHO9888996.1 DNA topoisomerase [Salmonella enterica subsp. enterica serovar Infantis] Overall Protective Antigen Prediction = **0.2570** (Probable **NON-ANTIGEN**).

>EHO9888997.1 DUF494 domain-containing protein [Salmonella enterica subsp. enterica serovar Infantis] Overall Protective Antigen Prediction = **0.4793** (Probable **ANTIGEN**).

>EHO9888998.1 DNA-protecting protein DprA [Salmonella enterica subsp. enterica serovar Infantis] Overall Protective Antigen Prediction = **0.3568** (Probable **NON-ANTIGEN**).

>EHO9888999.1 peptide deformylase [Salmonella enterica subsp. enterica serovar Infantis] Overall Protective Antigen Prediction = **0.4309** (Probable **ANTIGEN**).

>EHO9889000.1 methionyl-tRNA formyltransferase [Salmonella enterica subsp. enterica serovar Infantis] Overall Protective Antigen Prediction = **0.4280** (Probable **ANTIGEN**).

>EHO9889001.1 16S rRNA (cytosine(967)-C(5))-methyltransferase RsmB [Salmonella enterica subsp. enterica serovar Infantis] Overall Protective Antigen Prediction = **0.3478** (Probable **NON-ANTIGEN**).

>EHO9889002.1 Trk system potassium transporter TrkA [Salmonella enterica subsp. enterica serovar Infantis] Overall Protective Antigen Prediction = **0.3245** (Probable **NON-ANTIGEN**).

>EHO9889003.1 large-conductance mechanosensitive channel protein MscL [Salmonella enterica subsp. enterica serovar Infantis] Overall Protective Antigen Prediction = **0.3128** (Probable **NON-ANTIGEN**).

>EHO9889004.1 alternative ribosome-rescue factor A [Salmonella enterica subsp. enterica serovar Infantis] Overall Protective Antigen Prediction = **0.6186** (Probable **ANTIGEN**).

>EHO9889005.1 Zn(2+)-responsive transcriptional regulator [Salmonella enterica subsp. enterica serovar Infantis] Overall Protective Antigen Prediction = **0.5830** (Probable **ANTIGEN**).

>EHO9889006.1 DUF1992 domain-containing protein [Salmonella enterica subsp. enterica serovar Infantis] Overall Protective Antigen Prediction = **0.4077** (Probable **ANTIGEN**).

>EHO9889007.1 50S ribosomal protein L17 [Salmonella enterica subsp. enterica serovar Infantis] Overall Protective Antigen Prediction = **0.4926** (Probable **ANTIGEN**).

>EHO9889008.1 DNA-directed RNA polymerase subunit alpha [Salmonella enterica subsp. enterica serovar Infantis] Overall Protective Antigen Prediction = **0.5519** (Probable **ANTIGEN**).

>EHO9889009.1 30S ribosomal protein S4 [Salmonella enterica subsp. enterica serovar Infantis] Overall Protective Antigen Prediction = **0.6586** (Probable **ANTIGEN**).

>EHO9889010.1 30S ribosomal protein S11 [Salmonella enterica subsp. enterica serovar Infantis] Overall Protective Antigen Prediction = **0.5659** (Probable **ANTIGEN**).

>EHO9889011.1 30S ribosomal protein S13 [Salmonella enterica subsp. enterica serovar Infantis] Overall Protective Antigen Prediction = **0.5589** (Probable **ANTIGEN**).

>EHO9889012.1 50S ribosomal protein L36 [Salmonella enterica subsp. enterica serovar Infantis] Overall Protective Antigen Prediction = **0.6244** (Probable **ANTIGEN**).

>EHO9889013.1 preprotein translocase subunit SecY [Salmonella enterica subsp. enterica serovar

Infantis] Overall Protective Antigen Prediction = **0.5234** (Probable **ANTIGEN**).

>EHO9889014.1 50S ribosomal protein L15 [Salmonella enterica subsp. enterica serovar Infantis] Overall Protective Antigen Prediction = **1.1049** (Probable **ANTIGEN**).

>EHO9889015.1 50S ribosomal protein L30 [Salmonella enterica subsp. enterica serovar Infantis] Overall Protective Antigen Prediction = **0.2701** (Probable **NON-ANTIGEN**).

>EHO9889016.1 30S ribosomal protein S5 [Salmonella enterica subsp. enterica serovar Infantis] Overall Protective Antigen Prediction = **0.4417** (Probable **ANTIGEN**).

>EHO9889017.1 50S ribosomal protein L18 [Salmonella enterica subsp. enterica serovar Infantis] Overall Protective Antigen Prediction = **0.4393** (Probable **ANTIGEN**).

>EHO9889018.1 50S ribosomal protein L6 [Salmonella enterica subsp. enterica serovar Infantis] Overall Protective Antigen Prediction = **0.7033** (Probable **ANTIGEN**).

>EHO9889019.1 30S ribosomal protein S8 [Salmonella enterica subsp. enterica serovar Infantis] Overall Protective Antigen Prediction = **0.4427** (Probable **ANTIGEN**).

>EHO9889020.1 30S ribosomal protein S14 [Salmonella enterica subsp. enterica serovar Infantis] Overall Protective Antigen Prediction = **0.4789** (Probable **ANTIGEN**).

>EHO9889021.1 50S ribosomal protein L5 [Salmonella enterica subsp. enterica serovar Infantis] Overall Protective Antigen Prediction = **0.5794** (Probable **ANTIGEN**).

>EHO9889022.1 50S ribosomal protein L24 [Salmonella enterica subsp. enterica serovar Infantis] Overall Protective Antigen Prediction = **0.5797** (Probable **ANTIGEN**).

>EHO9889023.1 50S ribosomal protein L14 [Salmonella enterica subsp. enterica serovar Infantis] Overall Protective Antigen Prediction = **0.4409** (Probable **ANTIGEN**).

>EHO9889024.1 30S ribosomal protein S17 [Salmonella enterica subsp. enterica serovar Infantis] Overall Protective Antigen Prediction = **0.1802** (Probable **NON-ANTIGEN**).

>EHO9889025.1 50S ribosomal protein L29 [Salmonella enterica subsp. enterica serovar Infantis] Overall Protective Antigen Prediction = **0.5750** (Probable **ANTIGEN**).

>EHO9889026.1 50S ribosomal protein L16 [Salmonella enterica subsp. enterica serovar Infantis] Overall Protective Antigen Prediction = **0.3948** (Probable **NON-ANTIGEN**).

>EHO9889027.1 30S ribosomal protein S3 [Salmonella enterica subsp. enterica serovar Infantis] Overall Protective Antigen Prediction = **0.5633** (Probable **ANTIGEN**).

>EHO9889028.1 50S ribosomal protein L22 [Salmonella enterica subsp. enterica serovar Infantis] Overall Protective Antigen Prediction = **0.3912** (Probable **NON-ANTIGEN**).

>EHO9889029.1 30S ribosomal protein S19 [Salmonella enterica subsp. enterica serovar Infantis] Overall Protective Antigen Prediction = **0.4921** (Probable **ANTIGEN**).

>EHO9889030.1 50S ribosomal protein L2 [Salmonella enterica subsp. enterica serovar Infantis] Overall Protective Antigen Prediction = **0.7733** (Probable **ANTIGEN**).

>EHO9889031.1 50S ribosomal protein L23 [Salmonella enterica subsp. enterica serovar Infantis]

Overall Protective Antigen Prediction = **0.5721** (Probable **ANTIGEN**).

>EHO9889032.1 50S ribosomal protein L4 [Salmonella enterica subsp. enterica serovar Infantis] Overall Protective Antigen Prediction = **0.5019** (Probable **ANTIGEN**).

>EHO9889033.1 50S ribosomal protein L3 [Salmonella enterica subsp. enterica serovar Infantis] Overall Protective Antigen Prediction = **0.7534** (Probable **ANTIGEN**).

>EHO9889034.1 30S ribosomal protein S10 [Salmonella enterica subsp. enterica serovar Infantis] Overall Protective Antigen Prediction = **0.7148** (Probable **ANTIGEN**).

>EHO9889035.1 leader peptidase HopD [Salmonella enterica subsp. enterica serovar Infantis] Overall Protective Antigen Prediction = **0.3941** (Probable **NON-ANTIGEN**).

>EHO9889036.1 bacterioferritin [Salmonella enterica subsp. enterica serovar Infantis] Overall Protective Antigen Prediction = **0.2714** (Probable **NON-ANTIGEN**).

>EHO9889037.1 bacterioferritin-associated ferredoxin [Salmonella enterica subsp. enterica serovar Infantis] Overall Protective Antigen Prediction = **0.3616** (Probable **NON-ANTIGEN**).

>EHO9889038.1 elongation factor Tu, partial [Salmonella enterica subsp. enterica serovar Infantis] Overall Protective Antigen Prediction = **0.4368** (Probable **ANTIGEN**).

>EHO9889039.1 type II secretion system protein, partial [Salmonella enterica subsp. enterica serovar Infantis] Overall Protective Antigen Prediction = **0.3967** (Probable **NON-ANTIGEN**).

>EHO9889040.1 lytic transglycosylase domain-containing protein [Salmonella enterica subsp. enterica serovar Infantis] Overall Protective Antigen Prediction = **0.2901** (Probable **NON-ANTIGEN**).

>EHO9889041.1 prepilin peptidase [Salmonella enterica subsp. enterica serovar Infantis] Overall Protective Antigen Prediction = **0.7568** (Probable **ANTIGEN**).

>EHO9889042.1 KUP/HAK/KT family potassium transporter [Salmonella enterica subsp. enterica serovar Infantis] Overall Protective Antigen Prediction = **0.4199** (Probable **ANTIGEN**).

>EHO9889043.1 hypothetical protein KND05_004505 [Salmonella enterica subsp. enterica serovar Infantis] Overall Protective Antigen Prediction = **0.5391** (Probable **ANTIGEN**).

>EHO9889044.1 replication initiation protein [Salmonella enterica subsp. enterica serovar Infantis] Overall Protective Antigen Prediction = **0.5350** (Probable **ANTIGEN**).

>EHO9889045.1 plasmid-partitioning protein SopA [Salmonella enterica subsp. enterica serovar Infantis] Overall Protective Antigen Prediction = **0.5954** (Probable **ANTIGEN**).

>EHO9889046.1 ParB/RepB/Spo0J family plasmid partition protein [Salmonella enterica subsp. enterica serovar Infantis] Overall Protective Antigen Prediction = **0.3595** (Probable **NON-ANTIGEN**).

>EHO9889047.1 transposase [Salmonella enterica subsp. enterica serovar Infantis] Overall Protective Antigen Prediction = **0.4746** (Probable **ANTIGEN**).

>EHO9889048.1 DNA repair protein RadC [Salmonella enterica subsp. enterica serovar Infantis] Overall Protective Antigen Prediction = **0.4383** (Probable **ANTIGEN**).

>EHO9889049.1 MFS transporter [Salmonella enterica subsp. enterica serovar Infantis] Overall Protective Antigen Prediction = **0.5063** (Probable **ANTIGEN**).

>EHO9889050.1 2,5-didehydrogluconate reductase DkgB [Salmonella enterica subsp. enterica serovar Infantis] Overall Protective Antigen Prediction = **0.4261** (Probable **ANTIGEN**).

>EHO9889051.1 LysR family transcriptional regulator [Salmonella enterica subsp. enterica serovar Infantis] Overall Protective Antigen Prediction = **0.4394** (Probable **ANTIGEN**).

>EHO9889052.1 MFS transporter [Salmonella enterica subsp. enterica serovar Infantis] Overall Protective Antigen Prediction = **0.4264** (Probable **ANTIGEN**).

>EHO9889053.1 endonuclease/exonuclease/phosphatase family protein [Salmonella enterica subsp. enterica serovar Infantis] Overall Protective Antigen Prediction = **0.2656** (Probable **NON-ANTIGEN**).

>EHO9889054.1 class I SAM-dependent methyltransferase [Salmonella enterica subsp. enterica serovar Infantis] Overall Protective Antigen Prediction = **0.3513** (Probable **NON-ANTIGEN**).

>EHO9889055.1 murein transglycosylase D [Salmonella enterica subsp. enterica serovar Infantis] Overall Protective Antigen Prediction = **0.6085** (Probable **ANTIGEN**).

>EHO9889056.1 hydroxyacylglutathione hydrolase [Salmonella enterica subsp. enterica serovar Infantis] Overall Protective Antigen Prediction = **0.3676** (Probable **NON-ANTIGEN**).

>EHO9889057.1 class I SAM-dependent methyltransferase [Salmonella enterica subsp. enterica serovar Infantis] Overall Protective Antigen Prediction = **0.3000** (Probable **NON-ANTIGEN**).

>EHO9889058.1 ribonuclease HI [Salmonella enterica subsp. enterica serovar Infantis] Overall Protective Antigen Prediction = **0.4881** (Probable **ANTIGEN**).

>EHO9889059.1 DNA polymerase III subunit epsilon [Salmonella enterica subsp. enterica serovar Infantis] Overall Protective Antigen Prediction = **0.4800** (Probable **ANTIGEN**).

>EHO9889060.1 ImpA family type VI secretion system protein [Salmonella enterica subsp. enterica serovar Infantis] Overall Protective Antigen Prediction = **0.3481** (Probable **NON-ANTIGEN**).

>EHO9889061.1 type VI secretion system baseplate subunit TssG [Salmonella enterica subsp. enterica serovar Infantis] Overall Protective Antigen Prediction = **0.5034** (Probable **ANTIGEN**).

>EHO9889062.1 type VI secretion system baseplate subunit TssF [Salmonella enterica subsp. enterica serovar Infantis] Overall Protective Antigen Prediction = **0.5490** (Probable **ANTIGEN**).

>EHO9889063.1 type VI secretion system baseplate subunit TssE [Salmonella enterica subsp. enterica serovar Infantis] Overall Protective Antigen Prediction = **0.4807** (Probable **ANTIGEN**).

>EHO9889064.1 impE family protein [Salmonella enterica subsp. enterica serovar Infantis] Overall Protective Antigen Prediction = **0.5282** (Probable **ANTIGEN**).

>EHO9889065.1 type VI secretion system-associated protein TagK [Salmonella enterica subsp. enterica serovar Infantis] Overall Protective Antigen Prediction = **0.6039** (Probable **ANTIGEN**).

>EHO9889066.1 type VI secretion system ATPase TssH [Salmonella enterica subsp. enterica serovar

Infantis] Overall Protective Antigen Prediction = **0.4269** (Probable **ANTIGEN**).

>EHO9889067.1 type VI secretion system contractile sheath small subunit [Salmonella enterica subsp. enterica serovar Infantis] Overall Protective Antigen Prediction = **0.4798** (Probable **ANTIGEN**).

>EHO9889068.1 type VI secretion system contractile sheath large subunit [Salmonella enterica subsp. enterica serovar Infantis] Overall Protective Antigen Prediction = **0.5047** (Probable **ANTIGEN**).

>EHO9889069.1 BPSL0067 family protein [Salmonella enterica subsp. enterica serovar Infantis] Overall Protective Antigen Prediction = **0.3838** (Probable **NON-ANTIGEN**).

>EHO9889070.1 hypothetical protein KND05_004541 [Salmonella enterica subsp. enterica serovar Infantis] Overall Protective Antigen Prediction = **0.5370** (Probable **ANTIGEN**).

>EHO9889071.1 type VI secretion system tube protein Hcp, partial [Salmonella enterica subsp. enterica serovar Infantis] Overall Protective Antigen Prediction = **0.8947** (Probable **ANTIGEN**).

>EHO9889072.1 hypothetical protein KND05_004544 [Salmonella enterica subsp. enterica serovar Infantis] Overall Protective Antigen Prediction = **0.1220** (Probable **NON-ANTIGEN**).

>EHO9889073.1 excisionase [Salmonella enterica subsp. enterica serovar Infantis] Overall Protective Antigen Prediction = **0.5033** (Probable **ANTIGEN**).

>EHO9889074.1 tyrosine-type recombinase/integrase [Salmonella enterica subsp. enterica serovar Infantis] Overall Protective Antigen Prediction = **0.5341** (Probable **ANTIGEN**).

>EHO9889075.1 putative acyl-CoA thioester hydrolase [Salmonella enterica subsp. enterica serovar Infantis] Overall Protective Antigen Prediction = **0.5976** (Probable **ANTIGEN**).

>EHO9889076.1 6-phosphogluconolactonase [Salmonella enterica subsp. enterica serovar Infantis] Overall Protective Antigen Prediction = **0.5720** (Probable **ANTIGEN**).

>EHO9889077.1 pyridoxal phosphatase [Salmonella enterica subsp. enterica serovar Infantis] Overall Protective Antigen Prediction = **0.3619** (Probable **NON-ANTIGEN**).

>EHO9889078.1 molybdenum ABC transporter ATP-binding protein ModC [Salmonella enterica subsp. enterica serovar Infantis] Overall Protective Antigen Prediction = **0.3739** (Probable **NON-ANTIGEN**).

>EHO9889079.1 molybdate ABC transporter permease subunit [Salmonella enterica subsp. enterica serovar Infantis] Overall Protective Antigen Prediction = **0.6333** (Probable **ANTIGEN**).

>EHO9889080.1 molybdate ABC transporter substrate-binding protein [Salmonella enterica subsp. enterica serovar Infantis] Overall Protective Antigen Prediction = **0.3636** (Probable **NON-ANTIGEN**).

>EHO9889081.1 AcrZ family multidrug efflux pump-associated protein [Salmonella enterica subsp. enterica serovar Infantis] Overall Protective Antigen Prediction = **0.4264** (Probable **ANTIGEN**).

>EHO9889082.1 molybdenum-dependent transcriptional regulator [Salmonella enterica subsp. enterica serovar Infantis] Overall Protective Antigen Prediction = **0.4536** (Probable **ANTIGEN**).

>EHO9889083.1 molybdate ABC transporter ATP-binding protein ModF [Salmonella enterica subsp.

enterica serovar Infantis] Overall Protective Antigen Prediction = **0.3682** (Probable **NON-ANTIGEN**).

>EHO9889084.1 DUF2167 domain-containing protein [Salmonella enterica subsp. enterica serovar Infantis] Overall Protective Antigen Prediction = **0.3431** (Probable **NON-ANTIGEN**).

>EHO9889085.1 UDP-glucose 4-epimerase GalE [Salmonella enterica subsp. enterica serovar Infantis] Overall Protective Antigen Prediction = **0.4494** (Probable **ANTIGEN**).

>EHO9889086.1 galactose-1-phosphate uridylyltransferase [Salmonella enterica subsp. enterica serovar Infantis] Overall Protective Antigen Prediction = **0.3989** (Probable **NON-ANTIGEN**).

>EHO9889087.1 galactokinase [Salmonella enterica subsp. enterica serovar Infantis] Overall Protective Antigen Prediction = **0.5523** (Probable **ANTIGEN**).

>EHO9889088.1 galactose-1-epimerase [Salmonella enterica subsp. enterica serovar Infantis] Overall Protective Antigen Prediction = **0.5404** (Probable **ANTIGEN**).

>EHO9889089.1 2,3-diphosphoglycerate-dependent phosphoglycerate mutase [Salmonella enterica subsp. enterica serovar Infantis] Overall Protective Antigen Prediction = **0.3585** (Probable **NON-ANTIGEN**).

>EHO9889090.1 ABC transporter ATP-binding protein [Salmonella enterica subsp. enterica serovar Infantis] Overall Protective Antigen Prediction = **0.4169** (Probable **ANTIGEN**).

>EHO9889091.1 iron ABC transporter permease [Salmonella enterica subsp. enterica serovar Infantis] Overall Protective Antigen Prediction = **0.4013** (Probable **ANTIGEN**).

>EHO9889092.1 triphosphoribosyl-dephospho-CoA synthase [Salmonella enterica subsp. enterica serovar Infantis] Overall Protective Antigen Prediction = **0.3936** (Probable **NON-ANTIGEN**).

>EHO9889093.1 sodium ion-translocating decarboxylase subunit beta, partial [Salmonella enterica subsp. enterica serovar Infantis] Overall Protective Antigen Prediction = **0.4902** (Probable **ANTIGEN**).

>EHO9889094.1 maltose/maltodextrin ABC transporter ATP-binding protein MalK [Salmonella enterica subsp. enterica serovar Infantis] Overall Protective Antigen Prediction = **0.4816** (Probable **ANTIGEN**).

>EHO9889095.1 maltoporin [Salmonella enterica subsp. enterica serovar Infantis] Overall Protective Antigen Prediction = **0.7406** (Probable **ANTIGEN**).

>EHO9889096.1 maltose operon protein MalM [Salmonella enterica subsp. enterica serovar Infantis] Overall Protective Antigen Prediction = **0.4405** (Probable **ANTIGEN**).

>EHO9889097.1 chorismate lyase [Salmonella enterica subsp. enterica serovar Infantis] Overall Protective Antigen Prediction = **0.2071** (Probable **NON-ANTIGEN**).

>EHO9889098.1 4-hydroxybenzoate octaprenyltransferase [Salmonella enterica subsp. enterica serovar Infantis] Overall Protective Antigen Prediction = **0.5116** (Probable **ANTIGEN**).

>EHO9889099.1 glycerol-3-phosphate 1-O-acyltransferase PlsB [Salmonella enterica subsp. enterica serovar Infantis] Overall Protective Antigen Prediction = **0.3877** (Probable **NON-ANTIGEN**).

>EHO9889100.1 diacylglycerol kinase [Salmonella enterica subsp. enterica serovar Infantis] Overall Protective Antigen Prediction = **0.3535** (Probable **NON-ANTIGEN**).

>EHO9889101.1 repressor LexA [Salmonella enterica subsp. enterica serovar Infantis] Overall Protective Antigen Prediction = **0.3803** (Probable **NON-ANTIGEN**).

>EHO9889102.1 MATE family efflux transporter DinF [Salmonella enterica subsp. enterica serovar Infantis] Overall Protective Antigen Prediction = **0.5309** (Probable **ANTIGEN**).

>EHO9889103.1 hypothetical protein KND05_004575 [Salmonella enterica subsp. enterica serovar Infantis] Overall Protective Antigen Prediction = **0.6256** (Probable **ANTIGEN**).

>EHO9889104.1 CsbD family protein [Salmonella enterica subsp. enterica serovar Infantis] Overall Protective Antigen Prediction = **0.7512** (Probable **ANTIGEN**).

>EHO9889105.1 zinc uptake transcriptional repressor Zur [Salmonella enterica subsp. enterica serovar Infantis] Overall Protective Antigen Prediction = **0.4486** (Probable **ANTIGEN**).

>EHO9889106.1 conjugal transfer protein TraF [Salmonella enterica subsp. enterica serovar Infantis] Overall Protective Antigen Prediction = **0.6386** (Probable **ANTIGEN**).

>EHO9889107.1 tRNA dihydrouridine(20/20a) synthase DusA [Salmonella enterica subsp. enterica serovar Infantis] Overall Protective Antigen Prediction = **0.3617** (Probable **NON-ANTIGEN**).

>EHO9889108.1 envelope stress response protein PspG [Salmonella enterica subsp. enterica serovar Infantis] Overall Protective Antigen Prediction = **0.6924** (Probable **ANTIGEN**).

>EHO9889109.1 quinone oxidoreductase [Salmonella enterica subsp. enterica serovar Infantis] Overall Protective Antigen Prediction = **0.5218** (Probable **ANTIGEN**).

>EHO9889110.1 replicative DNA helicase [Salmonella enterica subsp. enterica serovar Infantis] Overall Protective Antigen Prediction = **0.4032** (Probable **ANTIGEN**).

>EHO9889111.1 alanine racemase [Salmonella enterica subsp. enterica serovar Infantis] Overall Protective Antigen Prediction = **0.3351** (Probable **NON-ANTIGEN**).

>EHO9889112.1 colanic acid biosynthesis phosphomannomutase CpsG, partial [Salmonella enterica subsp. enterica serovar Infantis] Overall Protective Antigen Prediction = **0.5627** (Probable **ANTIGEN**).

>EHO9889113.1 undecaprenyl-phosphate glucose phosphotransferase [Salmonella enterica subsp. enterica serovar Infantis] Overall Protective Antigen Prediction = **0.3667** (Probable **NON-ANTIGEN**).

>EHO9889114.1 colanic acid undecaprenyl disphosphate flippase WzxC [Salmonella enterica subsp. enterica serovar Infantis] Overall Protective Antigen Prediction = **0.4142** (Probable **ANTIGEN**).

>EHO9889115.1 colanic acid biosynthesis pyruvyl transferase WcaK [Salmonella enterica subsp. enterica serovar Infantis] Overall Protective Antigen Prediction = **0.3916** (Probable **NON-ANTIGEN**).

>EHO9889116.1 colanic acid biosynthesis glycosyltransferase WcaL [Salmonella enterica subsp. enterica serovar Infantis] Overall Protective Antigen Prediction = **0.3490** (Probable **NON-ANTIGEN**).

>EHO9889117.1 colanic acid biosynthesis protein WcaM [Salmonella enterica subsp. enterica serovar Infantis] Overall Protective Antigen Prediction = **0.6799** (Probable **ANTIGEN**).

>EHO9889118.1 GalU regulator GalF [Salmonella enterica subsp. enterica serovar Infantis] Overall Protective Antigen Prediction = **0.2782** (Probable **NON-ANTIGEN**).

>EHO9889119.1 EpsG family protein [Salmonella enterica subsp. enterica serovar Infantis] Overall Protective Antigen Prediction = **0.6325** (Probable **ANTIGEN**).

>EHO9889120.1 glycosyltransferase family 1 protein [Salmonella enterica subsp. enterica serovar Infantis] Overall Protective Antigen Prediction = **0.3144** (Probable **NON-ANTIGEN**).

>EHO9889121.1 glycosyltransferase [Salmonella enterica subsp. enterica serovar Infantis] Overall Protective Antigen Prediction = **0.3691** (Probable **NON-ANTIGEN**).

>EHO9889122.1 glycosyltransferase [Salmonella enterica subsp. enterica serovar Infantis] Overall Protective Antigen Prediction = **0.2912** (Probable **NON-ANTIGEN**).

>EHO9889123.1 mannose-1-phosphate guanylyltransferase/mannose-6-phosphate isomerase [Salmonella enterica subsp. enterica serovar Infantis] Overall Protective Antigen Prediction = **0.2970** (Probable **NON-ANTIGEN**).

>EHO9889124.1 colanic acid biosynthesis phosphomannomutase CpsG, partial [Salmonella enterica subsp. enterica serovar Infantis] Overall Protective Antigen Prediction = **0.5391** (Probable **ANTIGEN**).

>EHO9889125.1 acetylornithine deacetylase [Salmonella enterica subsp. enterica serovar Infantis] Overall Protective Antigen Prediction = **0.4490** (Probable **ANTIGEN**).

>EHO9889126.1 N-acetyl-gamma-glutamyl-phosphate reductase [Salmonella enterica subsp. enterica serovar Infantis] Overall Protective Antigen Prediction = **0.3968** (Probable **NON-ANTIGEN**).

>EHO9889127.1 acetylglutamate kinase [Salmonella enterica subsp. enterica serovar Infantis] Overall Protective Antigen Prediction = **0.3417** (Probable **NON-ANTIGEN**).

>EHO9889128.1 argininosuccinate lyase [Salmonella enterica subsp. enterica serovar Infantis] Overall Protective Antigen Prediction = **0.3525** (Probable **NON-ANTIGEN**).

>EHO9889129.1 DNA-binding transcriptional regulator OxyR [Salmonella enterica subsp. enterica serovar Infantis] Overall Protective Antigen Prediction = **0.3991** (Probable **NON-ANTIGEN**).

>EHO9889130.1 Si-specific NAD(P)(+) transhydrogenase [Salmonella enterica subsp. enterica serovar Infantis] Overall Protective Antigen Prediction = **0.3905** (Probable **NON-ANTIGEN**).

>EHO9889131.1 HTH-type transcriptional repressor FabR [Salmonella enterica subsp. enterica serovar Infantis] Overall Protective Antigen Prediction = **0.3411** (Probable **NON-ANTIGEN**).

>EHO9889132.1 YijD family membrane protein [Salmonella enterica subsp. enterica serovar Infantis] Overall Protective Antigen Prediction = **0.6653** (Probable **ANTIGEN**).

>EHO9889133.1 tRNA (uridine(54)-C5)-methyltransferase TrmA [Salmonella enterica subsp. enterica serovar Infantis] Overall Protective Antigen Prediction = **0.4523** (Probable **ANTIGEN**).

>EHO9889134.1 TonB-dependent vitamin B12 receptor BtuB [Salmonella enterica subsp. enterica serovar Infantis] Overall Protective Antigen Prediction = **0.4708** (Probable **ANTIGEN**).

>EHO9889135.1 glutamate racemase [Salmonella enterica subsp. enterica serovar Infantis] Overall Protective Antigen Prediction = **0.2599** (Probable **NON-ANTIGEN**).

>EHO9889136.1 cellulose biosynthesis protein BcsG [Salmonella enterica subsp. enterica serovar Infantis] Overall Protective Antigen Prediction = **0.5281** (Probable **ANTIGEN**).

>EHO9889137.1 cellulose biosynthesis protein BcsF [Salmonella enterica subsp. enterica serovar Infantis] Overall Protective Antigen Prediction = **0.3445** (Probable **NON-ANTIGEN**).

>EHO9889138.1 cellulose biosynthesis protein BcsE [Salmonella enterica subsp. enterica serovar Infantis] Overall Protective Antigen Prediction = **0.3250** (Probable **NON-ANTIGEN**).

>EHO9889139.1 YhjR family protein [Salmonella enterica subsp. enterica serovar Infantis] Overall Protective Antigen Prediction = **0.4586** (Probable **ANTIGEN**).

>EHO9889140.1 cellulose biosynthesis protein BcsQ [Salmonella enterica subsp. enterica serovar Infantis] Overall Protective Antigen Prediction = **0.4331** (Probable **ANTIGEN**).

>EHO9889141.1 UDP-forming cellulose synthase catalytic subunit [Salmonella enterica subsp. enterica serovar Infantis] Overall Protective Antigen Prediction = **0.4827** (Probable **ANTIGEN**).

>EHO9889142.1 cellulose biosynthesis cyclic di-GMP-binding regulatory protein BcsB [Salmonella enterica subsp. enterica serovar Infantis] Overall Protective Antigen Prediction = **0.4642** (Probable **ANTIGEN**).

>EHO9889143.1 cellulase [Salmonella enterica subsp. enterica serovar Infantis] Overall Protective Antigen Prediction = **0.4100** (Probable **ANTIGEN**).

>EHO9889144.1 cellulose biosynthesis protein BcsC, partial [Salmonella enterica subsp. enterica serovar Infantis] Overall Protective Antigen Prediction = **0.3723** (Probable **NON-ANTIGEN**).

>EHO9889145.1 arginine/agmatine antiporter [Salmonella enterica subsp. enterica serovar Infantis] Overall Protective Antigen Prediction = **0.4927** (Probable **ANTIGEN**).

>EHO9889146.1 dicarboxylate transporter/tellurite-resistance protein TehA [Salmonella enterica subsp. enterica serovar Infantis] Overall Protective Antigen Prediction = **0.4650** (Probable **ANTIGEN**).

>EHO9889147.1 arginine decarboxylase [Salmonella enterica subsp. enterica serovar Infantis] Overall Protective Antigen Prediction = **0.3346** (Probable **NON-ANTIGEN**).

>EHO9889148.1 helix-turn-helix transcriptional regulator [Salmonella enterica subsp. enterica serovar Infantis] Overall Protective Antigen Prediction = **0.4863** (Probable **ANTIGEN**).

>EHO9889149.1 3'-5' exonuclease, partial [Salmonella enterica subsp. enterica serovar Infantis] Overall Protective Antigen Prediction = **0.6188** (Probable **ANTIGEN**).

>EHO9889150.1 NirD/YgiW/YdeI family stress tolerance protein [Salmonella enterica subsp. enterica serovar Infantis] Overall Protective Antigen Prediction = **0.5438** (Probable **ANTIGEN**).

>EHO9889151.1 MFS transporter [Salmonella enterica subsp. enterica serovar Infantis] Overall

Protective Antigen Prediction = **0.5604** (Probable **ANTIGEN**).

>EHO9889152.1 hypothetical protein KND05_004629 [Salmonella enterica subsp. enterica serovar Infantis] Overall Protective Antigen Prediction = **0.2998** (Probable **NON-ANTIGEN**).

>EHO9889153.1 aminoglycoside N-acetyltransferase AAC(3)-IVa [Salmonella enterica subsp. enterica serovar Infantis] Overall Protective Antigen Prediction = **0.3293** (Probable **NON-ANTIGEN**).

>EHO9889154.1 aminoglycoside O-phosphotransferase APH(4)-Ia [Salmonella enterica subsp. enterica serovar Infantis] Overall Protective Antigen Prediction = **0.4714** (Probable **ANTIGEN**).

>EHO9889155.1 IS6 family transposase [Salmonella enterica subsp. enterica serovar Infantis] Overall Protective Antigen Prediction = **0.3630** (Probable **NON-ANTIGEN**).

>EHO9889156.1 IS91-like element ISVsa3 family transposase, partial [Salmonella enterica subsp. enterica serovar Infantis] Overall Protective Antigen Prediction = **0.5435** (Probable **ANTIGEN**).

>EHO9889157.1 hypothetical protein KND05_004637 [Salmonella enterica subsp. enterica serovar Infantis] Overall Protective Antigen Prediction = **0.7828** (Probable **ANTIGEN**).

>EHO9889158.1 hypothetical protein KND05_004638 [Salmonella enterica subsp. enterica serovar Infantis] Overall Protective Antigen Prediction = **0.5451** (Probable **ANTIGEN**).

>EHO9889159.1 antitermination protein [Salmonella enterica subsp. enterica serovar Infantis] Overall Protective Antigen Prediction = **0.5405** (Probable **ANTIGEN**).

>EHO9889160.1 fumarate hydratase FumD [Salmonella enterica subsp. enterica serovar Infantis] Overall Protective Antigen Prediction = **0.3568** (Probable **NON-ANTIGEN**).

>EHO9889161.1 ParA family protein [Salmonella enterica subsp. enterica serovar Infantis] Overall Protective Antigen Prediction = **0.2961** (Probable **NON-ANTIGEN**).

>EHO9889162.1 helix-turn-helix transcriptional regulator [Salmonella enterica subsp. enterica serovar Infantis] Overall Protective Antigen Prediction = **0.3586** (Probable **NON-ANTIGEN**).

>EHO9889163.1 helix-turn-helix domain-containing protein [Salmonella enterica subsp. enterica serovar Infantis] Overall Protective Antigen Prediction = **0.1373** (Probable **NON-ANTIGEN**).

>EHO9889164.1 hypothetical protein KND05_004644 [Salmonella enterica subsp. enterica serovar Infantis] Overall Protective Antigen Prediction = **0.4161** (Probable **ANTIGEN**).

>EHO9889165.1 hypothetical protein KND05_004645 [Salmonella enterica subsp. enterica serovar Infantis] Overall Protective Antigen Prediction = **0.2042** (Probable **NON-ANTIGEN**).

>EHO9889166.1 replication protein, partial [Salmonella enterica subsp. enterica serovar Infantis] Overall Protective Antigen Prediction = **0.6565** (Probable **ANTIGEN**).

>EHO9889167.1 hypothetical protein KND05_004647 [Salmonella enterica subsp. enterica serovar Infantis] Overall Protective Antigen Prediction = **0.5555** (Probable **ANTIGEN**).

>EHO9889168.1 pentapeptide repeat-containing protein [Salmonella enterica subsp. enterica serovar Infantis] Overall Protective Antigen Prediction = **1.2287** (Probable **ANTIGEN**).

>EHO9889169.1 HNH endonuclease [Salmonella enterica subsp. enterica serovar Infantis] Overall Protective Antigen Prediction = **0.6944** (Probable **ANTIGEN**).

>EHO9889170.1 HNH endonuclease [Salmonella enterica subsp. enterica serovar Infantis] Overall Protective Antigen Prediction = **0.9361** (Probable **ANTIGEN**).

>EHO9889171.1 helix-turn-helix domain-containing protein [Salmonella enterica subsp. enterica serovar Infantis] Overall Protective Antigen Prediction = **0.5133** (Probable **ANTIGEN**).

>EHO9889172.1 transcriptional regulator [Salmonella enterica subsp. enterica serovar Infantis] Overall Protective Antigen Prediction = **0.2959** (Probable **NON-ANTIGEN**).

>EHO9889173.1 hypothetical protein KND05_004653 [Salmonella enterica subsp. enterica serovar Infantis] Overall Protective Antigen Prediction = **0.5010** (Probable **ANTIGEN**).

>EHO9889174.1 DUF2740 family protein [Salmonella enterica subsp. enterica serovar Infantis] Overall Protective Antigen Prediction = **0.0034** (Probable **NON-ANTIGEN**).

>EHO9889175.1 replication protein, partial [Salmonella enterica subsp. enterica serovar Infantis] Overall Protective Antigen Prediction = **0.5622** (Probable **ANTIGEN**).

>EHO9889176.1 hypothetical protein KND05_004656 [Salmonella enterica subsp. enterica serovar Infantis] Overall Protective Antigen Prediction = **0.5179** (Probable **ANTIGEN**).

>EHO9889177.1 hypothetical protein KND05_004658 [Salmonella enterica subsp. enterica serovar Infantis] Overall Protective Antigen Prediction = **0.5048** (Probable **ANTIGEN**).

>EHO9889178.1 UDP-N-acetylmuramate dehydrogenase [Salmonella enterica subsp. enterica serovar Infantis] Overall Protective Antigen Prediction = **0.4471** (Probable **ANTIGEN**).

>EHO9889179.1 bifunctional biotin--[acetyl-CoA-carboxylase] ligase/biotin operon repressor BirA [Salmonella enterica subsp. enterica serovar Infantis] Overall Protective Antigen Prediction = **0.4375** (Probable **ANTIGEN**).

>EHO9889180.1 type I pantothenate kinase [Salmonella enterica subsp. enterica serovar Infantis] Overall Protective Antigen Prediction = **0.1875** (Probable **NON-ANTIGEN**).

>EHO9889181.1 hypothetical protein KND05_004662 [Salmonella enterica subsp. enterica serovar Infantis] Overall Protective Antigen Prediction = **0.1243** (Probable **NON-ANTIGEN**).

>EHO9889182.1 elongation factor Tu, partial [Salmonella enterica subsp. enterica serovar Infantis] Overall Protective Antigen Prediction = **0.4007** (Probable **ANTIGEN**).

>EHO9889183.1 replication protein, partial [Salmonella enterica subsp. enterica serovar Infantis] Overall Protective Antigen Prediction = **0.2174** (Probable **NON-ANTIGEN**).

>EHO9889184.1 AAA family ATPase [Salmonella enterica subsp. enterica serovar Infantis] Overall Protective Antigen Prediction = **0.4184** (Probable **ANTIGEN**).

>EHO9889185.1 DUF4752 family protein [Salmonella enterica subsp. enterica serovar Infantis] Overall Protective Antigen Prediction = **0.3043** (Probable **NON-ANTIGEN**).

>EHO9889186.1 hypothetical protein KND05_004671 [Salmonella enterica subsp. enterica serovar Infantis] Overall Protective Antigen Prediction = **0.5449** (Probable **ANTIGEN**).

>EHO9889187.1 hypothetical protein KND05_004672 [Salmonella enterica subsp. enterica serovar Infantis] Overall Protective Antigen Prediction = **0.3934** (Probable **NON-ANTIGEN**).

>EHO9889188.1 YbcN family protein [Salmonella enterica subsp. enterica serovar Infantis] Overall Protective Antigen Prediction = **0.3652** (Probable **NON-ANTIGEN**).

>EHO9889189.1 NinE family protein [Salmonella enterica subsp. enterica serovar Infantis] Overall Protective Antigen Prediction = **0.6767** (Probable **ANTIGEN**).

>EHO9889190.1 hypothetical protein KND05_004675 [Salmonella enterica subsp. enterica serovar Infantis] Overall Protective Antigen Prediction = **0.7112** (Probable **ANTIGEN**).

>EHO9889191.1 protein ninF [Salmonella enterica subsp. enterica serovar Infantis] Overall Protective Antigen Prediction = **0.5945** (Probable **ANTIGEN**).

>EHO9889192.1 penicillin binding protein PBP4B [Salmonella enterica subsp. enterica serovar Infantis] Overall Protective Antigen Prediction = **0.4048** (Probable **ANTIGEN**).

>EHO9889193.1 GDP-mannose pyrophosphatase NudK [Salmonella enterica subsp. enterica serovar Infantis] Overall Protective Antigen Prediction = **0.3974** (Probable **NON-ANTIGEN**).

>EHO9889194.1 DUF1176 domain-containing protein [Salmonella enterica subsp. enterica serovar Infantis] Overall Protective Antigen Prediction = **0.4594** (Probable **ANTIGEN**).

>EHO9889195.1 hypothetical protein KND05_004680 [Salmonella enterica subsp. enterica serovar Infantis] Overall Protective Antigen Prediction = **0.5034** (Probable **ANTIGEN**).

>EHO9889196.1 autotransporter outer membrane beta-barrel domain-containing protein, partial [Salmonella enterica subsp. enterica serovar Infantis] Overall Protective Antigen Prediction = **0.8838** (Probable **ANTIGEN**).

>EHO9889197.1 EAL domain-containing protein [Salmonella enterica subsp. enterica serovar Infantis] Overall Protective Antigen Prediction = **0.3198** (Probable **NON-ANTIGEN**).

>EHO9889198.1 hypothetical protein KND05_004683 [Salmonella enterica subsp. enterica serovar Infantis] Overall Protective Antigen Prediction = **0.7549** (Probable **ANTIGEN**).

>EHO9889199.1 hypothetical protein KND05_004684 [Salmonella enterica subsp. enterica serovar Infantis] Overall Protective Antigen Prediction = **0.2478** (Probable **NON-ANTIGEN**).

>EHO9889200.1 antitermination protein [Salmonella enterica subsp. enterica serovar Infantis] Overall Protective Antigen Prediction = **0.5136** (Probable **ANTIGEN**).

>EHO9889201.1 phage holin family protein [Salmonella enterica subsp. enterica serovar Infantis] Overall Protective Antigen Prediction = **0.5080** (Probable **ANTIGEN**).

>EHO9889202.1 lysozyme [Salmonella enterica subsp. enterica serovar Infantis] Overall Protective Antigen Prediction = **0.4302** (Probable **ANTIGEN**).

>EHO9889203.1 lysis protein [Salmonella enterica subsp. enterica serovar Infantis] Overall Protective Antigen Prediction = **0.9048** (Probable **ANTIGEN**).

>EHO9889204.1 protease FtsH-inhibitory lysogeny factor CIII [Salmonella enterica subsp. enterica

serovar Infantis] Overall Protective Antigen Prediction = **0.0482** (Probable **NON-ANTIGEN**).

>EHO9889205.1 DUF5444 family protein [Salmonella enterica subsp. enterica serovar Infantis] Overall Protective Antigen Prediction = **0.3306** (Probable **NON-ANTIGEN**).

>EHO9889206.1 hypothetical protein KND05_004691 [Salmonella enterica subsp. enterica serovar Infantis] Overall Protective Antigen Prediction = **0.3272** (Probable **NON-ANTIGEN**).

>EHO9889207.1 recombinase [Salmonella enterica subsp. enterica serovar Infantis] Overall Protective Antigen Prediction = **0.8463** (Probable **ANTIGEN**).

>EHO9889208.1 sigma-70 family RNA polymerase sigma factor [Salmonella enterica subsp. enterica serovar Infantis] Overall Protective Antigen Prediction = **0.6797** (Probable **ANTIGEN**).

>EHO9889209.1 DUF2856 family protein [Salmonella enterica subsp. enterica serovar Infantis] Overall Protective Antigen Prediction = **0.5655** (Probable **ANTIGEN**).

>EHO9889210.1 DUF2737 family protein [Salmonella enterica subsp. enterica serovar Infantis] Overall Protective Antigen Prediction = **0.2717** (Probable **NON-ANTIGEN**).

>EHO9889211.1 HNH endonuclease, partial [Salmonella enterica subsp. enterica serovar Infantis] Overall Protective Antigen Prediction = **0.6526** (Probable **ANTIGEN**).

>EHO9889212.1 Rpn family recombination-promoting nuclease/putative transposase [Salmonella enterica subsp. enterica serovar Infantis] Overall Protective Antigen Prediction = **0.3742** (Probable **NON-ANTIGEN**).

>EHO9889213.1 YebC/PmpR family DNA-binding transcriptional regulator [Salmonella enterica subsp. enterica serovar Infantis] Overall Protective Antigen Prediction = **0.4949** (Probable **ANTIGEN**).

>EHO9889214.1 heme lyase NrfEFG subunit NrfE, partial [Salmonella enterica subsp. enterica serovar Infantis] Overall Protective Antigen Prediction = **0.6935** (Probable **ANTIGEN**).

>EHO9889215.1 cytochrome c maturation protein CcmE [Salmonella enterica subsp. enterica serovar Infantis] Overall Protective Antigen Prediction = **0.5479** (Probable **ANTIGEN**).

>EHO9889216.1 heme exporter protein CcmD [Salmonella enterica subsp. enterica serovar Infantis] Overall Protective Antigen Prediction = **0.5758** (Probable **ANTIGEN**).

>EHO9889217.1 heme ABC transporter permease [Salmonella enterica subsp. enterica serovar Infantis] Overall Protective Antigen Prediction = **0.3681** (Probable **NON-ANTIGEN**).

>EHO9889218.1 tail fiber protein [Salmonella enterica subsp. enterica serovar Infantis] Overall Protective Antigen Prediction = **0.2244** (Probable **NON-ANTIGEN**).

>EHO9889219.1 tail fiber assembly protein [Salmonella enterica subsp. enterica serovar Infantis] Overall Protective Antigen Prediction = **0.4512** (Probable **ANTIGEN**).

>EHO9889220.1 tail fiber assembly protein [Salmonella enterica subsp. enterica serovar Infantis] Overall Protective Antigen Prediction = **0.4231** (Probable **ANTIGEN**).

>EHO9889221.1 type VI secretion system amidase effector protein Tae4 [Salmonella enterica subsp. enterica serovar Infantis] Overall Protective Antigen Prediction = **0.2128** (Probable **NON-ANTIGEN**

).

>EHO9889222.1 type VI secretion system amidase immunity protein Tai4 [Salmonella enterica subsp. enterica serovar Infantis] Overall Protective Antigen Prediction = **0.4581** (Probable **ANTIGEN**).

>EHO9889223.1 type VI secretion system tube protein Hcp [Salmonella enterica subsp. enterica serovar Infantis] Overall Protective Antigen Prediction = **1.0482** (Probable **ANTIGEN**).

>EHO9889224.1 type VI secretion system lipoprotein TssJ, partial [Salmonella enterica subsp. enterica serovar Infantis] Overall Protective Antigen Prediction = **0.5966** (Probable **ANTIGEN**).

>EHO9889225.1 biotin/lipoyl-binding protein, partial [Salmonella enterica subsp. enterica serovar Infantis] Overall Protective Antigen Prediction = **0.5440** (Probable **ANTIGEN**).

>EHO9889226.1 sodium ion-translocating decarboxylase subunit beta, partial [Salmonella enterica subsp. enterica serovar Infantis] Overall Protective Antigen Prediction = **0.3858** (Probable **NON-ANTIGEN**).

>EHO9889227.1 IS3-like element ISSen1 family transposase, partial [Salmonella enterica subsp. enterica serovar Infantis] Overall Protective Antigen Prediction = **0.3666** (Probable **NON-ANTIGEN**).

>EHO9889228.1 IS630 family transposase, partial [Salmonella enterica subsp. enterica serovar Infantis] Overall Protective Antigen Prediction = **0.4179** (Probable **ANTIGEN**).

>EHO9889229.1 biotin/lipoyl-binding protein, partial [Salmonella enterica subsp. enterica serovar Infantis] Overall Protective Antigen Prediction = **0.7023** (Probable **ANTIGEN**).

>EHO9889230.1 sodium ion-translocating decarboxylase subunit beta, partial [Salmonella enterica subsp. enterica serovar Infantis] Overall Protective Antigen Prediction = **0.4005** (Probable **ANTIGEN**).

>EHO9889231.1 glycosyltransferase family 2 protein, partial [Salmonella enterica subsp. enterica serovar Infantis] Overall Protective Antigen Prediction = **0.4631** (Probable **ANTIGEN**).

>EHO9889232.1 heme exporter protein CcmB, partial [Salmonella enterica subsp. enterica serovar Infantis] Overall Protective Antigen Prediction = **0.8025** (Probable **ANTIGEN**).

>EHO9889233.1 elongation factor Tu, partial [Salmonella enterica subsp. enterica serovar Infantis] Overall Protective Antigen Prediction = **0.4020** (Probable **ANTIGEN**).

>EHO9889234.1 IS6-like element IS26 family transposase, partial [Salmonella enterica subsp. enterica serovar Infantis] Overall Protective Antigen Prediction = **0.6682** (Probable **ANTIGEN**).

>EHO9889235.1 oxaloacetate decarboxylase, partial [Salmonella enterica subsp. enterica serovar Infantis] Overall Protective Antigen Prediction = **0.4752** (Probable **ANTIGEN**).

>EHO9889236.1 tetratricopeptide repeat protein, partial [Salmonella enterica subsp. enterica serovar Infantis] Overall Protective Antigen Prediction = **0.5950** (Probable **ANTIGEN**).

>EHO9889237.1 IS200/IS605-like element IS200F family transposase, partial [Salmonella enterica subsp. enterica serovar Infantis] Overall Protective Antigen Prediction = **0.3566** (Probable **NON-ANTIGEN**).

>EHO9889238.1 transposase, partial [Salmonella enterica subsp. enterica serovar Infantis] Overall Protective Antigen Prediction = **0.1081** (Probable **NON-ANTIGEN**).

>EHO9889239.1 transposase, partial [Salmonella enterica subsp. enterica serovar Infantis] Overall Protective Antigen Prediction = **0.0730** (Probable **NON-ANTIGEN**).

>EHO9889240.1 IS110 family transposase, partial [Salmonella enterica subsp. enterica serovar Infantis] Overall Protective Antigen Prediction = **0.1634** (Probable **NON-ANTIGEN**).

>EHO9889241.1 IS110 family transposase, partial [Salmonella enterica subsp. enterica serovar Infantis] Overall Protective Antigen Prediction = **0.7526** (Probable **ANTIGEN**).

>EHO9889242.1 autotransporter-associated beta strand repeat-containing protein, partial [Salmonella enterica subsp. enterica serovar Infantis] Overall Protective Antigen Prediction = **0.9712** (Probable **ANTIGEN**).

>EHO9889243.1 bifunctional phosphopantothenoylcysteine decarboxylase/phosphopantothenate--cysteine ligase CoaBC, partial [Salmonella enterica subsp. enterica serovar Infantis] Overall Protective Antigen Prediction = **0.4896** (Probable **ANTIGEN**).

>EHO9889244.1 dUTP diphosphatase [Salmonella enterica subsp. enterica serovar Infantis] Overall Protective Antigen Prediction = **0.7433** (Probable **ANTIGEN**).

>EHO9889245.1 nucleoid occlusion factor SlmA [Salmonella enterica subsp. enterica serovar Infantis] Overall Protective Antigen Prediction = **0.5588** (Probable **ANTIGEN**).

>EHO9889246.1 orotate phosphoribosyltransferase [Salmonella enterica subsp. enterica serovar Infantis] Overall Protective Antigen Prediction = **0.2727** (Probable **NON-ANTIGEN**).

>EHO9889247.1 ribonuclease PH [Salmonella enterica subsp. enterica serovar Infantis] Overall Protective Antigen Prediction = **0.5559** (Probable **ANTIGEN**).

>EHO9889248.1 YicC family protein [Salmonella enterica subsp. enterica serovar Infantis] Overall Protective Antigen Prediction = **0.5609** (Probable **ANTIGEN**).

>EHO9889249.1 LysR family transcriptional regulator [Salmonella enterica subsp. enterica serovar Infantis] Overall Protective Antigen Prediction = **0.4448** (Probable **ANTIGEN**).

>EHO9889250.1 HARLDQ motif MBL-fold protein [Salmonella enterica subsp. enterica serovar Infantis] Overall Protective Antigen Prediction = **0.3265** (Probable **NON-ANTIGEN**).

>EHO9889251.1 trimeric intracellular cation channel family protein [Salmonella enterica subsp. enterica serovar Infantis] Overall Protective Antigen Prediction = **0.4391** (Probable **ANTIGEN**).

>EHO9889252.1 NAD-dependent DNA ligase LigB [Salmonella enterica subsp. enterica serovar Infantis] Overall Protective Antigen Prediction = **0.3964** (Probable **NON-ANTIGEN**).

>EHO9889253.1 guanylate kinase [Salmonella enterica subsp. enterica serovar Infantis] Overall Protective Antigen Prediction = **0.4600** (Probable **ANTIGEN**).

>EHO9889254.1 DNA-directed RNA polymerase subunit omega [Salmonella enterica subsp. enterica serovar Infantis] Overall Protective Antigen Prediction = **0.5834** (Probable **ANTIGEN**).

>EHO9889255.1 bifunctional GTP diphosphokinase/guanosine-3',5'-bis pyrophosphate 3'-

pyrophosphohydrolase [Salmonella enterica subsp. enterica serovar Infantis] Overall Protective Antigen Prediction = **0.4628** (Probable **ANTIGEN**).

>EHO9889256.1 tRNA (guanosine(18)-2'-O)-methyltransferase TrmH [Salmonella enterica subsp. enterica serovar Infantis] Overall Protective Antigen Prediction = **0.3484** (Probable **NON-ANTIGEN**).

>EHO9889257.1 ATP-dependent DNA helicase RecG [Salmonella enterica subsp. enterica serovar Infantis] Overall Protective Antigen Prediction = **0.4144** (Probable **ANTIGEN**).

>EHO9889258.1 sodium/glutamate symporter, partial [Salmonella enterica subsp. enterica serovar Infantis] Overall Protective Antigen Prediction = **0.7999** (Probable **ANTIGEN**).

>EHO9889259.1 permease, partial [Salmonella enterica subsp. enterica serovar Infantis] Overall Protective Antigen Prediction = **-0.0233** (Probable **NON-ANTIGEN**).

>EHO9889260.1 BtpA family protein SgcQ [Salmonella enterica subsp. enterica serovar Infantis] Overall Protective Antigen Prediction = **0.2536** (Probable **NON-ANTIGEN**).

>EHO9889261.1 PTS sugar transporter subunit IIA [Salmonella enterica subsp. enterica serovar Infantis] Overall Protective Antigen Prediction = **0.3992** (Probable **NON-ANTIGEN**).

>EHO9889262.1 ribulose-phosphate 3 epimerase family protein [Salmonella enterica subsp. enterica serovar Infantis] Overall Protective Antigen Prediction = **0.5272** (Probable **ANTIGEN**).

>EHO9889263.1 DeoR/GlpR transcriptional regulator [Salmonella enterica subsp. enterica serovar Infantis] Overall Protective Antigen Prediction = **0.2799** (Probable **NON-ANTIGEN**).

>EHO9889264.1 cryptic aminoglycoside N-acetyltransferase AAC(6')-Iy/Iaa [Salmonella enterica subsp. enterica serovar Infantis] Overall Protective Antigen Prediction = **0.3014** (Probable **NON-ANTIGEN**).

>EHO9889265.1 alpha-hydroxy-acid oxidizing protein [Salmonella enterica subsp. enterica serovar Infantis] Overall Protective Antigen Prediction = **0.5037** (Probable **ANTIGEN**).

>EHO9889266.1 polyisoprenoid-binding protein [Salmonella enterica subsp. enterica serovar Infantis] Overall Protective Antigen Prediction = **0.4712** (Probable **ANTIGEN**).

>EHO9889267.1 glucan biosynthesis protein [Salmonella enterica subsp. enterica serovar Infantis] Overall Protective Antigen Prediction = **0.4723** (Probable **ANTIGEN**).

>EHO9889268.1 carboxylesterase/lipase family protein [Salmonella enterica subsp. enterica serovar Infantis] Overall Protective Antigen Prediction = **0.3725** (Probable **NON-ANTIGEN**).

>EHO9889269.1 VOC family protein [Salmonella enterica subsp. enterica serovar Infantis] Overall Protective Antigen Prediction = **0.3680** (Probable **NON-ANTIGEN**).

>EHO9889270.1 LysR family transcriptional regulator [Salmonella enterica subsp. enterica serovar Infantis] Overall Protective Antigen Prediction = **0.3606** (Probable **NON-ANTIGEN**).

>EHO9889271.1 Tar ligand binding domain-containing protein [Salmonella enterica subsp. enterica serovar Infantis] Overall Protective Antigen Prediction = **0.5874** (Probable **ANTIGEN**).

>EHO9889272.1 S-(hydroxymethyl)glutathione dehydrogenase/class III alcohol dehydrogenase

[Salmonella enterica subsp. enterica serovar Infantis] Overall Protective Antigen Prediction = **0.5300** (Probable **ANTIGEN**).

>EHO9889273.1 metal/formaldehyde-sensitive transcriptional repressor [Salmonella enterica subsp. enterica serovar Infantis] Overall Protective Antigen Prediction = **0.3602** (Probable **NON-ANTIGEN**).

>EHO9889274.1 effector protein, partial [Salmonella enterica subsp. enterica serovar Infantis] Overall Protective Antigen Prediction = **0.8798** (Probable **ANTIGEN**).

>EHO9889275.1 transposase, partial [Salmonella enterica subsp. enterica serovar Infantis] Overall Protective Antigen Prediction = **0.2709** (Probable **NON-ANTIGEN**).

>EHO9889276.1 EAL domain-containing protein [Salmonella enterica subsp. enterica serovar Infantis] Overall Protective Antigen Prediction = **0.4951** (Probable **ANTIGEN**).

>EHO9889277.1 broad-spectrum mercury transporter MerE [Salmonella enterica subsp. enterica serovar Infantis] Overall Protective Antigen Prediction = **0.1650** (Probable **NON-ANTIGEN**).

>EHO9889278.1 mercury resistance co-regulator MerD [Salmonella enterica subsp. enterica serovar Infantis] Overall Protective Antigen Prediction = **0.3857** (Probable **NON-ANTIGEN**).

>EHO9889279.1 mercury(II) reductase [Salmonella enterica subsp. enterica serovar Infantis] Overall Protective Antigen Prediction = **0.5596** (Probable **ANTIGEN**).

>EHO9889280.1 organomercurial transporter MerC [Salmonella enterica subsp. enterica serovar Infantis] Overall Protective Antigen Prediction = **0.2045** (Probable **NON-ANTIGEN**).

>EHO9889281.1 mercury resistance system periplasmic binding protein MerP [Salmonella enterica subsp. enterica serovar Infantis] Overall Protective Antigen Prediction = **0.3522** (Probable **NON-ANTIGEN**).

>EHO9889282.1 mercuric transport protein MerT [Salmonella enterica subsp. enterica serovar Infantis] Overall Protective Antigen Prediction = **0.4464** (Probable **ANTIGEN**).

>EHO9889283.1 Hg(II)-responsive transcriptional regulator [Salmonella enterica subsp. enterica serovar Infantis] Overall Protective Antigen Prediction = **0.3528** (Probable **NON-ANTIGEN**).

>EHO9889284.1 relaxase [Salmonella enterica subsp. enterica serovar Infantis] Overall Protective Antigen Prediction = **0.8136** (Probable **ANTIGEN**).

>EHO9889285.1 tetracycline resistance transcriptional repressor TetR(A) [Salmonella enterica subsp. enterica serovar Infantis] Overall Protective Antigen Prediction = **0.5897** (Probable **ANTIGEN**).

>EHO9889286.1 tetracycline efflux MFS transporter Tet(A) [Salmonella enterica subsp. enterica serovar Infantis] Overall Protective Antigen Prediction = **0.4535** (Probable **ANTIGEN**).

>EHO9889287.1 EamA family transporter [Salmonella enterica subsp. enterica serovar Infantis] Overall Protective Antigen Prediction = **0.5316** (Probable **ANTIGEN**).

>EHO9889288.1 cysteine hydrolase [Salmonella enterica subsp. enterica serovar Infantis] Overall Protective Antigen Prediction = **0.4888** (Probable **ANTIGEN**).

>EHO9889289.1 class 1 integron integrase IntI1 [Salmonella enterica subsp. enterica serovar Infantis]

Overall Protective Antigen Prediction = **0.4675** (Probable **ANTIGEN**).

>EHO9889290.1 ANT(3")-Ia family aminoglycoside nucleotidyltransferase AadA1 [Salmonella enterica subsp. enterica serovar Infantis] Overall Protective Antigen Prediction = **0.2619** (Probable **NON-ANTIGEN**).

>EHO9889291.1 quaternary ammonium compound efflux SMR transporter QacE delta 1 [Salmonella enterica subsp. enterica serovar Infantis] Overall Protective Antigen Prediction = **0.3843** (Probable **NON-ANTIGEN**).

>EHO9889292.1 sulfonamide-resistant dihydropteroate synthase Sul1 [Salmonella enterica subsp. enterica serovar Infantis] Overall Protective Antigen Prediction = **0.3201** (Probable **NON-ANTIGEN**).

>EHO9889293.1 GNAT family N-acetyltransferase [Salmonella enterica subsp. enterica serovar Infantis] Overall Protective Antigen Prediction = **0.6325** (Probable **ANTIGEN**).

>EHO9889294.1 IS21-like element IS1326 family helper ATPase IstB [Salmonella enterica subsp. enterica serovar Infantis] Overall Protective Antigen Prediction = **0.4151** (Probable **ANTIGEN**).

>EHO9889295.1 IS21-like element IS1326 family transposase [Salmonella enterica subsp. enterica serovar Infantis] Overall Protective Antigen Prediction = **0.4533** (Probable **ANTIGEN**).

>EHO9889296.1 TniB family NTP-binding protein [Salmonella enterica subsp. enterica serovar Infantis] Overall Protective Antigen Prediction = **0.3411** (Probable **NON-ANTIGEN**).

>EHO9889297.1 transposase, partial [Salmonella enterica subsp. enterica serovar Infantis] Overall Protective Antigen Prediction = **0.3794** (Probable **NON-ANTIGEN**).

>EHO9889298.1 DNA polymerase IV, partial [Salmonella enterica subsp. enterica serovar Infantis] Overall Protective Antigen Prediction = **0.4129** (Probable **ANTIGEN**).

>EHO9889299.1 RNA ligase RtcB family protein [Salmonella enterica subsp. enterica serovar Infantis] Overall Protective Antigen Prediction = **0.2530** (Probable **NON-ANTIGEN**).

>EHO9889300.1 peptide chain release factor H [Salmonella enterica subsp. enterica serovar Infantis] Overall Protective Antigen Prediction = **0.6578** (Probable **ANTIGEN**).

>EHO9889301.1 cytosol nonspecific dipeptidase [Salmonella enterica subsp. enterica serovar Infantis] Overall Protective Antigen Prediction = **0.4784** (Probable **ANTIGEN**).

>EHO9889302.1 xanthine phosphoribosyltransferase [Salmonella enterica subsp. enterica serovar Infantis] Overall Protective Antigen Prediction = **0.4003** (Probable **ANTIGEN**).

>EHO9889303.1 esterase FrsA [Salmonella enterica subsp. enterica serovar Infantis] Overall Protective Antigen Prediction = **0.4758** (Probable **ANTIGEN**).

>EHO9889304.1 Crl family RNA polymerase assembly factor, partial [Salmonella enterica subsp. enterica serovar Infantis] Overall Protective Antigen Prediction = **0.0566** (Probable **NON-ANTIGEN**).

>EHO9889305.1 aminoglycoside N-acetyltransferase AAC(3)-IVa [Salmonella enterica subsp. enterica serovar Infantis] Overall Protective Antigen Prediction = **0.3293** (Probable **NON-ANTIGEN**).

>EHO9889306.1 aminoglycoside O-phosphotransferase APH(4)-Ia [Salmonella enterica subsp. enterica serovar Infantis] Overall Protective Antigen Prediction = **0.4714** (Probable **ANTIGEN**).

>EHO9889307.1 IS6 family transposase [Salmonella enterica subsp. enterica serovar Infantis] Overall Protective Antigen Prediction = **0.3630** (Probable **NON-ANTIGEN**).

>EHO9889308.1 IS91 family transposase, partial [Salmonella enterica subsp. enterica serovar Infantis] Overall Protective Antigen Prediction = **0.5287** (Probable **ANTIGEN**).
