# Supplementary material for: In silico design of a multiepitope subunit vaccine targeting Salmonella enterica serovar Infantis: an immunoinformatics and reverse vaccinology approach
Source: Front Immunol. 2026 Feb 6;17:1717278. doi: 10.3389/fimmu.2026.1717278 (PMC12920593; doi:10.3389/fimmu.2026.1717278)
Supplement: Supplementary File 6 — Antigenicity prediction results by Vaxijen 2.0 Tool, for selected CTL, HTL, and B-cell epitopes. [file DataSheet6.pdf]

Supplementary File 9 shows antigenicity prediction results by Vaxijen 2.0 Tool, for selected CTL, HTL and B-cell epitopes

CTL Epitope Results

VaxiJen RESULTS

Model selected: bacteria

Threshold for this model: 0.4

Your Sequence:

> seq 1

VAAFAAIVV

Overall Prediction for the Protective Antigen = **0.2582** (Probable **NON-ANTIGEN**).

Your Sequence:

> seq 2

KVAAFAAIV

Overall Prediction for the Protective Antigen = **0.1090** (Probable **NON-ANTIGEN**).

Your Sequence:

> seq 3

AAFAAIVVS

Overall Prediction for the Protective Antigen = **0.2200** (Probable **NON-ANTIGEN**).

Your Sequence:

> seq 4

VPQWGGGGN

Overall Prediction for the Protective Antigen = **2.0683** (Probable **ANTIGEN**).

Your Sequence:

> seq 5

LKVAAFAAI

Overall Prediction for the Protective Antigen = **0.6080** (Probable **ANTIGEN**).

Your Sequence:

> seq 6

RKSETTITQ

Overall Prediction for the Protective Antigen = **1.1735** (Probable **ANTIGEN**).

Your Sequence:

> seq 7

NATIDQWNA

Overall Prediction for the Protective Antigen = **0.4996** (Probable **ANTIGEN**).

Your Sequence:

> seq 8

NNATIDQWN

Overall Prediction for the Protective Antigen = **0.4871** (Probable **ANTIGEN**).

Your Sequence:

> seq 9

DNSTIELTQ

Overall Prediction for the Protective Antigen = **0.6103** (Probable **ANTIGEN**).

Your Sequence:

> seq 10

KSETTITQS

Overall Prediction for the Protective Antigen = **0.9452** (Probable **ANTIGEN**).

Your Sequence:

> seq 11

NSTIELTQN

Overall Prediction for the Protective Antigen = **0.4079** (Probable **ANTIGEN**).

Your Sequence:

> seq 12

PQWGGGGNH

Overall Prediction for the Protective Antigen = **2.2734** (Probable **ANTIGEN**).

Your Sequence:

> seq 13

FRNNATIDQ

Overall Prediction for the Protective Antigen = **0.3683** (Probable **NON-ANTIGEN**).

Your Sequence:

> seq 14

QNGFRNNAT

Overall Prediction for the Protective Antigen = **0.6154** (Probable **ANTIGEN**).

Your Sequence:

> seq 15

KNSDITVGQ

Overall Prediction for the Protective Antigen = **1.5501** (Probable **ANTIGEN**).

Your Sequence:

> seq 16

TQNGFRNNA

Overall Prediction for the Protective Antigen = **0.6604** (Probable **ANTIGEN**).

Your Sequence:

> seq 17

RQVGFGNNA

Overall Prediction for the Protective Antigen = **0.0136** (Probable **NON-ANTIGEN**).

Your Sequence:

> seq 18

NSDITVGQY

Overall Prediction for the Protective Antigen = **1.1410** (Probable **ANTIGEN**).

Your Sequence:

> seq 19

ATIDQWNAK

Overall Prediction for the Protective Antigen = **0.9673** (Probable **ANTIGEN**).

Your Sequence:

> seq 20

VVPQWGGGG

Overall Prediction for the Protective Antigen = **1.6447** (Probable **ANTIGEN**).

Your Sequence:

> seq 21

QVGFGNNAT

Overall Prediction for the Protective Antigen = **0.5152** (Probable **ANTIGEN**).

Your Sequence:

> seq 22

VRQVGFGNN

Overall Prediction for the Protective Antigen = **-0.0324** (Probable **NON-ANTIGEN**).

Your Sequence:

> seq 23

LLKVAAFAA

Overall Prediction for the Protective Antigen = **0.0667** (Probable **NON-ANTIGEN**).

Your Sequence:

> seq 24

AFAAIVVSG

Overall Prediction for the Protective Antigen = **0.1231** (Probable **NON-ANTIGEN**).

Your Sequence:

> seq 25

RNNATIDQW

Overall Prediction for the Protective Antigen = **0.7152** (Probable **ANTIGEN**).

Your Sequence:

> seq 26

GFRNNATID

Overall Prediction for the Protective Antigen = **0.7068** (Probable **ANTIGEN**).

Your Sequence:

> seq 27

GVVPQWGGG

Overall Prediction for the Protective Antigen = **0.8672** (Probable **ANTIGEN**).

Your Sequence:

> seq 28

YGNGADVGQ

Overall Prediction for the Protective Antigen = **1.7583** (Probable **ANTIGEN**).

Your Sequence:

> seq 29

AGVVPQWGG

Overall Prediction for the Protective Antigen = **0.2341** (Probable **NON-ANTIGEN**).

Your Sequence:

> seq 30

LTQNGFRNN

Overall Prediction for the Protective Antigen = **0.6202** (Probable **ANTIGEN**).

Your Sequence:

> seq 31

SALAGVVPQ

Overall Prediction for the Protective Antigen = **0.5381** (Probable **ANTIGEN**).

Your Sequence:

> seq 32

NGFRNNATI

Overall Prediction for the Protective Antigen = **0.2795** (Probable **NON-ANTIGEN**).

Your Sequence:

> seq 33

GSALAGVVP

Overall Prediction for the Protective Antigen = **0.7254** (Probable **ANTIGEN**).

Your Sequence:

> seq 34

QWGGGGNHN

Overall Prediction for the Protective Antigen = **2.0099** (Probable **ANTIGEN**).

Your Sequence:

> seq 35

GYGNGADVG

Overall Prediction for the Protective Antigen = **2.2555** (Probable **ANTIGEN**).

Your Sequence:

> seq 36

VGFGNNATA

Overall Prediction for the Protective Antigen = **0.3902** (Probable **NON-ANTIGEN**).

Your Sequence:

> seq 37

LAGVVPQWG

Overall Prediction for the Protective Antigen = **0.6361** (Probable **ANTIGEN**).

Your Sequence:

> seq 38

FAAIVVSGS

Overall Prediction for the Protective Antigen = **0.1257** (Probable **NON-ANTIGEN**).

Your Sequence:

> seq 39

SGYGNGADV

Overall Prediction for the Protective Antigen = **2.5833** (Probable **ANTIGEN**).

Your Sequence:

> seq 40

GGNHNGGGN

Overall Prediction for the Protective Antigen = **4.0471** (Probable **ANTIGEN**).

Your Sequence:

> seq 41

GNNAALVNQ

Overall Prediction for the Protective Antigen = **0.6181** (Probable **ANTIGEN**).

Your Sequence:

> seq 42

WGGGGNHNG

Overall Prediction for the Protective Antigen = **2.1254** (Probable **ANTIGEN**).

Your Sequence:

> seq 43

FGNNATANQ

Overall Prediction for the Protective Antigen = **0.8082** (Probable **ANTIGEN**).

Your Sequence:

> seq 44

GSANAALAL

Overall Prediction for the Protective Antigen = **0.9693** (Probable **ANTIGEN**).

Your Sequence:

> seq 45

GNHNGGGNS

Overall Prediction for the Protective Antigen = **3.7121** (Probable **ANTIGEN**).

Your Sequence:

> seq 46

SANAALALQ

Overall Prediction for the Protective Antigen = **0.8548** (Probable **ANTIGEN**).

Your Sequence:

> seq 47

MVRQVGFGN

Overall Prediction for the Protective Antigen = **-0.3954** (Probable **NON-ANTIGEN**).

Your Sequence:

> seq 48

GFGNNATAN

Overall Prediction for the Protective Antigen = **1.1198** (Probable **ANTIGEN**).

Your Sequence:

> seq 49

QYGGNNAAL

Overall Prediction for the Protective Antigen = **1.1175** (Probable **ANTIGEN**).

Your Sequence:

> seq 50

VMVRQVGFG

Overall Prediction for the Protective Antigen = **0.1376** (Probable **NON-ANTIGEN**).

Your Sequence:

> seq 51

QSGYGNGAD

Overall Prediction for the Protective Antigen = **2.4991** (Probable **ANTIGEN**).

Your Sequence:

> seq 52

GGGGNHNGG

Overall Prediction for the Protective Antigen = **3.8407** (Probable **ANTIGEN**).

Your Sequence:

> seq 53

GGNNAALVN

Overall Prediction for the Protective Antigen = **1.2358** (Probable **ANTIGEN**).

Your Sequence:

> seq 54

GGGNHNGGG

Overall Prediction for the Protective Antigen = **4.1434** (Probable **ANTIGEN**).

Your Sequence:

> seq 55

GQYGGNNAA

Overall Prediction for the Protective Antigen = **0.8574** (Probable **ANTIGEN**).

Your Sequence:

> seq 56

ADNSTIELT

Overall Prediction for the Protective Antigen = **1.1084** (Probable **ANTIGEN**).

Your Sequence:

> seq 57

GNGADVGQG

Overall Prediction for the Protective Antigen = **2.4223** (Probable **ANTIGEN**).

Your Sequence:

> seq 58

STIELTQNG

Overall Prediction for the Protective Antigen = **0.4365** (Probable **ANTIGEN**).

Your Sequence:

> seq 59

SGSALAGVV

Overall Prediction for the Protective Antigen = **0.6736** (Probable **ANTIGEN**).

Your Sequence:

> seq 60

YGGNNAALV

Overall Prediction for the Protective Antigen = **0.7019** (Probable **ANTIGEN**).

Your Sequence:

> seq 61

SDITVGQYG

Overall Prediction for the Protective Antigen = **0.9778** (Probable **ANTIGEN**).

Your Sequence:

> seq 62

ELTQNGFRN

Overall Prediction for the Protective Antigen = **0.5069** (Probable **ANTIGEN**).

Your Sequence:

> seq 63

ALAGVVPQW

Overall Prediction for the Protective Antigen = **0.9056** (Probable **ANTIGEN**).

Your Sequence:

> seq 64

GADNSTIEL

Overall Prediction for the Protective Antigen = **1.6544** (Probable **ANTIGEN**).

Your Sequence:

> seq 65

YGSANAALA

Overall Prediction for the Protective Antigen = **0.6446** (Probable **ANTIGEN**).

Your Sequence:

> seq 66

GNNATANQY

Overall Prediction for the Protective Antigen = **1.5590** (Probable **ANTIGEN**).

Your Sequence:

> seq 67

AKNSDITVG

Overall Prediction for the Protective Antigen = **1.7127** (Probable **ANTIGEN**).

Your Sequence:

> seq 68

IDQWNAKNS

Overall Prediction for the Protective Antigen = **1.0757** (Probable **ANTIGEN**).

Your Sequence:

> seq 69

NGADVGQGA

Overall Prediction for the Protective Antigen = **1.5519** (Probable **ANTIGEN**).

Your Sequence:

> seq 70

GADVGQGAD

Overall Prediction for the Protective Antigen = **1.7226** (Probable **ANTIGEN**).

Your Sequence:

> seq 71

SETTITQSG

Overall Prediction for the Protective Antigen = **0.8171** (Probable **ANTIGEN**).

Your Sequence:

> seq 72

TQSGYGNGA

Overall Prediction for the Protective Antigen = **2.0593** (Probable **ANTIGEN**).

Your Sequence:

> seq 73

VGQYGGNNA

Overall Prediction for the Protective Antigen = **0.4812** (Probable **ANTIGEN**).

Your Sequence:

> seq 74

IELTQNGFR

Overall Prediction for the Protective Antigen = **0.7568** (Probable **ANTIGEN**).

Your Sequence:

> seq 75

ADVGQGADN

Overall Prediction for the Protective Antigen = **1.1297** (Probable **ANTIGEN**).

Your Sequence:

> seq 76

ARKSETTIT

Overall Prediction for the Protective Antigen = **1.2696** (Probable **ANTIGEN**).

Your Sequence:

> seq 77

TIDQWNAKN

Overall Prediction for the Protective Antigen = **0.7302** (Probable **ANTIGEN**).

Your Sequence:

> seq 78

NNAALVNQT

Overall Prediction for the Protective Antigen = **-0.0089** (Probable **NON-ANTIGEN**).

Your Sequence:

> seq 79

ANAALALQS

Overall Prediction for the Protective Antigen = **0.4420** (Probable **ANTIGEN**).

HTL ; IL-4 Epitope Reults

VaxiJen RESULTS

Model selected: bacteria

Threshold for this model: 0.4

Your Sequence:

> seq 1

LKVAAFAAIVVSGSA

Overall Prediction for the Protective Antigen = **0.5636** (Probable **ANTIGEN**).

Your Sequence:

> seq 2

SIYQYGSANAALALQ

Overall Prediction for the Protective Antigen = **0.5348** (Probable **ANTIGEN**).

Your Sequence:

> seq 3

TLSIYQYGSANAALA

Overall Prediction for the Protective Antigen = **0.5850** (Probable **ANTIGEN**).

Your Sequence:

> seq 5

YQYGSANAALALQSD

Overall Prediction for the Protective Antigen = **0.7353** (Probable **ANTIGEN**).

Your Sequence:

> seq 7

DSTLSIYQYGSANAA

Overall Prediction for the Protective Antigen = **0.5283** (Probable **ANTIGEN**).

Your Sequence:

> seq 8

SDITVGQYGGNNAAL

Overall Prediction for the Protective Antigen = **0.9408** (Probable **ANTIGEN**).

Your Sequence:

> seq 9

WGGGGNHNGGGNSSG

Overall Prediction for the Protective Antigen = **3.1991** (Probable **ANTIGEN**).

Your Sequence:

> seq 10

VRQVGFGNNATANQY

Overall Prediction for the Protective Antigen = **0.5258** (Probable **ANTIGEN**).

Your Sequence:

> seq 11

ETTITQSGYGNGADV

Overall Prediction for the Protective Antigen = **1.6424** (Probable **ANTIGEN**).

Your Sequence:

> seq 12

QWGGGGNHNGGGNSS

Overall Prediction for the Protective Antigen = **3.0075** (Probable **ANTIGEN**).

Your Sequence:

> seq 13

NGADVGQGADNSTIE

Overall Prediction for the Protective Antigen = **1.5630** (Probable **ANTIGEN**).

Your Sequence:

> seq 14

AALALQSDARKSETT

Overall Prediction for the Protective Antigen = **1.2804** (Probable **ANTIGEN**).

Your Sequence:

> seq 15

ALALQSDARKSETTI

Overall Prediction for the Protective Antigen = **1.1651** (Probable **ANTIGEN**).

Your Sequence:

> seq 16

LALQSDARKSETTIT

Overall Prediction for the Protective Antigen = **1.2757** (Probable **ANTIGEN**).

Your Sequence:

> seq 17

GADVGQGADNSTIEL

Overall Prediction for the Protective Antigen = **1.4914** (Probable **ANTIGEN**).

Your Sequence:

> seq 18

NSDITVGQYGGNNAA

Overall Prediction for the Protective Antigen = **1.0406** (Probable **ANTIGEN**).

Your Sequence:

> seq 19

SETTITQSGYGNGAD

Overall Prediction for the Protective Antigen = **1.5416** (Probable **ANTIGEN**).

Your Sequence:

> seq 20

PDSTLSIYQYGSANA

Overall Prediction for the Protective Antigen = **0.5301** (Probable **ANTIGEN**).

Your Sequence:

> seq 21

ADVGQGADNSTIELT

Overall Prediction for the Protective Antigen = **1.3071** (Probable **ANTIGEN**).

Your Sequence:

> seq 22

VVPQWGGGGNHNGGG

Overall Prediction for the Protective Antigen = **2.5530** (Probable **ANTIGEN**).

HTL ; INF Epitope Reults

VaxiJen RESULTS

Model selected: bacteria

Threshold for this model: 0.4

Your Sequence:

> seq 2

LKVAAFAAIVVSGSA

Overall Prediction for the Protective Antigen = **0.5636** (Probable **ANTIGEN**).

Your Sequence:

> seq 3

SIYQYGSANAALALQ

Overall Prediction for the Protective Antigen = **0.5348** (Probable **ANTIGEN**).

Your Sequence:

> seq 7

DSTLSIYQYGSANAA

Overall Prediction for the Protective Antigen = **0.5283** (Probable **ANTIGEN**).

Your Sequence:

> seq 9

TVGQYGGNNAALVNQ

Overall Prediction for the Protective Antigen = **0.5985** (Probable **ANTIGEN**).

Your Sequence:

> seq 10

DITVGQYGGNNAALV

Overall Prediction for the Protective Antigen = **0.8905** (Probable **ANTIGEN**).

Your Sequence:

> seq 11

ITVGQYGGNNAALVN

Overall Prediction for the Protective Antigen = **0.5778** (Probable **ANTIGEN**).

Your Sequence:

> seq 12

GGNHNGGGNSSGPDS

Overall Prediction for the Protective Antigen = **2.9569** (Probable **ANTIGEN**).

Your Sequence:

> seq 13

GGGNHNGGGNSSGPD

Overall Prediction for the Protective Antigen = **3.2976** (Probable **ANTIGEN**).

Your Sequence:

> seq 14

TQSGYGNGADVGQGA

Overall Prediction for the Protective Antigen = **1.9939** (Probable **ANTIGEN**).

Your Sequence:

> seq 15

GGGGNHNGGGNSSGP

Overall Prediction for the Protective Antigen = **3.4904** (Probable **ANTIGEN**).

Your Sequence:

> seq 16

GNHNGGGNSSGPDST

Overall Prediction for the Protective Antigen = **2.7098** (Probable **ANTIGEN**).

Your Sequence:

> seq 17

QSGYGNGADVGQGAD

Overall Prediction for the Protective Antigen = **2.1109** (Probable **ANTIGEN**).

Your Sequence:

> seq 18

ITQSGYGNGADVGQG

Overall Prediction for the Protective Antigen = **1.7130** (Probable **ANTIGEN**).

Your Sequence:

> seq 19

SGYGNGADVGQGADN

Overall Prediction for the Protective Antigen = **1.9006** (Probable **ANTIGEN**).

Your Sequence:

> seq 20

GYGNGADVGQGADNS

Overall Prediction for the Protective Antigen = **1.8032** (Probable **ANTIGEN**).

Your Sequence:

> seq 21

TTITQSGYGNGADVG

Overall Prediction for the Protective Antigen = **1.4626** (Probable **ANTIGEN**).

Your Sequence:

> seq 22

SDITVGQYGGNNAAL

Overall Prediction for the Protective Antigen = **0.9408** (Probable **ANTIGEN**).

Your Sequence:

> seq 23

WGGGGNHNGGGNSSG

Overall Prediction for the Protective Antigen = **3.1991** (Probable **ANTIGEN**).

Your Sequence:

> seq 24

YGNGADVGQGADNST

Overall Prediction for the Protective Antigen = **1.7563** (Probable **ANTIGEN**).

Your Sequence:

> seq 25

GNGADVGQGADNSTI

Overall Prediction for the Protective Antigen = **1.7760** (Probable **ANTIGEN**).

Your Sequence:

> seq 26

QWGGGGNHNGGGNSS

Overall Prediction for the Protective Antigen = **3.0075** (Probable **ANTIGEN**).

Your Sequence:

> seq 27

NGADVGQGADNSTIE

Overall Prediction for the Protective Antigen = **1.5630** (Probable **ANTIGEN**).

Your Sequence:

> seq 28

GSALAGVVPQWGGGG

Overall Prediction for the Protective Antigen = **1.2112** (Probable **ANTIGEN**).

Your Sequence:

> seq 29

ALALQSDARKSETTI

Overall Prediction for the Protective Antigen = **1.1651** (Probable **ANTIGEN**).

Your Sequence:

> seq 30

GADVGQGADNSTIEL

Overall Prediction for the Protective Antigen = **1.4914** (Probable **ANTIGEN**).

Your Sequence:

> seq 31

NSDITVGQYGGNNAA

Overall Prediction for the Protective Antigen = **1.0406** (Probable **ANTIGEN**).

Your Sequence:

> seq 32

PDSTLSIYQYGSANA

Overall Prediction for the Protective Antigen = **0.5301** (Probable **ANTIGEN**).

Your Sequence:

> seq 33

ADVGQGADNSTIELT

Overall Prediction for the Protective Antigen = **1.3071** (Probable **ANTIGEN**).

Your Sequence:

> seq 34

VVPQWGGGGNHNGGG

Overall Prediction for the Protective Antigen = **2.5530** (Probable **ANTIGEN**).

B-cell Epitope Reults

VaxiJen RESULTS

Model selected: bacteria

Threshold for this model: 0.4

Your Sequence:

> seq 1

NNAALVNQTASDSS

Overall Prediction for the Protective Antigen = **0.6840** (Probable **ANTIGEN**).

Your Sequence:

> seq 2

SETTITQSGYGNGADV

Overall Prediction for the Protective Antigen = **1.6164** (Probable **ANTIGEN**).

Your Sequence:

> seq 3

PQWGGGGNHNGGGNSS

Overall Prediction for the Protective Antigen = **2.9955** (Probable **ANTIGEN**).

Your Sequence:

> seq 4

HNGGGNSSGPDSTLSI

Overall Prediction for the Protective Antigen = **2.2504** (Probable **ANTIGEN**).

Your Sequence:

> seq 5

DNSTIELTQNGFRN

Overall Prediction for the Protective Antigen = **0.4934** (Probable **ANTIGEN**).

Your Sequence:

> seq 6

SGPDSTLSIYQYGS

Overall Prediction for the Protective Antigen = **0.7228** (Probable **ANTIGEN**).

Your Sequence:

> seq 7

NATIDQWNAKNSDI

Overall Prediction for the Protective Antigen = **1.1590** (Probable **ANTIGEN**).

Your Sequence:

> seq 8

AKNSDITVGQYG

Overall Prediction for the Protective Antigen = **1.2452** (Probable **ANTIGEN**).

Your Sequence:

> seq 9

DVGQGADNSTIELT

Overall Prediction for the Protective Antigen = **1.3224** (Probable **ANTIGEN**).

Your Sequence:

> seq 10

DSTLSIYQYGSANAAL

Overall Prediction for the Protective Antigen = **0.4855** (Probable **ANTIGEN**).

Your Sequence:

> seq 11

ITQSGYGNGADVGQ

Overall Prediction for the Protective Antigen = **1.5362** (Probable **ANTIGEN**).

Your Sequence:

> seq 12

NATIDQWNAKNSDITV

Overall Prediction for the Protective Antigen = **1.2540** (Probable **ANTIGEN**).

Your Sequence:

> seq 13

VMVRQVGFGNNATANQ

Overall Prediction for the Protective Antigen = **0.5443** (Probable **ANTIGEN**).

Your Sequence:

> seq 14

ADVGQGADNSTIELTQ

Overall Prediction for the Protective Antigen = **1.0983** (Probable **ANTIGEN**).

Your Sequence:

> seq 15

SGYGNGADVGQGADNS

Overall Prediction for the Protective Antigen = **1.8510** (Probable **ANTIGEN**).

Your Sequence:

> seq 16

GGGGNHNGGGNSSG

Overall Prediction for the Protective Antigen = **4.0168** (Probable **ANTIGEN**).

Your Sequence:

> seq 17

VVPQWGGGGNHNGG

Overall Prediction for the Protective Antigen = **2.1460** (Probable **ANTIGEN**).

Your Sequence:

> seq 18

KSETTITQSGYGNG

Overall Prediction for the Protective Antigen = **1.3537** (Probable **ANTIGEN**).

Your Sequence:

> seq 19

DITVGQYGGNNAALVN

Overall Prediction for the Protective Antigen = **0.8790** (Probable **ANTIGEN**).

Your Sequence:

> seq 20

STIELTQNGFRNNATI

Overall Prediction for the Protective Antigen = **0.4795** (Probable **ANTIGEN**).
