# Supplementary material for: In silico design of a multiepitope subunit vaccine targeting Salmonella enterica serovar Infantis: an immunoinformatics and reverse vaccinology approach
Source: Front Immunol. 2026 Feb 6;17:1717278. doi: 10.3389/fimmu.2026.1717278 (PMC12920593; doi:10.3389/fimmu.2026.1717278)
Supplement: Supplementary File 11 — Results of Antigenicity, allergenicity, solubility along with Physico-chemical properties and Blastp analysis of MEV (Multi-epitope Vaccine). [file DataSheet11.docx]

**Supplementary File 11 shows Antigenicity, allergenicity, solubility, Blastp analysis and physicochemical properties of MEV (Multi-epitope Vaccine)**

**MEV SEQUENCE (Multi-epitope vaccine sequence)**

MIKLKFGVFFTVLLSSAYAHGTPQNITDLCAEYHNTQIYTLNDKIFSYTESLAGKREMAIITFKNGAIFQVEVPGSQHIDSQKKAIERMKDTLRIAYLTEAKVEKLCVWNNKTPHAIAAISMANEAAAKGGGNHNGGGAAYGGNHNGGGNAAYGGGGNHNGGAAYGNHNGGGNSAAYSGYGNGADVGPGPGLKVAAFAAIVVSGSAGPGPGSIYQYGSANAALALQGPGPGSTLSIYQYGSANAALGPGPGDSTLSIYQYGSANAAGPGPGSDITVGQYGGNNAALGPGPGWGGGGNHNGGGNSSGGPGPGQWGGGGNHNGGGNSSGPGPGNGADVGQGADNSTIEGPGPGGADVGQGADNSTIELGPGPGNSDITVGQYGGNNAAGPGPGPDSTLSIYQYGSANAGPGPGADVGQGADNSTIELTGPGPGVVPQWGGGGNHNGGGKKGGGGNHNGGGNSSGKKPQWGGGGNHNGGGNSSKKHNGGGNSSGPDSTLSIKKVVPQWGGGGNHNGGKKSGYGNGADVGQGADNS

1. **Vaxijen – Antigenicity prediction results for MEV (Copied from pdf)**

**Model selected: bacteria**

**Threshold for this model: 0.4**

**Your Sequence:**

MIKLKFGVFFTVLLSSAYAHGTPQNITDLCA

EYHNTQIYTLNDKIFSYTESLAGKREMAIIT

FKNGAIFQVEVPGSQHIDSQKKAIERMKDTL

RIAYLTEAKVEKLCVWNNKTPHAIAAISMAN

EAAAKGGGNHNGGGAAYGGNHNGGGNAAYGG

GGNHNGGAAYGNHNGGGNSAAYSGYGNGADV

GPGPGLKVAAFAAIVVSGSAGPGPGSIYQYG

SANAALALQGPGPGSTLSIYQYGSANAALGP

GPGDSTLSIYQYGSANAAGPGPGSDITVGQY

GGNNAALGPGPGWGGGGNHNGGGNSSGGPGP

GQWGGGGNHNGGGNSSGPGPGNGADVGQGAD

NSTIEGPGPGGADVGQGADNSTIELGPGPGN

SDITVGQYGGNNAAGPGPGPDSTLSIYQYGS

ANAGPGPGADVGQGADNSTIELTGPGPGVVP

QWGGGGNHNGGGKKGGGGNHNGGGNSSGKKP

QWGGGGNHNGGGNSSKKHNGGGNSSGPDSTL

SIKKVVPQWGGGGNHNGGKKSGYGNGADVGQ

GADNS

**Overall Prediction for the Protective Antigen = 1.5127 ( Probable ANTIGEN ).**

1. **Antigenpro -- Antigenicity prediction results for MEV**

**Name: MEV**

**Amino Acids:**

MIKLKFGVFFTVLLSSAYAHGTPQNITDLCAEYHNTQIYTLNDKIFSYTESLAGKREMAIITFKNGAIFQVEVPGSQHIDSQKKAIERMKDTLRIAYLTEAKVEKLCVWNNKTPHAIAAISMANEAAAKGGGNHNGGGAAYGGNHNGGGNAAYGGGGNHNGGAAYGNHNGGGNSAAYSGYGNGADVGPGPGLKVAAFAAIVVSGSAGPGPGSIYQYGSANAALALQGPGPGSTLSIYQYGSANAALGPGPGDSTLSIYQYGSANAAGPGPGSDITVGQYGGNNAALGPGPGWGGGGNHNGGGNSSGGPGPGQWGGGGNHNGGGNSSGPGPGNGADVGQGADNSTIEGPGPGGADVGQGADNSTIELGPGPGNSDITVGQYGGNNAAGPGPGPDSTLSIYQYGSANAGPGPGADVGQGADNSTIELTGPGPGVVPQWGGGGNHNGGGKKGGGGNHNGGGNSSGKKPQWGGGGNHNGGGNSSKKHNGGGNSSGPDSTLSIKKVVPQWGGGGNHNGGKKSGYGNGADVGQGADNS

**Predicted Probability of Antigenicity:**

**0.799964**

For an explanation of the output format, please see <http://scratch.proteomics.ics.uci.edu/explanation.html>

1. **AllerTOP v. 2.0 -- Allergenicity prediction result for MEV**

**Bioinformatics tool for allergenicity prediction**

Your sequence is:

**PROBABLE NON-ALLERGEN**

The nearest protein is:

UniProtKB accession number Q60EQ4

defined as non-allergen

1. **ALGPRED -- Allergenicity prediction result for MEV**

| **Name of sequence** MEV  **Length of Sequence** 532  **Predicted On** Wed Aug 31 21:08:55 2022  **NON ALLERGEN**  Prediction by mapping of IgE epitope  The protein sequence does not contain experimentally proven IgE epitope  **MAST RESULT** : No Hits found  **NON ALLERGEN**  **Prediction by SVM method based on amino acid composition**  **NON ALLERGEN** |  |
| --- | --- |

1. **Blast P – Screenshot from NCBI BlastP result (MEV against *Gallus gallus*)**


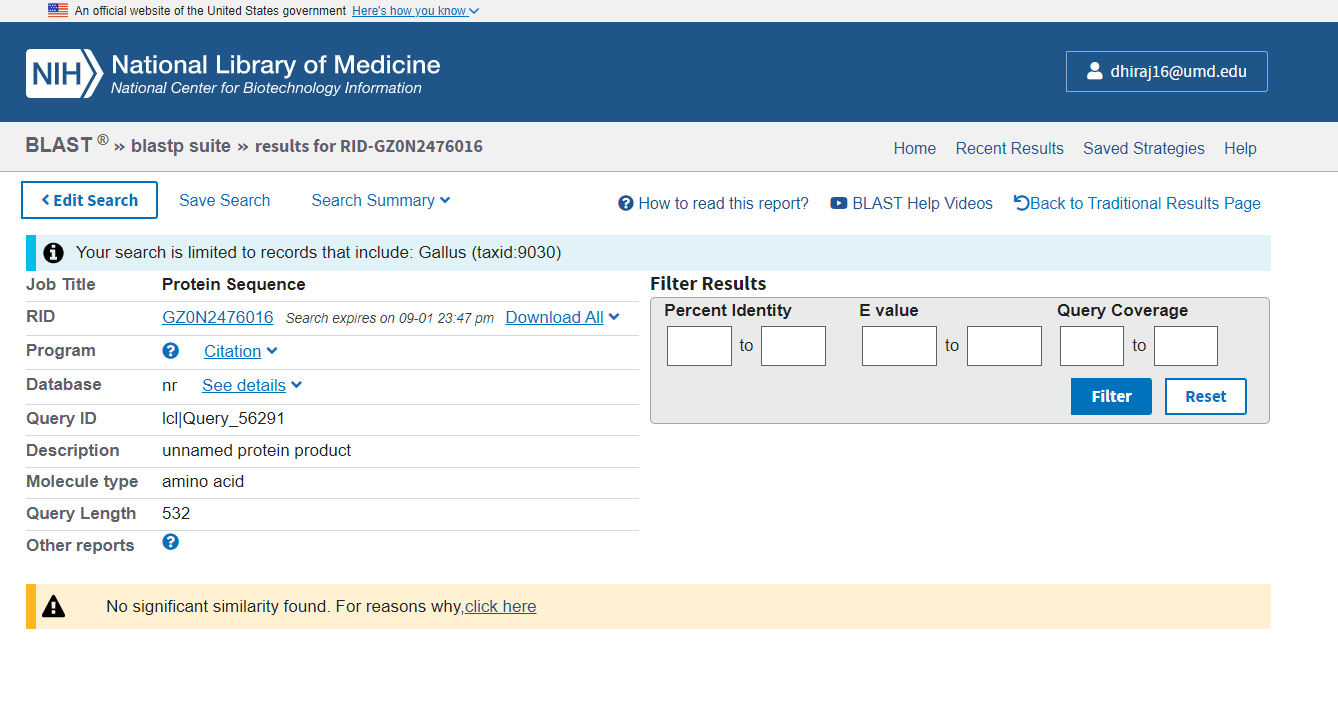


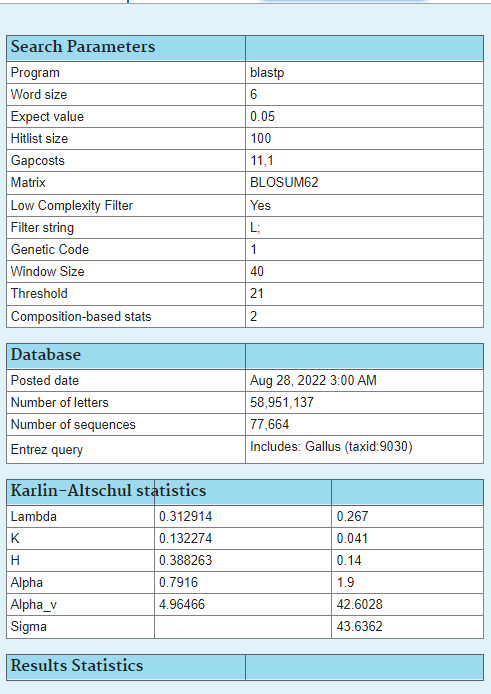


1. **Results from PROTPARAM tool of Expasy server to predict the physico-chemical properties (Copied from pdf for convenience)**

**ProtParam**

**User-provided sequence:**

10 20 30 40 50 60
MIKLKFGVFF TVLLSSAYAH GTPQNITDLC AEYHNTQIYT LNDKIFSYTE SLAGKREMAI

 70 80 90 100 110 120
ITFKNGAIFQ VEVPGSQHID SQKKAIERMK DTLRIAYLTE AKVEKLCVWN NKTPHAIAAI

 130 140 150 160 170 180
SMANEAAAKG GGNHNGGGAA YGGNHNGGGN AAYGGGGNHN GGAAYGNHNG GGNSAAYSGY

 190 200 210 220 230 240
GNGADVGPGP GLKVAAFAAI VVSGSAGPGP GSIYQYGSAN AALALQGPGP GSTLSIYQYG

 250 260 270 280 290 300
SANAALGPGP GDSTLSIYQY GSANAAGPGP GSDITVGQYG GNNAALGPGP GWGGGGNHNG

 310 320 330 340 350 360
GGNSSGGPGP GQWGGGGNHN GGGNSSGPGP GNGADVGQGA DNSTIEGPGP GGADVGQGAD

 370 380 390 400 410 420
NSTIELGPGP GNSDITVGQY GGNNAAGPGP GPDSTLSIYQ YGSANAGPGP GADVGQGADN

 430 440 450 460 470 480
STIELTGPGP GVVPQWGGGG NHNGGGKKGG GGNHNGGGNS SGKKPQWGGG GNHNGGGNSS

 490 500 510 520 530
KKHNGGGNSS GPDSTLSIKK VVPQWGGGGN HNGGKKSGYG NGADVGQGAD NS

[References](https://web.expasy.org/protparam/protpar-ref.html) and [documentation](https://web.expasy.org/protparam/protparam-doc.html) are available.

**Number of amino acids:** 532

**Molecular weight:** 51181.98

**Theoretical pI:** 6.56

Top of Form

**Amino acid composition:** 
Ala (A) 54 10.2%

Arg (R) 3 0.6%

Asn (N) 51 9.6%

Asp (D) 18 3.4%

Cys (C) 2 0.4%

Gln (Q) 20 3.8%

Glu (E) 11 2.1%

Gly (G) 139 26.1%

His (H) 15 2.8%

Ile (I) 23 4.3%

Leu (L) 20 3.8%

Lys (K) 23 4.3%

Met (M) 4 0.8%

Phe (F) 7 1.3%

Pro (P) 34 6.4%

Ser (S) 41 7.7%

Thr (T) 20 3.8%

Trp (W) 6 1.1%

Tyr (Y) 21 3.9%

Val (V) 20 3.8%

Pyl (O) 0 0.0%

Sec (U) 0 0.0%

(B) 0 0.0%

(Z) 0 0.0%

(X) 0 0.0%

Bottom of Form

**Total number of negatively charged residues (Asp + Glu):** 29

**Total number of positively charged residues (Arg + Lys):** 26

**Atomic composition:**

Carbon C 2192

Hydrogen H 3333

Nitrogen N 671

Oxygen O 744

Sulfur S 6

**Formula:** C_2192_H_3333_N_671_O_744_S_6_

**Total number of atoms:** 6946

**Extinction coefficients:**

Extinction coefficients are in units of M^-1^ cm^-1^, at 280 nm measured in water.

Ext. coefficient 64415

Abs 0.1% (=1 g/l) 1.259, assuming all pairs of Cys residues form cystines

Ext. coefficient 64290

Abs 0.1% (=1 g/l) 1.256, assuming all Cys residues are reduced

**Estimated half-life:**

The N-terminal of the sequence considered is M (Met).

The estimated half-life is: 30 hours (mammalian reticulocytes, in vitro).

>20 hours (yeast, in vivo).

>10 hours (Escherichia coli, in vivo).

**Instability index:**

The instability index (II) is computed to be 21.53

This classifies the protein as stable.

**Aliphatic index:** 52.58

**Grand average of hydropathicity (GRAVY):** -0.560
