# Supplementary material for: In silico design of a multiepitope subunit vaccine targeting Salmonella enterica serovar Infantis: an immunoinformatics and reverse vaccinology approach
Source: Front Immunol. 2026 Feb 6;17:1717278. doi: 10.3389/fimmu.2026.1717278 (PMC12920593; doi:10.3389/fimmu.2026.1717278)
Supplement: Supplementary File 13 — Results from ligplot+ representing complete analysis of MEV-immune receptor interaction of each of 4 immune receptors used in the manuscript. [file DataSheet13.docx]

**Supplementary File 13;** contains complete pictures from ligplot+ representation/analysis of MEV-immune receptor interaction for each of 4 immune receptors used in the manuscript.


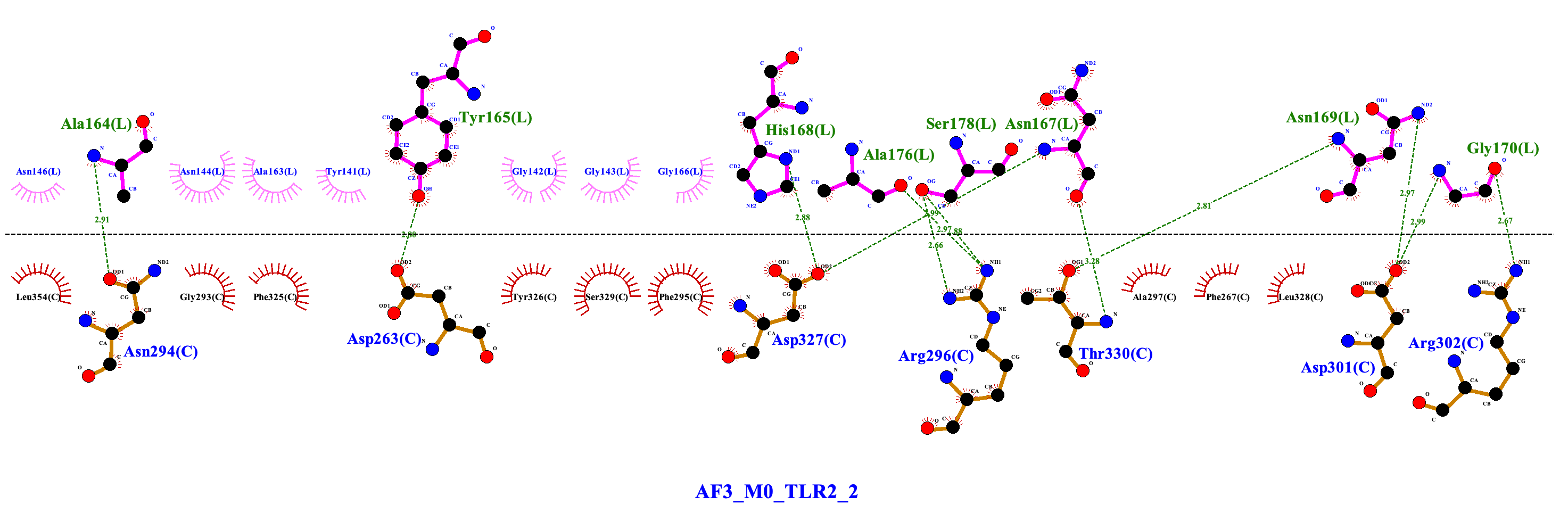


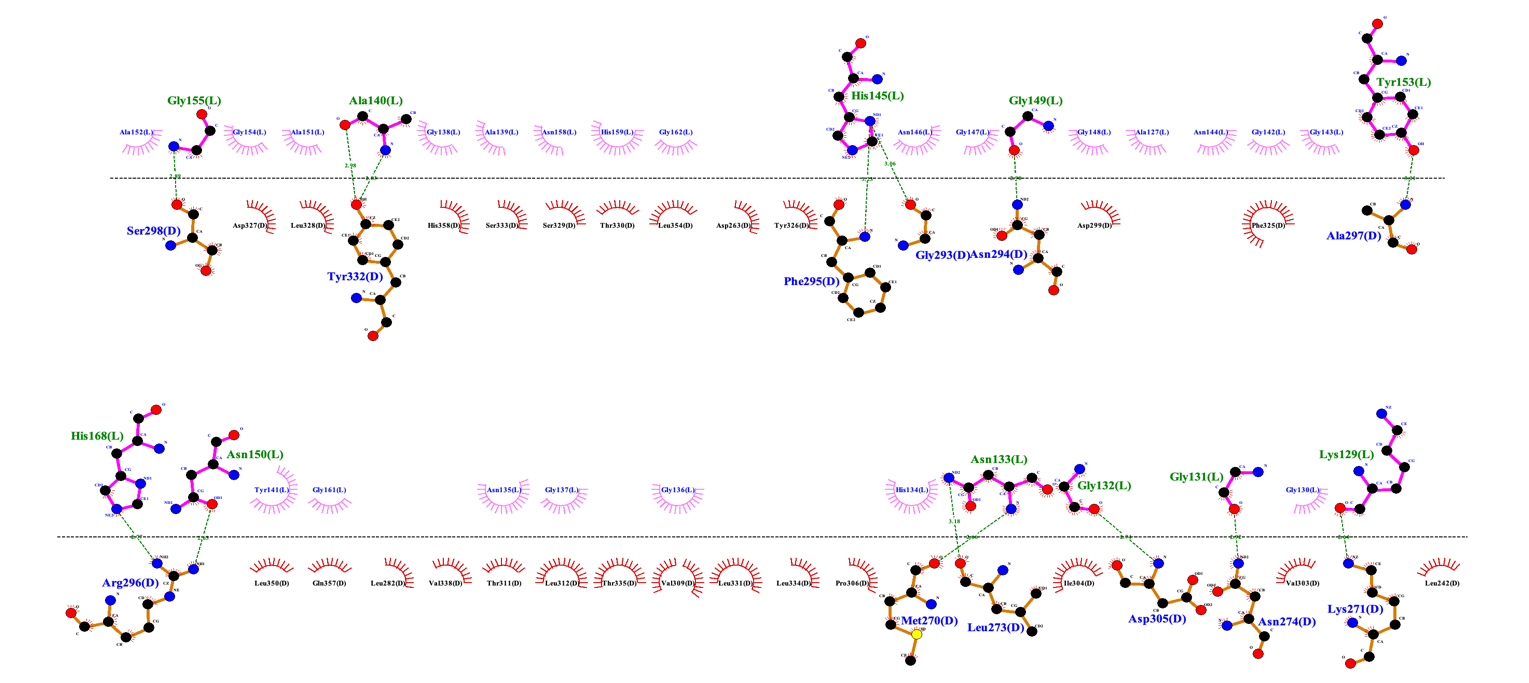


**Figure S1. Ligplot+ representation of interactions between MEV ligand (L) with TLR2 immune receptor.**


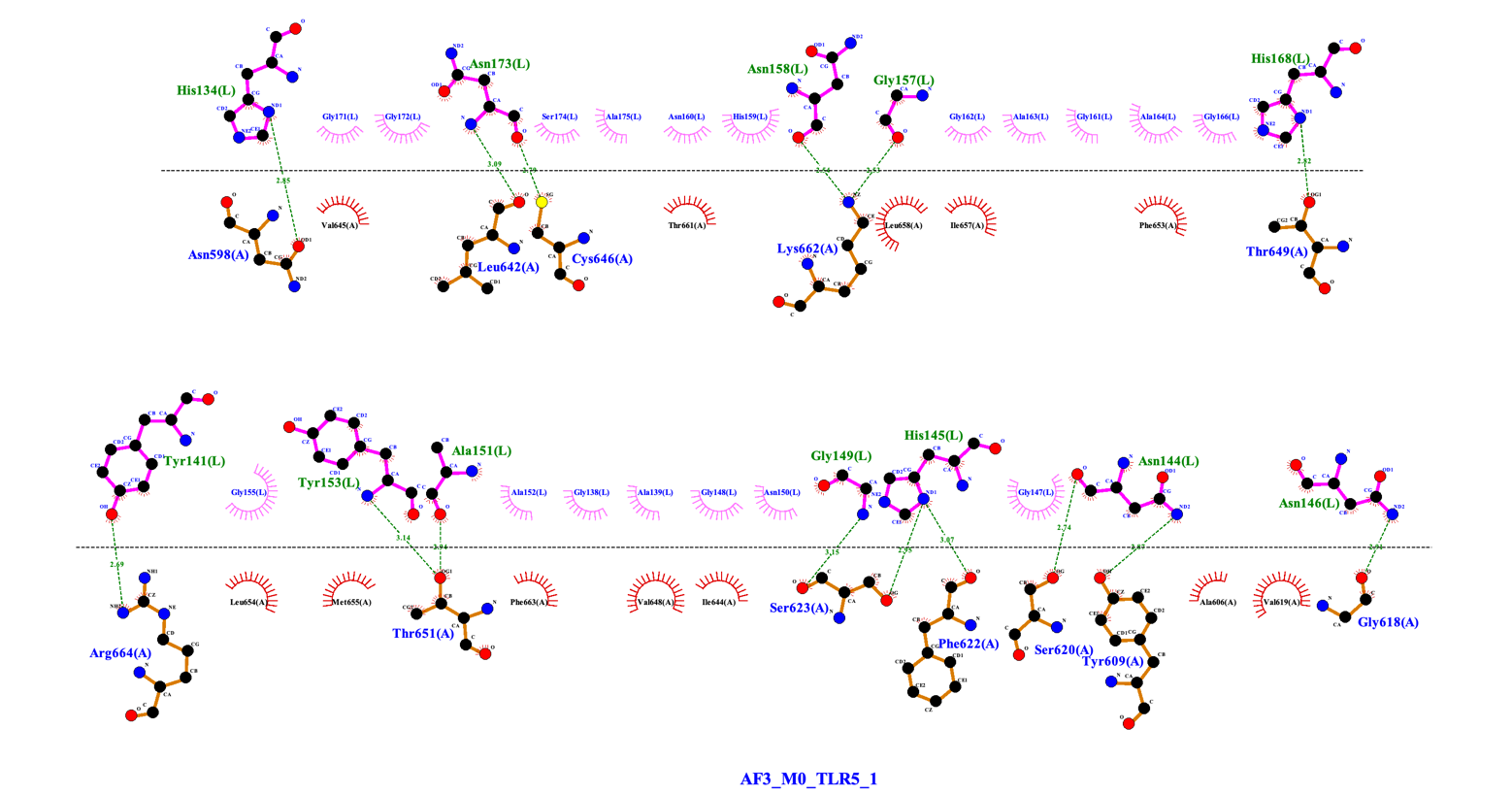


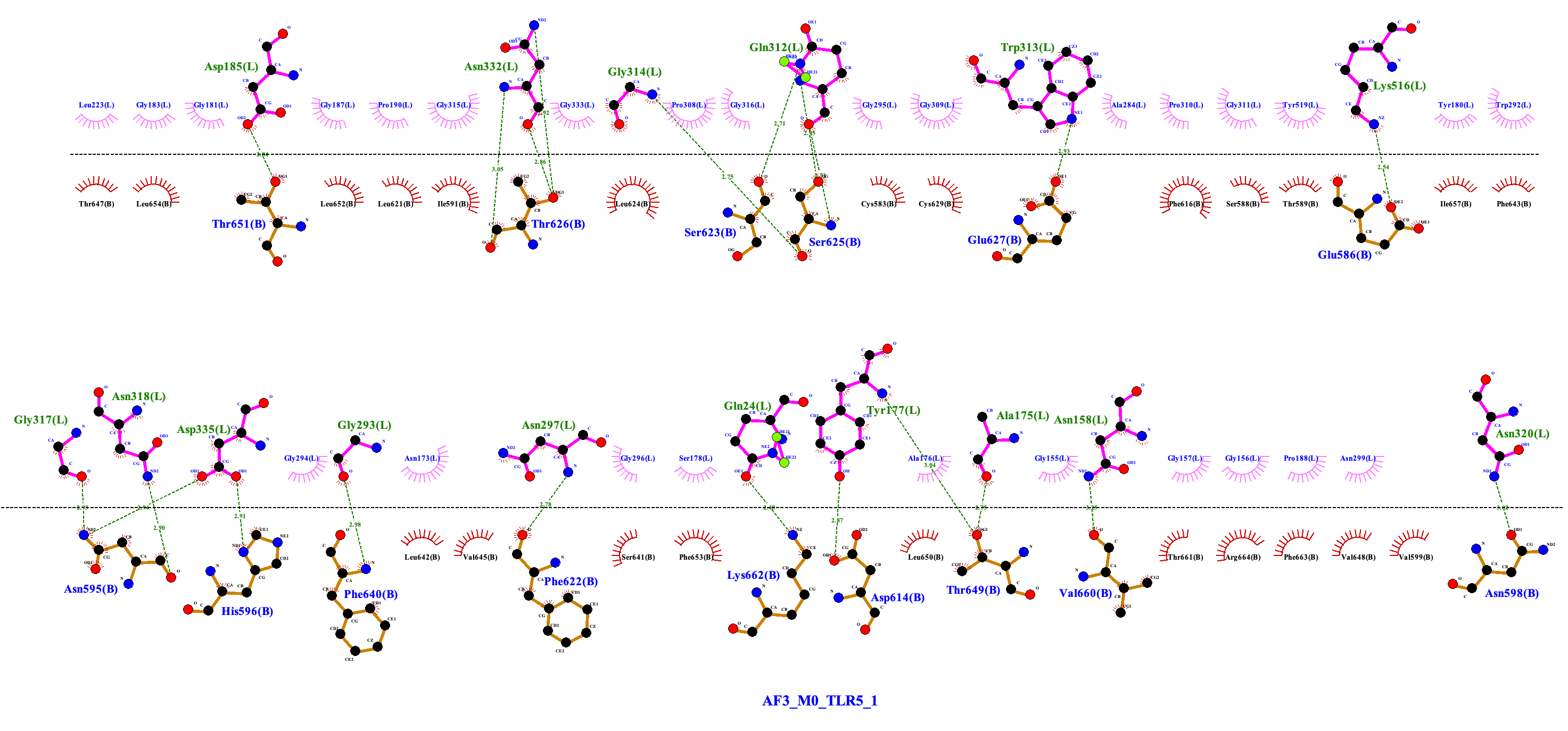


**Figure S2. Ligplot+ representation of interactions between MEV ligand (L) with TLR5 immune receptor.**


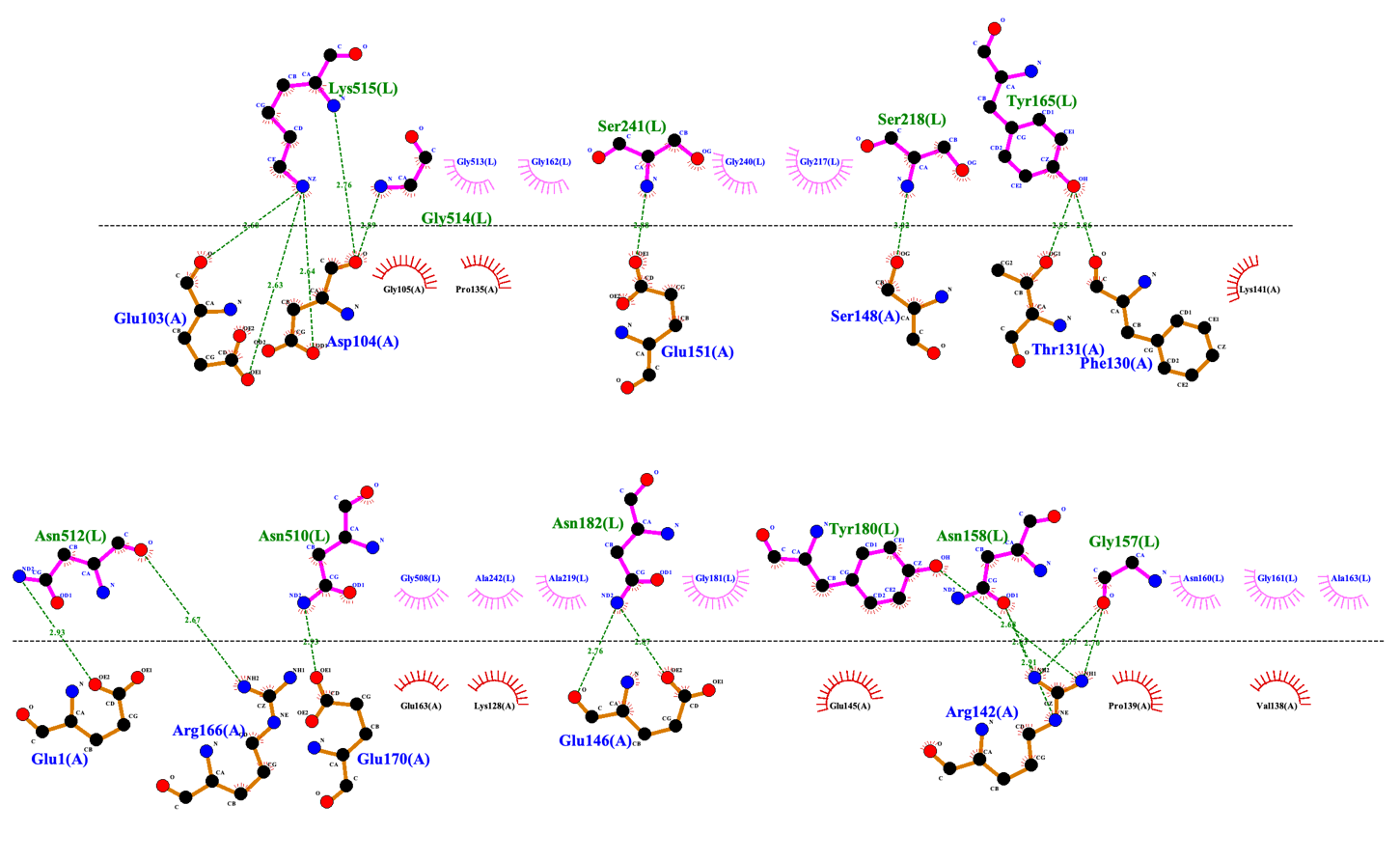


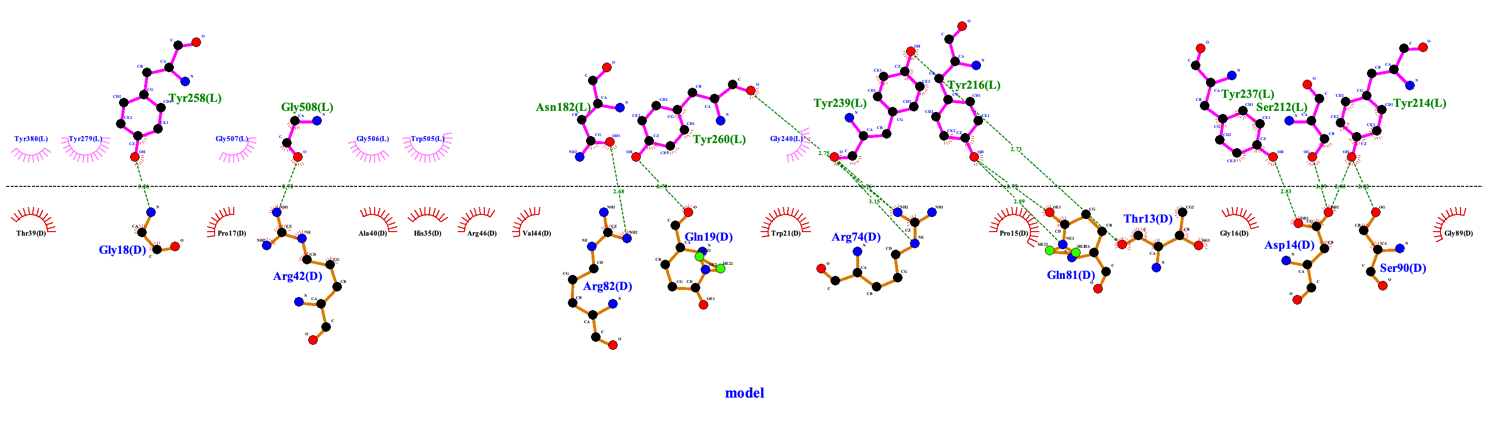


**Figure S3. Ligplot+ representation of interactions between MEV ligand (L) with MHC1 immune receptor.**

**
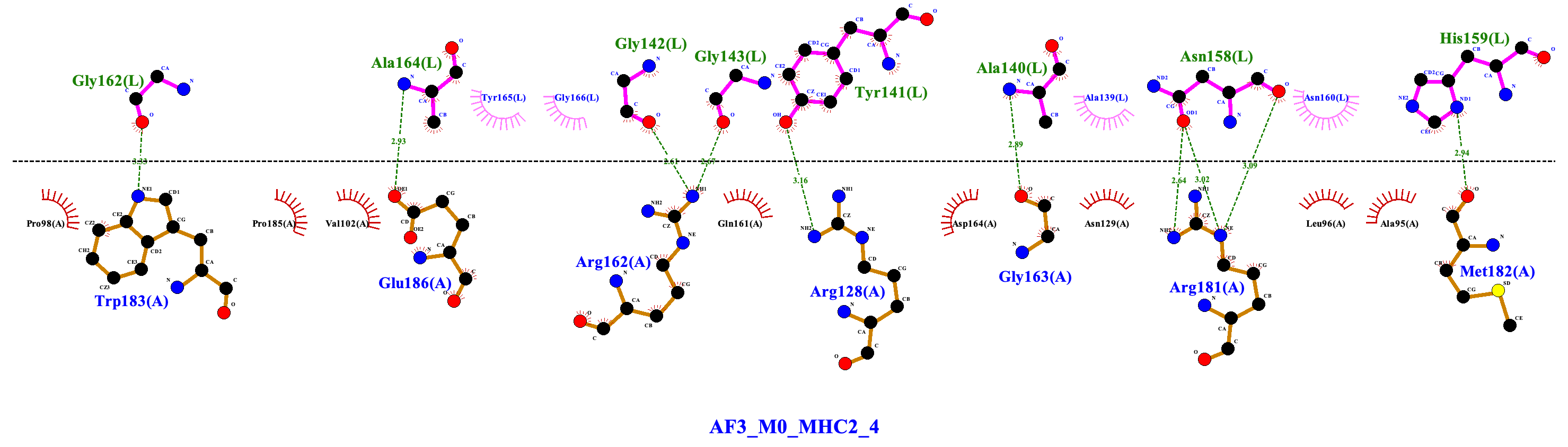
**


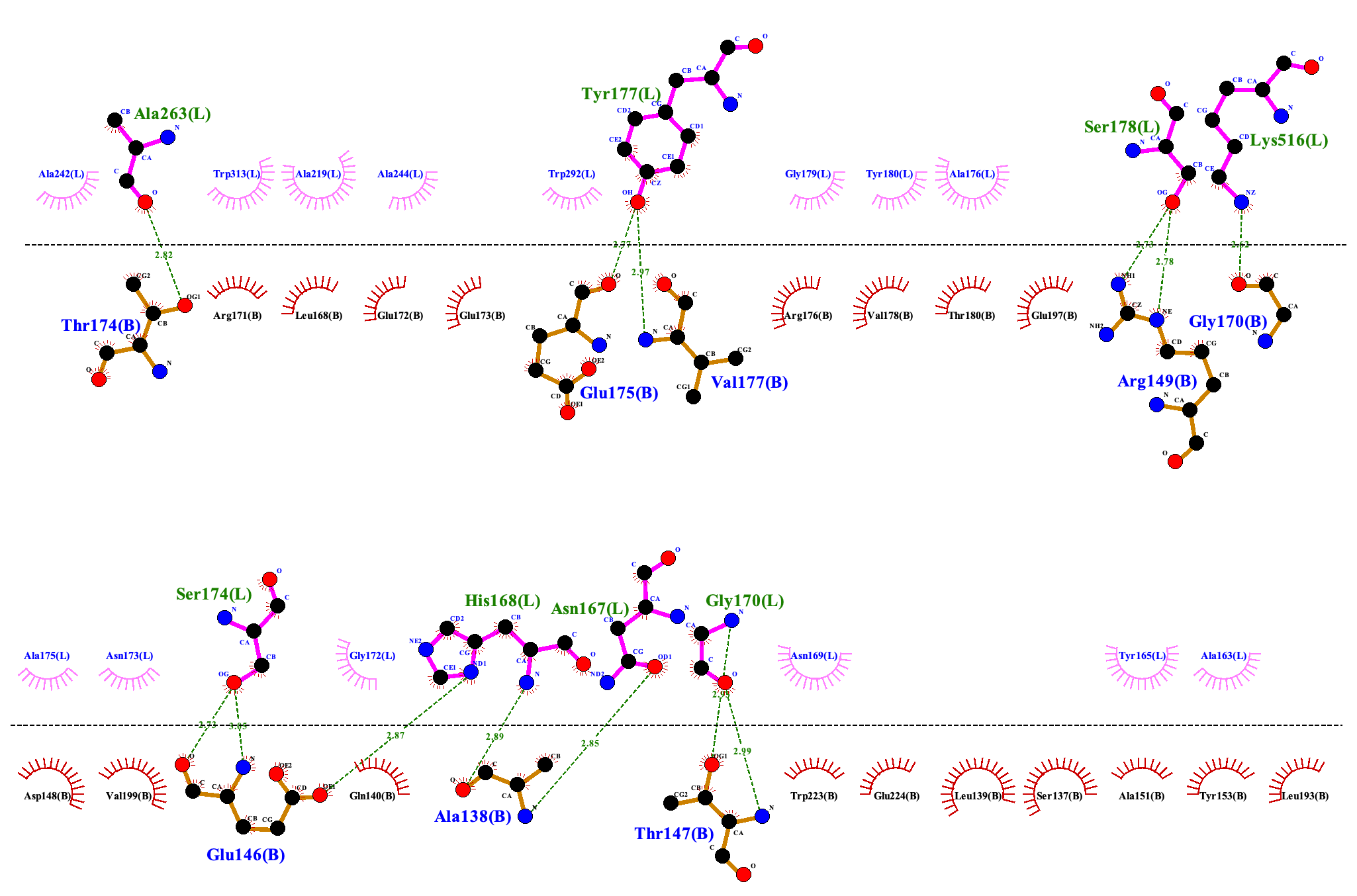


**Figure S4. Ligplot+ representation of interactions between MEV ligand (L) with MHC2 immune receptor.**
